# Phenothiazine Sulfoxides as Active Photocatalysts for the Synthesis of γ-Lactones

**DOI:** 10.1021/jacs.5c01988

**Published:** 2025-04-02

**Authors:** Niklas Hölter, Nils H. Rendel, Leander Spierling, Adrian Kwiatkowski, Roman Kleinmans, Constantin G. Daniliuc, Oliver S. Wenger, Frank Glorius

**Affiliations:** † Organisch-Chemisches Institut, University of Münster, Corrensstraße 36, 48149 Münster, Germany; ‡ Department of Chemistry, University of Basel, St. Johanns-Ring 19, CH-4056 Basel, Switzerland

## Abstract

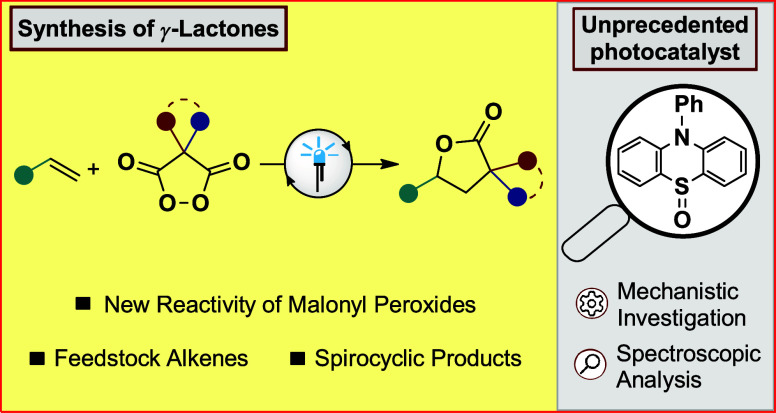

*N*-substituted phenothiazines are prominent and highly effective organic photoredox catalysts, particularly known for their strong reducing capabilities. Despite their wide utility, the closely related phenothiazine sulfoxides, which easily form upon oxidation, have been largely overlooked and have not been explored in the context of photocatalysis. Herein, we describe the discovery and application of *N*-phenylphenothiazine sulfoxide as a photocatalyst for the reductive activation of cyclic malonyl peroxides, giving access to complex γ-lactones starting from simple olefins. Detailed mechanistic studies were carried out to better understand the in situ formation of the active catalyst species from a commercial precursor, as well as the catalyst species interconversion and the photocatalytic mechanism for the formation of γ-lactone products. Specifically, we employed a broad range of mechanistic tools, including time-resolved spectroscopy, spectroelectrochemistry, transient UV–vis absorption spectroscopy, cyclic voltammetry, isotopic labeling, radical trapping experiments, NMR spectroscopy, and density functional theory (DFT) calculations. The synthetic utility of this protocol is demonstrated in a substrate scope study, highlighting the facile access to complex spirocyclic γ-lactones, which are widely recognized for their biological importance.

## Introduction

γ-Lactones are widely occurring structural motifs in natural products, many of them associated with remarkable biological activities, such as anti-inflammatory, anticancer, or antibiotic effects.^1,2^ This has made them a focal point of ongoing research in medicinal chemistry, requiring robust synthetic methodologies to access these structures with complex substitution patterns starting from simple and accessible building blocks.^1,3^ While the most obvious retrosynthetic disconnection is the intramolecular esterification or nucleophilic substitution from 4-hydroxy- or 4-halobutanoic acid derivatives, these reactants are often challenging to access.^2,4^ In contrast, annulation strategies offer a more streamlined method for γ-lactone synthesis. In this regard, α-halocarboxylic acids have been employed as radical precursors under metal-catalyzed^5^ or photocatalyzed^6^ conditions, enabling their transformation into γ-lactones by annulation with olefins as readily available reaction partners.

Inspired by these methodologies, we envisioned that cyclic malonyl peroxides could serve as similar annulation precursors by O–O bond cleavage and extrusion of CO_2_.^7^ These substrates can be easily prepared by the well-established electrophilic functionalization of malonic esters followed by cyclization to the peroxide. Recently, cyclic malonyl peroxides were studied by Tomkinson and co-workers in the context of metal-free dihydroxylation,^8^ oxyamination,^9^ and other dioxygenation reactions.^10^ Moreover, Terent’ev and co-workers showcased several applications of the oxyfunctionalization of enolates^11^ as well as several metal-catalyzed C–H oxyfunctionalization reactions.^12^ Although reports of cyclic malonyl peroxides and their decarboxylative decomposition reactions under UV light date back to the 70s,^13^ to the best of our knowledge, these compounds have never been explored under photocatalytic conditions. In contrast, several examples of the photocatalytic single-electron reduction of acyclic diacyl peroxides have been reported,^14^ prompting us to further investigate this unexplored mode of reactivity of cyclic malonyl peroxides.

**Figure 1:**
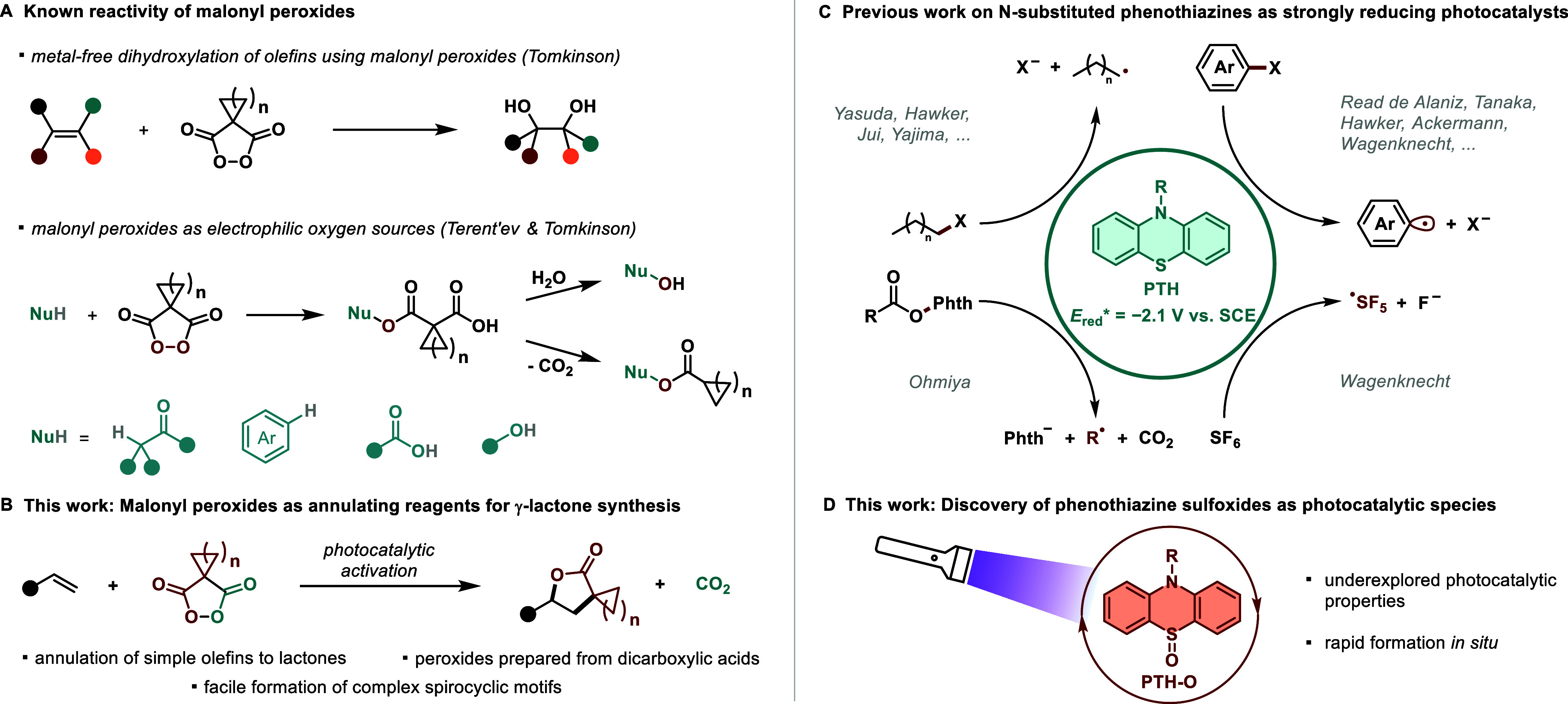
(A) Previous work on malonyl peroxides as a metal-free olefin dihydroxylation reagent and electrophilic oxygen source. (B) Working hypothesis for the use of cyclic malonyl peroxides as radical precursors to form γ-lactones from olefins. (C) *N*-substituted phenothiazines as strongly reducing photocatalysts for the generation of diverse radicals by reductive mesolytic bond cleavage. (D) Photocatalytic properties of phenothiazine sulfoxides are discovered in this work.

During the development of our synthetic methodology, we employed the well-known photocatalyst *N*-phenylphenothiazine (**PTH**).^15^**PTH** possesses a very high excited-state reduction potential of *E*_1/2_(PTH*/**PTH**^**•+**^) = −2.1 V vs SCE^16^ and has been successfully employed for the reductive cleavage of C_aryl_–X,^16,17^ C_alkyl_–X,^18^ N–O,^19−22^ and even S–F bonds (Figure 1C).^23,24^ Notably, radical cations of **PTH** and related phenothiazines are very persistent and have also been studied extensively both as intermediates^19−22,25^ and as photocatalytically active species in two-photon catalytic processes.^23,24,26−28^ In contrast, reports concerning the corresponding phenothiazine sulfoxides remain exceptionally scarce, although their formation under oxidative conditions has been known for a long time.^29−32^ The few existing reports, however, mainly focus on the usage of phenothiazine sulfoxides as reagents for the in situ formation of sulfonium salts and their downstream use in metal-based or electrochemical couplings.^33^ The photophysical properties and possible applications in catalysis, in contrast, seem to be essentially unknown yet. Herein, we showcase the use of *N*-phenylphenothiazine sulfoxide as the active photocatalyst in the synthesis of γ-lactones and elucidate the underlying photophysical processes, catalyst species interconversions, and the product-forming catalytic cycle. Our results thereby disclose important aspects that are relevant for a more detailed understanding of reaction mechanisms based on this catalyst class.

## Results and Discussion

### Reaction Discovery and Optimization

We began our investigation using cyclobutane malonyl peroxide **2a** as a model substrate alongside styrene **1a** as a radical-accepting olefin (Table 1A). Screening of a range of photocatalysts revealed that *N*-phenylphenothiazine (**PTH**) delivered the highest yield of product **3a** (entry 4), while thioxanthone, Ir(ppy)_3_, and [Ir(dF(CF_3_)ppy)_2_(dtbpy)]PF_6_ produced lower yields (entries 1–3). Using **PTH** as an effective organic photocatalyst, we explored the impact of solvents and found that several choices enabled product formation, with MeCN and DCE demonstrating superior performance (entries 4–6).

**Table 1:**
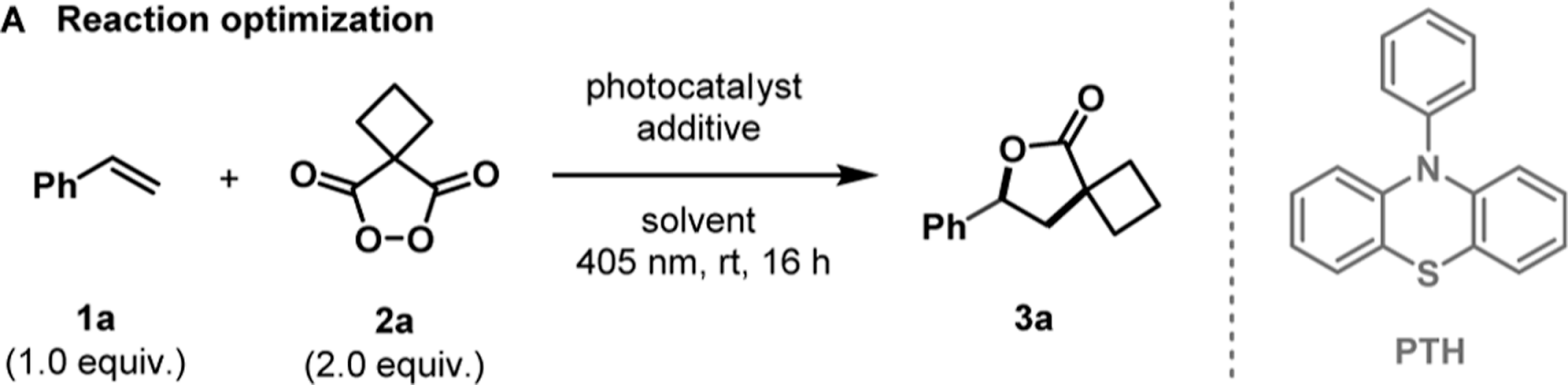

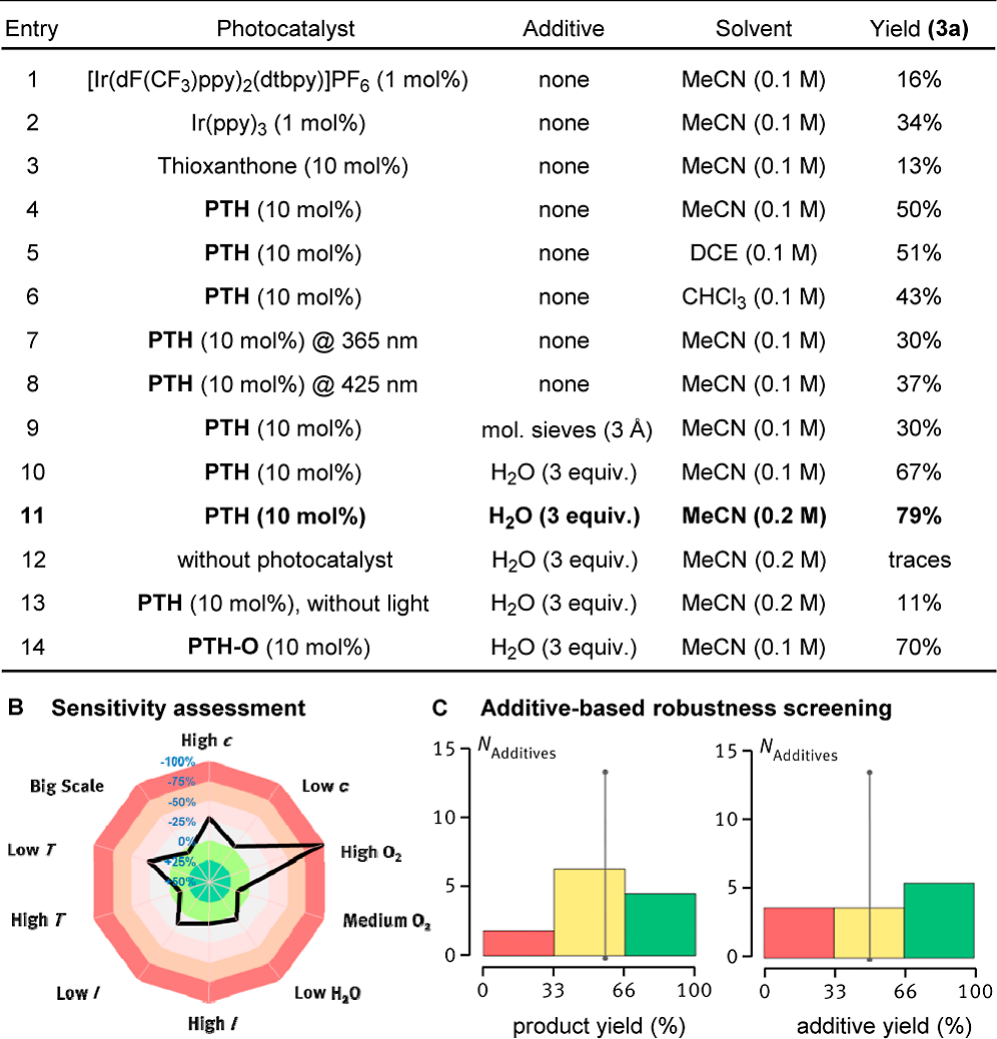
Reaction Optimization and Assessment of Sensitivity and Robustness^a^

a Reactions performed on a 0.1 mmol scale. Yields determined by GC with flame ionization detection (GC–FID) using mesitylene as an internal standard.

We next examined the effects of additives and the operating light sources on the reaction. While most additives did not improve reactivity, the presence of water notably enhanced both the yield and reproducibility of the reaction (entries 9 and 10, see Supporting Information for a detailed discussion). In terms of light sources, wavelengths from 365 to 425 nm promoted product formation, with 405 nm selected as optimal, balancing high yield and milder conditions compared to 365 or 380 nm (entries 7 and 8). Increasing the reaction concentration to *c* = 0.2 M improved the yield to 79%, representing the best condition tested (entry 11). Control experiments confirmed that no product formed in the absence of the photocatalyst (entry 12), while minor levels of product formation were observed in the absence of light (entry 13), likely due to the ground-state reactivity of **PTH** with **2a**, as further elaborated in the mechanistic section. For a more detailed discussion of the reaction optimization, refer to the Supporting Information.

With optimized conditions in hand, we next examined the reaction’s sensitivity to external parameters using our group’s condition-based screening methodology.^34^ Consistent with the optimization results, the reaction demonstrated insensitivity to both water and low levels of residual dissolved oxygen, while exposure to air resulted in a loss of reactivity. Additionally, the screening revealed that the reaction proceeds well under elevated temperatures and high irradiances and can be scaled up without a decrease in performance (Table 1B). To evaluate the reaction’s robustness against functional groups, we employed our additive-based screening procedure and monitored both the product yield and additive consumption in the reaction in the presence of several functional group-bearing additives.^35^ The screening revealed a high tolerance for diverse functional groups, including internal alkynes, nitriles, alcohols, aryl halides, and benzothiazoles. Notably, ketones were tolerated without any observed competing Baeyer–Villiger-type reactivity. In contrast, oxidation-sensitive functional groups, such as unprotected amines, electron-rich pyridines, and aldehydes, were incompatible with the reactions. The extensive list of the results and a detailed discussion can be found in the Supporting Information (Table S8); a summary is presented in Table 1C.

### Investigation of the Reaction Mechanism

**Figure 2:**
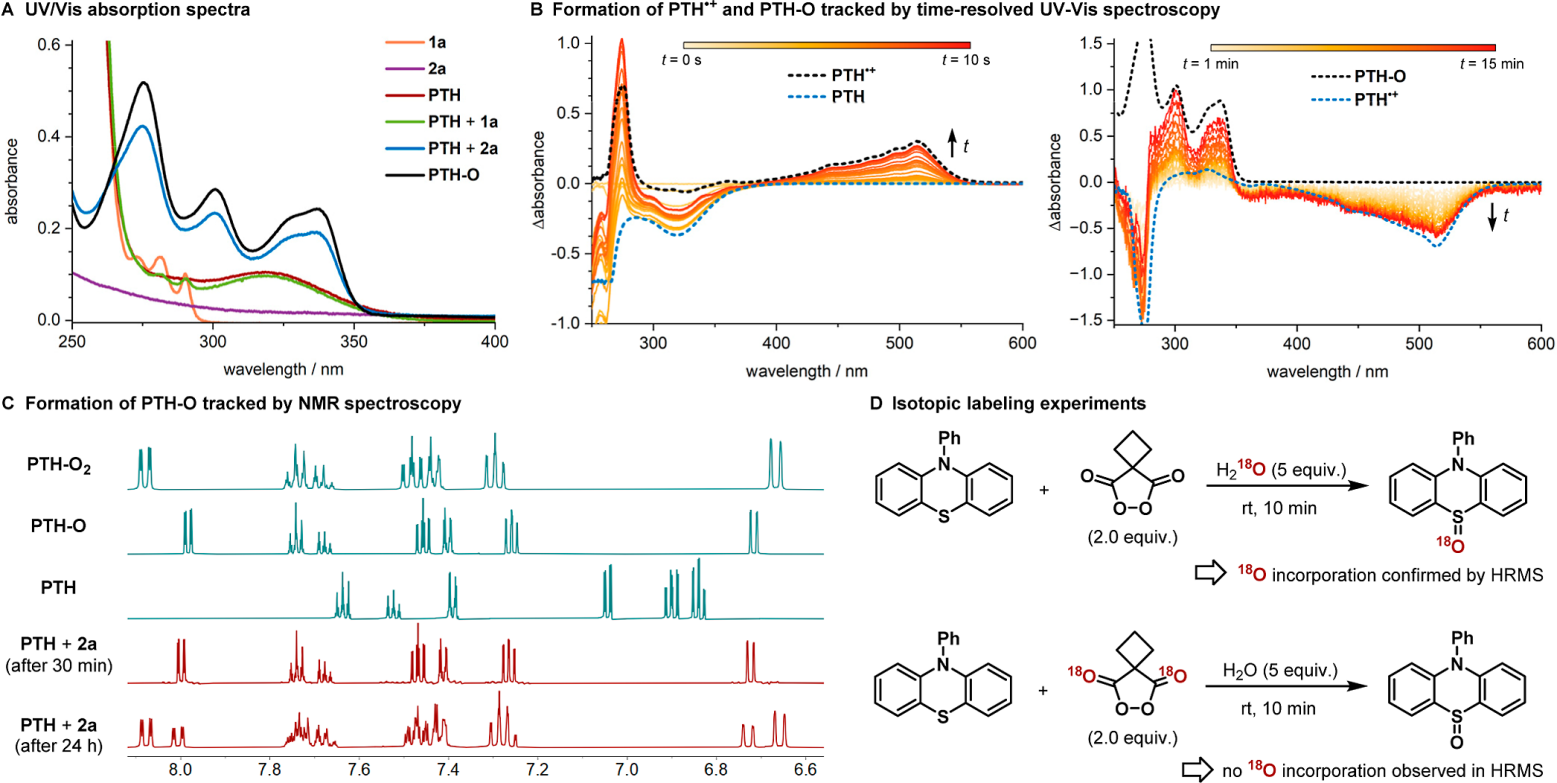
(A) UV/vis absorption spectra of the reaction components and their mixtures. (B) Time-resolved analyses of a **PTH**/**2a** mixture to track the formation of **PTH-O**, proceeding via radical cation **PTH**^•**+**^. (C) Confirmation of **PTH-O** formation and its decomposition to sulfone **PTH-O**_**2**_ by NMR spectroscopy. (D) Isotopic labeling studies to resolve the origin of the **PTH-O** oxygen.

**Figure 3:**
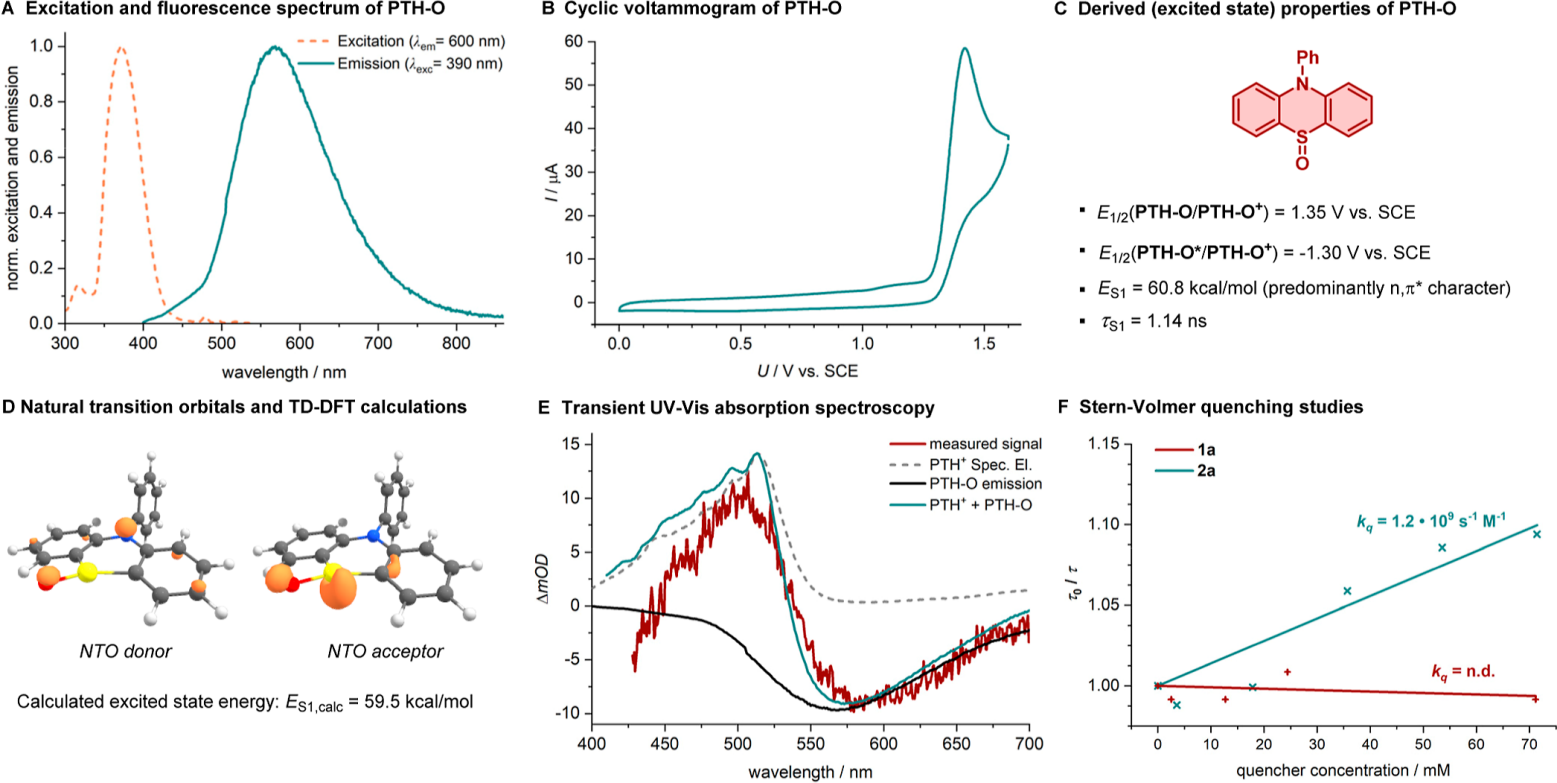
(A) Recorded excitation and emission spectra of **PTH-O**. (B) Cyclic voltammetry studies of **PTH-O**. (C) Derived ground- and excited-state properties. (D) TD-DFT-calculated natural transition orbitals and excited-state energy of the S_1_ excited state of **PTH-O**. (E) Transient absorption spectrum of the reaction mixture. (F) Stern–Volmer lifetime quenching studies.

Having established a synthetic blueprint, we next turned our attention to investigating the underlying mechanism of the reaction. Notably, the LED wavelengths of 405 and 425 nm do not align with the reported absorption spectrum of the **PTH** photocatalyst.^16^ To explore this discrepancy, we recorded UV–vis absorption spectra for the reaction components and their combinations (Figure 2A). While the presence of styrene **2a** had little impact on the absorption spectrum of **PTH**, a distinct and new absorption profile emerged when **PTH** was mixed with malonyl peroxide **2a**. The distinct shape of the absorption profile suggests the formation of a new species rather than an EDA complex. To further probe this new species, we analyzed a mixture of **PTH** and **2a** in the absence of both light and styrene using NMR spectroscopy. Rapid and full conversion of **PTH** to a new, unknown species was observed within 30 min. Furthermore, a continued but slow reaction of this species with an excess of peroxide was observed to yield another unknown compound over the course of 24 h. By comparing the spectra to independently synthesized pure samples, we identified these species as the sulfoxide (**PTH-O**) and sulfone (**PTH-O**_**2**_) derivatives of the photocatalyst (Figure 2C). In addition, UV–vis absorption analyses of the authentic standards revealed that the absorption profile of **PTH-O** matches that of the **PTH**/**2a** mixture (Figure 2A). Furthermore, **PTH-O** shows an excitation maximum in the wavelength range of the operating light source (Figure 3A) and was found to catalyze the reaction effectively (Table 1, entry 14), producing yields comparable to those obtained with **PTH**. In contrast, the sulfone **PTH-O**_**2**_ neither absorbs light at the reaction wavelength range nor does it enable notable product formation when used in place of **PTH**. Therefore, we hypothesize that the overoxidation of **PTH-O** to **PTH-O**_**2**_ represents a possible pathway of catalyst degradation (see Supporting Information for **PTH-O**_**2**_ spectra and detailed discussion).

We next sought to more closely investigate the formation of **PTH-O** within the reaction mixture. Time-resolved UV–vis absorption spectroscopy indicates that rapid single electron transfer (SET) between **2a** (*E*_1/2,calc._ = 0.80 V vs SCE)^36^ and **PTH** (*E*_1/2_ = 0.67 V vs SCE) generates the stable radical cation **PTH**^**•+**^. This species has been extensively characterized^27,28^ and was identified in our studies by in situ HRMS analysis and via its distinctive absorption spectrum (Figure 2B, left). Literature precedence suggests that **PTH**^**•+**^ can either undergo another SET or disproportionation to form the short-lived dication **PTH**^**2+**^, which is subsequently trapped by water to yield the sulfoxide **PTH-O**.^30−32,37^ This hypothesis is supported both by time-resolved spectroscopy, which shows the comparably slow interconversion of **PTH**^**•+**^ into **PTH-O** (Figure 2B, right), and isotopic labeling experiments, which reveal that the oxygen incorporated into **PTH-O** originates from the water additive rather than malonyl peroxide **2a** (Figure 2D). Furthermore, the initial SET also accounts for the product-forming background reaction observed in the absence of light, which showed a linear dependence on the amount of **PTH** employed (see Table S11 for a detailed discussion).

### Characterization and Excited-State Dynamics of the PTH-O Photocatalyst

As there are no prior reports on the use of phenothiazine sulfoxides as photocatalysts, we next turned our attention toward the characterization of their ground- and excited-state properties. Although the overall extinction coefficient is weak in the visible light range (refer Figure S10), a distinct excitation band, ranging from 350 to 425 nm (λ_max,exc._ = 373 nm), was observed (Figure 3A). Considering the extinction coefficient of ε_405nm_ = 42 M^–1^ cm^–1^, the short excited-state lifetime of τ = 1.14 ns (determined by TCSPC), and the TD-DFT-calculated natural transition orbitals (Figure 3D), we hypothesize that the corresponding excited state is a singlet state with predominant *n*,π* character. Based on the fluorescence onset, the experimental excited-state energy was approximated as *E*_S1_ = 60.8 kcal/mol. Combined with a ground-state oxidation potential of *E*_1/2_(**PTH-O**/**PTH-O**^**•+**^) = 1.35 V vs SCE, obtained by cyclic voltammetry (Figure 3B), the excited-state oxidation potential is determined as *E*_1/2_(PTH-O*/**PTH-O**^**•+**^) = −1.30 V vs SCE (Figure 3C).

To investigate the excited-state dynamics of **PTH-O** upon 405 nm irradiation, a stock solution of **PTH** (0.5 mM), **2a** (10 mM), **1a** (5 mM), and H_2_O (15 mM) in MeCN was prepared. Upon mixing, the solution was left for 15 min until the red color from the **PTH**^**•+**^ disappeared and then irradiated with a 405 nm laser in a picosecond transient UV–vis absorption setup. The resulting transient absorption spectrum shows two distinct features (Figure 3E). The first resembles the ground-state absorption of **PTH**^**•+**^, which can either result from the formation of this species due to incomplete ground-state reactivity of **PTH** with **2a** or by photocleavage of the S–O bond of **PTH-O** at high irradiances.^32^ Any fluctuations in the concentration of **PTH**^**•+**^ become immediately visible due to its high extinction coefficient. The second feature is a stimulated-emission-bleach of **PTH-O** between 525 and 650 nm. Together, this advocates for **PTH-O** as the active photocatalyst since the transient absorption data reveals PTH-O* as the main excited-state species upon 405 nm irradiation.

Evidence of the role of **PTH-O** is further strengthened by Stern–Volmer lifetime quenching studies. The photoluminescence lifetime of excited-state **PTH-O** was recorded upon addition of **2a** and **1a** (with concentrations increasing from 3 to 71 mM, approaching the solubility limit of **2a**). The obtained data revealed an experimental quenching constant of *k*_q_(**2a**) = 1.2 × 10^9^ M^–1^ s^–1^ for **2a**, whereas no quenching was observed upon the addition of styrene **1a** (Figure 3F). The initial photocatalytic elementary step after excitation of **PTH-O** with 405 nm light is therefore most likely a SET to **2a**. An alternative quenching pathway based on triplet–triplet energy transfer^38^ seems implausible due to the short luminescence lifetime of **PTH-O**, which is also not quenched by oxygen. Additionally, no dark triplet state could be observed in transient UV–vis absorption spectroscopy. Lastly, a high dynamic vertical triplet energy^39^ of *E*_T,DvTE_ = 73.2 kcal/mol was calculated for peroxide **2a**, further contradicting the feasibility of triplet–triplet energy transfer.

### Proposed Reaction Mechanism and Catalytic Cycle

**Figure 4:**
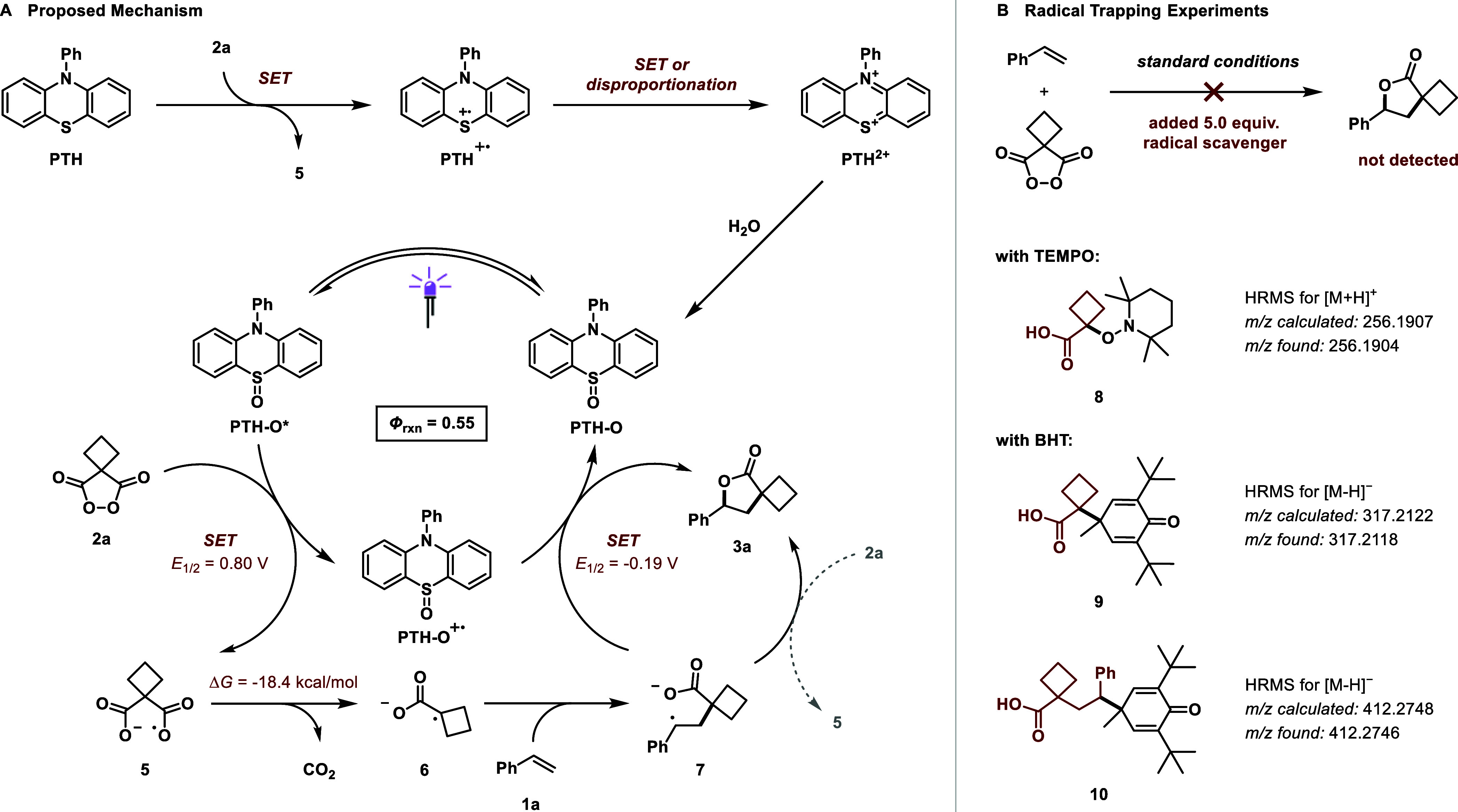
(A) Proposed reaction mechanism for the formation of **PTH-O** and the subsequent catalytic cycle. (B) Radical trapping experiments using TEMPO and BHT to detect intermediate radicals.

With the necessary information about the photocatalyst and its primary photoreactivity in hand, we evaluated the full reaction mechanism and catalytic cycle (Figure 4A). The identified key step for the formation of lactone **3a** is the reductive cleavage of the peroxide bond of **2a** by excited-state PTH-O*, which is thermodynamically feasible (Δ*E* = 2.10 V) and supported by Stern–Volmer quenching studies (Figure 3F). The resulting radical anion **5** undergoes rapid decarboxylation (Δ*G* = −18.4 kcal/mol) to form the more stable carbon-centered radical **6**, which was detected in radical trapping studies using both TEMPO and BHT (Figure 4B, compounds **8** and **9**). These radical trapping studies also support the addition of **6** into styrene **2a**, yielding benzylic radical **7**, which could be trapped with BHT (Figure 4B, compound **10**). This stabilized radical (*E*_1/2,calc._ = −0.19 V) can then undergo oxidative radical-polar crossover (ORPC) with both the oxidized photocatalyst species **PTH-O**^**•+**^ to turn over the catalytic cycle or with **2a** to propagate a chain reaction. While chain propagation between **2a** and **7** is thermodynamically feasible and also accounts for the observed product formation in the dark, the reaction quantum yield was found to be small with a value of *Φ* = 0.55, indicating that potential chain propagation would be rather inefficient. In any case, the resulting benzylic cation cyclizes immediately with the carboxylate to form product **3a**.

### Reaction Scope Investigation

**Scheme 1:**
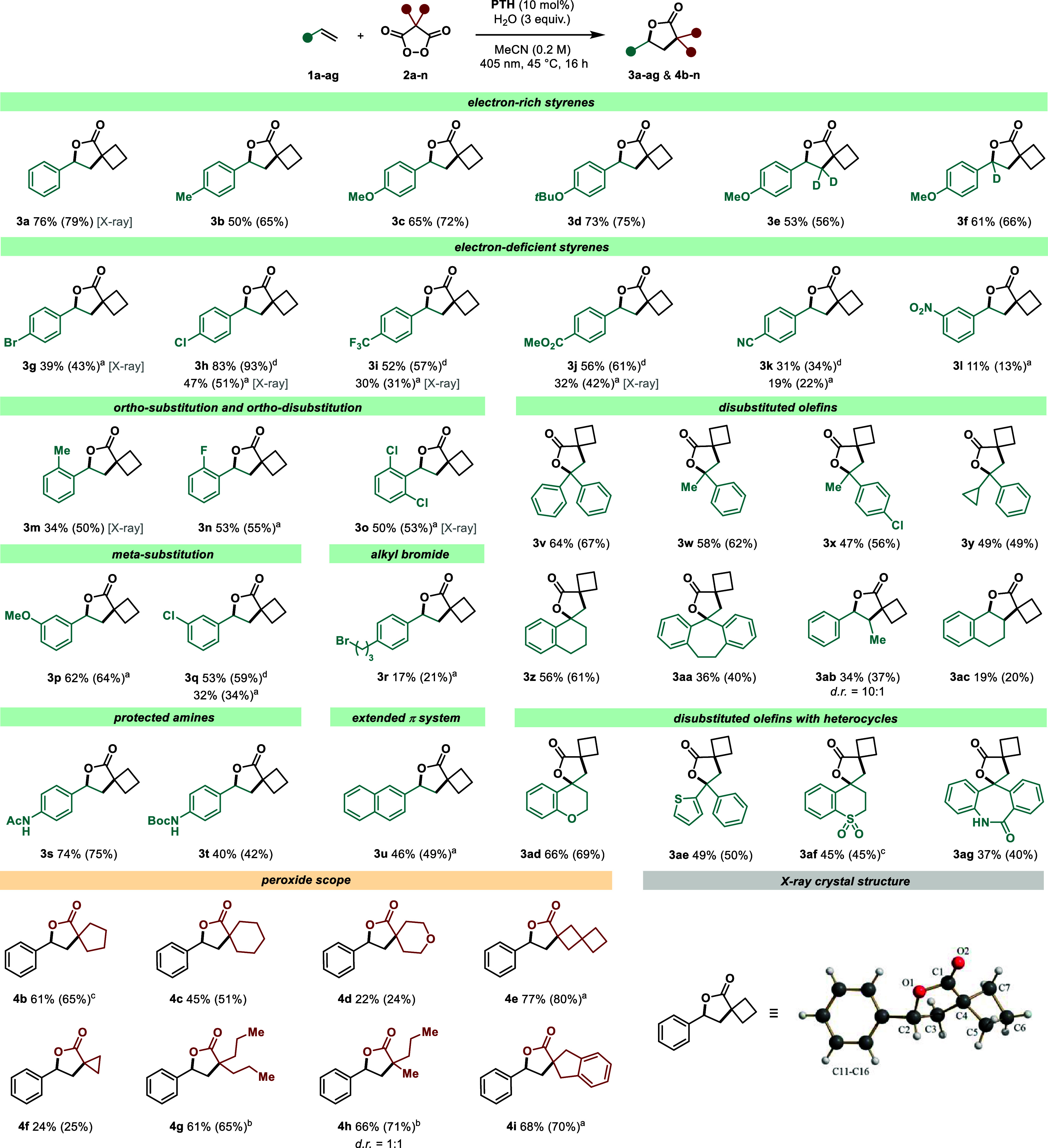
Substrate Scope Investigation Reactions were performed on a 0.25 mmol scale using 2.0 equiv of malonyl peroxide, 3.0 equiv of water, and 10 mol % **PTH** in MeCN (0.2 M). Brackets indicate yields of the crude reaction mixture determined by GC/Polyarc-FID using mesitylene as an internal standard. ^a^Reaction was performed in DCE (0.2 M). ^b^Reaction was performed using 5.0 equiv of peroxide. ^c^Reaction was performed using 4.0 equiv of peroxide. ^d^**2-MeO-PTH** was used instead of **PTH**.

After having established a mechanistic proposal for the discovered reaction, we turned our attention toward the investigation of the reaction’s substrate scope (Scheme 1). Notably, a variety of substituted styrenes were successfully transformed into the corresponding γ-lactone products. Consistent with our proposed ORPC-based mechanism, electron-rich styrenes bearing *para*-alkyl (**3b**) or *para*-alkoxy (**3c** and **3d**) substituents were generally producing excellent yields ranging from 50% to 80% in shorter reaction times. The same applies for a broad range of 1,1-disubstituted olefins (**3v**–**3aa** and **3ad**–**3ag**), which also form stabilized carbocations and almost consistently give product yields of above 50%. However, 1,2-disubstituted olefins resulted in diminished reactivity and lower yields of 34% (**3ab**) and 19% (**3ac**), likely due to an increased steric demand for the radical attack step.

In contrast to electron-rich styrenes, lower yields ranging from 19% to 47% were obtained for electron-deficient substrates, for example, those bearing a CF_3_-(**3i**), ester (**3j**), or nitrile (**3k**) group in the *para* position. Similarly, *meta*-nitro- and *meta*-chloro-substituted products **3l** and **3q** were obtained only with yields of 11% and 32%, respectively. For most of these challenging, electron-deficient substrates, higher yields (e.g., 83% for **3h**, 52% for **3i**, 56% for **3j**, and 53% for **3q**) were obtained by switching to **2-MeO-PTH** as a catalyst derivative, which likely forms a more stable active sulfoxide. Several halogen substituents including fluorine (**3n**), chlorine (**3h**, **3o**, **3q**, and **3x**), and bromine (**3g**) in various positions other than *meta* did not notably alter the yield and provided the potential for the facile further functionalization of the product motifs using established cross-coupling chemistry. Also, simple *ortho*-substitutions (**3m** and **3n**) and *ortho*-disubstitutions (**3o**) did not result in a decrease in yield. The compatibility of *N*-acetyl- and *N*-Boc-protected amines was demonstrated in entries **3s** and **3t**, which were isolated in 74% and 40% yields, respectively. Moreover, extended π-systems such as naphthalene, which could potentially interfere with the photocatalyst’s absorption, are tolerated, with the corresponding product (**3u**) being isolated in 46% yield. Pleasingly, various heterocyclic systems such as chromanes (**3ad**, 66%), thiophenes (**3ae**, 49%), thiochromane sulfones (**3af**, 45%, starting from thiochromane), and azepan-2-ones (**3ag**, 37%) were successfully incorporated into the lactone products. Next, we turned our attention toward the investigation of the malonyl peroxide scope. In addition to cyclobutane malonyl peroxide **2a**, other derivatives with larger spiro-cyclic rings such as cyclopentane and cyclohexane were successfully transformed into oxaspiro[4.4]nonane-1-one **4b** (61%) and oxaspiro[4.5]decane-1-one **4c** (45%). Also, product **4e** bearing two spirocenters was obtained in high yield (77%). In contrast, for a tetrahydropyrane-based peroxide, the obtained yield for product **4d** decreased to 22%. Similarly, with a spiro-cyclopropane ring in the backbone, a decrease in yield was observed for oxa-spiro-[2.4]heptan-4-one **4f** (24%), which is likely attributed to the low stability of the intermediate cyclopropyl radical with almost sp^2^-hybridization.^12^ Pleasingly, nonspirocyclic malonyl peroxides could also be successfully transformed into the lactone products **4g** and **4h** with 61% and 66% yields, respectively. A spirocyclic indane-derived lactone product **4j** was obtained in 68% yield, with no competing benzylic oxidation observed. For eight of the reported substrates, we report solid-state crystal structures to confirm the product identity and regioselectivity (see Supporting Information for details).

## Conclusions

To summarize, we have discovered that the widely applied photocatalyst *N*-phenylphenothiazine can be oxidized in situ in the presence of water, its corresponding sulfoxide, which shows unprecedented photocatalytic activity. The mechanism of its formation, as well as its ground- and excited-state properties and excited-state dynamics, was extensively investigated by various spectroscopic techniques, isotopic labeling and NMR experiments, and (TD-)DFT calculations. Using this catalyst, a novel reactivity of cyclic malonyl peroxide was discovered, which allows the facile synthesis of complex spirocyclic γ-lactones from simple activated olefins. Based on the investigated photocatalyst properties and additional mechanistic experiments, we studied the catalytic cycle for the formation of γ-lactone products. As the key mechanistic step of the reaction, we identified the mild single-electron reduction of the peroxide bond, yielding a reactive carbon-centered radical after decarboxylation. The scope of this transformation includes a variety of activated olefins and peroxides, with many functional groups such as protected amines, esters, aryl halides, and several S- and O-heterocycles being tolerated.

We anticipate that this work will serve as a reference for future mechanistic investigations of reactions, including phenothiazine photocatalysts under oxidizing conditions, highlighting the possibility of generating other catalytically active species in situ. Furthermore, the discovered activation mode of cyclic peroxides holds the potential to harness these substrates and related substrate classes as structurally useful synthons in other reactions.

## Supporting Information Available

All experimental procedures, characterization data, additional mechanistic discussions, and computational details (PDF)

## Abbreviations

MeCN: acetonitrile
DCE: 1,2-dichloroethane
BHT: 2,6-di-*tert*-butyl-4-methylphenol
GC: gas chromatography
FID: flame ionization detection
MS: mass spectrometry
NTO: natural transition orbital
ORPC: oxidative radical-polar crossover
PTH: phenothiazine
SCE: standard calomel electrode
TCSPC: time-correlated single photon counting
TD-DFT: time-dependent density functional theory
TEMPO: (2,2,6,6-tetramethylpiperidin-1-yl)oxyl
