# Supplementary material for: Phenothiazine Sulfoxides as Active Photocatalysts for the Synthesis of γ-Lactones
Source: J Am Chem Soc. 2025 Apr 2;147(15):12908–16. doi: 10.1021/jacs.5c01988 (PMC12007001; doi:10.1021/jacs.5c01988)
Supplement: Supplementary file 1 — ja5c01988_si_001.pdf [file ja5c01988_si_001.pdf]

*Supplementary Information*

# Phenothiazine Sulfoxides as Active Photocatalysts for the Synthesis of γ-Lactones

Niklas Hölter,[1, ‡] Nils H. Rendel,[1, ‡] Leander Spierling,[2] Adrian Kwiatkowski,[1] Roman Kleinmans,[1] Constantin G. Daniliuc,[1] Oliver S. Wenger,[2,*] Frank Glorius[1,*]

[1]Organisch-Chemisches Institut, University of Münster, Corrensstraße 36, 48149 Münster, Germany.

[2]Department of Chemistry, University of Basel, St. Johanns-Ring 19, CH-4056 Basel, Switzerland.

[‡]These authors contributed equally.

*Correspondence to: oliver.wenger@unibas.ch; glorius@uni-muenster.de

# TABLE OF CONTENTS

1. GENERAL EXPERIMENTAL ........ 3
1.1. Glassware, Solvents and Reagents ........ 3
1.1.1. Photochemical set-up and light sources ........ 3
1.2. Analytical Techniques ........ 5
1.2.1. Thin layer chromatography ........ 5
1.2.2. NMR spectroscopy ........ 5
1.2.3. Mass spectrometry ........ 5
1.3. Naming of Compounds ........ 6
1.4 Safety ........ 6
2. EXPERIMENTAL DATA ........ 7
2.1. General Procedures ........ 7
2.1.1. General Procedure A: Synthesis of $\gamma$-Lactones from malonylperoxides and olefins ........ 7
2.1.2. General Procedure B: Synthesis of malonylperoxides ........ 7
2.1.3. General Procedure C: Synthesis of dicarboxylic acid ........ 8
2.1.4. General Procedure D: Synthesis of olefines ........ 8
2.2. Synthesis of Starting Material ........ 9
2.2.1. Preparation of Malonyl Peroxides ........ 9
2.2.1.1. Synthesis of Dicarboxylic Acid ........ 9
2.2.1.2. Synthesis of Malonyl Peroxides ........ 12
2.2.2. Synthesis of Olefines ........ 18
2.3. Synthesis of Photocatalyst ........ 24
2.4. Reaction Optimization ........ 28
2.4.1. Establishing the photocatalyst ........ 28
2.4.2. Investigation of the solvent ........ 29
2.4.3. Optimization of the stoichiometry ........ 29
2.4.4. Investigation of the influence of water ........ 30
2.4.5. Screening of light sources and additives ........ 30
2.4.6. Screening of photocatalyst for electron-deficient styrenes ........ 31
2.5 Synthesis of $\gamma$–Lactones ........ 33
2.6. Substrate Limitations ........ 55
2.7. Sensitivity Screen ........ 56
2.8. Additive-based Robustness Screen ........ 58
3. MECHANISTIC INVESTIGATIONS ........ 61
3.1. UV/vis absorption spectroscopy ........ 61
3.2. Steady-state emission spectroscopy ........ 62
3.3. Lifetime measurements and Stern-Volmer analysis ........ 63
3.4. Cyclic Voltammetry and spectroelectrochemistry ........ 64
3.4.1. Cyclic voltammetry ........ 64

3.4.2. Spectroelectrochemistry and time-resolved absorption spectra ....64
3.5. Transient absorption measurements ....65
3.6. Trapping Experiments ....67
3.7. Isotopic labelling experiments ....68
3.8. Dark experiments ....73
3.9. Reactivity of different photocatalyst species ....74
3.10. Quantum yields measurement ....75
3.11. Check for dihydroxylation reactivity ....77

4. COMPUTATIONAL STUDIES .... 79
4.1. Density functional theory ....79
4.1.1. Decarboxylation of 2a ....79
4.1.2. Calculation of redox potentials ....80
4.1.3. Calculation of natural transition orbitals by TD-DFT ....82
4.2. Dynamic vertical triplet energies ....84
4.2.1. Optimized geometries for the MD simulation initialization ....84
4.2.2 Vertical triplet energy distributions and DvTEs ....86

5. X-RAY CRYSTALLOGRAPHY .... 88
5.1. X-Ray Diffraction Data ....88

6. ACKNOWLEDGEMENTS .... 97

7. SPECTROSCOPIC DATA .... 98

# 1. GENERAL EXPERIMENTAL

## 1.1. Glassware, Solvents and Reagents

All reactions were conducted under an inert atmosphere of argon using Schlenk manifold techniques unless stated otherwise. All glassware and Teflon-coated magnetic stir bars were dried in an oven at 80 °C prior to use. All anhydrous solvents were commercially supplied and stored over 3 Å mol. sieves or dried using an activated alumina column drying system (MeCN, $CH_2Cl_2$, hexane, toluene, THF, $Et_2O$, DMF, MeOH). Reagents were purchased from commercial sources and used as received.

### 1.1.1. Photochemical set-up and light sources

Unless otherwise stated, photochemical reactions were performed in a Hepatochem EvoluChemTM PhotoRedOx Box Duo device and irradiated with two EvoluChem™ HCK1012-02-010 LEDs (18 W each, $\lambda_{max}$ = 405 nm) or HCK1012-01-008 (30 W each, $\lambda_{max}$ = 450 nm). Detailed emission spectra and details regarding the light sources can be found in Figure S2 and S3 as well as on the website of the manufacturer (https://hepatochem.com/photoreactors-leds-accessories/photoreactor-leds-evoluchem/) With internal fan turned on, the reaction temperature was determined between 30 °C and 35 °C.

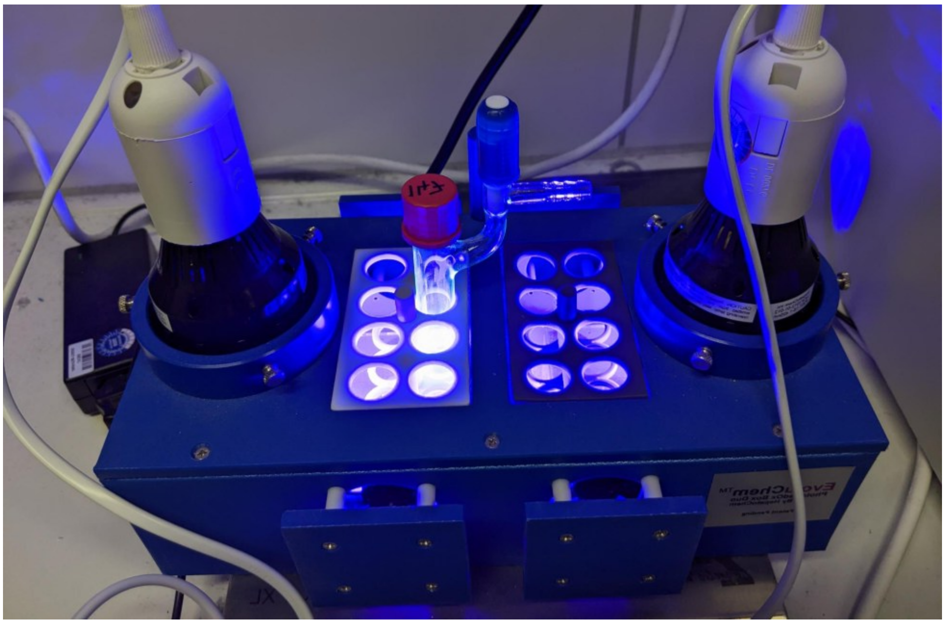

**Figure S1**: Experimental set-up for photochemical reactions.

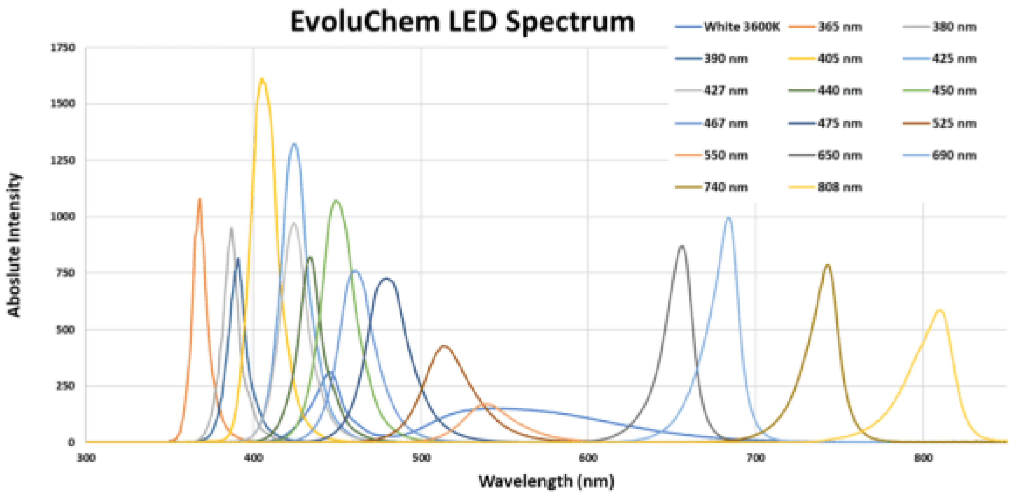


**Figure S2**: Emission spectra (not normalized) as given by the manufacturer.[1]

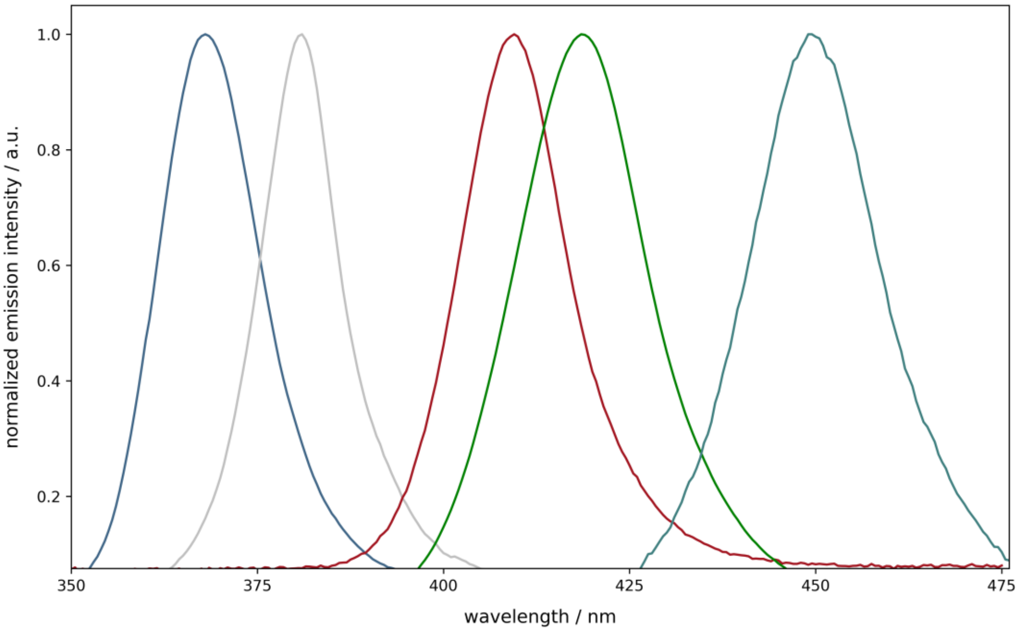


**Figure S3**: Emission spectra (normalized) of utilized LED light sources.

blue: 365 nm, gray: 380 nm, red: 405 nm, green: 425 nm, teal: 450 nm

## 1.2. Analytical Techniques

### 1.2.1. Thin layer chromatography

Thin layer chromatography (TLC) was performed to monitor reactions when practical using Merck silica gel 60 $F_{254}$ aluminum plates and visualised under UV light, or by staining with aqueous basic potassium permanganate or phosphomolybdic acid followed by heating.

### 1.2.2. NMR spectroscopy

Spectra were recorded on a Bruker Avance II 400, Agilent DD2 500 or DD2 600 spectrometers. All spectral data was acquired at 295 K. Deuterated solvents were purchased from Eurisotop ($CDCl_3$, deuteration > 99.8%, $CD_3CN$ deuteration > 99.8%, DMSO-$d_6$ deuteration > 99.8%). Chemical shifts ($\delta$) are reported in parts per million (ppm) and referenced to $CDCl_3$ ($^1$H: 7.26 ppm; $^{13}$C: 77.16 ppm), to $CD_3CN$ ($^1$H: 1.94 ppm; $^{13}$C: 118.3 ppm), to DMSO-$d_6$ ($^1$H: 2.50 ppm; $^{13}$C: 39.5 ppm). Coupling constants (*J*) are given in Hertz (Hz) and refer to corresponding multiplicities (s = singlet, d = doublet, t = triplet, q = quartet, quin = quintet, hex = hextet, h = heptet, m = multiplet, app = apparent, br. = broad signal, dd = doublet of doublets, etc.). The $^1$H NMR spectra are reported as follows: chemical shift (multiplicity, coupling constants, number of protons). NMR assignments were made according to spin systems, using two-dimensional NMR spectroscopy (COSY, HSQC, HMBC) to assist the characterisation. NMR yields were determined by $^1$H NMR analysis using dibromomethane as an internal standard. The *d.r.* and *r.r.* values were determined by $^1$H NMR analysis of the crude reaction mixture. When only a single regioisomer was detected, no *r.r.* is given. >20:1 *d.r.* indicates when only a single diastereomer could be detected.

### 1.2.3. Mass spectrometry

**High resolution mass spectra (HRMS)** were carried out by the MS service division of the Institute of Organic Chemistry at the University of Münster. All spectra were measured on a Bruker Daltonics MicroToF, Thermo Fisher Scientific Orbitrap LTQ XL, Thermo Fisher Scientific Exploris 120 Electrospray Orbitrap or Thermo Fisher Scientific Orbitrap Velos Pro mass spectrometer using electrospray ionization (ESI).

**GC-MS** samples were filtered over a pad of silica and eluted with EtOAc before analysis. GC-MS chromatograms were recorded on an Agilent Technologies 7890A GC-system (HP-5MS column: 0.25 mm × 30 m, film: 0.25 µm) with an Agilent 5977B Mass Selective Detector (MSD).

**GC-FID** measurements were performed on Agilent Technologies 7890A GC-system (HP-5MS column: 0.25 mm × 30 m, film: 0.25 µm) equipped with a Polyarc® system (connected to an Aux. EPC and a Thermal Aux. Zone) and a flame ionization detector (FID). Quantitative analysis was performed using mesitylene as an internal standard. Reaction yields were calculated as the ratio of the carbon-normalized peak areas of an analyte (*a*) and the standard (*s*):

$$Yield\ (\%) = \frac{Area_{\mathrm{a}}/Carbon\ Count_{\mathrm{a}}}{Area_{\mathrm{s}}/Carbon\ Count_{\mathrm{s}}} \cdot 100\% \qquad (1)$$

For more details on the quantification workflow, refer to:

Katzenburg, Glorius et al., *Digital Discovery*, **2025**, 4, 384-392 (https://doi.org/10.1039/D4DD00347K)

### 1.3. Naming of Compounds

Compound names are those generated by ChemDraw Professional 23.1.1 software (PerkinElmer), following the IUPAC nomenclature.

### 1.4 Safety

Peroxides can be particularly hazardous and tend to violent decomposition reactions. Work with peroxides should therefore be carried out by experienced laboratory personnel under special precautionary measures. Differential scanning calorimetric (DSC) measurements of some representative peroxides are appended in the starting material synthesis section of this document. DSC data was recorded and appended to the characterization data, exothermic processes are shown as positive heat flow values, whereas endothermic processes are represented by negative values. Even if no violent exothermic decomposition was observed for the peroxides presented in this manuscript, care should be taken due to potential instabilities upon shock or electrostatic charging.

# 2. EXPERIMENTAL DATA

## 2.1. General Procedures

### 2.1.1. General Procedure A: Synthesis of γ-Lactones from malonylperoxides and olefins

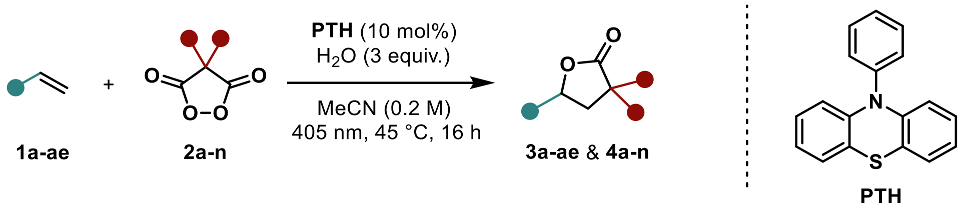


To an oven-dried 10 mL Schlenk tube equipped with a Teflon-coated magnetic stir bar was added PTH (10 mol%). The Schlenk tube was evacuated and backfilled with argon three times before MeCN (0.2 M) was added under a positive argon pressure. The respective olefin **2** (1.0 equiv.), water (3.0 equiv.). While stirring, solid malonyl peroxide (2.0 equiv. – 5.0 equiv.) was added and the mixture turned dark red. After roughly 1 min, the reaction mixture became clear and yellowish, was immediately placed in a photoreactor and irradiated for 24 h with blue LEDs (405 nm, 18 W).[**A**] After this time, the solvent was removed under reduced pressure and the crude product was purified by flash column chromatography on silica gel to yield the corresponding γ-Lactones (**3**&**4**).[B]

Notes: (**A**) No fan cooling was used during irradiation, allowing the reaction vessel to reach temperatures of approximately 45 °C.

(**B**) The product is often not visible on the TLC plate using UV. The only stain that worked was a freshly prepared phosphomolybdic acid stain.

### 2.1.2. General Procedure B: Synthesis of malonylperoxides

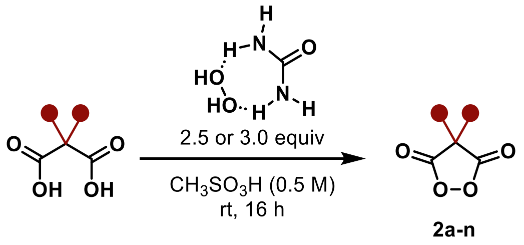


The product was synthesized following a adapted literature procedure.[2] Urea-hydrogen peroxide adduct (2.5 or 3.0 equiv.) was dissolved in non-aqueous methanesulfonic acid (0.5 M) in an oven-dried round-bottomed flask cooled with a water bath at room temperature. The 1,1-dicarboxylic acid (1.0 equiv.) was then added in one portion and the mixture was stirred overnight. The reaction mixture was then poured into a separatory funnel containing ice and EtOAc (50 mL per 10 mmol), extracted and phases were separated. The organic layer was washed with sat. aq. $NaHCO_3$ solution (2x 50 mL per 10 mmol), sat. aq. NaCl solution (50 mL per 10 mmol) and dried over $Na_2SO_4$. Removal of the solvent under reduced pressure gave the pure diacyl peroxide.

### 2.1.3. General Procedure C: Synthesis of dicarboxylic acid

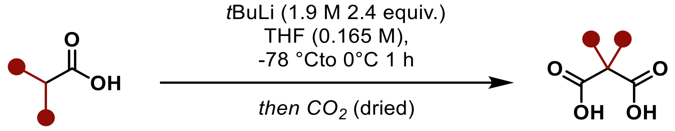


The product was synthesized following a adapted literature procedure.[3] Carboxylic acid (1.0 equiv.) and THF (0.165 M) were added to an oven-dried Schlenk flask equipped with a Teflon-coated magnetic stirring rod. The solution was cooled to -78 °C and *t*BuLi (1.9 M in pentane, 2.4 equiv.) was added dropwise. After complete addition, the reaction was warmed to 0 °C and stirred for 1 h. A round bottom flask was filled with dry ice and the outlet passed through a gas wash bottle filled with conc. sulfuric acid as drying reagent. The stream of $CO_2$ was bubbled through the solution using a needle. After 1 h, the reaction mixture was quenched by slowly adding water. The layers were separated and the organic layer was discarded. The aqueous layer was acidified to pH = 3 with aqueous HCl (2M) and extracted with diethyl ether (2x30 mL). The combined organic layers were dried over $Na_2SO_4$ and the solvent was removed under reduced pressure to give the dicarboxylic acid.

### 2.1.4. General Procedure D: Synthesis of olefines

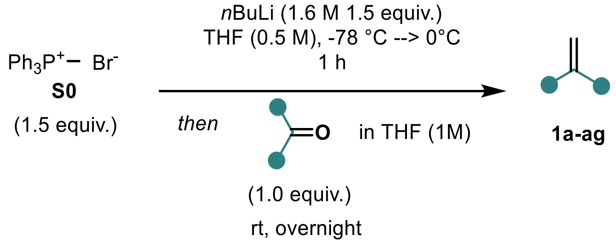


To an oven dried Schlenk flask with a Teflon-coated magnetic stir bar was added **S0** (1.5 equiv.) and Schlenk tube was evacuated and backfilled with argon three times before THF (0.5 M) was added. The reaction was cooled to -78 °C and *n*BuLi (1.6 M in hexane, 1.5 equiv.) was added dropwise. The reaction was warmed to room temperature and stirred for 1 h. The ketone was dissolved in THF (1 M) and was added dropwise. The reaction was stirred overnight and afterwards quenched with sat. aq. $NH_4Cl$ solution (5 mL per 1 mmol). The layers were sperarated and the aqueous layer was extracted with EtOAc (5 mL per 1 mmol). The combined organic layers were washed with sat. aq. NaCl (15 ml per 1 mmol), dried over $MgSO_4$ and the solvent was removed under reduced pressure. The product was purified by flash column chromatography on silica gel to yield the corresponding olefine.

## 2.2. Synthesis of Starting Material

### 2.2.1. Preparation of Malonyl Peroxides

#### 2.2.1.1. Synthesis of Dicarboxylic Acid

**diethyl tetrahydro-4H-pyran-4,4-dicarboxylate (S1-2d)**

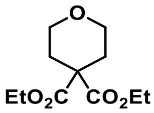


To an oven dried Schlenk flask with a Teflon-coated magnetic stir bar was added Sodium (3.00 equiv., 15.0 mmol, 345 mg) in dry ethanol (10 mL). Dimethyl malonate (5.00 mmol, 629 µL, 1.0 equiv) was added dropwise followed by bis(2-bromoethyl) ether (1.00 equiv., 5.00 mmol, 1.16 g, 629 µL) at 0 °C and the reaction was subsequently refluxed for 4 h (conversion followed by GC-MS). After cooling to room temperature, the mixture was quenched with aqeous HCl (1 M 30 ml) and extracted with ethyl acetate (3x30 ml). The combined organic layers were dried over MgSO4 and the solvent was evaporated. Purification of the crude mixture by column chromatography ($SiO_2$; 98:2 to 95:5 pentane:EtOAc) yielded **S1-2d** as a colorless liquid (437 mg, 1.90 mmol, 38%).

**TLC**: $R_f$ = 0.56 (90:10 pentane:EtOAc)

**NMR Spectroscopy (*see spectra*):**

$^1$**H NMR** (400 MHz, $CDCl_3$): $\delta_H$ 4.17 (q, *J* = 7.1 Hz, 4H), 3.65 – 3.60 (m, 4H), 2.10 – 2.01 (m, 4H), 1.21 (t, *J* = 7.1 Hz, 6H);

The NMR spectra is in accordance with the literature.[4]

**HRMS** ($ESI^+$): *m*/*z* calc'd for **S1-2d** $C_{11} H_{18} O_5$ Na $[M+Na]^+$: 253.10464, found: 253.10419.

**tetrahydro-4H-pyran-4,4-dicarboxylic acid (S2-2d)**

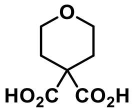


In a round bottom flask diethyl tetrahydro-4H-pyran-4,4-dicarboxylate **S1-2d** (437 mg, 1.90 mmol, 1.0 equiv.) was suspended in aqueous NaOH (*w* = 30%, 20 ml). The reaction was stirred at room temperature overnight. The reaction mixture was washed with ethyl acetate and the organic layer was discarded. The aqueous layer was acidified to pH = 1 with conc. aqueous HCl (while maintaining a temperature below 40 °C) and subsequently extracted with ethyl acetate (3x 30 mL). The combined organic layers were dried over $Na_2SO_4$ and the solvent was evaporated to give the title product as a colorless solid (260 mg, 1.49 mmol, 78%).

**NMR Spectroscopy (*see spectra*):**

$^1$**H NMR** (400 MHz, DMSO-$d_6$): $\delta_H$ 12.88 (s, 2H), 3.59 – 3.52 (m, 4H), 1.91 (m, 4H);

$^{13}$**C NMR** (101 MHz, DMSO-$d_6$): $\delta_C$ 172.2, 64.1, 51.5, 30.9.

**HRMS** ($ESI^+$): *m*/*z* calc'd for **S2-2d** $C_7H_9O_5$ $[M-H]^-$: 173.04555, found: 173.04550.

**diethyl spiro[3.3]heptane-2,2-dicarboxylate (S1-2e)**

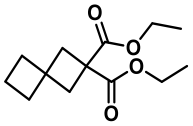

The title compound was prepared according to a modified literature procedure. THF (200 mL) was added to an oven-dried Schlenk flask with a Teflon-coated magnetic stir bar. At 0°C $LiAlH_4$ (4.0 g, 101 mmol, ) was added in portions. In a second oven dried Schlenk flask cyclobutane-1,1-dicarboxylic acid was dissolved in THF. The dicarboxylic acid solution was then added dropwise to the LiAlH4 solution at 0 °C. After complete addition the reaction was stirred at room temperature for 16 h. The reaction was slowly quenched by adding water dropwise. The layerswere separated and the aqueous layer was extracted with EtOAc (4x100 mL). The combined organic layers were dried over $MgSO_4$ and the solvent was evaporated under reduced pressure. The crude product was used without further purification.

The crude product was dissolved in 80 mL of DCM and pyridine (12 mL), 4-(dimethylamino)pyridine (0.91 g, 7.4 mmol, 20mol%) and p-toluenesulfonyl chloride (15.7 g, 82.4 mmol, 2.2 equiv.) were added. The reaction was stirred for 16 h at room temperature. The solution was washed with saturated aqueous NaCl solution (3x80 mL). The organic layer was dried over $MgSO_4$ and the solvent was removed under reduced pressure. The intermediate product was isolated as a white solid **Int 1** (12.9 g, 30.4 mmol, 81% over two steps).

To an oven dried Schlenk flask with a Teflon-coated magnetic stir bar were added *p*-xylene (36 mL) and diethyl malonate (4.9 mL, 32 mmol, 2.5 equiv.). Sodium metal (0.74 g, 32 mmol, 2.5 equiv.) was chopped into small pieces, washed with pentane and added to the solution. The reaction was heated to 100 °C. The colour changed to orange and the reaction was cooled to room temperature. Then **Int 1** (5.5 g, 12.8 mmol, 1.0 equiv.) was added and the reaction was stirred at reflux for 16 h. The reaction was allowed to cool down to room temperature and sat. aqueous $NH_4Cl$ solution (30 mL) was added. The layers were separated and the organic layer was washed with sat. aqueous $NaHCO_3$ solution (30 mL). The organic layer was dried over $MgSO_4$ and the solvent was removed under reduced pressure. evaporated. Purification of the crude mixture by column chromatography ($SiO_2$; 98:2 → 90:10 pentane:EtOAc) afforded **S1-2e** as a colorless liquid (1.45 g, 6.0 mmol, 47%).

**NMR Spectroscopy (*see spectra*):**

$^1$**H NMR** (500 MHz, $CDCl_3$): $\delta_H$ 4.18 (q, *J* = 7.1 Hz, 4H), 2.55 (s, 4H), 2.00 (t, *J* = 7.5 Hz, 4H), 1.85 – 1.70 (m, 2H), 1.24 (t, *J* = 7.1 Hz, 6H);

$^{13}$**C NMR** (101 MHz, $CDCl_3$): $\delta_C$ 172.1, 61.4, 48.8, 41.6, 38.6, 35.3, 16.2, 14.2.

**HRMS** ($ESI^+$): *m*/*z* calc'd for **S1-2e** $C_{13}H_{20}O_4Na$ $[M+Na]^+$: 263.12538, found: 263.12525.

**spiro[3.3]heptane-2,2-dicarboxylic acid (S2-2e)**

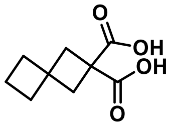

In a round bottom diethyl spiro[3.3]heptane-2,2-dicarboxylate **S1-2e** (1.45 g, 6.0 mmol, 1.0 equiv.) was dissolved in MeOH(50 mL) and to a aqueous KOH solution (2M, 50 mL) was added. The reaction was stirred at room temperature for 16 h. The solution was acidifieid to pH = 1 using aqueous HCl (2M 60 mL) and the layer were separated. The aqueous layer was extracted with EtOAc and the combined organic layer were dried over $Na_2SO_4$. The solvent was removed under reduced pressure and the compound was afforeded as a white solid (880 mg, 4.8 mmol, 80%).

**NMR Spectroscopy (*see spectra*):**

$^{1}$**H NMR** (500 MHz, DMSO-*d6*): $\delta_H$ 12.59 (s, 2H), 2.41 (s, 3H), 1.93 (t, *J* = 7.4 Hz, 4H), 1.77 – 1.68 (m, 2H);

$^{13}$**C NMR** (101 MHz, DMSO-*d6*): $\delta_C$ 172.9, 47.9, 41.1, 37.7, 34.7, 15.6.

**HRMS** (ESI$^+$): *m*/*z* calc'd for **S2-2e** $C_9H_{11}O_4$ [M-H]$^-$: 183.06519, found: 183.06626.

### 2.2.1.2. Synthesis of Malonyl Peroxides

**6,7-dioxaspiro[3.4]octane-5,8-dione (2a)**

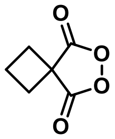

The title compound was prepared according to General Procedure B from cyclobutane-1,1-dicarboxylic acid (2.88 g, 20 mmol, 1.0 equiv.) and hydrogen peroxide–urea (5.64 g, 60 mmol, 3.0 equiv.). The product was obtained as a white solid (14.4 mmol 2.05 g, 72%)

**NMR Spectroscopy (*see spectra*):**

$^{1}$**H NMR** (599 MHz, $CDCl_3$): $\delta_H$ 2.72 – 2.63 (m, 4H), 2.37 – 2.28 (m, 2H);

$^{13}$**C NMR** (151 MHz, $CDCl_3$): $\delta_C$ 174.1, 40.6, 29.0, 16.3.

**HRMS** ($ESI^+$): *m/z* calc'd for **2a** $C_6H_8O_4H$ $[M+Na]^+$: 145.04954, found: 145.04936.

**DSC:** No violent decomposition was observed upon heating in DSC.

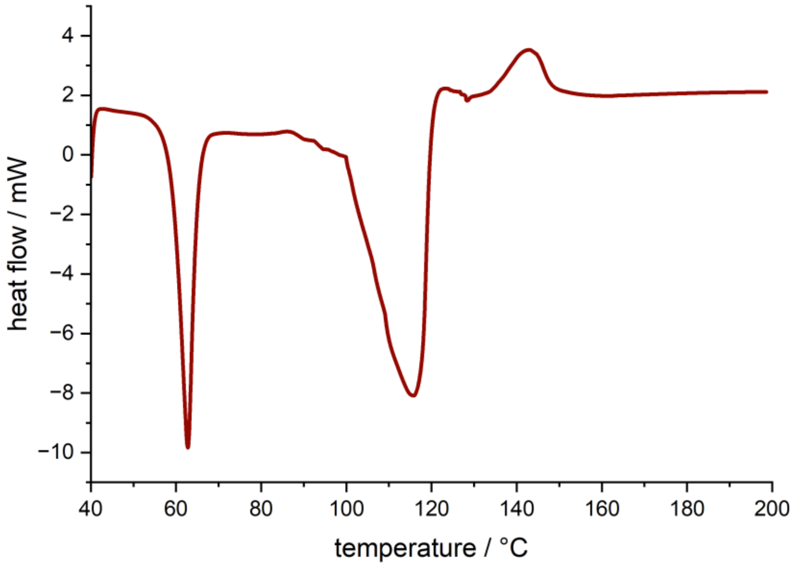


**2,3-dioxaspiro[4.4]nonane-1,4-dione (2b)**

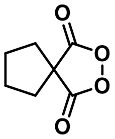

The title compound was prepared according to General Procedure B from cyclopentane-1,1-dicarboxylic acid (3.16 g, 20 mmol, 1.0 equiv.) and hydrogen peroxide–urea (5.64 g, 60 mmol, 3.0 equiv.). The product **2b** was obtained as a white solid (2.25 g, 14.4 mmol, 72%).

**NMR Spectroscopy (*see spectra*):**

**$^{1}$H NMR** (599 MHz, $CDCl_3$): $\delta_H$ 2.31 – 2.22 (m, 4H), 2.06 – 1.97 (m, 4H);

**$^{13}$C NMR** (151 MHz, $CDCl_3$): $\delta_C$ 175.8, 47.0, 37.8, 26.8.

**HRMS** (ESI$^+$): *m*/*z* calc'd for **2b** $C_7H_8O_4H$ [M+H]$^-$: 157.05063, found: 157.05048.

**DSC:** No violent decomposition was observed upon heating in DSC.

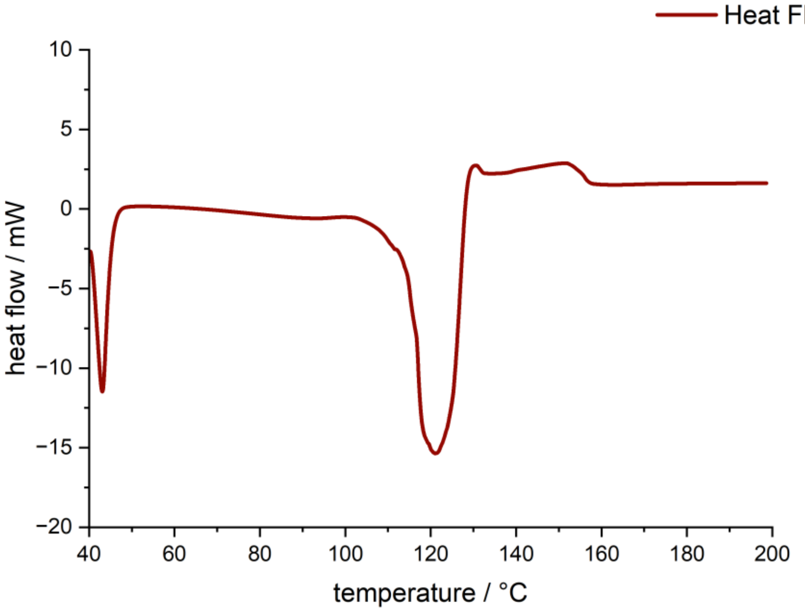


**2,3-dioxaspiro[4.5]decane-1,4-dione (2c)**

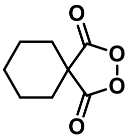


The title compound was prepared according to General Procedure B from cyclohexane-1,1-dicarboxylic acid (860 mg, 5.0 mmol, 1.0 equiv.) and hydrogen peroxide–urea (1,18 g,12.5 mol, 2.5 equiv.). The product **2c** was obtained as a white solid (759 mg, 4.5 mmol, 89%).

**NMR Spectroscopy (*see spectra*):**

**$^{1}$H NMR** (599 MHz, $CDCl_3$): $\delta_H$ 1.99 – 1.94 (m, 4H), 1.85 – 1.78 (m, 4H), 1.64 – 1.57 (m, 2H);

**$^{13}$C NMR** (151 MHz, $CDCl_3$): $\delta_C$ 174.1, 41.8, 30.7, 24.2, 19.4.

**HRMS** (ESI$^+$): *m*/*z* calc'd for **2c** $C_8H_{10}O_4H$ [M+H]$^-$: 171.06628, found: 171.06606.

**2,3,8-trioxaspiro[4.5]decane-1,4-dione (2d)**

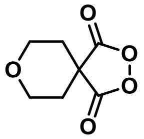

The title compound was prepared according to General Procedure B from tetrahydro-4H-pyran-4,4-dicarboxylic acid (226 mg, 1.3 mmol, 1.0 equiv.) and hydrogen peroxide–urea (306 mg, 3.25 mmol, 2.5 equiv.). The product **2d** was obtained as a white solid (80 mg, 0.47 mmol, 36%).

**NMR Spectroscopy (*see spectra*):**

$^{1}$**H NMR** (599 MHz, $CDCl_3$): $\delta_H$ 4.00 – 3.94 (m, 4H), 2.11 – 2.05 (m, 4H);

$^{13}$**C NMR** (151 MHz, $CDCl_3$): $\delta_C$ 173.1, 61.1, 39.6, 29.6.

**HRMS** (ESI$^+$): *m*/*z* calc'd for **2d** $C_7H_8O_5$ $[M+Na]^+$: 195.02639, found: 195.02631.

**DSC:** No violent decomposition was observed upon heating in DSC.

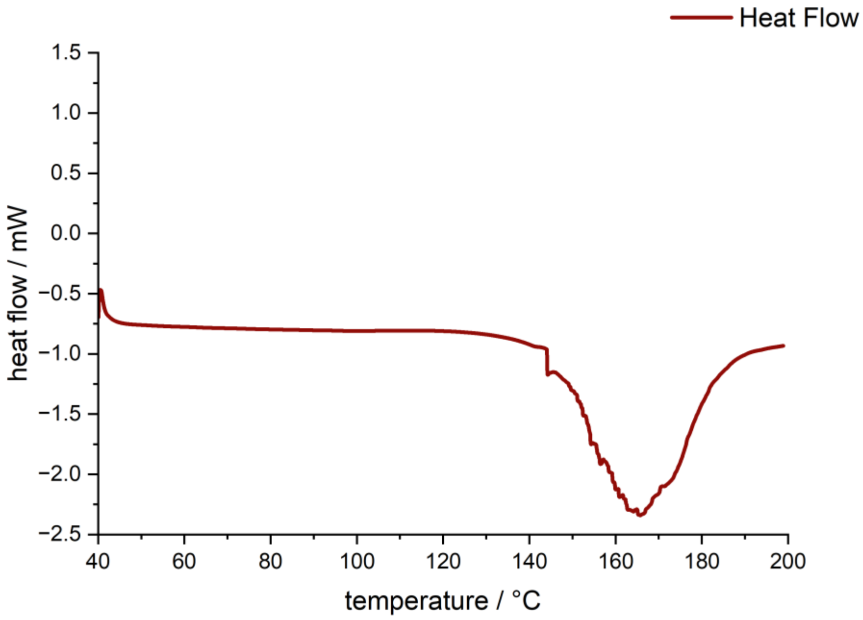


**Crystal structure:** *see crystal structure*

**8,9-dioxadispiro[3.1.46.14]undecane-7,10-dione (2e)**

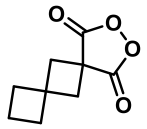


The title compound was prepared according to General Procedure B from spiro[3.3]heptane-2,2-dicarboxylic acid (**S2-2e**) (880 mg, 4.8 mmol, 1.0 equiv.) and hydrogen peroxide–urea (1.35 g, 14.4 mmol, 3.0 equiv.). The product **2e** was obtained as a white solid (730 mg, 4.0 mmol, 83%).

**NMR Spectroscopy (*see spectra*):**

$^{1}$**H NMR** (599 MHz, $CDCl_3$): $\delta_H$ 2.68 (s, 4H), 2.20 (t, *J* = 7.7 Hz, 4H), 1.85 (quin, *J* = 7.7 Hz, 2H).

$^{13}$**C NMR** (151 MHz, $CDCl_3$): $\delta_C$ 174.3, 41.3, 38.9, 37.3, 35.2, 15.6.

**HRMS** (ESI$^+$): *m*/*z* calc'd for **2e** $C_9H_{11}O_4$ [M+H]$^-$: 183.06628, found: 183.06625.

**DSC:** No violent decomposition was observed upon heating in DSC.

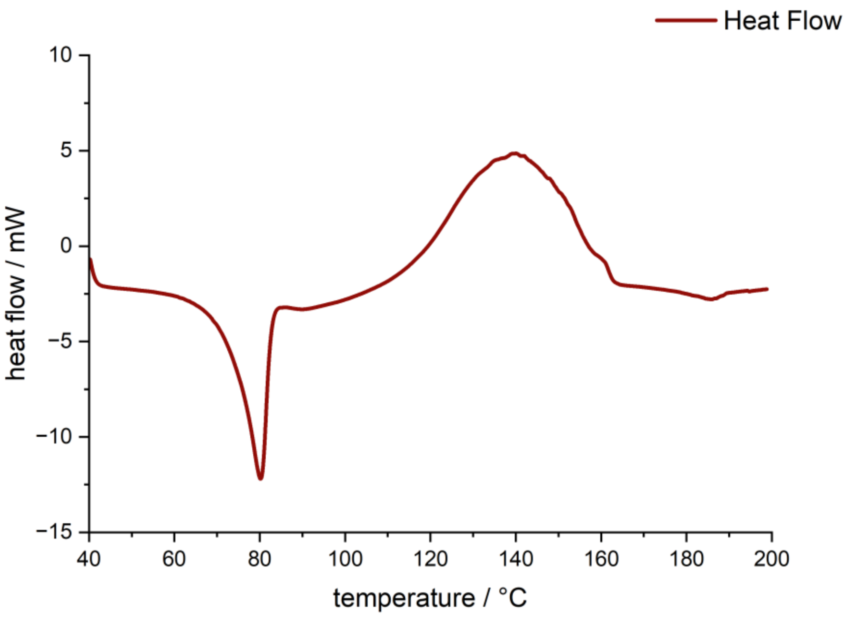


**5,6-dioxaspiro[2.4]heptane-4,7-dione (2f)**

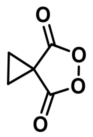


The title compound was prepared according to General Procedure B from cyclopropane-1,1-dicarboxylic acid (3.90 g, 30 mmol, 1.0 equiv.) and hydrogen peroxide–urea (8.47 g, 90 mmol, 3.0 equiv.). The product **2f** was obtained as a white solid (2.93 g, 22.8 mmol, 76%).

**NMR Spectroscopy (*see spectra*):**

$^{1}$**H NMR** (599 MHz, $CDCl_3$): $\delta_H$ 2.08 (s, 4H);

$^{13}$**C NMR** (151 MHz, $CDCl_3$): $\delta_C$ 172.3, 23.8, 19.9

**HRMS** (ESI$^+$): *m*/*z* calc'd for **2f** $C_5H_6O_4H$ $[M+H]^+$: 131.03389, found 131.03366:

**DSC:** No violent decomposition was observed upon heating in DSC.

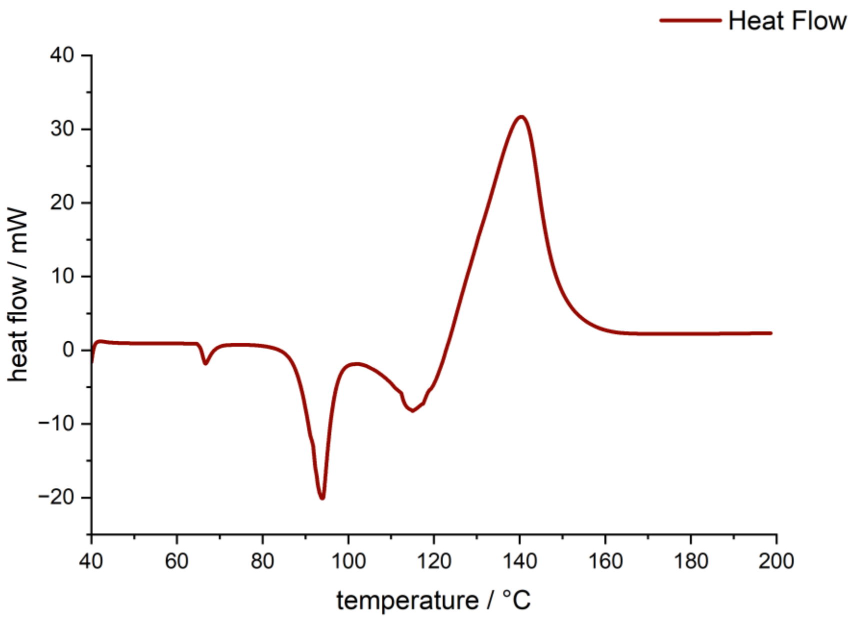


**4,4-dipropyl-1,2-dioxolane-3,5-dione (2g)**

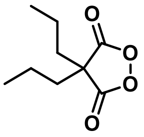

The title compound was prepared according to General Procedure B from 2,2-dipropyl malonic acid (1.88 g, 10 mmol, 1.0 equiv.) and hydrogen peroxide–urea (2.35 g, 25 mmol, 2.5 equiv.). The product was obtained as a colorless liquid (1.628 g, 88%).

**NMR Spectroscopy (*see spectra*):**

$^{1}$**H NMR** (599 MHz, $CDCl_3$): $\delta_H$ 1.89 – 1.82 (m, 4H), 1.38 – 1.29 (m, 4H), 0.92 (t, *J* = 7.3 Hz, 6H);

$^{13}$**C NMR** (151 MHz, $CDCl_3$): $\delta_C$ 174.4, 49.7, 37.8, 18.1, 13.7.

**4-methyl-4-propyl-1,2-dioxolane-3,5-dione (2h)**

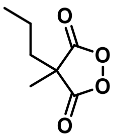

The dicarboxylic acid was prepared according to General Procedure C using 2-methylpentanoic acid (1.6 mL, 13.2 mmol, 1.00 equiv.) resulting in a yellow solid crude product (1.38 g). The crude product was used without further purification and the title compound was prepared according to General Procedure B using hydrogen

peroxide–urea (2.43 g, 25.9 mmol, 3.0 equiv.). The product **2h** was obtained as a clear liquid (1.12 g, 7.1 mmol, 54% over two steps).

**NMR Spectroscopy (*see spectra*):**

$^1$**H NMR** (400 MHz, $CDCl_3$): $\delta_H$ 1.91 – 1.85 (m, 2H), 1.54 (s, 3H), 1.42 – 1.31 (m, 2H), 0.95 (t, $J$ = 7.3 Hz, 3H);

$^{13}$**C NMR** (101 MHz, $CDCl_3$): $\delta_C$ 174.5, 44.1, 38.7, 20.6, 18.2, 13.7.

**HRMS** (ESI$^+$): *m/z* calc'd for **2h** $C_7H_{10}O_4CH_3OHNa$ [M+MeOH+Na]$^+$: 213.07334, found: 213.07307.

**1,3-dihydrospiro[indene-2,4'-[1,2]dioxolane]-3',5'-dione (2j)**

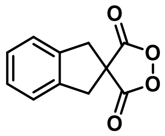


The dicarboxylic acid was prepared according to General Procedure C using 2,3-dihydro-1H-indene-2-carboxylic acid (1.36 g, 8.38 mmol, 1.00 equiv.) resulting in a white solid crude product (836 mg). The crude product was used without further purification and the title compound was prepared according to General Procedure B using hydrogen peroxide–urea (1.14 g, 12.2 mmol, 3.0 equiv.). The product **2j** was obtained as a white solid (502 mg, 1.39 mmol, 17% over two steps).

**NMR Spectroscopy (*see spectra*):**

$^1$**H NMR** (400 MHz, $CDCl_3$): $\delta_H$ 7.31 – 7.28 (m, 2H), 7.28 – 7.25 (m, 2H), 3.66 (s, 4H).;

$^{13}$**C NMR** (101 MHz, $CDCl_3$): $\delta_C$ 174.6, 137.4, 128.4, 124.6, 46.5, 42.7.

**HRMS** (ESI$^+$): *m/z* calc'd for **2j** $C_{11}H_9O_4$ [M-H]$^-$: 205.05063, found: 205.05052.

**DSC:** No violent, but slightly exothermic decomposition was observed upon heating in DSC.

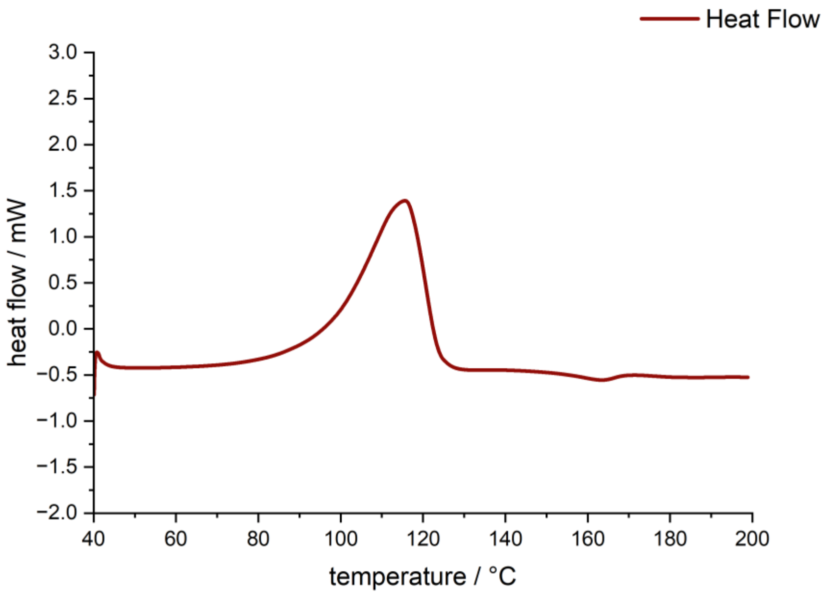

### 2.2.2. Synthesis of Olefines

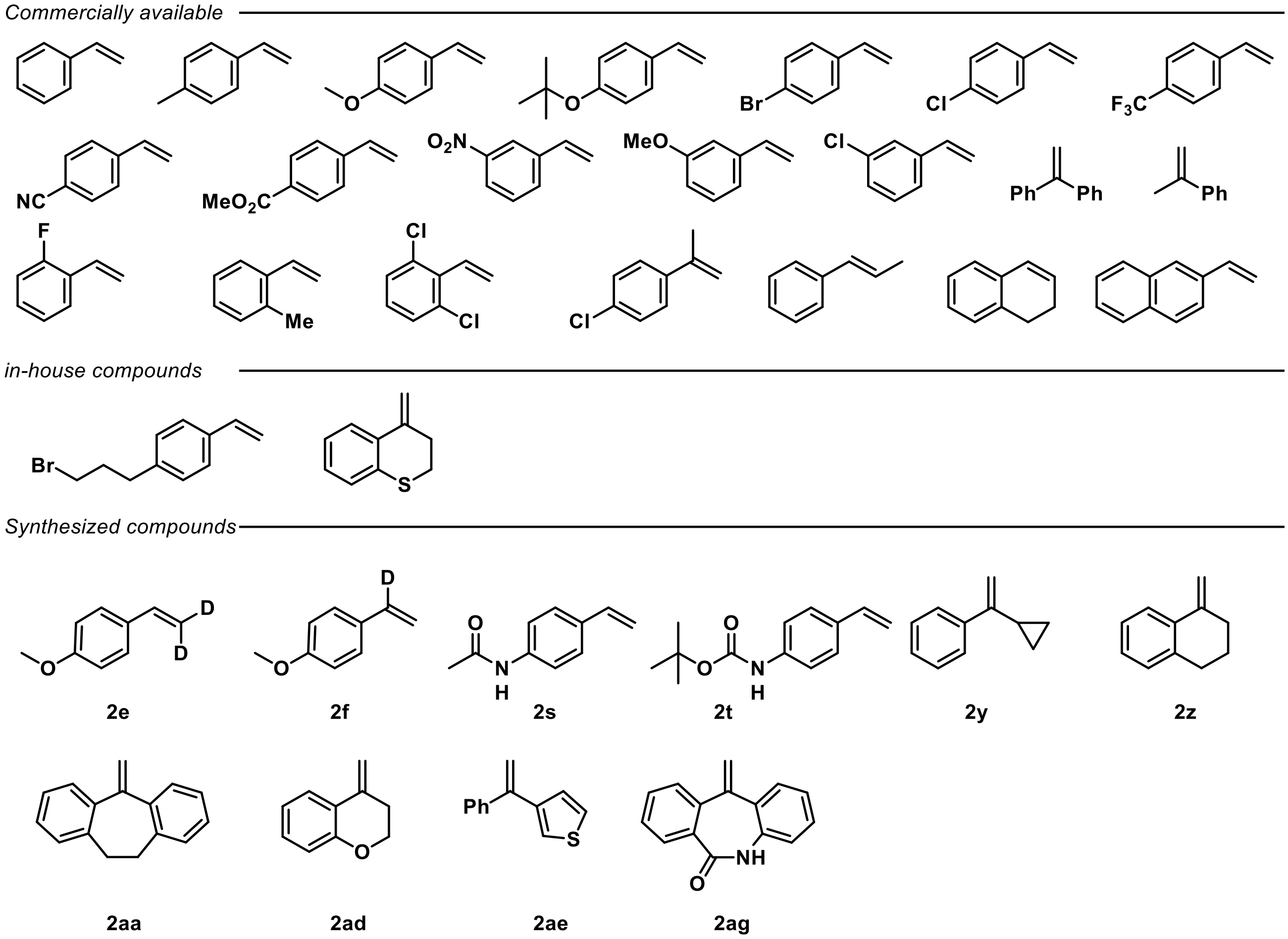


**Figure S4:** Overview of the alkenes employed in this work.

**(methyl-$d_3$)triphenylphosphonium iodide (S1-1e)**

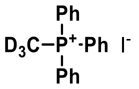

The title compound was synthesized following a literature procdedure.[5] To an oven dried round bottom flask equipped with a Teflon-coated magnetic stir bar was added triphenylphosphine (5.34 g, 16.5 mmol, 1.20 equiv.) and toluene (30.0 mL). Methyl-$d_3$ iodide (2.00 g, 13.8 mmol 1.00 equiv.) was added dropwise and the reaction was stirred at room temperature for 16 h. The reaction was filtered and the residue was washed with toluene (3x10 mL) and pentane (3x10 mL). The product **S1-1e** was isolated as a white solid.

**NMR Spectroscopy (*see spectra*):**

$^1$**H NMR** (400 MHz, CDCl$_3$): $\delta_H$ 7.74 – 7.66 (m, 3H), 7.65 – 7.53 (m, 12H);

$^{13}$**C NMR** (126 MHz, CDCl$_3$): $\delta_C$ 135.0 (d, $J$ = 3.0 Hz), 132.9, 130.3 (d, $J$ = 12.9 Hz), 118.4 (d, $J$ = 88.7 Hz), 11.5 – 9.5 (m);

$^{31}$**P NMR** (162 MHz, CDCl$_3$): $\delta_P$ 21.1.

**HRMS** (ESI$^+$): $m/z$ calc'd for $C_{19}$ $H_{15}$ $PD_3$ [M-I]$^+$: 280.13289, found: 280.13292.

**1-methoxy-4-(vinyl-2,2-d2)benzene (1e)**

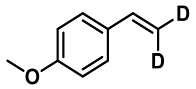

The compound was synthesized following a modified version of the General Procedure D An oven dried Schlenk flaks was charged with KOtBu (273 mg, 2.43 mmol, 1.19 equiv.) and (methyl-$d_3$)triphenylphosphonium iodide 1.00 g, 2.46 mmol, 1.21 equiv.). The Schlenk Tube was evacuated and backfilled with argon three times before THF (0.5 M) was added at 0 °C and stirred for 1 h at rt. The aldehyde 1-methoxy-benzaldehyde (250 µL, 2.04 mmol, 1.00 equiv) was dissolved in THF (1 mL) and was added dropwise at 0 °C. The reaction was stirred for 16 h and the work up is copied from General Procedure D. Purification by silica gel column chromatography (99:1 → 98:2 pentane:EtOAc) afforded the product **1e** as a colourless oil (150 mg, 1.10 mmol, 55% yield, 86% deuterium incorporation).

**TLC**: $R_f$ = 0.51 (98:2 pentane:EtOAc)

**NMR Spectroscopy (*see spectra*):**

$^1$**H NMR** (500 MHz, $CDCl_3$): $\delta_H$ 7.38 – 7.32 (m, 2H), 6.90 – 6.83 (m, 2H), 6.65 (s, 1H), 3.82 (s, 3H);

$^{13}$**C NMR** (126 MHz, $CDCl_3$): $\delta_C$ 159.5, 136.2, 130.6, 127.5, 114.1, 111.8 – 111.1 (m), 55.4.

**GC-MS** ($EI^+$): *m*/*z* calc'd for **1e** $C_9H_8D_2O$ $[M]^+$: 136.08572, found: 136.1 121.0 105.0

**formyl-d, 4-methoxy-benzaldehyde (S1-1f)**

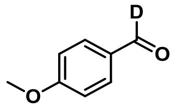

The title compound **S1-1f** previously synthesized in-house.[6]

**TLC**: $R_f$ = 0.22 (98:2 pentane:EtOAc)

**NMR Spectroscopy (*see spectra*):**

$^1$**H NMR** (400 MHz, $CDCl_3$): $\delta_H$ 7.88 – 7.79 (m, 2H), 7.04 – 6.96 (m, 2H), 3.88 (d, *J* = 1.0 Hz, 3H);

$^{13}$**C NMR** (101 MHz, $CDCl_3$): $\delta_C$ 190.6 (t, *J* = 25.9 Hz), 164.7, 132.1, 130.0 (t, *J* = 3.5 Hz), 114.4, 55.7.

**HRMS** ($ESI^+$): *m*/*z* calc'd for $C_8H_7O_2DNa$ $[M+Na]^+$: 160.04793, found: 160.04788.

**1-methoxy-4-(vinyl-1-*d*)benzene (1f)**

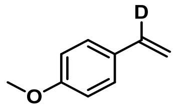

The compound was synthesized following a modified version of the General Procedure D using formyl-*d*, 4-methoxy-benzaldehyde **S1-1f** (588 mg, 4.29 mmol, 1.35 equiv), triphenylphosphonium bromide (1.30 g, 3.60 mmol, 1.11 equiv.) and KOtBu (364 mg, 25 mmol, 1.00 equiv.). An oven dried Schlenk flaks was charged with KOtBu and triphenylphosphonium bromide. The Schlenk Tube was evacuated and backfilled with argon

three times before THF (0.5 M) was added at 0 °C and stirred for 1 h at rt. The aldehyde was dissolved in THF (1 mL) and was added dropwise at 0 °C. The reaction was stirred for 16 h and the work up is copied from General Procedure D. Purification by silica gel column chromatography (100:0 → 98:2 pentane:EtOAc) afforded the product **1f** as a colourless oil (150 mg, 1.10 mmol, 55% yield, 86:14 deuterated and no deuterated).

**TLC**: $R_f$ = 0.5 (98:2 pentane:EtOAc)

**NMR Spectroscopy (*see spectra*):**

$^1$**H NMR** (400 MHz, $CDCl_3$): $\delta_H$ 7.38 – 7.32 (m, 2H), 6.90 – 6.83 (m, 2H), 5.63 – 5.58 (m, 1H), 5.14 – 5.10 (m, 1H), 3.81 (s, 3H);

$^{13}$**C NMR** (101 MHz, $CDCl_3$): $\delta_C$ 159.4, 135.9 (d, *J* = 23.5 Hz), 130.4, 127.4, 113.9, 111.4, 55.3.

**GC-MS** ($EI^+$): *m/z* calc'd for **1f** $C_9H_9DO$ $[M]^+$: 135.07944, found: 135.1 120.0 104.0

**N-(4-vinylphenyl)acetamide (1s)**

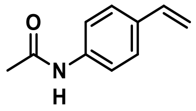

The title compound was synthesized following a literature procdedure.[7] To an oven dried round bottom flask equipped with a Teflon-coated magnetic stir bar was added 4-vinylaniline (235 µL, 2.00 mmol, 1.00 equiv.) and DCM (5.0 mL). Acetic anhydride (200 µL, 2.20 mmol 1.10 equiv.) was added dropwise and the reaction was stirred at room temperature for16 h. The reaction was quenched by the addition of sat. aq. $NaHCO_3$ solution (10 mL). The Layers were separated, and the aqueous layer was extracted with $CH_2Cl_2$ (3x 10 mL), dried over $MgSO_4$ and the solvent was removed under reduced pressure. The compound was purified by flash column chromatography ($SiO_2$; 33:67 pentane:EtOAc) to afford **1s** (0.280 g, 1.74 mmol, 87%) as a white solid.

**TLC**: $R_f$ = 0.30 (33:67 pentane/EtOAc)

**NMR Spectroscopy (*see spectra*):**

$^1$**H NMR** (400 MHz, $CDCl_3$): $\delta_H$ 7.47 (d, *J* = 8.3 Hz, 2H), 7.36 (d, *J* = 8.3 Hz, 2H), 7.20 (br s, 1H), 6.67 (dd, *J* = 17.6, 10.9 Hz, 1H), 5.68 (d, *J* = 17.6 Hz, 1H), 5.19 (d, *J* = 10.9 Hz, 1H), 2.18 (s, 3H);

$^{13}$**C NMR** (101 MHz, $CDCl_3$): $\delta_C$ 168.1, 137.4, 136.1, 133.8, 126.9, 119.7, 113.1, 24.7.

**HRMS** ($ESI^+$): *m/z* calc'd for $C_{10}H_{11}NONa$: $[M+Na]^+$: 184.07329, found: 184.07320.

**tert-butyl (4-vinylphenyl)carbamate (1t)**

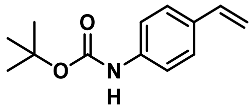

The title compound **1t** previously synthesized in-house.[8]

**TLC**: $R_f$ = 0.5 (90:10 pentane:EtOAc)

**NMR Spectroscopy (*see spectra*):**

**$^1$H NMR** (400 MHz, $CDCl_3$): $\delta_H$ 7.33 (d, $J$ = 2.6 Hz, 4H), 6.65 (ddd, $J$ = 17.5, 10.8, 1.4 Hz, 1H), 6.47 (s, 1H), 5.65 (dd, $J$ = 17.6, 1.1 Hz, 1H), 5.16 (dd, $J$ = 10.9, 1.1 Hz, 1H), 1.52 (s, 9H);

**$^{13}$C NMR** (101 MHz, $CDCl_3$): $\delta_C$ 152.7, 138.1, 136.3, 132.7, 127.0, 118.5, 112.5, 28.5.

**HRMS** (ESI$^+$): $m/z$ calc'd for $C_{13}H_{17}NO_2Na$ [M+Na]$^+$: 242.11515, found: 242.11524.

**(1-cyclopropylvinyl)benzene (1y)**

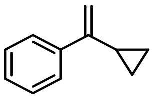

The title compound previously synthesized in-house.[9]

**TLC**: $R_f$ = 0.8 (100:0 pentane)

**NMR Spectroscopy (*see spectra*):**

**$^1$H NMR** (500 MHz, $CDCl_3$): $\delta_H$ .66 – 7.60 (m, 2H), 7.38 (m, 2H), 7.34 – 7.28 (m, 1H), 5.32 (d, $J$ = 1.0 Hz, 1H), 4.98 (d, $J$ = 1.0 Hz, 1H), 1.74 – 1.65 (m, 1H), 0.92 – 0.82 (m, 2H), 0.69 – 0.60 (m, 2H);

**$^{13}$C NMR** (126 MHz, $CDCl_3$): $\delta_C$ 149.52, 141.79, 128.28, 127.57, 126.26, 109.14, 15.78, 6.82..

**GC-MS** (EI$^+$): $m/z$ calc'd for **1y** $C_{11}H_{12}$ [M]$^+$: 144.093390, found: 144.1, 129.0, 103.2.

**1-methylene-1,2,3,4-tetrahydronaphthalene (1z)**

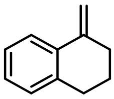

The compound was synthesized following General Procedure D using 3,4-dihydronaphthalen-1(2H)-one (532 µL, 4.00 mmol, 1.00 equiv), methyltriphenylphosphonium bromide (2.14 g, 6.00 mmol, 1.50 equiv.) and *n*BuLi (3.8 mL, 6.00 mmol, 1.50 equiv.). Purification by silica gel column chromatography (100 % pentane) afforded the product **1z** as a colourless oil (300 mg, 2.08 mmol, 52% yield).

**TLC**: $R_f$ = 0.7 (50:50 pentane:EtOAc)

**NMR Spectroscopy (*see spectra*):**

**$^1$H NMR** (400 MHz, $CDCl_3$): $\delta_H$ 7.70 – 7.61 (m, 1H), 7.21 – 7.13 (m, 2H), 7.12 – 7.09 (m, 1H), 5.48 (d, $J$ = 1.3 Hz, 1H), 4.96 (d, $J$ = 1.4 Hz, 1H), 2.85 (t, $J$ = 6.3 Hz, 2H), 2.59 – 2.51 (m, 2H), 1.94 – 1.84 (m, 2H);

**$^{13}$C NMR** (101 MHz, $CDCl_3$): $\delta_C$ 143.5, 137.3, 134.7, 129.2, 127.6, 125.9, 124.2, 107.9, 33.3, 30.5, 23.8.

**GC-MS** (EI$^+$): $m/z$ calc'd for **1z** $C_{11}H_{12}$ [M]$^+$: 144,09390, found: 144.1 129.0.

**5-methylene-10,11-dihydro-5H-dibenzo[a,d][7]annulene (1aa)**

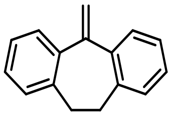

The compound was synthesized following General Procedure D using 10,11-Dihydro-5H-dibenzo[a,d][7]annulen-5-one (417 mg, 2.00 mmol, 1.00 equiv), methyltriphenylphosphonium bromide (1.07 g, 3.00 mmol, 1.50 equiv.) and *n*BuLi (1.9 mL, 3.00 mmol, 1.50 equiv.). Purification by silica gel column chromatography (100% pentane) afforded the product **1aa** as a white solid (295 mg, 1.43 mmol, 71% yield).

**TLC**: $R_f$ = 0.5 (100% pentane)

**NMR Spectroscopy (*see spectra*):**

$^1$**H NMR** (400 MHz, $CDCl_3$): $\delta_H$ 7.38 – 7.34 (m, 2H), 7.23 – 7.15 (m, 4H), 7.14 – 7.11 (m, 2H), 5.42 (d, $J$ = 1.0 Hz, 2H), 3.15 (s, 4H);

$^{13}$**C NMR** (101 MHz, $CDCl_3$): $\delta_C$ 151.8, 141.2, 138.4, 128.9, 128.2, 127.7, 126.2, 117.5, 33.3.

**GC-MS** ($EI^+$): $m/z$ calc'd for **1aa** $C_{16}H_{14}$ $[M]^+$: 206.10955, found: 206.1 191.1 178.1.

**4-methylenechromane (1ad)**

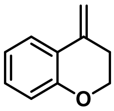

The compound was synthesized following General Procedure D using chroman-4-one (530 µL, 4.00 mmol, 1.00 equiv), methyltriphenylphosphonium bromide (2.14 g, 6.00 mmol, 1.50 equiv.) and *n*BuLi (3.8 mL, 6.00 mmol, 1.50 equiv.). Purification by silica gel column chromatography ($SiO_2$, 99:1 → 98:2 pentane:$Et_2O$) afforded the product **1ad** as a colourless oil (249 mg, 1.70 mmol, 43% yield).

**TLC**: $R_f$ = 0.2 (100% pentane)

**NMR Spectroscopy (*see spectra*):**

$^1$**H NMR** (400 MHz, $CDCl_3$): $\delta_H$ 7.57 (dd, $J$ = 7.9, 1.6 Hz, 1H), 7.17 (ddd, $J$ = 8.5, 7.1, 1.6 Hz, 1H), 6.90 (ddd, $J$ = 8.2, 7.2, 1.3 Hz, 1H), 6.86 – 6.82 (m, 1H), 5.55 – 5.49 (m, 1H), 4.92 – 4.87 (m, 1H), 4.25 (d, $J$ = 5.6 Hz, 2H), 2.69 (t, $J$ = 5.7 Hz, 2H);

The NMR spectra is in accordance with the literature.[10]

**3-(1-phenylvinyl)thiophene (1ae)**

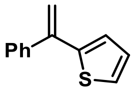


The compound was synthesized following Gener Procedure D using phenyl(thiophen-2-yl)methanone (377 mg, 2.00 mmol, 1 equiv), methyltriphenylphosphonium bromide (1.07 g, 3.00 mmol, 1.50 equiv.) and *n*BuLi (1.9 mL, 3.00 mmol, 1.50 equiv.). Purification by silica gel column chromatography ($SiO_2$, 100:0 pentane) afforded the product **1ae** as a colourless oil (220 mg, 1.18 mmol, 59% yield).

**TLC**: $R_f$ = 0.6; (100 pentane)

**NMR Spectroscopy (*see spectra*):**

**$^1$H NMR** (400 MHz, $CDCl_3$): $\delta_H$ 7.48 – 7.41 (m, 2H), 7.39 – 7.34 (m, 3H), 7.28 – 7.21 (m, 2H), 6.98 (ddd, *J* = 5.1, 3.6, 0.9 Hz, 1H), 6.91 (dd, *J* = 3.6, 1.2 Hz, 1H), 5.59 (d, *J* = 1.0 Hz, 1H), 5.25 (d, *J* = 1.0 Hz, 1H).;

**$^{13}$C NMR** (101 MHz, $CDCl_3$): $\delta_C$ 144.8, 143.4, 141.1, 128.3, 128.2, 128.1, 127.3, 126.5, 125.0, 113.7.

**GC-MS** ($EI^+$): *m*/*z* calc'd for **1ae** $C_{12}H_{10}S$ $[M]^+$: 186,05032, found:186.0 171.0.

**11-methylene-5,11-dihydro-6H-dibenzo[b,e]azepin-6-one (1ag)**

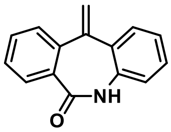


The compound was synthesized following General Procedure D with modified stoichometrics using 5H-dibenzo[b,e]azepine-6,11-dione (446 mg, 2.0 mmol, 1.0 equiv.), methyltriphenylphosphonium bromide (1.57 g, 4.40 mmol, 2.20 equiv.)) and *n*BuLi (2.75 mL, 6.00 mmol, 2.20 equiv.). Purification by silica gel column chromatography (70:30 → 50:50 pentane:EtOAc) afforded the product **1ag** as a grey solid (154 mg, 0.700 mmol, 35%).

**TLC**: $R_f$ = 0.7 (50:50 pentane:EtOAc)

**NMR Spectroscopy (*see spectra*):**

**$^1$H NMR** (400 MHz, $CDCl_3$): $\delta_H$ 8.42 (s, 1H), 8.05 (dd, *J* = 7.9, 1.4 Hz, 1H), 7.54 (td, *J* = 7.5, 1.5 Hz, 1H), 7.42 (td, *J* = 7.6, 1.4 Hz, 1H), 7.35 (ddd, *J* = 7.6, 2.7, 1.4 Hz, 2H), 7.30 – 7.26 (m, 1H), 7.16 (td, *J* = 7.5, 1.2 Hz, 1H), 7.05 – 6.97 (m, 1H), 5.47 (dd, *J* = 15.0, 1.2 Hz, 2H);

**$^{13}$C NMR** (101 MHz, $CDCl_3$): $\delta_C$ 168.8, 149.5, 147.7, 142.6, 134.4, 134.3, 133.0, 130.9, 129.8, 128.9, 128.5, 128.2, 127.5, 125.3, 120.1, 120.0, 119.5.

**HRMS** ($ESI^+$): *m*/*z* calc'd for **1ag** $C_{15}$ $H_{11}$ NONa $[M+Na]^+$: 244.07329, found: 244.07317.

## 2.3. Synthesis of Photocatalyst

### 10-phenyl-10H-phenothiazine (PTH)

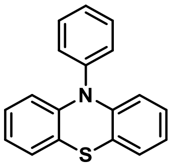

The title compound was synthesized according to a literature procedure[11] using 10*H*-phenothiazine (996 mg, 5.00 mmol, 1.00 equiv.), chlorobenzene (716 µL, 7.00 mmol, 1.40 equiv.), sodium *tert*-butoxide (673 mg, 7.00 mmol, 1.40 equiv.), RuPhos ligand (23 mg, 50 µmol, 1 mol%,) and RuPhos Pd G4 (43 mg, 50 µmol, 1 mol%,) in 1,4-dioxane (40 mL) at 110 °C for 15 h. The reaction mixture was diluted with $CH_2Cl_2$, washed with water (30 mL) and brine (30 mL), dried over $MgSO_4$ and then run through a short silica column with 5% EtOAc/pentane. Evaporation of the solvent yielded the title product as a slightly yellowish crystalline solid (1.38 g, 5.0 mmol, quantative).

**NMR Spectroscopy (*see spectra*):**

$^1$**H NMR** (400 MHz, $CDCl_3$): $\delta_H$ 7.64 – 7.57 (m, 2H), 7.52 – 7.45 (m, 1H), 7.44 – 7.37 (m, 2H), 7.02 (dd, *J* = 7.3, 1.8 Hz, 2H), 6.88 – 6.78 (m, 4H), 6.21 (dd, *J* = 8.1, 1.5 Hz, 2H);

$^{13}$**C NMR** (126 MHz, $CDCl_3$): $\delta_C$ 144.4, 141.2, 131.0, 130.9, 128.3, 127.0, 126.9, 122.6, 120.3, 116.2.

**HRMS** (ESI): *m*/*z* calculated for **PTH** $C_{18}H_{13}NS^+$ $[M]^+$: 275.07632, found: 275.07631.

### 10-phenyl-10H-phenothiazine 5-oxide (PTH-O)

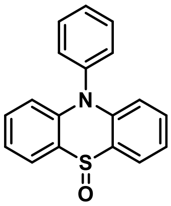

The title compound was synthesized according to an adopted literature procedure.[12] 10-phenyl-10*H*-phenothiazine (138 mg, 0.50 mmol, 1.0 equiv.) was dissolved in acetic acid (0.75 mL) and $H_2O$ (45 µL, 2.5 mmol, 5.0 equiv.). Sodium nitrite (69 mg, 1.0 mmol, 2.0 equiv.) was added slowly and the reaction was stirred at room temperature overnight. $H_2O$ (2 mL) and $CH_2Cl_2$ (3 mL) were added, and the reaction was extracted with $CH_2Cl_2$ (10 mL) two more times. The combined organic layer was washed with sat. aqueous $NaHCO_3$ solution (20 mL) and dried over $Na_2SO_4$. Evaporation of the solvent and purification by column chromatography (MeOH/$CH_2Cl_2$ 1%→5%) and recrystallization from heptane/chloroform gave the title compound as a yellow solid (106 mg, 036 mmol, 72%).

**NMR Spectroscopy (*see spectra*):**

$^1$**H NMR** (600 MHz, $CD_3CN$): $\delta_H$ 7.98 (dd, *J* = 7.7, 1.6 Hz, 2H), 7.76 – 7.72 (m, 2H), 7.70 – 7.66 (m, 1H), 7.46 (ddd, *J* = 8.7, 7.1, 1.6 Hz, 2H), 7.41 – 7.39 (m, 1H), 7.26 (ddd, *J* = 7.7, 7.1, 1.0 Hz, 2H), 6.72 (dd, *J* = 8.7, 1.0 Hz, 2H);

**$^{13}$C NMR** (126 MHz, $CD_3CN$): $\delta_C$ 140.3, 139.8, 133.7, 132.5, 132.4, 131.3, 130.6, 124.0, 123.1.

**HRMS** (ESI): *m/z* calculated for **PTH-O** $C_{18}H_{13}NOSNa^+$ [M+Na]$^+$: 314.0610, found: 314.0606.

**Crystal structure:** *see crystal structure*

**10-phenyl-10H-phenothiazine 5,5-dioxide (PTH-O$_2$)**

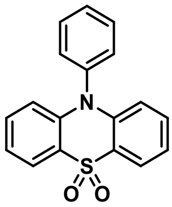


Following an adapted literature procedure[13] 10-phenyl-10*H*-phenothiazine (**PTH,** 275 mg, 1.0 mmol, 1.0 equiv.) was dissolved in $CH_2Cl_2$ (5 mL) in an oven dried pressure tube. $H_2O_2$ (0.75 mL, 30% in $H_2O$) was added and the reaction was stirred at 45 °C overnight. The reaction mixture was extracted with DCM (3x 5 mL), concentrated and purified by column chromatography ($SiO_2$, 90:10 → 80:20 pentane:EtOAc) to obtain the title product as a white solid (190 mg, 0.62 mmol, 62%).

**TLC**: $R_f$ = 0.28 (80:20 pentane:EtOAc)

**NMR Spectroscopy (*see spectra*):**

**$^1$H NMR** (600 MHz, $CDCl_3$): $\delta_H$ 8.18 (dd, *J* = 8.0, 1.6 Hz, 1H), 7.74 – 7.68 (m, 1H), 7.66 – 7.60 (m, 1H), 7.42 – 7.36 (m, 2H), 7.28 – 7.22 (m, 1H), 6.63 (d, *J* = 8.7 Hz, 1H).

**$^{13}$C NMR** (151 MHz, $CDCl_3$): $\delta_C$ 140.9, 139.0, 132.9, 131.5, 130.6, 129.9, 123.5, 122.7, 122.1, 117.3.

**HRMS** (ESI$^+$): *m/z* calc'd for **PTH-O$_2$** $C_{18}H_{13}NO_2SNa$ [M+Na]$^+$: 330.05592, found: 330.05484.

**Crystal structure:** *see crystal structure*

**10-(4-methoxyphenyl)-10H-phenothiazine (N-(4-OMe-Ph)-PTH)**

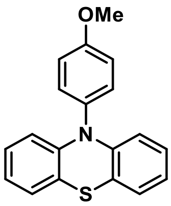


The title compound was synthesized according to a modified literature procedure[14] using 10*H*-phenothiazine (1.26 g, 5.50 mmol, 1.10 equiv.), 1-bromo-4-methoxybenzene (626 µL, 5.00 mmol, 1.00 equiv.), potassium *tert*-butoxide (645 mg, 5.75 mmol, 1.15 equiv.), HP*t*Bu$_3$BF$_4$ (87 mg, 300 µmol, 6 mol%,) and Pd(dba)$_2$ (170 mg, 300 µmol, 6 mol%,) in 1,4-dioxane (20 mL) at 140 °C for 15 h. The reaction was allowed to cool down to room temperature. Water (30 mL) and $CH_2Cl_2$ (20 mL) were added the layer separated and the organic layer was washed with sat. aq. NaCl-solution (30 mL). The mixutre concentrated and purified by column chromatography ($SiO_2$, 100:0 → 98:2 pentane:$Et_2O$) to obtain the title compound as a white solid (1.39 g, 4.55mmol, 91%).

**TLC**: $R_f$ = 0.43 (98:2 pentane:EtOAc)

**NMR Spectroscopy (see spectra):**

**$^{1}$H NMR** (400 MHz, $CD_3CN$): $\delta_H$ 7.39 – 7.30 (m, 2H), 7.25 – 7.16 (m, 2H), 7.04 (dd, *J* = 7.4, 1.7 Hz, 2H), 6.96 – 6.89 (m, 2H), 6.84 (td, *J* = 7.4, 1.3 Hz, 2H), 6.23 (dd, *J* = 8.2, 1.3 Hz, 2H), 3.90 (s, 3H);

**$^{13}$C NMR** (101MHz, $CD_3CN$): $\delta_C$ 160.5, 145.5, 134.1, 128.1, 127.5, 123.4, 120.3, 116.9, 116.7, 56.3.

**GC-MS** (EI$^+$): *m*/*z* calc'd for **N-(4-OMe-Ph)-PTH** $C_{19}H_{12}F_3NS$ [M]$^+$: 343.08744, found: 305.1, 290.1, 273.1, 198.1.

**10-(4-(trifluoromethyl)phenyl)-10H-phenothiazine (N-(4-CF$_3$-Ph)-PTH)**

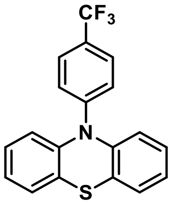


The title compound was synthesized according to a modified literature procedure[14] using 10*H*-phenothiazine (1.26 g, 5.50 mmol, 1.10 equiv.), 1-bromo-4-methoxybenzene (700 µL, 5.00 mmol, 1.00 equiv.), potassium *tert*-butoxide (645 mg, 5.75 mmol, 1.15 equiv.), HP*t*$Bu_3BF_4$ (87 mg, 300 µmol, 6 mol%,) and Pd(dba)$_2$ (170 mg, 300 µmol, 6 mol%,) in 1,4-dioxane (20 mL) at 140 °C for 15 h. The reaction was allowed to cool down to room temperature. Water (30 mL) and $CH_2Cl_2$ (20 mL) were added the layer separated and the organic layer was washed with sat. aq. NaCl-solution (30 mL). The mixutre concentrated and purified by column chromatography ($SiO_2$, 100:0 → 98:2 pentane:$Et_2O$) to obtain the title compound as a white solid (1.43 g, 4.18 mmol, 84%).

**TLC**: $R_f$ = 0.53 (98:2 pentane:EtOAc)

**NMR Spectroscopy (see spectra):**

**$^{1}$H NMR** (400 MHz, $CD_3CN$): $\delta_H$ 7.74 – 7.66 (m, 2H), 7.31 (dt, *J* = 8.5, 2.1 Hz, 4H), 7.20 (d, *J* = 1.6 Hz, 4H), 7.09 (td, *J* = 7.5, 1.3 Hz, 2H), 6.87 (dd, *J* = 8.0, 1.3 Hz, 2H);

**$^{13}$C{$^{19}$F} NMR** (126 MHz, $CD_3CN$): $\delta_C$ 147.0, 142.4, 127.9, 127.5, 127.5, 127.2, 125.5, 125.0, 124.5, 122.8, 122.2;

**$^{19}$F NMR** (376 MHz, $CD_3CN$): $\delta_F$ -62.5.

**GC-MS** (EI$^+$): *m*/*z* calc'd for **N-(4-CF3-Ph)-PTH** $C_{19}H_{12}F_3NS$ [M]$^+$: 343.06426, found: 343.1, 324.1, 311.1, 198.1.

**2-methoxy-10-phenyl-10H-phenothiazine (2-MeO-PTH)**

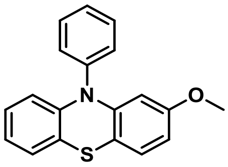


The title compound was synthesized according to a literature procedure[14] using 2-methoxy-10*H*-phenothiazine (1.26 g, 5.50 mmol, 1.10 equiv.), bromobenzene (520 µL, 5.00 mmol, 1.00 equiv.), potassium *tert*-butoxide (645 mg, 5.75 mmol, 1.15 equiv.), HP*t*$Bu_3BF_4$ (87 mg, 300 µmol, 6 mol%,) and Pd(dba)$_2$ (170 mg, 300 µmol,

6 mol%,) in 1,4-dioxane (20 mL) at 140 °C for 15 h. The reaction was allowed to cool down to room temperature. Water (30 mL) and $CH_2Cl_2$ (20 mL) were added the layer separated and the organic layer was washed with sat. aq. NaCl-solution (30 mL). The mixutre concentrated and purified by column chromatography ($SiO_2$, 100:0 → 98:2 pentane:$Et_2O$) to obtain the title compound as a grey solid (1.02 g, 3.33 mmol, 67%).

**TLC**: $R_f$ = 0.54 (98:2 pentane:EtOAc)

**NMR Spectroscopy (*see spectra*):**

**$^1$H NMR** (400 MHz, $CD_3CN$): $\delta_H$ 7.72 – 7.62 (m, 2H), 7.61 – 7.52 (m, 1H), 7.46 – 7.38 (m, 2H), 7.08 (dd, *J* = 7.4, 1.8 Hz, 1H), 6.98 (dd, *J* = 8.4, 1.8 Hz, 1H), 6.96 – 6.83 (m, 2H), 6.49 (dd, *J* = 8.4, 2.5 Hz, 1H), 6.26 (dd, *J* = 8.1, 1.4 Hz, 1H), 5.79 (d, *J* = 2.5 Hz, 1H), 3.59 (s, 3H);

**$^{13}$C NMR** (101MHz, $CD_3CN$): $\delta_C$ 160.4, 146.4, 144.8, 141.9, 131.8, 131.4, 129.4, 128.1, 127.9, 127.6, 123.7, 121.6, 117.3, 111.8, 108.0, 104.9, 55.8.

**HRMS** (ESI): *m*/*z* calculated for **2-MeO-PTH** $C_{19}H_{15}NOSNa^+$ $[M+Na]^+$: 328.07666, found: 328.07671.

**10-phenyl-2-(trifluoromethyl)-10H-phenothiazine (2-CF$_3$-PTH)**

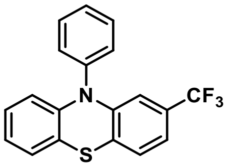


The title compound was synthesized according to a literature procedure[14] using 2-(trifluoromethyl)-10*H*-phenothiazine (1.46 g, 5.50 mmol, 1.10 equiv.), bromobenzene (520 µL, 5.00 mmol, 1.00 equiv.), potassium *tert*-butoxide (645 mg, 5.75 mmol, 1.15 equiv.), HP*t*$Bu_3BF_4$ (87 mg, 300 µmol, 6 mol%,) and $Pd(dba)_2$ (170 mg, 300 µmol, 6 mol%,) in 1,4-dioxane (20 mL) at 140 °C for 15 h. The reaction was allowed to cool down to room temperature. Water (30 mL) and $CH_2Cl_2$ (20 mL) were added the layer separated and the organic layer was washed with sat. aq. NaCl-solution (30 mL). The mixutre concentrated and purified by column chromatography ($SiO_2$, 100:0 → 98:2 pentane:$Et_2O$) to obtain the title compound as a yellow viscous oil (1.43 g, 4.16 mmol, 83%).

**TLC**: $R_f$ = 0.71 (98:2 pentane:EtOAc)

**NMR Spectroscopy (*see spectra*):**

**$^1$H NMR** (400 MHz, $CD_3CN$): $\delta_H$ 7.71 – 7.66 (m, 2H), 7.64 – 7.55 (m, 1H), 7.43 (dq, *J* = 6.7, 1.2 Hz, 2H), 7.16 (dd, *J* = 7.9, 1.2 Hz, 1H), 7.08 (ddd, *J* = 7.9, 2.0, 0.9 Hz, 1H), 7.02 (dd, *J* = 7.4, 1.7 Hz, 1H), 6.95 – 6.81 (m, 2H), 6.32 – 6.27 (m, 1H), 6.17 (dd, *J* = 8.1, 1.5 Hz, 1H);

**$^{13}$C{$^{19}$F} NMR** (126 MHz, $CD_3CN$): $\delta_C$ 145.6, 144.3, 141.0, 132.3, 131.6, 130.0, 129.5, 128.6, 128.0, 127.6, 126.0, 125.0, 124.2, 119.9, 119.5, 117.3, 112.5;

**$^{19}$F NMR** (376 MHz, $CD_3CN$): $\delta_F$ -63.7.

**HRMS** (ESI): *m*/*z* calculated for **2-CF3-PTH** $C_{19}H_{11}NSF_3$ $[M-H]^-$: 342.05698, found: 342.05667.

## 2.4. Reaction Optimization

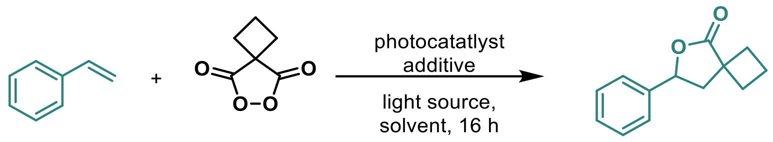


**1a** **2a** **3a**

Reactions were performed using **1a** (0.10 mmol) according to a modified <u>**General Procedure A**</u>. Modifications to standard conditions and key observations from each study are stated.

### 2.4.1. Establishing the photocatalyst

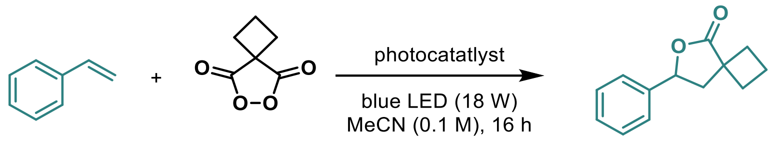


**1a** **2a** **3a**

**Table S1:** Photocatalyst optimization studies for the photocatalytic reaction between styrene and cyclobutane malonyl peroxide 2a.

| entry | photocatalyst | yield | rSM |
|---|---|---|---|
| #1 | [Ir(ppy)$_2$(dtbpy)]PF$_6$ (1 mol%, 450 nm) | traces | 67% |
| #2 | [Ir(mppy)$_2$(dtbpy)]PF$_6$ (1 mol%, 450 nm) | traces | 73% |
| #3 | [Ir(dFCF$_3$ppy)$_2$(dtbpy)]PF$_6$ (1 mol%, 450 nm) | 16% | 30% |
| #4 | Ir(ppy)$_3$ (1 mol%, 450 nm) | 34% | 47% |
| #5 | **4CzIPN** (10 mol%, 405 nm) | - | 74% |
| #6 | **TXT** (10 mol%, 405 nm) | 13% | 30% |
| #7 | **PTH** (10 mol%, 405 nm) | 50% | 9% |
| #8 | **PTH-2** (10 mol%, 405 nm) | 18% | 52% |
| #9 | without photocatalyst | traces | 70% |
| #10 | **PTH** (10 mol%, 405 nm), without light | 11% | 46% |

Reaction performed on a 0.1 mmol scale using styrene and **2a** (2 equiv.) in MeCN (0.1 M). The vessel was irradiated with blue LEDs (18 W) for 16 h. Product yields and styrene rSM determined by GC-FID using mesitylene as internal standard.

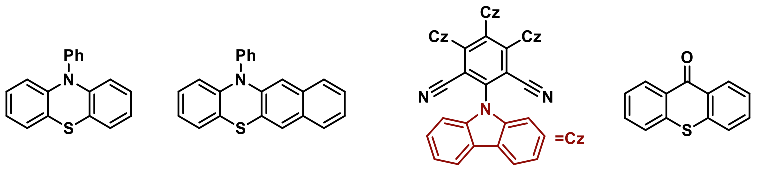


**PTH** **PTH-2** **4CzIPN** **TXT**

**Key observations**: Upon screening photocatalysts across a wide range of oxidation potentials, it was demonstrated that **PTH** was most effectively promoting product formation, whereas other photocatalysts such as Ir-F, Ir(ppy3) and **TXT** afforded lower yields. Thereby, a reductive cleavage of the peroxide bond by reducing photocatalyst was assumed and the most successful, organic photocatalyst was further investigated.

### 2.4.2. Investigation of the solvent

**Table S2:** Solvent optimization for the reaction between **1a** and **2a** catalyzed by **PTH**.

| entry | solvent (0.1 M) | yield | rSM |
|---|---|---|---|
| #1 | MeCN | 50% | 9% |
| #2 | $PhCH_3$ | 24% | 40% |
| #3 | DMF | 35% | 30% |
| #4 | EtOAc | 16% | 62% |
| #5 | DME | 20% | 34% |
| #6 | DCE | 51% | 2% |
| #7 | $CHCl_3$ | 43% | 2% |
| #8 | $PhCF_3$ | 31% | 15% |
| #9 | 2-Me-THF | 30% | 19% |
| #10 | dioxane | 19% | 15% |
| #11 | dimethyl carbonate | 19% | 14% |

Reaction performed on a 0.1 mmol scale using styrene, **2a** (2 equiv.) and **PTH** (10 mol%), irradiated with 405 nm LEDs (18 W) for 16 h. Product yields and styrene rSM determined by GC-FID using mesitylene as internal standard.

**Additional Note:** The water content of the solvent was determined using Karl-Fischer. MeCN (30 ppm), $CHCl_3$ (1.1 ppm), DCE (0.8 ppm), DMF (23.4 ppm), $PhCH_3$ (2.4 ppm)

### 2.4.3. Optimization of the stoichiometry

**Table S3:** Peroxide equivalents and photocatalyst loading optimization for the photocatalytic reaction between styrene and cyclobutane malonyl peroxide **2a** catalyzed by **PTH**.

| entry | peroxide equivalents | photocatalyst loading | yield | rSM |
|---|---|---|---|---|
| #1 | 1.0 equiv. | 10 mol% | 12% | 81% |
| #2 | 1.5 equiv. | 10 mol% | 28% | 55% |
| #3 | 2.0 equiv. | 10 mol% | 42% | 33% |
| #4 | 3.0 equiv. | 10 mol% | 69% | 0% |
| #5 | 5.0 equiv. | 10 mol% | 55% | 4% |
| #6 | 3.0 equiv. | 1 mol% | 24% | 0% |
| #7 | 3.0 equiv. | 5 mol% | 26% | 0% |
| #8 | 3.0 equiv. | 10 mol% | 40% | 0% |
| #9 | 3.0 equiv. | 25 mol% | 61% | 4% |
| #10 | 2.0 equiv. | 25 mol% | 59% | 0% |
| #11 [a] | 2.0 equiv. | 5 mol% | traces | 55% |
| #12 [b] | 2.0 equiv. | 1 mol% | 36% | 0% |
| #13 [b] | 2.0 equiv. | 5 mol% | 48% | 0% |

Reaction performed on a 0.1 mmol scale in MeCN (0.1 M) using styrene, **2a** and **PTH**, irradiated with 405 nm LEDs (18 W) for 16 h. Product yields and styrene rSM determined by GC-FID using mesitylene as internal standard. [a] Reaction set up and performed under exclusion of light at 30 °C. [b] Reaction performed under optimized conditions, using 3.0 equiv. of water additive, MeCN (0.2 M) as solvent and 405 nm LEDs (18 W) without fan for 16 h.

### 2.4.4. Investigation of the influence of water

**Table S4:** Water additive optimization studies for the photocatalytic reaction between styrene and cyclobutane malonyl peroxide **2a** catalyzed by **PTH**.

| entry | variation | Yield% | rSM |
|---|---|---|---|
| #1 | flame-dried glassware, dried (4 Å molecular sieves) reagents, added 3 Å molecular sieves to reaction | 30 | 0 |
| #2 | oven-dried (80 °C) glassware, dry solvent, Alox-filtered styrene | 42 | 0 |
| #3 | non-dried glassware, Alox-filtered styrene, added 5.4 equiv. $H_2O$ (10 µL) | 59 | 0 |
| #4 [a] | standard conditions, added 0.3 equiv. $H_2O$ (1 µL) | 57 | 11 |
| #5 [a] | standard conditions, added 1.4 equiv. $H_2O$ (5 µL) | 59 | 7 |
| #6 [a] | standard conditions, added 2.7 equiv. $H_2O$ (10 µL) | 67 | 0 |
| #7 [a] | standard conditions, added 5.0 equiv. $H_2O$ (18 µL) | 69 | 0 |
| #8 [a] | MeCN (0.2 M), added 3.0 equiv. $H_2O$ (11 µL) | 79 | 0 |

Reaction performed on a 0.1 mmol scale in MeCN (0.2 M) using styrene, **2a** (3.0 eq) and **PTH** (10 mol%), irradiated with 405 nm LEDs (18 W) for 16 h. Product yields and styrene rSM determined by GC-FID using mesitylene as internal standard. [a] Reaction performed on a 0.2 mmol scale using 2.0 equiv. of **2a**.

**Key observations**: Interestingly, the addition of water increased both the yield and the reproducibility of the reaction, whereas the utilization of flame-dried glassware, dry solvents and additional drying agent even lowered the yield. The necessity of water is explained by the formation of **PTH-O** using water (see manuscript for discussion). Increasing the concentration of the reaction to 0.2 M further boosted the reactivity.

### 2.4.5. Screening of light sources and additives

**Table S5:** Optimization studies for the photocatalytic reaction between styrene and cyclobutane malonyl peroxide **2a** catalyzed by **PTH**. Screening of light sources and additives.

| entry | variation | yield | rSM |
|---|---|---|---|
| #1 | Additive $LiBF_4$ (10 mol%) | 26% | 36% |
| #2 | Additive formic acid (1.0 equiv.) | traces | 81% |
| #3 | Additive $NEt_3$ (1.0 equiv.) | - | 92% |
| #4 | 365 nm (30 W) instead of 405 nm | 30% | 20% |
| #5 | 425 nm (18 W) instead of 405 nm | 37% | 4% |

Reaction performed on a 0.1 mmol scale in MeCN (0.2 M) using styrene, **2a** (3.0 eq) and **PTH** (10 mol%), irradiated with 405 nm LEDs (18 W) for 16 h. Product yields and styrene rSM determined by GC-FID using mesitylene as internal standard.

**Key observations**: None of the tested additives resulting in a yield improvement. However, quite a broad variation in the irradiation wavelength was tolerated, with even 425 nm LEDs enabling product formation. Since the dark control reaction (**Table S1**) showed no product formation, but the **PTH** photocatalyst is known to only absorb light with wavelengths below 380 nm, this prompted us to further investigate the reaction mechanism.

### 2.4.6. Screening of photocatalyst for electron-deficient styrenes

To further enhance the yield of low-yielding, electron-deficient substrates, we tested several derivatives of the **PTH** photocatalyst in the reaction. All reactions were set up according to general procedure A, the summarized results are given below.

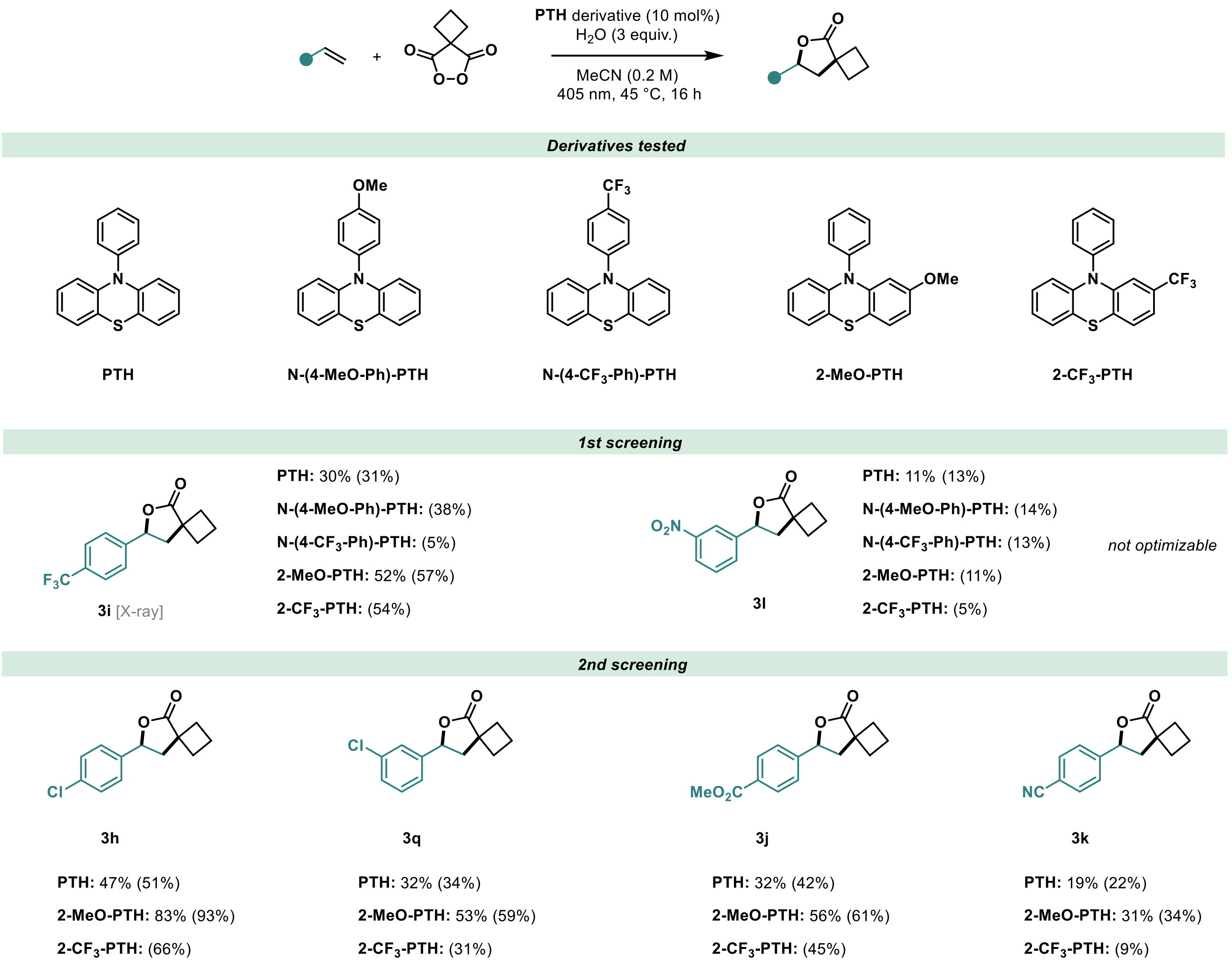


**Figure S5a:** Screening of different PTH-derivatives for yield improvements with electron-deficient styrenes

**Discussion:** As observed in the first screening, introducing any substituents at the N-phenyl substituent did not improve the yield for any of the substrates tested. In general, obtaining optimized results for *m*-$NO_2$-styrene was not successful. **2-MeO-PTH** was generally resulting in the highest yield improvements for previously unsuccessful substrates, also for substrates **3h**, **3q**, **3j** and **3k**. The electron-deficient **2-$CF_3$-PTH**, however, also did increase the yields of substrates **3i**, **3h**, and **3j** notably. This contradicts the hypothesis that the improvement is caused by better-matching redox-potentials of the photocatalyst (where a trend would be observed) – instead we believe that any substituent introduced at the phenothiazine core enhances the stability of the photocatalytically active sulfoxide. This was also observed in an NMR-tracked competition experiment (Figure S5b), awhere an equimolar mixture of **PTH** and **2-$CF_3$-PTH** was subjected to increasing amounts of **2a** at room temperature. In the NMR spectra, it was observed that the unsubstituted **PTH-O** degraded more rapidly compared to it's substituted analogues.

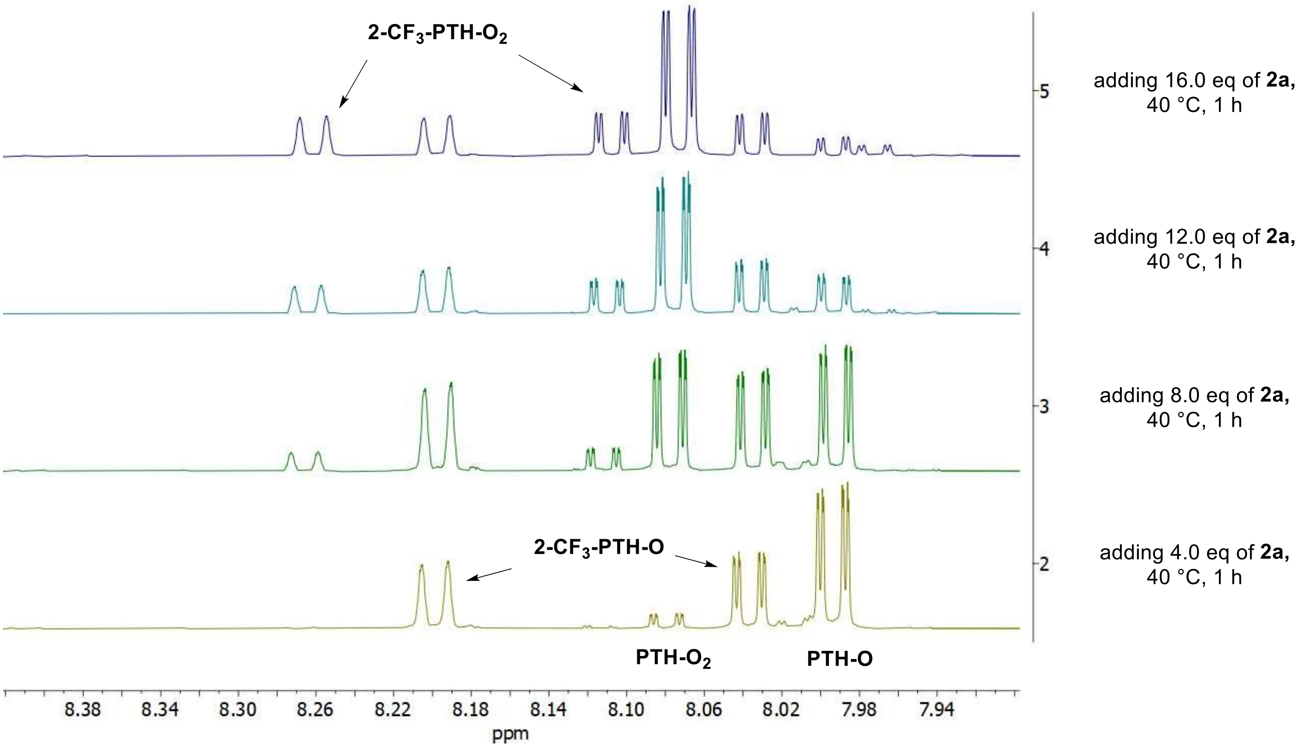


**Figure S5b:** Competition experiment for the degradation of **PTH-O** and **2-$CF_3$-PTH-O**.

## 2.5 Synthesis of $\gamma$-Lactones

### 7-phenyl-6-oxaspiro[3.4]octan-5-one (3a)

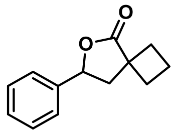

Synthesised following General Procedure A using: styrene (57 µL, 0.5 mmol, 1.0 equiv) and 6,7-dioxaspiro[3.4]octane-5,8-dione **2a** (142 mg, 1.00 mmol, 2.0 equiv) in MeCN. Purified by flash column chromatography ($SiO_2$; 95:5 → 90:10 pentane:EtOAc) to afford **3a** (77 mg, 0.38 mmol, 76%) as a white solid.

**GC-FID yield**: 79%

**TLC**: $R_f$ =0.36 (90:10 pentane:EtOAc)

**NMR Spectroscopy (*see spectra*):**

> **$^1$H NMR** (599 MHz, $CDCl_3$): $\delta_H$ 7.38 – 7.34 (m, 2H), 7.33 – 7.29 (m, 3H), 5.34 (dd, *J* = 8.8, 6.3 Hz, 1H), 2.76 (dd, *J* = 12.9, 6.3 Hz, 1H), 2.61 – 2.49 (m, 2H), 2.26 (dd, *J* = 12.9, 8.8 Hz, 1H), 2.19 – 2.11 (m, 2H), 2.05 – 1.99 (m, 1H), 1.96 – 1.91 (m, 1H);

> **$^{13}$C NMR** (151 MHz, $CDCl_3$): $\delta_C$ 180.8, 139.6, 128.8, 128.4, 125.4, 78.0, 44.8, 44.8, 31.7, 29.4, 16.6.

**HRMS** (ESI$^+$): *m*/*z* calc'd for **3a** $C_{14}H_{16}O_2Na$ $[M+Na]^+$: 225.08860, found: 225.08844.

**Crystal structure:** *see crystal structure*

### 7-(p-tolyl)-6-oxaspiro[3.4]octan-5-one (3b)

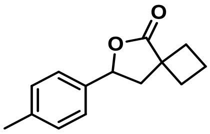

Synthesised following General Procedure A using: 4-Methylstyrene (33 µL, 0.25 mmol, 1.0 equiv) and 6,7-dioxaspiro[3.4]octane-5,8-dione **2a** (71 mg, 0.50 mmol, 2.0 equiv) in MeCN. Purified by flash column chromatography ($SiO_2$; 100:0 → 95:5 pentane:EtOAc) to afford **3b** (27 mg, 0.125 mmol, 50%) as a colourless solid.

**GC-FID yield**: 65%

**TLC**: $R_f$ =0.31 (95:5 pentane:EtOAc)

**NMR Spectroscopy (*see spectra*):**

> **$^1$H NMR** (599 MHz, $CDCl_3$): $\delta_H$ 7.21 – 7.17 (m, 4H), 5.31 (dd, *J* = 8.9, 6.2 Hz, 1H), 2.74 (dd, *J* = 12.9, 6.3 Hz, 1H), 2.63 – 2.55 (m, 1H), 2.55 – 2.50 (m, 1H), 2.35 (s, 3H), 2.25 (dd, *J* = 12.9, 8.9 Hz, 1H), 2.20 – 2.10 (m, 2H), 2.08 – 1.99 (m, 1H), 1.98 – 1.92 (m, 1H);

> **$^{13}$C NMR** (151 MHz, $CDCl_3$): $\delta_C$ 180.9, 138.3, 136.5, 129.4, 125.5, 78.1, 44.7, 31.7, 29.3, 21.3, 16.6.

**HRMS** (ESI$^+$): *m*/*z* calc'd for **3b** $C_{14}H_{16}O_2Na$ $[M+Na]^+$: 239.10425, found: 239.10419.

**7-(4-methoxyphenyl)-6-oxaspiro[3.4]octan-5-one (3c)**

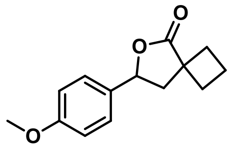

Synthesised following General Procedure A using: styrene (34 mL, 0.25 mmol, 1.0 equiv) and 6,7-dioxaspiro[3.4]octane-5,8-dione **2a** (71 mg, 0.5 mmol, 2.0 equiv) in MeCN. Purified by flash column chromatography ($SiO_2$; 95:5 → 75:25 pentane:EtOAc) to afford 3c (39 mg, 0.17 mmol, 65%) as a yellow solid.

**GC-FID yield**: 72%

**TLC**: $R_f$ =0.73 (80:20 pentane:EtOAc)

**NMR Spectroscopy (*see spectra*):**

$^1$**H NMR** (500 MHz, $CDCl_3$): $\delta_H$ 7.25 – 7.20 (m, 2H), 6.90 – 6.84 (m, 2H), 5.28 (dd, $J$ = 9.1, 6.1 Hz, 1H), 3.79 (s, 3H), 2.71 (dd, $J$ = 12.9, 6.1 Hz, 1H), 2.62 – 2.56 (m, 1H), 2.54 – 2.47 (m, 1H), 2.25 (dd, $J$ = 12.9, 9.1 Hz, 1H), 2.19 – 2.10 (m, 2H), 2.06 – 1.92 (m, 2H);

$^{13}$**C NMR** (126 MHz, $CDCl_3$): $\delta_C$ 180.8, 159.8, 131.2, 127.1, 114.1, 78.0, 55.4, 44.9, 44.6, 31.7, 29.2, 16.6.

**HRMS** (ESI$^+$): $m/z$ calc'd for **3c** $C_{14}H_{16}O_3Na$ $[M+Na]^+$: 255.09917, found: 255.09914.

**7-(4-(tert-butoxy)phenyl)-6-oxaspiro[3.4]octan-5-one (3d)**

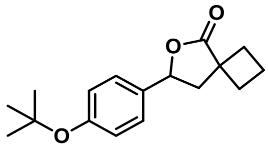

Synthesised following General Procedure A using: 1-(tert-butoxy)-4-vinylbenzene (47 µL, 0.50 mmol, 1.0 equiv.) and 6,7-dioxaspiro[3.4]octane-5,8-dione **2a** (142 mg, 1.0 mmol, 2.0 equiv) in MeCN. Purified by flash column chromatography ($SiO_2$; 100:0 → 90:10 pentane:EtOAc) to afford **3d** (49 mg, 0.18 mmol, 73%) as a colourless solid.

**GC-FID yield**: 75%

**TLC**: $R_f$ = 0.40 (90:10 pentane:EtOAc)

**NMR Spectroscopy (*see spectra*):**

$^1$**H NMR** (599 MHz, $CDCl_3$): $\delta_H$ 7.22 – 7.15 (m, 2H), 6.99 – 6.94 (m, 2H), 5.29 (dd, $J$ = 9.1, 6.1 Hz, 1H), 2.73 (dd, $J$ = 13.0, 6.2 Hz, 1H), 2.63 – 2.54 (m, 1H), 2.53 – 2.46 (m, 1H), 2.26 (dd, $J$ = 12.9, 9.1 Hz, 1H), 2.20 – 2.09 (m, 2H), 2.07 – 1.90 (m, 2H), 1.33 (s, 9H);

$^{13}$**C NMR** (151 MHz, $CDCl_3$): $\delta_C$ 180.8, 155.7, 133.9, 126.3, 124.2, 78.8, 78.0, 44.9, 44.6, 31.7, 29.2, 28.9, 16.6.

**HRMS** (ESI$^+$): $m/z$ calc'd for **3d** $C_{17}H_{22}O_3Na$ $[M+Na]^+$: 297.14612, found: 297.14599.

**7-(4-methoxyphenyl)-6-oxaspiro[3.4]octan-5-one-8,8-*d*$_2$ (3e)**

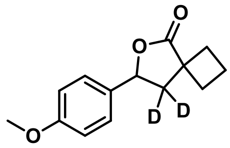

Synthesised following General Procedure A using 1-methoxy-4-(vinyl-2,2-*d*$_2$)benzene **1e** (34 mg, 0.25mmol, 1.0 equiv) and 6,7-dioxaspiro[3.4]octane-5,8-dione **2a** (71 mg, 0.50 mmol, 2.0 equiv) in MeCN. Purified by flash column chromatography ($SiO_2$; 98:2 → 80:20 pentane:EtOAc) to afford **3e** (31 mg, 0.13 mmol, 53%) as a white solid.

**GC-FID yield**: 56%

**TLC**: $R_f$ =0.71 (80:20 pentane:EtOAc)

**NMR Spectroscopy (*see spectra*):**

$^1$**H NMR** (500 MHz, $CDCl_3$): $\delta_H$ 7.27 – 7.22 (m, 2H), 6.94 – 6.87 (m, 2H), 5.30 (s, 1H), 3.82 (s, 3H), 2.65 – 2.57 (m, 1H), 2.57 – 2.49 (m, 1H), 2.23 – 2.11 (m, 2H), 2.09 – 1.94 (m, 2H);

$^{13}$**C NMR** (126 MHz, $CDCl_3$): $\delta_C$ 180.9, 159.8, 131.3, 127.1, 114.2, 78.0, 55.5, 44.8, 31.7, 29.2, 16.7; *one carbon is not visible since the coupling to the deuterium.*

**HRMS** (ESI$^+$): *m/z* calc'd for **3e** $C_{14}H_{14}O_3D_2Na$ [M+Na]$^+$: 257.11172, found: 257.11175.

**7-(4-methoxyphenyl)-6-oxaspiro[3.4]octan-5-one-7-*d* (3f)**

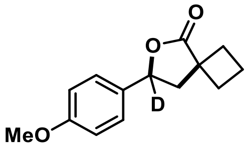

Synthesised following General Procedure A using 1-methoxy-4-(vinyl-1-*d*)benzene **1f** (34 mg, 0.25mmol, 1.0 equiv) and 6,7-dioxaspiro[3.4]octane-5,8-dione **2a** (71 mg, 0.50 mmol, 2.0 equiv) in MeCN. Purified by flash column chromatography ($SiO_2$; 98:2 → 80:20 pentane:EtOAc) to afford **3f** (35 mg, 0.15 mmol, 61%) as a white solid.

**GC-FID yield**: 66%

**TLC**: $R_f$ =0.67 (80:20 pentane:EtOAc)

**NMR Spectroscopy (*see spectra*):**

$^1$**H NMR** (599 MHz, $CDCl_3$): $\delta_H$ 7.25 – 7.22 (m, 2H), 6.91 – 6.88 (m, 2H), 3.80 (d, $J$ = 0.8 Hz, 3H), 2.71 (d, $J$ = 12.9 Hz, 1H), 2.65 – 2.56 (m, 1H), 2.55 – 2.48 (m, 1H), 2.25 (d, $J$ = 12.9 Hz, 1H), 2.19 – 2.11 (m, 2H), 2.08 – 1.92 (m, 2H).

$^{13}$**C NMR** (151 MHz, $CDCl_3$): $\delta_C$ 180.9, 159.8, 131.2, 127.1, 114.2, 77.7 (t, $J$ = 23.3 Hz) 55.4, 45.0, 44.5, 31.7, 29.2, 16.6.

**HRMS** (ESI$^+$): *m/z* calc'd for **3f** $C_{14}H_{15}O_3DNa$ [M+Na]$^+$: 256.10544, found: 256.10541.

**7-(4-bromophenyl)-6-oxaspiro[3.4]octan-5-one (3g)**

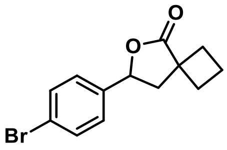

Synthesised following General Procedure A using: 4-bomo-styrene (33 μL, 0.25 mmol, 1.0 equiv) and 6,7-dioxaspiro[3.4]octane-5,8-dione **2a** (71 mg, 0.50 mmol, 2.0 equiv) in DCE. Purified by flash column chromatography ($SiO_2$; 80:15:5 pentane:$CH_2Cl_2$:EtOAc) to afford **3g** (27 mg, 0.098 mmol, 39%) as a white solid.

**GC-FID yield**: 43%

**TLC**: $R_f$ =0.36 (80:15:5 pentane:$CH_2Cl_2$:EtOAc)

**NMR Spectroscopy (*see spectra*):**

$^1$**H NMR** (599 MHz, $CDCl_3$): $\delta_H$ 7.38 – 7.34 (m, 2H), 7.33 – 7.29 (m, 3H), 5.34 (dd, $J$ = 8.8, 6.3 Hz, 1H), 2.76 (dd, $J$ = 12.9, 6.3 Hz, 1H), 2.61 – 2.49 (m, 2H), 2.26 (dd, $J$ = 12.9, 8.8 Hz, 1H), 2.19 – 2.11 (m, 2H), 2.05 – 1.99 (m, 1H), 1.96 – 1.91 (m, 1H);

$^{13}$**C NMR** (151 MHz, $CDCl_3$): $\delta_C$ 180.8, 139.6, 128.86 128.4, 125.4, 78.0, 44.8, 44.8, 31.7, 29.4, 16.6.

**HRMS** (ESI$^+$): *m*/*z* calc'd for **3g** $(C_{13}H_{13}O_2Br)_2Na$ $[2M+Na]^+$: 585.00716, found: 585.00701.

**Crystal structure:** *see crystal structure*

**7-(4-chlorophenyl)-6-oxaspiro[3.4]octan-5-one (3h)**

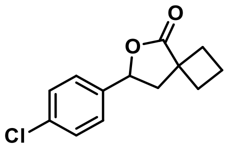

Synthesised following General Procedure A using: 4-chloro-styrene (32 μL, 0.25 mmol, 1.0 equiv.) and 6,7-dioxaspiro[3.4]octane-5,8-dione **2a** (71 mg, 0.50 mmol, 2.0 equiv) in DCE. Purified by flash column chromatography ($SiO_2$; 95:5 → 90:10 pentane:EtOAc) to afford **3h** (32 mg, 0.12 mmol, 47%) as a yellow solid.

**GC-FID yield**: 51%

**Variation:** Using **2-MeO-PTH** instead of **PTH** as a photocatalyst in general procedure A, the product **3h** was isolated in a higher yield of 83% (56 mg, 0.21 mmol).

**TLC**: $R_f$ = 0.47 (90:10 pentane:EtOAc)

**NMR Spectroscopy (*see spectra*):**

$^1$**H NMR** (500MHz, $CDCl_3$): $\delta_H$ 7.37 – 7.32 (m, 2H), 7.26 – 7.23 (m, 2H), 5.32 (dd, $J$ = 8.8, 6.3 Hz, 1H), 2.77 (dd, $J$ = 13.0, 6.3 Hz, 1H), 2.62 – 2.44 (m, 2H), 2.28 – 2.10 (m, 3H), 2.07 – 1.98 (m, 1H), 1.98 – 1.91 (m, 1H);

$^{13}$**C NMR** (126 MHz, $CDCl_3$): $\delta_C$ 180.6, 138.1, 134.3, 129.0, 126.8, 77.3, 44.7, 44.7, 31.6, 29.4, 16.6.

**HRMS** (ESI$^+$): *m/z* calc'd for **3h** $C_{13}H_{13}O_2ClNa$ [M+Na]$^+$: 259.04963, found: 259.04957.

**Crystal structure:** *see crystal structure*

**7-(4-(trifluoromethyl)phenyl)-6-oxaspiro[3.4]octan-5-one (3i)**

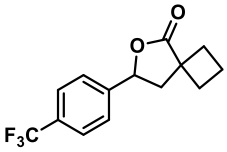

Synthesised following General Procedure A using: 4-trifluoromethyl-styrene (39 µL, 0.25mmol, 1.0 equiv) and 6,7-dioxaspiro[3.4]octane-5,8-dione **2a** (71 mg, 0.50 mmol, 2.0 equiv) in DCE. Purified by flash column chromatography ($SiO_2$; 100:0 → 90:10 pentane:EtOAc) to afford **3i** (20 mg, 0.07 mmol, 30%) as a white solid.

**GC-FID yield**: 31%

**Variation:** Using **2-MeO-PTH** instead of **PTH** as a photocatalyst in general procedure A, the product **3i** was isolated in a higher yield of 52% (35 mg, 0.13 mmol).

**TLC**: $R_f$ =0.61 (90:10 pentane:EtOAc)

**NMR Spectroscopy (*see spectra*):**

$^1$**H NMR** (599 MHz, $CDCl_3$): $\delta_H$ 7.66 – 7.62 (m, 2H), 7.47 – 7.41 (m, 2H), 5.40 (dd, *J* = 8.7, 6.5 Hz, 1H), 2.83 (dd, *J* = 12.9, 6.5 Hz, 1H), 2.65 – 2.49 (m, 2H), 2.29 – 2.12 (m, 3H), 2.08 – 2.00 (m, 1H), 1.98 – 1.91 (m, 1H);

$^{13}$**C NMR** (151 MHz, $CDCl_3$): $\delta_C$. 180.3, 143.6 (d, *J* = 1.3 Hz), 130.5 (d, *J* = 32.6 Hz), 125.8 (d, *J* = 3.8 Hz), 125.4, 123.9 (q, *J* = 272.1 Hz), 76.9, 44.5, 44.5, 31.5, 29.3, 16.5;

$^{19}$**F NMR** (564 MHz, $CDCl_3$) $\delta_F$ -62.7.

**HRMS** (ESI$^+$): *m/z* calc'd for **3i** $C_{14}H_{13}O_2F_3Na$ [M+Na], 293.07599, found: 293.07597.

**Crystal structure:** *see crystal structure*

**methyl 4-(5-oxo-6-oxaspiro[3.4]octan-7-yl)benzoate (3j)**

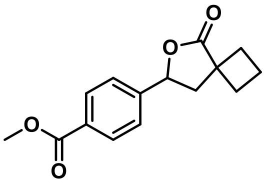

Synthesised following General Procedure A using: methyl 4-vinylbenzoate (41 mg, 0.25 mmol, 1.0 equiv) and 6,7-dioxaspiro[3.4]octane-5,8-dione **2a** (85 mg, 0.6 mmol, 2.0 equiv) in DCE. Purified by flash column chromatography ($SiO_2$; 100:0 → 90:10 pentane:EtOAc) to afford **3j** (21 mg, 0.08 mmol, 32%) as a white solid.

**GC-FID yield**: 42%

**Variation:** Using **2-MeO-PTH** instead of **PTH** as a photocatalyst in general procedure A, the product **3j** was isolated in a higher yield of 56% (37 mg, 0.14 mmol).

**TLC**: $R_f$ = 0.39 (90:10 pentane:EtOAc)

**NMR Spectroscopy (*see spectra*):**

$^1$**H NMR** (599 MHz, $CDCl_3$): $\delta_H$ 8.07 – 8.01 (m, 2H), 7.41 – 7.35 (m, 2H), 5.40 (dd, *J* = 8.6, 6.5 Hz, 1H), 3.92 (s, 3H), 2.82 (dd, *J* = 12.9, 6.5 Hz, 1H), 2.63 – 2.50 (m, 2H), 2.24 (dd, *J* = 12.9, 8.6 Hz, 1H), 2.21 – 2.12 (m, 2H), 2.09 – 1.99 (m, 1H), 1.96 – 1.89 (m, 1H);

$^{13}$**C NMR** (151 MHz, $CDCl_3$): $\delta_C$ 180.5, 166.7, 144.7, 130.2, 130.2, 125.2, 77.3, 52.3, 44.7, 44.6, 31.6, 29.5, 16.6.

**HRMS** (ESI$^+$): *m*/*z* calc'd for **3j** $C_{15}H_{16}O_4Na$ [M+Na]$^+$: 283.09408, found: 283.09401.

**Crystal structure:** see crystal structure

### 4-(5-oxo-6-oxaspiro[3.4]octan-7-yl)benzonitrile (3k)

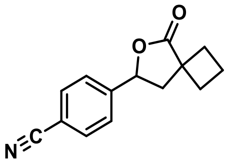

Synthesised following General Procedure A using: 4-cyano-styrene (39 µL, 0.300 mmol, 1.00 equiv) and 6,7-dioxaspiro[3.4]octane-5,8-dione **2a** (85 mg, 0.6 mmol, 2.0 equiv) in DCE. Purified by flash column chromatography ($SiO_2$; 95:5 → 90:10 pentane:EtOAc) to afford **3k** (11.0 mg, 0.05 mmol, 15%) as a yellow solid.

**GC-FID yield**: 22%

**Variation:** Using **2-MeO-PTH** instead of **PTH** as a photocatalyst in general procedure A (0.25 mmol scale), the product **3k** was isolated in a higher yield of 31% (18 mg, 0.08 mmol).

**TLC**: $R_f$ = 0.35 (90:10 pentane:EtOAc)

**NMR Spectroscopy (*see spectra*):**

$^1$**H NMR** (500 MHz, $CDCl_3$): $\delta_H$ 7.75 – 7.62 (m, 2H), 7.47 – 7.38 (m, 2H), 5.38 (dd, *J* = 8.7, 6.5 Hz, 1H), 2.83 (dd, *J* = 12.9, 6.5 Hz, 1H), 2.66 – 2.46 (m, 2H), 2.23 – 2.13 (m, 3H), 2.08 – 1.98 (m, 1H), 1.97 – 1.89 (m, 1H);

$^{13}$**C NMR** (126 MHz, $CDCl_3$): $\delta_C$ 180.2, 145.0, 132.7, 125.9, 118.5, 112.4, 76.8, 44.5, 44.5, 31.6, 29.4, 16.6.

**HRMS** (ESI$^+$): *m*/*z* calc'd for **3k** $C_{14}H_{13}NO_2H$ [M+H]$^+$: 228.10191, found: 228.10185.

**7-(3-nitrophenyl)-6-oxaspiro[3.4]octan-5-one (3l)**

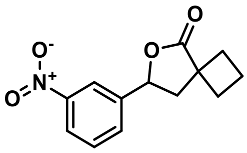

Synthesised following General Procedure A using: 1-nitro-3-vinylbenzene (35 µL, 0.25 mmol, 1.0 equiv) and 6,7-dioxaspiro[3.4]octane-5,8-dione **2a** (85 mg, 0.6 mmol, 2.0 equiv) in DCE. Purified by flash column chromatography ($SiO_2$; 95:5 → 80:20 pentane:EtOAc) to afford **3l** (6.8 mg, 0.028 mmol, 11%) as a yellow liquid.

**GC-FID yield**: 13%

**TLC**: $R_f$ = 0.34 (80:20 pentane:EtOAc)

**NMR Spectroscopy (*see spectra*):**

$^1$**H NMR** (599 MHz, $CDCl_3$): $\delta_H$ 8.19 (m, 2H), 7.72 – 7.66 (m, 1H), 7.60 – 7.55 (m, 1H), 5.43 (dd, *J* = 9.0, 6.4 Hz, 1H), 2.88 (dd, *J* = 12.9, 6.3 Hz, 1H), 2.64 – 2.51 (m, 2H), 2.25 (dd, *J* = 13.0, 9.0 Hz, 1H), 2.23 – 2.15 (m, 2H), 2.10 – 2.00 (m, 1H), 2.00 – 1.93 (m, 1H);

$^{13}$**C NMR** (151 MHz, $CDCl_3$): $\delta_C$. 180.1, 148.6, 141.9, 131.4, 130.0, 123.4, 120.5, 76.6, 44.6, 44.6, 31.7, 29.3, 16.6.

**HRMS** (ESI$^+$): *m*/*z* calc'd for **3l** $C_{13}H_{13}NO_4Na$ [M+Na]$^+$: 270.07368, found: 270.07367.

**7-(o-tolyl)-6-oxaspiro[3.4]octan-5-one (3m)**

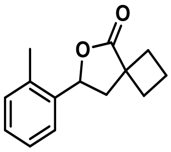

Synthesised following General Procedure A using: 2-methyl-styrene (39 µL, 0.30 mmol, 1.0 equiv.) and 6,7-dioxaspiro[3.4]octane-5,8-dione **2a** (85 mg, 0.60 mmol, 2.0 equiv) in MeCN. Purified by flash column chromatography ($SiO_2$; 95:5 → 90:10 pentane:EtOAc) to afford **3m** (22.0 mg, 0.10 mol, 34%) as a white solid.

**GC-FID yield**: 50%

**TLC**: $R_f$ = 0.44; (90:10 pentane:EtOAc)

**NMR Spectroscopy (*see spectra*):**

$^1$**H NMR** (500 MHz, $CDCl_3$): $\delta_H$ 7.35 – 7.30 (m, 1H), 7.24 – 7.19 (m, 2H), 7.19 – 7.15 (m, 1H), 5.54 (dd, *J* = 6.5, 0.6 Hz, 1H), 2.79 (dd, *J* = 12.9, 6.5 Hz, 1H), 2.65 – 2.53 (m, 2H), 2.34 (s, 3H), 2.24 – 2.12 (m, 3H), 2.07 – 1.90 (m, 2H);

$^{13}$**C NMR** (126 MHz, $CDCl_3$): $\delta_C$ 180.9, 137.6, 134.1, 130.6, 128.0, 126.4, 124.3, 75.6, 44.5, 43.4, 31.6, 29.8, 19.0, 16.5.

**HRMS** (ESI$^+$): *m*/*z* calc'd for **3m** $C_{14}H_{16}O_2Na$ [M+Na]$^+$: 239.10425, found: 239.10417.

**Crystal structure:** *see crystal structure*

**7-(2-fluorophenyl)-6-oxaspiro[3.4]octan-5-one (3n)**

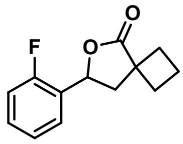

Synthesised following General Procedure A using: 2-Fluorostyrene (30 µL, 0.25mmol, 1.0 equiv) and 6,7-dioxaspiro[3.4]octane-5,8-dione **2a** (71 mg, 0.50 mmol, 2.0 equiv) in DCE. Purified by flash column chromatography ($SiO_2$; 100:0 → 95:5 pentane:EtOAc) to afford 3n (29 mg, 0.13 mmol, 53%) as a colourless liquid.

**GC-FID yield**: 55%

**TLC**: $R_f$ =0.51 (95:5 pentane:EtOAc)

**NMR Spectroscopy (*see spectra*):**

**$^1$H NMR** (599 MHz, $CDCl_3$): $\delta_H$ 7.38 (tdd, $J$ = 7.6, 1.8, 0.7 Hz, 1H), 7.33 – 7.27 (m, 1H), 7.15 (td, $J$ = 7.6, 1.2 Hz, 1H), 7.06 (ddd, $J$ = 10.6, 8.2, 1.2 Hz, 1H), 5.59 (dd, $J$ = 8.3, 6.6 Hz, 1H), 2.85 (ddd, $J$ = 13.0, 6.6, 1.2 Hz, 1H), 2.63 – 2.51 (m, 2H), 2.26 (dd, $J$ = 13.0, 8.4 Hz, 1H), 2.22 – 2.11 (m, 2H), 2.08 – 1.99 (m, 1H), 1.96 – 1.90 (m, 1H);

**$^{13}$C NMR** (151 MHz, $CDCl_3$): $\delta_C$ 180.5, 159.5 (d, $J$ = 246.5 Hz), 129.7, 126.9, 126.4 (d, $J$ = 3.8 Hz), 124.4 (d, = 3.6 Hz), 115.4, 72.8 (d, $J$ = 3.5 Hz), 44.3, 43.5 (d, $J$ = 1.5 Hz), 31.5, 29.6, 16.5;

**$^{19}$F NMR** (564 MHz, $CDCl_3$): $\delta_F$ -118.7 (m).

**HRMS** (ESI$^+$): $m/z$ calc'd for 3n $C_{13}H_{13}O_2FNa$ $[M+Na]^+$: 243.07918, found: 243.07900.

**7-(2,6-dichlorophenyl)-6-oxaspiro[3.4]octan-5-one (3o)**

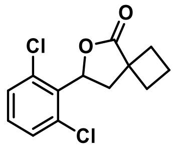

Synthesised following General Procedure A using: *o*-,*o*-dichlorostyrene (68 µL, 0.50 mmol, 1.0 equiv.) and 6,7-dioxaspiro[3.4]octane-5,8-dione **2a** (142 mg, 1.0 mmol, 2.0 equiv) in DCE. Purified by flash column chromatography ($SiO_2$; 100:0 → 90:10 pentane:EtOAc) to afford **3o** (68 mg, 0.25mmol, 50%) as a white solid.

**GC-FID yield**: 53%

**TLC**: $R_f$ =0.38 (95:5 pentane:EtOAc)

**NMR Spectroscopy (*see spectra*):**

**$^1$H NMR** (599 MHz, $CDCl_3$): $\delta_H$ 7.33 – 7.31 (m, 2H), 7.22 – 7.18 (m, 1H), 6.10 (dd, $J$ = 9.7, 7.7 Hz, 1H), 2.76 (dd, $J$ = 13.2, 7.7 Hz, 1H), 2.75 – 2.70 (m, 1H), 2.66 (dd, $J$ = 13.2, 9.7 Hz, 1H), 2.60 – 2.54 (m, 1H), 2.26 – 2.17 (m, 2H), 2.13 – 2.02 (m, 2H);

**$^{13}$C NMR** (151 MHz, $CDCl_3$): $\delta_C$ 181.0, 135.1, 133.1, 130.2, 129.7, 74.3, 44.6, 40.6, 33.6, 29.5, 16.7.

**HRMS** (ESI$^+$): *m/z* calc'd for **3o** $C_{13}H_{12}O_4Cl_2Na$ [M+Na]$^+$: 290.01066, found: 290.01054.

**Crystal structure:** *see crystal structure*

**7-(3-methoxyphenyl)-6-oxaspiro[3.4]octan-5-one (3p)**

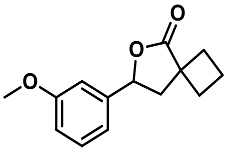

Synthesised following General Procedure A using 3-Vinylanisole (35 µL, 0.25 mmol, 1.0 equiv.) and 6,7-dioxaspiro[3.4]octane-5,8-dione **2a** (71 mg, 0.50 mmol, 2.0 equiv.) in DCE. Purified by flash column chromatography ($SiO_2$; 100:0 → 90:10 pentane:EtOAc) to afford **3p** (36 mg, 0.15 mmol, 62%) as a colourless liquid.

**GC-FID yield**: 64%

**TLC**: $R_f$ =0.48 (90:10 pentane:EtOAc)

**NMR Spectroscopy (*see spectra*):**

**$^1$H NMR** (500 MHz, $CDCl_3$): $\delta_H$ 7.31 – 7.27 (m, 1H), 6.91 – 6.85 (m, 3H), 5.34 (dd, *J* = 8.7, 6.3 Hz, 1H), 3.82 (d, *J* = 0.6 Hz, 3H), 2.78 (dd, *J* = 13.0, 6.4 Hz, 1H), 2.66 – 2.50 (m, 2H), 2.27 (dd, *J* = 12.9, 8.7 Hz, 1H), 2.22 – 2.12 (m, 2H), 2.09 – 1.93 (m, 2H);

**$^{13}$C NMR** (126 MHz, $CDCl_3$): $\delta_C$ 180.8, 160.1, 141.3, 129.9, 117.6, 114.0, 110.8, 77.9, 55.5, 44.7, 31.7, 29.5, 16.6

**HRMS** (ESI$^+$): *m/z* calc'd for **3p** $C_{14}H_{16}O_3Na$ [M+Na]$^+$: 255.09917, found: 255.09905.

**7-(3-chlorophenyl)-6-oxaspiro[3.4]octan-5-one (3q)**

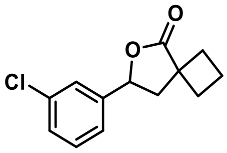

Synthesised following General Procedure A using: 1-chloro-3-vinylbenzene (32 µLc, 0.25 mmol, 1.0 equiv) and 6,7-dioxaspiro[3.4]octane-5,8-dione **2a** (85 mg, 0.6 mmol, 2.0 equiv) in DCE. Purified by flash column chromatography ($SiO_2$; 100:0 → 90:10 pentane:EtOAc) to afford **3q** (19 mg, 0.080 mmol, 32%) as a yellow liquid.

**GC-FID yield**: 34%

**Variation:** Using **2-MeO-PTH** instead of **PTH** as a photocatalyst in general procedure A, the product **3q** was isolated in a higher yield of 53% (31 mg, 0.13 mmol).

**TLC**: $R_f$ = 0.4 (90:10 pentane:EtOAc)

**NMR Spectroscopy (*see spectra*):**

**$^1$H NMR** (599 MHz, $CDCl_3$): $\delta_H$ 7.33 – 7.28 (m, 3H), 7.21 – 7.18 (m, 1H), 5.31 (dd, *J* = 8.9, 6.4 Hz, 1H), 2.78 (dd, *J* = 12.9, 6.4 Hz, 1H), 2.64 – 2.49 (m, 2H), 2.23 (dd, *J* = 13.0, 8.8 Hz, 1H), 2.20 – 2.13 (m, 2H), 2.08 – 1.98 (m, 1H), 1.98 – 1.92 (m, 1H)

**$^{13}$C NMR** (151 MHz, $CDCl_3$): $\delta_C$. 180.5, 141.7, 134.9, 130.2, 128.6, 125.6, 123.5, 77.1, 44.7, 31.7, 29.4, 16.6

**HRMS** (ESI$^+$): *m/z* calc'd for **3q** $C_{13}H_{13}O_2ClNa$ [M+Na]$^+$: 259.04963, found: 259.04961.

**7-(4-(3-bromopropyl)phenyl)-6-oxaspiro[3.4]octan-5-one (3r)**

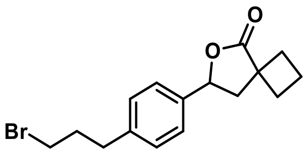


Synthesised following General Procedure A using: 1-chloro-3-vinylbenzene (32 µLc, 0.25 mmol, 1.0 equiv) and 6,7-dioxaspiro[3.4]octane-5,8-dione **2a** (85 mg, 0.6 mmol, 2.0 equiv) in DCE. Purified by flash column chromatography ($SiO_2$; 100:0 → 90:10 pentane:EtOAc) to afford **3q** (14 mg, 0.043 mmol, 17%) as a yellow liquid.

**GC-FID yield**: 21%

**TLC**: $R_f$ = 0.41 (90:10 pentane:EtOAc)

**NMR Spectroscopy (*see spectra*):**

**$^1$H NMR** (599 MHz, $CDCl_3$): $\delta_H$ 7.25 – 7.19 (m, 4H), 5.32 (dd, *J* = 9.0, 6.2 Hz, 1H), 3.38 (t, *J* = 6.6 Hz, 2H), 2.80 – 2.76 (m, 2H), 2.76 – 2.73 (m, 1H), 2.62 – 2.57 (m, 1H), 2.56 – 2.50 (m, 1H), 2.25 (dd, *J* = 12.9, 9.0 Hz, 1H), 2.20 – 2.12 (m, 4H), 2.07 – 1.99 (m, 1H), 1.99 – 1.92 (m, 1H)

**$^{13}$C NMR** (151 MHz, $CDCl_3$): $\delta_c$ 180.8, 141.0, 137.4, 129.0, 125.8, 78.0, 44.9, 44.7, 34.1, 33.7, 33.1, 31.7, 29.3, 16.6.

**HRMS** (ESI$^+$): *m/z* calc'd for **3r** $C_{16}H_{19}O_2BrNa$ [M+Na]$^+$: 345.04606, found: 345.04607.

**N-(4-(5-oxo-6-oxaspiro[3.4]octan-7-yl)phenyl)acetamide (3s)**

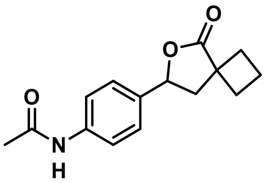


Synthesised following General Procedure A using: N-(4-vinylphenyl)acetamide **1s** (40 mg, 0.25mmol, 1.0 equiv) and 6,7-dioxaspiro[3.4]octane-5,8-dione **2a** (71 mg, 0.50 mmol, 2.0 equiv) in MeCN. Purified by flash column chromatography (SiO2; 75:25 → 25:75 pentane:EtOAc) to afford **3s** (48 mg, 0.19 mmol, 74%) as a white solid.

**GC-FID yield**: 75%

**TLC**: $R_f$ =0.12 (90:10 pentane:EtOAc)

**NMR Spectroscopy (*see spectra*):**

**$^1$H NMR** (599 MHz, $CDCl_3$): $\delta_H$ 8.21 (s, 1H), 7.55 – 7.48 (m, 2H), 7.21 – 7.14 (m, 2H), 5.29 (dd, $J$ = 8.9, 6.2 Hz, 1H), 2.73 (dd, $J$ = 13.0, 6.2 Hz, 1H), 2.59 – 2.43 (m, 2H), 2.22 (dd, $J$ = 13.0, 9.0 Hz, 1H), 2.19 – 2.08 (m, 4H), 2.07 – 1.98 (m, 1H), 1.97 – 1.91 (m, 1H);

**$^{13}$C NMR** (151 MHz, $CDCl_3$): $\delta_C$ 181.2, 169.1, 138.5, 134.6, 126.2, 120.2, 78.1, 44.9, 44.4, 31.6, 29.2, 24.5, 16.6.

**HRMS** (ESI$^+$): $m/z$ calc'd for **3s** $C_{15}H_{17}NO_3Na$ $[M+Na]^+$: 282.11006, found: 282.10996.

**tert-butyl (4-(5-oxo-6-oxaspiro[3.4]octan-7-yl)phenyl)carbamate (3t)**

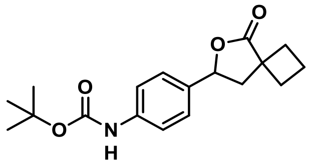

Synthesised following General Procedure A using tert-butyl (4-vinylphenyl)carbamate **1t** (55 mg, 0.25mmol, 1.0 equiv) and 6,7-dioxaspiro[3.4]octane-5,8-dione **2a** (71 mg, 0.50 mmol, 2.0 equiv) in MeCN. Purified by flash column chromatography ($SiO_2$; 98:2 → 80:20 pentane:EtOAc) to afford **3t** (35 mg, 0.11 mmol, 40%) as a white solid.

**GC-FID yield**: 42%

**TLC**: $R_f$ =0.18 (90:10 pentane:EtOAc)

**NMR Spectroscopy (*see spectra*):**

**$^1$H NMR** (599 MHz, $CDCl_3$): $\delta_H$ 7.36 (d, $J$ = 8.2 Hz, 2H), 7.24 – 7.18 (m, 2H), 6.63 (s, 1H), 5.31 – 5.25 (m, 1H), 2.71 (dd, $J$ = 13.0, 6.2 Hz, 1H), 2.60 – 2.54 (m, 1H), 2.54 – 2.45 (m, 1H), 2.22 (dd, $J$ = 13.0, 8.9 Hz, 1H), 2.19 – 2.08 (m, 2H), 2.06 – 1.90 (m, 2H), 1.50 (s, 9H);

**$^{13}$C NMR** (151 MHz, $CDCl_3$): $\delta_C$ 180.9, 152.8, 138.7, 133.7, 126.3, 118.7, 80.8, 77.9, 44.9, 44.6, 31.6, 29.3, 28.4, 16.6.

**HRMS** (ESI$^+$): $m/z$ calc'd for **3t** $C_{18}H_{13}NO_4Na$ $[M+Na]^+$: 340.15193, found: 340.15178.

**7-(naphthalen-2-yl)-6-oxaspiro[3.4]octan-5-one (3u)**

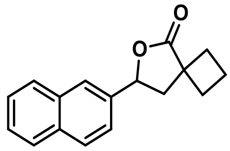

Synthesised following General Procedure A using: 2-vinylnaphthalene (39 mg, 0.25 mmol, 1.0 equiv) and 6,7-dioxaspiro[3.4]octane-5,8-dione **2a** (85 mg, 0.6 mmol, 2.0 equiv) in DCE. Purified by flash column chromatography ($SiO_2$; 100:0 → 85:15 pentane:EtOAc) to afford **3u** (29 mg, 0.11 mmol, 46%) as a slight yellow solid.

**GC-FID yield**: 49%

**TLC**: $R_f$ = 0.31 (90:10 pentane:EtOAc)

**NMR Spectroscopy (*see spectra*):**

$^{1}$**H NMR** (599 MHz, $CDCl_3$): $\delta_H$ 7.87 – 7.78 (m, 4H), 7.54 – 7.48 (m, 2H), 7.39 (ddd, *J* = 8.5, 1.8, 0.5 Hz, 1H), 5.52 (dd, *J* = 8.6, 6.3 Hz, 1H), 2.85 (dd, *J* = 13.0, 6.4 Hz, 1H), 2.66 – 2.53 (m, 2H), 2.36 (dd, *J* = 12.9, 8.7 Hz, 1H), 2.25 – 2.13 (m, 2H), 2.09 – 2.00 (m, 1H), 1.99 – 1.92 (m, 1H);

$^{13}$**C NMR** (151 MHz, $CDCl_3$): $\delta_C$. 180.9, 136.9, 133.3, 133.2, 128.8, 128.2, 127.9, 126.7, 126.5, 124.4, 123.1, 78.1, 44.8, 44.7, 31.7, 29.5, 16.7

**HRMS** (ESI$^+$): *m*/*z* calc'd for **3u** $C_{17}H_{16}O_2Na$ $[M+Na]^+$: 275.10425, found: 275.10412.

**7,7-diphenyl-6-oxaspiro[3.4]octan-5-one (3v)**

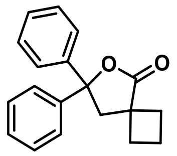


Synthesised following General Procedure A using: 1,1-diphenyl-ethene (53 µL, 0.3 mmol, 1.0 equiv.) and 6,7-dioxaspiro[3.4]octane-5,8-dione **2a** (71 mg, 0.60 mmol, 2.0 equiv) in DCE. Purified by flash column chromatography ($SiO_2$; 98:2 → 90:10 pentane:EtOAc) to afford **3v** (54 mg, 0.19 mmol, 64%) as a white solid.

**GC-FID yield**: 67%

**TLC**: $R_f$ =0.54 (90:10 pentane:EtOAc)

**NMR Spectroscopy (*see spectra*):**

$^{1}$**H NMR** (599 MHz, $CDCl_3$): $\delta_H$ 7.45 – 7.39 (m, 4H), 7.35 – 7.28 (m, 4H), 7.27 – 7.20 (m, 2H), 3.06 (s, 2H), 2.37 – 2.28 (m, 2H), 2.14 – 1.84 (m, 2H), 1.88 – 1.77 (m, 2H).

$^{13}$**C NMR** (151 MHz, $CDCl_3$): $\delta_C$ 180.5, 143.9, 128.6, 127.8, 125.4, 87.0, 49.0, 44.9, 31.0, 31.0, 16.8.

**HRMS** (ESI$^+$): *m*/*z* calc'd for **3v** $C_{19}H_{18}O_2Na$ $[M+Na]^+$: 301.11990, found: 301.11987.

**7-methyl-7-phenyl-6-oxaspiro[3.4]octan-5-one (3w)**

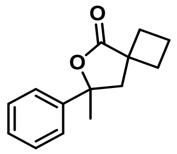


Synthesised following General Procedure A using: 1-Phenyl-1-methyl-ethene (36 µL, 0.3 mmol, 1.0 equiv.) and 6,7-dioxaspiro[3.4]octane-5,8-dione **2a** (85 mg, 0.60 mmol, 2.0 equiv) in MeCN. Purified by flash column chromatography ($SiO_2$; 98:2 → 90:10 pentane:EtOAc) to afford **3w** (38 mg, 0.17 mmol, 58%) as a white solid.

**GC-FID yield**: 62%

**TLC**: $R_f$ =0.46 (95:5 pentane:EtOAc)

**NMR Spectroscopy (*see spectra*):**

$^1$**H NMR** (500 MHz, $CDCl_3$): $\delta_H$ 7.27 (s, 4H), 7.21 – 7.16 (m, 1H), 2.59 (d, $J$ = 12.9 Hz, 1H), 2.57 – 2.49 (m, 1H), 2.46 (d, $J$ = 12.9 Hz, 1H), 2.21 – 2.14 (m, 1H), 2.08 – 1.98 (m, 2H), 1.89 – 1.80 (m, 1H), 1.64 – 1.55 (m, 4H);

$^{13}$**C NMR** (126 MHz, $CDCl_3$): $\delta_C$ 181.0, 145.2, 128.6, 127.5, 124.2, 84.3, 50.0, 45.1, 31.7, 31.4, 30.2, 16.8.

**HRMS** (ESI$^+$): $m/z$ calc'd for **3w** $C_{14}H_{16}O_2Na$ [M+Na]$^+$: 255.09917, found: 255.09905.

**7-(4-chlorophenyl)-7-methyl-6-oxaspiro[3.4]octan-5-one (3x)**

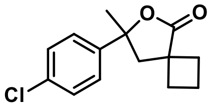

Synthesised following General Procedure A using: 1,1-diphenyl-ethene (53 µL, 0.3 mmol, 1.0 equiv.) and 6,7-dioxaspiro[3.4]octane-5,8-dione **2a** (85 mg, 0.60 mmol, 2.0 equiv) in MeCN. Purified by flash column chromatography ($SiO_2$; 98:2 → 90:10 pentane:EtOAc) to afford **3x** (34 mg, 0.14 mmol, 47%) as a yellow solid.

**GC-FID yield**: 56%

**TLC**: $R_f$ = 0.49 (90:10 pentane:EtOAc)

**NMR Spectroscopy (*see spectra*):**

$^1$**H NMR** (599 MHz, $CDCl_3$): $\delta_H$ 7.31 – 7.24 (m, 4H), 2.63 – 2.55 (m, 2H), 2.51 (dd, $J$ = 12.9, 0.9 Hz, 1H), 2.27 – 2.20 (m, 1H), 2.13 – 2.05 (m, 2H), 1.95 – 1.86 (m, 1H), 1.69 – 1.64 (m, 1H), 1.61 (s, 3H);

$^{13}$**C NMR** (151 MHz, $CDCl_3$): $\delta_C$ 180.5, 143.7, 133.3, 128.6, 125.5, 83.7, 49.7, 44.9, 31.6, 31.1, 30.0, 16.6.

**HRMS** (ESI$^+$): $m/z$ calc'd for **3x** $C_{14}H_{15}O_2ClNa$ [M+Na]$^+$: 273.06528, found: 273.06515.

**7-cyclopropyl-7-phenyl-6-oxaspiro[3.4]octan-5-one (3y)**

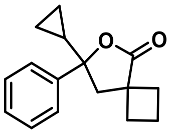

Synthesised following General Procedure A using: (1-cyclopropylvinyl)benzene **1y** (31 µL, 0.20 mmol, 1.0 equiv) and 6,7-dioxaspiro[3.4]octane-5,8-dione **2a** (56 mg, 0.40 mmol, 2.0 equiv) in MeCN. Purified by flash column chromatography ($SiO_2$; 95:5 → 80:20 pentane:EtOAc) to afford **3y** (24 mg, 0.099mmol, 49%) as a colourless solid.

**GC-FID yield**: 49%

**TLC**: $R_f$ =0.59 (90:10 pentane:EtOAc)

**NMR Spectroscopy (*see spectra*):**

$^1$**H NMR** (500 MHz, $CDCl_3$): $\delta_H$ 7.66 – 7.60 (m, 2H), 7.38 (m, 2H), 7.34 – 7.28 (m, 1H), 5.32 (d, $J$ = 1.0 Hz, 1H), 4.98 (d, $J$ = 1.0 Hz, 1H), 1.74 – 1.65 (m, 1H), 0.92 – 0.82 (m, 2H), 0.69 – 0.60 (m, 2H);

$^{13}$**C NMR** (125 MHz, CDCl$_3$): $\delta_C$. 149.52, 141.79, 128.28, 127.57, 126.26, 109.14, 15.78, 6.82.

**HRMS** (ESI$^+$): *m*/*z* calc'd for **3y** $C_{16}H_{18}O_2Na$ [M+Na]$^+$: 265.11990, found: 265.11950.

**3'',4''-dihydro-2''H,3'H,5'H-dispiro[cyclobutane-1,4'-furan-2',1''-naphthalen]-5'-one (3z)**

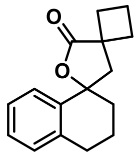


Synthesised following General Procedure A using: 1-methylene-1,2,3,4-tetrahydronaphthalene **1z** (43 mg, 0.3 mmol, 1.0 equiv.) and 6,7-dioxaspiro[3.4]octane-5,8-dione **2a** (71 mg, 0.60 mmol, 2.0 equiv) in MeCN. Purified by flash column chromatography (SiO$_2$; 98:2 → 93:7 pentane:EtOAc) to afford **3z** (41 mg, 0.17 mmol, 56%) as a colourless oil.

**GC-FID yield**: 61%

**TLC**: $R_f$ =0.36 (90:10 pentane:EtOAc)

**NMR Spectroscopy (*see spectra*):**

$^1$**H NMR** (500 MHz, CDCl$_3$): $\delta_H$ 7.25 – 7.21 (m, 1H), 7.21 – 7.17 (m, 2H), 7.11 – 7.06 (m, 1H), 2.88 – 2.75 (m, 2H), 2.75 – 2.63 (m, 2H), 2.61 (d, *J* = 13.7 Hz, 1H), 2.47 (dd, *J* = 13.7, 1.0 Hz, 1H), 2.31 – 2.14 (m, 2H), 2.09 (m, 2H), 2.05 – 1.95 (m, 2H), 1.95 – 1.80 (m, 2H);

$^{13}$**C NMR** (151 MHz, CDCl$_3$): $\delta_C$ 181.6, 138.8, 137.1, 129.0, 128.0, 126.6, 126.2, 83.6, 50.6, 45.1, 36.3, 34.3, 31.3, 29.0, 20.1, 16.8.

**HRMS** (ESI$^+$): *m*/*z* calc'd for **3z** $C_{16}H_{18}O_2Na$ [M+Na]$^+$: 265.11990, found: 265.11966.

**10'',11''-dihydro-3'H,5'H-dispiro[cyclobutane-1,4'-furan-2',5''-dibenzo[a,d][7]annulen]-5'-one (3aa)**

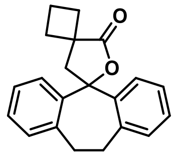


Synthesised following General Procedure A using: 5-methylene-10,11-dihydro-5H-dibenzo[a,d][7]annulene **1aa** (52 mg, 0.25 mmol, 1.0 equiv.) and 6,7-dioxaspiro[3.4]octane-5,8-dione **2a** (71 mg, 0.50 mmol, 2.0 equiv) in MeCN. Purified by flash column chromatography (SiO$_2$; 100:0→ 80:20 pentane:EtOAc) to afford **3aa** (28 mg, 0.091 mmol, 36%) as a white solid.

**GC-FID yield**: 40%

**TLC**: $R_f$ = 0.47 (95:5 pentane:EtOAc)

**NMR Spectroscopy (*see spectra*):**

$^1$**H NMR** (599 MHz, CDCl$_3$): $\delta_H$ 7.63 – 7.54 (m, 2H), 7.21 – 7.13 (m, 6H), 3.60 – 3.49 (m, 2H), 3.08 (s, 2H), 3.05 – 2.97 (m, 2H), 2.50 – 2.41 (m, 2H), 2.13 – 2.01 (m, 1H), 1.95 – 1.82 (m, 1H), 1.76 – 1.67 (m, 2H);

> **$^{13}$C NMR** (151 MHz, $CDCl_3$): $\delta_C$ 180.8, 142.0, 136.8, 131.0, 128.0, 126.5, 124.2, 86.2, 53.3, 44.4, 32.9, 31.8, 16.8.

**HRMS** (ESI$^+$): *m*/*z* calc'd for **3aa** $C_{21}H_{20}O_2Na$ [M+Na]$^+$: 327.13555, found: 327.13559.

**8-methyl-7-phenyl-6-oxaspiro[3.4]octan-5-one (3ab)**

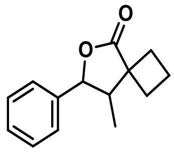


Synthesised following General Procedure A using: (E)-prop-1-en-1-ylbenzene (32 µL, 0.25 mmol, 1.0 equiv.) and 6,7-dioxaspiro[3.4]octane-5,8-dione **2a** (71 mg, 0.50 mmol, 2.0 equiv) in MeCN. Purified by flash column chromatography ($SiO_2$; 100:0→ 80:20 pentane:EtOAc) to afford **3ab** as a 10:1 mixture of diastereomers (21 mg, 0.099 mmol, 34%) as a colourless oil.

**GC-FID yield**: 37%

**TLC**: $R_f$ = 0.31 (90:10 pentane:EtOAc)

**NMR Spectroscopy (*see spectra*):**

> **$^1$H NMR** (599 MHz, $CDCl_3$): $\delta_H$ 7.40 – 7.32 (m, 2.82H), 7.31 – 7.28 (m, 1.78H), 7.23 – 7.21 (m, 0.31H, $d_{min}$), 5.48 (d, *J* = 5.6 Hz, 0.15H, $d_{min}$), 4.75 (d, *J* = 9.2 Hz, 0.85H, $d_{maj}$), 2.63 (qd, *J* = 7.2, 5.6 Hz, 0.16H, $d_{min}$), 2.57 – 2.46 (m, 1H), 2.44 – 2.33 (m, 1H), 2.30 – 2.13 (m, 2.96H), 2.11 – 2.05 (m, 0.18H), 2.00 – 1.86 (m, 1.88H), 1.17 (d, *J* = 6.9 Hz, 2.5H, $d_{maj}$), 0.60 (d, *J* = 7.2 Hz, 0.45H, $d_{min}$);

> **$^{13}$C NMR** (151 MHz, $CDCl_3$): $\delta_C$ 181.1, 180.9, 138.0, 136.7, 128.7, 128.7, 128.5, 127.9, 126.1, 125.5, 84.8, 80.7, 49.8, 48.4, 48.0, 45.3, 31.5, 27.5, 24.8, 24.7, 16.5, 16.3, 11.2, 10.3.

**HRMS** (ESI$^+$): *m*/*z* calc'd for **3ab** $C_{14}H_{16}O_2Na$ [M+Na]$^+$: 239.10535, found: 239.10417.

**3a',4',5',9b'-tetrahydro-2'H-spiro[cyclobutane-1,3'-naphtho[1,2-b]furan]-2'-one (3ac)**

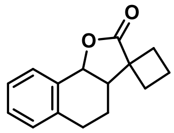


Synthesised following General Procedure A using: 1,2-dihydronaphthalene (33 µL, 0.25 mmol, 1.0 equiv.) and 6,7-dioxaspiro[3.4]octane-5,8-dione **2a** (71 mg, 0.50 mmol, 2.0 equiv) in MeCN. Purified by flash column chromatography ($SiO_2$; 100:0→ 80:20 pentane:EtOAc) to afford **3ac** (11 mg, 0.048 mmol, 19%) as a colourless liquid.

**GC-FID yield**: 20%

**TLC**: No $R_f$ value was determined as the product did not stain. Isolation was performed by identification of the product by GC-MS.

**NMR Spectroscopy (*see spectra*):**

$^{1}$**H NMR** (599 MHz, $CDCl_3$): $\delta_H$ 7.44 – 7.42 (m, 1H), 7.28 – 7.24 (m, 2H), 7.21 – 7.12 (m, 1H), 5.28 (d, $J$ = 5.0 Hz, 1H), 2.76 – 2.66 (m, 1H), 2.54 (m, 1H), 2.50 – 2.41 (m, 1H), 2.34 – 2.23 (m, 3H), 2.22 – 2.13 (m, 2H), 2.05 – 1.85 (m, 3H);

$^{13}$**C NMR** (151 MHz, $CDCl_3$): $\delta_C$ 180.2, 137.9, 131.4, 131.1, 129.1, 128.9, 126.8, 75.2, 49.9, 45.4, 32.4, 28.3, 23.5, 20.9, 16.6.

**HRMS** ($ESI^+$): *m*/*z* calc'd for **3ac** $C_{15}H_{16}O_2Na$ $[M+Na]^+$: 251.10425, found: 251.10424.

**3'H,5'H-dispiro[chromane-4,2'-furan-4',1''-cyclobutan]-5'-one (3ad)**

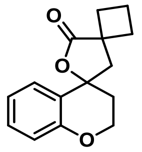

Synthesised following General Procedure A using: 4-methylenechromane (37 mg, 0.25mmol, 1.0 equiv) and 6,7-dioxaspiro[3.4]octane-5,8-dione **2a** (71 mg, 0.50 mmol, 2.0 equiv) in MeCN. Purified by flash column chromatography ($SiO_2$; 100:0 → 85:15 pentane:EtOAc) to afford **3ad** (38 mg, 0.13 mmol, 66%) as a white solid.

**GC-FID yield**: 69%

**TLC**: $R_f$ =0.31 (90:10 pentane:EtOAc)

**NMR Spectroscopy (*see spectra*):**

$^{1}$**H NMR** (500 MHz, $CDCl_3$): $\delta_H$ 7.24 – 7.15 (m, 2H), 6.92 (ddd, $J$ = 7.8, 7.2, 1.3 Hz, 1H), 6.83 (dt, $J$ = 7.7, 1.2 Hz, 1H), 4.31 (ddd, $J$ = 11.2, 7.8, 3.3 Hz, 1H), 4.23 (ddd, $J$ = 11.3, 7.9, 3.2 Hz, 1H), 2.77 – 2.67 (m, 2H), 2.66 – 2.60 (m, 1H), 2.52 (d, $J$ = 13.8 Hz, 1H), 2.32 – 2.14 (m, 4H), 2.09 – 1.98 (m, 2H);

$^{13}$**C NMR** (126 MHz, $CDCl_3$): $\delta_C$. 180.7, 154.9, 130.2, 126.9, 123.7, 121.1, 117.4, 78.6, 63.1, 49.4, 45.0, 35.9, 34.0, 31.1, 16.7.

**HRMS** ($ESI^+$): *m*/*z* calc'd for **3ad** $C_{15}H_{16}O_3Na$ $[M+Na]^+$: 267.09917, found: 267.09912.

**7-phenyl-7-(thiophen-2-yl)-6-oxaspiro[3.4]octan-5-one (3ae)**

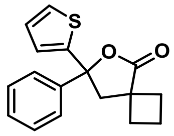

Synthesised following General Procedure A using: 2-(1-phenylvinyl)thiophene **1ae** (47 mg, 0.25 mmol, 1.0 equiv.) and 6,7-dioxaspiro[3.4]octane-5,8-dione **2a** (71 mg, 0.50 mmol, 2.0 equiv) in MeCN. Purified by flash column chromatography ($SiO_2$; 98:2 → 90:10 pentane:EtOAc) to afford **3ae** (35 mg, 0.12 mmol, 49%) as a opaque solid.

**GC-FID yield**: 50%

**TLC**: $R_f$ = 0.43 (95:5 pentane:EtOAc)

**NMR Spectroscopy (*see spectra*):**

$^{1}$**H NMR** (599 MHz, $CDCl_3$): $\delta_H$ 7.40 – 7.37 (m, 2H), 7.28 (ddt, $J$ = 7.8, 6.3, 0.8 Hz, 2H), 7.24 – 7.21 (m, 1H), 7.20 – 7.18 (m, 1H), 6.88 (ddd, $J$ = 3.6, 1.3, 0.7 Hz, 1H), 6.85 (ddd, $J$ = 5.0, 3.6, 0.7 Hz, 1H), 3.08 (d, $J$ = 13.0 Hz, 1H), 2.94 (d, $J$ = 13.1 Hz, 1H), 2.42 – 2.34 (m, 1H), 2.27 – 2.19 (m, 1H), 2.09 – 2.00 (m, 1H), 1.97 – 1.91 (m, 1H), 1.90 – 1.83 (m, 1H), 1.75 – 1.67 (m, 1H);

$^{13}$**C NMR** (151 MHz, $CDCl_3$): $\delta_C$ 180.1, 147.8, 143.5, 128.6, 128.1, 126.8, 126.2, 125.4, 125.1, 85.0, 50.7, 45.1, 31.3, 31.1, 16.8.

**HRMS** (ESI$^+$): *m*/*z* calc'd for **3ae** $C_{17}H_{16}O_2SNa$ $[M+Na]^+$: 307.07632, found: 307.07630.

**3'H,5'H-dispiro[cyclobutane-1,4'-furan-2',4''-thiochroman]-5'-one 1'',1''-dioxide (3af)**

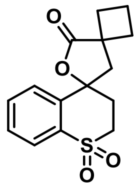

Synthesised following General Procedure A using: 4-methylenethiochromane (41 mg, 0.25mmol, 1.0 equiv) and 6,7-dioxaspiro[3.4]octane-5,8-dione **2a** (142 mg, 1.0 mmol, 4.0 equiv) in MeCN. Purified by flash column chromatography ($SiO_2$; 75:20:5 → 60:25:15 pentane:$CH_2Cl_2$:EtOAc) to afford **3af** (38 mg, 0.13 mmol, 45%) as a colourless solid.

**GC-FID yield**: 45%

**TLC**: No $R_f$ value was determined as the product did not stain. Isolation was performed by identification of the product by GC-MS.

**NMR Spectroscopy (*see spectra*):**

$^{1}$**H NMR** (599 MHz, $CDCl_3$): $\delta_H$ 7.93 (ddd, $J$ = 7.8, 1.5, 0.5 Hz, 1H), 7.60 (ddd, $J$ = 8.0, 7.4, 1.5 Hz, 1H), 7.58 – 7.53 (m, 1H), 7.37 – 7.31 (m, 1H), 3.61 (ddd, $J$ = 14.1, 8.6, 3.2 Hz, 1H), 3.47 (ddd, $J$ = 14.2, 10.3, 3.1 Hz, 1H), 2.83 (dddd, $J$ = 14.2, 10.3, 3.2, 0.6 Hz, 1H), 2.74 – 2.65 (m, 4H), 2.62 (ddd, $J$ = 14.8, 8.6, 3.1 Hz, 1H), 2.34 – 2.26 (m, 1H), 2.25 – 2.19 (m, 1H), 2.17 – 2.11 (m, 1H), 2.10 – 1.98 (m, 1H).

$^{13}$**C NMR** (151 MHz, $CDCl_3$): $\delta_C$ 180.0, 138.1, 138.0, 133.4, 130.2, 126.9, 124.0, 79.6, 49.2, 47.7, 44.4, 34.3, 33.8, 31.6, 16.7.

**HRMS** (ESI$^+$): *m*/*z* calc'd for **3af** $C_{15}H_{16}O_4SNa$ $[M+Na]^+$: 315.06615, found: 315.06624.

**3'H,5'H-dispiro[cyclobutane-1,4'-furan-2',11''-dibenzo[b,e]azepine]-5',6''(5''H)-dione (3ag)**

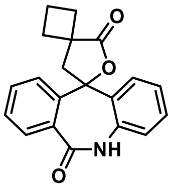

Synthesised following General Procedure A using: 11-methylene-5,11-dihydro-6H-dibenzo[b,e]azepin-6-one 1**ag** (66 mg, 0.3 mmol, 1.0 equiv.) and 6,7-dioxaspiro[3.4]octane-5,8-dione **2a** (85 mg, 0.60 mmol, 2.0 equiv) in MeCN. Purified by flash column chromatography ($SiO_2$; 90:10→ 50:50 pentane:EtOAc) to afford **3ag** (35 mg, 0.11 mmol, 37%) as a white solid.

**GC-FID yield**: 40%

**TLC**: $R_f$ = 0.62 (60:40 pentane:EtOAc)

**NMR Spectroscopy (*see spectra*):**

$^1$**H NMR** (599 MHz, $CDCl_3$): $\delta_H$ 9.99 (s, 1H), 8.04 (dd, $J$ = 7.8, 1.4 Hz, 1H), 7.67 (td, $J$ = 7.8, 1.3 Hz, 2H), 7.53 (ddd, $J$ = 8.0, 7.3, 1.5 Hz, 1H), 7.41 (td, $J$ = 7.5, 1.2 Hz, 1H), 7.30 (ddd, $J$ = 7.8, 7.2, 1.5 Hz, 1H), 7.25 (dd, $J$ = 7.9, 1.3 Hz, 1H), 7.20 (ddd, $J$ = 8.0, 7.2, 1.4 Hz, 1H), 2.98 (d, $J$ = 13.1 Hz, 1H), 2.91 (d, $J$ = 13.2 Hz, 1H), 2.43 – 2.22 (m, 2H), 2.09 – 1.98 (m, 1H), 1.86 (dtt, $J$ = 11.6, 9.4, 6.9 Hz, 1H), 1.74 – 1.62 (m, 2H);

$^{13}$**C NMR** (151 MHz, $CDCl_3$): $\delta_C$ 179.7, 169.8, 169.7, 144.5, 135.0, 133.1, 132.9, 131.6, 128.9, 128.4, 128.0, 125.7, 123.6, 121.7, 121.7, 83.3, 47.5, 44.0, 31.4, 30.8, 16.7.

**HRMS** (ESI$^+$): $m/z$ calc'd for **3ag** $C_{20}H_{17}O_3NNa$ $[M+Na]^+$: 342.11006, found: 342.11016.

**3-phenyl-2-oxaspiro[4.4]nonan-1-one (4b)**

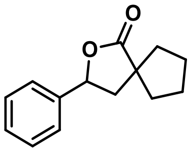

Synthesised following General Procedure A using styrene (57 µL, 0.5 mmol, 1.0 equiv.) and 2,3-dioxaspiro[4.5]decane-1,4-dione **2b** (312 mg, 2.0 mmol, 4.0 equiv.) in MeCN. Purified by flash column chromatography ($SiO_2$; 95:5 pentane:EtOAc) to afford **4b** (66 mg, 0.31 mmol, 61%) as a colourless solid.

**GC-FID yield:** 65%

**TLC**: $R_f$ = 0.40 (95:5 pentane:EtOAc)

**NMR Spectroscopy (*see spectra*):**

$^1$**H NMR** (599 MHz, $CDCl_3$): $\delta_H$ .7.42 – 7.30 (m, 5H), 5.41 (dd, $J$ = 9.6, 6.2 Hz, 1H), 2.53 (dd, $J$ = 12.7, 6.2 Hz, 1H), 2.24 – 2.16 (m, 1H), 2.10 (dd, $J$ = 12.7, 9.6 Hz, 1H), 2.04 – 1.98 (m, 1H), 1.93 – 1.82 (m, 3H), 1.78 – 1.62 (m, 2H), 1.61 – 1.55 (m, 1H);

$^{13}$**C NMR** (151 MHz, $CDCl_3$): $\delta_C$ 182.3, 139.7, 128.8, 128.4, 125.5, 78.4, 50.6, 46.0, 37.4, 36.9, 25.7, 25.6.

**HRMS** (ESI$^+$): $m/z$ calc'd for **4b** $C_{14}H_{16}O_2Na$ $[M+Na]^+$: 239.10425, found: 239.10416.

**3-phenyl-2-oxaspiro[4.5]decan-1-one (4c)**

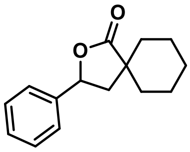

Synthesised following General Procedure A using styrene (57 µL, 0.5 mmol, 1.0 equiv.) and 2,3-dioxaspiro[4.5]decane-1,4-dione **2c** (170 mg, 1.0 mmol, 2.0 equiv.) in MeCN. Purified by flash column chromatography ($SiO_2$; 95:5 → 90:10 pentane:EtOAc) to afford **4c** (52 mg, 0.23 mmol, 45%) as a colourless solid.

**GC-FID yield:** 49%

**TLC**: $R_f$ = 0.24 (95:5 pentane:EtOAc)

**NMR Spectroscopy (*see spectra*):**

$^1$**H NMR** (599 MHz, $CDCl_3$): $\delta_H$ 7.41 – 7.30 (m, 5H), 5.43 (dd, *J* = 9.7, 6.6 Hz, 1H), 2.69 (dd, *J* = 13.1, 6.6 Hz, 1H), 1.97 (ddd, *J* = 13.1, 9.7, 1.1 Hz, 1H), 1.87 (ddd, *J* = 13.4, 12.2, 3.9 Hz, 1H), 1.82 – 1.61 (m, 5H), 1.56 – 1.50 (m, 1H), 1.49 – 1.33 (m, 2H), 1.29 – 1.19 (m, 1H);

$^{13}$**C NMR** (151 MHz, $CDCl_3$): $\delta_C$ 181.4, 140.0, 128.9, 128.4, 125.4, 78.1, 45.4, 42.4, 34.3, 31.7, 25.4, 22.4, 22.3.

**HRMS** ($ESI^+$): *m*/*z* calc'd for **4c** $C_{15}H_{18}O_2Na$ $[M+Na]^+$: 253.11990, found: 253.11976.

**3-phenyl-2,8-dioxaspiro[4.5]decan-1-one (4d)**

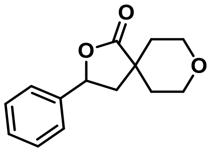

Synthesised following General Procedure A using styrene (28.5 µL, 0.25 mmol, 1.0 equiv.) and 2,3,8-trioxaspiro[4.5]decane-1,4-dione **2c** (86 mg, 0.50 mmol, 2.0 equiv.) in MeCN. Purified by flash column chromatography ($SiO_2$; 100:0 → 75:25 pentane:EtOAc) to afford **4d** (12.7 mg, 0.054 mmol, 22%) as white solid.

**GC-FID yield:** 24%

**TLC**: $R_f$ = 0.19 (95:5 pentane:EtOAc)

**NMR Spectroscopy (*see spectra*):**

$^1$**H NMR** (500MHz, $CDCl_3$): $\delta_H$ 7.42 – 7.38 (m, 2H), 7.36 – 7.32 (m, 3H), 5.49 (dd, *J* = 9.5, 6.5 Hz, 1H), 4.05 (dd, *J* = 11.8, 5.1 Hz, 1H), 3.99 (ddd, *J* = 12.1, 5.1, 4.0 Hz, 1H), 3.66 (ddd, *J* = 12.1, 9.4, 3.1 Hz, 1H), 3.50 (ddd, *J* = 11.8, 9.4, 3.1 Hz, 1H), 2.74 (dd, *J* = 13.1, 6.5 Hz, 1H), 2.17 – 2.00 (m, 3H), 1.74 – 1.67 (m, 1H), 1.53 – 1.47 (m, 1H);

$^{13}$**C NMR** (125 MHz, $CDCl_3$): $\delta_C$ 179.7, 139.5, 129.0, 128.6, 125.4, 77.9, 64.2, 63.9, 42.9, 42.6, 33.8, 32.1.

**HRMS** ($ESI^+$): *m*/*z* calc'd for **4d** $C_{14}H_{16}O_3Na$ $[M+Na]^+$: 255.09917, found: 255.09899.

**9-phenyl-8-oxadispiro[3.1.46.14]undecan-7-one (4e)**

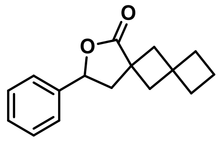

Synthesised following General Procedure A using styrene (29 µL, 0.25 mmol, 1.0 equiv.) and 8,9-dioxadispiro[3.1.46.14]undecane-7,10-dione **2e** (91 mg, 0.50 mmol, 2.0 equiv.) in DCE. Purified by flash column chromatography (SiO2; 95:5 → 85:15 pentane:EtOAc) to afford 4g (46.7 mg, 0.19 mmol, 49%) as a colourless solid.

**GC-FID yield:** 80%

**TLC**: $R_f$ = 0.22 (95:5 pentane:EtOAc)

**NMR Spectroscopy (*see spectra*):**

$^1$**H NMR** (599 MHz, $CDCl_3$): $\delta_H$ 7.39 – 7.34 (m, 2H), 7.33 – 7.28 (m, 3H), 5.33 (dd, *J* = 8.8, 6.3 Hz, 1H), 2.71 (dd, *J* = 12.9, 6.3 Hz, 1H), 2.62 (dd, *J* = 11.8, 1.2 Hz, 1H), 2.50 (dd, *J* = 11.6, 1.2 Hz, 1H), 2.24 (dd, *J* = 12.9, 8.8 Hz, 1H), 2.16 (ddd, *J* = 11.5, 3.5, 1.0 Hz, 1H), 2.11 – 2.00 (m, 4H), 1.92 (ddd, *J* = 11.9, 3.5, 1.0 Hz, 1H), 1.81 (quin, *J* = 7.6 Hz, 2H);

$^{13}$**C NMR** (151 MHz, $CDCl_3$): $\delta_C$ 180.8, 139.5, 128.7, 128.4, 125.4, 78.0, 44.9, 44.0, 42.1, 40.3, 38.9, 36.0, 34.6, 16.2.

**HRMS** (ESI$^+$): *m*/*z* calc'd for **4e** $C_{16}H_{18}O_2Na$ [M+Na]$^+$: 265.11990, found: 265.11986.

**6-phenyl-5-oxaspiro[2.4]heptan-4-one (4f)**

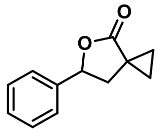

Synthesised following General Procedure A using styrene (28.5 µL, 0.25 mmol, 1.0 equiv.) and 5,6-dioxaspiro[2.4]heptane-4,7-dione **2f** (64 mg, 0.50 mmol, 2.0 equiv.) in MeCN. Purified by flash column chromatography ($SiO_2$; 50:50 → 0:100 pentane:$CH_2Cl_2$) to afford **4f** (11 mg, 0.058 mmol, 24%) as a colourless solid.

**GC-FID yield:** 25%

**TLC**: $R_f$ =0.5 (0:100 pentane:$CH_2Cl_2$)

**NMR Spectroscopy (*see spectra*):**

$^1$**H NMR** (599 MHz, $CDCl_3$): $\delta_H$ 7.41 – 7.32 (m, 5H), 5.61 (dd, *J* = 7.7 Hz, 7.8 Hz, 1H), 2.62 (dd, *J* = 12.8, 7.8 Hz, 1H), 2.38 (dd, *J* = 12.8, 7.7 Hz, 1H), 1.41 – 1.36 (m, 1H), 1.33 – 1.29 (m, 1H), 1.09 – 1.05 (m, 1H), 0.98 – 0.94 (m, 1H);

$^{13}$**C NMR** (151 MHz, $CDCl_3$): $\delta_C$. 179.8, 140.2, 128.9, 128.5, 125.6, 78.8, 38.5, 20.7, 16.1, 14.5.

**HRMS** (ESI$^+$): *m*/*z* calc'd for **4f** $C_{12}H_{12}O_2Na$ [M+Na]$^+$ 211.07295:, found: 211.07288.

**5-phenyl-3,3-dipropyldihydrofuran-2(3H)-one (4g)**

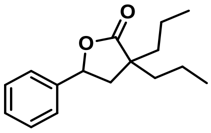

Synthesised following General Procedure A using styrene (57 µL, 0.5 mmol, 1.0 equiv.) and 4,4-dipropyl-1,2-dioxolane-3,5-dione **2g** (466 mg, 2.5 mmol, 5.0 equiv.) in MeCN. Purified by flash column chromatography ($SiO_2$; 100:0 → 95:5 pentane:EtOAc) to afford **4g** (74.8 mg, 0.30 mmol, 61%) as a colourless solid.

**GC-FID yield:** 65%

**TLC**: $R_f$ =0.53 (95:5 pentane:EtOAc)

**NMR Spectroscopy (*see spectra*):**

> **$^1$H NMR** (599 MHz, $CDCl_3$): $\delta_H$ 7.40 – 7.36 (m, 2H), 7.34 – 7.30 (m, 3H), 5.40 (dd, $J$ = 9.7, 7.1 Hz, 1H), 2.46 (dd, $J$ = 13.2, 7.1 Hz, 1H), 2.13 (dd, $J$ = 13.2, 9.7 Hz, 1H), 1.68 – 1.56 (m, 4H), 1.56 – 1.47 (m, 1H), 1.46 – 1.35 (m, 2H), 1.28 – 1.19 (m, 1H), 0.97 (t, $J$ = 7.2 Hz, 3H), 0.92 (t, $J$ = 7.3 Hz, 3H);

> **$^{13}$C NMR** (151 MHz, $CDCl_3$): $\delta_C$ 180.9, 140.5, 128.9, 128.4, 125.4, 78.0, 48.5, 41.7, 39.1, 38.4, 17.9, 17.9, 14.6, 14.6.

**HRMS** (ESI$^+$): $m/z$ calc'd for **4g** $C_{16}H_{22}O_2Na$ $[M+Na]^+$: 269.15120, found: 269.15103.

**3-methyl-5-phenyl-3-propyldihydrofuran-2(3H)-one (4h)**

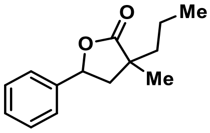


Synthesised following General Procedure A using styrene (29 µL, 0.25 mmol, 1.0 equiv.) and 4-methyl-4-propyl-1,2-dioxolane-3,5-dione **2h** (147 mg, 1.25 mmol, 5.0 equiv.) in MeCN. Purified by flash column chromatography ($SiO_2$; 100:0 → 95:5 pentane:EtOAc) to afford **4h** (1:1 *d.r.*; diastereomers separated on column) (36.1 mg, 0.16 mmol, 66%) as a colourless liquid.

**GC-FID yield:** 71% (combined diastereomers)

**TLC**: $R_f$ =0.45 (95:5 pentane:EtOAc)

**NMR Spectroscopy (*see spectra*) (*trans* 3-methyl-5-phenyl-3-propyldihydrofuran-2(3H)-one):**

> **$^1$H NMR** (599 MHz, $CDCl_3$): $\delta_H$ 7.41 – 7.37 (m, 2H), 7.35 – 7.32 (m, 3H), 5.44 (dd, $J$ = 10.2, 6.4 Hz, 1H), 2.34 (dd, $J$ = 12.9, 6.4 Hz, 1H), 2.14 (ddd, $J$ = 12.9, 10.3, 0.6 Hz, 1H), 1.66 – 1.56 (m, 2H), 1.50 – 1.40 (m, 1H), 1.35 (s, 3H), 1.29 – 1.17 (m, 1H), 0.93 (t, $J$ = 7.3 Hz, 3H);

> **$^{13}$C NMR** (151 MHz, $CDCl_3$): $\delta_C$. 181.5, 139.8, 128.9, 128.5, 125.5, 77.9, 44.7, 43.4, 39.9, 22.7, 17.9, 14.5.

**NMR Spectroscopy (*see spectra*) (*cis* 3-methyl-5-phenyl-3-propyldihydrofuran-2(3H)-one):**

> **$^1$H NMR** (599 MHz, $CDCl_3$): $\delta_H$ 7.42 – 7.35 (m, 2H), 7.35 – 7.30 (m, 3H), 5.43 (dd, *J* = 9.4, 6.8 Hz, 1H), 2.61 (dd, *J* = 13.1, 6.9 Hz, 1H), 2.02 (dd, *J* = 13.1, 9.4 Hz, 1H), 1.70 – 1.57 (m, 2H), 1.56 – 1.47 (m, 1H), 1.45 – 1.36 (m, 1H), 1.27 (s, 3H), 0.98 (t, *J* = 7.2 Hz, 3H);

> **$^{13}$C NMR** (151 MHz, $CDCl_3$): $\delta_C$. 181.5, 140.2, 128.9, 128.4, 125.4, 77.8, 44.4, 44.3, 40.0, 23.6, 18.0, 14.6.

**HRMS** (ESI$^+$): *m*/*z* calc'd for **4h** $C_{16}H_{22}O_2Na$ [M+Na]$^+$: 241.11990, found: 241.11986.

**5-phenyl-1',3',4,5-tetrahydro-2H-spiro[furan-3,2'-inden]-2-one (4i)**

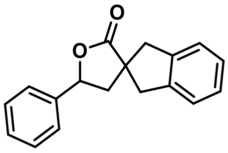


Synthesised following General Procedure A using styrene (29 µL, 0.25 mmol, 1.0 equiv.) and 1,3-dihydrospiro[indene-2,4'-[1,2]dioxolane]-3',5'-dione **2j** (102 mg, 0.50 mmol, 2.0 equiv.) in DCE. Purified by flash column chromatography (SiO2; 95:5 → 80:20 pentane:EtOAc) to afford 4i (45 mg, 0.17 mmol, 68%) as a colourless solid.

**GC-FID yield:** 70%

**TLC**: $R_f$ =0.22 (95:5 pentane:EtOAc)

**NMR Spectroscopy (*see spectra*):**

> **$^1$H NMR** (599 MHz, $CDCl_3$): $\delta_H$ 7.43 – 7.32 (m, 5H), 7.25 – 7.18 (m, 4H), 5.54 (dd, *J* = 9.5, 6.1 Hz, 1H), 3.67 (d, *J* = 16.0 Hz, 1H), 3.45 (d, *J* = 15.4 Hz, 1H), 3.19 (d, *J* = 15.6 Hz, 1H), 2.89 (d, *J* = 16.0 Hz, 1H), 2.70 (dd, *J* = 13.0, 6.1 Hz, 1H), 2.22 (dd, *J* = 12.9, 9.5 Hz, 1H);

> **$^{13}$C NMR** (151 MHz, $CDCl_3$): $\delta_C$ 180.8, 141.0, 139.6, 139.2, 128.9, 128.6, 127.3, 127.1, 125.5, 124.7, 124.6, 78.4, 51.3, 46.1, 43.5, 43.5.

**HRMS** (ESI$^+$): *m*/*z* calc'd for **4i** $C_{18}H_{16}O_2Na$ [M+Na]$^+$: 287.10425, found: 287.10418.

## 2.6. Substrate Limitations

**Table S6**: Substrate limitations of the photocatalytic formation of γ-lactones.

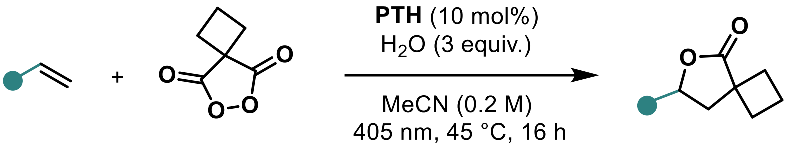


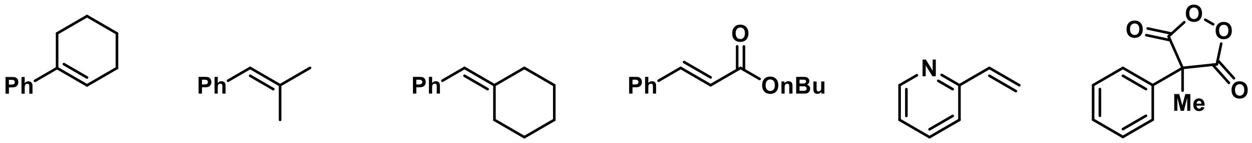


| A1 | A2 | A3 | A4 | A5 | A6 |
|---|---|---|---|---|---|
| 21% | 14%, 22%rsm | 17% | 20%, 19% rsm | 0% | 0% |

Reaction conditions: **2a/A6** (0.5 mmol), the respective alkene (0.25 mmol), **PTH** (10 mol%), MeCN (0.2 M), $H_2O$ (0.75 mmol), blue LEDs ($\lambda_{max}$ = 405 nm), 45 °C, 16 h. Yields given were determined by GC-FID analysis of the crude reaction using mesitylene as an internal standard.

A1-A4 were found to be too sterically hindered for the reaction. It was also found that the reaction does not form the product when easily oxidizable functional groups are present (A5). Peroxide A6 likely does not show the resulting reactivity because after decarboxylation, an even more stable benzylic radical is formed, so there is no driving force to attack the styrene and ORPC takes place at the substrate itself.

## 2.7. Sensitivity Screen

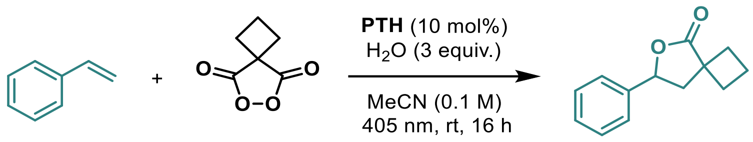


**1a** **2a** **3a**

Performed using a modified literature procedure.[15] All experiments were performed on a 0.2 mmol scale. Solid **2a** (2.0 eq, 57 mg) was dispensed into the reaction tubes. A stock solution (12x amount) of styrene **1a** (2.40 mmol, 250 mg, 275 µL), $H_2O$ (3 eq, 7.20 mmol, 130 mg, 130 µL) and **PTH** (10 mol%, 0.24 mmol, 66 mg) in 3.0 mL degassed MeCN was prepared. Before $H_2O$ was added to the stock solution, 270 µL were dispensed in reaction #6. Then, all experimental modifications were carried out as given in General Procedure A**Fehler! Verweisquelle konnte nicht gefunden werden.**, before aliquots of 280 µL of the stock solution were dispensed into the reaction tubes. All reaction mixtures except for #2 and #3 were degassed by 3 *freeze-pump-thaw* cycles.

Subsequent irradiation for 22 h and analysis by GC-FID resulted in the given reaction yields, and relative deviations from the reference reaction #1 were calculated.

**Table S7**: Sensitivity assessment of the photocatalytic formation of γ-lactones.

| Entry | Modification | Deviation from standard conditions | Yield[a] | Deviation from benchmark |
|---|---|---|---|---|
| 1 | low $H_2O$ | took aliquote of stock solution before $H_2O$ was added | 63% | -9% |
| 2 | high $O_2$ | reactions set up and carried out on air | 1% | -71% |
| 3 | medium $O_2$ | solvent sparged with air, reaction then carried out under argon atmosphere | 75% | +3% |
| 4 | low concentration | 270 µL MeCN | 67% | -5% |
| 5 | high concentration | 4 mL MeCN | 40% | -31% |
| 6 | high intensity | two 18 W LEDs, directly attached (d = 2 cm) but cooled with fan | 68% | -3% |
| 7 | low intensity | one 18 W LED, d = 30 cm (**Figure S8A**) | 56% | -15% |
| 8 | big scale | according to general procedure C on 5 mmol scale, irradiated with two 18 W LEDs and cooled with fan (**Figure S8B**) | 71%[b] | 0% |
| 9 | low temperature | carried out in low temperature photoreactor at 0 °C (**Fehler! Verweisquelle konnte nicht gefunden werden.**) | 47% | -24% |
| 10 | high temperature | carried out in photoreactor without fan | 74% | 3% |

[a] Yields were determined by GC-FID analysis using mesitylene as an internal standard. [b] Isolated yield.

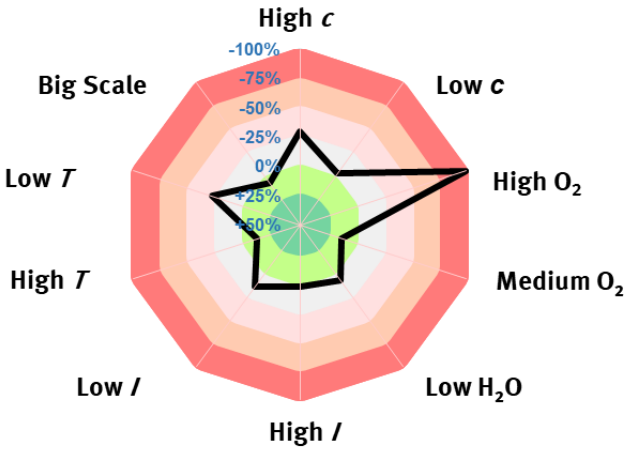


**Figure S6**: Radar diagram representation of sensitivity screen.

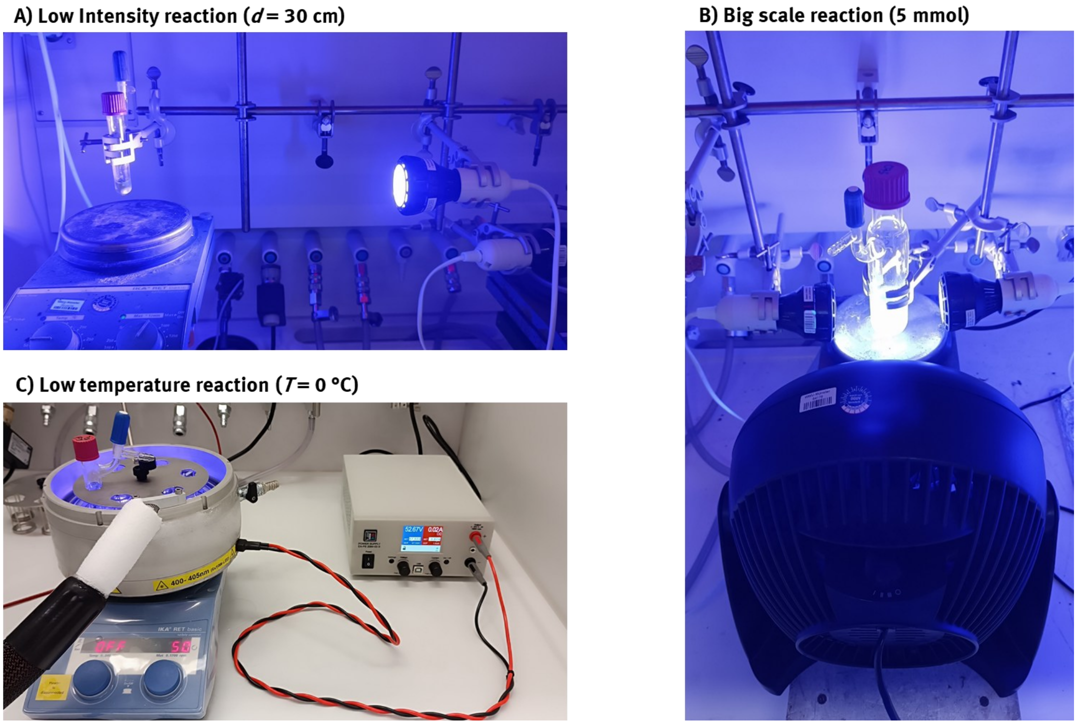


**Figure S7:** Experimental set-ups for A) low intensity reactions. B) high intensity and big scale reactions and C) low temperature reactions in a photoreactor cooled with an external cryostat and cooling liquid.

## 2.8. Additive-based Robustness Screen

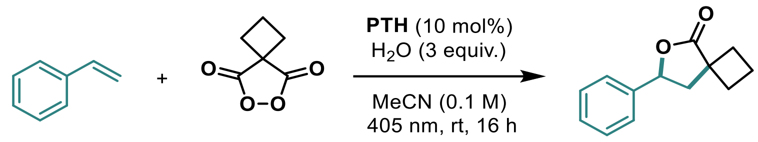


The tolerance of the reaction towards different functional groups was determined by means of a robustness screening according to a method developed by Glorius and co-workers.[16] For the robustness assement of the synthesis of the lactone, two stock solutions were prepared.

*stock solution A:* In a 10 mL Schlenk tube with a magnetic stir bar were added **PTH** (46.8 mg, 0.170 mmol, 10 mol%), MeCN (13.6 mL), distilled water (92 µL, 5.1 mmol, 3.0 equiv.) and styrene **1a** (194 µL, 1.70 mmol, 1.00 equiv.) in this order. The mixture was stirred continuously throughout the execution of the robustness screen.

*stock solution B:* In a 10 mL Schlenk tube with a magnetic stir bar were added 6,7-dioxaspiro[3.4]octane-5,8-dione **2a** (483.2 mg, 3.40 mmol, 2.00 equiv.) and MeCN (3.4 mL). The mixture was stirred continuously throughout the execution of the robustness screen.

**Standard conditions:** All reactions were executed in 10 mL Schlenk tubes with magnetic stir bars. Each Schlenk tube was charged with 0.8 mL of (A), 0.1 mmol of the respective additive and 0.2 mL of (B). The mixtures were continuously flooded with argon. The reactions were stirred overnight at room temperature under irradiation with LEDs ($\lambda_{max}$ = 405 nm). To each mixture Mesitylene (14 µL, 0.10 mmol, 1.00 equiv.) was added as an internal standard for yield measurements via GC-FID. The results are shown in **Table S8** and **Figure S9**.

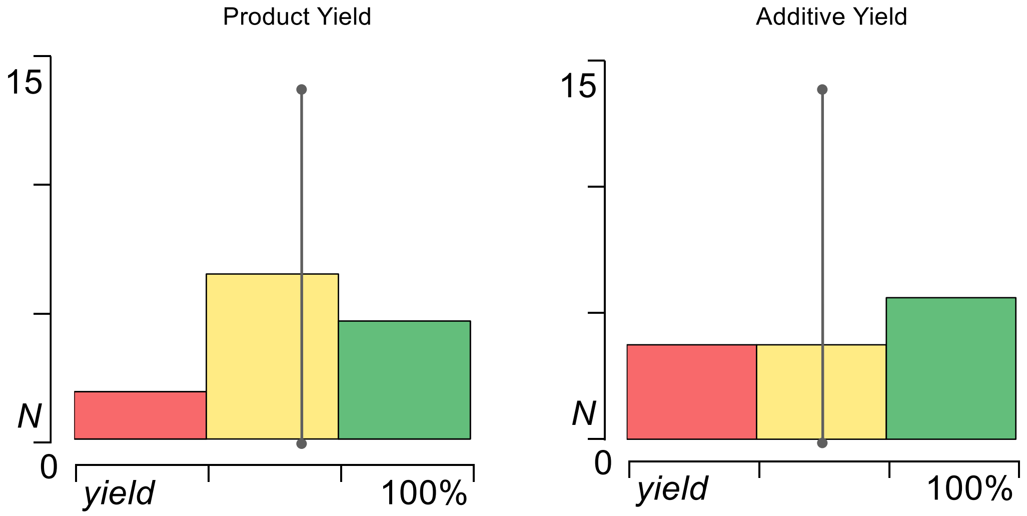


**Figure S8:** Bar graph representation of robustness screen. Left: Impact of additive normed on product yield. Right: Recovery of additive. Grey Line: average.

**Table S8**: Summary of additive-based robustness screen results.

| Entry | Additive | Additive Yield / % | Product Yield / % | Entry | Additive | Additive Yield / % | Product Yield / % |
|---|---|---|---|---|---|---|---|
| 1 | None | 0 | 80 | 9 | 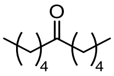 | 95 | 74 |
| 2 | 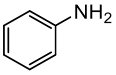 | 45 | 5 | 10 | 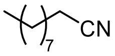 | 93 | 39 |
| 3 | 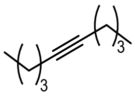 | 92 | 69 | 11 | 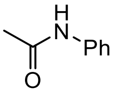 | 53 | 41 |
| 4 | 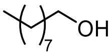 | 64 | 77 | 12 | 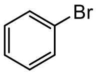 | 87 | 36 |
| 5 | 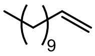 | 55 | 68 | 13 | 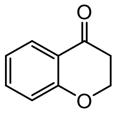 | 73 | 40 |
| 6 | 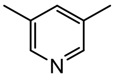 | 0 | 42 | 14 | 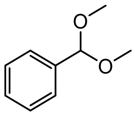 | 20 | 39 |
| 7 | 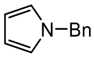 | 0 | 8 | 15 | 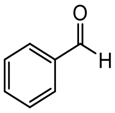 | 0 | 42 |
| 8 | 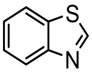 | 97 | 70 | | | | |
| **Average Additive Yield:** 55 % | | | | **Average Product Yield:** 46 % | | | |

[a] Yields were determined by GC-FID analysis using mesitylene as an internal standard. Ranges of 0% – 33% (red), 34% – 66% (yellow) and 67% – 100% (green.). Same ranges for product yields, relative to the reference reaction A0.

**Discussion of the robustness screen results:**

Entry 1: Reference reaction without any additive added.

Entry 2: Aniline was partially consumed, likely due to it's oxidation by **2a**. Since **2a** is preferably consumed in this side reaction, the product formation was supressed.

Entry 3: Alkyene additive was tolerated by the reaction, the additive was almost fully recovered.

Entry 4: The product yield was almost unaffected, indicating a good tolerance for free alcohols. Minor consumption of the alcohol can be attributed to the reaction of the additive with excess **2a**.

Entry 5: The product yield was almost unaffected, indicating a good tolerance for unactivated olefins. Minor consumption of the additive can be attributed to the reaction of the additive with excess **2a**, e.g. in a Tomkinson-type dihydroxylation.[2]

Entry 6: A lower product yield was observed in the presence of a pyridine. Full consumption of the additive indicates a preferred reaction of **2a** with the additive, potentially forming the N-oxide after oxidation. Therefore, only a minor amount of **2a** was left over for the productive reaction.

Entry 7: No additive was recovered and the reaction was suppressed completely. A likely reason is the preferred oxidation of the weak C-H bond, which is precedented in literature.[17]

Entry 8: In the presence of a benzothiazole, the yield of the reaction was almost unaffected. Full recovery of the additive indicated a perfect tolerance for benzothiazoles.

Entry 9: In the presence of a ketone, the yield of the reaction was almost unaffected. Full recovery of the additive indicated a perfect tolerance for aliphatic ketones. No Baeyer-Villiger-type oxidation was observed.

Entry 10: A decrease in yield was observed in the presence of a linear nitrile. Since the additive was fully recovered, this indicates an inhibition of the reaction, rather than a full intolerance.

Entry 11: In the presence of a N-phenyl substituted secondary amide, a decrease in reaction yield was observed. Since only half of the additive was recovered, a competing oxidation of the amide nitrogen by **2a** might be an explanation for the limited tolerance.

Entry 12: A decrease in yield was observed in the presence of bromobenzene. Since the additive was almost fully recovered, this indicates an inhibition of the reaction, rather than a full intolerance.

Entry 13: A decrease in yield was observed in the presence of benzochromane. Since a major amount of the additive was recovered, this indicates an inhibition of the reaction, rather than a full intolerance.

Entry 14 & 15: In the presence of an unprotected or acetal-protected aldehyde, the reaction yield dropped significantly. At the same time, none of the additives were recovered in meaningful quantities, indicating the reaction's intolerance against these functional groups. A possible explanation is the facile oxidation of the aldehyde carbon, which also accounts for the consumption of **2a** and the low reaction yield.

# 3. MECHANISTIC INVESTIGATIONS

## 3.1. UV/vis absorption spectroscopy

Optical spectroscopic studies in the Wenger lab were conducted at 293 K. Steady-state UV-Vis absorption spectra were recorded using a Cary 5000 spectrophotometer (Agilent Technologies). The cuvette pathlength was 10 mm.

Extinction coefficients were obtained by preparing 5 different solutions with different concentrations and recording their respective UV/Vis absorption spectra. Dividing by the concentration gave overlapping extinction coefficient spectra for all three complexes. Especially **Figure S11** (zoomed in) shows that **PTH-O** has the most intense absorption in the visible region up to 460 nm, explaining reactivity even upon 425 nm irradiation.

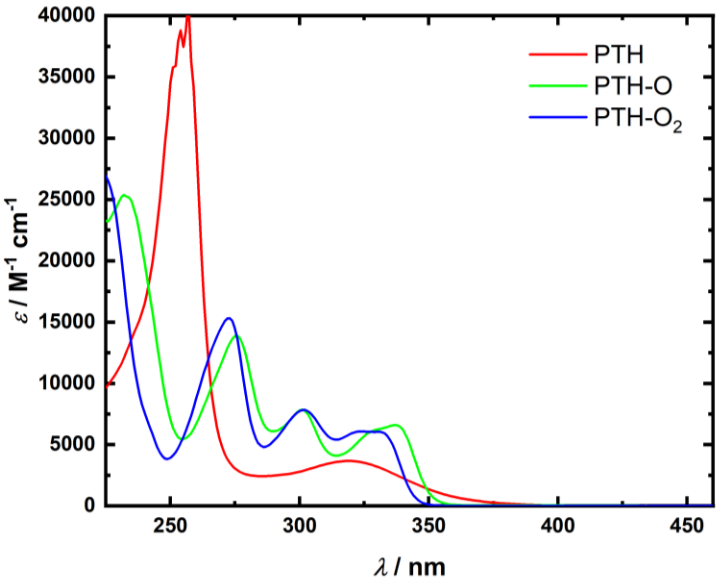


**Figure S9:** Extinction coefficient spectra of **PTH**, **PTH-O** and **PTH-$O_2$** in MeCN.

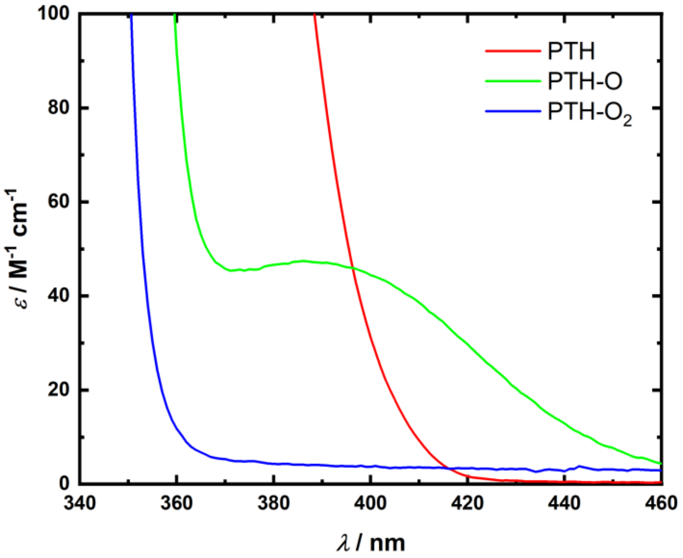


**Figure S10:** Magnified extinction coefficient spectra of **PTH**, **PTH-O** and **PTH-$O_2$** in MeCN.

UV/vis absorption spectra in the Glorius lab were recorded on a Jasco V-730 spectrophotometer, equipped with a temperature control unit at 25 °C. The samples were measured in a Starna® fluorescence quartz cuvettes (type: 29-F) with a chamber volume of 1.400 mL and dimensions of 48 mm × 12.5 mm × 12.5 mm, and a path length of 10 mm. Degassed solutions of styrene 1a (0.1 mM), 2a (1.0 mM), PTH (25 μM) and PTH-O (0.1 mM) in dry MeCN were prepared and measured.

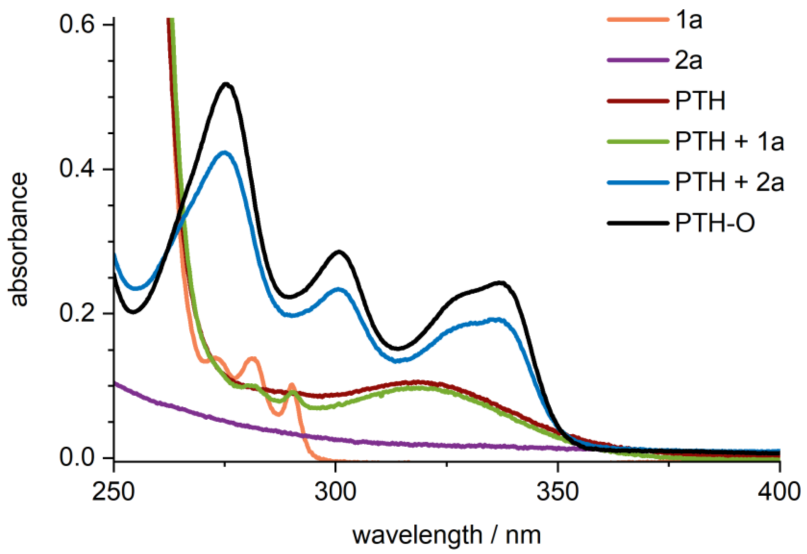


**Figure S11:** Magnified absorption spectra of solutions of **1a**, **2a**, **PTH**, **PTH-O** and mixtures in MeCN.

## 3.2. Steady-state emission spectroscopy

Steady-state luminescence spectra of an approximately 10 mM solution of PTH-O in MeCN were measured on a Fluorolog-322 from Horiba Jobin-Yvon, equipped with a Xenon lamp 450 W Illuminator (FL-1039A/40A) and a water-cooled photomultiplier tube (PMT Hamamatsu R2658 or R928). The luminescence spectra were corrected for the spectral response of the system.

The spectra were corrected for Raman scattering of the solvent by recording emission spectra at different excitation wavelengths to identify the Raman bands. An emission spectrum under the same settings and conditions of pure MeCN was recorded and this spectrum was subtracted from the sensitizer emission spectrum.

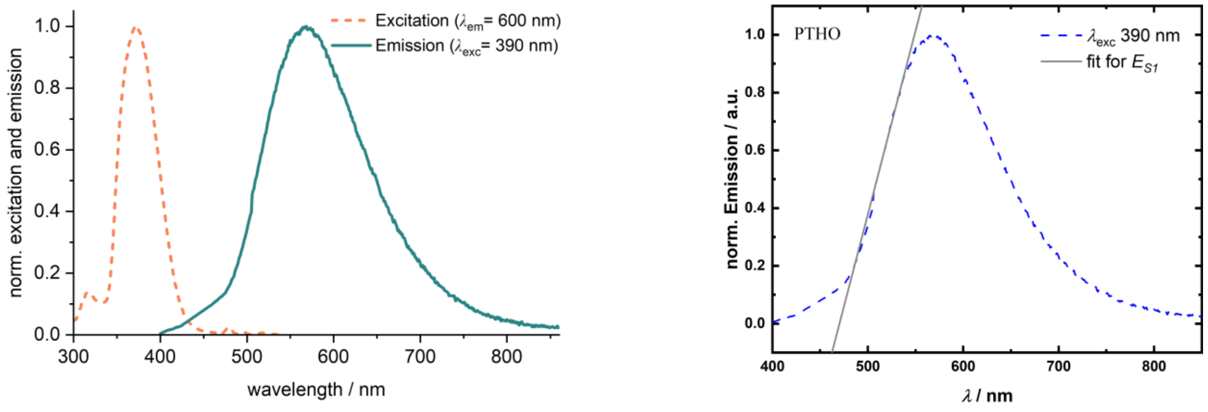


**Figure S12** Excitation and emission spectrum of **PTH-O** as well as determination of the S1 excited state energy by fluorescence onset.

### 3.3. Lifetime measurements and Stern-Volmer analysis

Photoluminescence lifetime studies were performed on an FLS1000 spectrometer (time-correlated single photon counting technique) from Edinburgh Instruments using a pulsed LED for excitation at 405.0 nm (Edinburgh Instruments, pulse width: 59.7 ps), where the cuvette holder was equipped with a temperature controller (TC 1, Quantum Northwest). For quenching experiments, the concentration of **PTH-O** was set to 0.2 mM. The lifetime in the absence of quencher was determined at $\tau$ =1.14 ns.

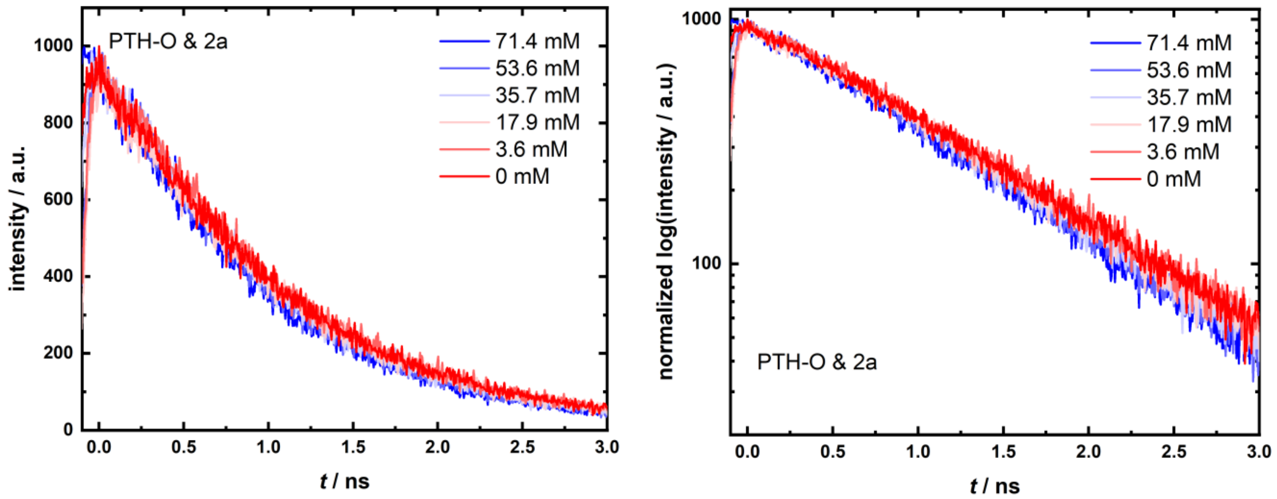


**Figure S13:** Decays of excited state **PTH-O** in TCSPC analysis in the absence and presence of **2a** as quencher.

**Table S9:** Raw data for the Stern-Volmer lifetime quenching.

| | | | | | |
|---|---|---|---|---|---|
| [**2a**] / mM | 0 | 3.57 | 17.86 | 35.71 | 53.57 | 71.42 |
| $\tau_0 / \tau$ | 1 | 0.98804 | 0.99918 | 1.05886 | 1.08569 | 1.09386 |
| | | | | | | |
| [**1a**] / mM | 0 | 2.5 | 12.7 | 24.4 | 71.15 | |
| $\tau_0 / \tau$ | 1 | 0.99153 | 0.99153 | 1.00862 | 0.99153 | |

## 3.4. Cyclic Voltammetry and spectroelectrochemistry

### 3.4.1. Cyclic voltammetry

Cyclic voltammetry was performed under $N_2$ or Ar atmosphere in a three-necked electrochemical cell. A glassy carbon disc electrode, a silver wire, and a saturated calomel electrode were used as working, counter, and reference electrodes, respectively. Sample solutions (1.0 mM) were prepared in dry MeCN with supporting electrolyte (0.1 M, tetra-*n*-butylammonium hexafluorophosphate, $TBAPF_6$) and solutions were bubbled with $N_2$ or Ar for 5 min prior to each measurement. The scan rate was 100 mV $s^{-1}$ unless stated otherwise and the response current signals were recorded on a Versastat4-200 potentiostat from Princeton Applied Research. Unless otherwise stated, spectra were recorded starting at an initial voltage of 0 V scanning towards positive potentials first. For irreversible redox events, the $E_{1/2}$ value was taken as the corresponding value, as has been described by Espinoza and coworkers.[18]

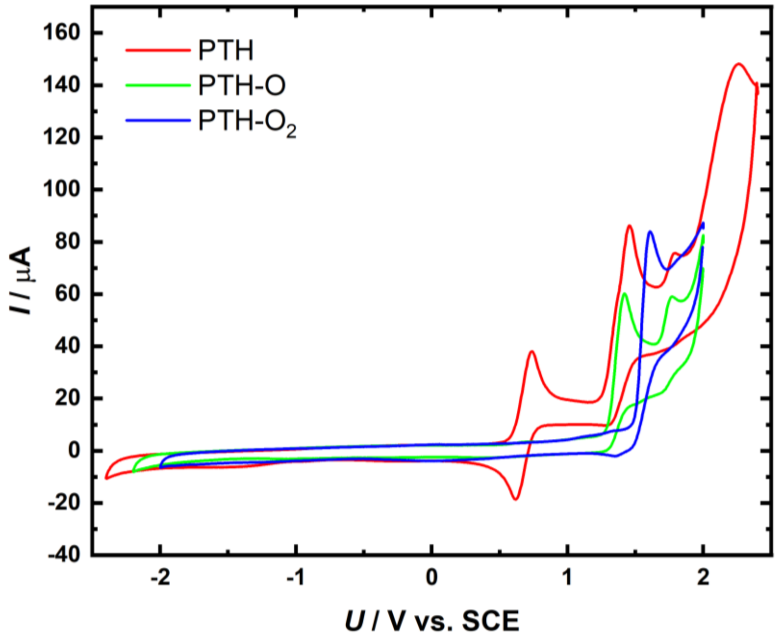


**Figure S14:** Cyclic voltammetry of **PTH, PTH-O** and **PTH-O₂** in 0.1 M $TBAPF_6$, using a glassy carbon disk working electrode, Ag wire counter electrode and an SCE reference electrode at 0.1 V/s scan rate.

### 3.4.2. Spectroelectrochemistry and time-resolved absorption spectra

Spectro-electrochemical measurements (SEC) by recording the UV-vis absorbance spectral changes upon electrochemical reduction or oxidation were measured using $N_2$ bubbled samples in dry MeCN in a cuvette with an optical path length of 1 mm. To a sample solution (typically 250 μM) containing a supporting electrolyte (0.1 M, $TBAPF_6$), continuous voltage was applied using Pt mesh, Pt wire, and saturated calomel electrodes as working, counter, and reference electrodes, respectively. The applied voltage was controlled by a Versastat4-200 potentiostat from Princeton Applied Research. The resulting UV-vis absorbance differences were recorded on a OceanHDX miniature spectrometer from Ocean Optics. Time-resolved UV/Vis absorption measurements (see manuscript) were carried out on the same setup without any applied voltage.

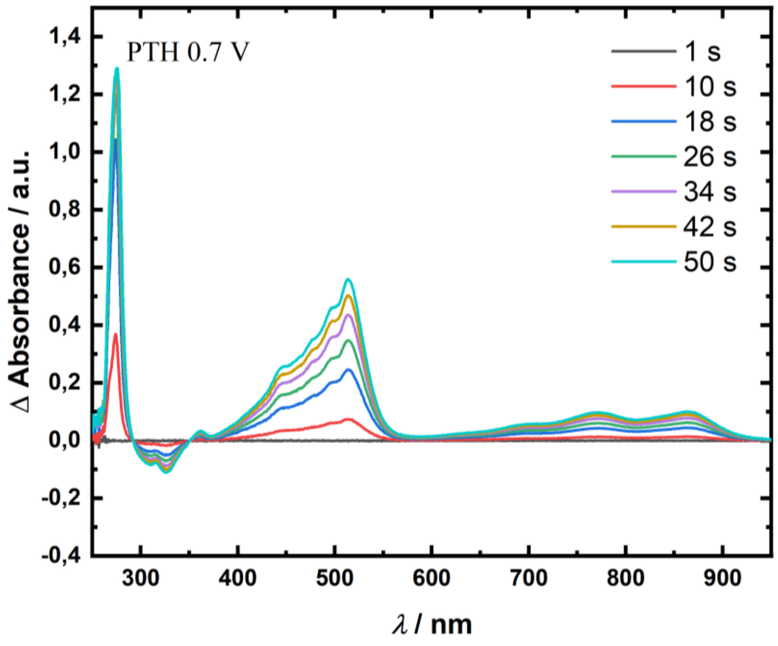


**Figure S15:** UV/Vis absorption difference spectra of **PTH** at an applied voltage of 0.7 V vs SCE.

The operation mode of recording difference spectra as the difference to a reference spectrum as the baseline shows the accumulation of PTH+ after time at a constant potential. The potential was kept at 0.7 V to prevent overoxidation and decomposition from $PTH^{2+}$.

### 3.5. Transient absorption measurements

UV-vis transient absorption measurements on the picosecond timescale were carried out with a TRASS instrument from Hamamatsu and a mode-locked picosecond $Nd:YVO_4$/YAG laser from Ekspla (PL2251B-20-SH/TH/FH with PRETRIG option, ca. 30 ps pulse width) as excitation light source. The frequency-tripled (355 nm) output of this laser powered an Ekspla PG402-264 OPA with a resulting energy output of max. 6 mJ at 405 nm. The solution was prepared with a concentration of 0.5 mM **PTH-O**, 10 mM **2a**, 5 mM **1a** and 15 mM $H_2O$, resembling the ratios actually used in the photocatalytic experiments.

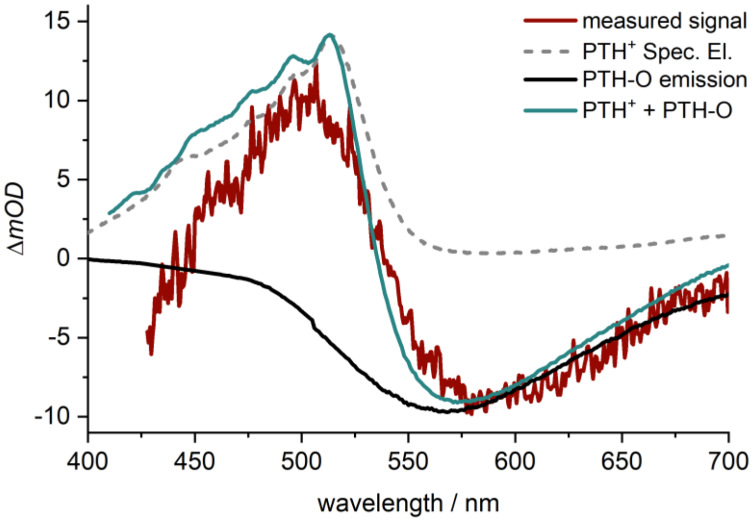


**Figure S16:** Transient absorption spectrum of the reaction mixture and pure samples for comparison.

Furthermore, the recorded data shows that after the laser is activated, only one signal emerges in the transient absorption spectrum. This signal is the stimulated emission signal of **PTH-O,** which decays completely and does not transform into another signal. The other signal present in the TA data is the ground state absorption of the PTH radical cation, which can be observed throughout the whole measurement and even before the laser is activated.

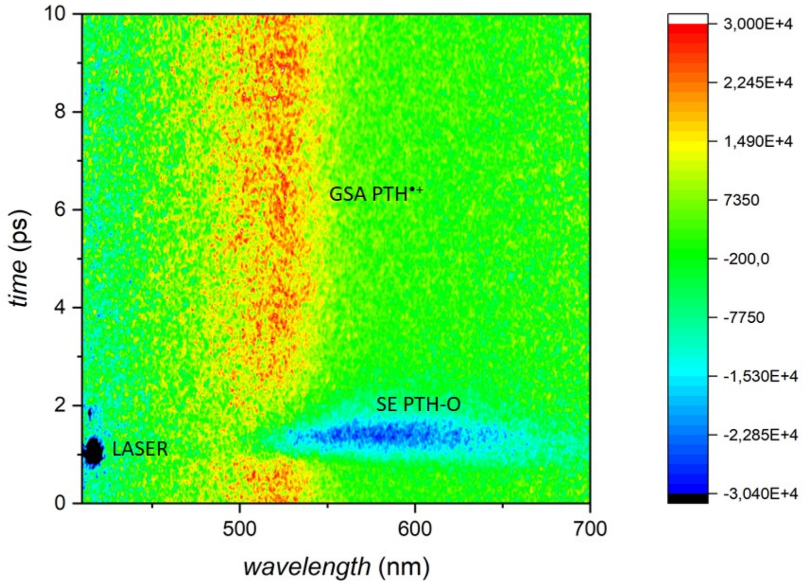


**Figure S17:** Transient absorption spectroscopy heatmap of the reaction mixture.

### 3.6. Trapping Experiments

A reaction was set up according to General Procedures A and allowed to react in the photoreactor for 10 min. While kept under irradiation in the photoreactor, the reaction tube was opened and 5.0 equiv. of TEMPO or BHT, respectively, were added to the reaction. The reaction was subsequently analyzed by negative ESI-HRMS to detect trapped species.

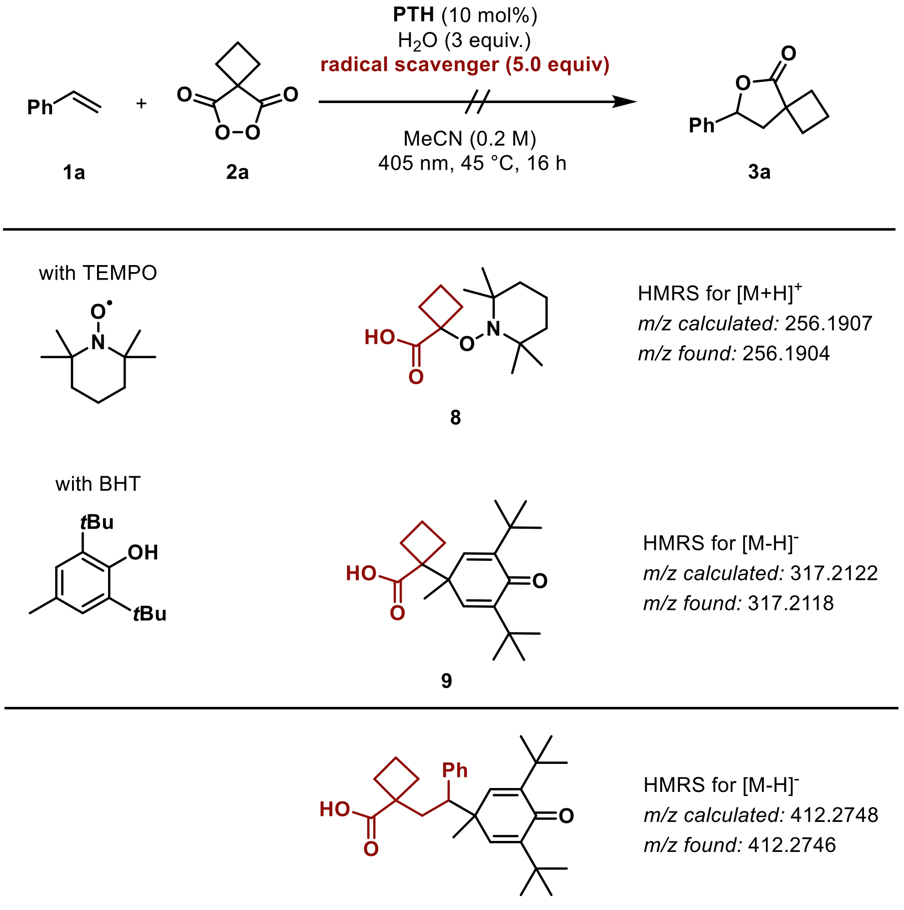


10

**Figure S18**: Trapping agents TEMPO and BHT shut down product formation and yield radical adducts, that were detected by ESI-HRMS.

## 3.7. Isotopic labelling experiments

Isotopic labelling experiments were carried out in order to investigate both the origin of the oxygen atom in **PTH-O** as well as in the product's lactone group. The computer software UMC (Universal Mass Calculator) was used to (e.g. simulate isotope patterns, evaluate the degree of deuteration/labelling, evaluate elemental compositions). UMC Version 3.14.0.101, Dr. Matthias C. Letzel, WWU Münster, Org.-Chem. Institut, Germany. https://www.uni-muenster.de/Chemie.oc/ms/downloads.html.

### 3.7.1. Synthesis of 18-oxygen labelled peroxide

$^{18}O$-labelled **2a** was synthesized according to a protocol by Tomkinson and coworkers[19]. The intermediate cyclobutane-1,1-dicarbxoylic acid was obtained in quantitative yield with 68% 4-fold $^{18}O$-incorporation and 32% 3-fold $^{18}O$-incorporation (**Figure S19**). After transforming the dicarboxylic acid into peroxide **$^{18}$O-2a**, the product showed 66% 2-fold $^{18}O$-incorporation and 28% 1-fold $^{18}O$-incorporation (**Figure S20**)

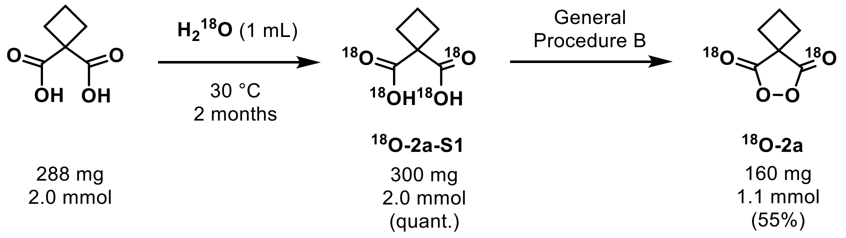


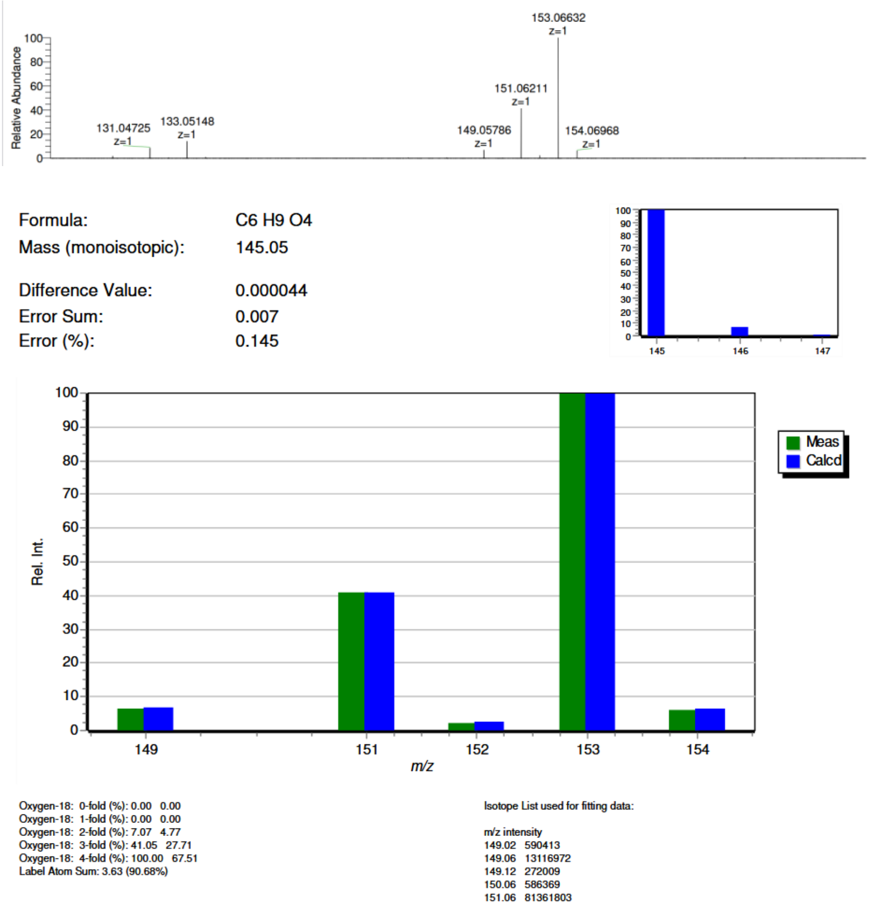


**Figure S19**: Incorporation of 18-oxygen into cyclobutane-1,1-dicarboxylic acid **$^{18}$O-2a-S1**.

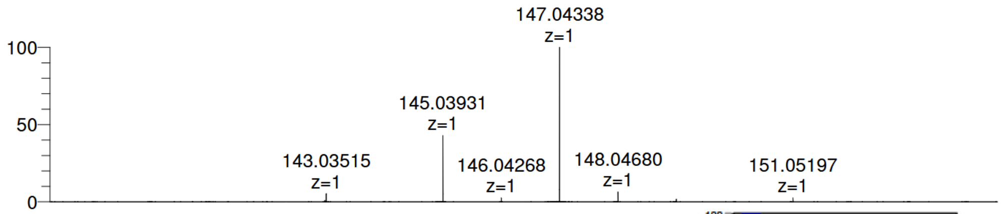


Formula: C6 H7 O4
Mass (monoisotopic): 143.03

Difference Value: 0.000052
Error Sum: 0.007
Error (%): 0.133

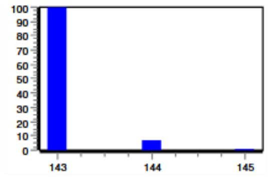


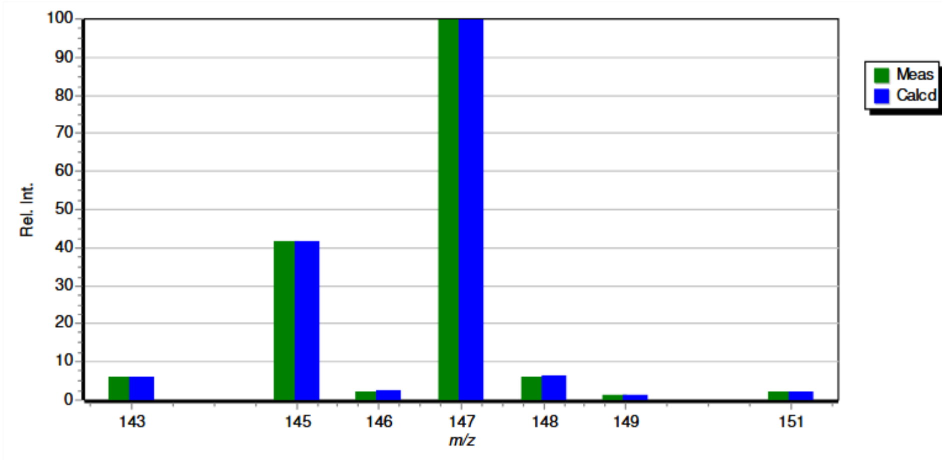


Oxygen-18: 0-fold (%): 6.22 4.09
Oxygen-18: 1-fold (%): 42.20 27.78
Oxygen-18: 2-fold (%): 100.00 65.83
Oxygen-18: 3-fold (%): 1.02 0.67
Oxygen-18: 4-fold (%): 2.48 1.63
Label Atom Sum: 1.68 (41.99%)

Isotope List used for fitting data:

| m/z | Intensity |
|---|---|
| 143.01 | 12038906 |
| 145.04 | 81718648 |
| 146.04 | 4626237 |
| 147.04 | 194369616 |
| 148.05 | 12205888 |
| 149.05 | 3155630 |
| 151.05 | 4850519 |

**Figure S20**: Incorporation of 18-oxygen into peroxide **$^{18}$O-2a**.

### 3.7.2. Probing the source of oxygen in PTH-O

To probe the source of oxygen in PTH-O, a mixture of PTH (25 µmol, 7 mg), peroxide 2a (2.0 equiv., 50 µmol, 7 mg) and water (5.0 equiv., 125 µmol, 2.3 µL) in MeCN (500 µL) was reacted for 5 min at room temperature. The mixtures were subsequently analyzed by HRMS.

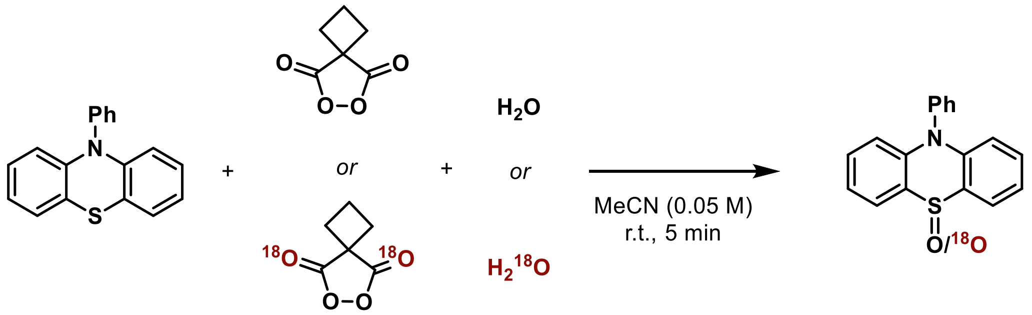


**Table S10:** 18-oxygen incorporation studies.

| entry | variation | result |
|---|---|---|
| #1 | $^{16}$O-peroxide, $^{16}$O-water | no $^{18}$O-incorporation |
| #2 | $^{18}$O-peroxide, $^{16}$O-water | no $^{18}$O-incorporation |
| #3 | $^{16}$O-peroxide, $^{18}$O-water | $^{18}$O-incorporation |
| #4 | $^{18}$O-peroxide, $^{16}$O-water, added 1.0 equiv. styrene | no $^{18}$O-incorporation |
| #5 | $^{16}$O-peroxide, $^{18}$O-water, added 1.0 equiv. styrene | $^{18}$O-incorporation |

#1

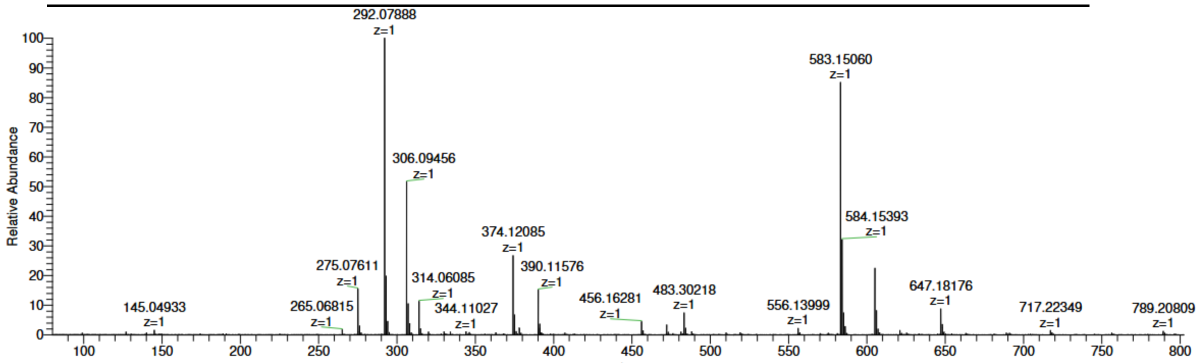


#2

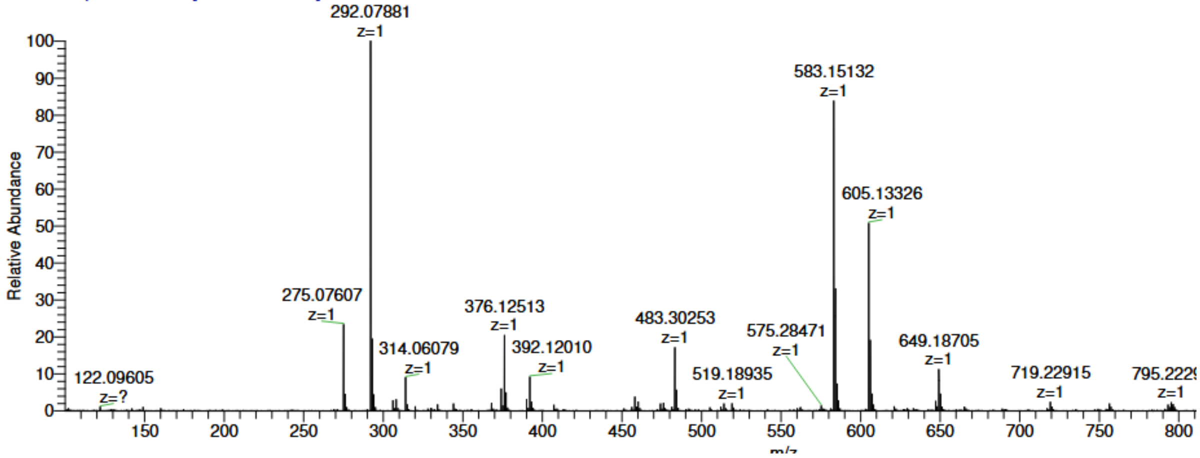

#3

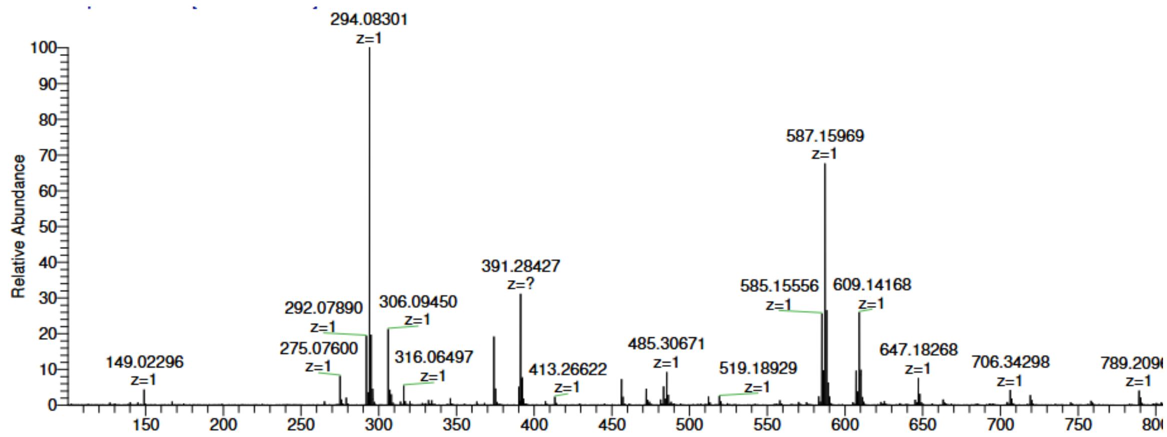


#4

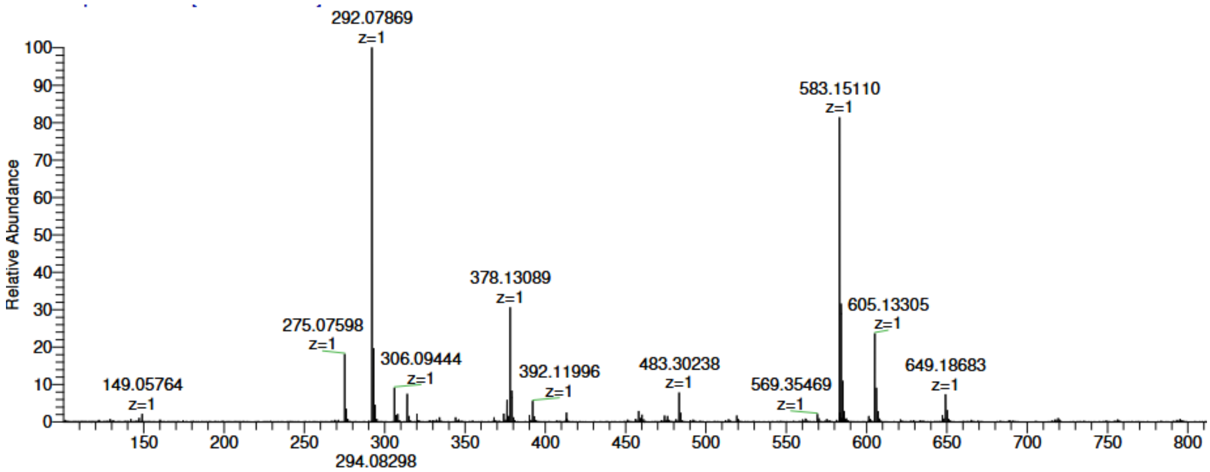


#5

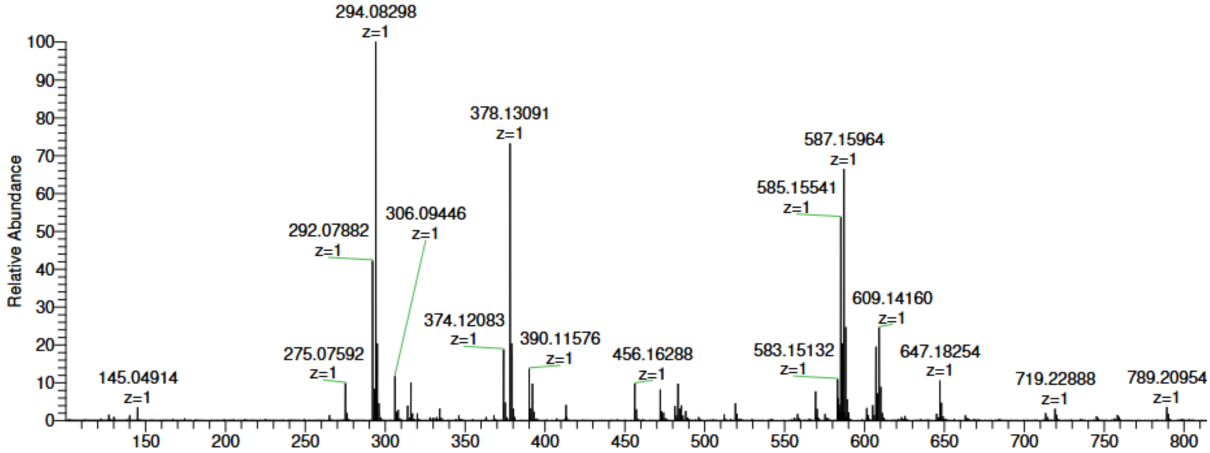


In case of entry #5, residual water in the reagents (styrene) is likely to cause the observation of some 16-oxygen **PTH-O**, although the majority of the detected m/z signals corresponds to 18-oxygen **PTH-O**.

### 3.7.3. Probing the incorporation of oxygen into the lactone product

To investigate the investigation of oxygen-18 into the lactone product, the standard reaction according to general procedure A was carried out using the $^{18}$**O-2a**:

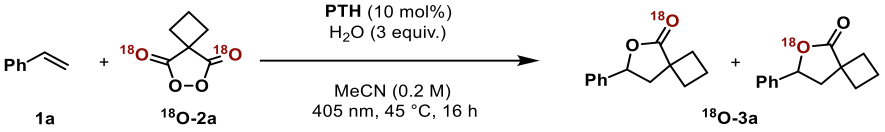


Analysis of the purified product (isolated in 72% yield) by IR-spectroscopy revealed that the incorporated heavy oxygen is occupying both the carbonyl as well as the ester-oxygen position in an almost statistical mixture ($^{16}$O was slightly more expressed due to the incomplete labelling of **2a**). This is consistent with the proposed mechanism, where carboxylate **7** can attack the formed benzylic cation with both of its equivalent oxygen atoms.

Unlabelled **3a**:

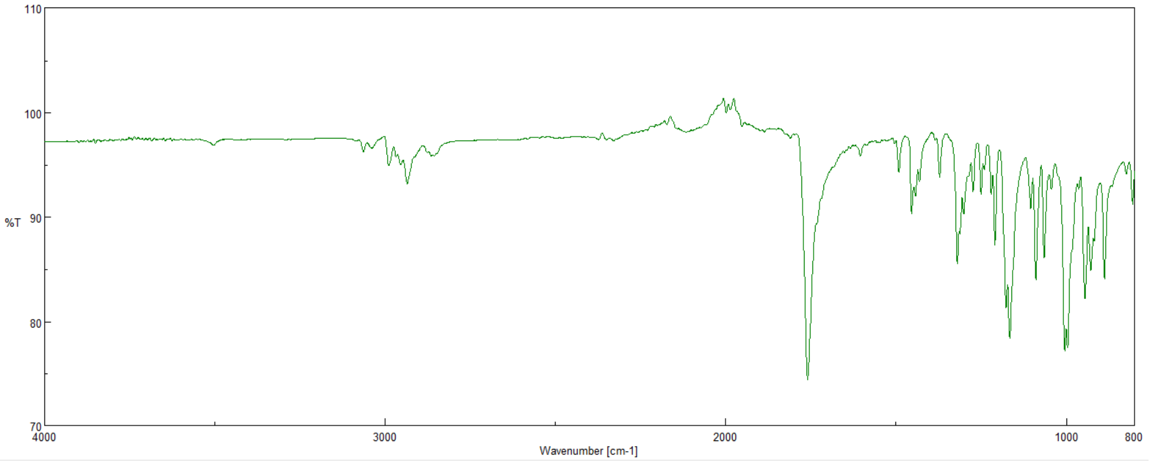


**Figure S21**: IR-Spectrum of isotopically unlabelled **3a**.

$^{18}$O-labelled **3a**:

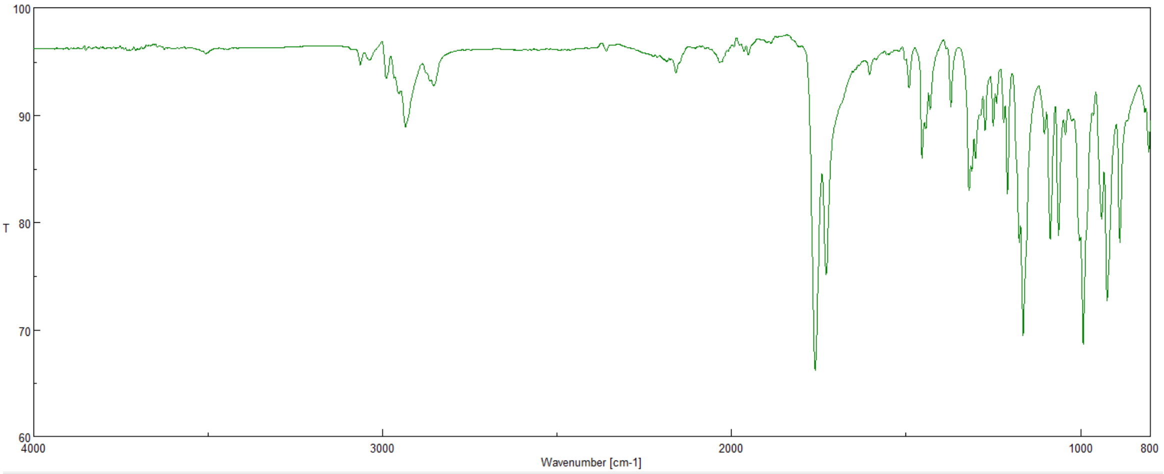


**Figure S22**: IR-Spectrum of isotopically unlabelled **3a**.

### 3.8. Dark experiments

In order to obtain information about the background reactivity, the reaction was performed under the exclusion of light with different (pre-)catalyst species and increasing catalyst and peroxide loadings:

**Table S11:** Dark reactions between **1a** and malonyl peroxide **2a** in the presence of varying amounts of **PTH**.

| entry | **PTH** loading | **3a yield** |
|---|---|---|
| #1 | 10 mol% | 21% |
| #2 | 5 mol% | 8% |
| #3 | 10 mol% | 16% |
| #4 | 25 mol% | 24% |
| #5 | 50 mol% | 11% |
| #6 | 50 mol%, 3.0 eq. peroxide | 65% |
| #7 | 100 mol% | 0% |
| #8 | 100 mol%, 3.0 eq. peroxide | 41% |

Reaction performed on a 0.1 mmol scale in MeCN (0.1 M) using styrene, **2a** (2.0 equiv.) in the dark for 24 h. Product yields determined by GC-FID using mesitylene as internal standard.

**Discussion:** As retrieved from **Table S11**, a significant increase of the dark background reactions product yield is observed when the loading of **PTH** is increased. However, when moving to catalysts loadings of 50% and higher, additional peroxide is required to further increase the product yield, indicating that the majority of the peroxide is consumed by reaction with the catalyst species. When the amount of peroxide is increased, a further rise in product yield is observed (entry 6). Note that, in contrast, when **PTH-O** is directly employed a photocatalyst, only traces of product are observed, which is also not dependent on the catalyst or peroxide loading.

This data agrees with our proposed mechanism and supports the release of reactive, lactone-forming radicals by SET of ground state **PTH** with **2a**. Also, since a large excess of peroxide is needed to promote product formation for PTH loadings > 50%, this supports that **2a** is as well consumed in other elementary steps, for example a second SET from **PTH** to **PTH**$^{\bullet+}$, or for the oxidative radical-polar crossover from intermediate **7**.

## 3.9. Reactivity of different photocatalyst species

To investigate the photocatalytic activity of different species – or their potential to be transformed into an active species, several experiments were carried out according to general procedure A. In each experiment, we employed different species instead of **PTH** and evaluated the reaction performance.

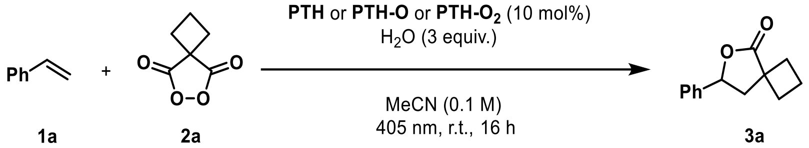


Based on this study, we propose that **PTH-O** is the most oxidized identified species of **PTH** which is still active in catalyzing the desired reaction. The reaction yield is comparable when employing **PTH**, **PTH**$^{\bullet+}$ or **PTH-O**, in agreement with our mechanistic hypothesis that all of these species are in situ interconverted to **PTH-O**, which is the actual active catalyst species.

**Table S12:** Photocatalytic lactonization reactions using different PTH species employed.

| entry | photocatalyst species | **3a** yield |
|---|---|---|
| #1 | **PTH** | 69% |
| #2 | **PTH**$^{\bullet+}$ (as PTH-BF4) | 66% |
| #3 | **PTH-O** | 70% |
| #4 | **PTH-O$_2$** | 15% |

Reaction performed on a 0.1 mmol scale in MeCN (0.1 M) using styrene, **2a** (2.0 equiv.) and a **PTH** photocatalyst species (10 mol%) under 405 nm (18 W) irradiation for 16 h. Product yields determined by GC-FID using mesitylene as internal standard.

## 3.10. Quantum yields measurement

### Determination of the photon flux

The photon flux was determined by ferrioxalate actinometry similar to a procedure by Yoon.[20] A 3 W violet LED ($\lambda_{max}$ = 395 nm) was used for the quantum yield measurement instead of the standard 18 W blue LEDs ($\lambda_{max}$ = 405 nm).

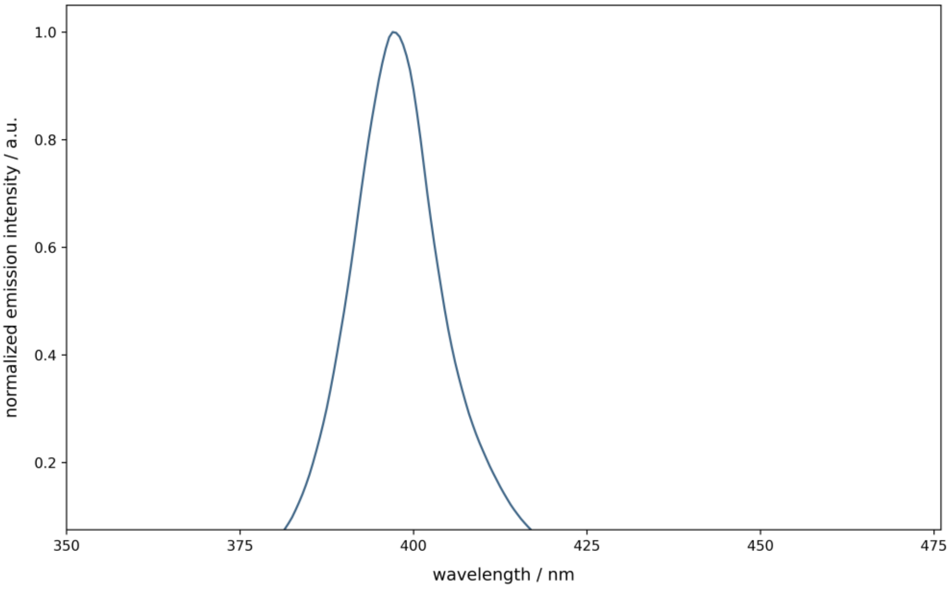


**Figure S22**: Emission profile of the 3 W 395 nm LED used for the determination of quantum yields.

A solution (10 mL, 0.15 M) of potassium ferrioxalate hydrate (737 mg, 1.50 mmol) in aq. $H_2SO_4$ (0.050 M) and a solution (20 mL, 0.005 M) of 1,10-phenanthroline monohydrate (20 mg, 0.10 mmol) and sodium acetate (4.50 g) in aq. $H_2SO_4$ (0.50 M) were prepared and kept in the dark. All following steps were conducted in the dark as well.

Four Schlenk tubes were charged with the prepared ferrioxalate solution (1.0 mL) and two of these tubes were successively irradiated with a violet LED for 60 s at a distance of 5 cm, while the other two tubes were left in the dark. Then, to all four Schlenk tubes was added the prepared phenanthroline solution (175 µL each) and the reaction mixtures were stirred for 60 mins. For all four solutions, the absorbance at 510 nm was measured and the difference between the average absorbance of the three irradiated samples and the average absorbance of the three control samples was determined ($\Delta A_{510\ nm}$ = 2.672).

The formed amount of Fe(II) was calculated based on the Lambert–Beer law (equation 2) with $V = 1.175 \cdot 10^{-3}$ L $l$ = 1.0 cm and ε = 11100 L·mol$^{-1}$·cm$^{-1}$. [21]

$$n_{Fe(II)} = \frac{V \cdot \Delta A510\ \mathrm{nm}}{l \cdot \varepsilon} \quad (2)$$

The fraction of light which was absorbed by the actinometer at $\lambda$ = 395 nm ($f$) was determined with equation 3 with the absorbance of the ferrioxalate stock solution at $\lambda$ = 395 nm being $A_{395\ nm} > 3$ ($f > 0.999$).

$$f = 1 - 10^{-A_{395nm}} \quad (3)$$

The photonflux $\phi_q$ was finally determined using equation 4 with $\phi_F$ = 1.13 (at $\lambda$ = 392 nm) and t = 60 s, giving $\phi_q$ = 3.42094·10$^{-9}$ mol·s$^{-1}$.[21,22]

$$\phi_q = \frac{n_{Fe(II)}}{\phi_F \cdot t \cdot f} \quad (4)$$

**Table S13:** Results of the photon flux determination.

| entry | $A$(510 nm) | $A_{control}$(510 nm) | Δ$A$ |
|---|---|---|---|
| #1 | 2.50401 | 0.312664 | |
| #2 | 2.48641 | 0.299964 | |
| **average** | **2.49521** | **0.306314** | **2.188896** |

Reaction performed on a 0.1 mmol scale in MeCN (0.1 M) using styrene, **2a** (2.0 equiv.) and a **PTH** photocatalyst species (10 mol%) under 395 nm (3 W) irradiation. Product yields determined by GC-FID using mesitylene as internal standard.

### Measurement of the reaction quantum yield

In an 10 mL Schlenk Tube equipped with a PTFE-coated stir bar was added **PTH** (2.9 mg, 10 mol%). The Schlenk tube was evacuated and backfilled with argon three times before MeCN (1 mL,0.2 M) was added under a positive argon pressure. The respective olefin **2a** (12µL, 1.0 equiv., 0.1 mmol), water (5.4 µL, 3.0 equiv., 0.3 mmol). While stirring, solid malonyl peroxide (28.4 mg, 2.0 equiv, 0.2 mmol). The Schlenk Tube was sealded and irradiated at 395 nm at ambient temperature for the indicated time using the same setup as for the photon flux determination. Then yield of the product formation was analyzed using GC-FID using mesitylene as internal standard. The same procedure was repeated two more times giving the same yield. The reaction's quantum yield was determined using equation 5 with the determined $\phi_q$ the irradiation time and the fraction of light absorbed ($f$ > 0.999; determined according to equation 3 with $A_{395\ nm}$ > 3) by the reaction mixture.

$$\phi_{rxn} = \frac{n_{product}}{\phi_q \cdot t \cdot f} \quad (5)$$

**Table S14:** Results of the reaction quantum yield determination

| entry | reaction time | yield | quantum yield |
|---|---|---|---|
| #1 | 20 min | 2.2% | 0.54 |
| #2 | 60 min | 6.6% | 0.54 |
| #3 | 60 min | 7.2% | 0.58 |
| **average** | | | **0.55** |

Reaction performed on a 0.1 mmol scale in MeCN (0.1 M) using styrene, **2a** (2.0 equiv.) and a **PTH** photocatalyst species (10 mol%) under 395 nm (3 W) irradiation. Product yields determined by GC-FID using mesitylene as internal standard.

### 3.11. Check for dihydroxylation reactivity

A reaction between styrene **1a** and peroxide **2a** was set up according to general procedure A on a 0.1 mmol scale. After 16 h of irradiation, the crude reaction mixture was checked for any of the reported dihydroxylation intermediates[2,19] by ESI-HRMS (possible products and their exact mass are shown).

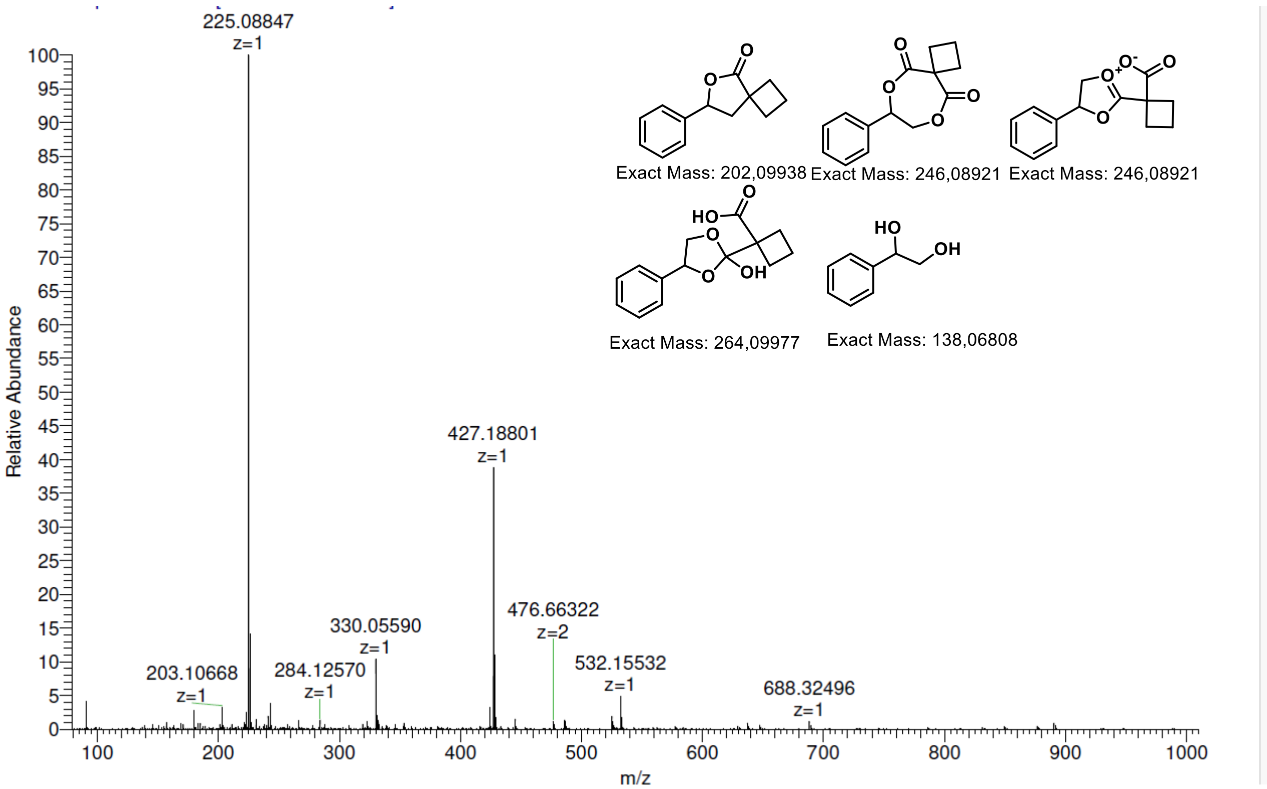


**Figure S24**: HRMS-Spectrum of the crude product before saponification and comparison to the intermediates observed by Tomkinson and coworkers.

The crude product was then stirred in aq. sodium hydroxide solution (1 M) for 4 h at 60 °C and subsequently extracted with chloroform (3x), according to the workup from Tomkinson et al.[2] To the crude NMR of the combined & dried organic layers was added $CH_2Br_2$ (0.1 mmol). The NMR showed no characteristic peaks of the diol reported by Tomkinson and coworkers (NMR on next page).

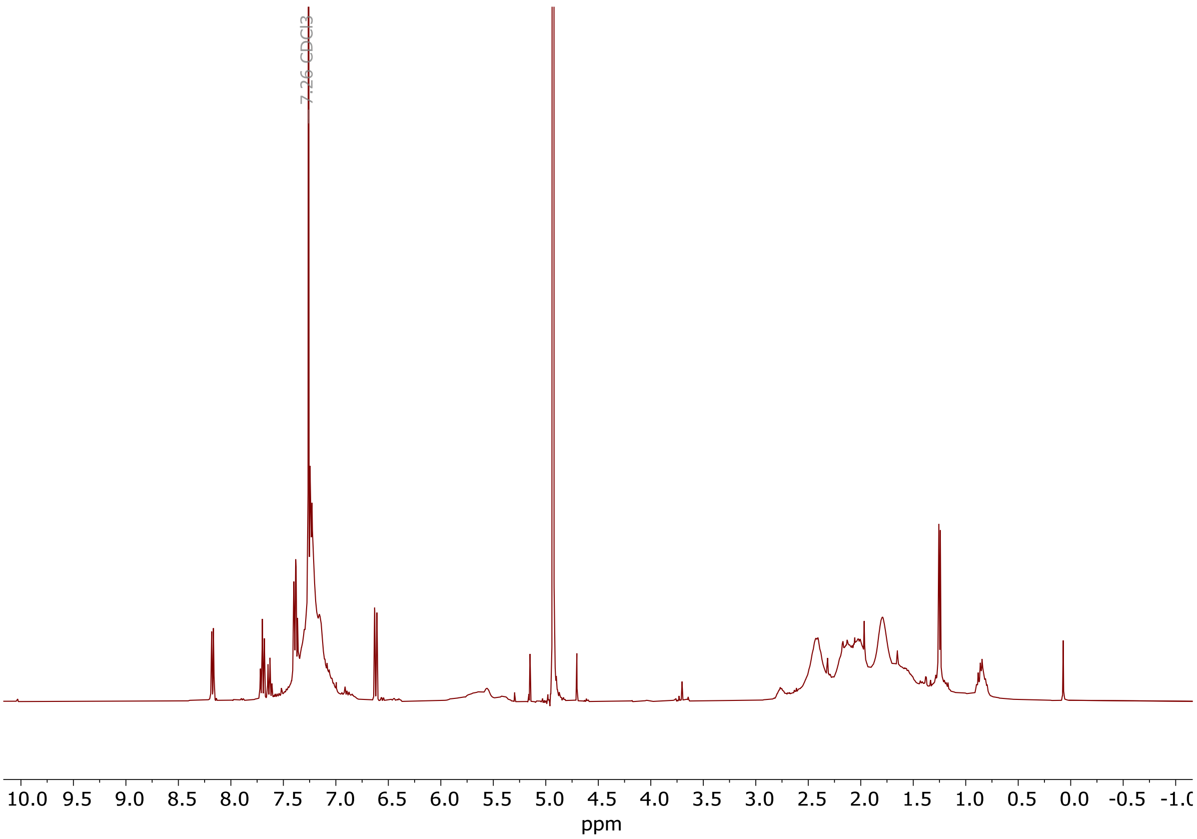


**Figure S25**: NMR-spectrum after the saponification showing no product for the dehydroxylated product.

# 4. COMPUTATIONAL STUDIES

## 4.1. Density functional theory

Density functional theory (DFT) calculations were performed using the ORCA 6.0.1[23] software package on the local high-performance computing cluster of the GLORIUS group, equipped with Intel® Xeon® Gold 6240 CPUs. Resulting structures were visualized in Avogadro[24] and Chemcraft[25]. For the initial guess structure generation, conformers were sampled using RDKit starting from SMILES notations. The obtained lowest-energy was preoptimized using GFN2-xTB.[26]

Geometry optimizations, frequency calculations and single point calculations were conducted using the ωB97-D3 functional[27] and the ma-def2-QZVP basis set[28]. No constraints on internal coordinates or symmetry were imposed. In case of convergence issues, the *TightOpt* and *VerySlowConv* keywords were set. All calculations included Grimme´s D3 dispersion correction[29], the CPCM implicit solvation model[30] with presets for acetonitrile, and the RIJCOSX approximation.[31] The geometries were confirmed to represent local minima on the potential energy surface by verifying the absence of imaginary frequencies after optimization.

Time-dependent density functional theory (TD-DFT) calculations were performed at the CAM-B3LYP[32]/def2-TZVP[28] level of theory, considering 15 roots. For natural transition orbitals (NTOs), configurations with a contribution threshold of $10^{-3}$ were considered, the resulting plots were generated using a grid size of 100.

### 4.1.1. Decarboxylation of 2a

Radical Anion $^{2}$**2a**$^{\cdot-}$ (Intermediate **5**)

| | |
|---|---|
| $E_{SCF}$ / hartree | -533.3482184 |
| $G_{total}$ / hartree | -533.2665912 |

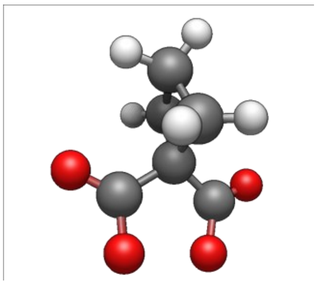

| | | | |
|---|---|---|---|
| O | -0.795071 | 2.306498 | 0.285457 |
| C | -1.264655 | 1.172121 | 0.353907 |
| O | -2.436494 | 0.938907 | 0.822421 |
| O | -2.579876 | -1.094010 | 0.291820 |
| C | -1.427044 | -1.221937 | -0.257744 |
| C | -0.480391 | -0.056518 | -0.060386 |
| C | 0.665981 | -0.347134 | 0.968664 |
| C | 1.681102 | 0.268032 | -0.012668 |
| C | 0.612571 | 0.185917 | -1.119757 |
| O | -1.095617 | -2.237803 | -0.865389 |
| H | 0.556617 | 0.116697 | 1.946807 |
| H | 0.802214 | -1.419964 | 1.092545 |
| H | 1.930067 | 1.294931 | 0.244821 |
| H | 2.599982 | -0.291822 | -0.173092 |
| H | 0.727197 | -0.697051 | -1.745348 |
| H | 0.471145 | 1.064218 | -1.745338 |

Intermediate **6**

| | |
|---|---|
| $E_{SCF}$ / hartree | -533.3694550 |
| $G_{total}$ / hartree | -533.2959180 |

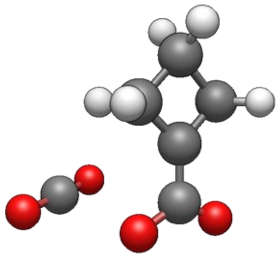

| | | | |
|---|---|---|---|
| O | -2.165153 | 2.451334 | 0.291133 |
| C | -2.444812 | 2.072163 | 1.346413 |
| O | -2.728952 | 1.745726 | 2.417989 |
| O | -2.261696 | -0.658374 | 0.501913 |
| C | -1.525207 | -0.689035 | -0.524715 |
| C | -0.102837 | -0.392301 | -0.340440 |
| C | 0.718342 | -0.029178 | 0.857400 |
| C | 1.957751 | 0.031060 | -0.077394 |
| C | 1.053622 | -0.327354 | -1.288642 |
| O | -1.913490 | -0.951521 | -1.697503 |
| H | 0.449375 | 0.918890 | 1.332445 |
| H | 0.759245 | -0.787869 | 1.643838 |
| H | 2.429589 | 1.009252 | -0.139677 |
| H | 2.719916 | -0.712655 | 0.144840 |
| H | 1.295414 | -1.265446 | -1.795945 |
| H | 0.974612 | 0.448117 | -2.056145 |

### 4.1.2. Calculation of redox potentials

For the calculation of redox potentials, a protocol by Nicewicz and coworkers was employed.[33] Gibbs free energies of the oxidized and reduced species were evaluated separately on the relaxed geometries. The reaction Gibbs free energy was calculated as the energy difference between the reduced and oxidized species and converted to a potential through division by the number of transmitted electrons (1) and the Faraday constant (23.061 kcal $mol^{-1}$ $V^{-1}$). To reference the potential, the absolute potential of SCE (4.42 V)[33] was subtracted from the obtained value:

$$E_{1/2,\mathrm{SCE}} = \frac{G_{reduced} - G_{oxidized}}{1 \cdot 23.061\ \frac{\mathrm{kcal}}{\mathrm{mol{\cdot}V}}} - 4.42\ \mathrm{V}$$

#### Geometries and reduction potential for 2a

Neutral **¹2a**

| | |
|---|---|
| $E_{SCF}$ / hartree | -533.1603570 |
| $G_{total}$ / hartree | -533.0747937 |

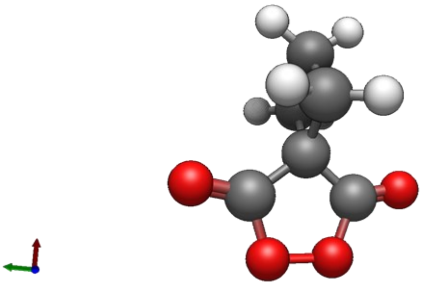

| | | | |
|---|---|---|---|
| O | -1.206022 | 2.226831 | 0.630932 |
| C | -1.388790 | 1.080424 | 0.368578 |
| O | -2.666498 | 0.586616 | 0.378965 |
| O | -2.640337 | -0.790284 | 0.028105 |
| C | -1.345937 | -1.172620 | -0.205318 |
| C | -0.442295 | -0.009815 | 0.008583 |
| C | 0.757794 | -0.230680 | 0.983705 |
| C | 1.756273 | 0.096148 | -0.142445 |
| C | 0.585897 | 0.299826 | -1.122730 |
| O | -1.119300 | -2.297290 | -0.521961 |
| H | 0.747937 | 0.465885 | 1.817232 |
| H | 0.799378 | -1.248400 | 1.361514 |
| H | 2.346175 | 0.991217 | 0.034904 |
| H | 2.422401 | -0.721695 | -0.402490 |
| H | 0.526862 | -0.419602 | -1.934915 |
| H | 0.474294 | 1.304729 | -1.520679 |

Radical Anion **²5**

| | |
|---|---|
| $E_{SCF}$ / hartree | -533.3482184 |
| $G_{total}$ / hartree | -533.2665912 |

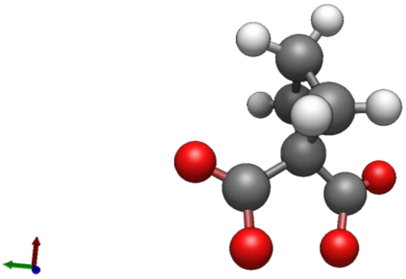

| | | | |
|---|---|---|---|
| O | -0.795071 | 2.306498 | 0.285457 |
| C | -1.264655 | 1.172121 | 0.353907 |
| O | -2.436494 | 0.938907 | 0.822421 |
| O | -2.579876 | -1.094010 | 0.291820 |
| C | -1.427044 | -1.221937 | -0.257744 |
| C | -0.480391 | -0.056518 | -0.060386 |
| C | 0.665981 | -0.347134 | 0.968664 |
| C | 1.681102 | 0.268032 | -0.012668 |
| C | 0.612571 | 0.185917 | -1.119757 |
| O | -1.095617 | -2.237803 | -0.865389 |
| H | 0.556617 | 0.116697 | 1.946807 |
| H | 0.802214 | -1.419964 | 1.092545 |
| H | 1.930067 | 1.294931 | 0.244821 |
| H | 2.599982 | -0.291822 | -0.173092 |
| H | 0.727197 | -0.697051 | -1.745348 |
| H | 0.471145 | 1.064218 | -1.745338 |

## Geometries and oxidation potential for 7

### Neutral intermediate radical $^{2}$**7**

| | |
|---|---|
| $E_{SCF}$ / hartree | -654.94020290 |
| $G_{total}$ / hartree | -654.72759179 |

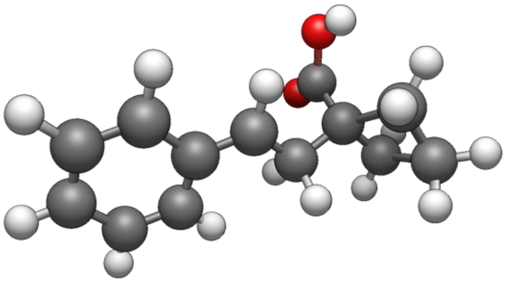

| | | | |
|---|---|---|---|
| O | 3.17921485 | -2.58174190 | -0.86018943 |
| C | 3.13006843 | -2.32757424 | 0.31765726 |
| C | 3.06173208 | -0.91193507 | 0.84636882 |
| C | 4.42742078 | -0.24680640 | 0.63701126 |
| C | 5.53824528 | -0.88570237 | 1.39907643 |
| C | 6.90659444 | -0.62078027 | 1.18639741 |
| C | 7.36552753 | 0.29922908 | 0.21305479 |
| C | 8.71531114 | 0.53099932 | 0.03738317 |
| C | 9.65813798 | -0.13626499 | 0.81483251 |
| C | 9.22999924 | -1.04558170 | 1.78124048 |
| C | 7.88559832 | -1.28401531 | 1.96696268 |
| C | 2.46621238 | -0.64871257 | 2.25467771 |
| C | 1.65862472 | 0.51929046 | 1.65165471 |
| C | 1.91570882 | -0.05947544 | 0.24603492 |
| O | 3.15013843 | -3.33265011 | 1.19879914 |
| H | 4.33333266 | 0.80876587 | 0.92020782 |
| H | 4.64585393 | -0.24397723 | -0.43489959 |
| H | 5.28818288 | -1.57730656 | 2.19599964 |
| H | 6.65174498 | 0.83291694 | -0.40049668 |
| H | 9.04132030 | 1.24068618 | -0.71222217 |
| H | 10.71392677 | 0.04929933 | 0.66961555 |
| H | 9.95642303 | -1.56927890 | 2.38935603 |
| H | 7.55946299 | -1.99228814 | 2.71896867 |
| H | 3.17587431 | -0.42042753 | 3.04821798 |
| H | 1.80476521 | -1.44901317 | 2.58777174 |
| H | 2.14992761 | 1.47886166 | 1.79896442 |
| H | 0.62079847 | 0.60338903 | 1.96374132 |
| H | 2.19930297 | 0.63105815 | -0.54614699 |
| H | 1.09025187 | -0.68008488 | -0.09858429 |
| H | 3.09356759 | -3.00780924 | 2.10313469 |

### Oxidized intermediate cation $^{1}$**8**

| | |
|---|---|
| $E_{SCF}$ / hartree | -654.79224203 |
| $G_{total}$ / hartree | -654.57294830 |

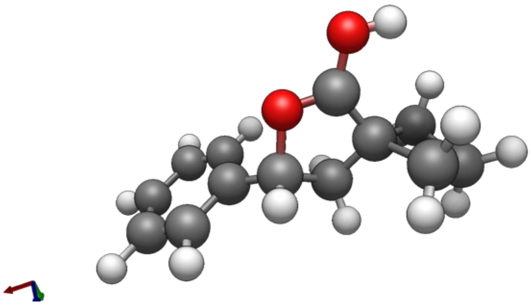

| | | | |
|---|---|---|---|
| O | 4.91495213 | -2.15615619 | 0.70740961 |
| C | 3.68819466 | -1.91805941 | 0.49221980 |
| C | 3.18130137 | -0.66483582 | 1.10025786 |
| C | 4.49697312 | 0.07379460 | 1.34094095 |
| C | 5.50223157 | -1.04999161 | 1.54098685 |
| C | 6.91193250 | -0.80913388 | 1.10692571 |
| C | 7.20954555 | -0.44712323 | -0.20375190 |
| C | 8.52172151 | -0.21231883 | -0.57689214 |
| C | 9.54277041 | -0.32352179 | 0.35892458 |
| C | 9.24927156 | -0.67903997 | 1.66580861 |
| C | 7.93597034 | -0.93009419 | 2.03657016 |
| C | 2.29363743 | -0.88382088 | 2.37205608 |
| C | 1.43439977 | 0.30757000 | 1.90956175 |
| C | 2.01609690 | 0.13852114 | 0.49171394 |
| O | 3.05270327 | -2.79871741 | -0.17336518 |
| H | 4.46276209 | 0.72700866 | 2.20881626 |
| H | 4.75749491 | 0.66705246 | 0.46446422 |
| H | 5.47186600 | -1.44518812 | 2.55406836 |
| H | 6.41819310 | -0.35289315 | -0.93713558 |
| H | 8.74958776 | 0.06015327 | -1.59870996 |
| H | 10.56703144 | -0.13212065 | 0.06731101 |
| H | 10.04106550 | -0.77006272 | 2.39717389 |
| H | 7.70469207 | -1.21575147 | 3.05509337 |
| H | 2.81969829 | -0.81714232 | 3.32098695 |
| H | 1.74797797 | -1.82438403 | 2.32095438 |
| H | 1.75753341 | 1.23966139 | 2.36808947 |
| H | 0.35890032 | 0.20478852 | 2.01830079 |
| H | 2.31140842 | 1.02832284 | -0.05838145 |
| H | 1.37145705 | -0.47208350 | -0.13985736 |
| H | 2.12189955 | -2.57136371 | -0.31595101 |

### 4.1.3. Calculation of natural transition orbitals by TD-DFT

Deviating from the standard procedure, the crystal structure of PTH-O was used as an initial guess geometry for the TD-DFT calculation. After ground state optimization, the first excited singlet state was geometry-optimized and the natural transition orbitals were plotted using the orca_plot tool.

**Geometries for ground and excited state PTH-O**

Ground state $^{1}$**PTH-O**

$E_{SCF}$ / hartree -1221.7587500

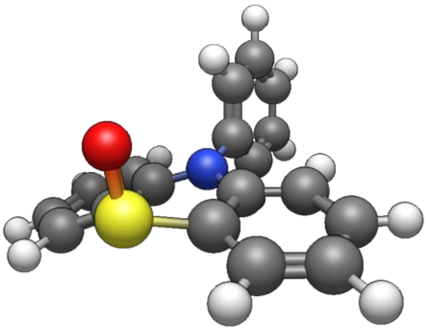

| | | | |
|---|---|---|---|
| O | -2.539695 | 0.519702 | -2.406881 |
| S | -2.892653 | 0.482884 | -0.968122 |
| C | -2.183740 | -0.989978 | -0.293894 |
| C | -3.020328 | -2.094592 | -0.197148 |
| C | -2.516284 | -3.339759 | 0.105469 |
| C | -1.147209 | -3.474272 | 0.288348 |
| C | -0.305551 | -2.385531 | 0.210842 |
| C | -0.811392 | -1.108335 | -0.056975 |
| N | 0.025560 | 0.006714 | -0.057363 |
| C | 1.444423 | -0.197565 | -0.052708 |
| C | 2.132402 | -0.372158 | 1.137707 |
| C | 3.502588 | -0.567571 | 1.118046 |
| C | 4.183701 | -0.588372 | -0.088629 |
| C | 3.492798 | -0.413456 | -1.275863 |
| C | 2.121708 | -0.217742 | -1.259565 |
| C | -0.464217 | 1.303651 | 0.091204 |
| C | -1.809875 | 1.605595 | -0.135283 |
| C | -2.303563 | 2.881099 | 0.107221 |
| C | -1.475105 | 3.888407 | 0.548884 |
| C | -0.127089 | 3.609105 | 0.724353 |
| C | 0.375532 | 2.345027 | 0.502682 |
| H | -4.077228 | -1.960723 | -0.391520 |
| H | -3.170690 | -4.196894 | 0.176360 |
| H | -0.724224 | -4.446454 | 0.504852 |
| H | 0.752247 | -2.522973 | 0.370578 |
| H | 1.589835 | -0.353688 | 2.073778 |
| H | 4.039348 | -0.703690 | 2.047293 |
| H | 5.254499 | -0.741178 | -0.102550 |
| H | 4.021231 | -0.429178 | -2.219487 |
| H | 1.565981 | -0.079398 | -2.177428 |
| H | -3.351811 | 3.074198 | -0.084148 |
| H | -1.863080 | 4.880156 | 0.732895 |
| H | 0.548217 | 4.389657 | 1.049347 |
| H | 1.426103 | 2.158823 | 0.660683 |

Excited state $^{1}$**PTH-O***

$E_{SCF}$ / hartree -1221.6276458

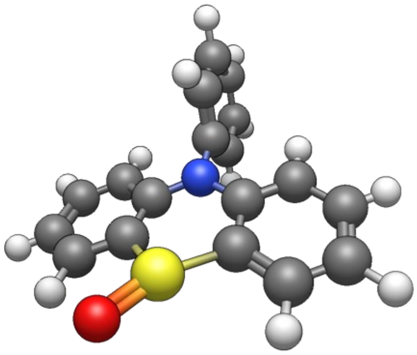

| | | | |
|---|---|---|---|
| O | -4.328093 | 1.334655 | 0.815922 |
| S | -3.144151 | 0.446163 | 0.440394 |
| C | -2.151067 | -1.058283 | -0.173772 |
| C | -2.936058 | -2.176430 | -0.418193 |
| C | -2.369612 | -3.422374 | -0.567772 |
| C | -0.991868 | -3.549519 | -0.432031 |
| C | -0.194573 | -2.450895 | -0.191604 |
| C | -0.762800 | -1.174286 | -0.085817 |
| N | 0.025548 | -0.041823 | 0.088235 |
| C | 1.443925 | -0.224232 | 0.205249 |
| C | 2.024272 | -0.249016 | 1.461051 |
| C | 3.392241 | -0.428275 | 1.583593 |
| C | 4.175848 | -0.585425 | 0.452793 |
| C | 3.590407 | -0.563015 | -0.803165 |
| C | 2.224191 | -0.381848 | -0.928725 |
| C | -0.474217 | 1.260171 | 0.025458 |
| C | -1.842818 | 1.588413 | 0.129456 |
| C | -2.232310 | 2.929523 | 0.060442 |
| C | -1.322249 | 3.942562 | -0.110621 |
| C | 0.025044 | 3.624825 | -0.200694 |
| C | 0.436773 | 2.315728 | -0.132151 |
| H | -4.012119 | -2.047781 | -0.466939 |
| H | -2.985059 | -4.289081 | -0.765593 |
| H | -0.528142 | -4.523565 | -0.515346 |
| H | 0.872124 | -2.583122 | -0.095649 |
| H | 1.400148 | -0.125337 | 2.335870 |
| H | 3.845310 | -0.446513 | 2.565553 |
| H | 5.243997 | -0.725940 | 0.549335 |
| H | 4.199446 | -0.685348 | -1.688657 |
| H | 1.755515 | -0.360855 | -1.903657 |
| H | -3.286244 | 3.142856 | 0.157023 |
| H | -1.655463 | 4.969169 | -0.169152 |
| H | 0.765068 | 4.403347 | -0.329987 |
| H | 1.489211 | 2.097061 | -0.207871 |

**First singlet excited state energies:**

*E* = 0.094737 au 2.578 eV 59.5 kcal/mol 20792.4 cm$^{-1}$

**Molecular orbital contributions:**

75a -> 76a : n= 0.97959422

74a -> 77a : n= 0.01676847

73a -> 78a : n= 0.00101408

**NTO donor orbital** **NTO acceptor orbital**

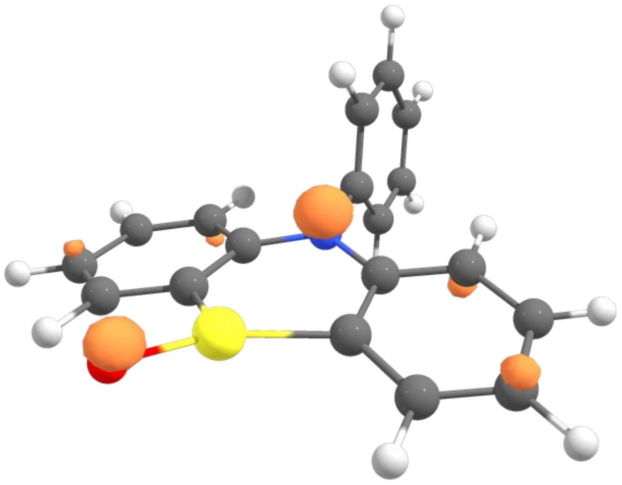

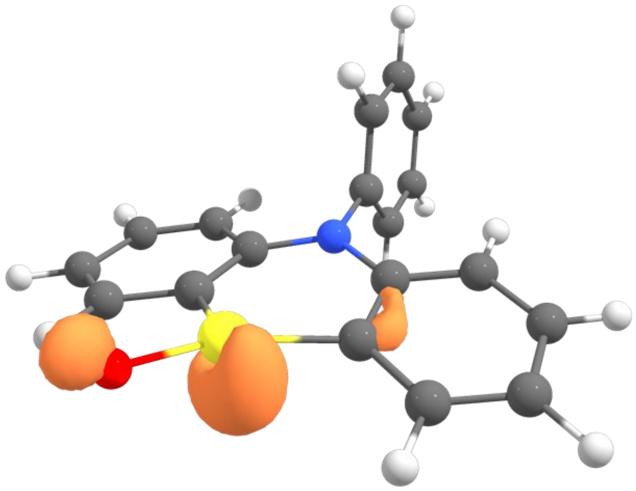

## 4.2. Dynamic vertical triplet energies

Dynamic vertical triplet energies were calculated using an adapted method originally developed by Paton and colleagues.[34] To obtain initial MD structures, geometry optimizations and high precision frequency calculations (at M06-2X/6-31G(d) level of theory)[35] were carried out using Gaussian 16[36] on the Palma II high performance computing cluster of the University of Münster. Based on this result, 25 MD trajectories were generated using the *milo* package developed by ESS and colleagues[37] interfering with Gaussian 16, also on the Palma II cluster. The milo MD simulations were run for 1000 fs at the M06-2X/MIDI![38] level of theory with a step size of 1 fs at a temperature of 298.15 K. Snapshots of the geometry were extracted every 5 fs from every of the 25 simulations, yielding 5000 geometries in total.

For each of the geometries, ORCA single point calculation job scripts (at M06-2X/6-31G(d)) level of theory, D3 dispersion correction, CPCM model for MeCN were automatically generated for the singlet and triplet state and executed in parallel on the local computing cluster of the GLORIUS group on Intel© Xeon® Gold 6240 CPUs.

Vertical singlet-triplet gaps were extracted for every snapshot. The collection of vertical gaps was subsequently plotted in a histogram using *matplotlib*[39] and approximated with a normal distribution with *scikit-learn*[40]. The corresponding cumulative distribution function (CDF) was calculated, and dynamic vertical triplet energies were determined as the energy including 0.8% of the distribution.

### 4.2.1. Optimized geometries for the MD simulation initialization

**2a**

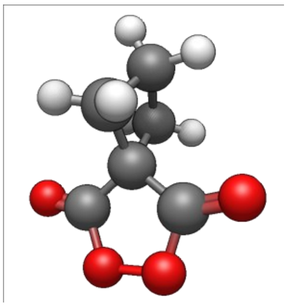

| | | | |
|---|---|---|---|
| O | -0.418261 | 2.257162 | -0.000001 |
| C | 0.204543 | 1.239641 | -0.000001 |
| C | -0.251454 | -0.182148 | 0.000000 |
| C | -1.327698 | -0.510003 | -1.075707 |
| C | -2.416585 | -0.321620 | -0.000000 |
| C | -1.327699 | -0.510000 | 1.075707 |
| C | 1.026419 | -0.953495 | 0.000003 |
| O | 2.083665 | -0.071492 | 0.000000 |
| O | 1.581146 | 1.266511 | -0.000000 |
| O | 1.218269 | -2.130126 | -0.000001 |
| H | -1.343285 | 0.140948 | -1.951477 |
| H | -1.215699 | -1.554018 | -1.379249 |
| H | -2.796650 | 0.701537 | -0.000002 |
| H | -3.249083 | -1.026094 | 0.000001 |
| H | -1.215700 | -1.554013 | 1.379254 |
| H | -1.343286 | 0.140954 | 1.951476 |

**2b**

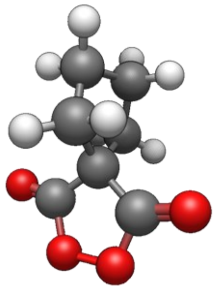

| | | | |
|---|---|---|---|
| O | -0.651185 | -2.337323 | 0.113908 |
| C | -0.877894 | -1.168012 | 0.056544 |
| C | 0.061977 | -0.000015 | -0.000010 |
| C | 1.006669 | 0.055329 | 1.241361 |
| C | 2.386779 | 0.381324 | 0.661688 |
| C | 2.386804 | -0.381349 | -0.661650 |
| C | 1.006696 | -0.055398 | -1.241357 |
| C | -0.877834 | 1.168031 | -0.056566 |
| O | -2.176118 | 0.713468 | -0.027716 |
| O | -2.176155 | -0.713379 | 0.027750 |
| O | -0.651066 | 2.337329 | -0.113961 |
| H | 1.016898 | -0.938394 | 1.700859 |
| H | 0.659953 | 0.777889 | 1.983732 |
| H | 2.466407 | 1.458026 | 0.474114 |
| H | 3.195171 | 0.090020 | 1.336737 |
| H | 2.466460 | -1.458049 | -0.474071 |
| H | 3.195202 | -0.090024 | -1.336680 |
| H | 0.660005 | -0.777998 | -1.983701 |
| H | 1.016920 | 0.938303 | -1.700901 |

**2c**

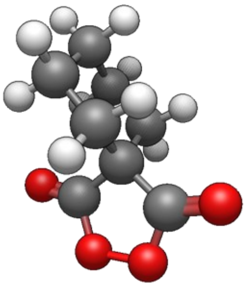

| | | | |
|---|---|---|---|
| O | 1.773455 | -2.086050 | 0.000004 |
| C | 1.531742 | -0.918765 | 0.000001 |
| C | 0.205724 | -0.208306 | 0.000003 |
| C | -0.592156 | -0.577161 | -1.272523 |
| C | -1.995466 | 0.029973 | -1.259614 |
| C | -2.761103 | -0.378757 | -0.000008 |
| C | -1.995474 | 0.029962 | 1.259607 |
| C | -0.592163 | -0.577170 | 1.272521 |
| C | 0.622295 | 1.236976 | 0.000007 |
| O | 1.996920 | 1.316945 | -0.000004 |
| O | 2.552234 | 0.002471 | -0.000007 |
| O | -0.023853 | 2.241027 | 0.000015 |
| H | -0.035190 | -0.264042 | -2.162464 |
| H | -0.648189 | -1.672813 | -1.292224 |
| H | -2.528986 | -0.297740 | -2.157598 |
| H | -1.921374 | 1.122335 | -1.306095 |
| H | -3.756147 | 0.076883 | -0.000009 |
| H | -2.906769 | -1.467961 | -0.000013 |
| H | -2.529000 | -0.297760 | 2.157586 |
| H | -1.921384 | 1.122323 | 1.306100 |
| H | -0.648195 | -1.672823 | 1.292213 |
| H | -0.035204 | -0.264057 | 2.162467 |

**2g**

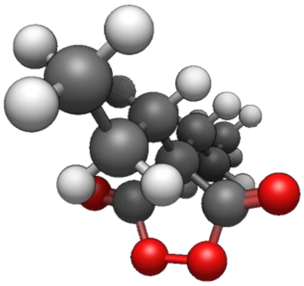

| | | | |
|---|---|---|---|
| O | -0.000003 | 0.464630 | 2.341383 |
| C | 0.000004 | 0.690006 | 1.170526 |
| O | 0.000026 | 1.987887 | 0.714597 |
| O | 0.000023 | 1.987902 | -0.714559 |
| C | -0.000000 | 0.690030 | -1.170515 |
| C | -0.000005 | -0.255633 | -0.000004 |
| C | -1.276478 | -1.115454 | -0.000012 |
| C | -2.573601 | -0.306623 | 0.000000 |
| C | -3.800754 | -1.214526 | -0.000007 |
| C | 1.276466 | -1.115460 | -0.000013 |
| C | 2.573594 | -0.306636 | -0.000003 |
| C | 3.800741 | -1.214546 | -0.000011 |
| O | -0.000011 | 0.464678 | -2.341377 |
| H | -1.234460 | -1.759976 | -0.886153 |
| H | -1.234457 | -1.759998 | 0.886113 |
| H | -2.600713 | 0.346501 | -0.881725 |
| H | -2.600709 | 0.346481 | 0.881740 |
| H | -4.723962 | -0.629827 | -0.000001 |
| H | -3.809359 | -1.858489 | -0.885135 |
| H | -3.809357 | -1.858505 | 0.885108 |
| H | 1.234444 | -1.759984 | -0.886153 |
| H | 1.234442 | -1.760001 | 0.886114 |
| H | 2.600707 | 0.346469 | 0.881736 |
| H | 2.600709 | 0.346488 | -0.881728 |
| H | 3.809339 | -1.858528 | 0.885103 |
| H | 3.809344 | -1.858506 | -0.885140 |
| H | 4.723953 | -0.629852 | -0.000002 |

### 4.2.2 Vertical triplet energy distributions and DvTEs

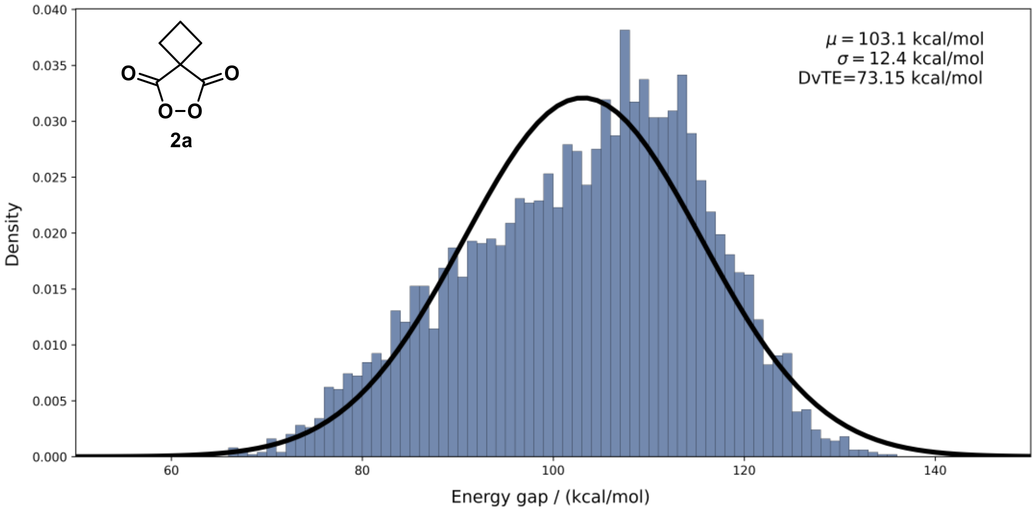


**Figure S26:** Histogram of vertical triplet energies obtained from different geometries in MD simulation of **2a**. Distriubtion was approximated using a normal distribution and DvTE was determined to include 0.8% of the distribution.

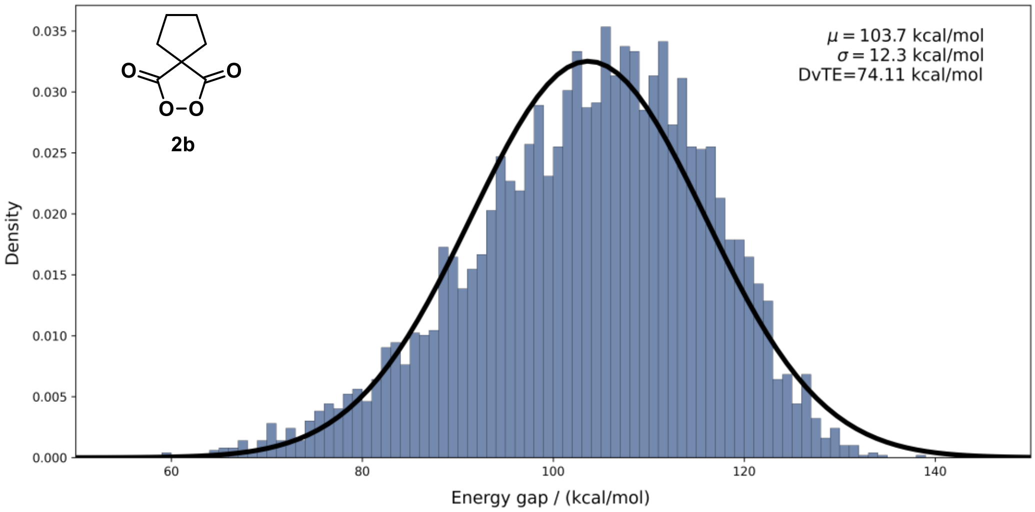


**Figure S27:** Histogram of vertical triplet energies obtained from different geometries in MD simulation of **2b**. Distriubtion was approximated using a normal distribution and DvTE was determined to include 0.8% of the distribution.

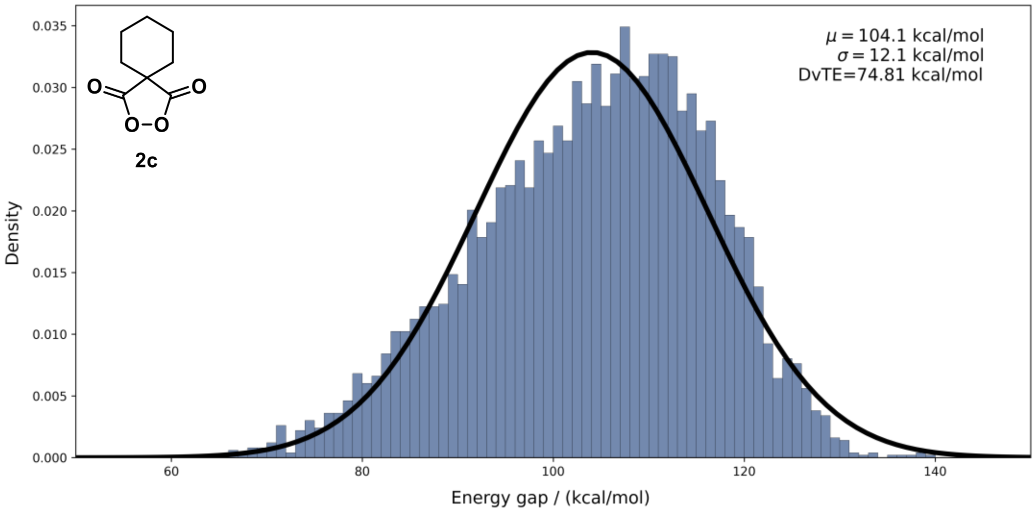


**Figure S28:** Histogram of vertical triplet energies obtained from different geometries in MD simulation of **2c**. Distriubtion was approximated using a normal distribution and DvTE was determined to include 0.8% of the distribution.

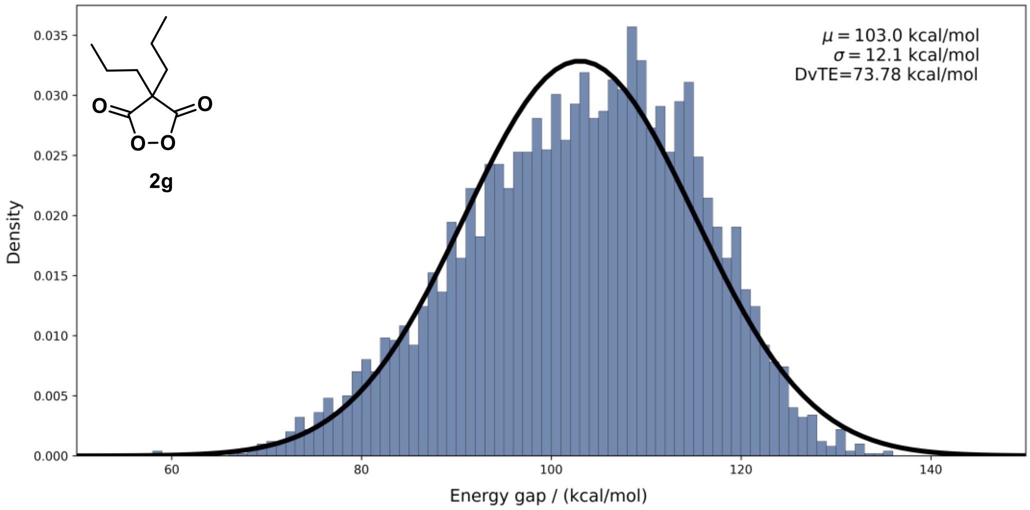


**Figure S29:** Histogram of vertical triplet energies obtained from different geometries in MD simulation of **2g**. Distriubtion was approximated using a normal distribution and DvTE was determined to include 0.8% of the distribution.

# 5. X-RAY CRYSTALLOGRAPHY

## 5.1. X-Ray Diffraction Data

Deposition numbers 2419998 (for 3a), 2419999 (for 3g), 2420000 (for 3h), 2420001 (for 3i), 2420002 (for 3j), 2420003 (for 3m), 2420004 (for 3o), 2420005 (for PHT-O2), 2420006 (for PHT-O) and 2420007 (for 2c) contain the supplementary crystallographic data for this paper. These data are provided free of charge by the joint Cambridge Crystallographic Data Centre and Fachinformationszentrum Karlsruhe Access Structures service.

**X-Ray diffraction:** Data sets for compounds **3a**, **3g**, **3h**, **3i**, **3j**, **3m**, **3o**, **PTH-O2**, **PTH-O** and **2c** were collected with a Bruker D8 Venture Photon III Diffractometer. Programs used: data collection: *APEX4* Version 2021.4-0 [41]; cell refinement: *SAINT* Version 8.40B (Bruker AXS Inc., **2021**); data reduction: *SAINT* Version 8.40B (Bruker AXS Inc., **2021**); absorption correction, *SADABS* Version 2016/2 (Bruker AXS Inc., **2021**); structure solution *SHELXT*-Version 2018-3[42]; structure refinement *SHELXL*- Version 2018-3[43] and graphics, *XP* [44](Version 5.1, Bruker AXS Inc., Madison, Wisconsin, USA, **1998**). *R*-values are given for observed reflections, and $wR^2$ values are given for all reflections.

**X-ray crystal structure analysis of 3a (glo10634):**

A colorless, prism-like specimen of $C_{13}H_{14}O_2$, approximate dimensions 0.090 mm x 0.124 mm x 0.154 mm, was used for the X-ray crystallographic analysis. The X-ray intensity data were measured on a single crystal diffractometer Bruker D8 Venture Photon III system equipped with a micro focus tube Cu ImS (CuKα, λ = 1.54178 Å) and a MX mirror monochromator. A total of 1766 frames were collected. The total exposure time was 14.47 hours. The frames were integrated with the Bruker SAINT software package using a wide-frame algorithm. The integration of the data using a monoclinic unit cell yielded a total of 18838 reflections to a maximum θ angle of 66.71° (0.84 Å resolution), of which 1808 were independent (average redundancy 10.419, completeness = 98.9%, $R_{int}$ = 3.72%, $R_{sig}$ = 1.87%) and 1651 (91.32%) were greater than $2\sigma(F^2)$. The final cell constants of a = 7.56120(10) Å, b = 6.02450(10) Å, c = 22.9720(4) Å, β = 98.1080(10)°, volume = 1035.97(3) Å$^3$, are based upon the refinement of the XYZ-centroids of 9968 reflections above 20 σ(I) with 13.21° < 2θ < 133.3°. Data were corrected for absorption effects using the multi-scan method (SADABS). The ratio of minimum to maximum apparent transmission was 0.900. The calculated minimum and maximum transmission coefficients (based on crystal size) are 0.9010 and 0.9410. The structure was solved and refined using the Bruker SHELXTL Software Package, using the space group $P2_1/c$, with Z = 4 for the formula unit, $C_{13}H_{14}O_2$. The final anisotropic full-matrix least-squares refinement on $F^2$ with 136 variables converged at R1 = 3.43%, for the observed data and wR2 = 8.85% for all data. The goodness-of-fit was 1.061. The largest peak in the final difference electron density synthesis was 0.252 e$^-$/Å$^3$ and the largest hole was -0.184 e$^-$/Å$^3$ with an RMS deviation of 0.036 e$^-$/Å$^3$. On the basis of the final model, the calculated density was 1.297 g/cm$^3$ and F(000), 432 e$^-$. CCDC Nr.: 2419998.

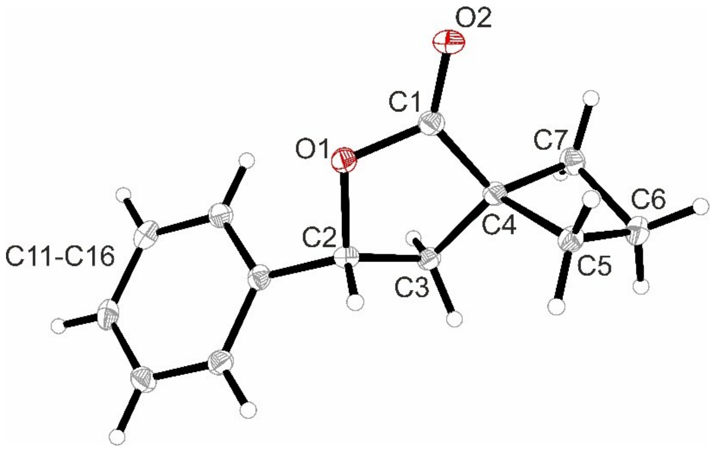


**Figure S30**: Crystal structure of compound **3a**. Thermal ellipsoids are shown at 50% probability.

**X-ray crystal structure analysis of 3g (glo10647):**

A colorless, prism-like specimen of $C_{13}H_{13}BrO_2$, approximate dimensions 0.064 mm x 0.068 mm x 0.114 mm, was used for the X-ray crystallographic analysis. The X-ray intensity data were measured on a single crystal diffractometer Bruker D8 Venture Photon III system equipped with a micro focus tube Mo ImS (MoKα, λ = 0.71073 Å) and a MX mirror monochromator. A total of 793 frames were collected. The total exposure time was 5.51 hours. The frames were integrated with the Bruker SAINT software package using a narrow-frame algorithm. The integration of the data using a monoclinic unit cell yielded a total of 21499 reflections to a maximum θ angle of 27.54° (0.77 Å resolution), of which 2674 were independent (average redundancy 8.040, completeness = 99.8%, $R_{int}$ = 5.94%, $R_{sig}$ = 3.02%) and 2257 (84.41%) were greater than 2σ($F^2$). The final cell constants of a = 8.1473(4) Å, b = 6.1214(3) Å, c = 23.5845(15) Å, β = 98.017(2)°, volume = 1164.73(11) $Å^3$, are based upon the refinement of the XYZ-centroids of 4343 reflections above 20 σ(I) with 6.747° < 2θ < 54.34°. Data were corrected for absorption effects using the multi-scan method (SADABS). The ratio of minimum to maximum apparent transmission was 0.863. The calculated minimum and maximum transmission coefficients (based on crystal size) are 0.6900 and 0.8070. The structure was solved and refined using the Bruker SHELXTL Software Package, using the space group $P2_1/n$, with Z = 4 for the formula unit, $C_{13}H_{13}BrO_2$. The final anisotropic full-matrix least-squares refinement on $F^2$ with 145 variables converged at R1 = 2.69%, for the observed data and wR2 = 5.97% for all data. The goodness-of-fit was 1.062. The largest peak in the final difference electron density synthesis was 0.422 $e^-/Å^3$ and the largest hole was -0.320 $e^-/Å^3$ with an RMS deviation of 0.072 $e^-/Å^3$. On the basis of the final model, the calculated density was 1.603 g/cm$^3$ and F(000), 568 $e^-$. CCDC Nr.: 2419999.

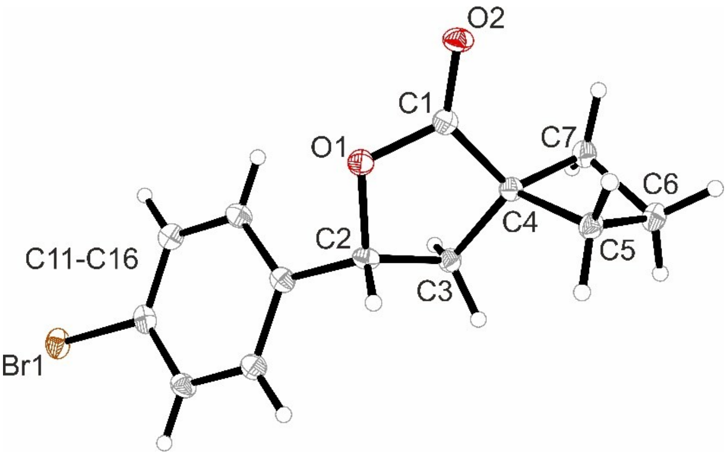


**Figure S31** Crystal structure of compound **3g**. Thermal ellipsoids are shown at 50% probability.

**X-ray crystal structure analysis of 3h (glo10646):**

A colorless, prism-like specimen of $C_{13}H_{13}ClO_2$, approximate dimensions 0.049 mm x 0.065 mm x 0.092 mm, was used for the X-ray crystallographic analysis. The X-ray intensity data were measured on a single crystal diffractometer Bruker D8 Venture Photon III system equipped with a micro focus tube Cu ImS (CuKα, λ = 1.54178 Å) and a MX mirror monochromator. A total of 2317 frames were collected. The total exposure time was 20.32 hours. The frames were integrated with the Bruker SAINT software package using a wide-frame algorithm. The integration of the data using a monoclinic unit cell yielded a total of 21377 reflections to a maximum θ angle of 68.29° (0.83 Å resolution), of which 2082 were independent (average redundancy 10.268, completeness = 99.5%, $R_{int}$ = 5.38%, $R_{sig}$ = 2.53%) and 1838 (88.28%) were greater than $2\sigma(F^2)$. The final cell constants of a = 8.0433(3) Å, b = 6.0667(2) Å, c = 23.5879(8) Å, β = 98.078(2)°, volume = 1139.58(7) $Å^3$, are based upon the refinement of the XYZ-centroids of 9950 reflections above 20 σ(I) with 11.22° < 2θ < 136.6°. Data were corrected for absorption effects using the multi-scan method (SADABS). The ratio of minimum to maximum apparent transmission was 0.881. The calculated minimum and maximum transmission coefficients (based on crystal size) are 0.7820 and 0.8740. The structure was solved and refined using the Bruker SHELXTL Software Package, using the space group $P2_1/n$, with Z = 4 for the formula unit, $C_{13}H_{13}ClO_2$. The final anisotropic full-matrix least-squares refinement on $F^2$ with 145 variables converged at R1 = 3.06%, for the observed data and wR2 = 7.94% for all data. The goodness-of-fit was 1.060. The largest peak in the final difference electron density synthesis was 0.270 $e^-/Å^3$ and the largest hole was -0.208 $e^-/Å^3$ with an RMS deviation of 0.041 $e^-/Å^3$. On the basis of the final model, the calculated density was 1.380 $g/cm^3$ and F(000), 496 $e^-$. CCDC Nr.: 2420000.

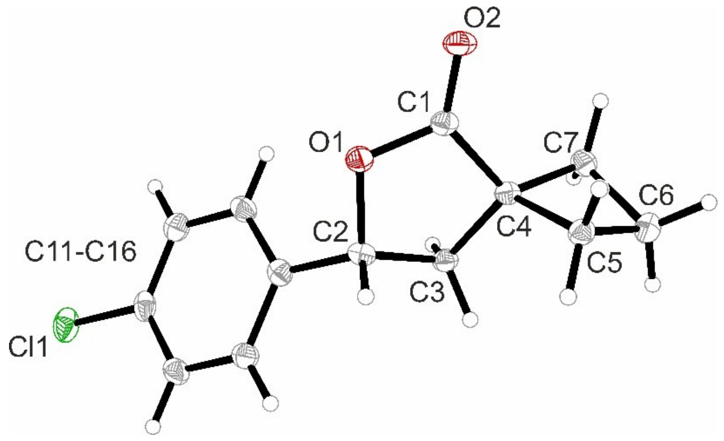


**Figure S32:** Crystal structure of compound **3h**. Thermal ellipsoids are shown at 50% probability.

**X-ray crystal structure analysis of 3i (glo10650):**

A colorless, prism-like specimen of $C_{14}H_{13}F_3O_2$, approximate dimensions 0.110 mm x 0.127 mm x 0.145 mm, was used for the X-ray crystallographic analysis. The X-ray intensity data were measured on a single crystal diffractometer Bruker D8 Venture Photon III system equipped with a micro focus tube Cu ImS (CuKα, λ = 1.54178 Å) and a MX mirror monochromator. A total of 1387 frames were collected. The total exposure time was 17.27 hours. The frames were integrated with the Bruker SAINT software package using a wide-frame algorithm. The integration of the data using an orthorhombic unit cell yielded a total of 12564 reflections to a maximum θ angle of 68.20° (0.83 Å resolution), of which 2177 were independent (average redundancy 5.771, completeness = 98.1%, $R_{int}$ = 2.76%, $R_{sig}$ = 1.97%) and 2142 (98.39%) were greater than 2σ($F^2$). The final cell constants of a = 6.0954(2) Å, b = 9.2480(2) Å, c = 21.5428(5) Å, volume = 1214.37(6) Å$^3$, are based upon the refinement of the XYZ-centroids of 9932 reflections above 20 σ(I) with 12.61° < 2θ < 136.3°. Data were corrected for absorption effects using the multi-scan method (SADABS). The ratio of minimum to maximum apparent transmission was 0.899. The calculated minimum and maximum transmission coefficients (based on crystal size) are 0.8560 and 0.8880. The structure was solved and refined using the Bruker SHELXTL Software Package, using the space group $P2_12_12_1$, with Z = 4 for the formula unit, $C_{14}H_{13}F_3O_2$. The final anisotropic full-matrix least-squares refinement on $F^2$ with 173 variables converged at R1 = 2.54%, for the observed data and wR2 = 6.31% for all data. The goodness-of-fit was 1.048. The largest peak in the final difference electron density synthesis was 0.159 e$^-$/Å$^3$ and the largest hole was -0.131 e$^-$/Å$^3$ with an RMS deviation of 0.031 e$^-$/Å$^3$. On the basis of the final model, the calculated density was 1.478 g/cm$^3$ and F(000), 560 e$^-$. CCDC Nr.: 2420001.

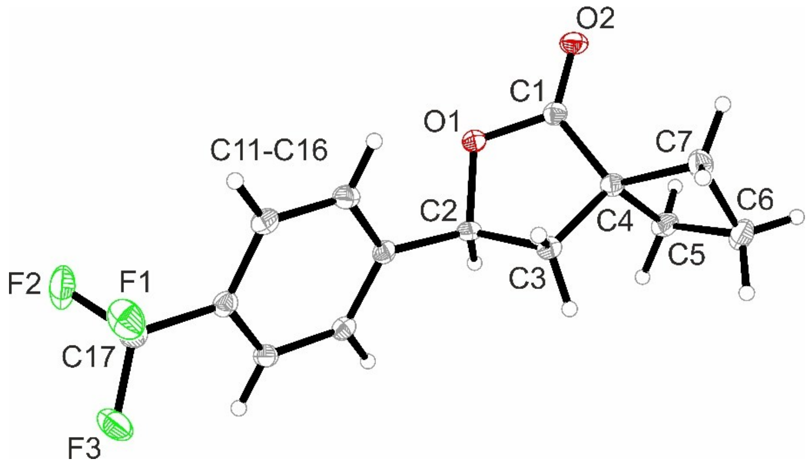


**Figure S33:** Crystal structure of compound **3i**. Thermal ellipsoids are shown at 50% probability.

**X-ray crystal structure analysis of 3j (glo10755):**

A colourless, needle-shaped crystal was mounted on a goniometer. Data for glo10755 were collected from a single crystal in 23.86 hours at 100(2) K on a Bruker D8 VENTURE KAPPA diffractometer with a microfocus sealed tube using a multilayer mirror as monochromator and a Bruker PHOTON III CPAD detector. The diffractometer was equipped with an Oxford Cryostream 700 low temperature device and used Cu $K_{\alpha}$ radiation ($\lambda$ = 1.54178 Å). All data were integrated with SAINT V8.41 yielding 10737 reflections of which 2294 where independent and 65.3 % were greater than $2\sigma(F^2)$. A Multi-Scan absorption correction using SADABS 2016/2 was applied. The structure was solved by Intrinsic Phasing methods with SHELXT 2018/2 and refined by full-matrix least-squares methods against $F^2$ using SHELXL-2019/2. All non-hydrogen atoms were refined with anisotropic displacement parameters. All hydrogen atoms were refined isotropic on calculated positions using a riding model with their $U_{iso}$ values constrained to 1.5 times the $U_{eq}$ of their pivot atoms for terminal sp$^3$ carbon atoms and 1.2 times for all other carbon atoms. CCDC Nr.: 2420002.

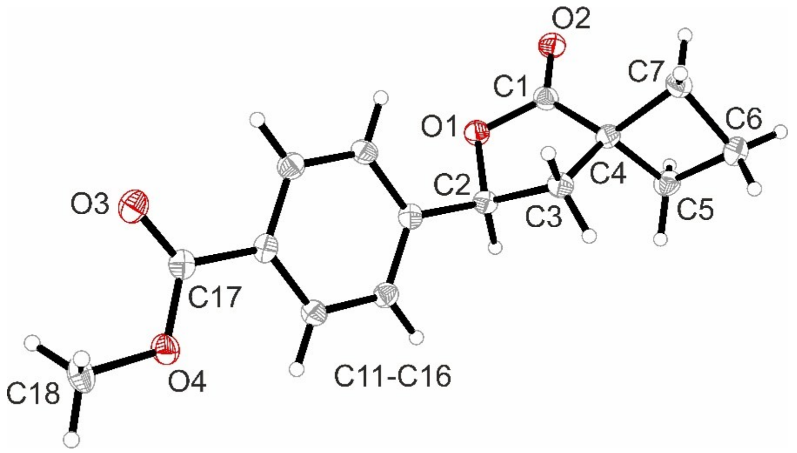


**Figure S34:** Crystal structure of compound **3j**. Thermal ellipsoids are shown at 50% probability.

**X-ray crystal structure analysis of 3m (glo10648):**

A colorless, prism-like specimen of $C_{14}H_{16}O_2$, approximate dimensions 0.049 mm x 0.106 mm x 0.138 mm, was used for the X-ray crystallographic analysis. The X-ray intensity data were measured on a single crystal

diffractometer Bruker D8 Venture Photon III system equipped with a micro focus tube Cu ImS (CuKα, λ = 1.54178 Å) and a MX mirror monochromator. A total of 1487 frames were collected. The total exposure time was 18.23 hours. The frames were integrated with the Bruker SAINT software package using a wide-frame algorithm. The integration of the data using a monoclinic unit cell yielded a total of 9969 reflections to a maximum θ angle of 66.67° (0.84 Å resolution), of which 1981 were independent (average redundancy 5.032, completeness = 98.9%, $R_{int}$ = 3.39%, $R_{sig}$ = 2.50%) and 1918 (96.82%) were greater than 2σ($F^2$). The final cell constants of a = 6.5929(2) Å, b = 8.8985(3) Å, c = 9.7330(3) Å, β = 95.9620(10)°, volume = 567.92(3) $Å^3$, are based upon the refinement of the XYZ-centroids of 7353 reflections above 20 σ(I) with 9.135° < 2θ < 133.1°. Data were corrected for absorption effects using the multi-scan method (SADABS). The ratio of minimum to maximum apparent transmission was 0.874. The calculated minimum and maximum transmission coefficients (based on crystal size) are 0.9140 and 0.9680. The structure was solved and refined using the Bruker SHELXTL Software Package, using the space group $P2_1$, with Z = 2 for the formula unit, $C_{14}H_{16}O_2$. The final anisotropic full-matrix least-squares refinement on $F^2$ with 146 variables converged at R1 = 2.76%, for the observed data and wR2 = 7.00% for all data. The goodness-of-fit was 1.058. The largest peak in the final difference electron density synthesis was 0.161 $e^-/Å^3$ and the largest hole was -0.142 $e^-/Å^3$ with an RMS deviation of 0.030 $e^-/Å^3$. On the basis of the final model, the calculated density was 1.265 g/cm$^3$ and F(000), 232 $e^-$. CCDC Nr.: 2420003.

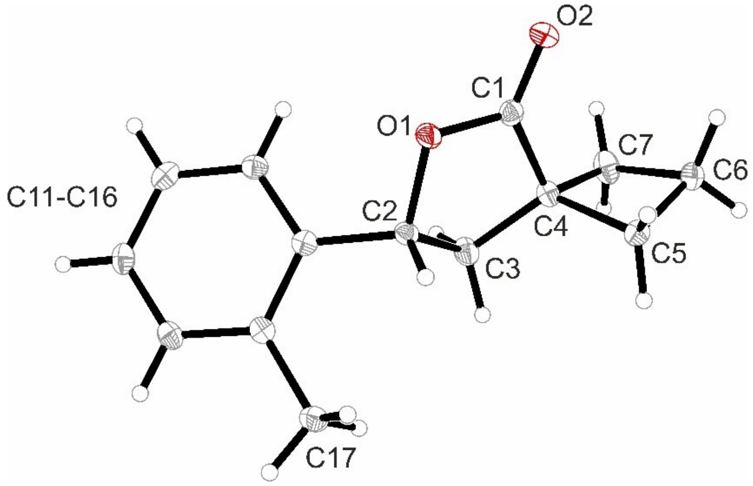


**Figure S35:** Crystal structure of compound **3m**. Thermal ellipsoids are shown at 50% probability.

**X-ray crystal structure analysis of 3o (glo10641):**

A colorless, prism-like specimen of $C_{13}H_{12}Cl_2O_2$, approximate dimensions 0.075 mm x 0.131 mm x 0.141 mm, was used for the X-ray crystallographic analysis. The X-ray intensity data were measured on a single crystal diffractometer Bruker D8 Venture Photon III system equipped with a micro focus tube Mo ImS (MoKα, λ = 0.71073 Å) and a MX mirror monochromator. A total of 1182 frames were collected. The total exposure time was 6.57 hours. The frames were integrated with the Bruker SAINT software package using a narrow-frame algorithm. The integration of the data using a monoclinic unit cell yielded a total of 26348 reflections to a maximum θ angle of 27.54° (0.77 Å resolution), of which 2650 were independent (average redundancy 9.943, completeness = 99.4%, $R_{int}$ = 3.42%, $R_{sig}$ = 1.53%) and 2412 (91.02%) were greater than 2σ($F^2$). The final cell constants of a = 5.53600(10) Å, b = 19.8216(4) Å, c = 10.8492(2) Å, β = 104.5480(10)°, volume

= 1152.34(4) Å$^3$, are based upon the refinement of the XYZ-centroids of 9921 reflections above 20 σ(I) with 4.389° < 2θ < 55.01°. Data were corrected for absorption effects using the multi-scan method (SADABS). The ratio of minimum to maximum apparent transmission was 0.959. The calculated minimum and maximum transmission coefficients (based on crystal size) are 0.9270 and 0.9600. The structure was solved and refined using the Bruker SHELXTL Software Package, using the space group $P2_1/n$, with Z = 4 for the formula unit, $C_{13}H_{12}Cl_2O_2$. The final anisotropic full-matrix least-squares refinement on $F^2$ with 154 variables converged at R1 = 2.65%, for the observed data and wR2 = 6.72% for all data. The goodness-of-fit was 1.076. The largest peak in the final difference electron density synthesis was 0.362 e$^-$/Å$^3$ and the largest hole was -0.199 e$^-$/Å$^3$ with an RMS deviation of 0.050 e$^-$/Å$^3$. On the basis of the final model, the calculated density was 1.563 g/cm$^3$ and F(000), 560 e$^-$. CCDC Nr.: 2420004.

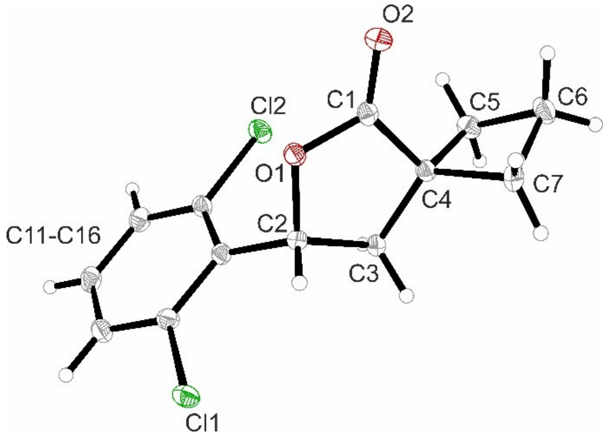


**Figure S36:** Crystal structure of compound **3o**. Thermal ellipsoids are shown at 50% probability.

**X-ray crystal structure analysis of PTH-$O_2$ (glo10740):**

A colourless, prism-shaped crystal was mounted on a loop. Data for glo10740 were collected from a single crystal in 16.37 hours at 100(2) K on a Bruker D8 VENTURE KAPPA diffractometer with a microfocus sealed tube using a multilayer mirror as monochromator and a Bruker PHOTON III CPAD detector. The diffractometer was equipped with an Oxford Cryostream 1000 low temperature device and used Cu $K_\alpha$ radiation (λ = 1.54178 Å). All data were integrated with SAINT V8.41 yielding 49262 reflections of which 5897 where independent and 98.6 % were greater than 2σ($F^2$).[1] A Multi-Scan absorption correction using SADABS 2016/2 was applied.[2] The structure was solved by Intrinsic Phasing methods with SHELXT 2018/2 and refined by full-matrix least-squares methods against $F^2$ using SHELXL-2019/2.[3,4] All non-hydrogen atoms were refined with anisotropic displacement parameters. All hydrogen atoms were refined isotropic on calculated positions using a riding model with their $U_{iso}$ values constrained to 1.5 times the $U_{eq}$ of their pivot atoms for terminal sp$^3$ carbon atoms and 1.2 times for all other carbon atoms. CCDC Nr.: 2420005.

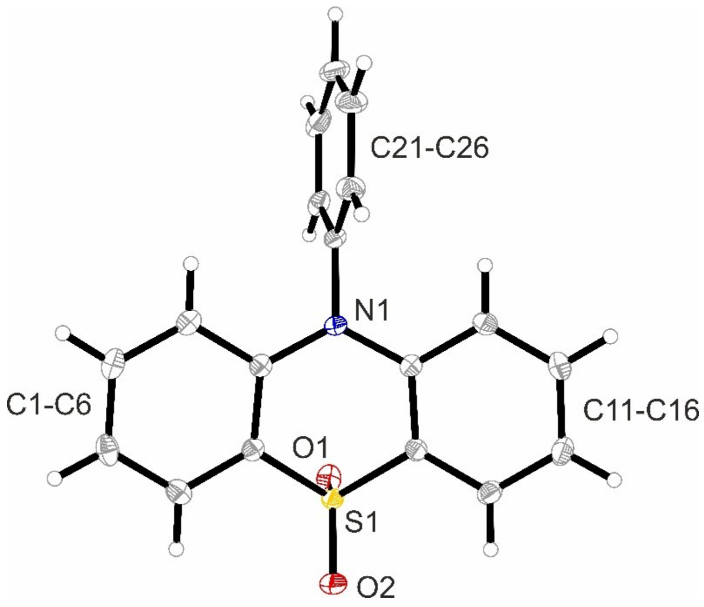


**Figure S37:** Crystal structure of compound **PTH-O2**. Only one molecule (molecule named with suffix "A") of two found in the asymmetric unit is shown. Thermal ellipsoids are shown at 50% probability.

**X-ray crystal structure analysis of PTH-O (glo10661):**

A light yellow, prism-like specimen of $C_{18}H_{13}NOS$, approximate dimensions 0.020 mm x 0.060 mm x 0.070 mm, was used for the X-ray crystallographic analysis. The X-ray intensity data were measured on a single crystal diffractometer Bruker D8 Venture Photon III system equipped with a micro focus tube Cu ImS (CuKα, λ = 1.54178 Å) and a MX mirror monochromator. A total of 1300 frames were collected. The total exposure time was 16.27 hours. The frames were integrated with the Bruker SAINT software package using a wide-frame algorithm. The integration of the data using a triclinic unit cell yielded a total of 11529 reflections to a maximum θ angle of 66.73° (0.84 Å resolution), of which 2441 were independent (average redundancy 4.723, completeness = 98.6%, $R_{int}$ = 4.85%, $R_{sig}$ = 3.75%) and 2136 (87.51%) were greater than 2σ($F^2$). The final cell constants of a = 7.8596(3) Å, b = 7.9135(4) Å, c = 12.6923(6) Å, α = 101.111(2)°, β = 97.394(2)°, γ = 112.983(2)°, volume = 694.73(6) $Å^3$, are based upon the refinement of the XYZ-centroids of 5582 reflections above 20 σ(I) with 7.286° < 2θ < 133.0°. Data were corrected for absorption effects using the multi-scan method (SADABS). The ratio of minimum to maximum apparent transmission was 0.854. The calculated minimum and maximum transmission coefficients (based on crystal size) are 0.8710 and 0.9600. The structure was solved and refined using the Bruker SHELXTL Software Package, using the space group *P*-1, with Z = 2 for the formula unit, $C_{18}H_{13}NOS$. The final anisotropic full-matrix least-squares refinement on $F^2$ with 190 variables converged at R1 = 3.61%, for the observed data and wR2 = 9.31% for all data. The goodness-of-fit was 1.056. The largest peak in the final difference electron density synthesis was 0.318 $e^-/Å^3$ and the largest hole was -0.440 $e^-/Å^3$ with an RMS deviation of 0.053 $e^-/Å^3$. On the basis of the final model, the calculated density was 1.393 g/$cm^3$ and F(000), 304 $e^-$. CCDC Nr.: 2420006.

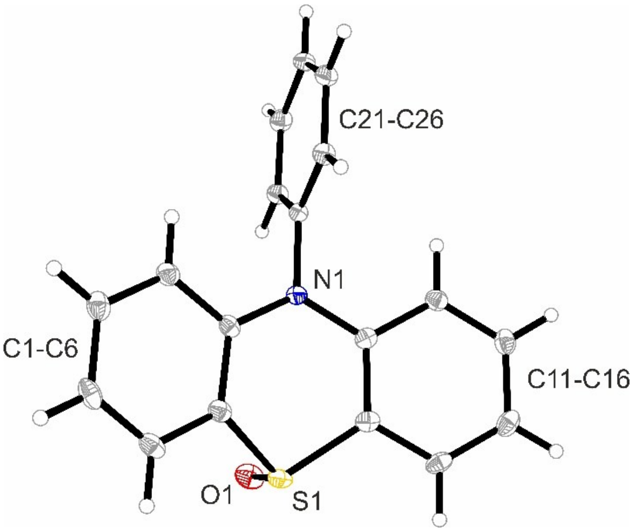


**Figure S38:** Crystal structure of compound **PTH-O**. Thermal ellipsoids are shown at 50% probability.

**X-ray crystal structure analysis of 2d (glo10644):**

A colorless, prism-like specimen of $C_7H_8O_5$, approximate dimensions 0.120 mm x 0.133 mm x 0.165 mm, was used for the X-ray crystallographic analysis. The X-ray intensity data were measured on a single crystal diffractometer Bruker D8 Venture Photon III system equipped with a micro focus tube Cu ImS (CuKα, λ = 1.54178 Å) and a MX mirror monochromator. A total of 2338 frames were collected. The total exposure time was 17.08 hours. The frames were integrated with the Bruker SAINT software package using a wide-frame algorithm. The integration of the data using a monoclinic unit cell yielded a total of 12774 reflections to a maximum θ angle of 66.68° (0.84 Å resolution), of which 1346 were independent (average redundancy 9.490, completeness = 95.9%, $R_{int}$ = 3.84%, $R_{sig}$ = 2.24%) and 1292 (95.99%) were greater than 2σ($F^2$). The final cell constants of a = 9.6011(4) Å, b = 6.5187(3) Å, c = 11.9568(5) Å, β = 104.8580(10)°, volume = 723.32(5) Å$^3$, are based upon the refinement of the XYZ-centroids of 9769 reflections above 20 σ(I) with 7.649° < 2θ < 133.3°. Data were corrected for absorption effects using the multi-scan method (SADABS). The ratio of minimum to maximum apparent transmission was 0.878. The calculated minimum and maximum transmission coefficients (based on crystal size) are 0.8280 and 0.8700. The structure was solved and refined using the Bruker SHELXTL Software Package, using the space group $P2_1/m$, with Z = 4 for the formula unit, $C_7H_8O_5$. The final anisotropic full-matrix least-squares refinement on $F^2$ with 133 variables converged at R1 = 3.25%, for the observed data and wR2 = 7.86% for all data. The goodness-of-fit was 1.083. The largest peak in the final difference electron density synthesis was 0.255 $e^-$/Å$^3$ and the largest hole was -0.179 $e^-$/Å$^3$ with an RMS deviation of 0.035 $e^-$/Å$^3$. On the basis of the final model, the calculated density was 1.581 g/cm$^3$ and F(000), 360 $e^-$. CCDC Nr.: 2420007.

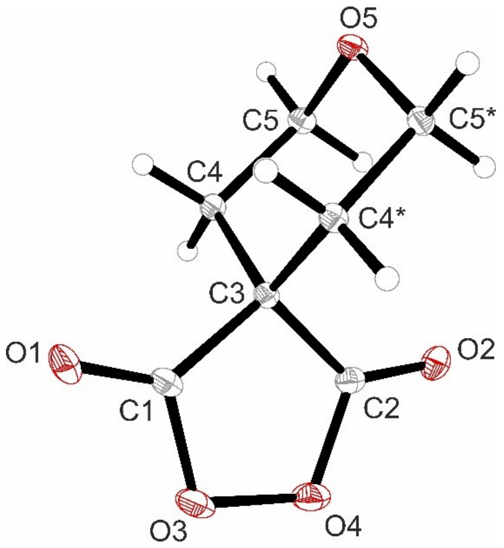


**Figure S39:** Crystal structure of compound **2d**. Only one molecule (molecule named with suffix “A”) of two found in the asymmetric unit is shown. Thermal ellipsoids are shown at 30% probability.

## 6. ACKNOWLEDGEMENTS

All calculations in Gaussian16 that were made for this publication were performed on the HPC cluster PALMA II of the University of Münster, subsidized by the DFG (INST 211/667-1).

# 7. SPECTROSCOPIC DATA

**$^{1}$H NMR** (599 MHz, $CDCl_3$) of **3a** see procedure

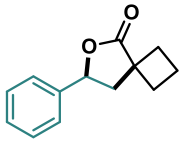


**3a**

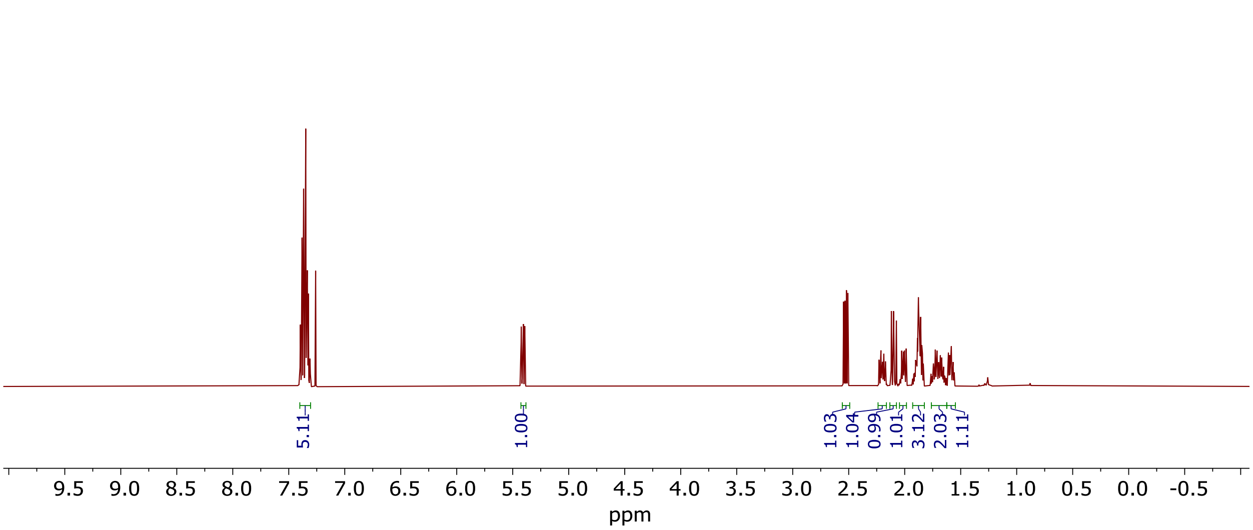


**$^{13}$C NMR** (151MHz, $CDCl_3$) of **3a**

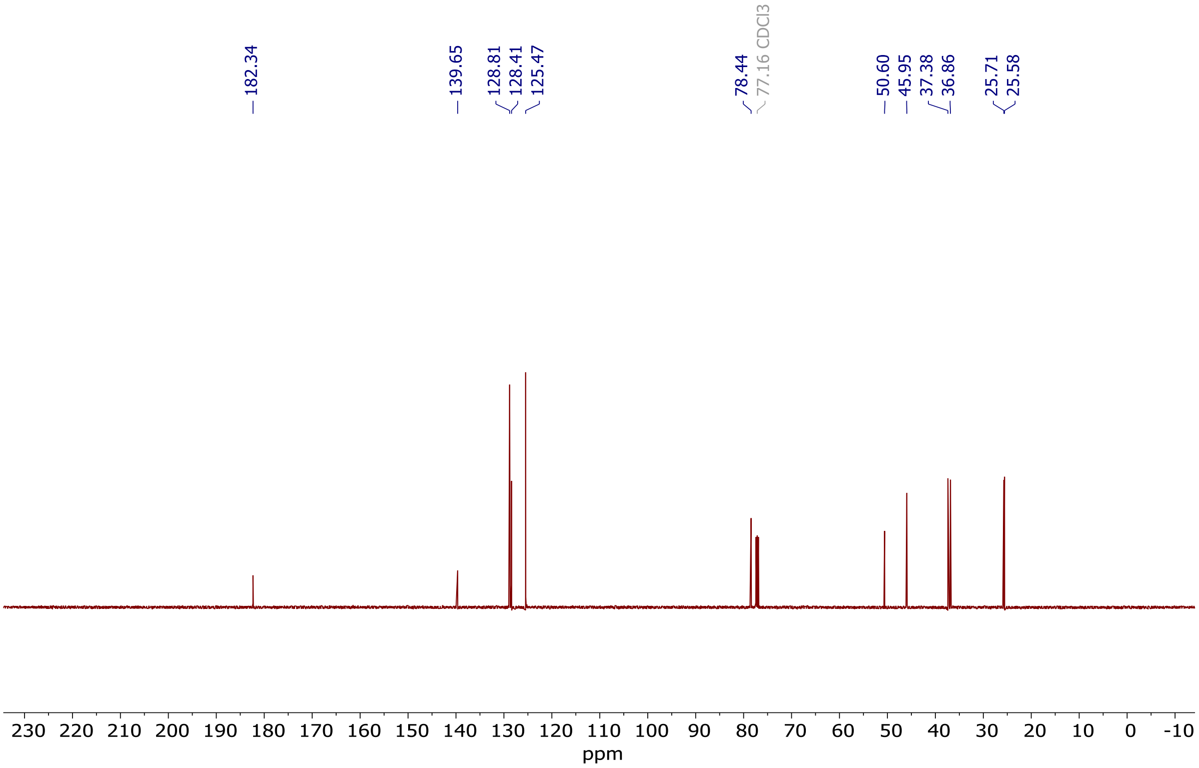

**$^{1}$H NMR** (599 MHz, $CDCl_3$) of **3b** see procedure

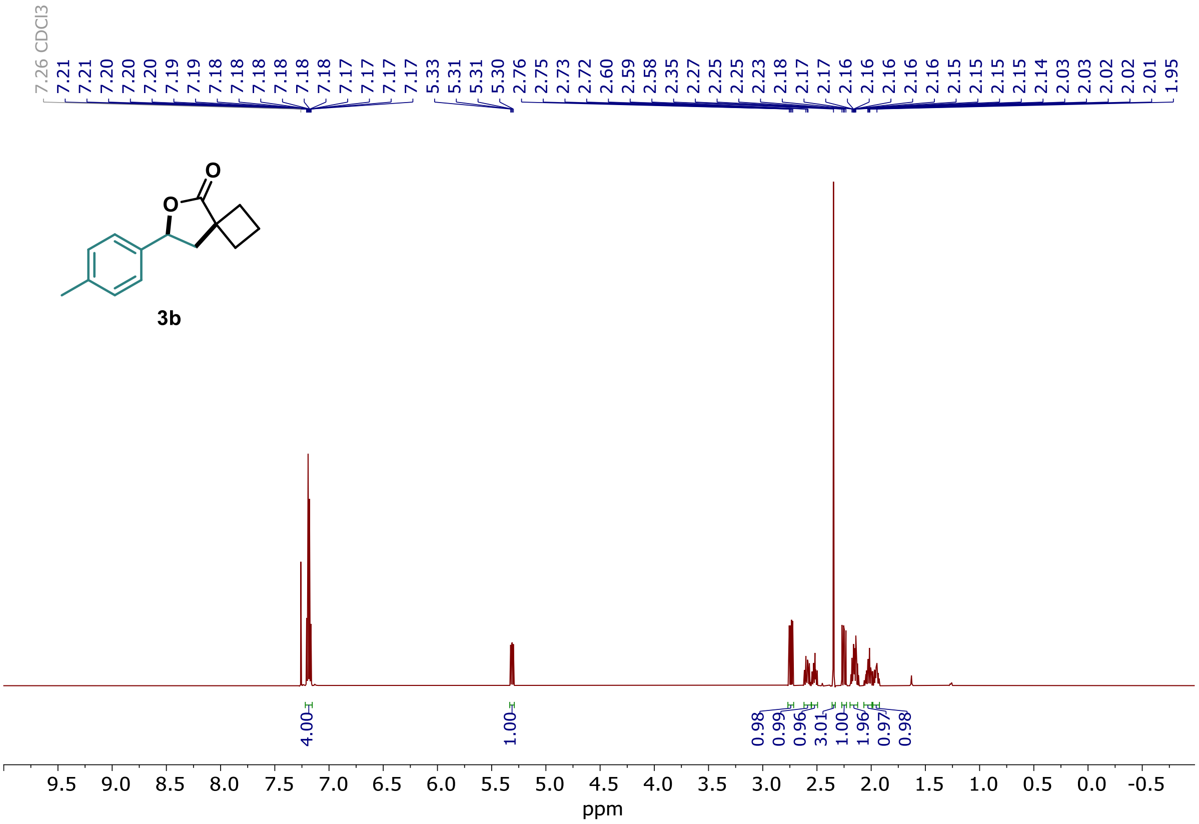


**$^{13}$C NMR** (151 MHz, $CDCl_3$) of **3b**

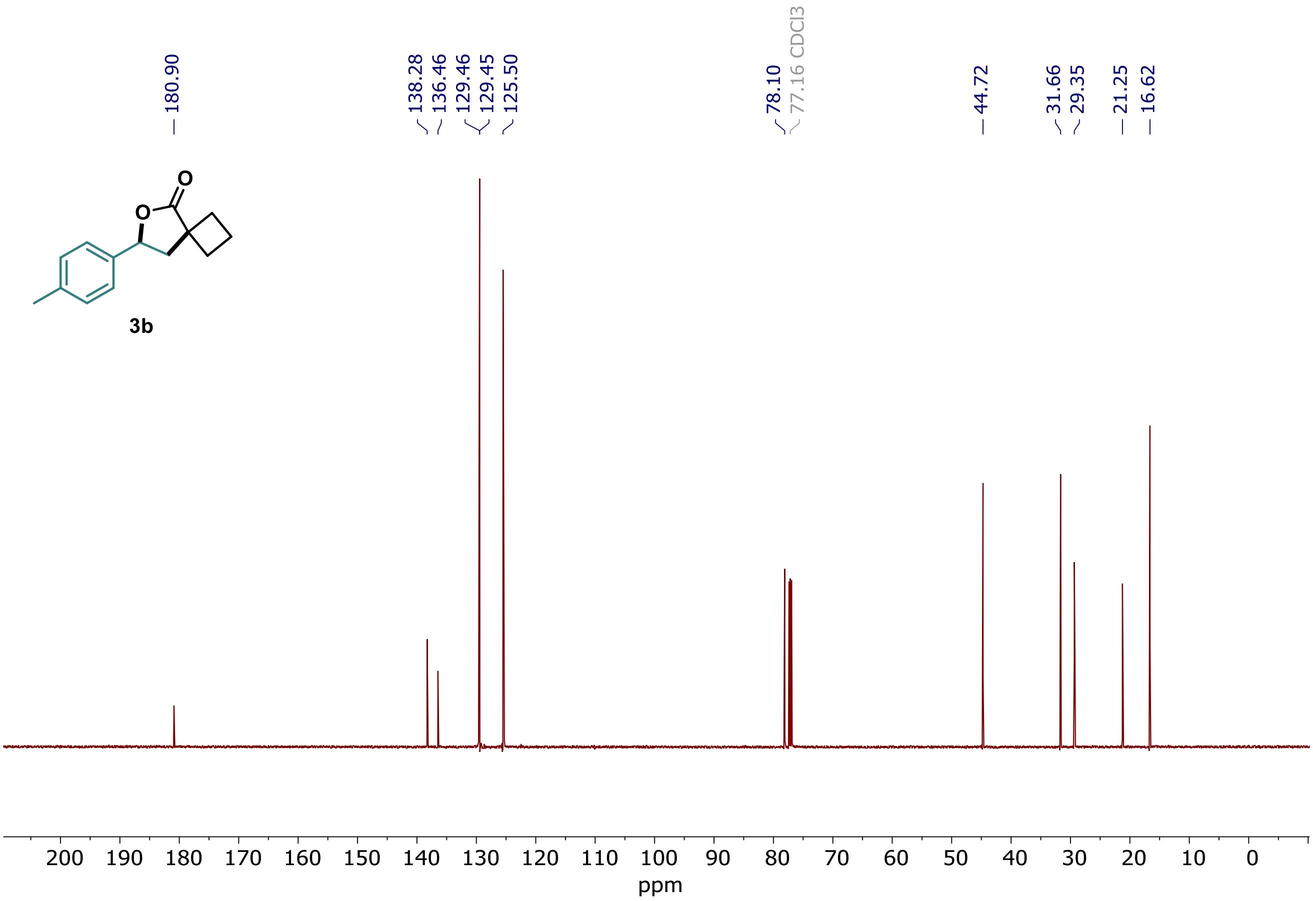

**$^{1}$H NMR** (500 MHz, CDCl$_3$) of **3c** see procedure

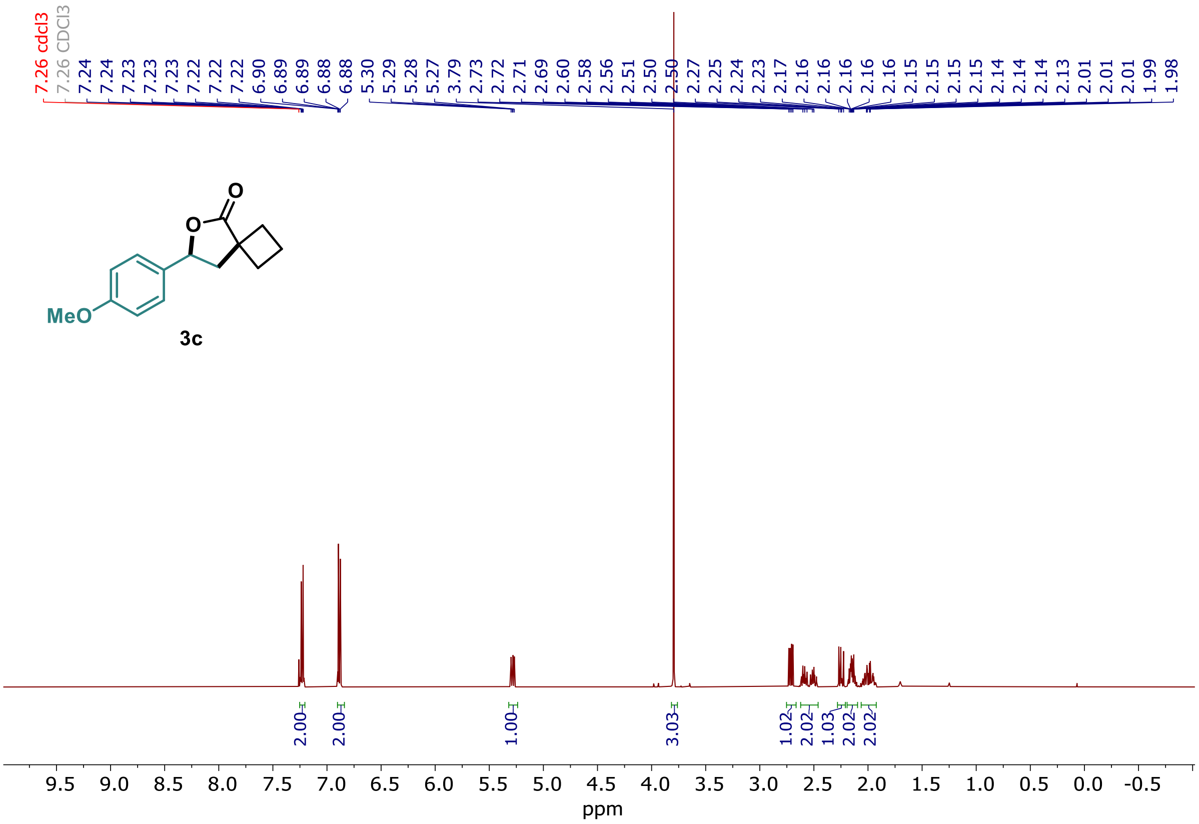


**$^{13}$C NMR** (126 MHz, CDCl3) of 3c

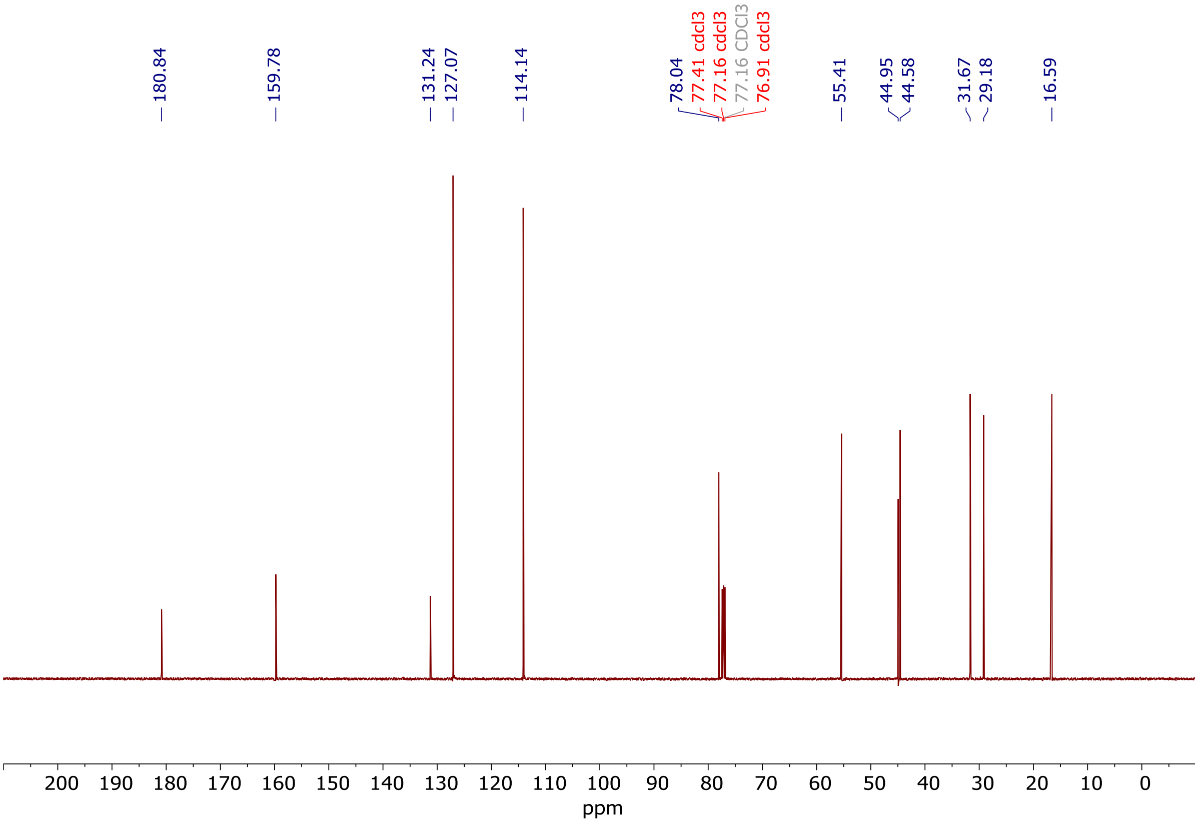

**$^{1}$H NMR** (500 MHz, CDCl$_3$) of **3d** see procedure

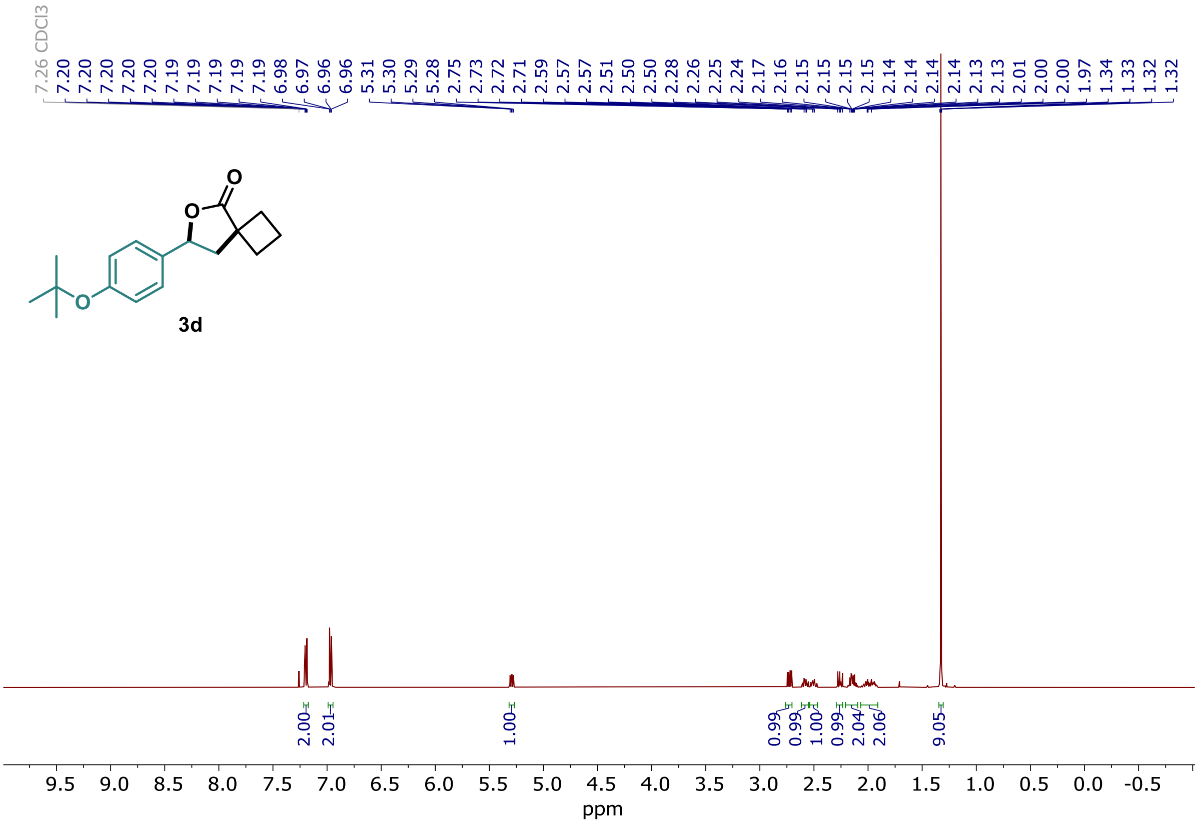


**$^{13}$C NMR** (126MHz, CDCl$_3$) of **3d**

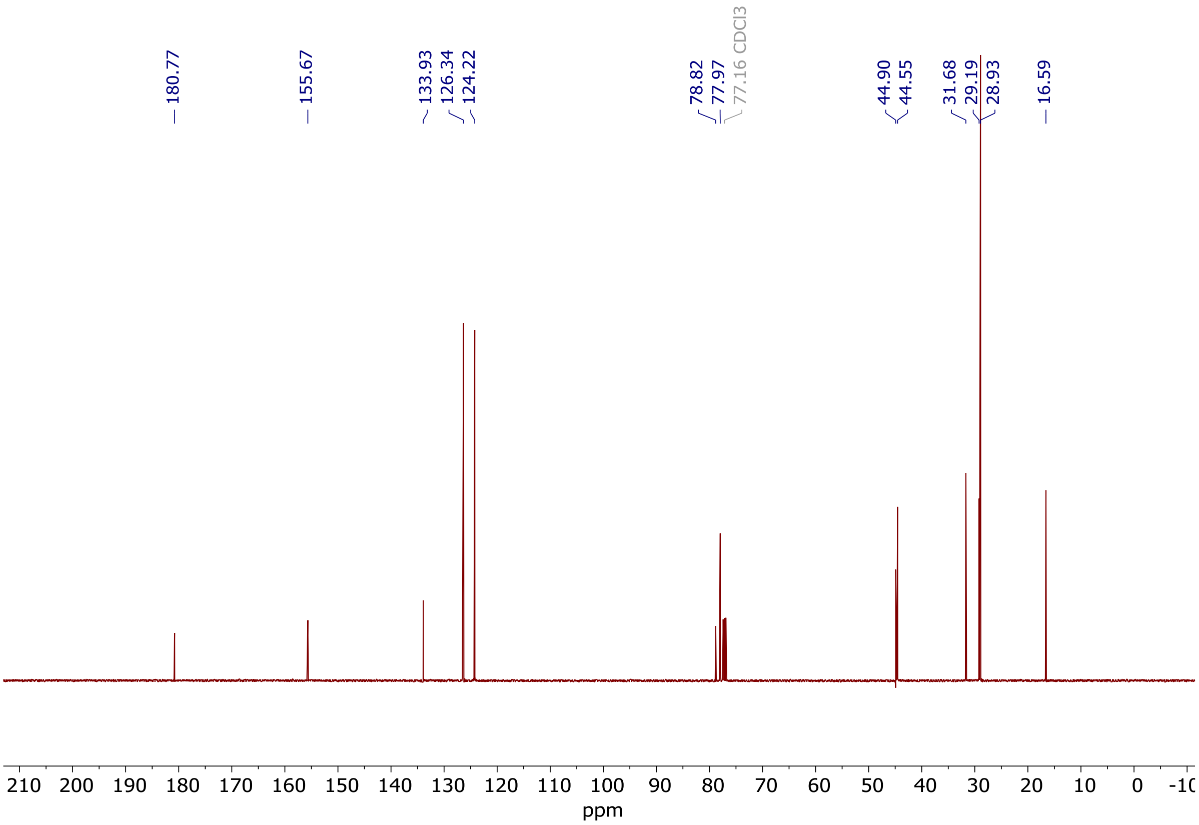

**¹H NMR** (500 MHz, CDCl₃) of **3e** see procedure

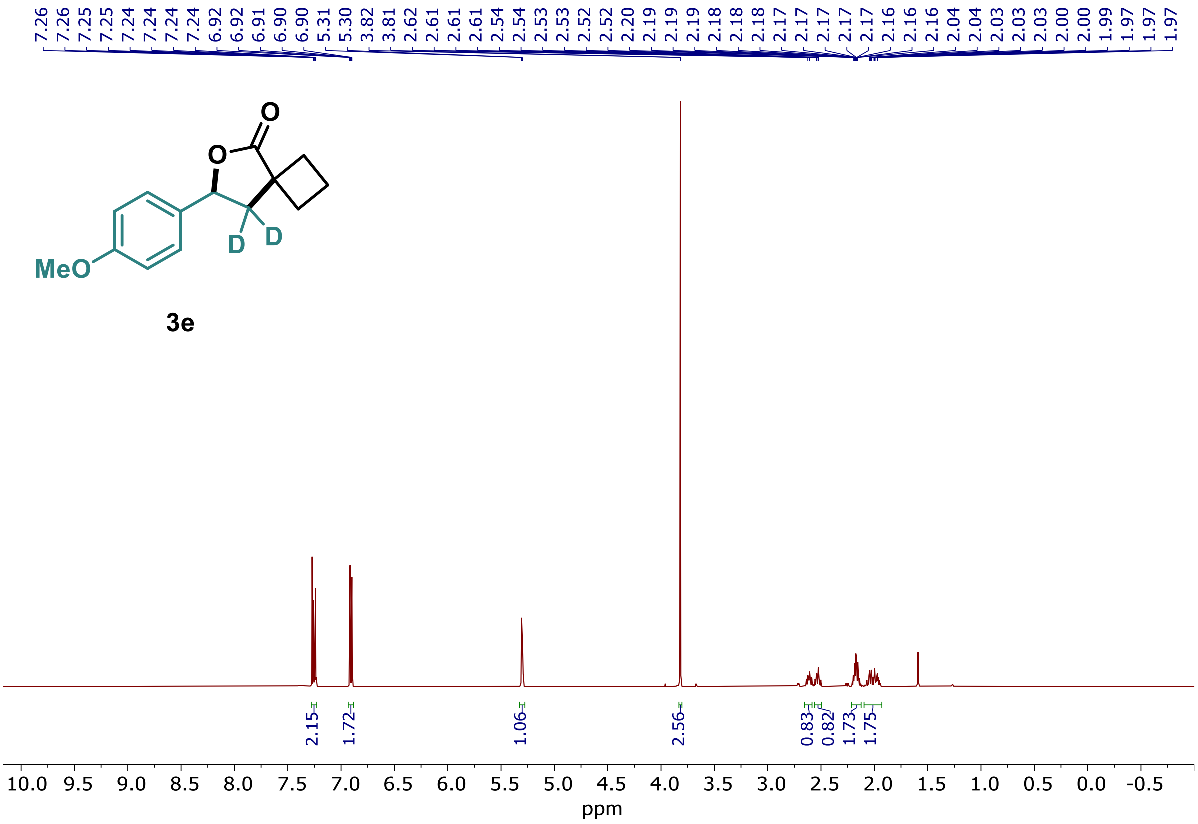


13C NMR (126 MHz, CDCl3) of 3e

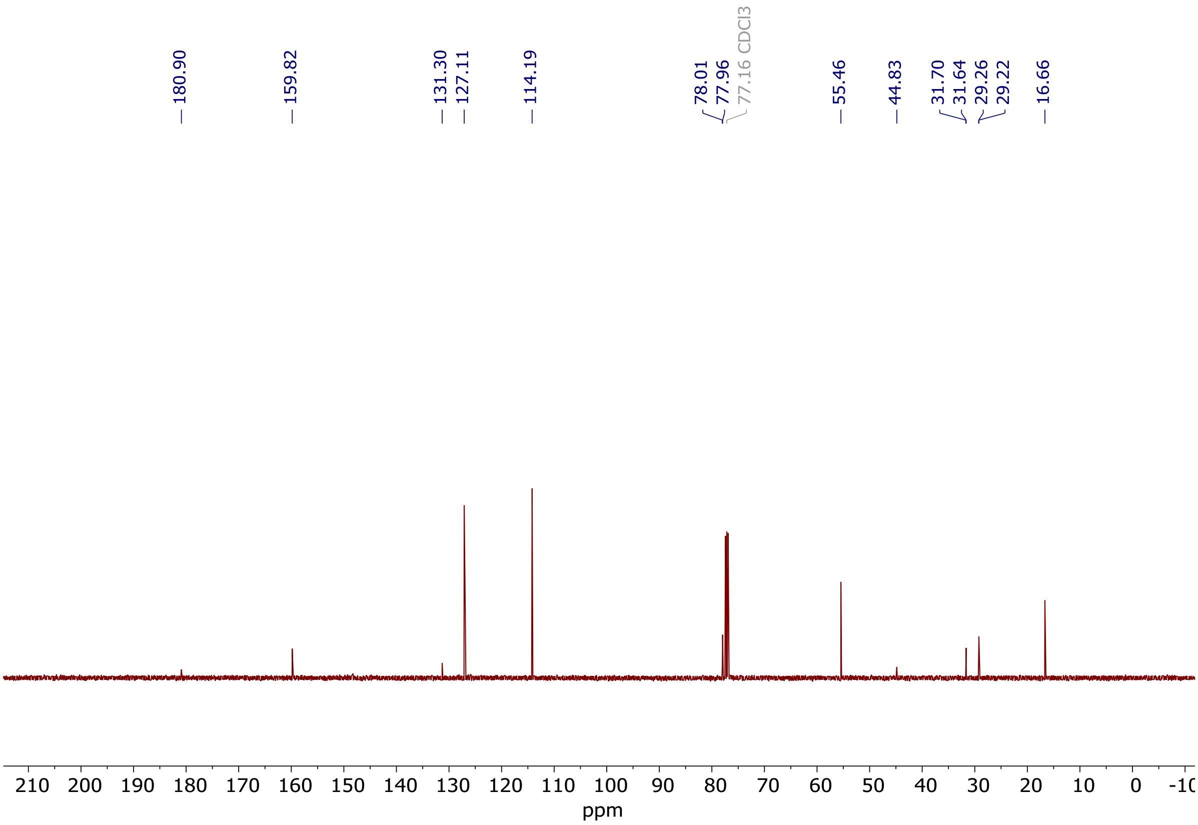

**$^{1}$H NMR** (599 MHz, $CDCl_3$) of **3f** see procedure

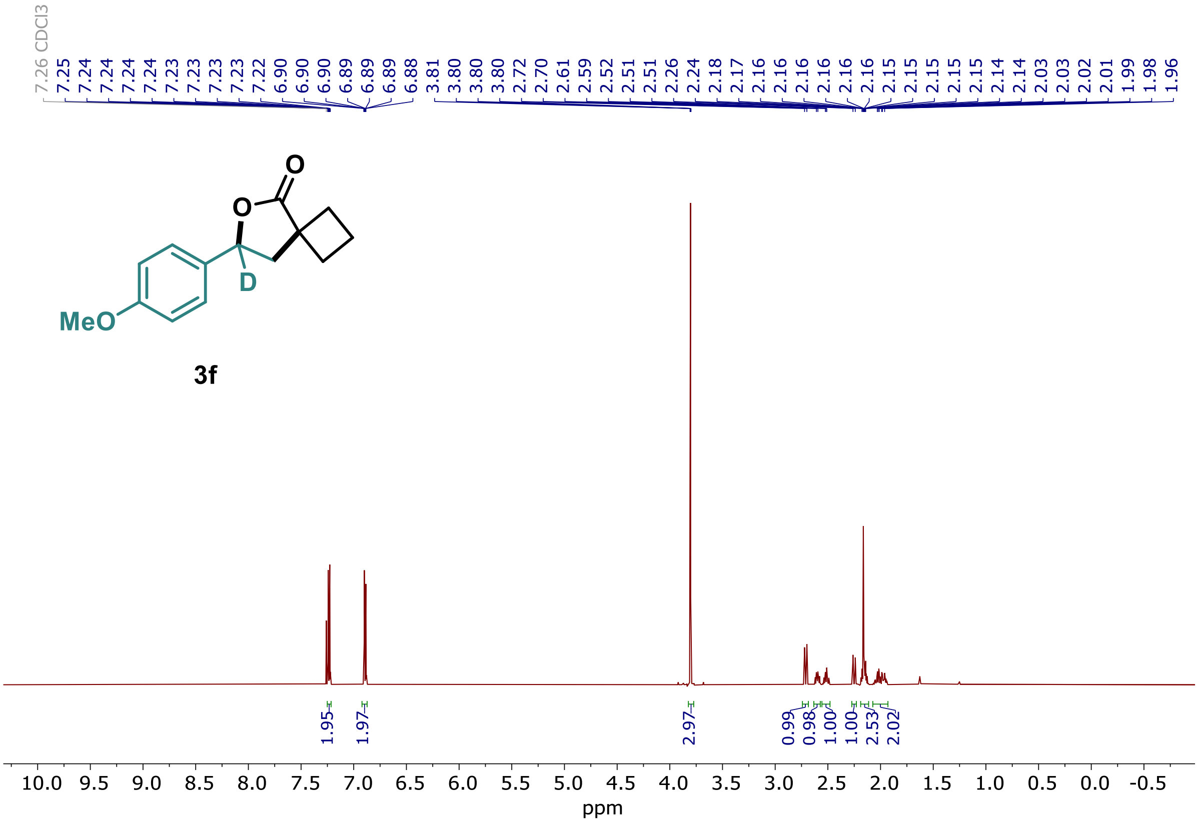


**$^{13}$C NMR** (151MHz, $CDCl_3$) of **3f**

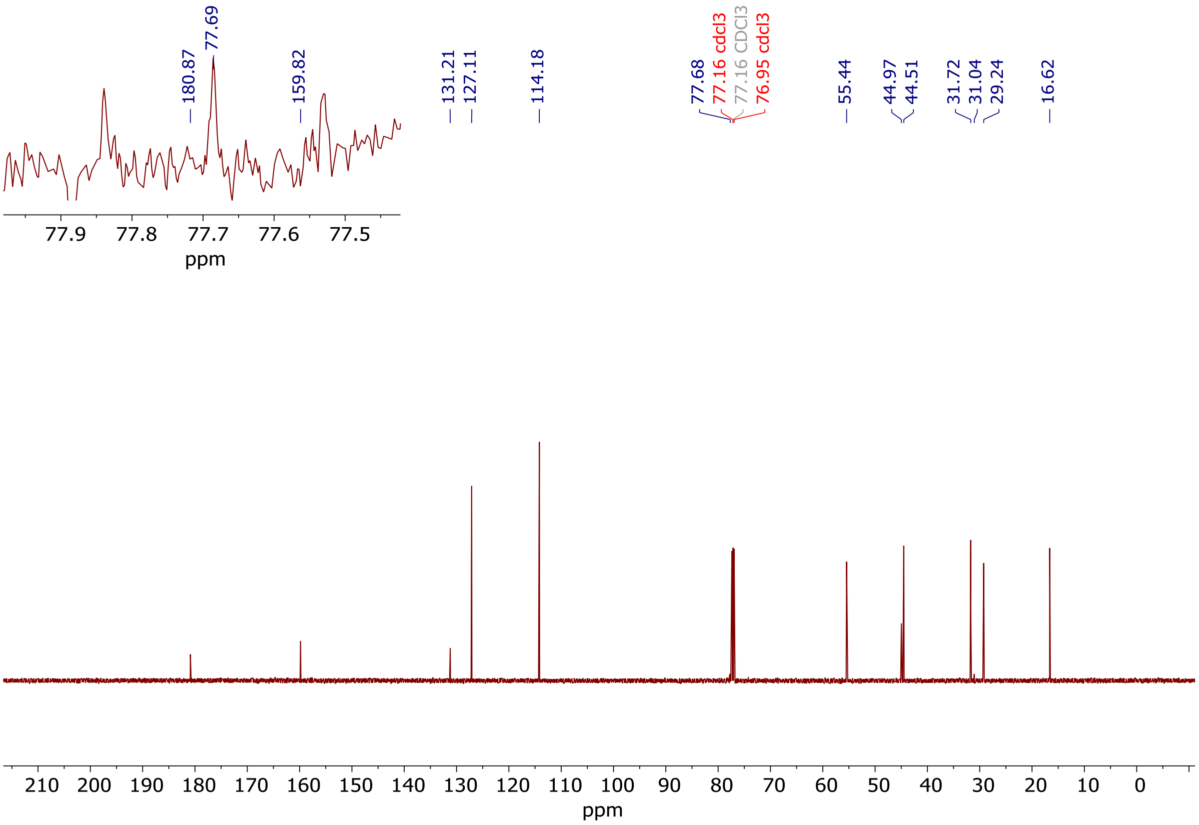

**$^{1}$H NMR** (599 MHz, CDCl3) of **3g** see procedure

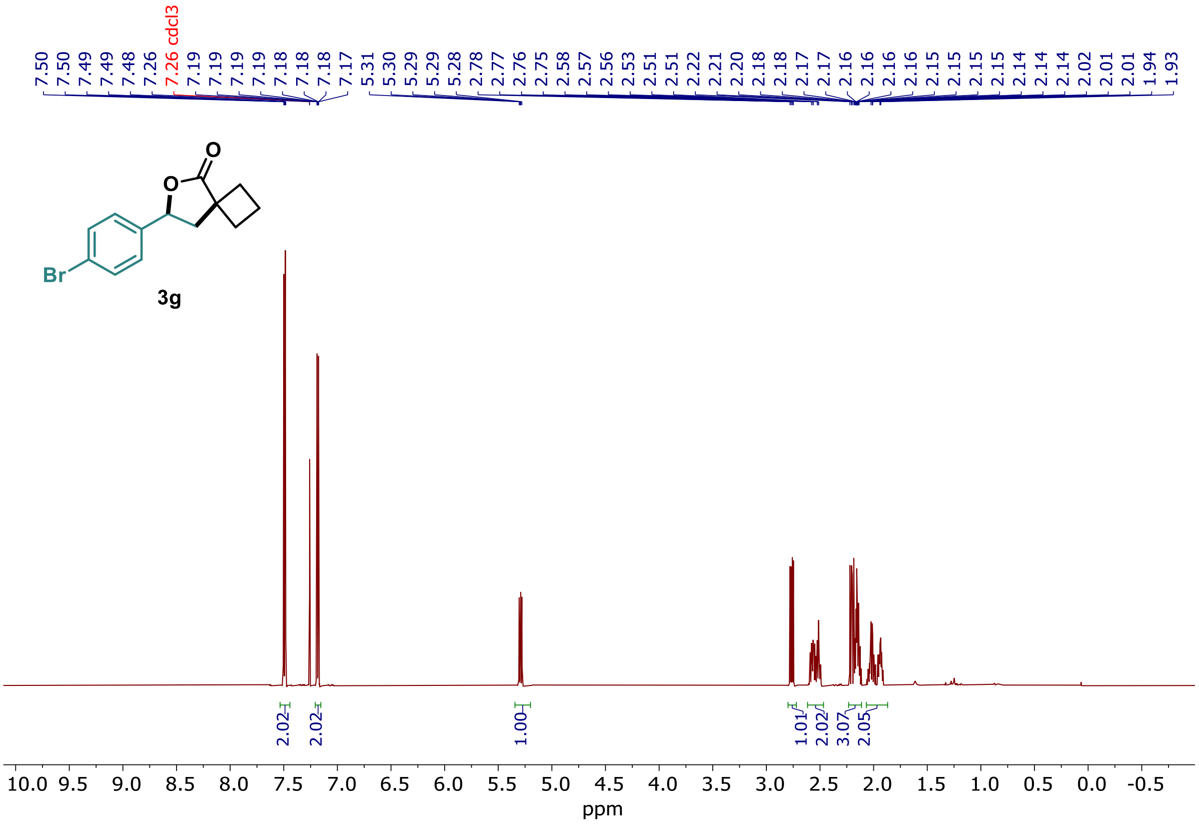


**$^{13}$C NMR** (151MHz, CDCl3) of **3g**

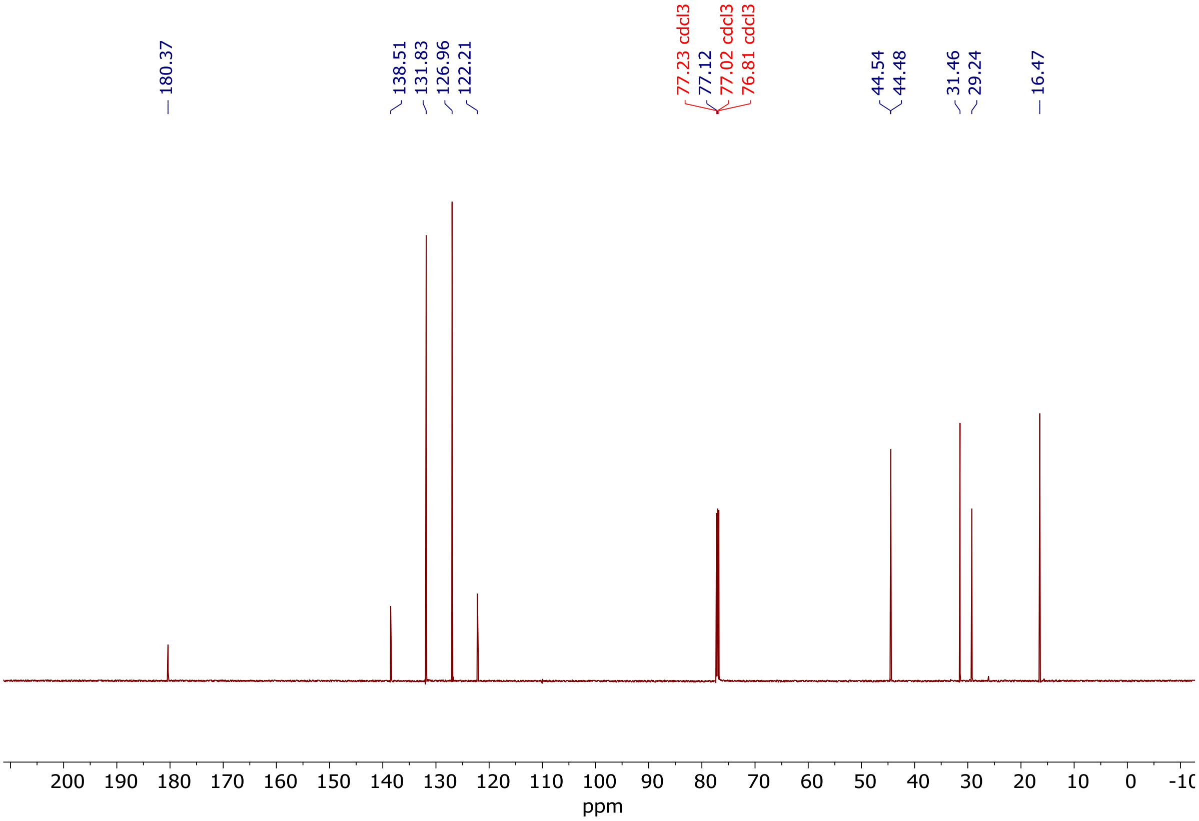

$^{1}$H NMR (500 MHz, CDCl$_3$) of **3h** see procedure

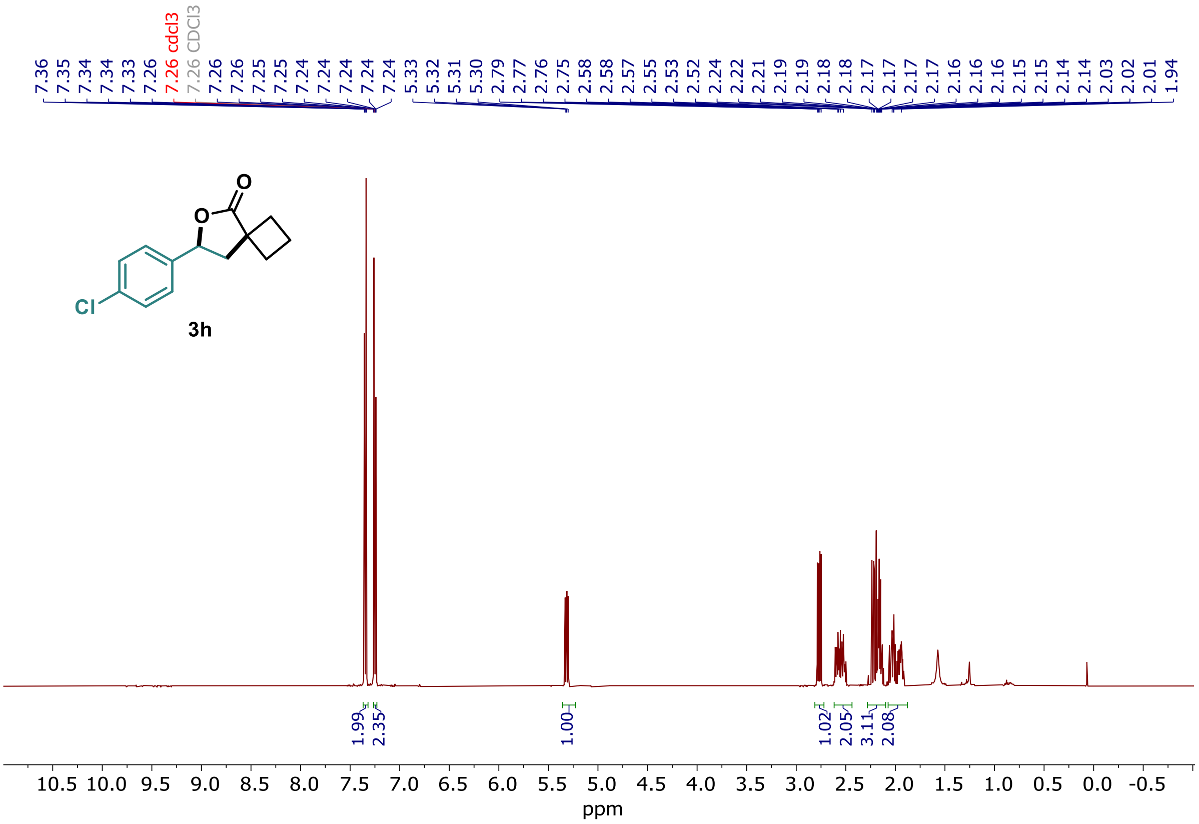


$^{13}$C NMR (126MHz, CDCl$_3$) of **3h**

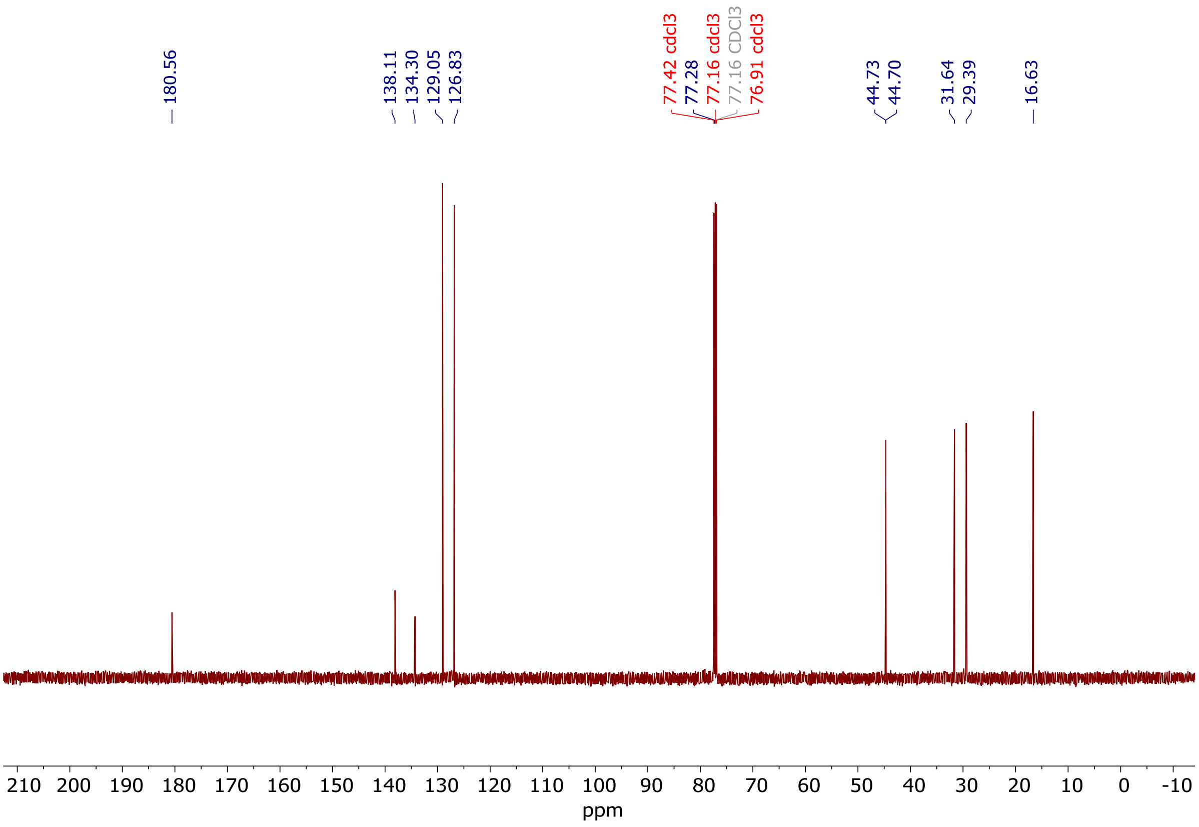

**$^{1}$H NMR** (500 MHz, $CDCl_3$) of **3i** see procedure

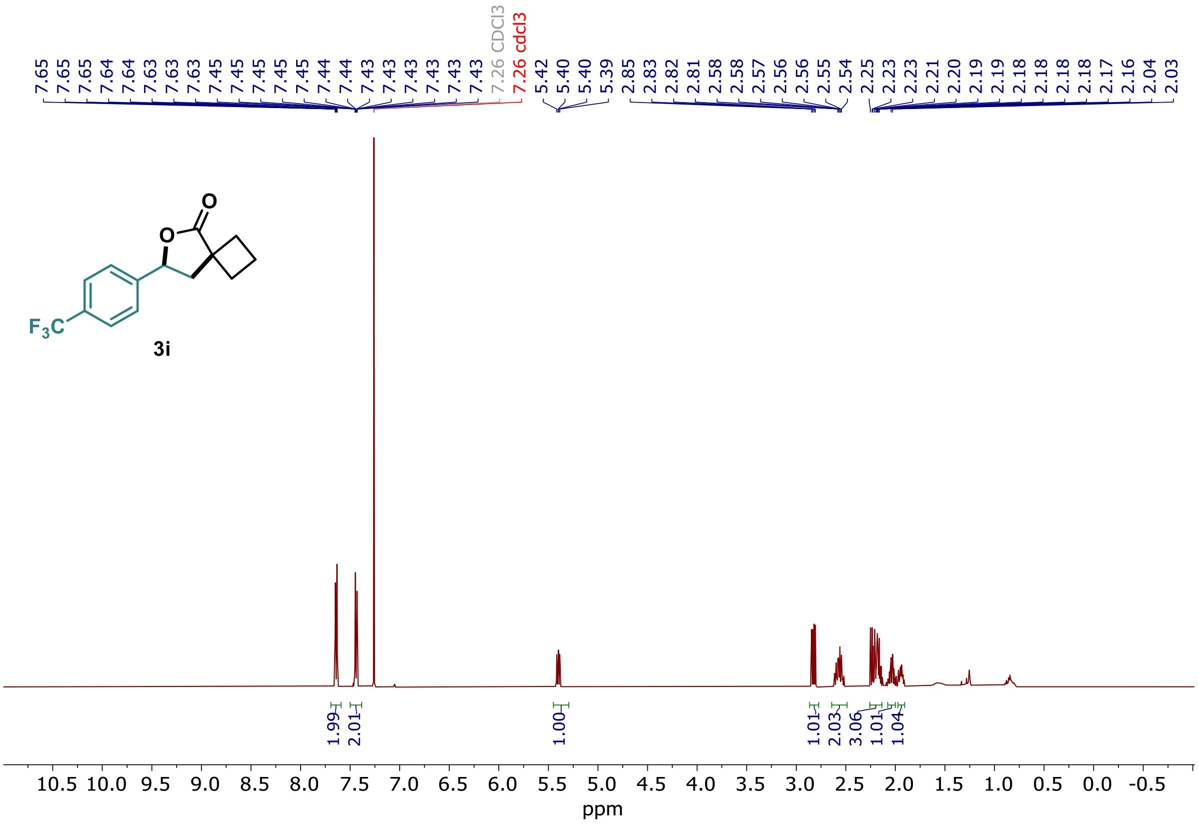


**$^{13}$C NMR** (126 MHz, $CDCl_3$) of **3i**

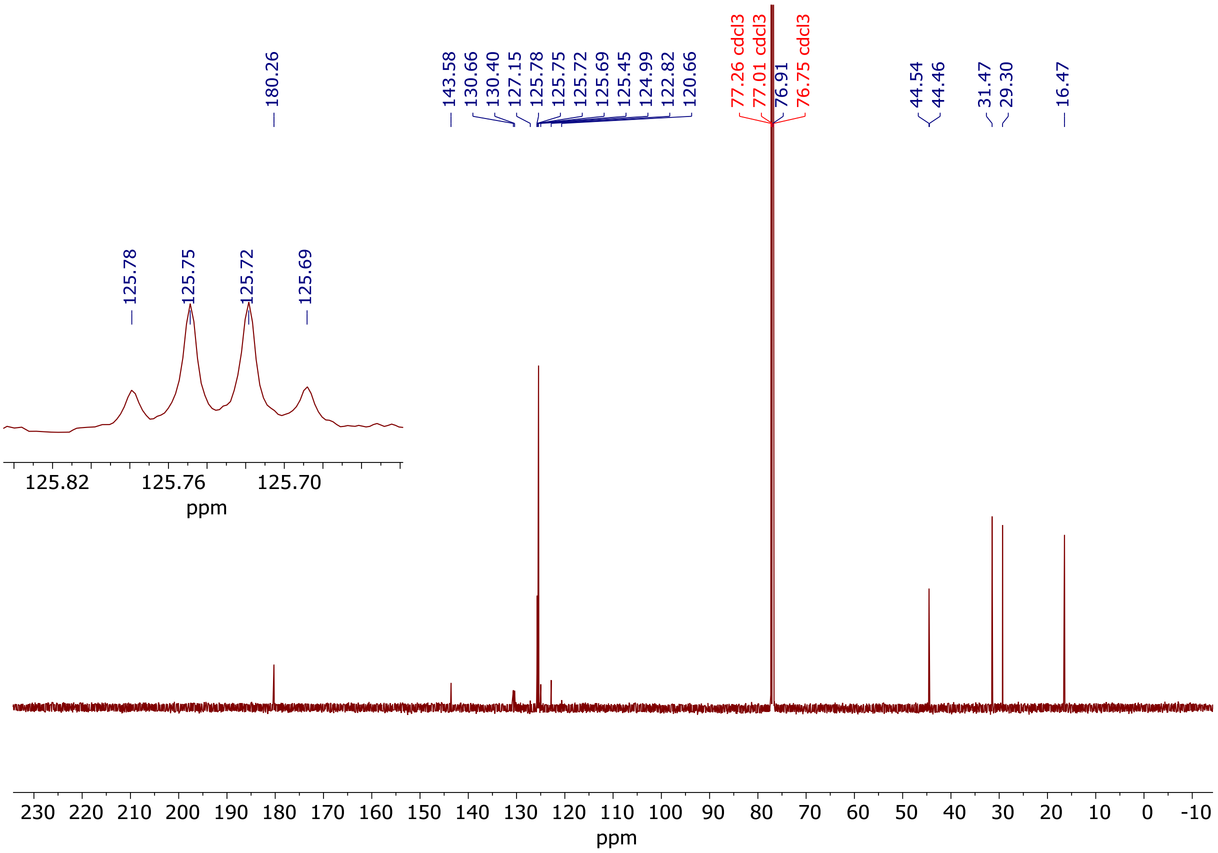

**$^{19}$F NMR** (470 MHz, $CDCl_3$) of **3i**

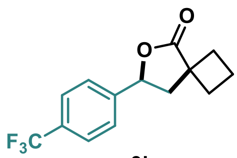


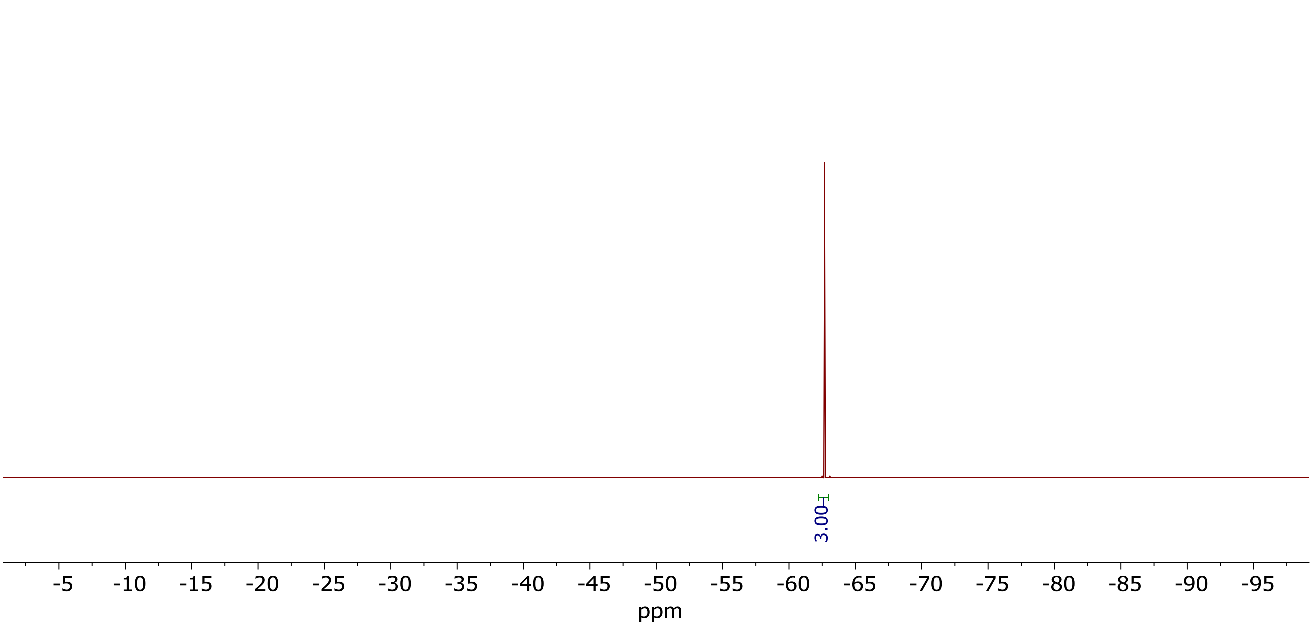

**$^{1}$H NMR** (500 MHz, $CDCl_3$) of **3j** see procedure

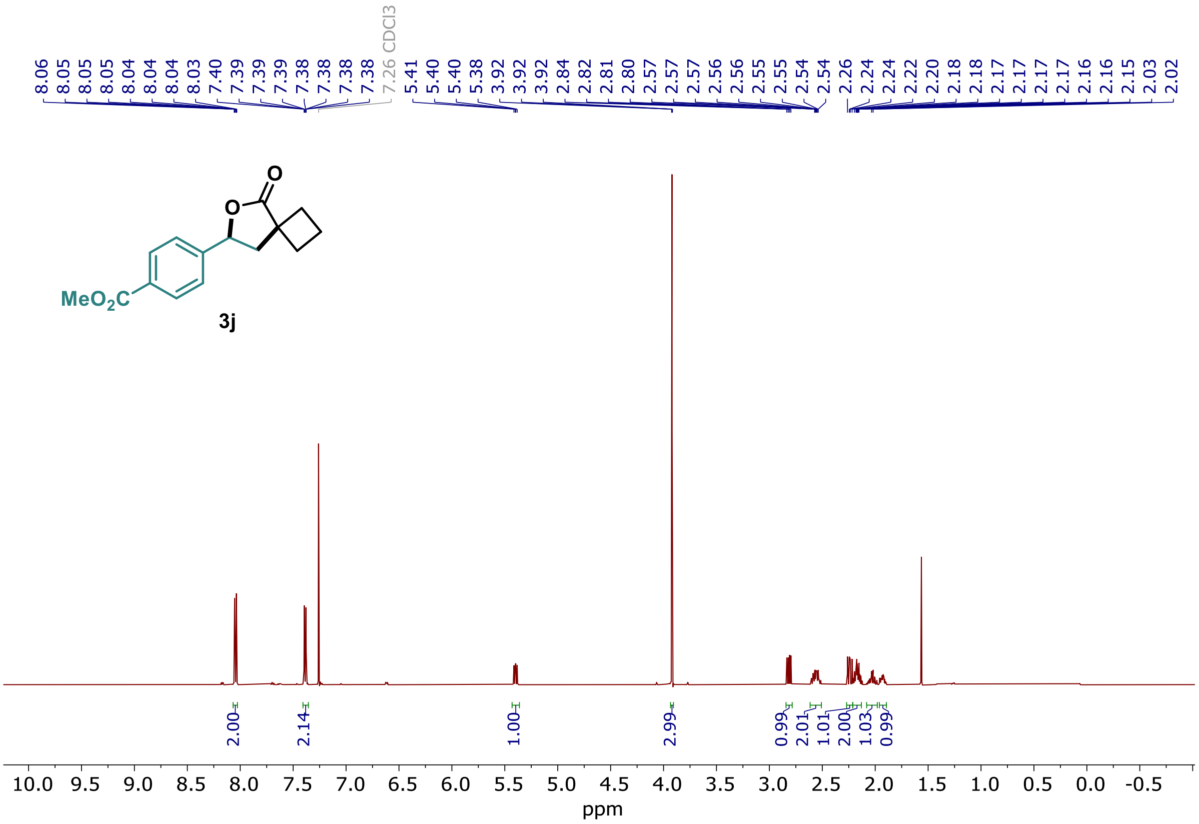


**$^{13}$C NMR** (126 MHz, $CDCl_3$) of **3j**

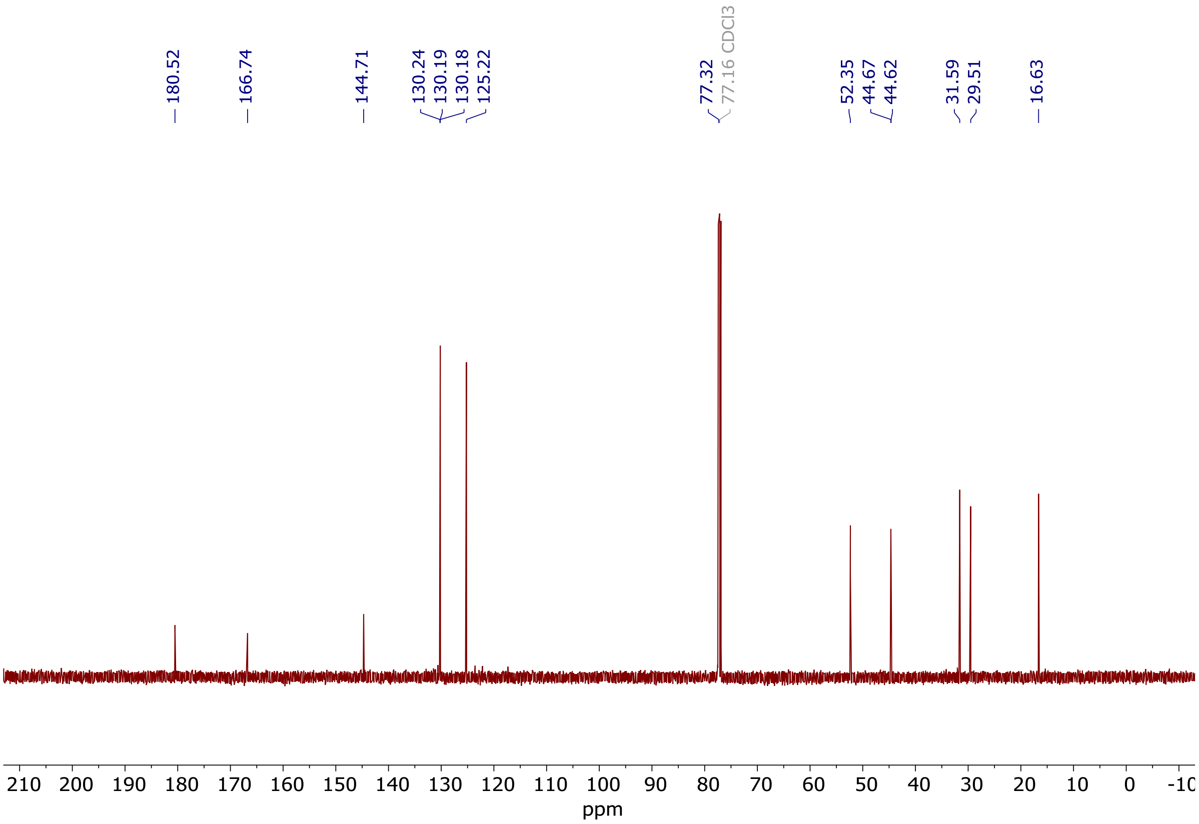

**$^{1}$H NMR** (500 MHz, $CDCl_3$) of **3k** see procedure

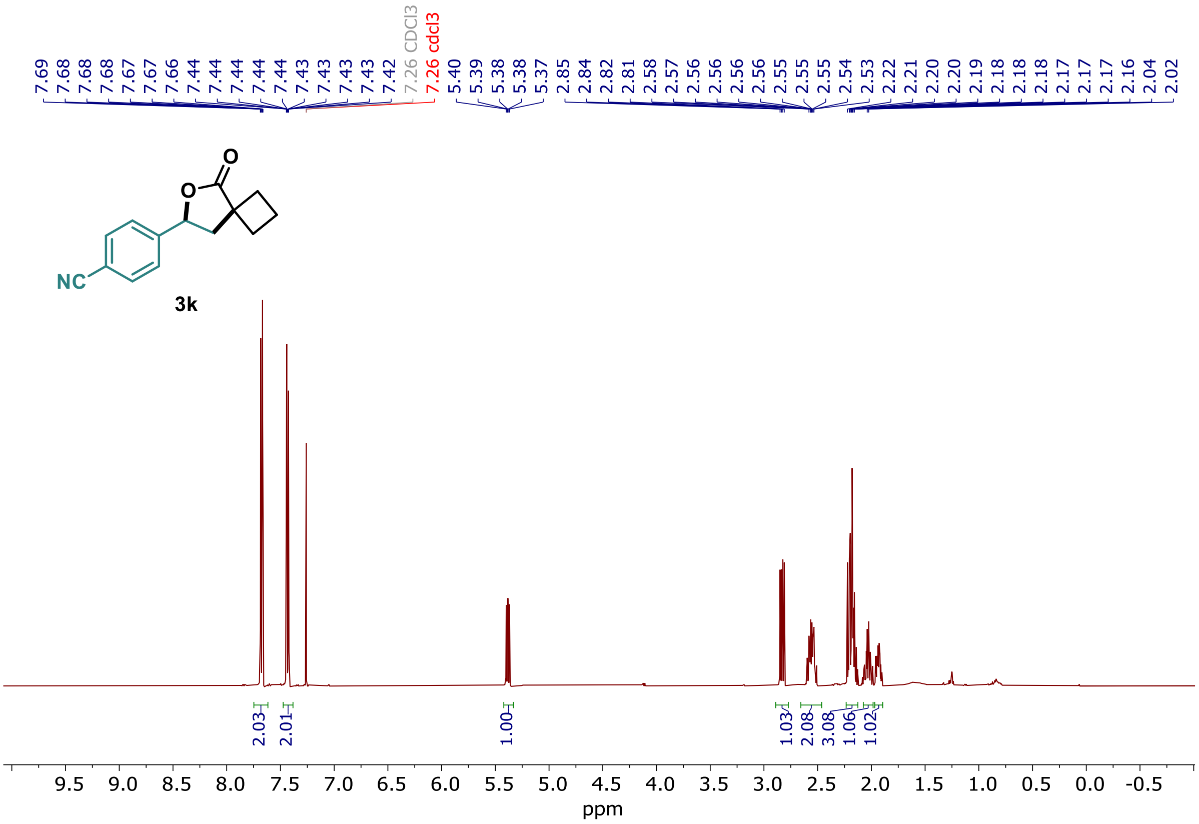


**$^{13}$C NMR** (126MHz, $CDCl_3$) of **3k**

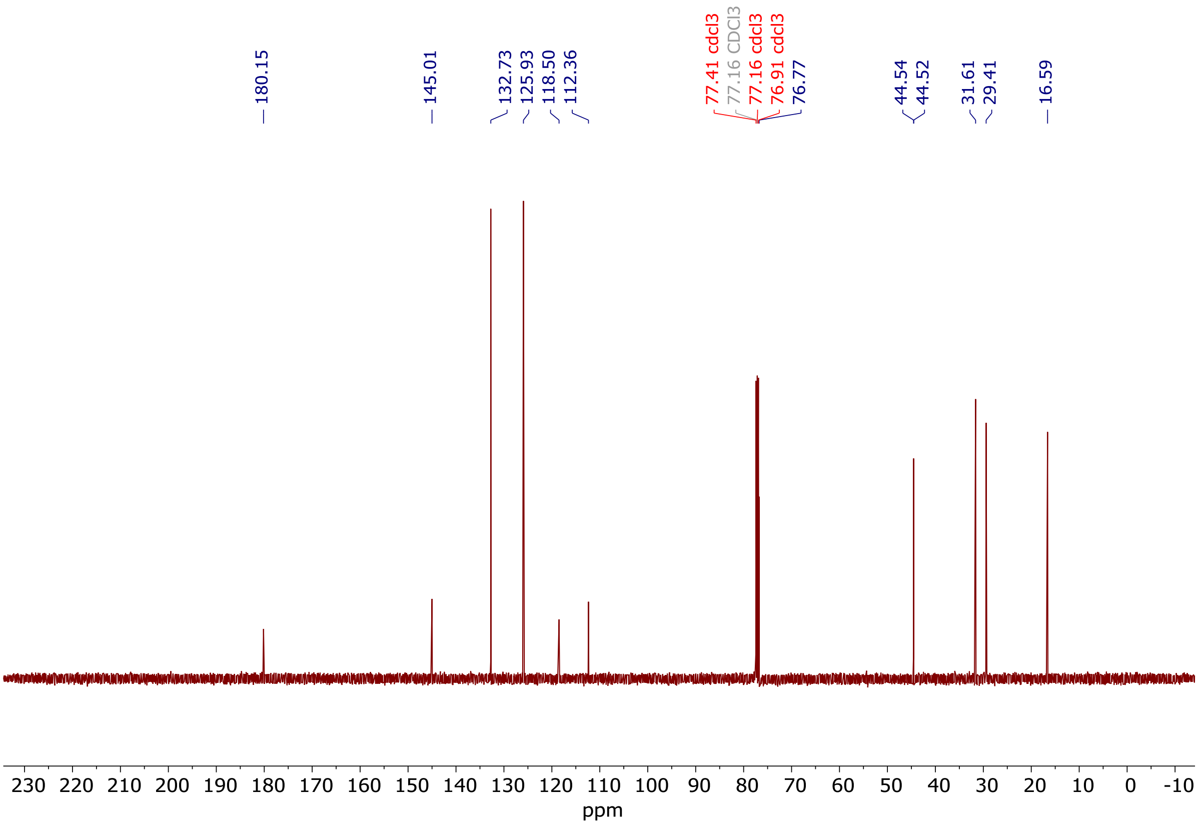

**$^{1}$H NMR** (599 MHz, $CDCl_3$) of **3l** see procedure

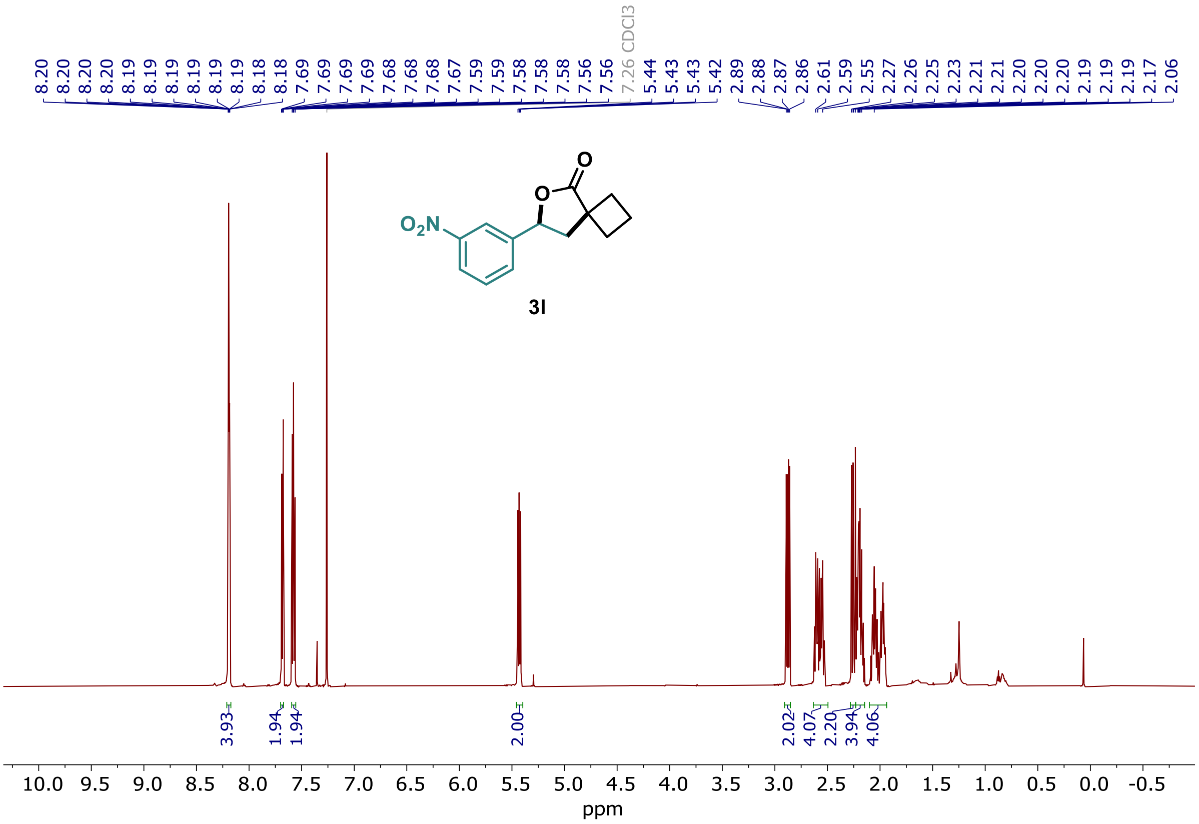


**$^{13}$C NMR** (151MHz, $CDCl_3$) of **3l**

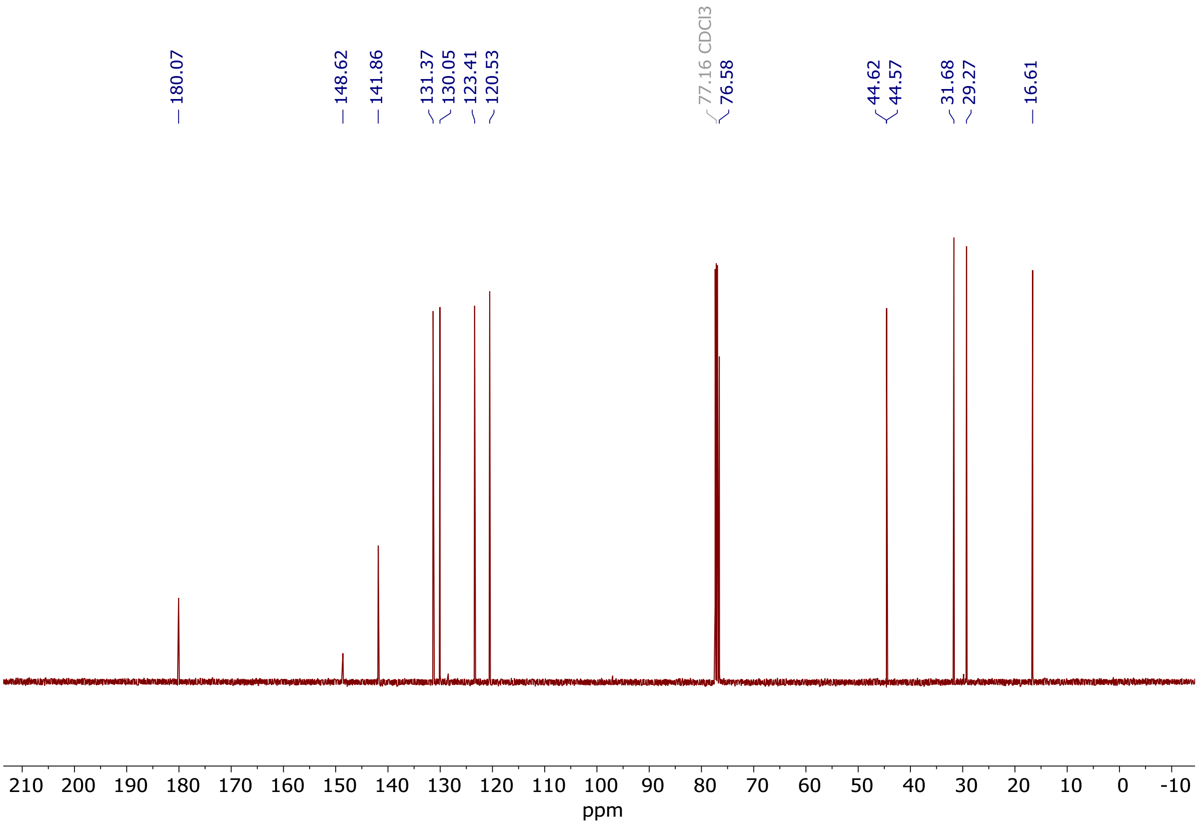

**$^{1}$H NMR** (599 MHz, CDCl$_3$) of **3m** see procedure

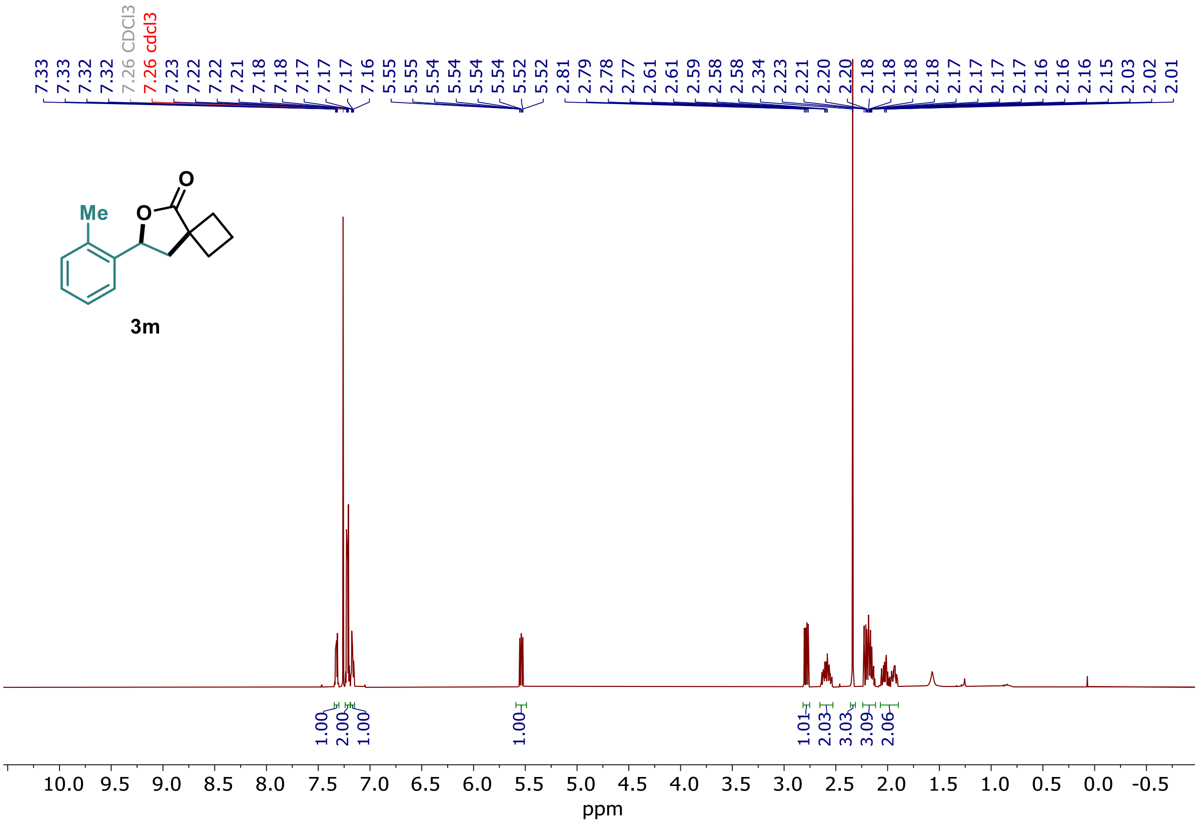


**$^{13}$C NMR** (151MHz, CDCl$_3$) of **3m**

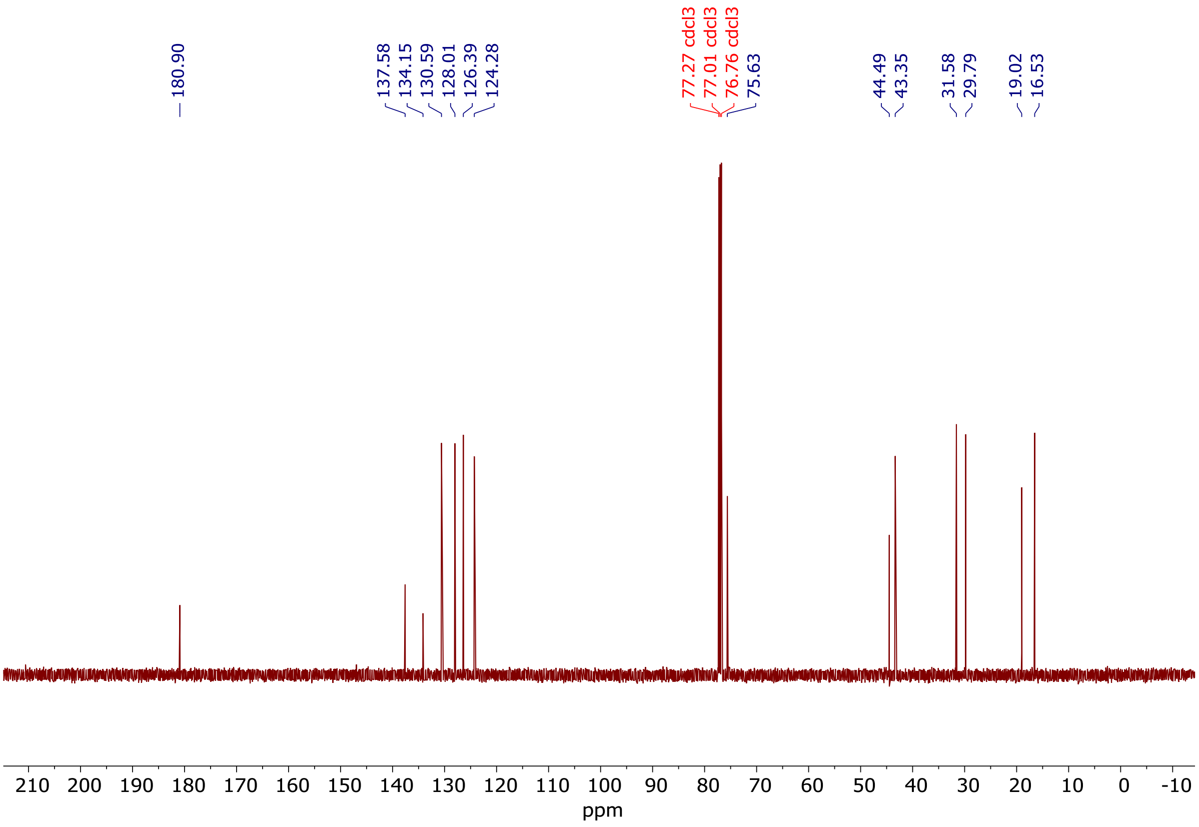

**$^{1}$H NMR** (599 MHz, $CDCl_3$) of **3n** see procedure

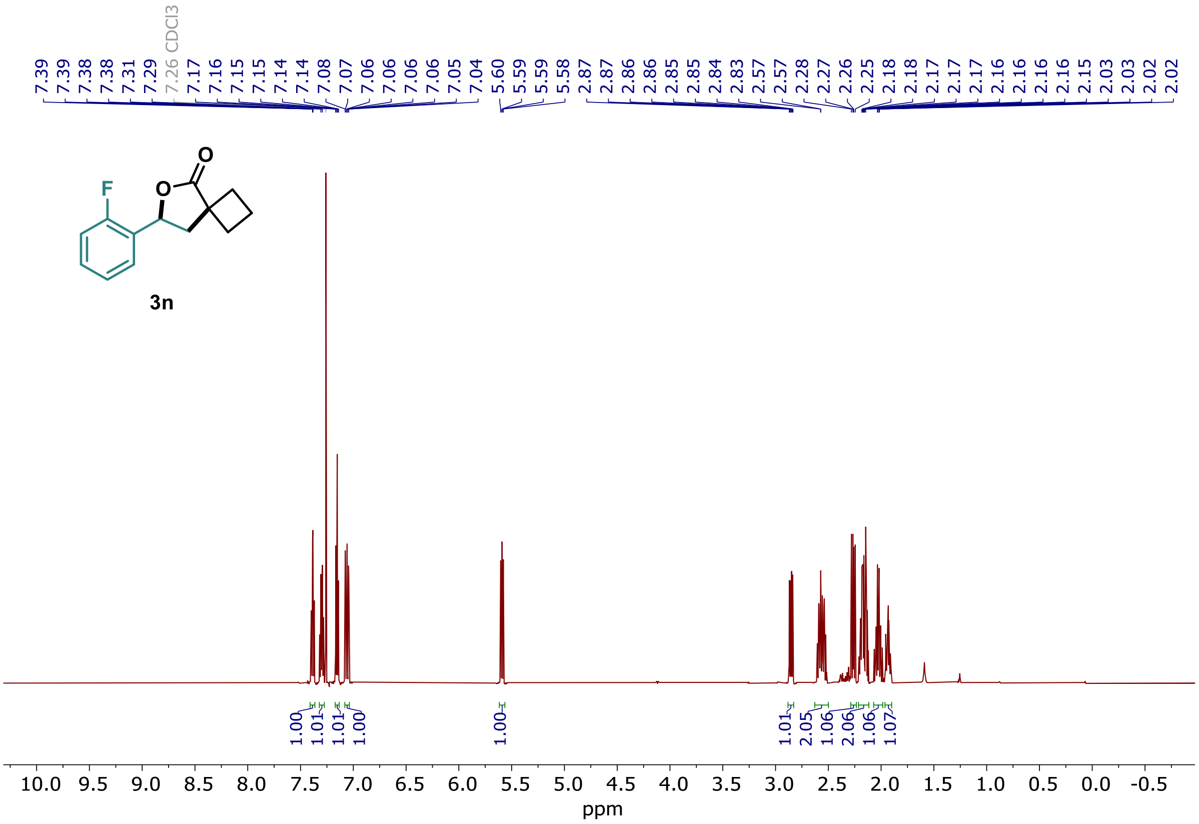


**$^{13}$C NMR** (151MHz, $CDCl_3$) of **3n**

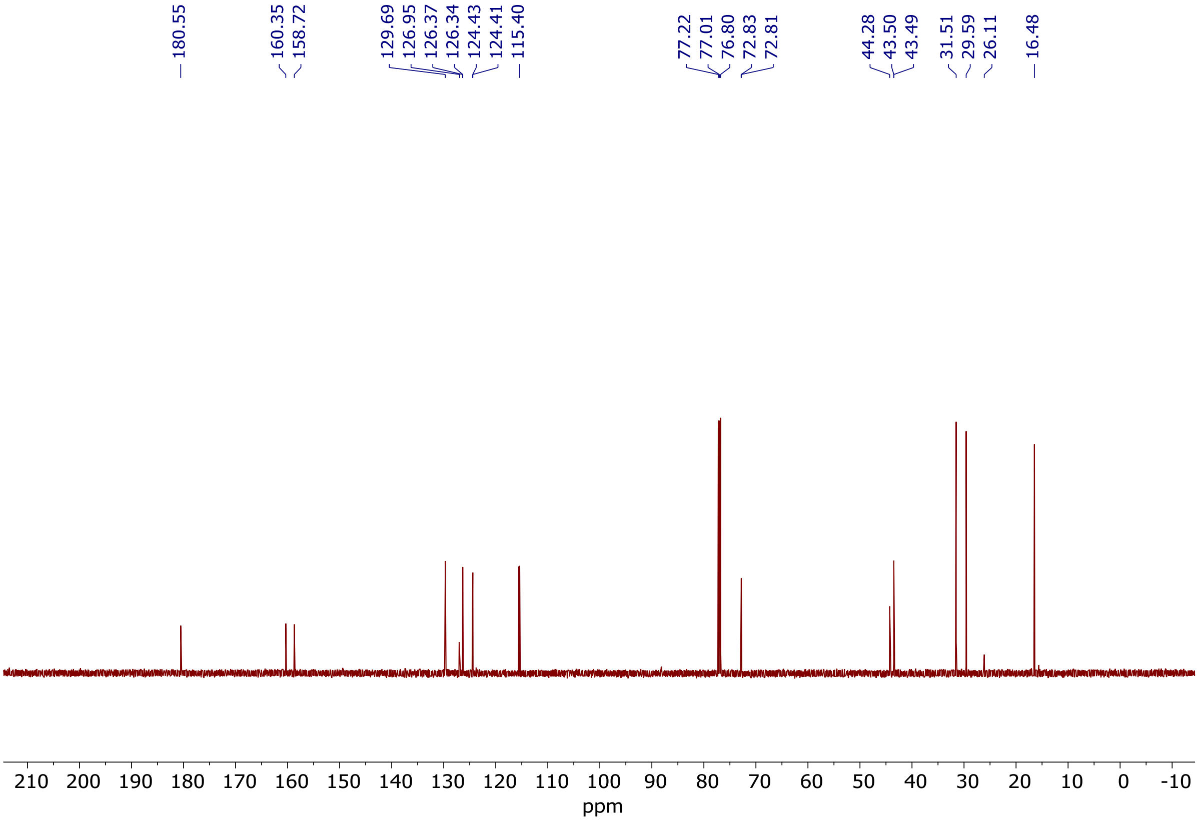

**$^{19}$F NMR** (564MHz, $CDCl_3$) of **3n**

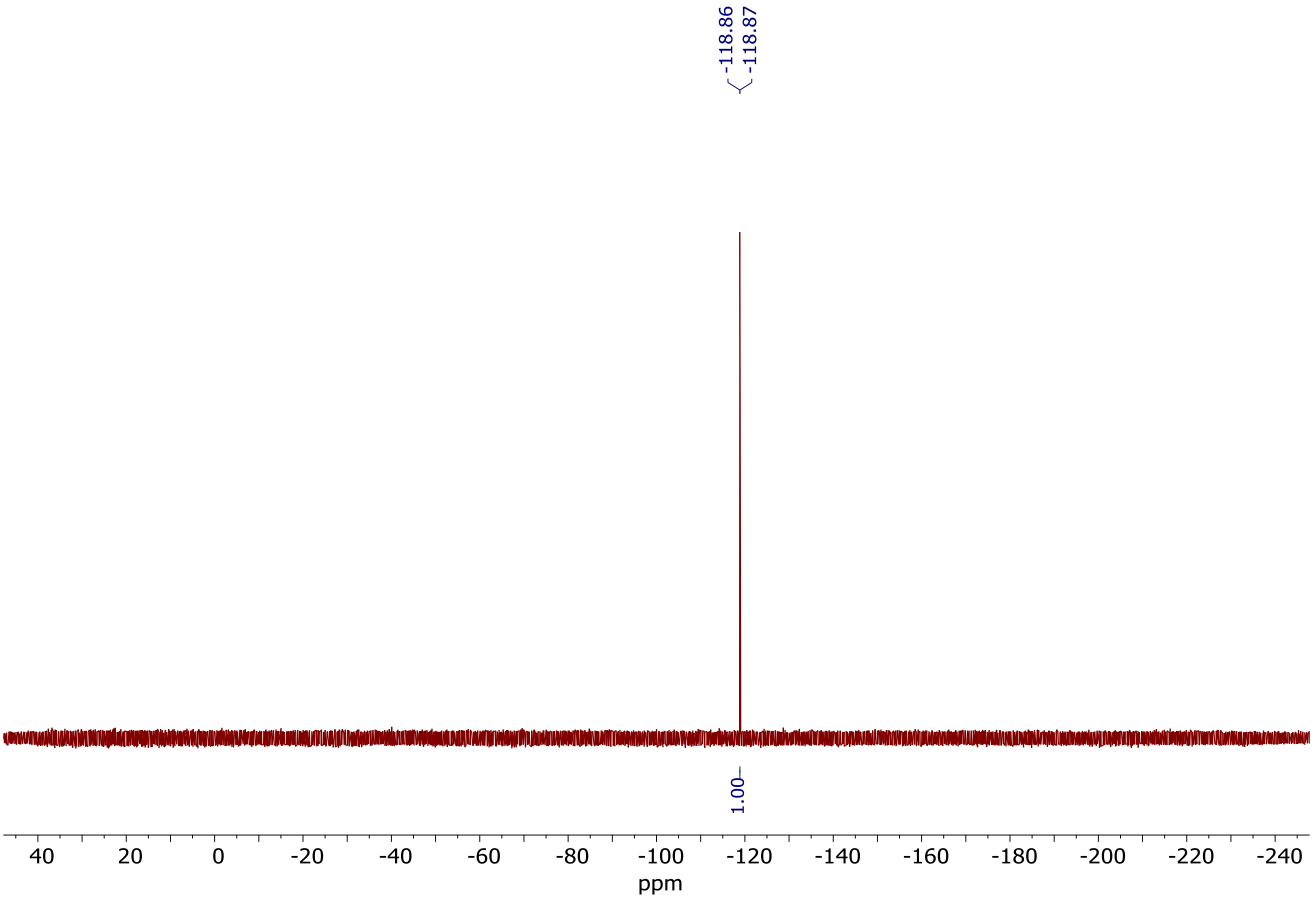

**$^{1}$H NMR** (599 MHz, CDCl$_3$) of **3o** see procedure

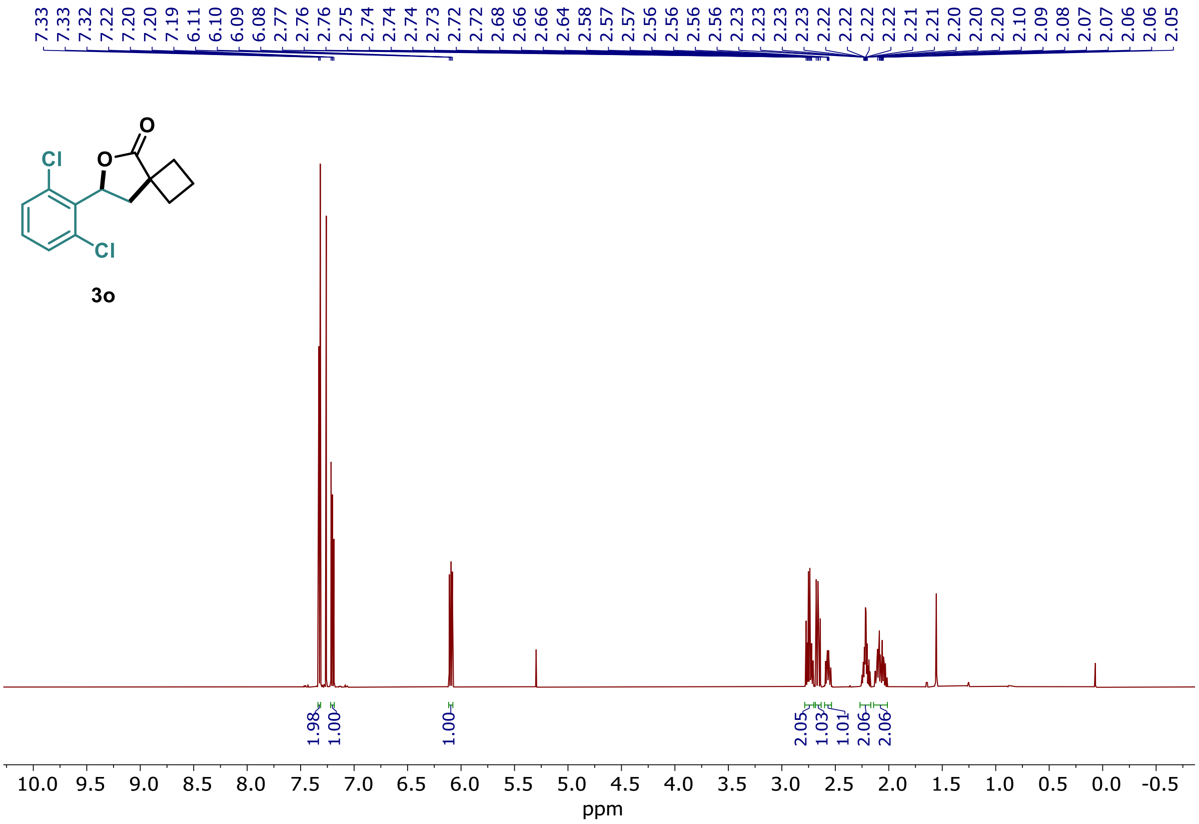


**$^{13}$C NMR** (151 MHz, CDCl$_3$) of **3o**

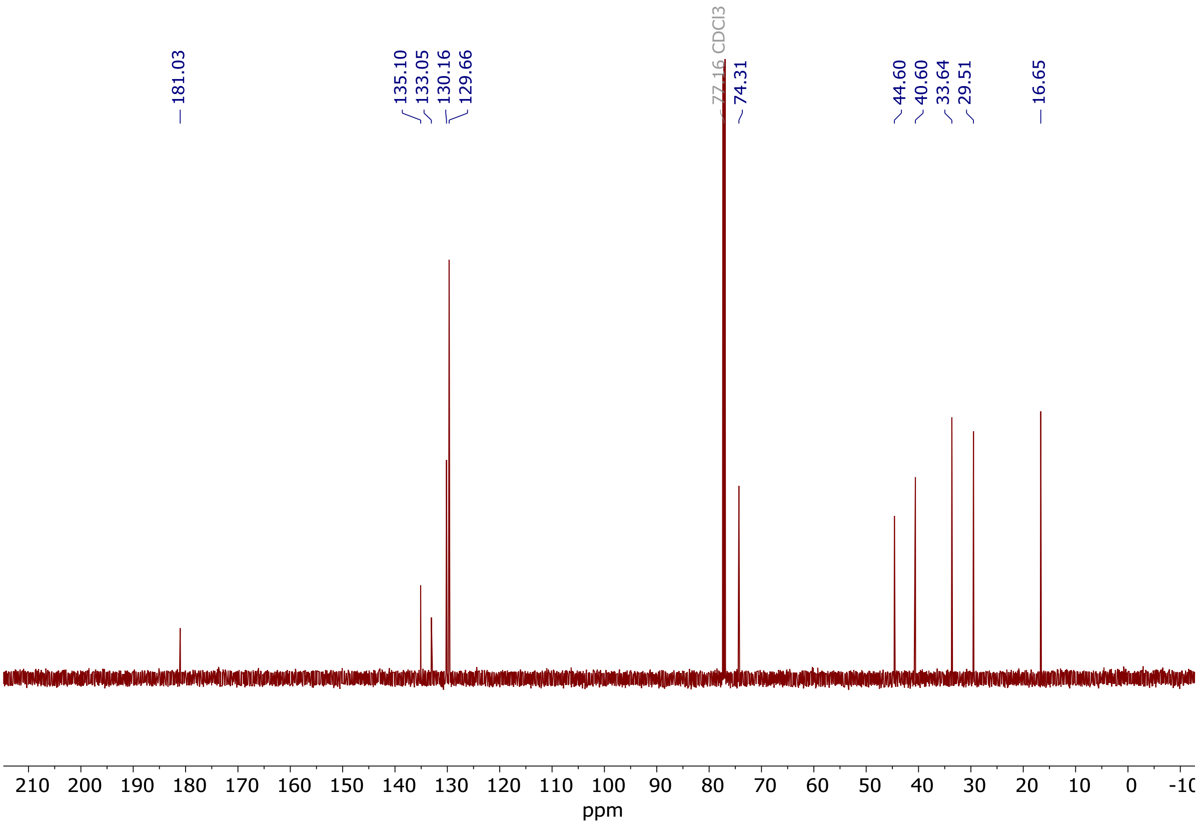

**¹H NMR** (500 MHz, $CDCl_3$) of **3p** see procedure

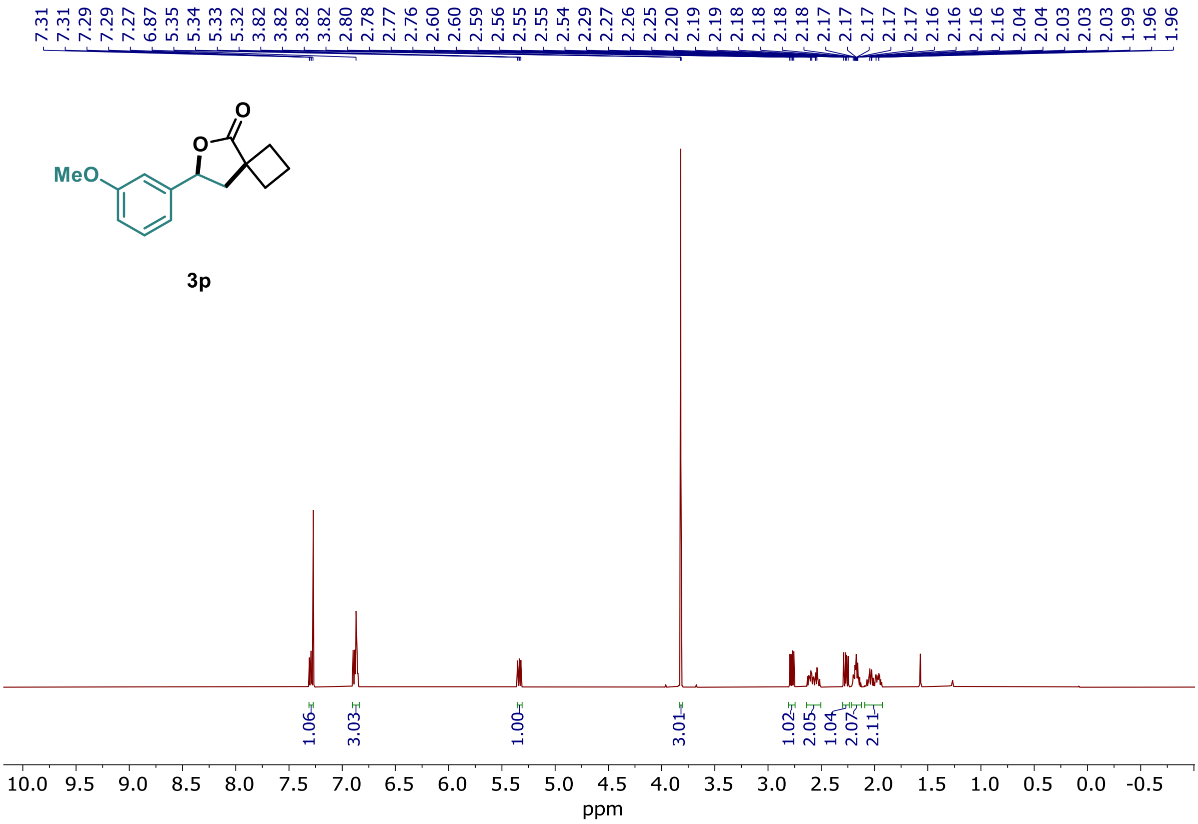


**¹³C NMR** (126 MHz, $CDCl_3$) of **3p**

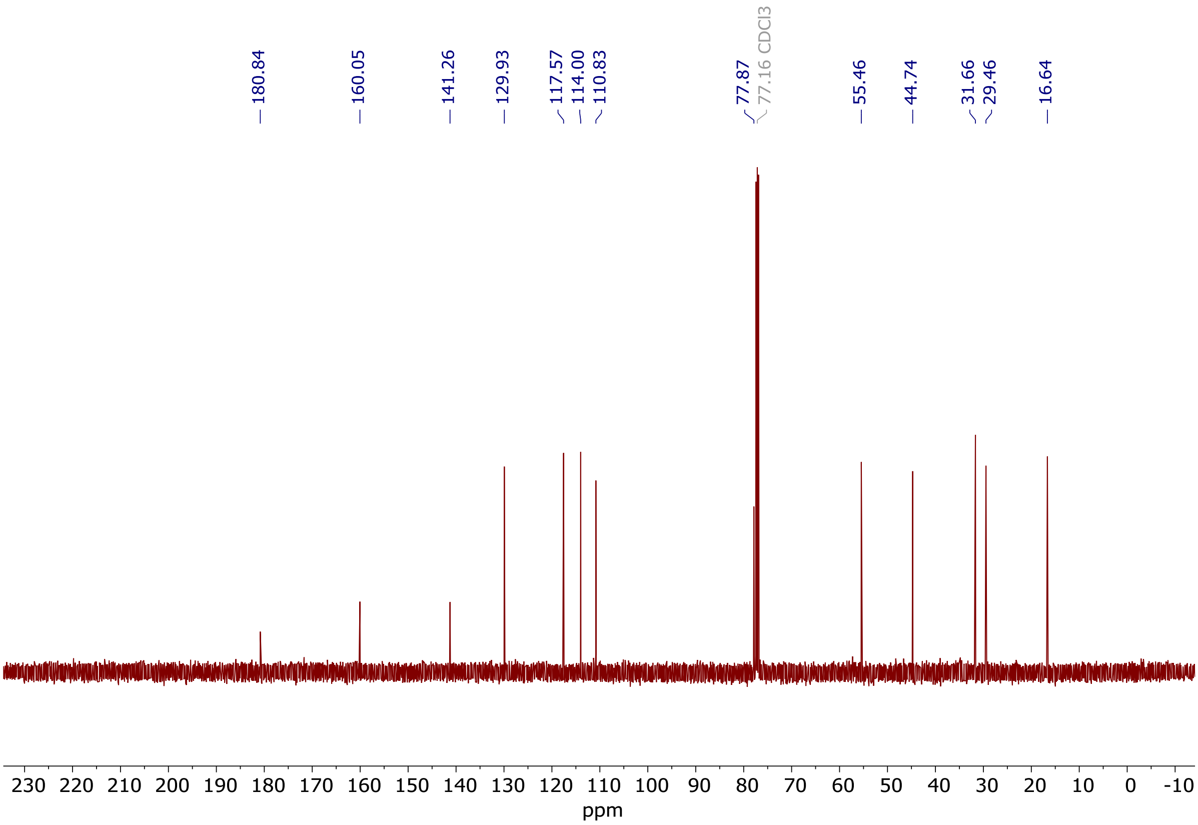

**$^{1}$H NMR** (500 MHz, $CDCl_3$) of **3q** see procedure

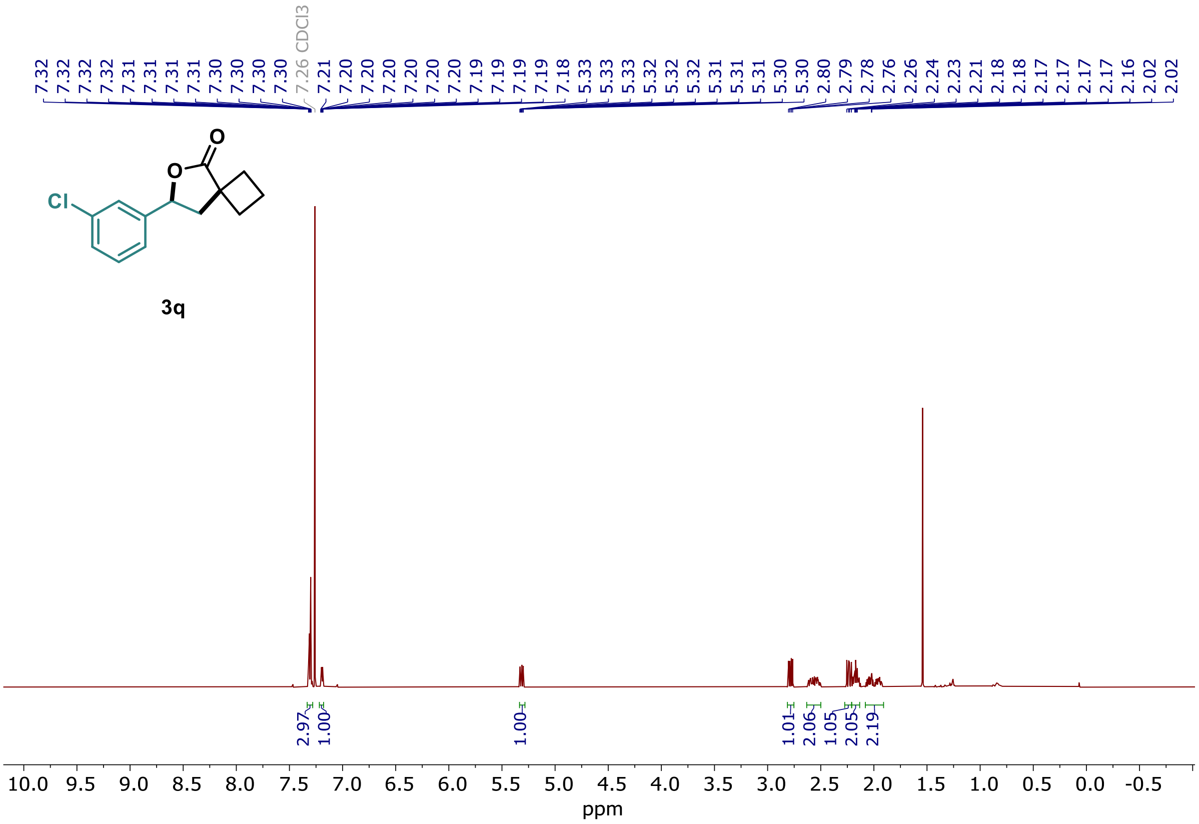


**$^{13}$C NMR** (126MHz, $CDCl_3$) of **3q**

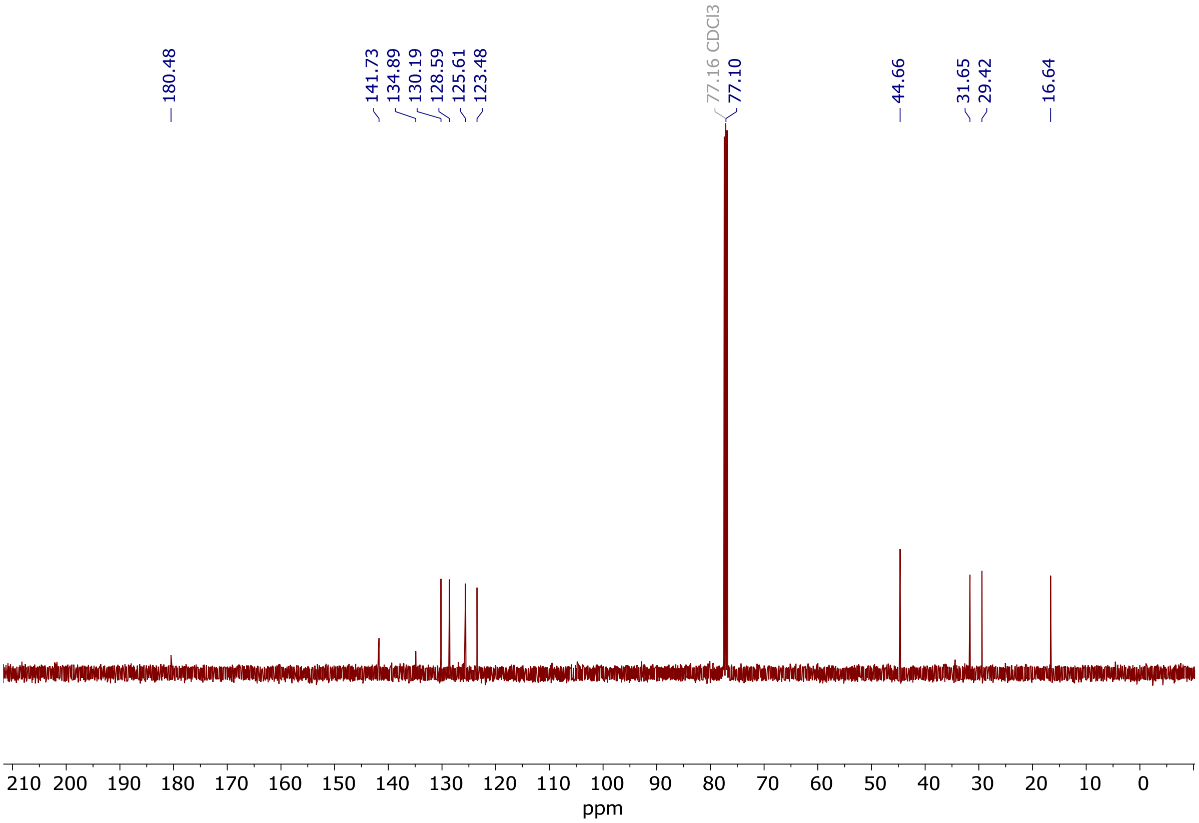

**$^{1}$H NMR** (599 MHz, $CDCl_3$) of **3r** see procedure

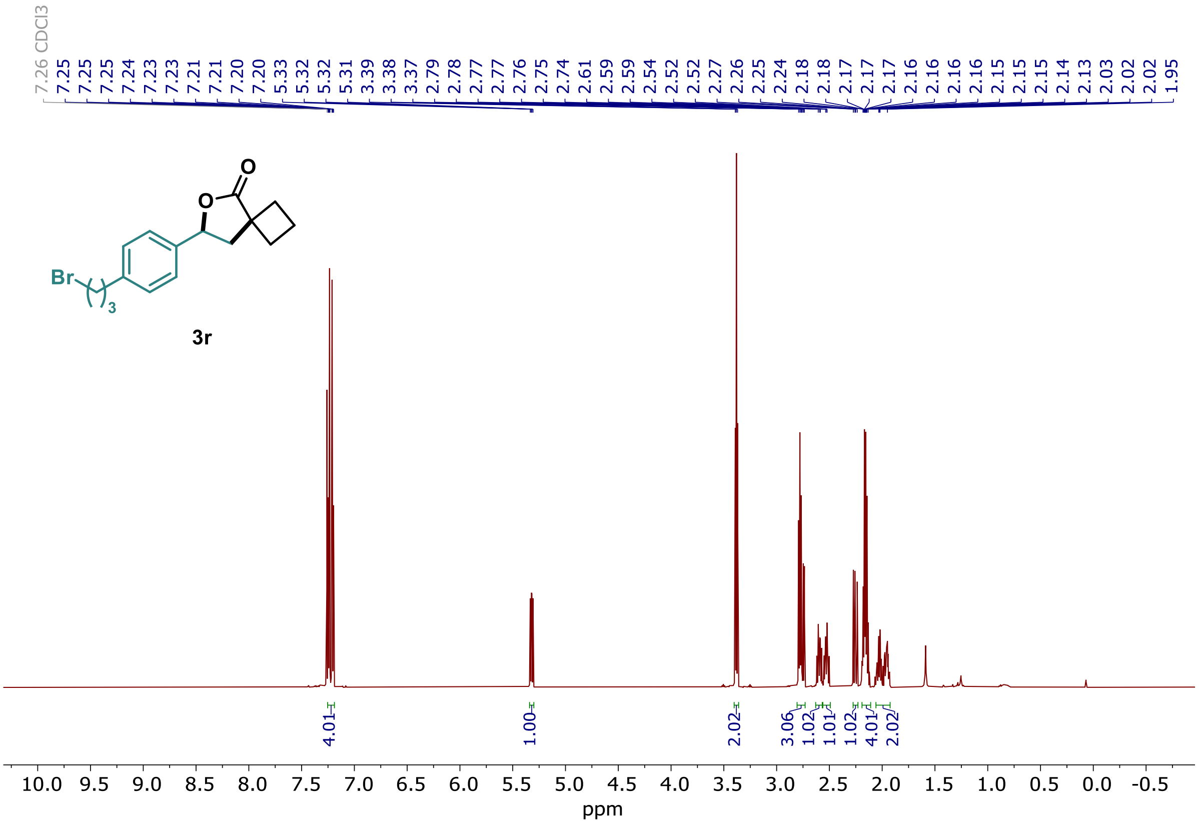


**$^{13}$C NMR** (151MHz, $CDCl_3$) of **3r**

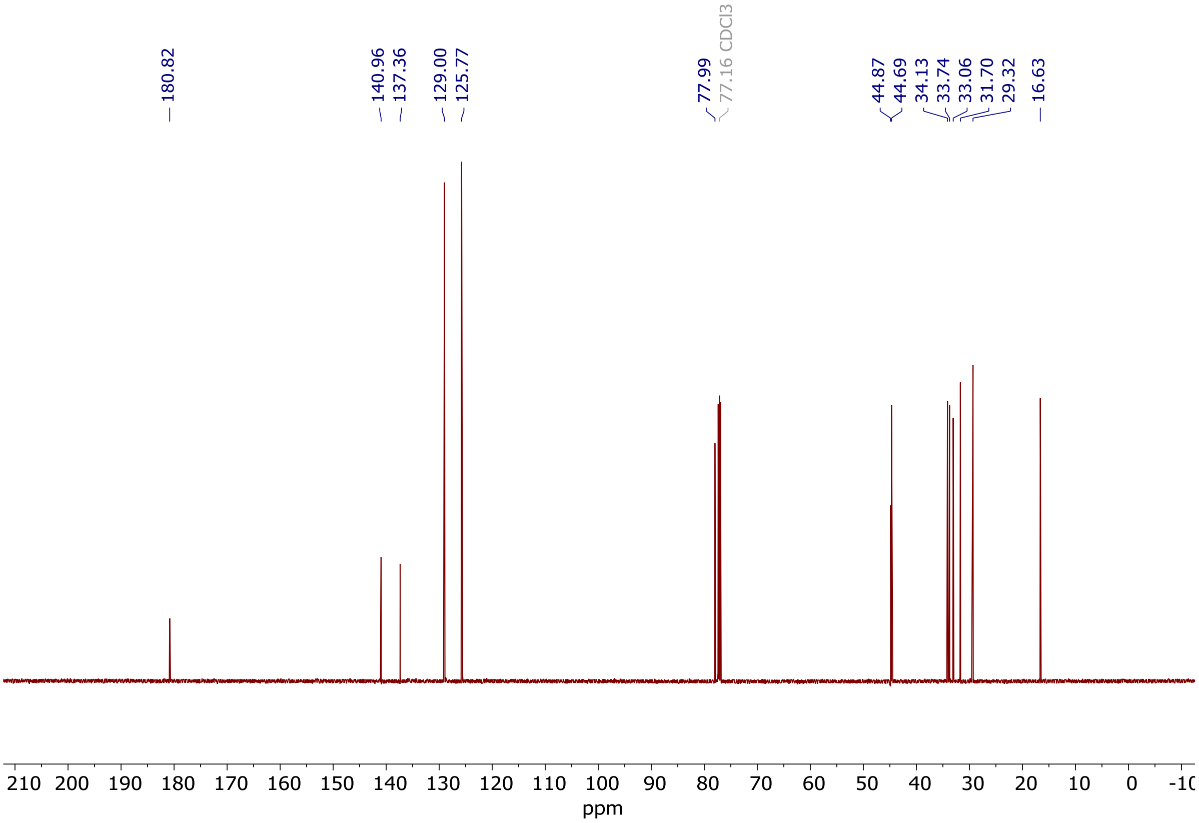

**$^{1}$H NMR** (500 MHz, $CDCl_3$) of **3s** see procedure

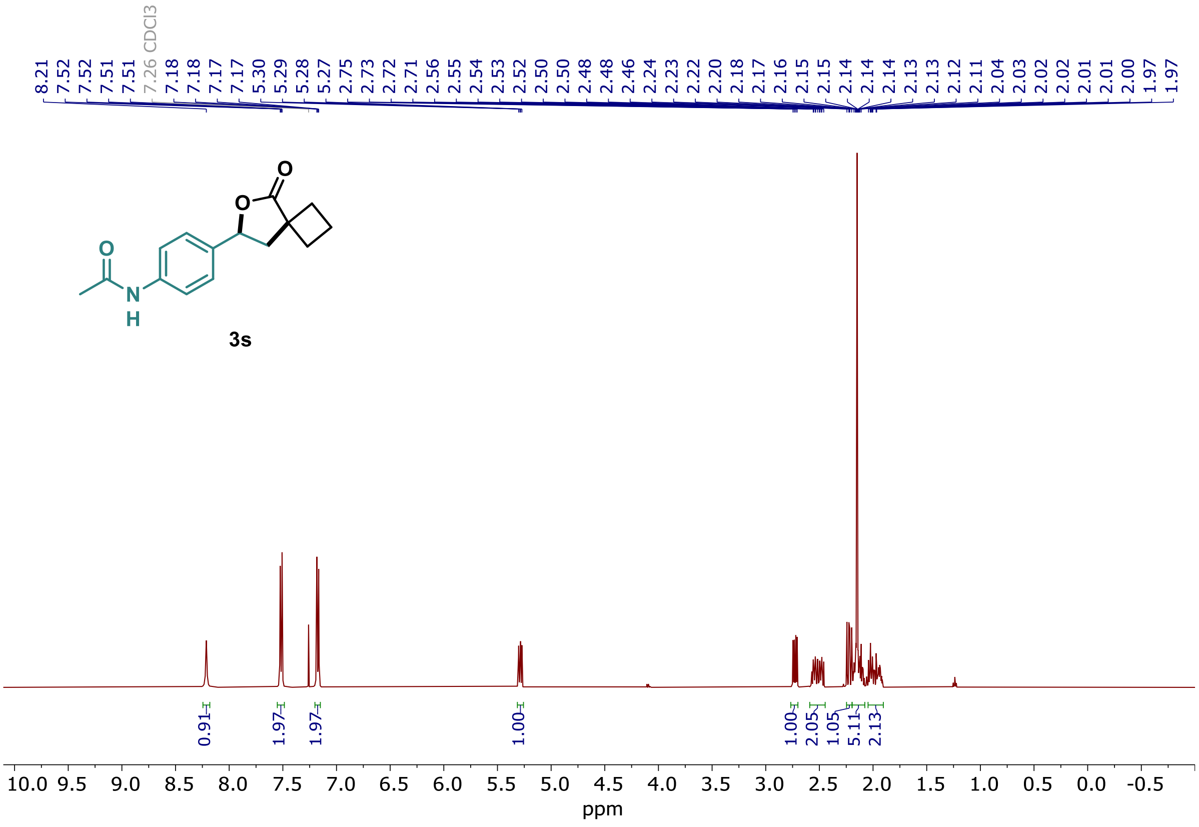


**$^{13}$C NMR** (126 MHz, $CDCl_3$) of **3s**

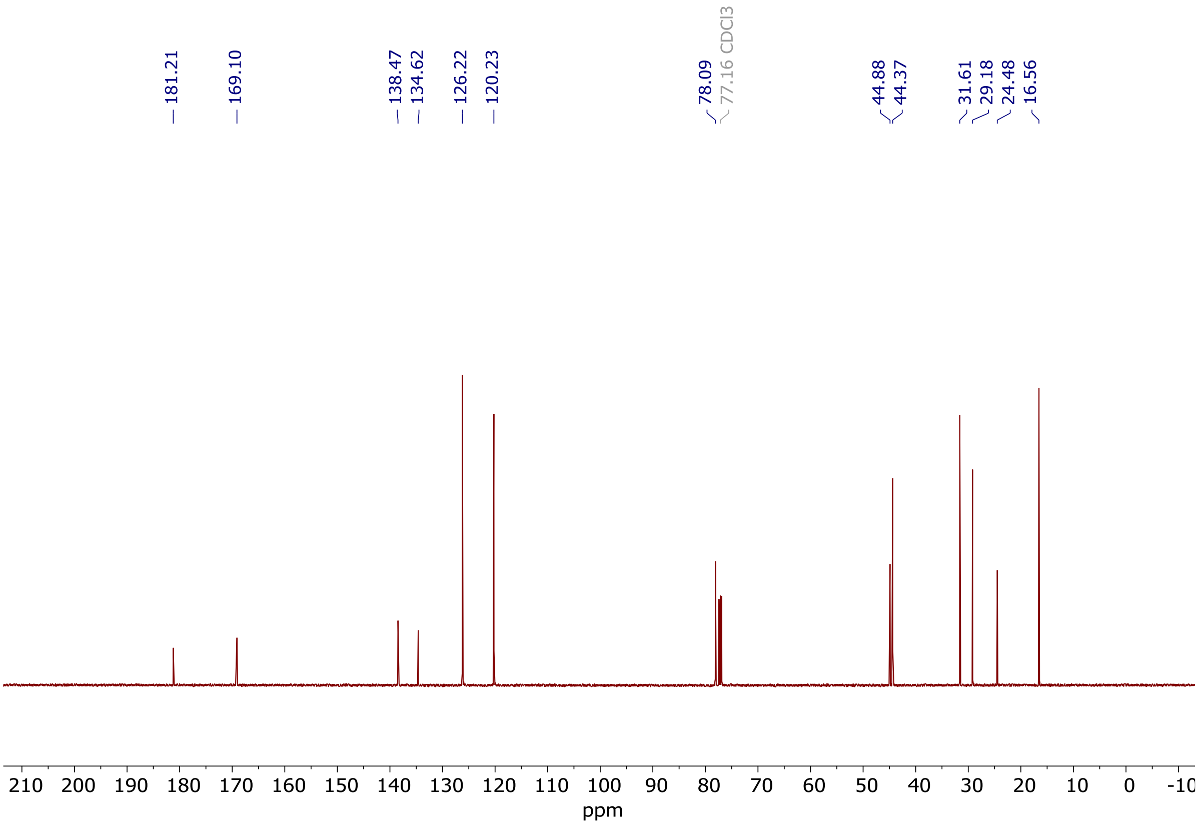

**$^{1}$H NMR** (599 MHz, CDCl₃) of **3t** see procedure

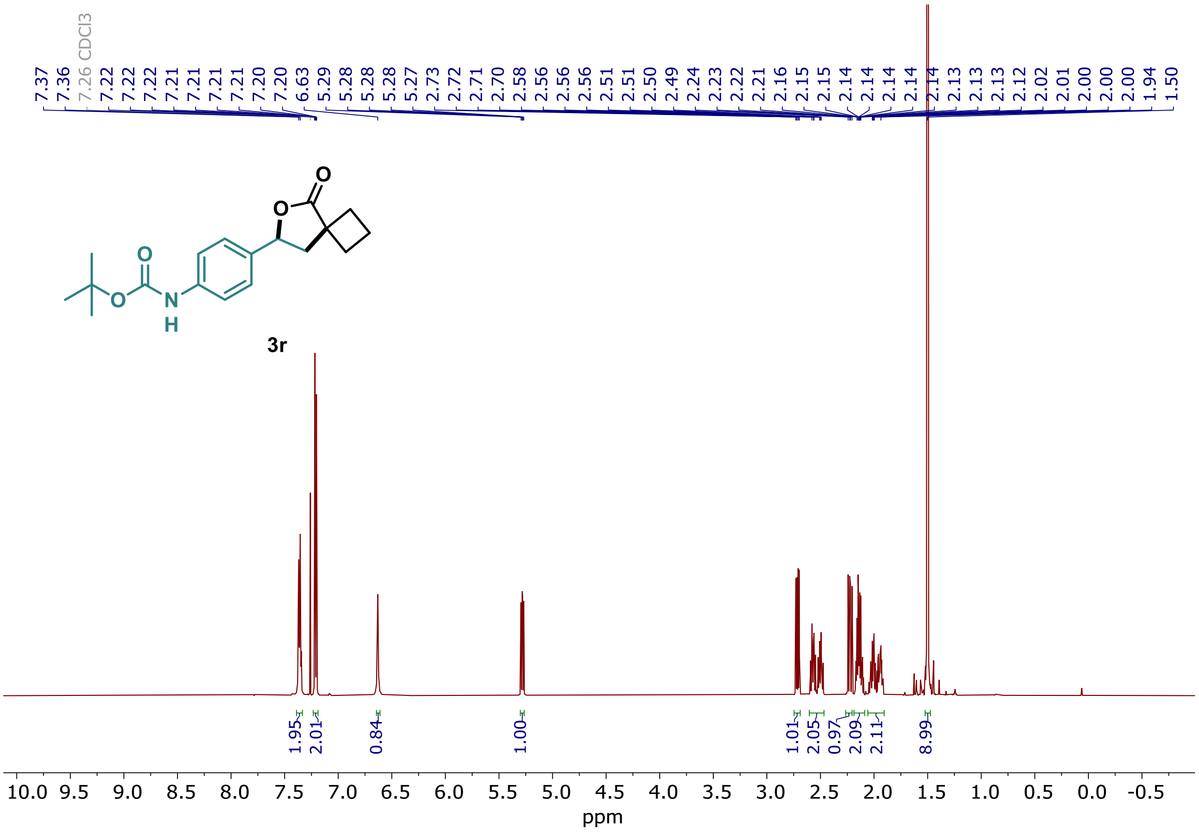


**$^{13}$C NMR** (151MHz, CDCl₃) of **3t**

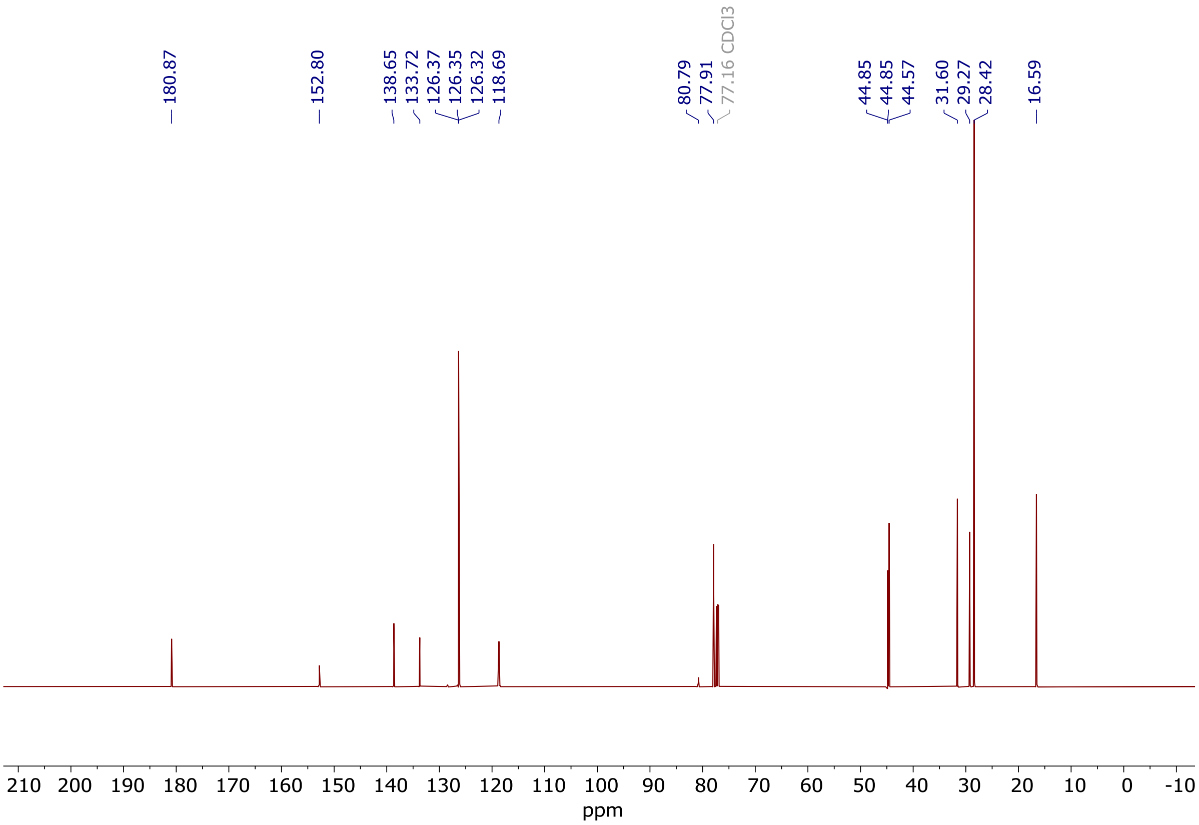

**$^{1}$H NMR** (599 MHz, $CDCl_3$) of **3u** see procedure

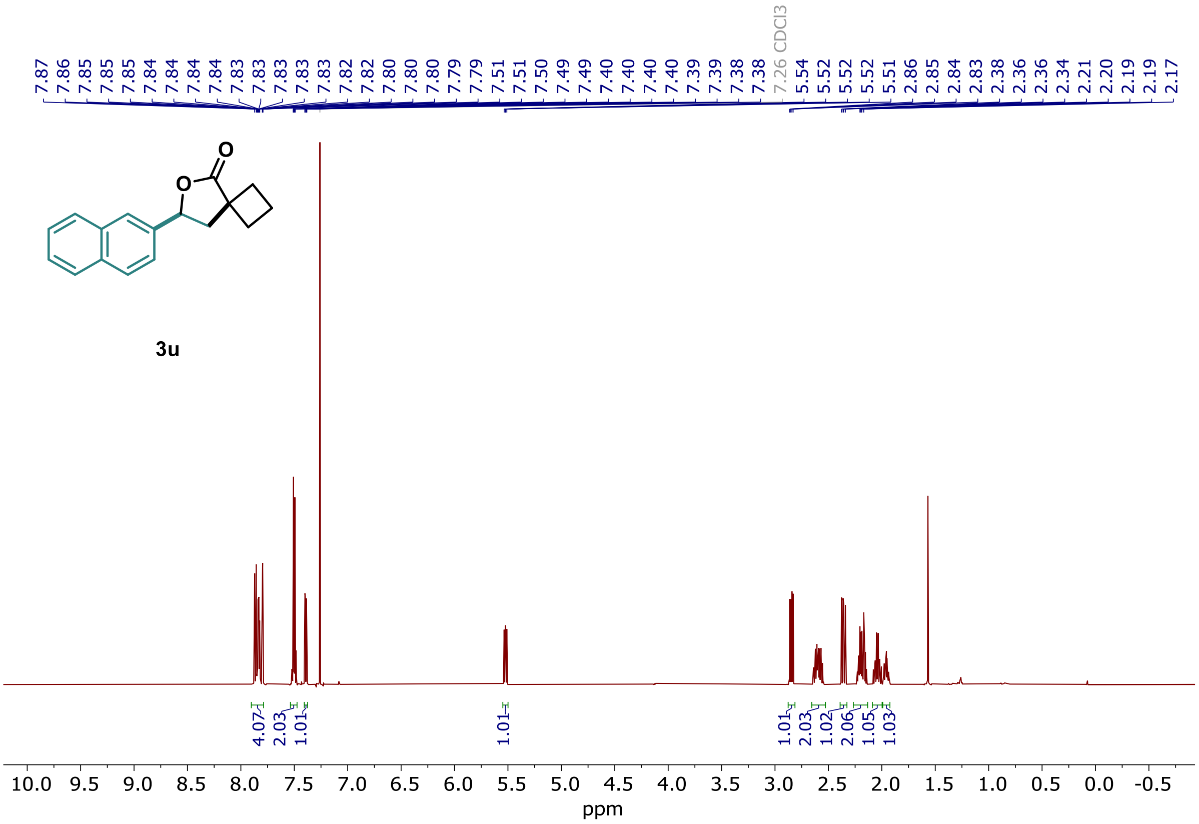


**$^{13}$C NMR** (151MHz, $CDCl_3$) of **3u**

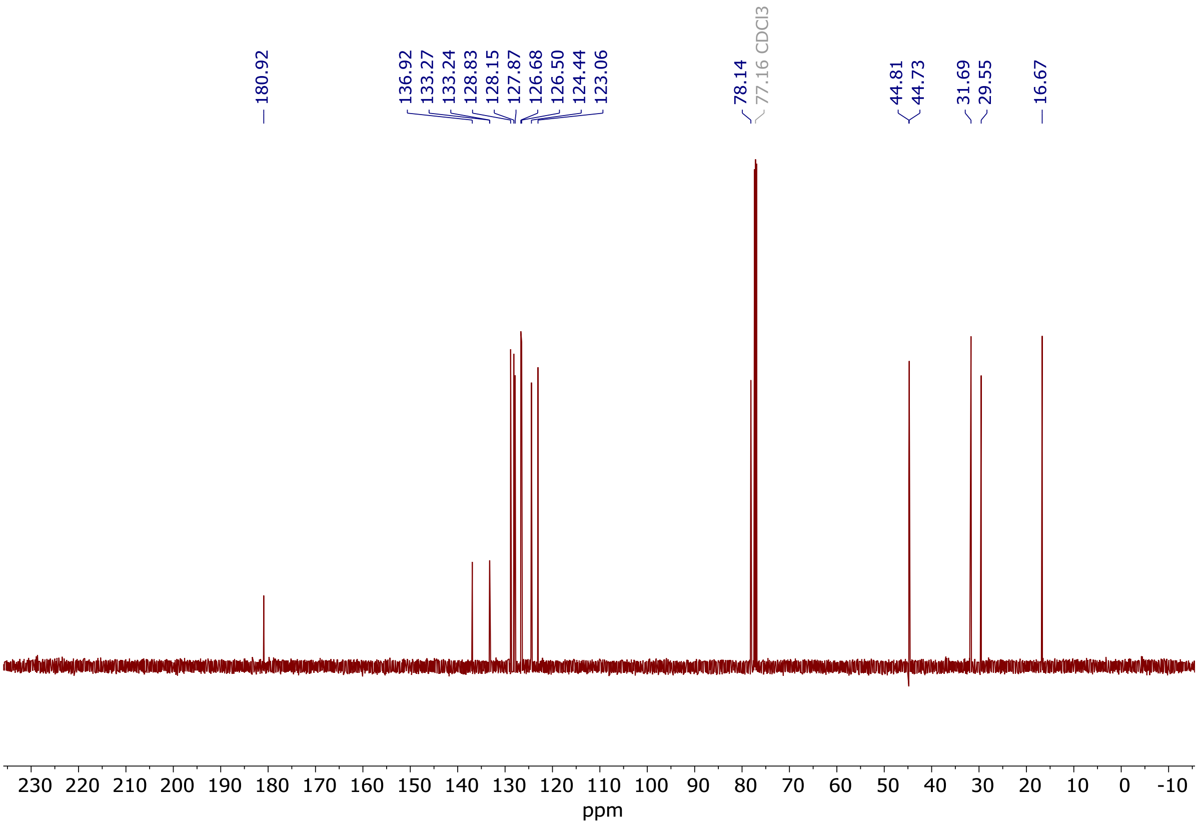

**$^{1}$H NMR** (599 MHz, CDCl3) of **3v** see procedure

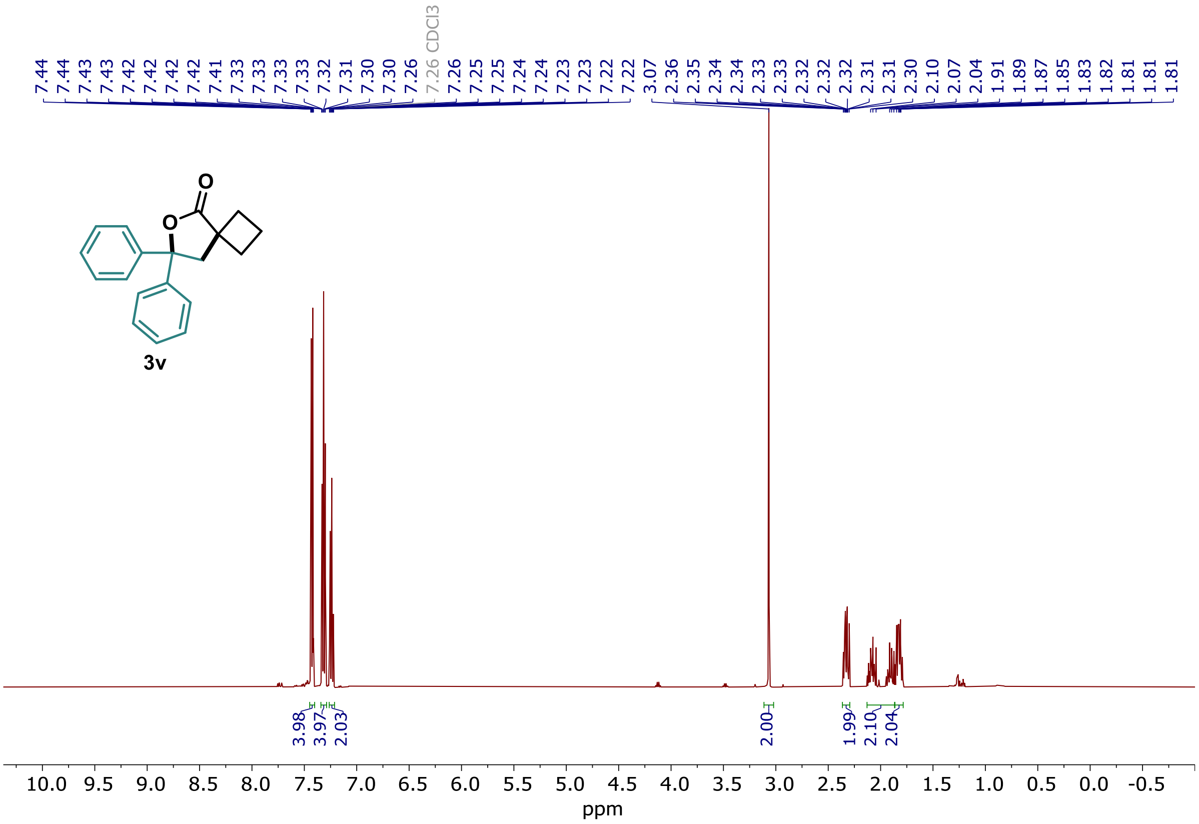


**$^{13}$C NMR** (151MHz, CDCl3) of **3v**

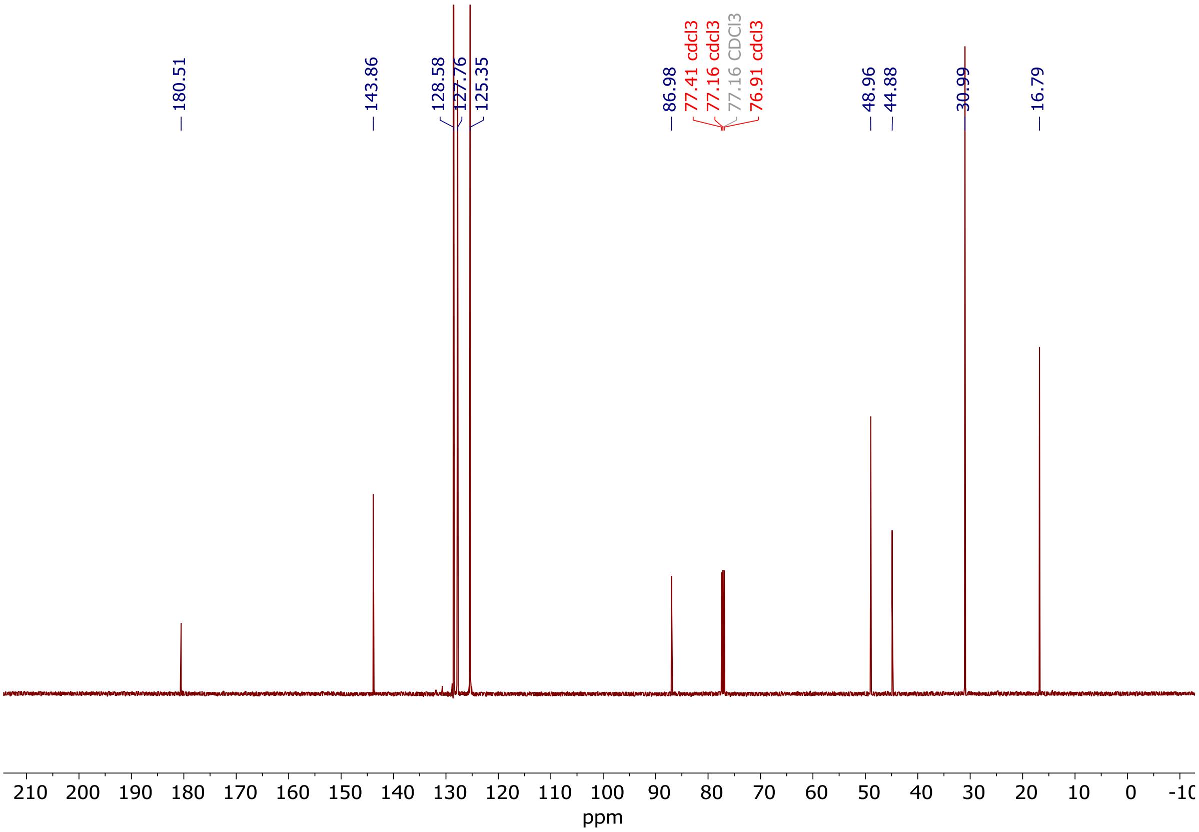

**$^{1}$H NMR** (500 MHz, $CDCl_3$) of **3w** see procedure

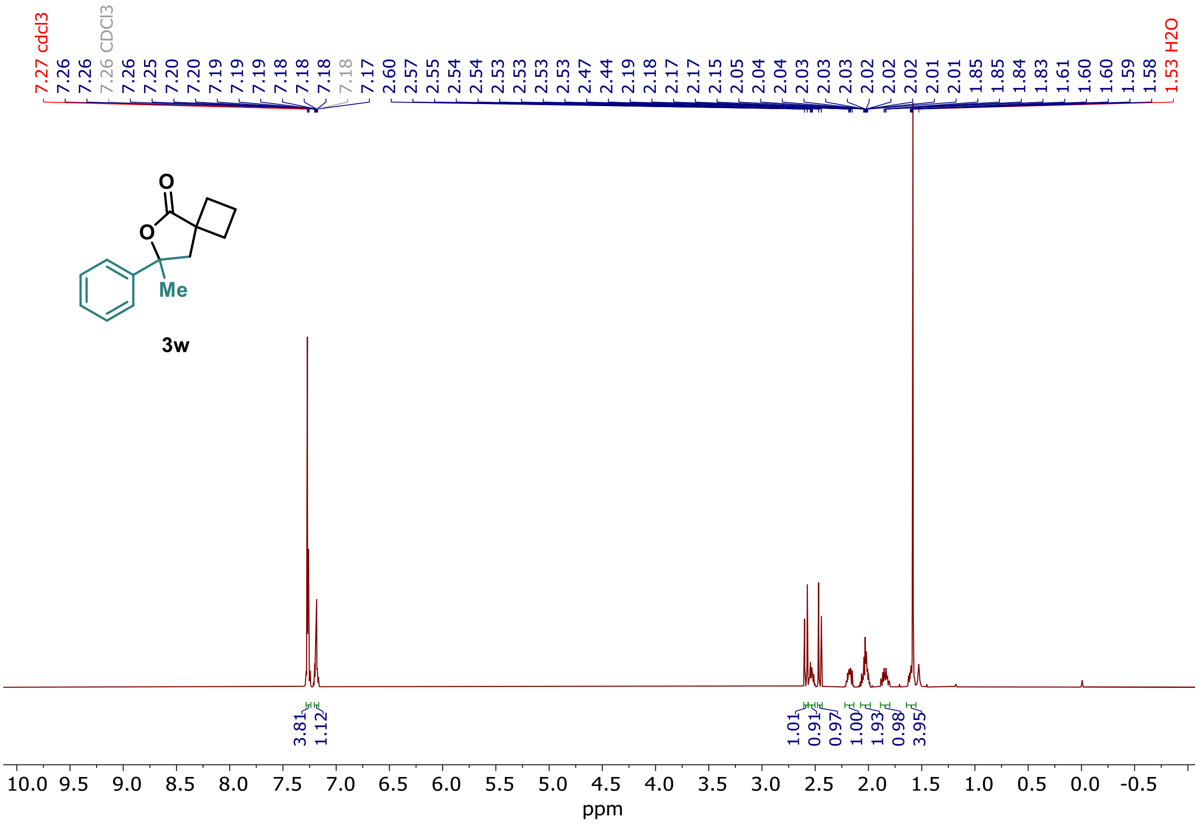


**$^{13}$C NMR** (126MHz, $CDCl_3$) of **3w**

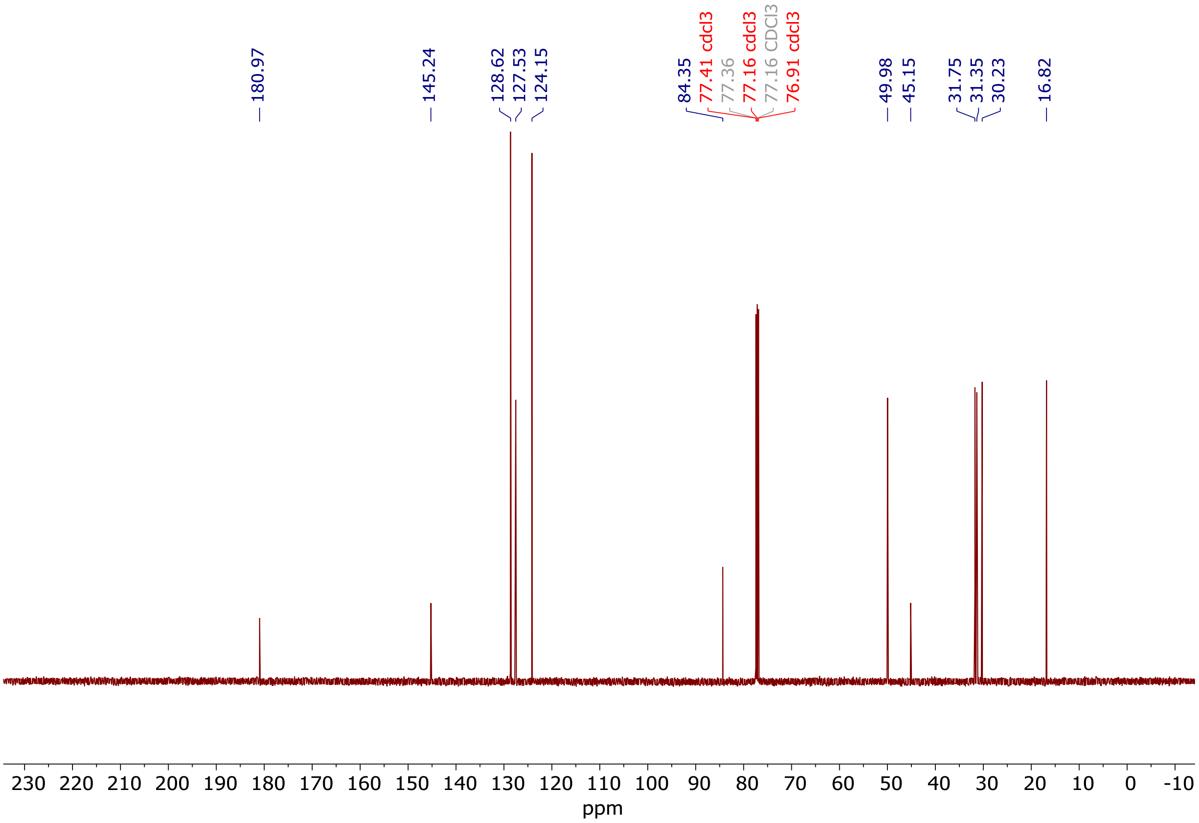

**$^{1}$H NMR** (599 MHz, $CDCl_3$) of **3x** see procedure

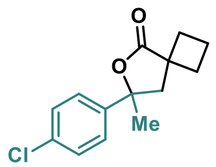


**3w**

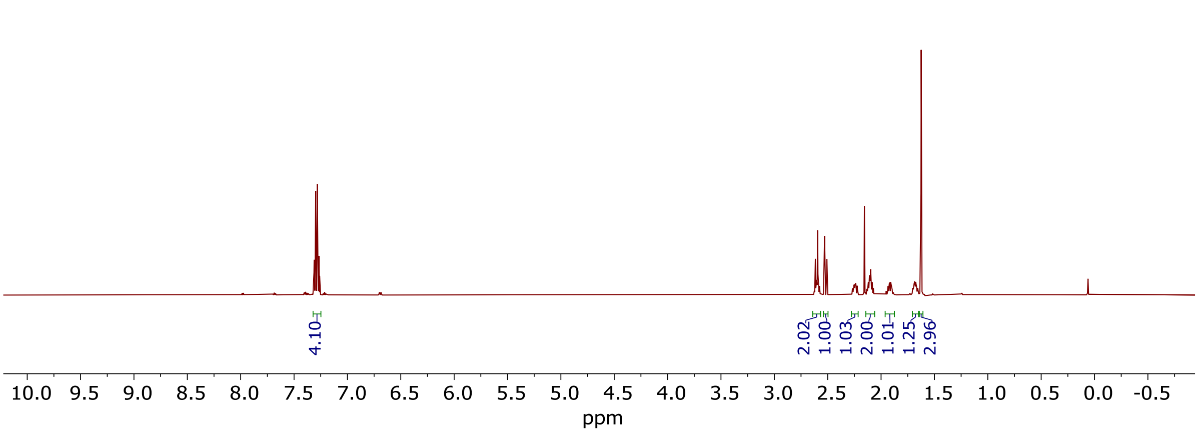


**$^{13}$C NMR** (151MHz, $CDCl_3$) of **3x**

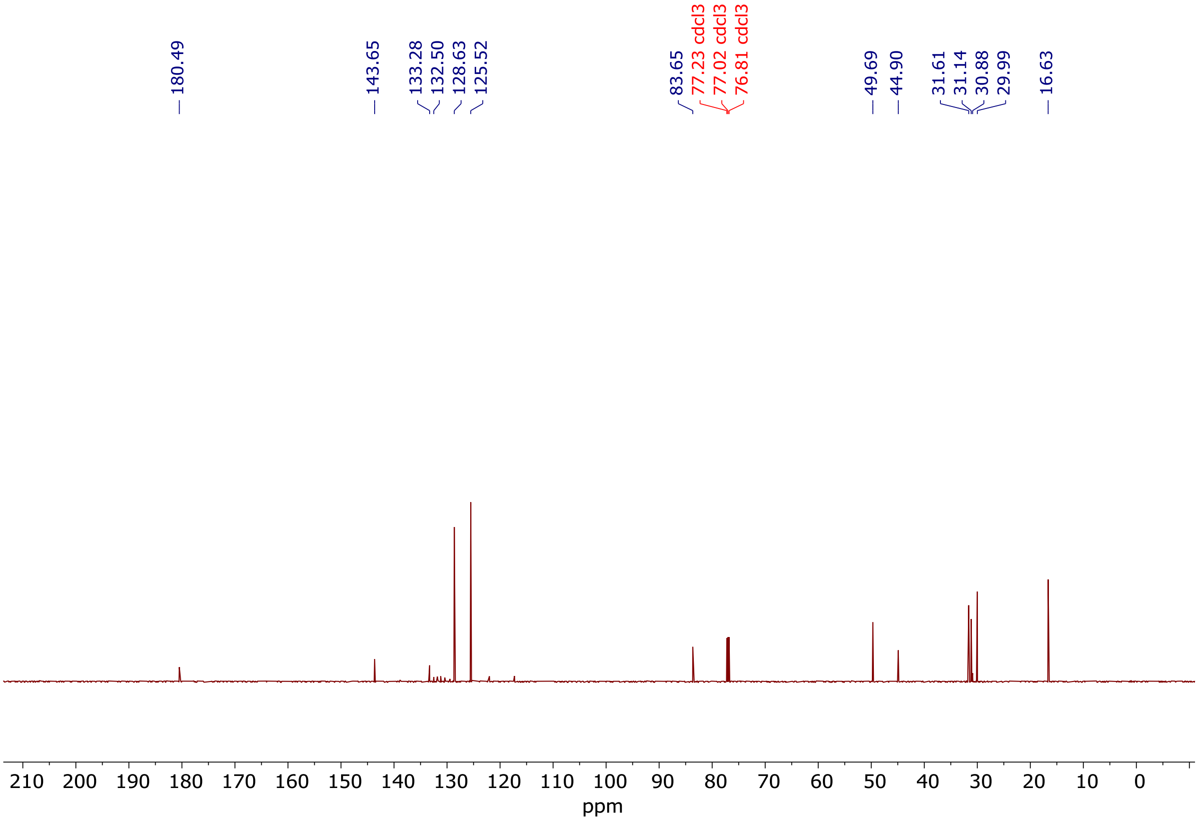

**$^{1}$H NMR** (599 MHz, CDCl$_3$) of **3y** see procedure

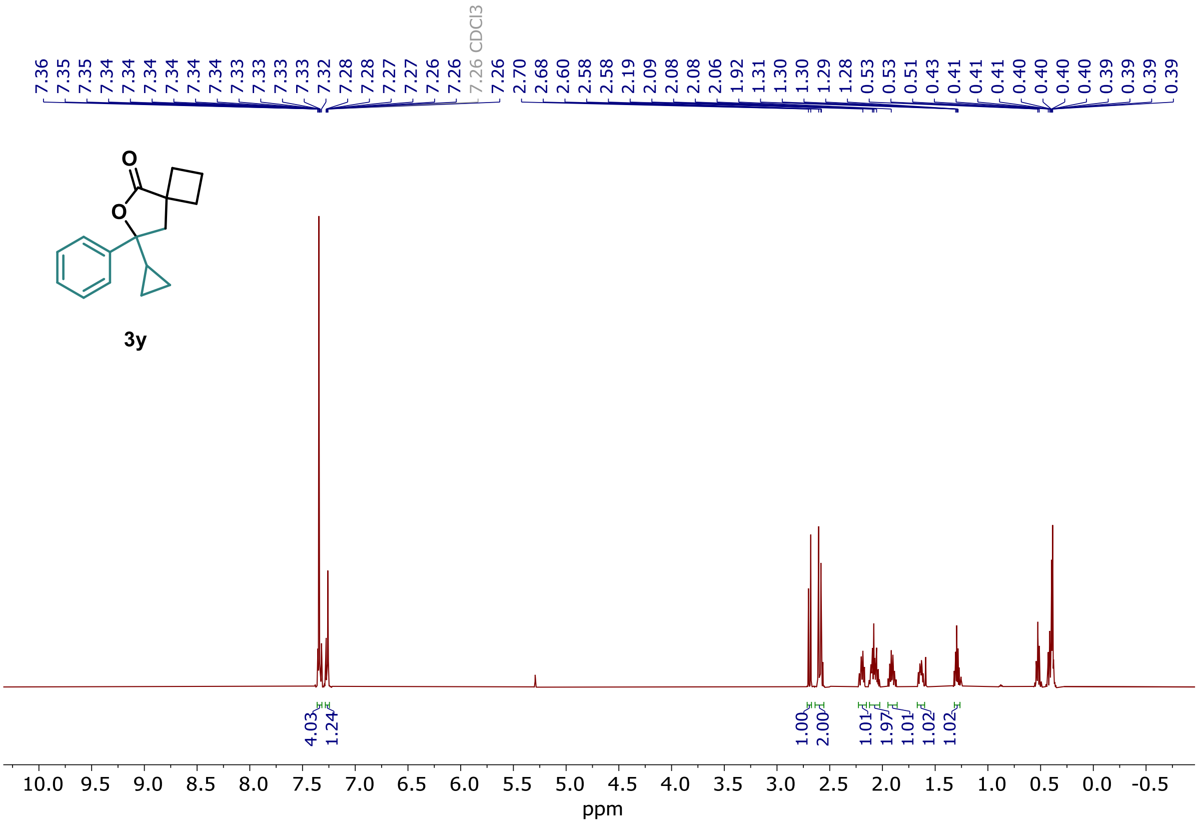


**$^{13}$C NMR** (151MHz, CDCl$_3$) of **3y**

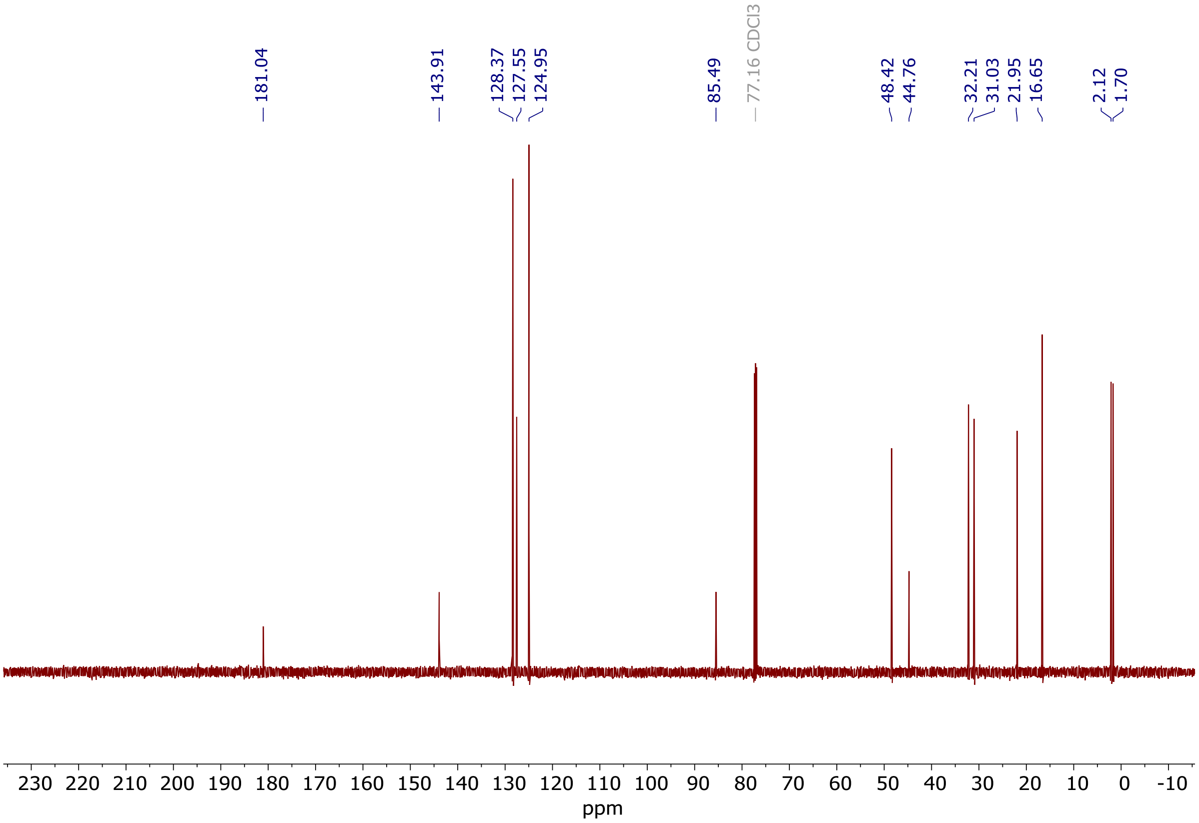

**$^{1}$H NMR** (500 MHz, $CDCl_3$) of **3z** see procedure

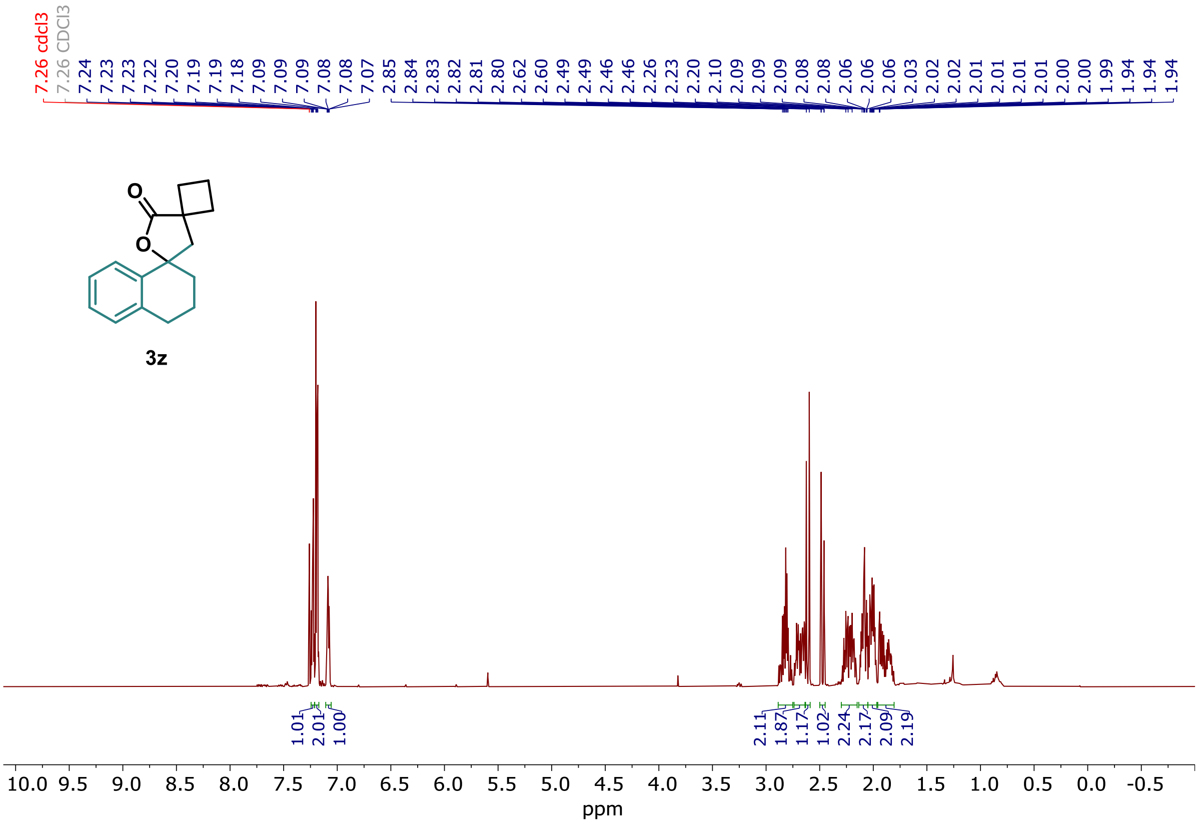


**$^{13}$C NMR** (126 MHz, $CDCl_3$) of **3z**

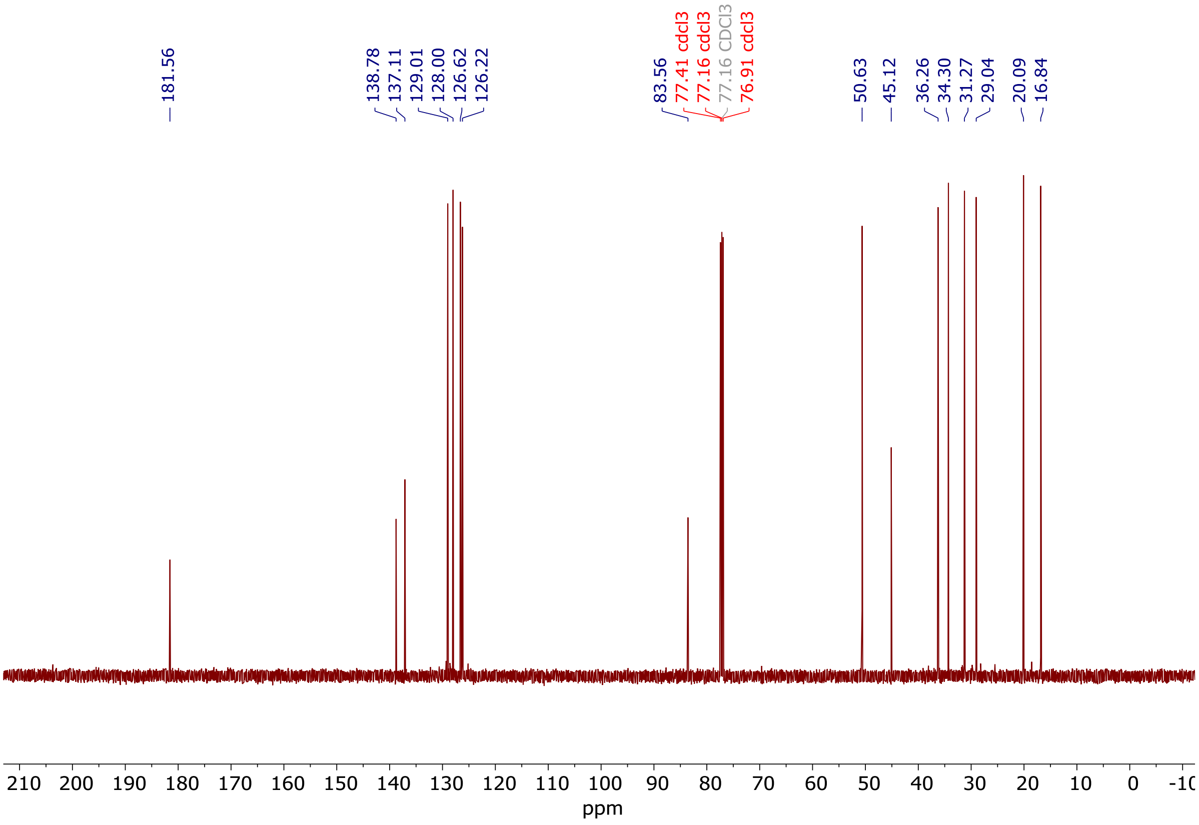

**$^{1}$H NMR** (500 MHz, CDCl$_3$) of **3aa** see procedure

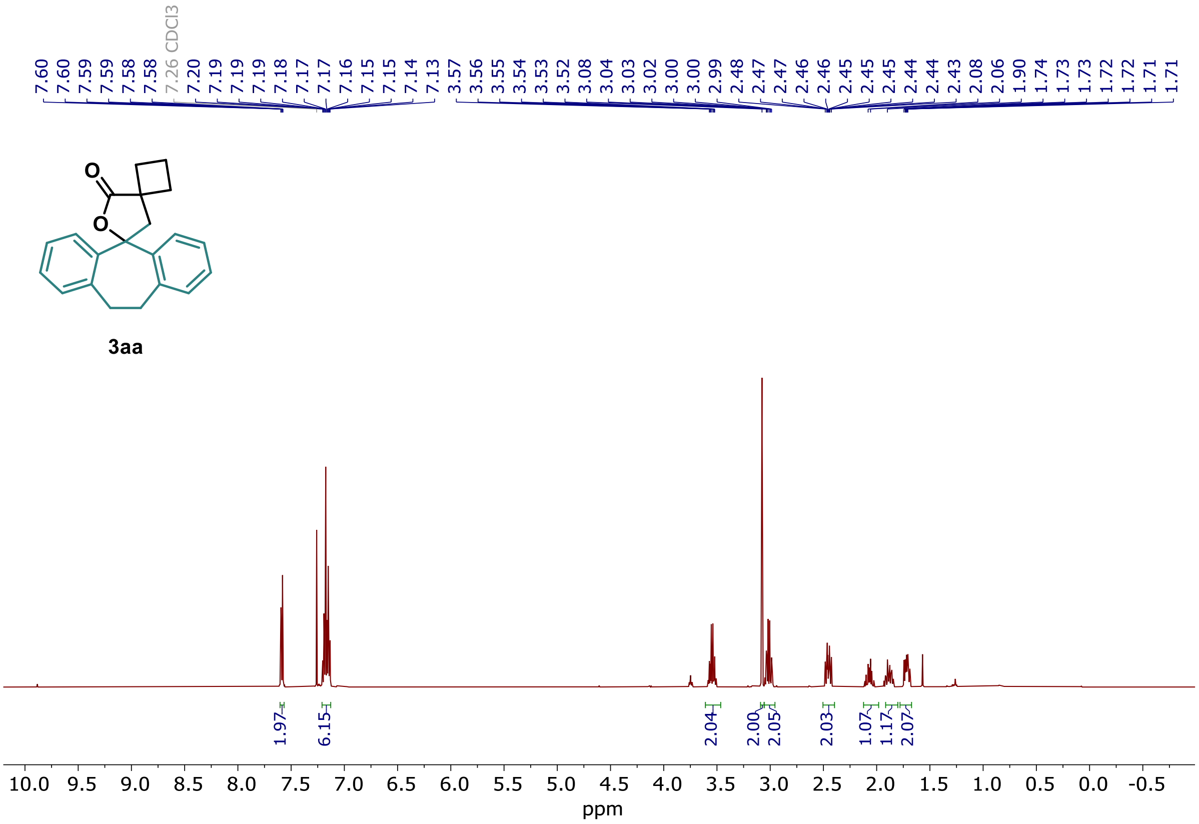


**$^{13}$C NMR** (126MHz, CDCl$_3$) of **3aa**

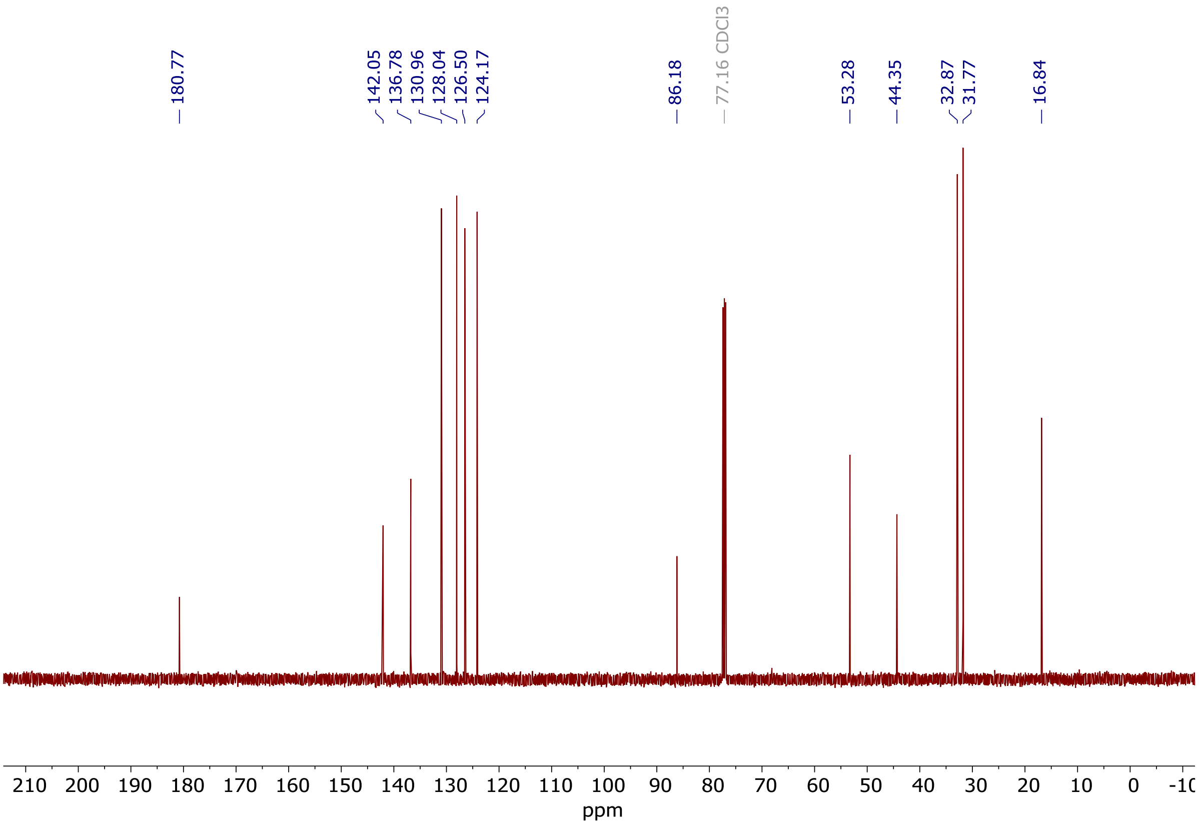

**$^{1}$H NMR** (599 MHz, CDCl3) of **3ab** see procedure

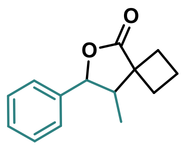

**3ab**

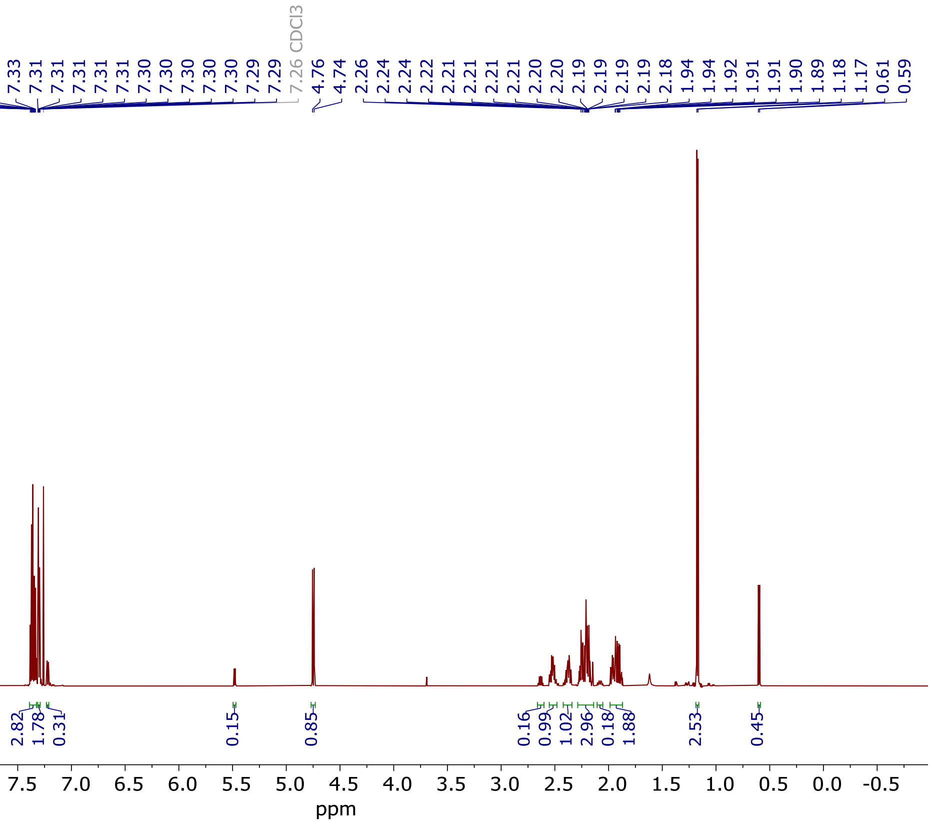


**$^{13}$C NMR** (151MHz, CDCl3) of **3ab**

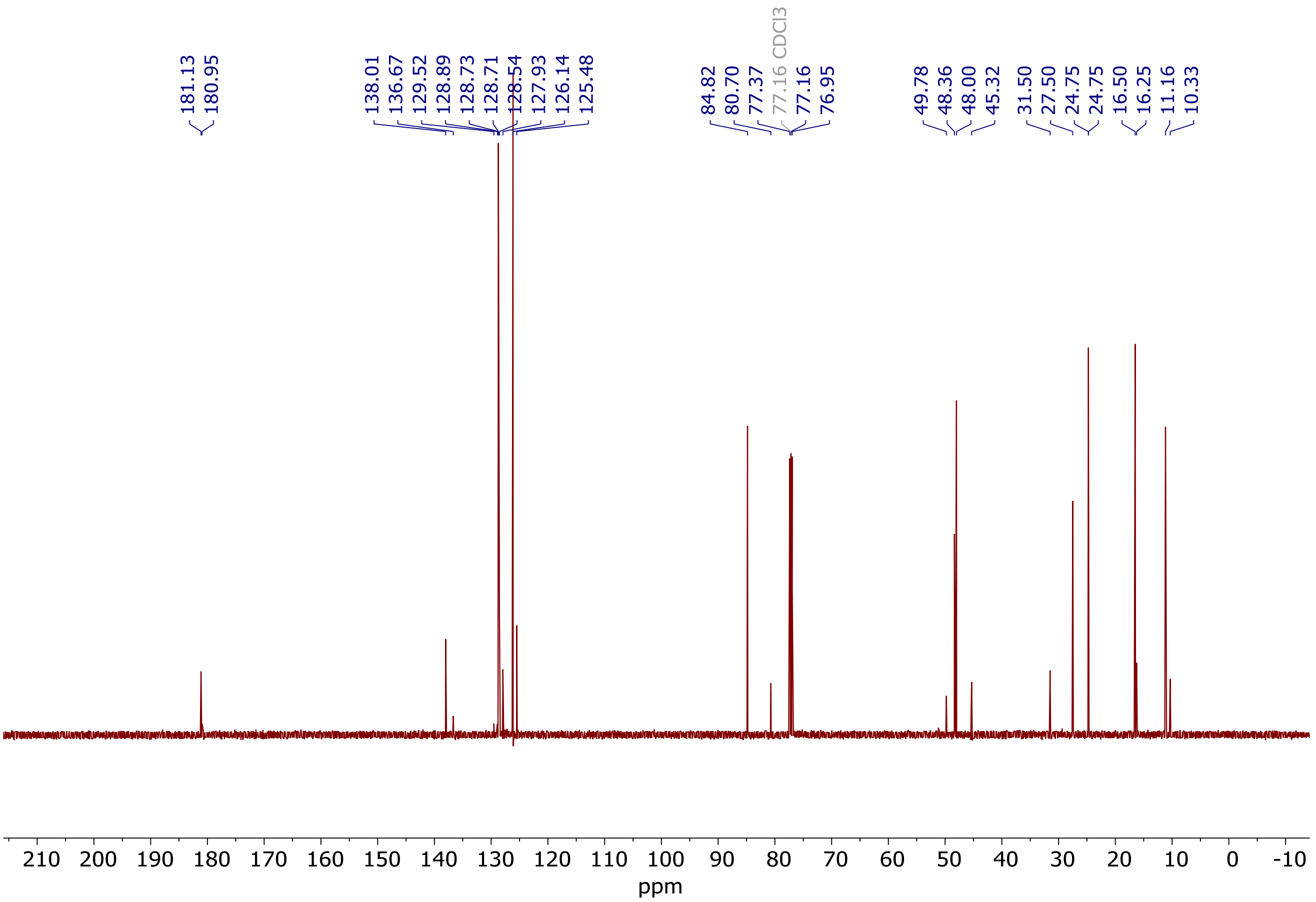

**$^{1}$H NMR** (599 MHz, $CDCl_3$) of **3ac** see procedure

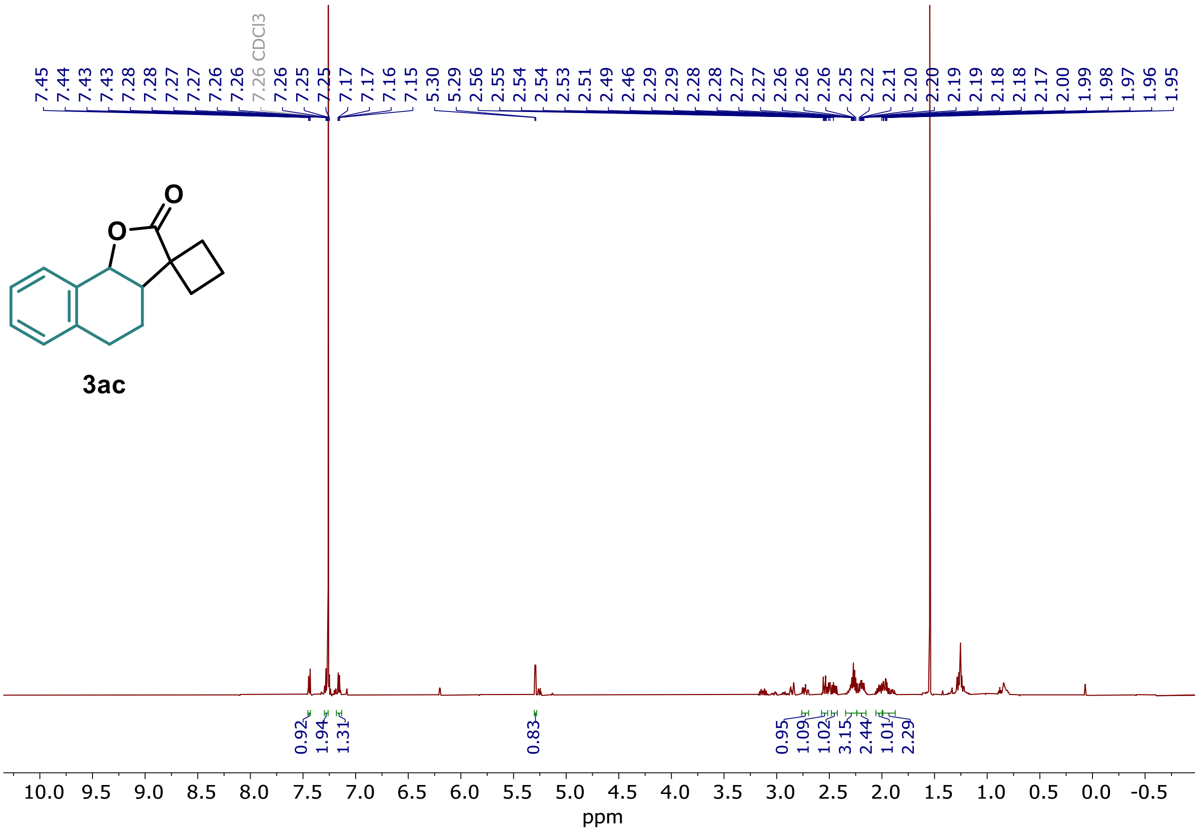

**3ac**

**$^{13}$C NMR** (151MHz, $CDCl_3$) of **3ac**

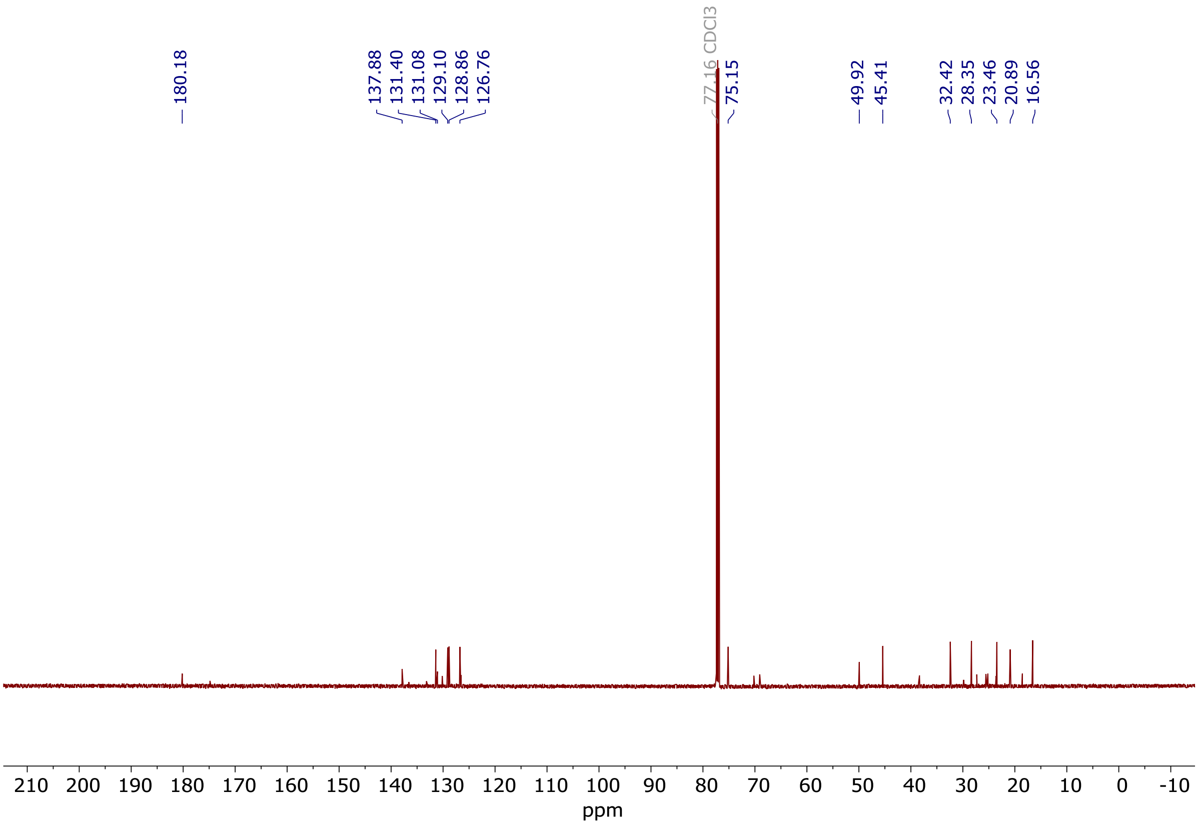

**$^{1}$H NMR** (500 MHz, $CDCl_3$) of **3ad** see procedure

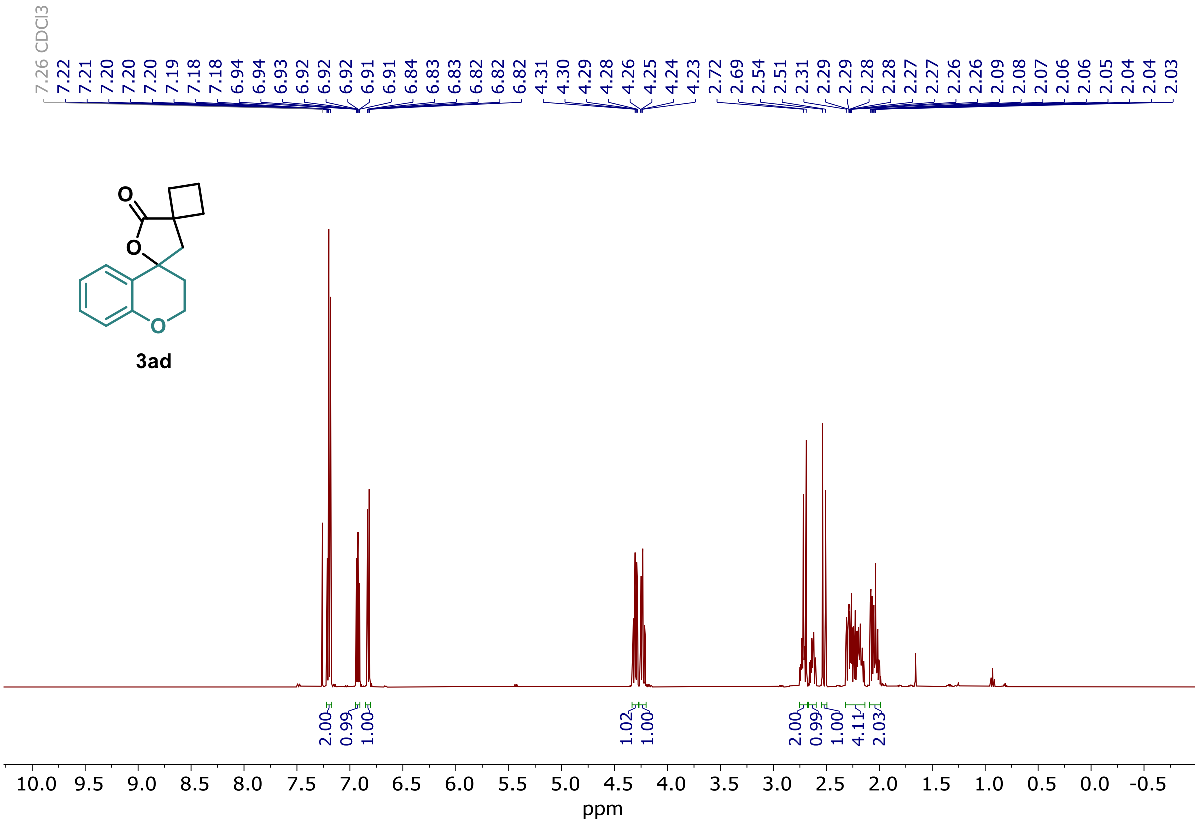

**$^{13}$C NMR** (126MHz, $CDCl_3$) of **3ad**

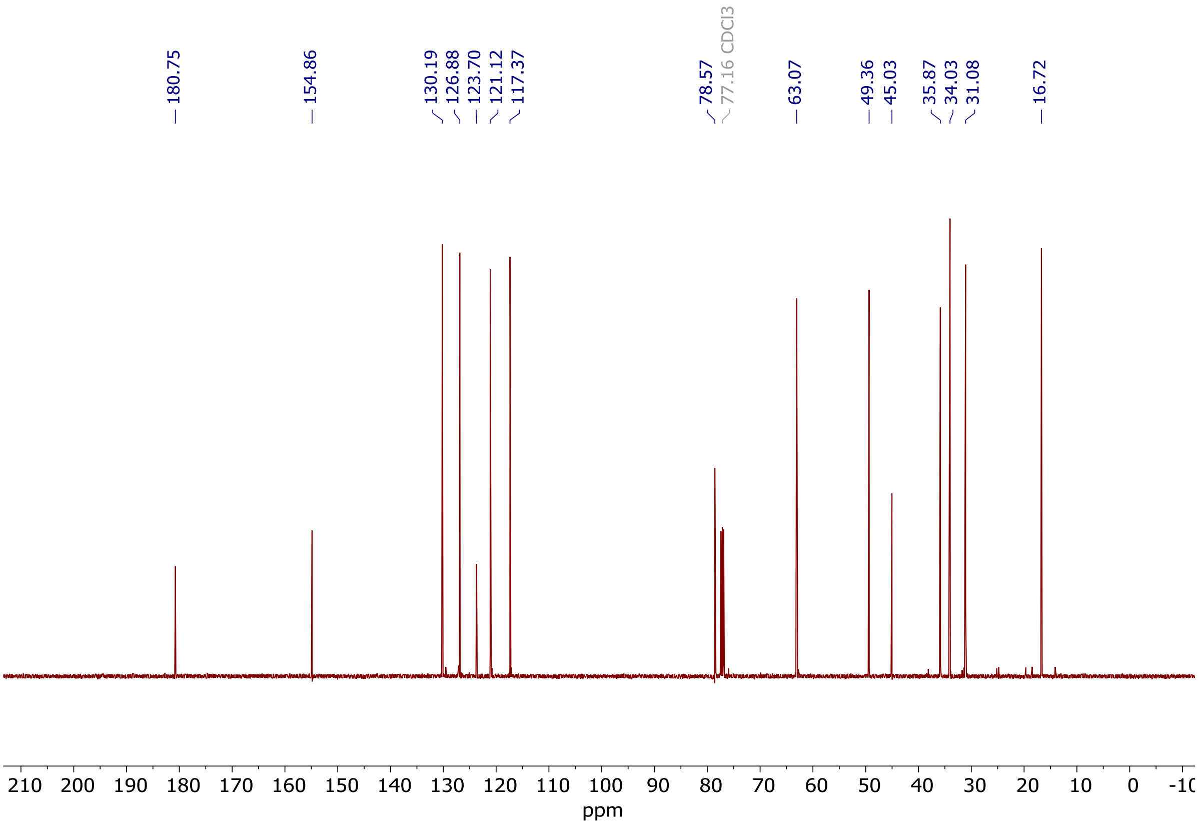

**$^{1}$H NMR** (599 MHz, $CDCl_3$) of **3ae** see procedure

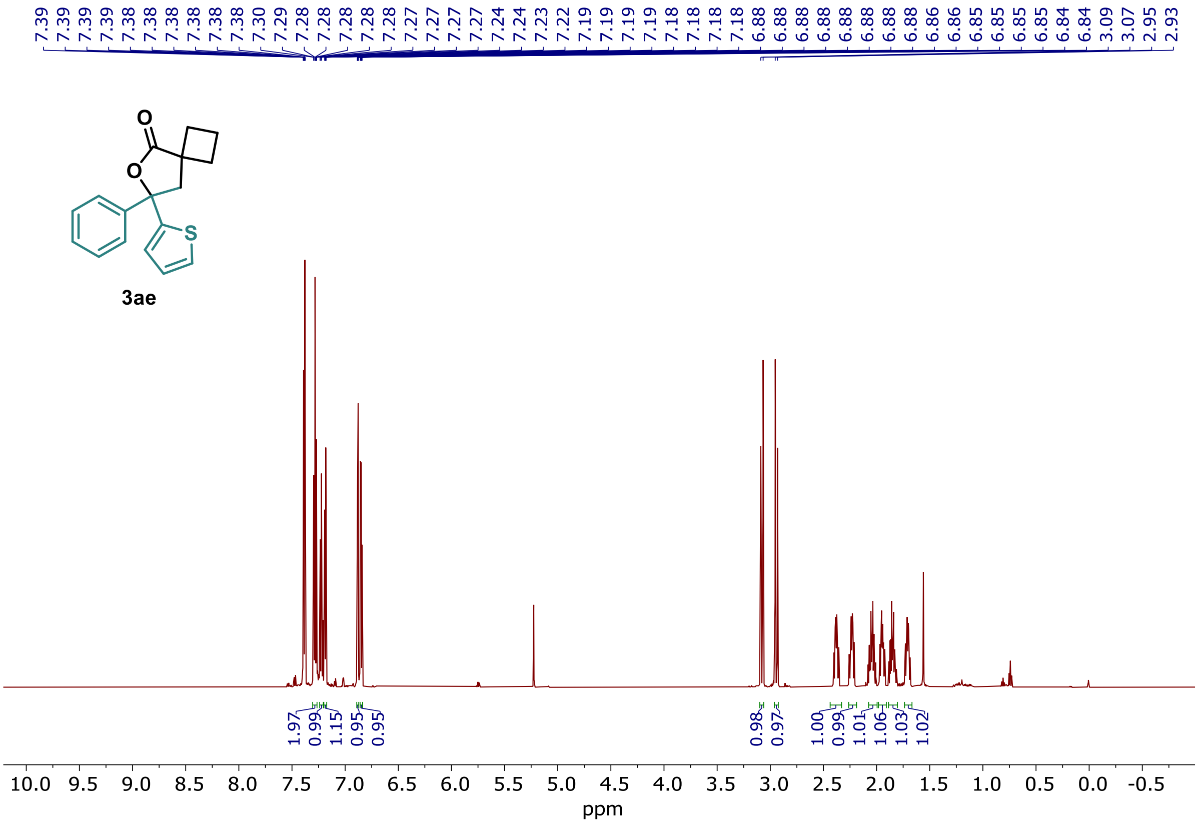


**$^{13}$C NMR** (151MHz, $CDCl_3$) of **3ae**

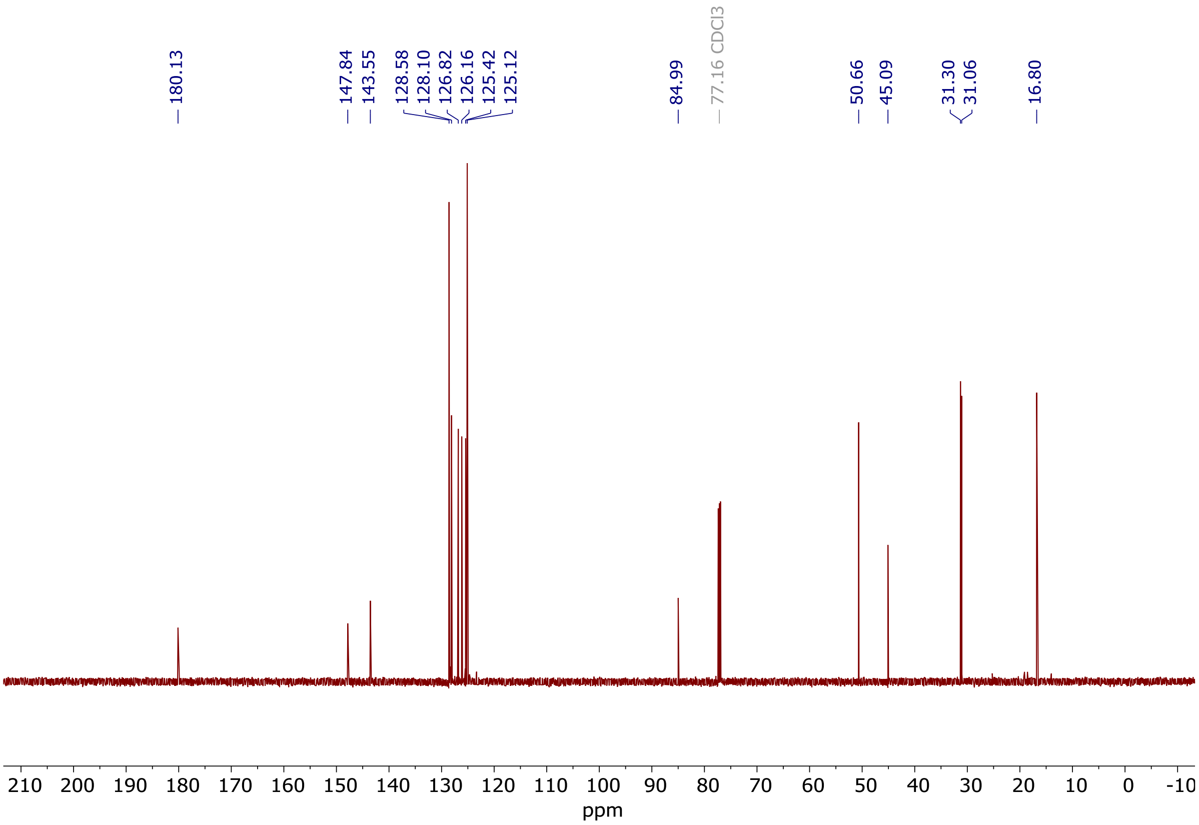

**¹H NMR** (599 MHz, $CDCl_3$) of **3af** see procedure

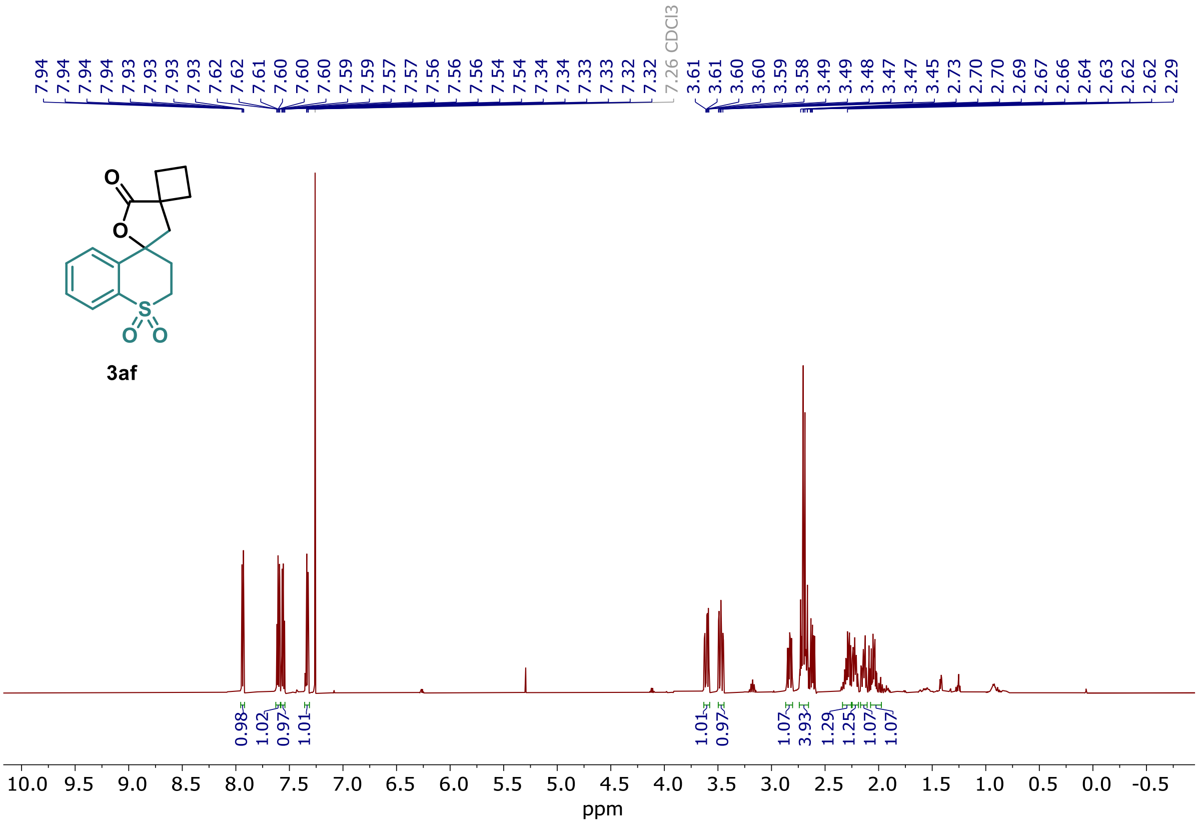


**¹³C NMR** (151MHz, $CDCl_3$) of **3af**

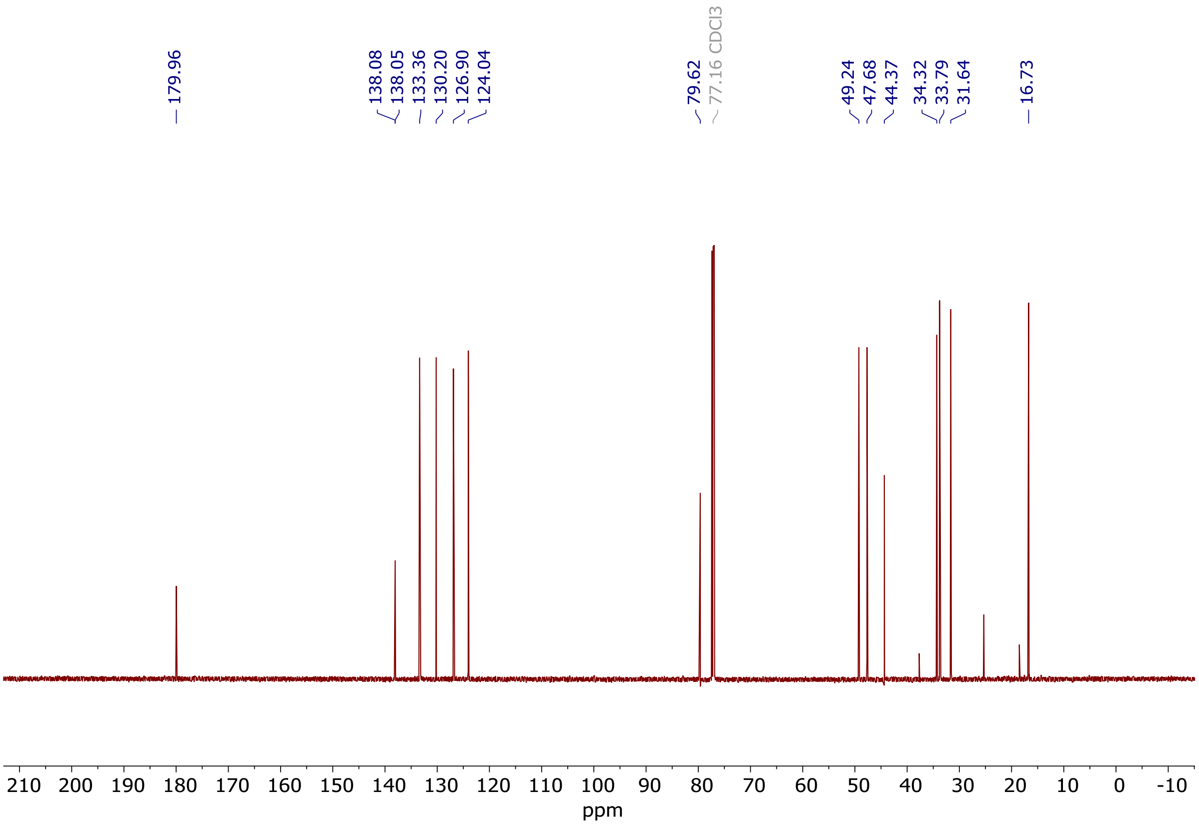

**¹H NMR** (599 MHz, $CDCl_3$) of **3ag** see procedure

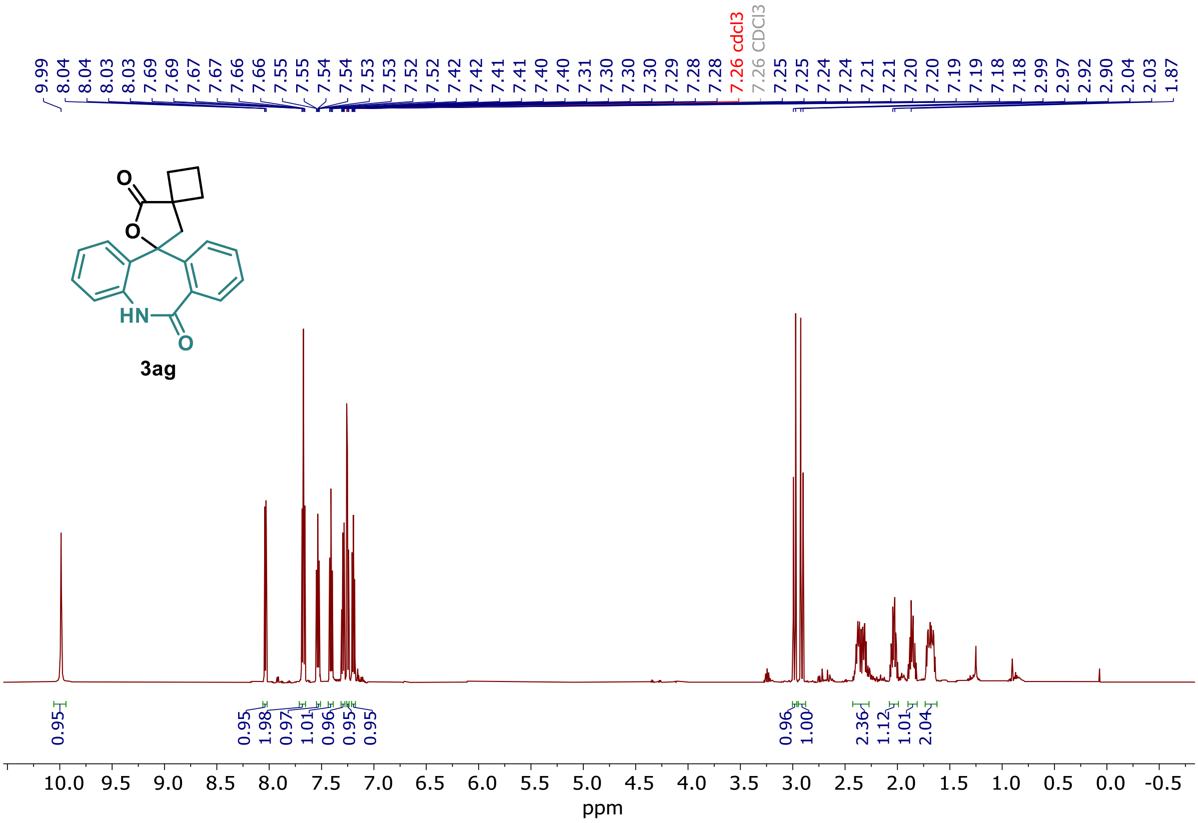


**¹³C NMR** (151MHz, $CDCl_3$) of **3ag**

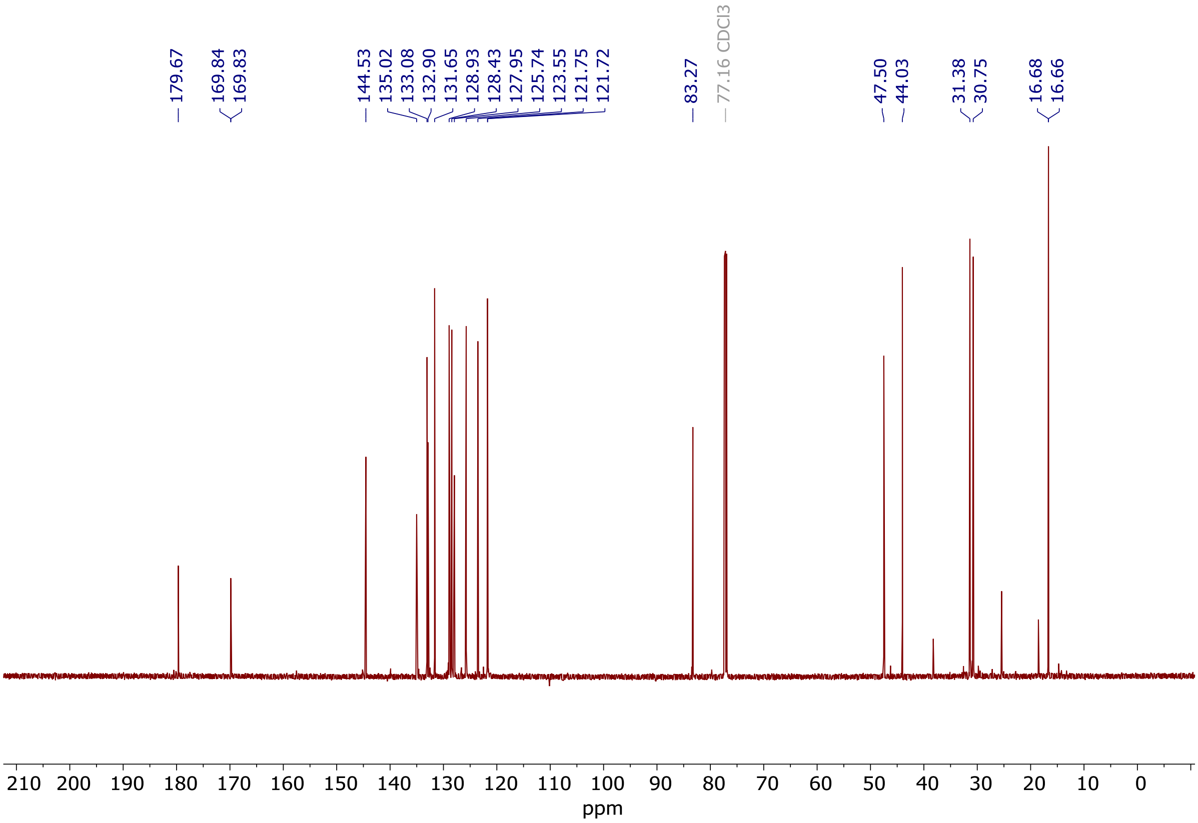

[1]**H NMR** (500MHz, $CDCl_3$) of **4b**see procedure

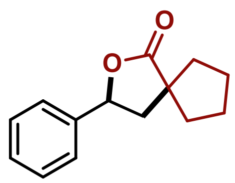

**4b**

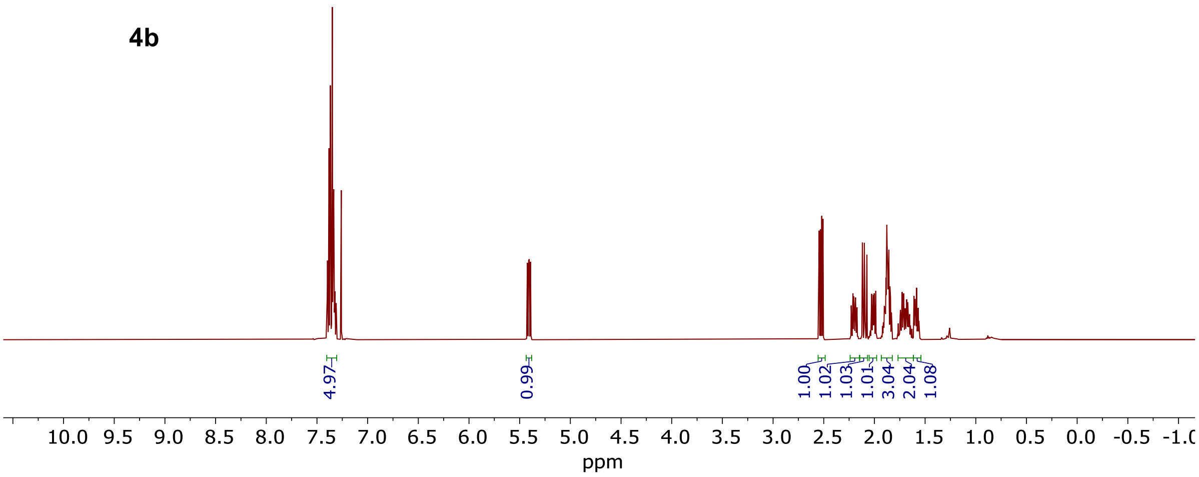


[13]**C NMR** (126MHz, $CDCl_3$) of **4b**

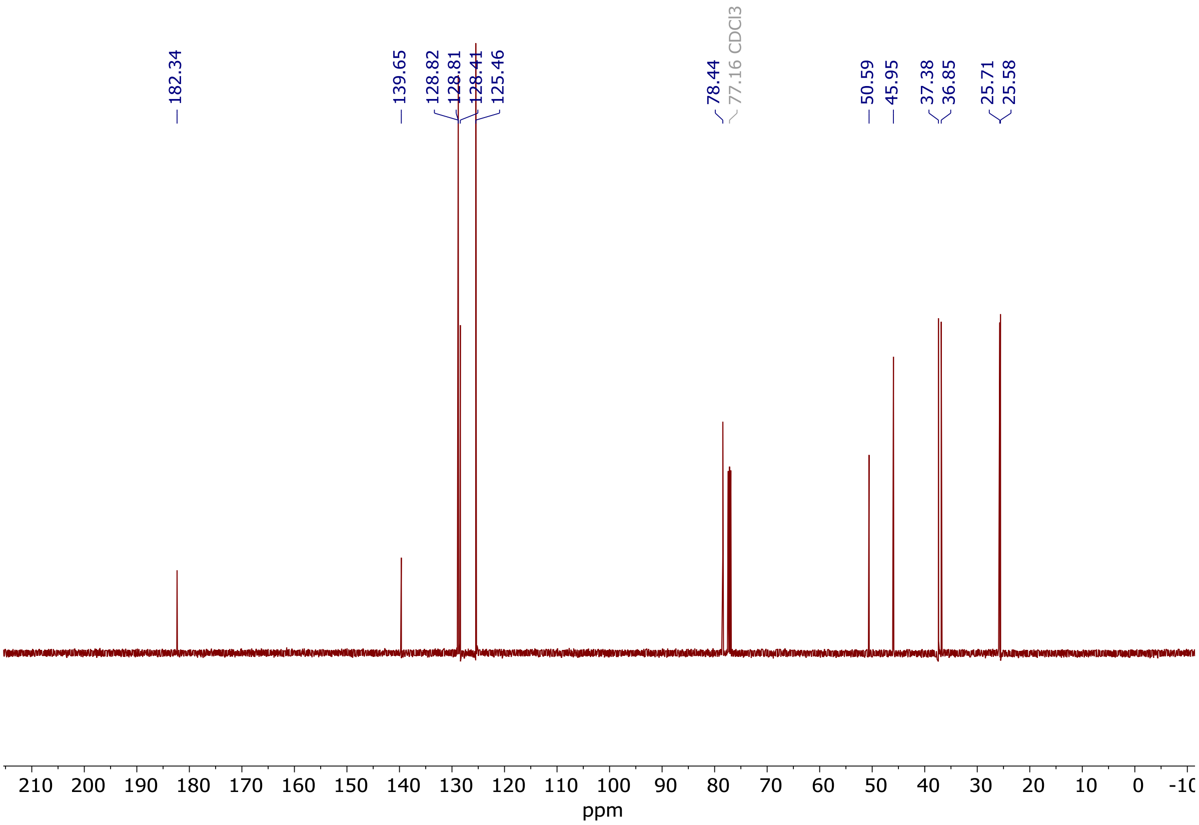

**$^{1}$H NMR** (599 MHz, $CDCl_3$) of **4c** see procedure

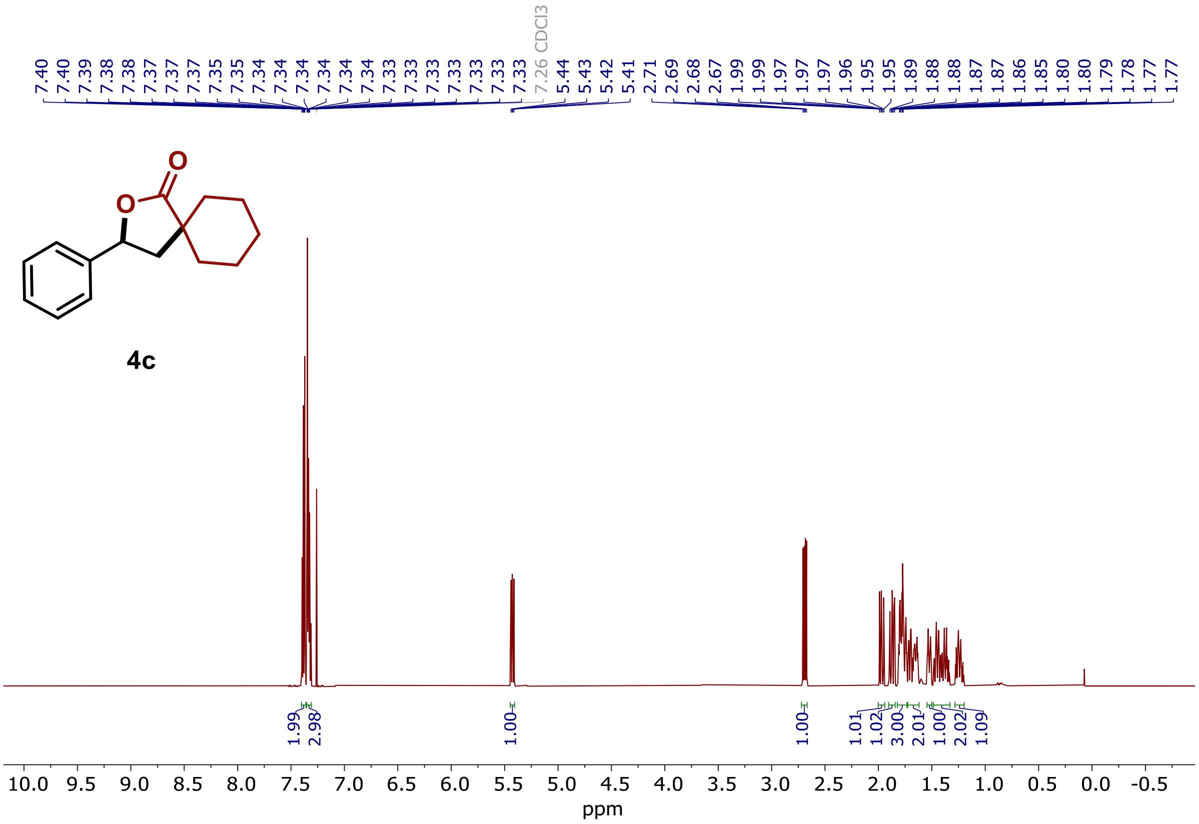


**$^{13}$C NMR** (151MHz, $CDCl_3$) of **4c**

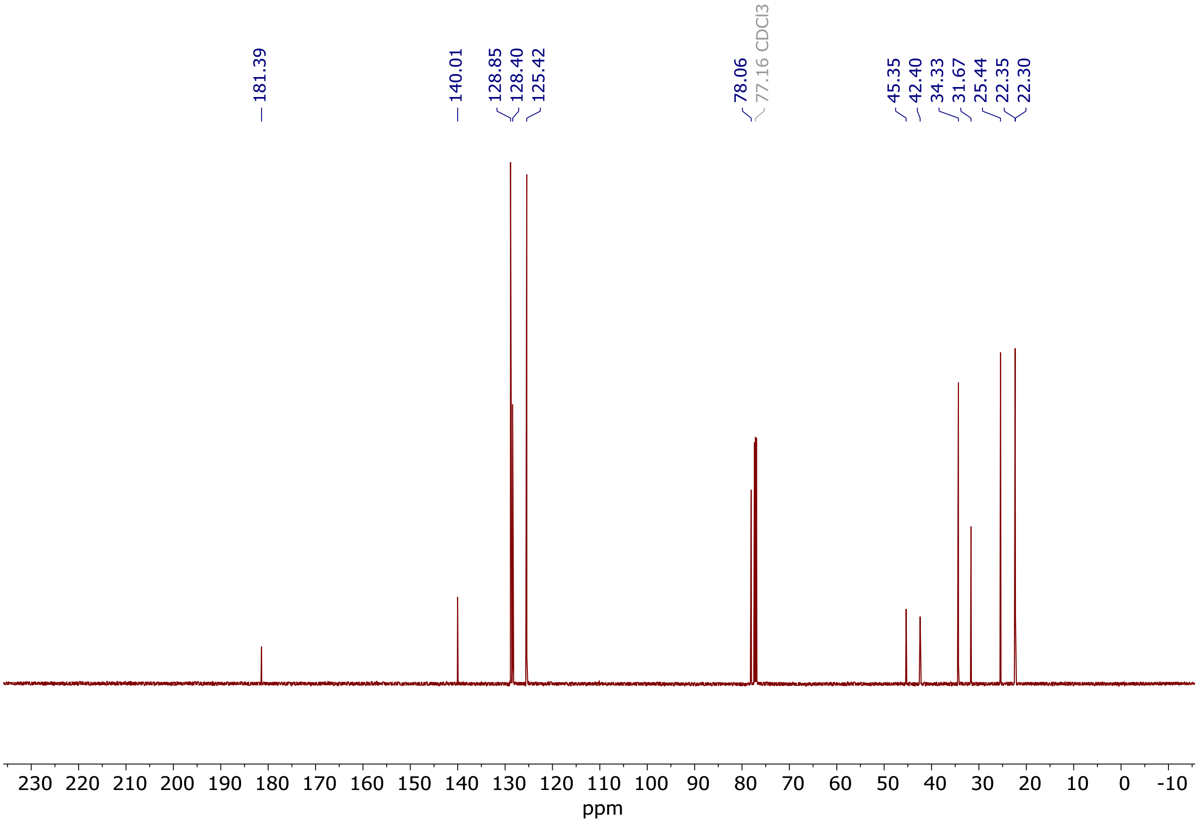

**$^{1}$H NMR** (500 MHz, CDCl₃) of **4d** see procedure

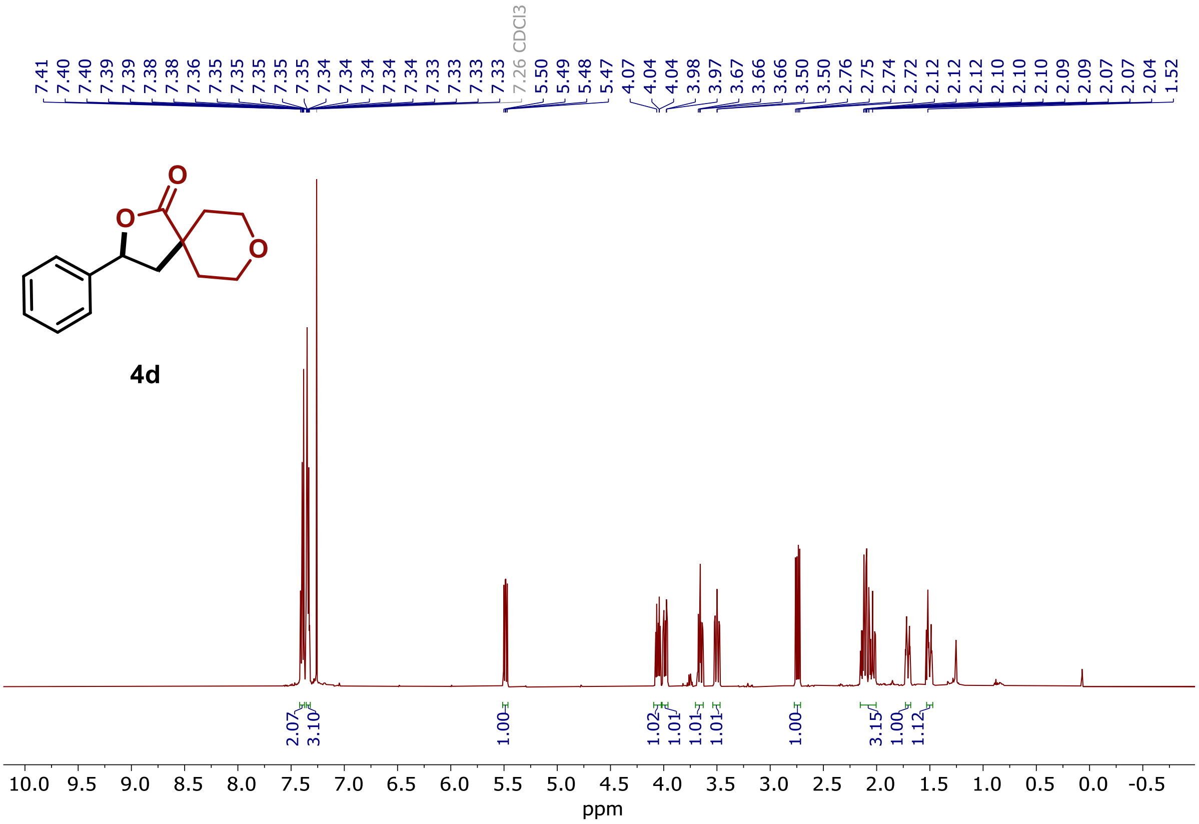

**4d**


**$^{13}$C NMR** (126MHz, CDCl₃) of **4d**

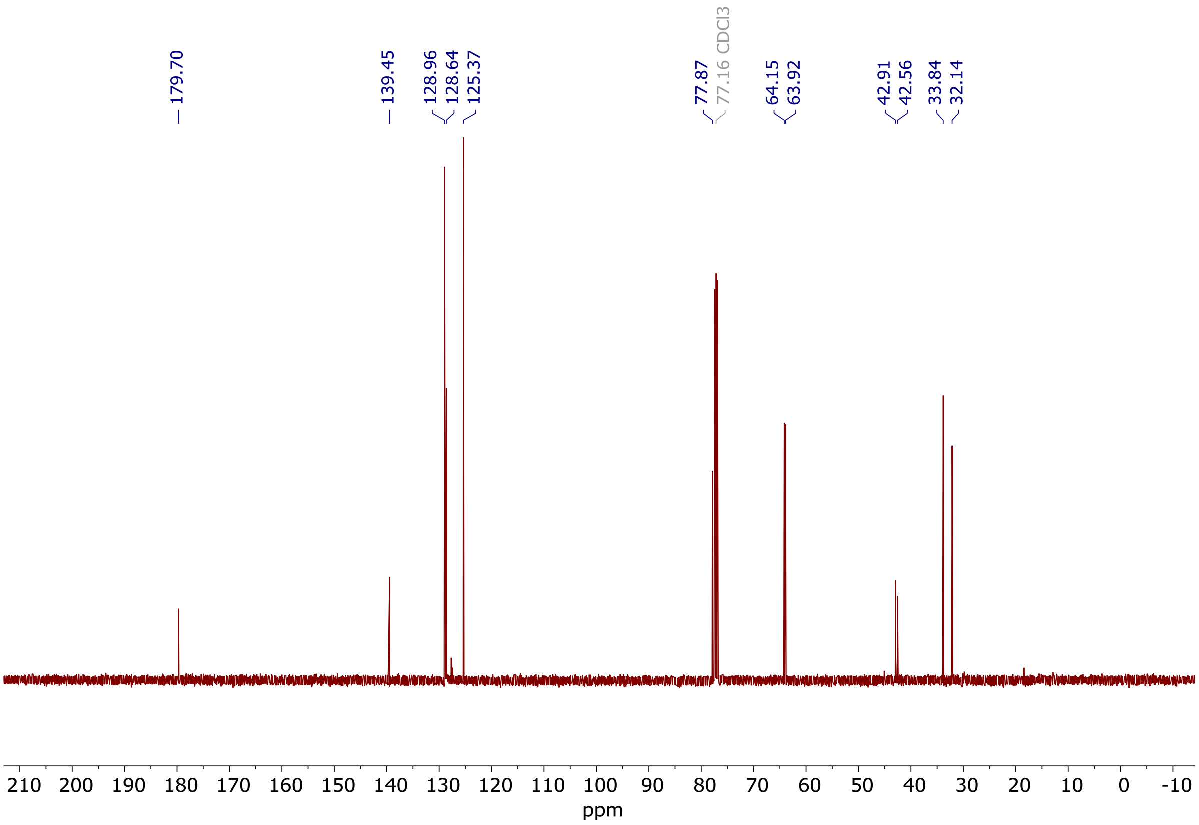

**$^{1}$H NMR** (599 MHz, $CDCl_3$) of **4e** see procedure

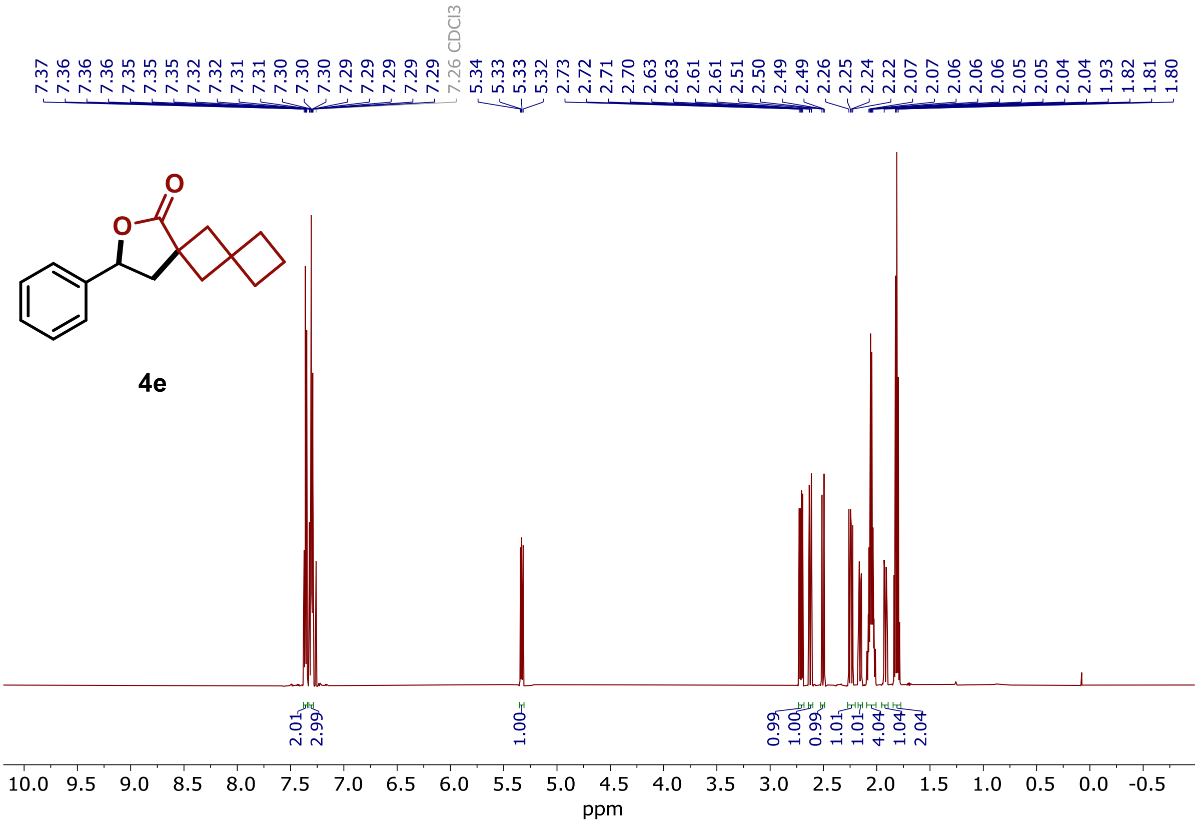


**$^{13}$C NMR** (151MHz, $CDCl_3$) of **4e**

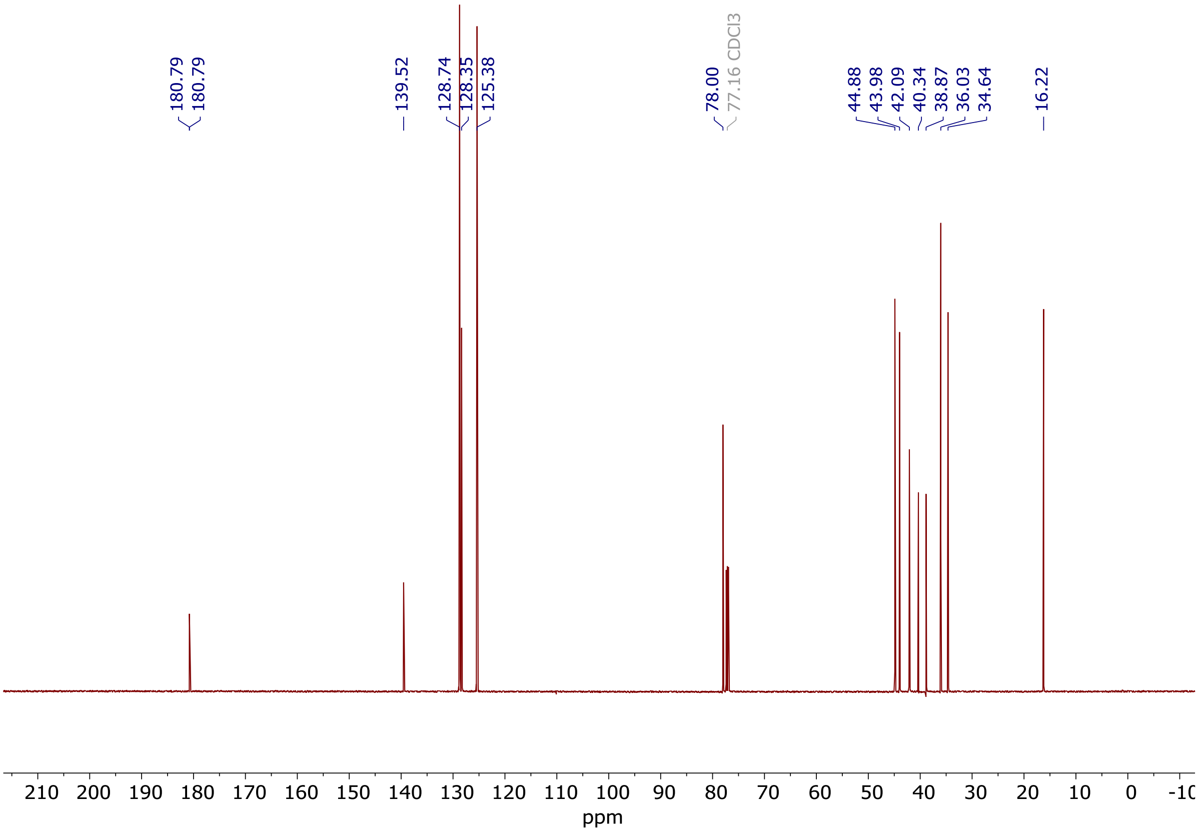

**¹H NMR** (599 MHz, $CDCl_3$) of **4f** see procedure

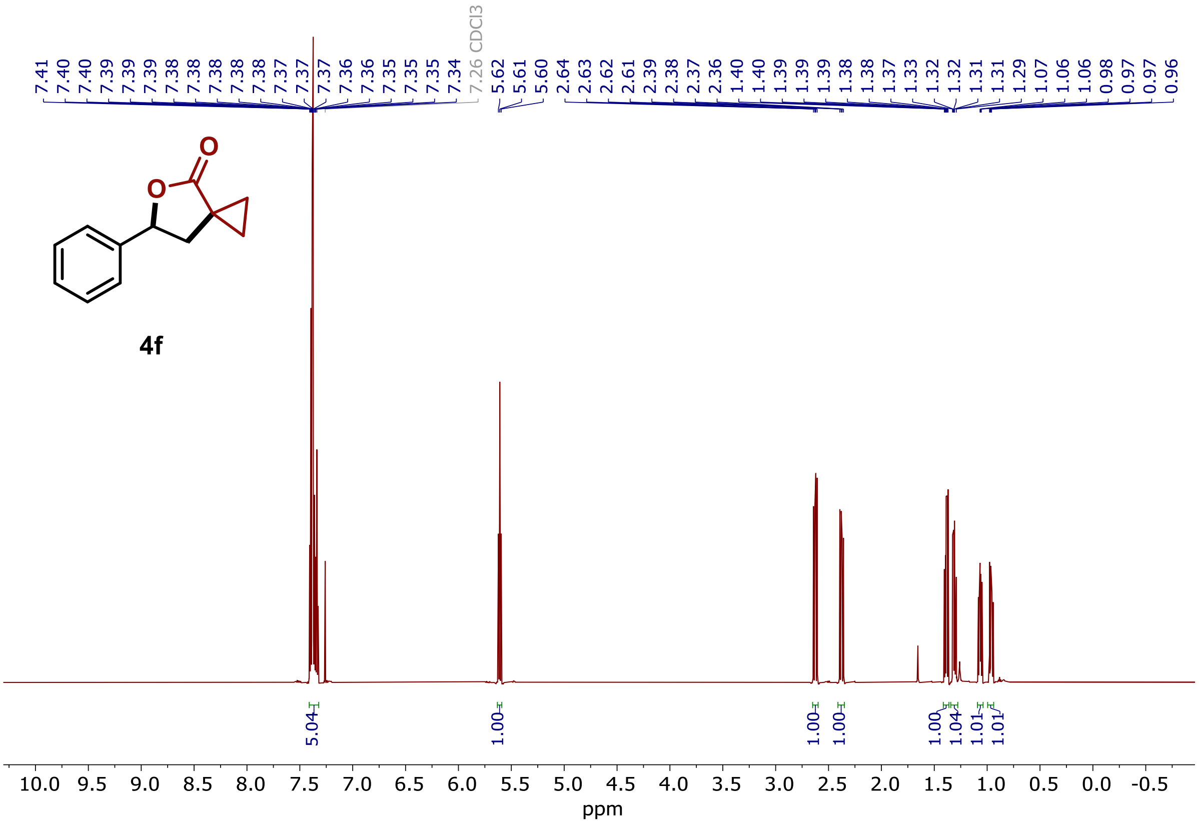


**¹³C NMR** (151MHz, $CDCl_3$) of **4f**

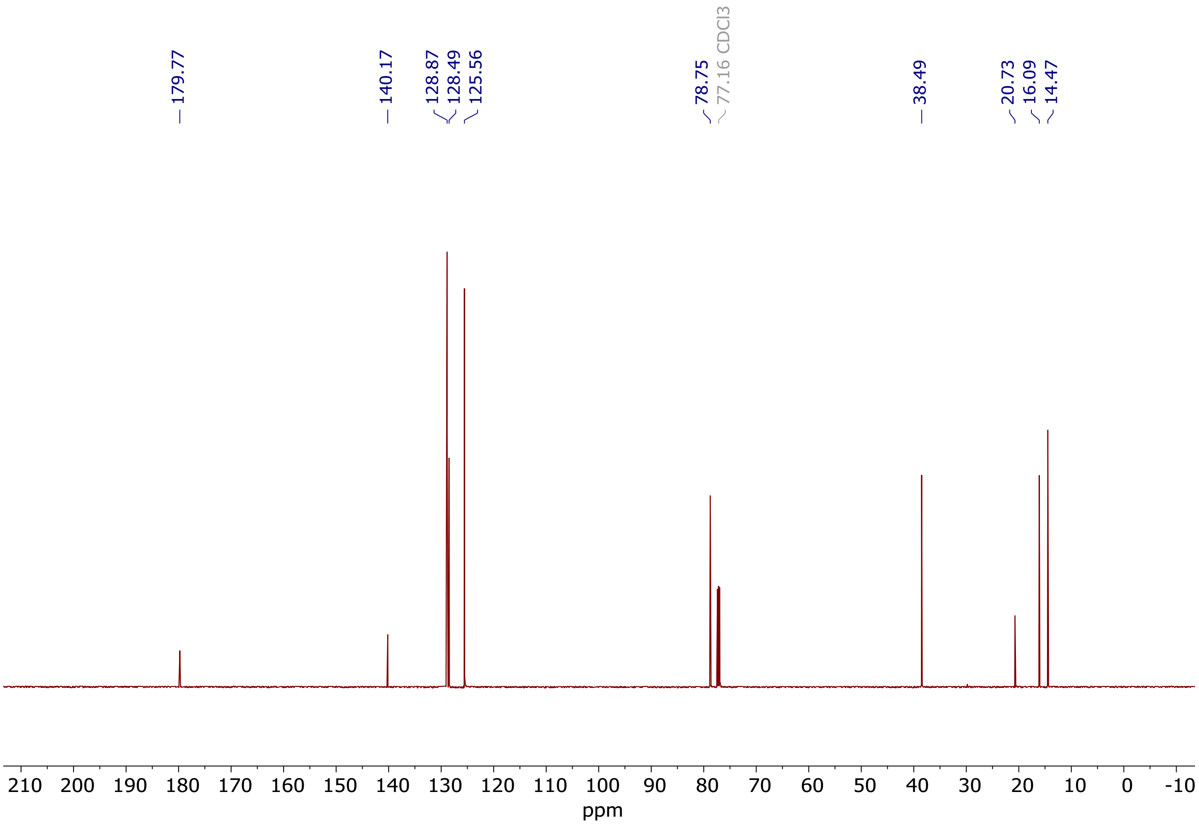

**$^{1}$H NMR** (599 MHz, $CDCl_3$) of **4g** see procedure

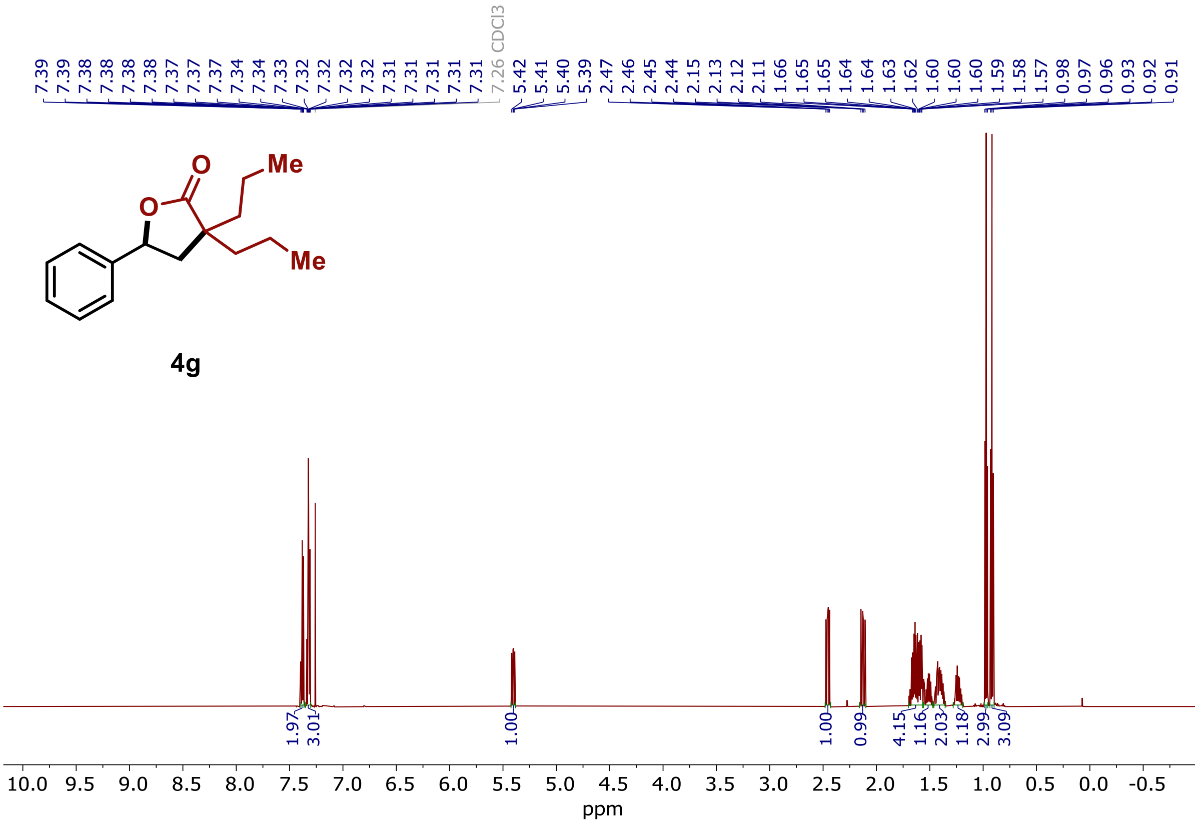


**$^{13}$C NMR** (151MHz, $CDCl_3$) of **4g**

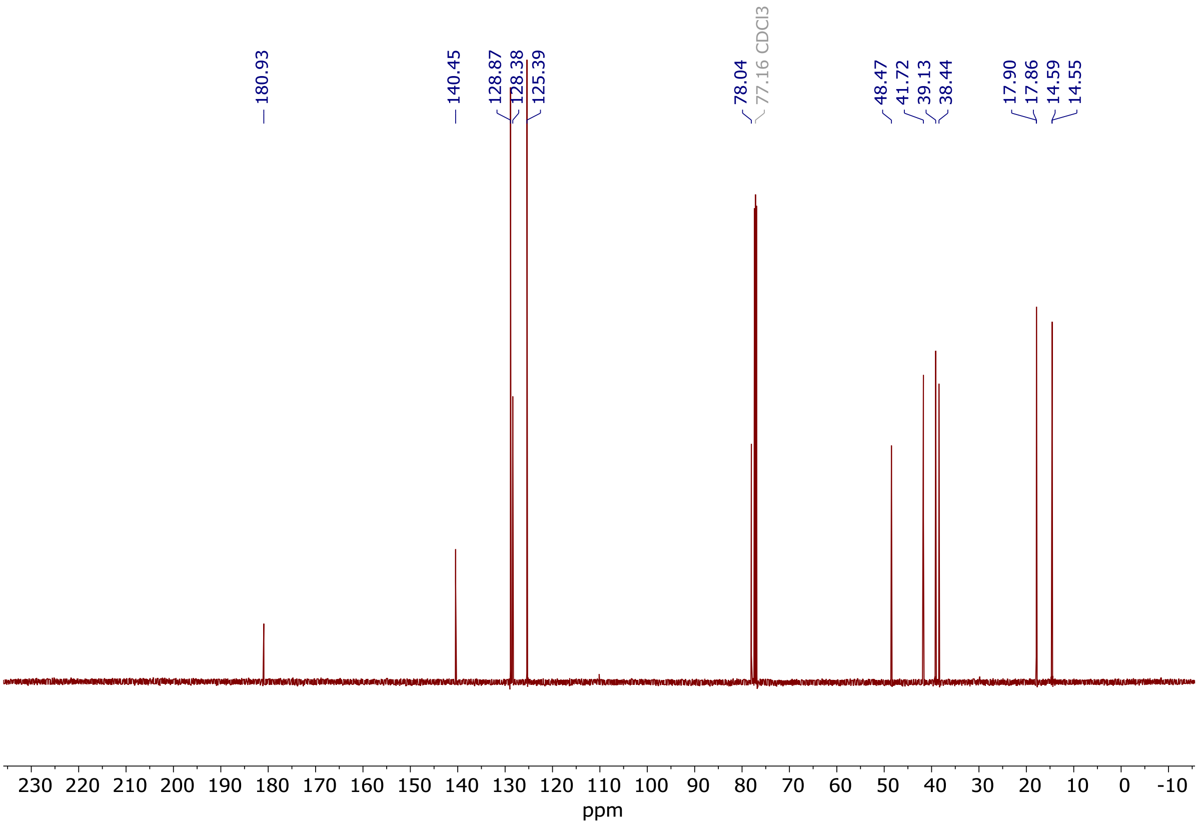

**$^1$H NMR** (599 MHz, CDCl₃) of the *trans* diastereomer of **4h** see procedure

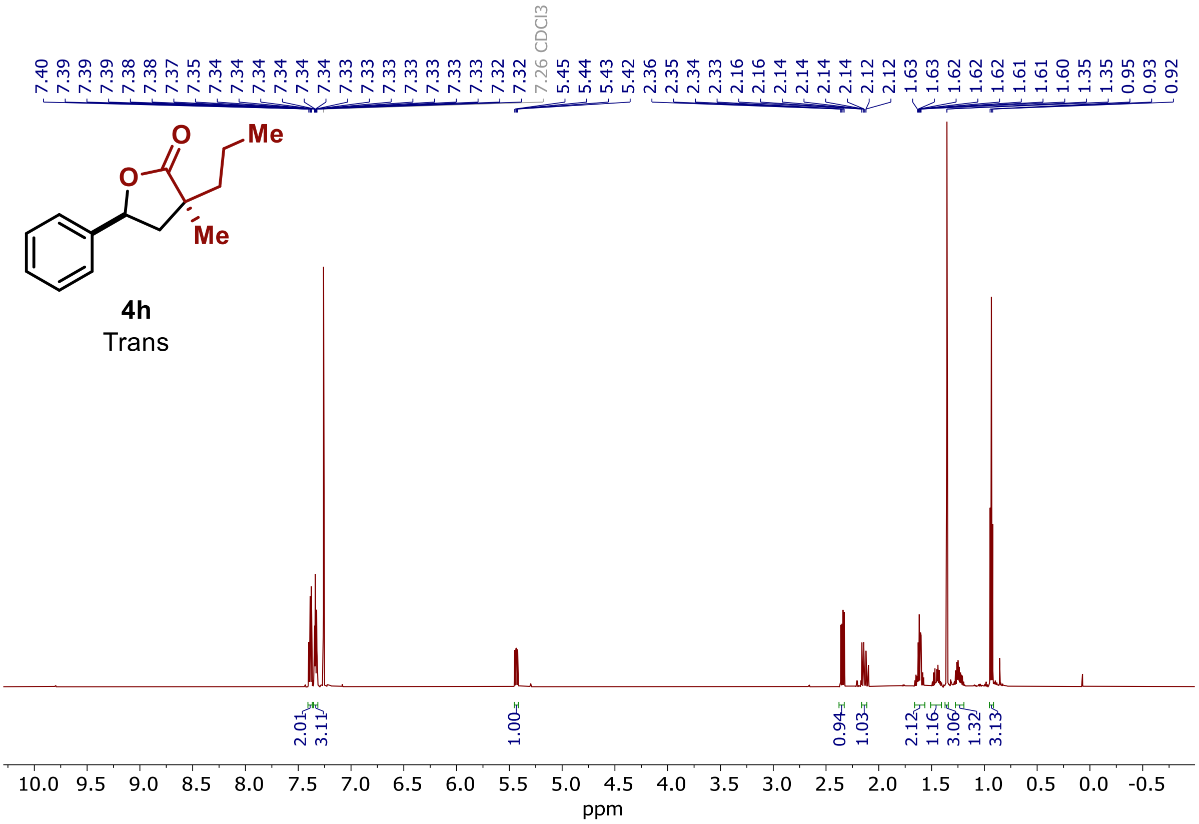


**$^{13}$C NMR** (151MHz, CDCl₃) of **4h**

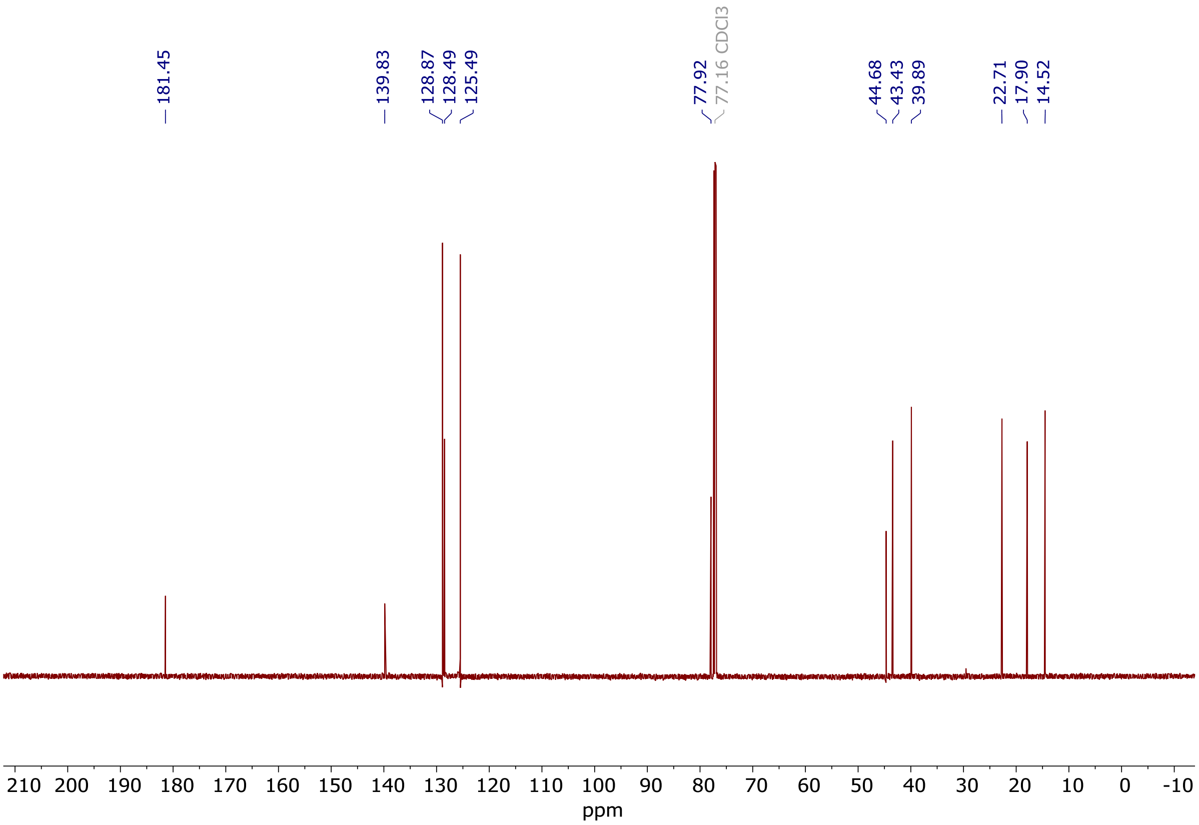

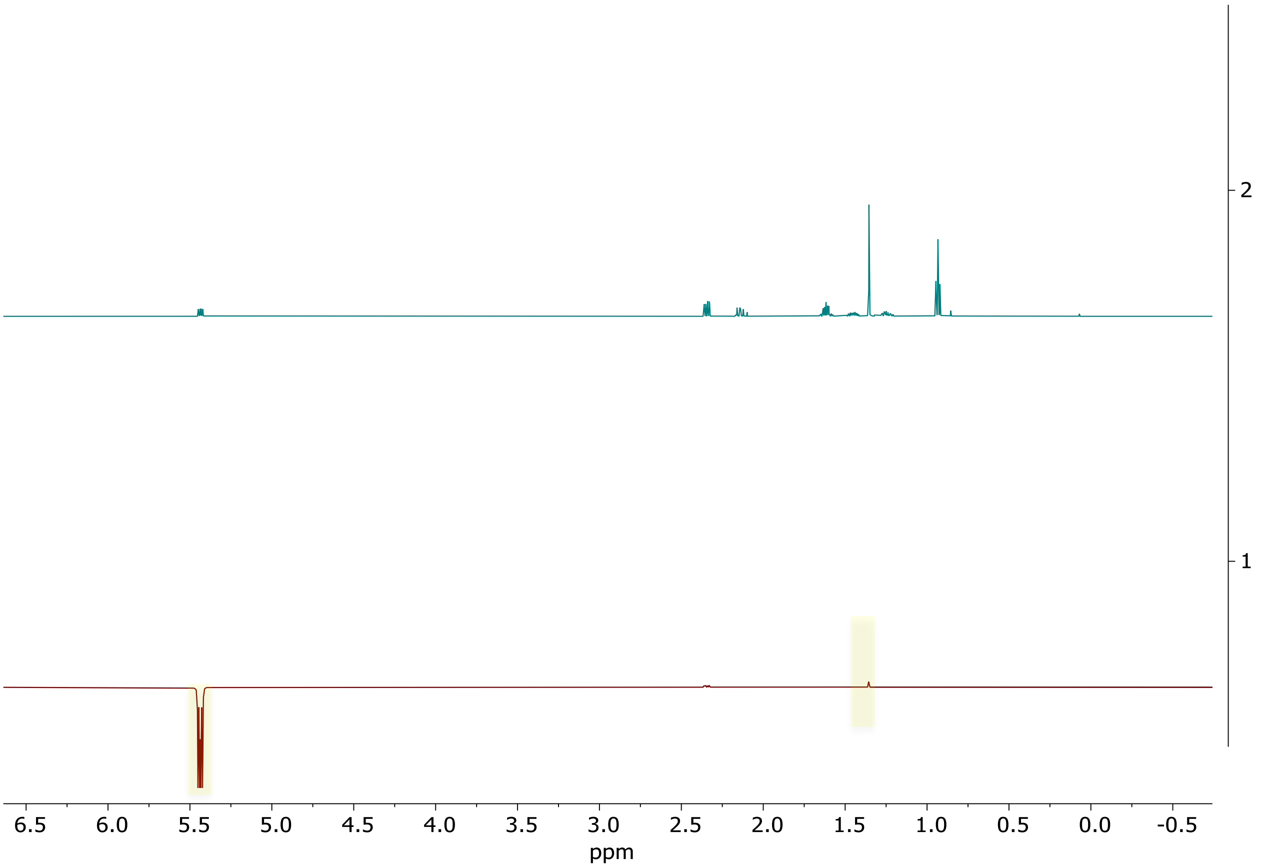


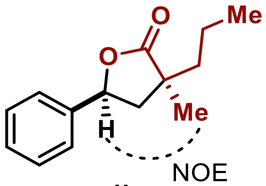


**4h**
Trans

**$^{1}$H NMR** (599 MHz, $CDCl_3$) of the *cis* diastereomer 2 of **4h** see procedure

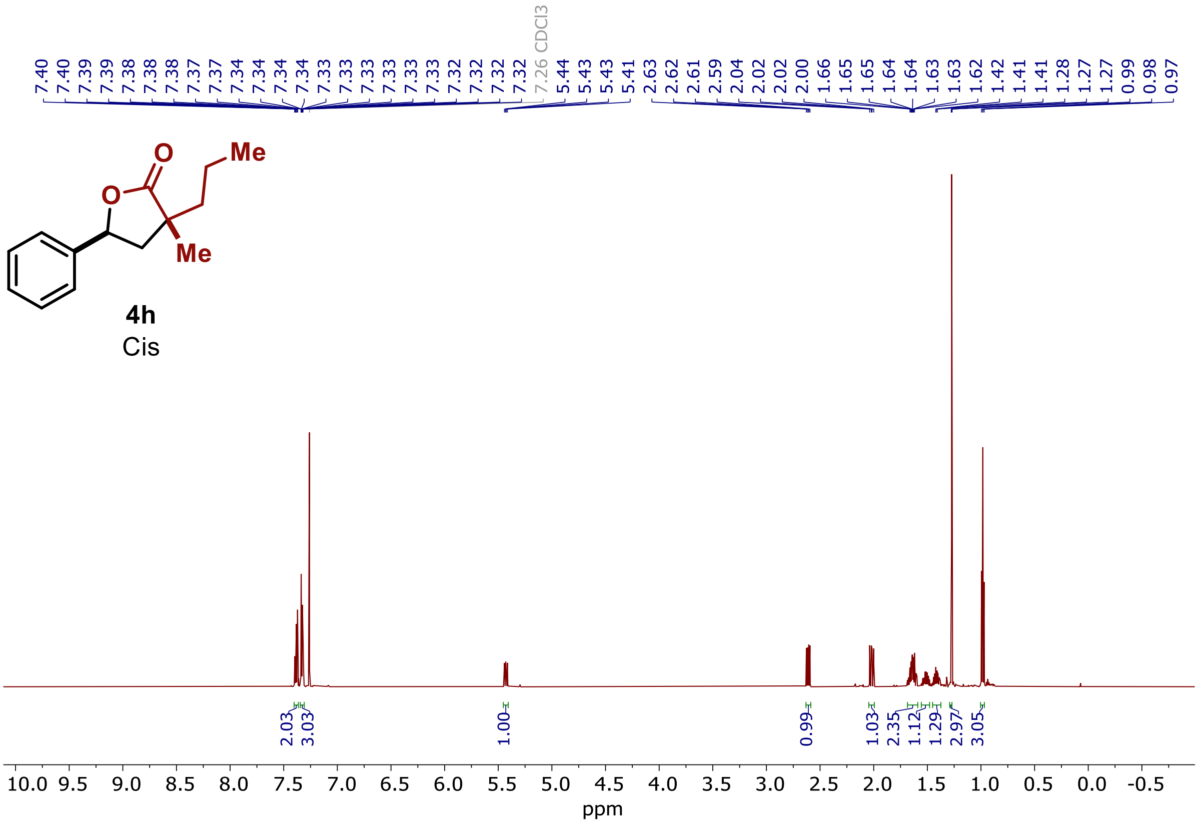


**$^{13}$C NMR** (151MHz, $CDCl_3$) of **4h**

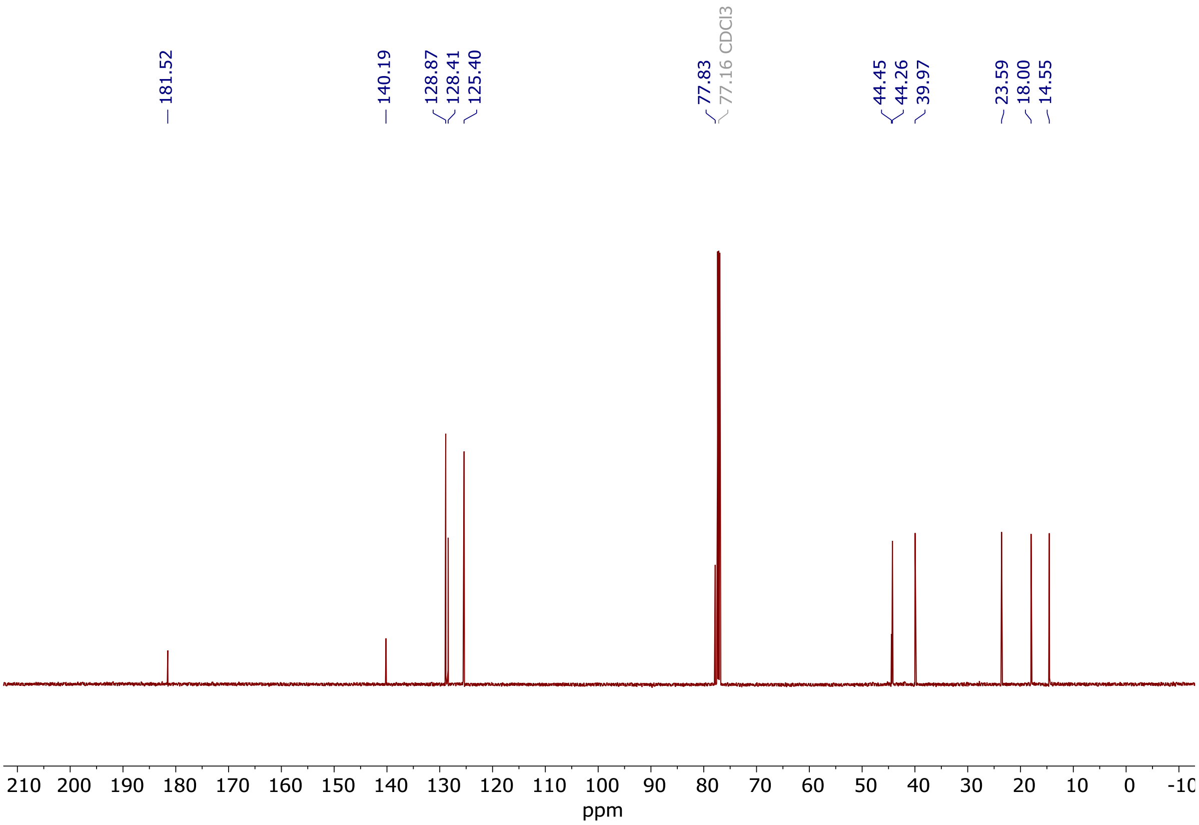

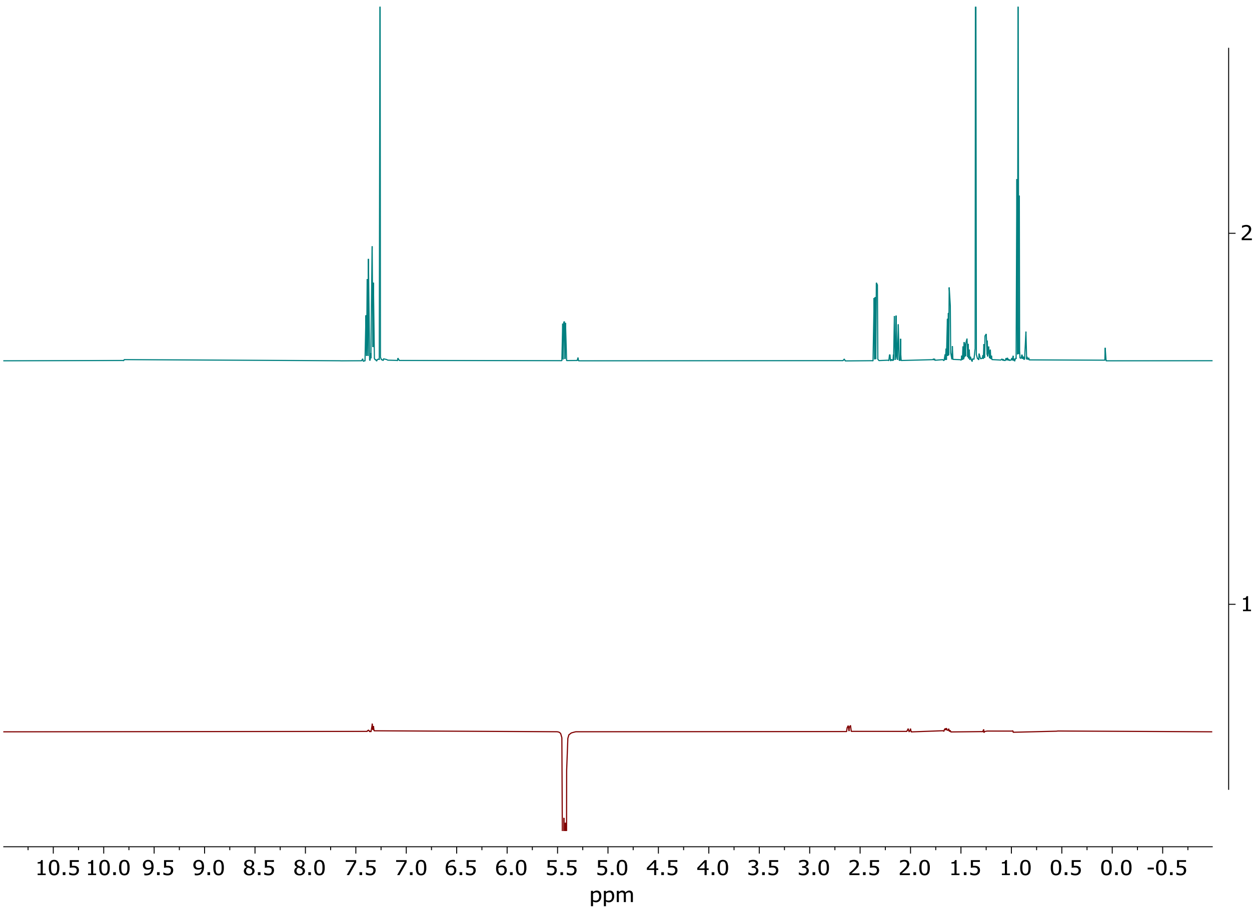
2
1
10.5 10.0 9.5 9.0 8.5 8.0 7.5 7.0 6.5 6.0 5.5 5.0 4.5 4.0 3.5 3.0 2.5 2.0 1.5 1.0 0.5 0.0 -0.5
ppm

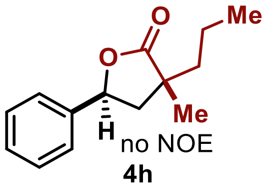
O
Me
O
Me
H
no NOE

**4h**
Cis

**¹H NMR** (599 MHz, $CDCl_3$) of **4i** see procedure

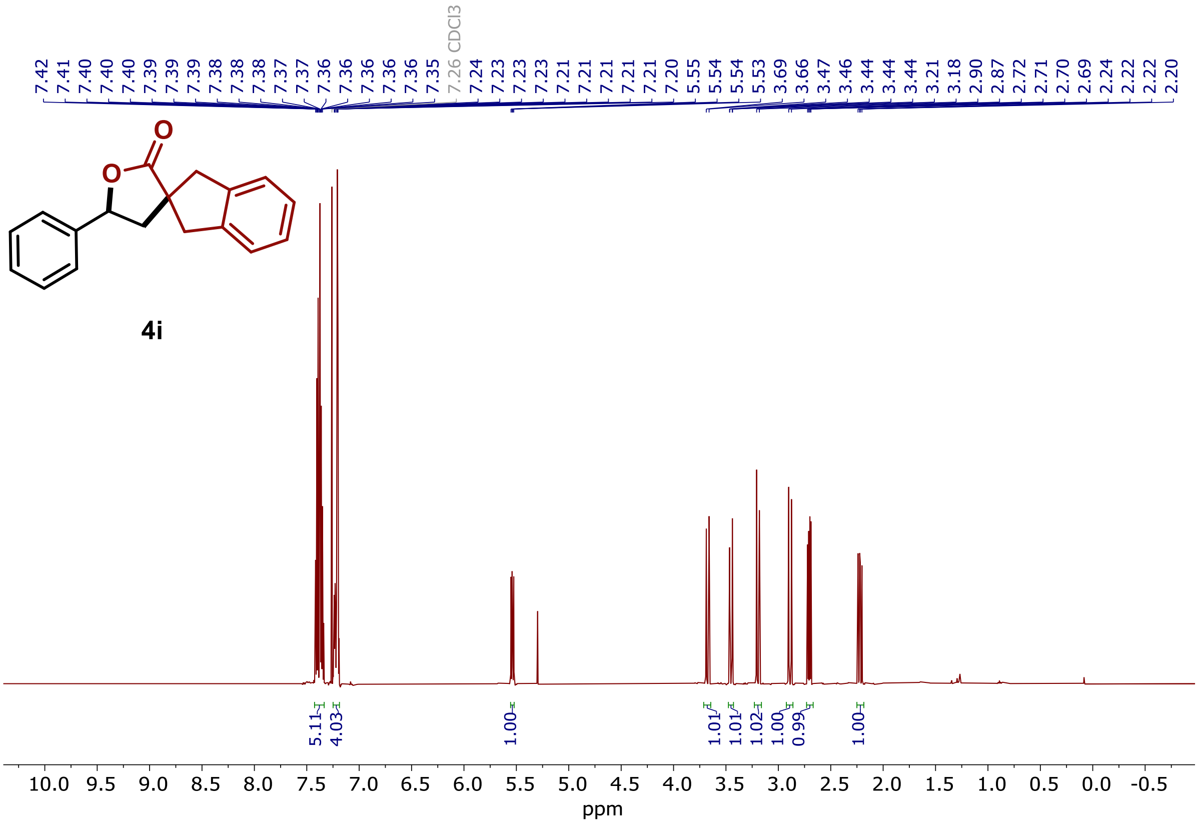


**¹³C NMR** (151MHz, $CDCl_3$) of **4i**

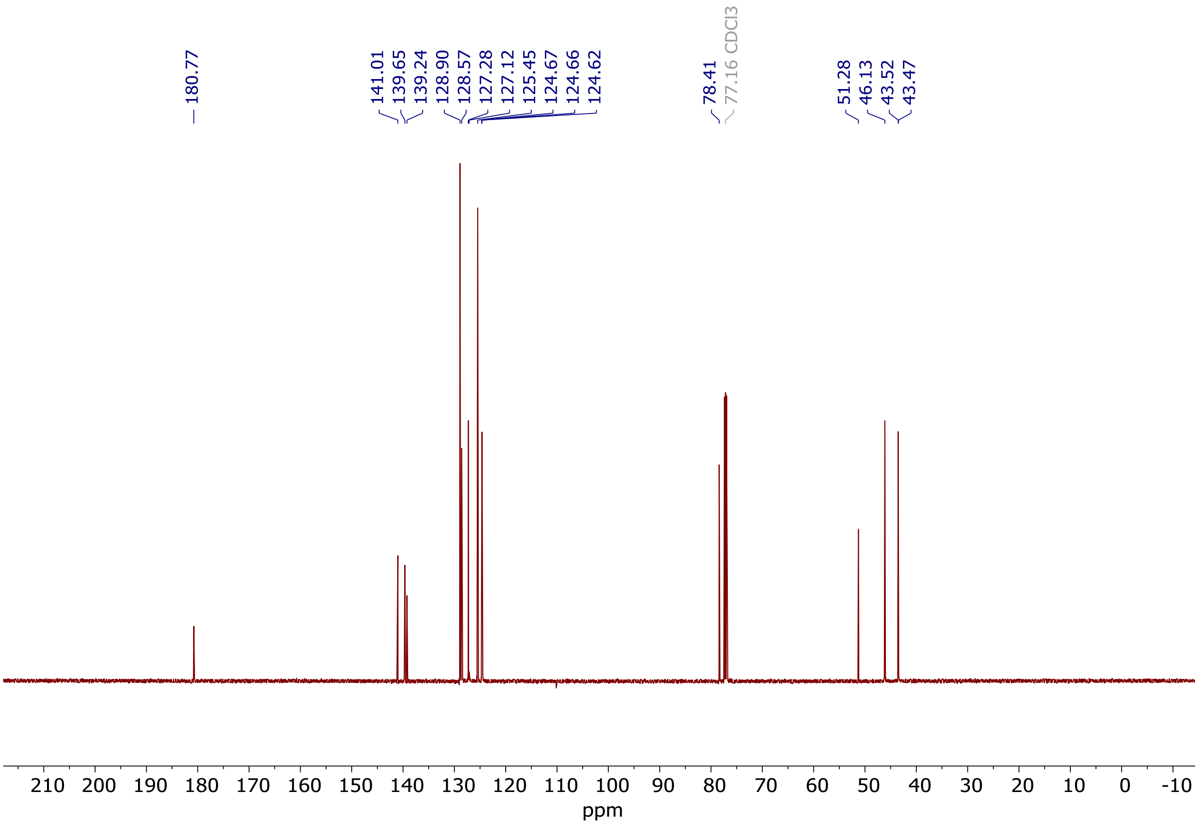

**$^{1}$H NMR** (500MHz, $CDCl_3$) of **PTH** see procedure

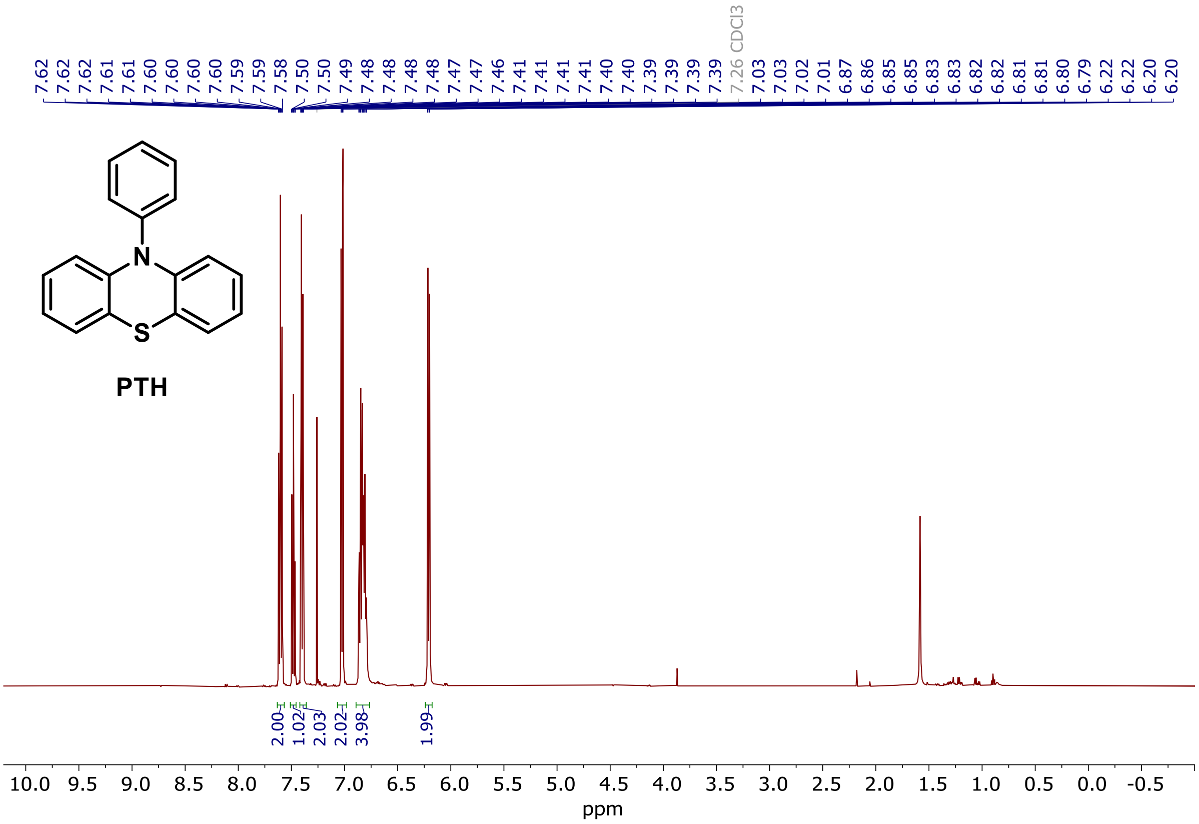


**$^{13}$C NMR** (126 MHz, $CDCl_3$) of **PTH**

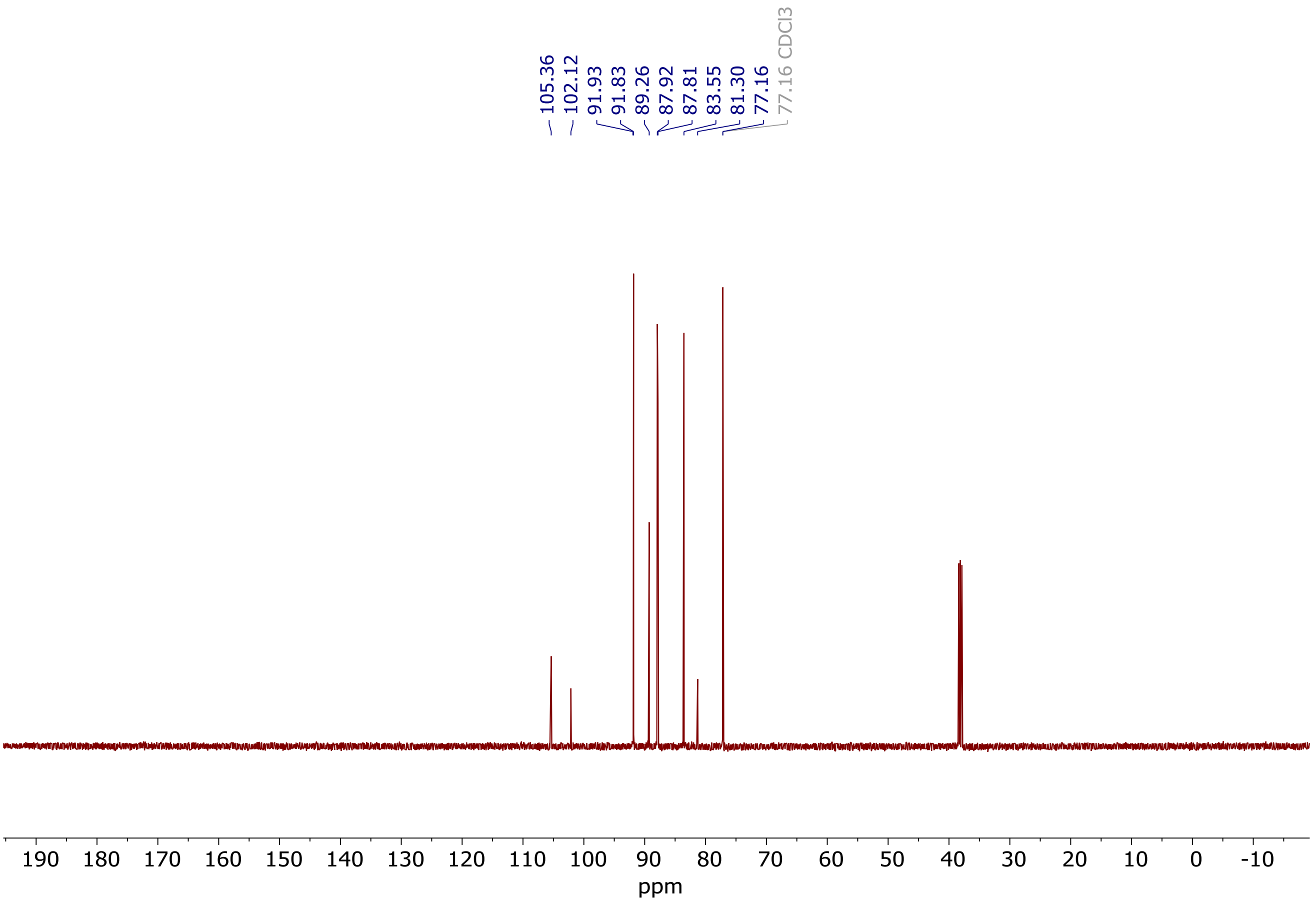

**$^{1}$H NMR** (599 MHz, $CD_3CN$) of **PTH-O** see procedure

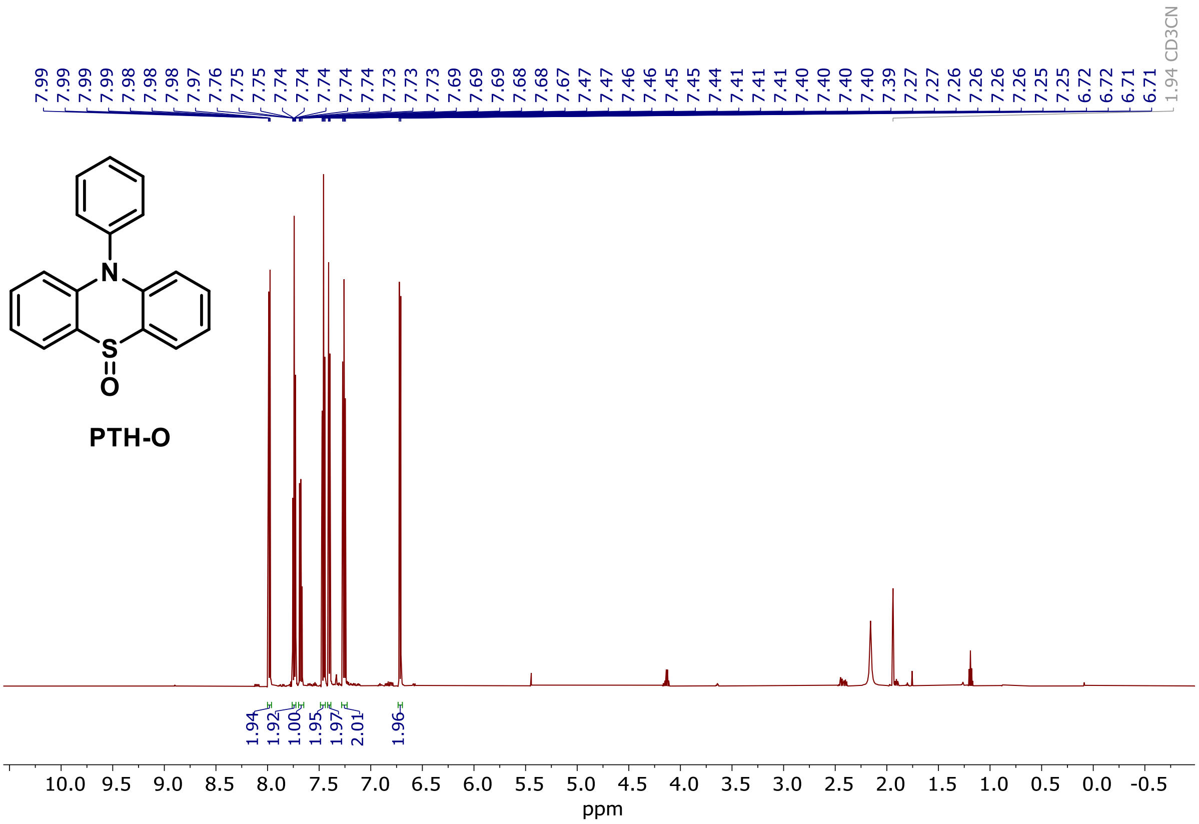


**$^{13}$C NMR** (151MHz, $CDl_3CN$) of **PTH-O**

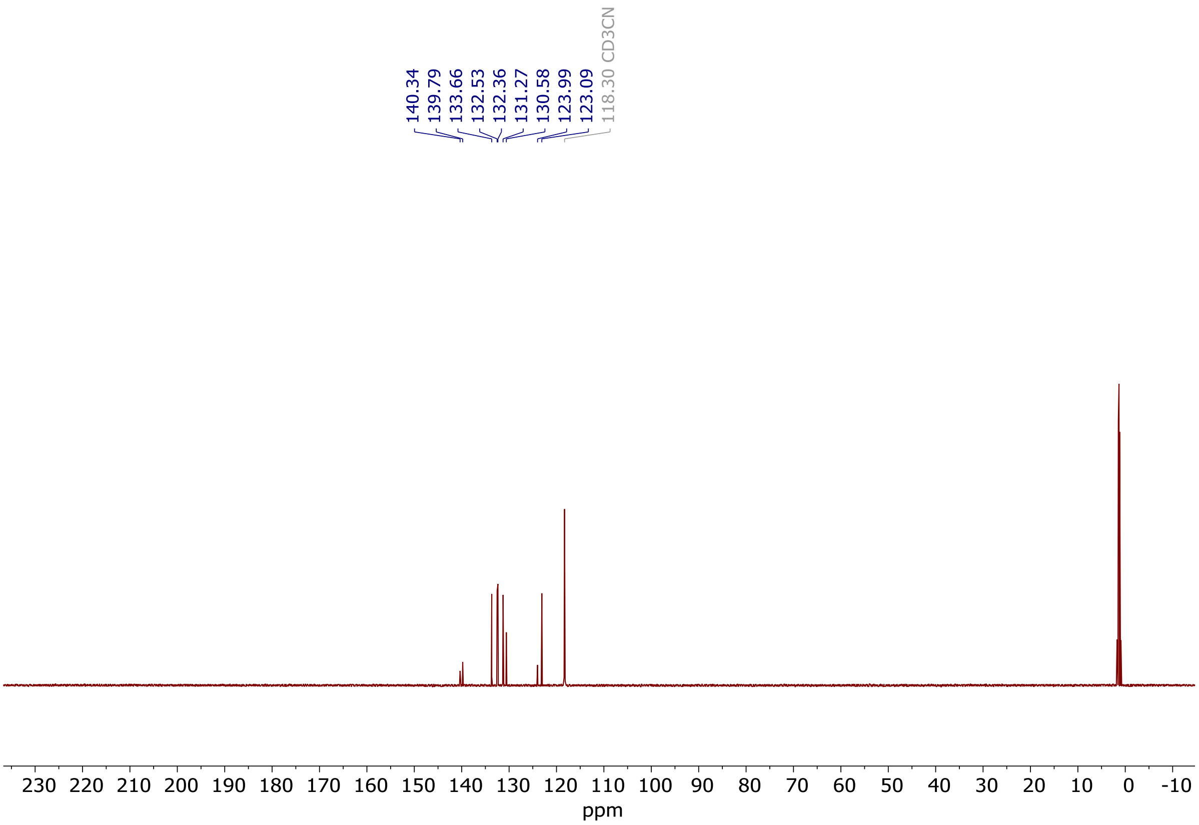

**$^{1}$H NMR** (599 MHz, $CDCl_3$) of **PTH-$O_2$** see procedure

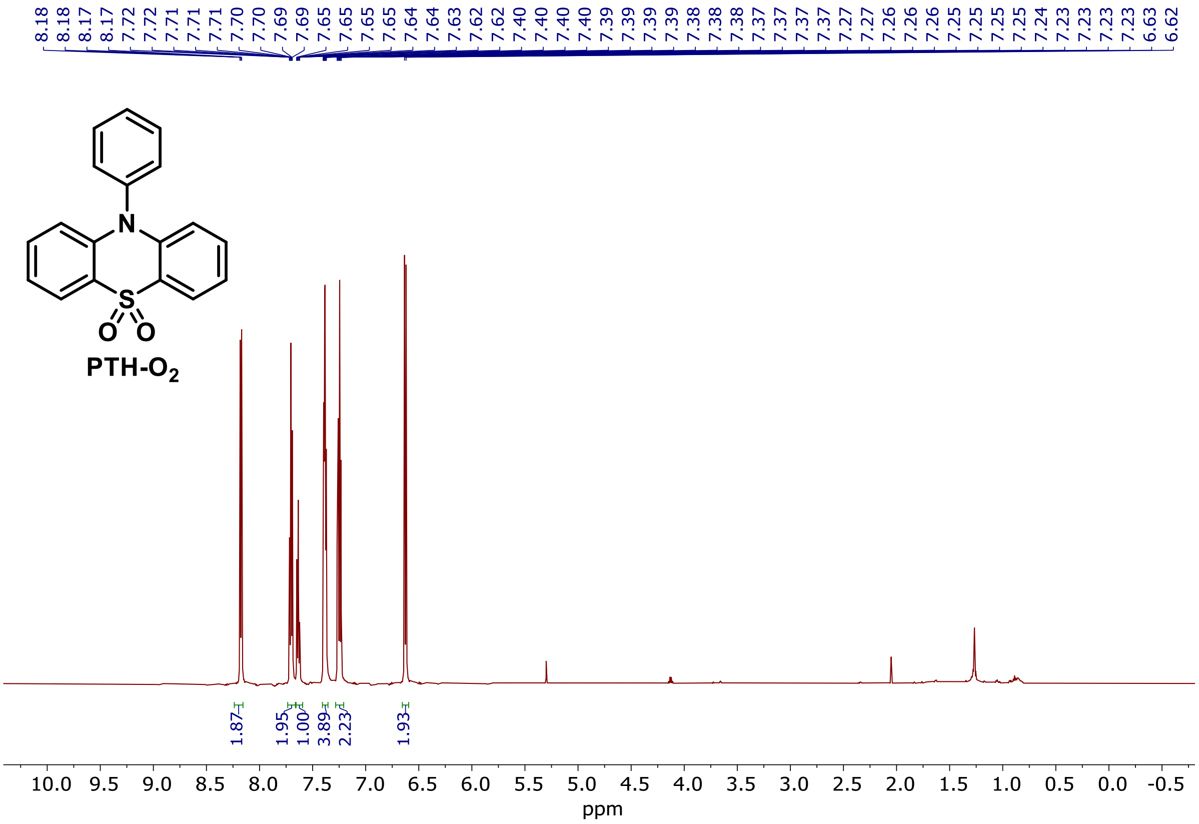


**$^{13}$C NMR** (151MHz, $CDCl_3$) of **PTH-$O_2$**

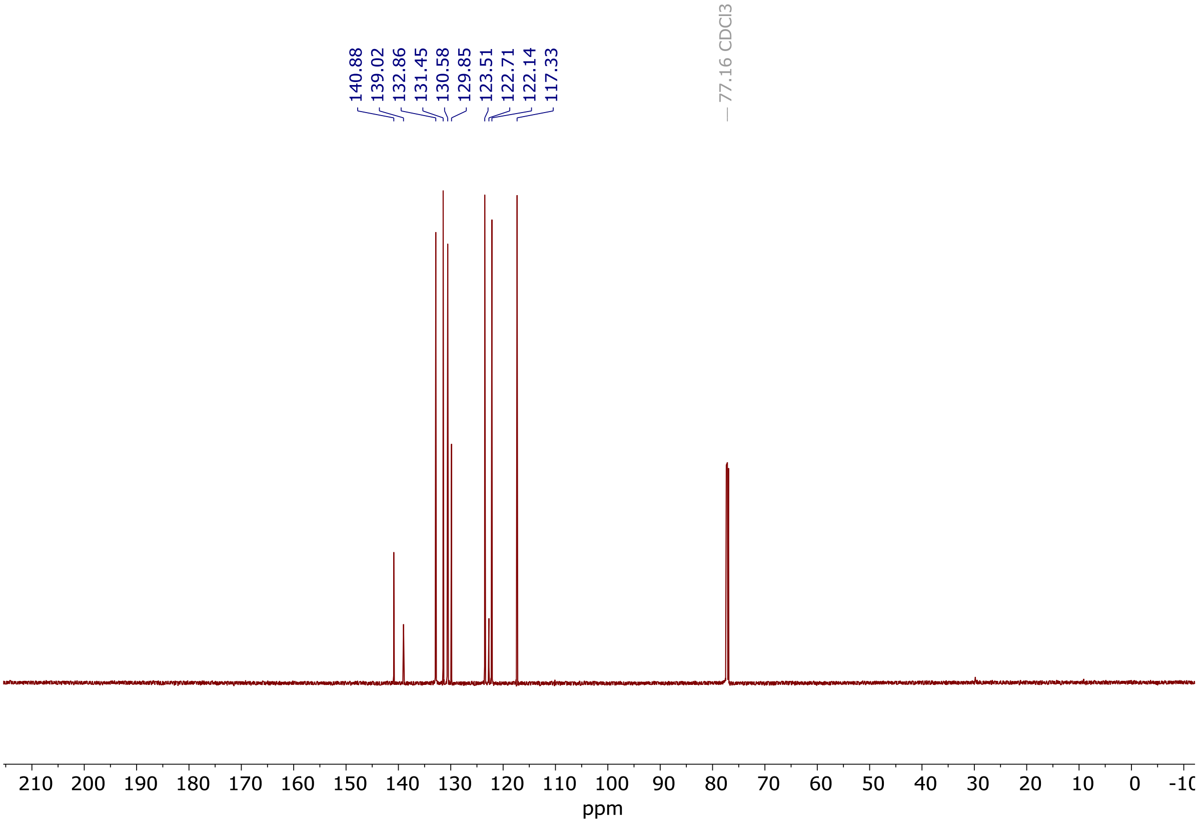

**$^{1}$H NMR** (400 MHz, $CD_3CN$) of **N-(4-OMe-Ph)-PTH** see procedure

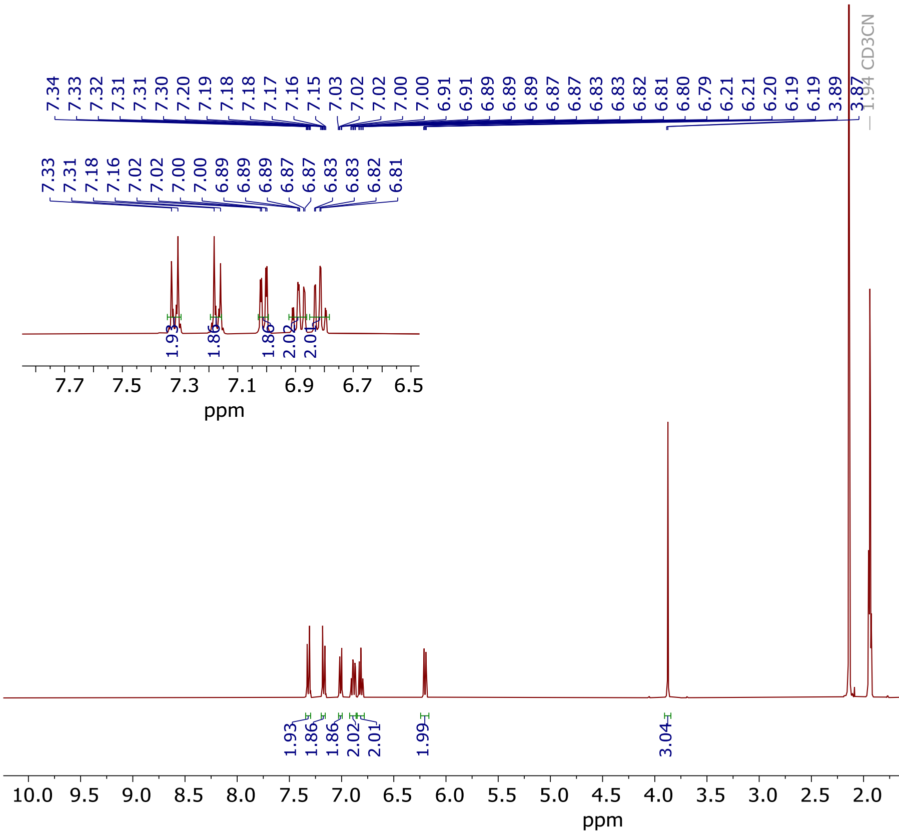


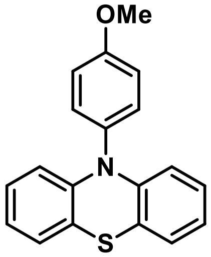


**$^{13}$C NMR** (101MHz, $CD_3CN$) of **N-(4-OMe-Ph)-PTH**

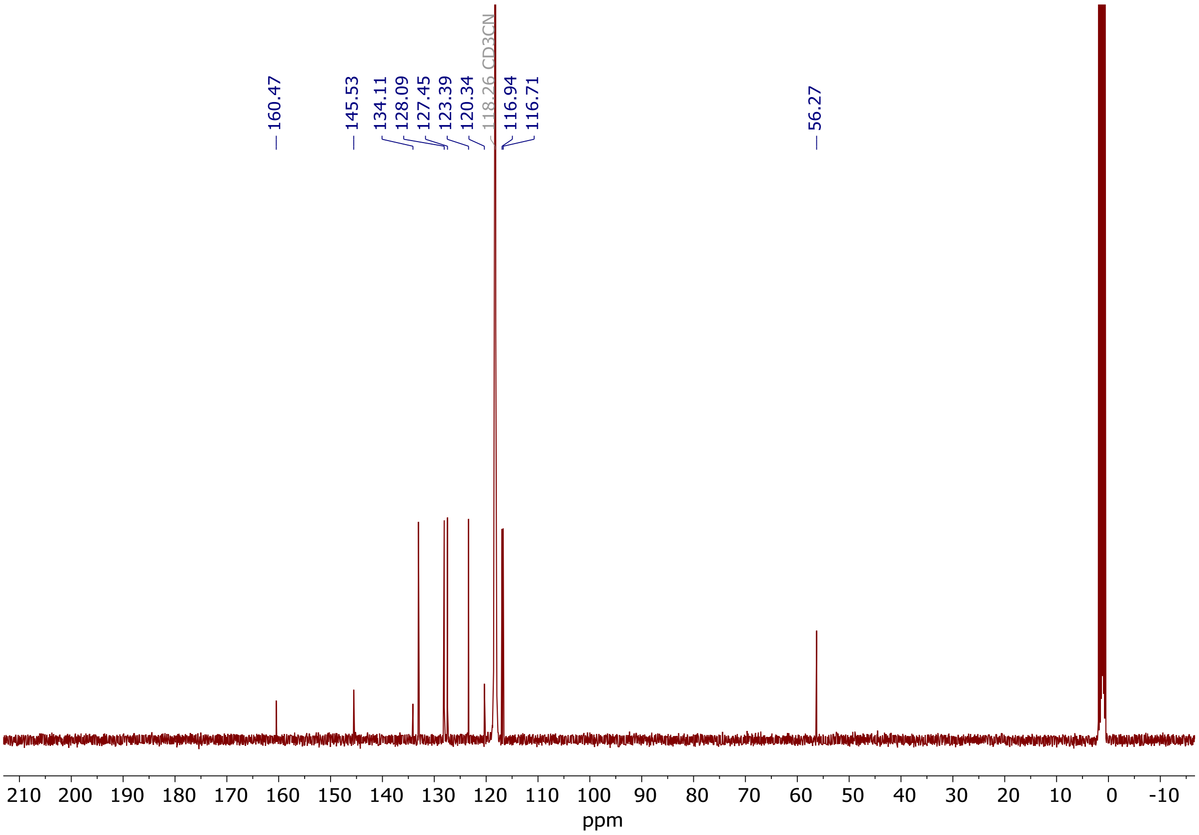

**$^{1}$H NMR** (400 MHz, $CD_3CN$) of **N-(4-$CF_3$-Ph)-PTH** see procedure

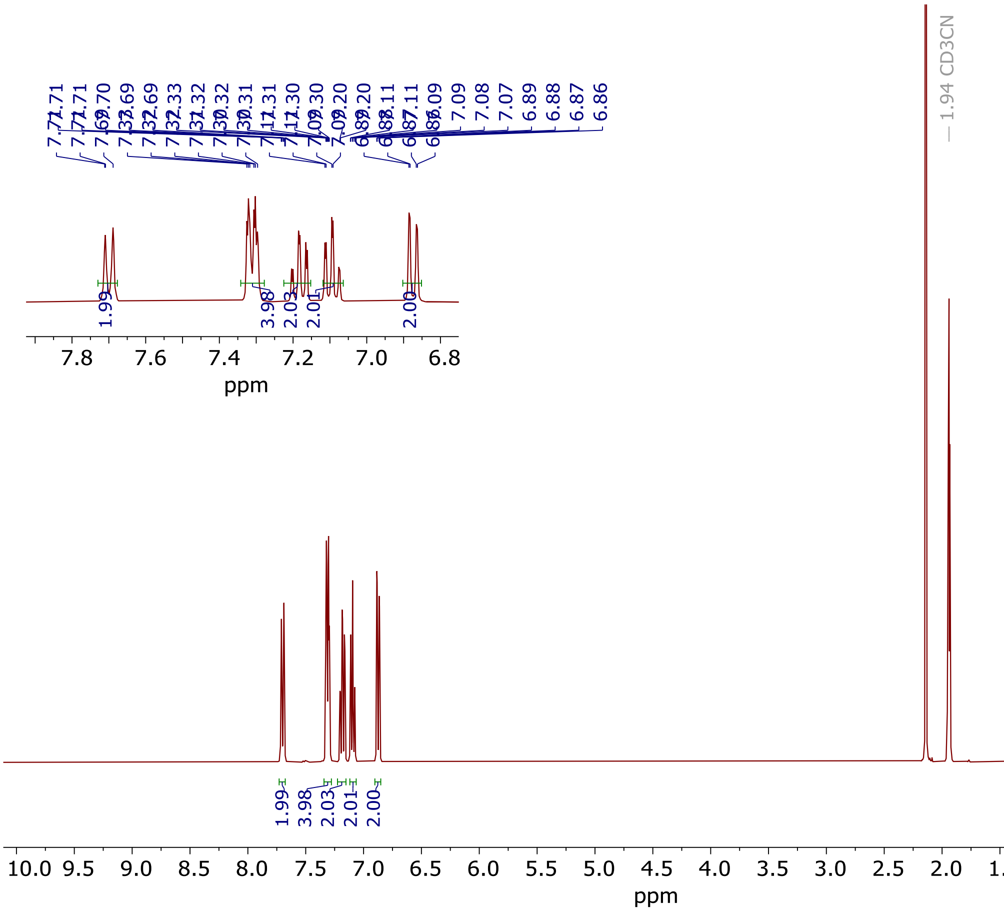


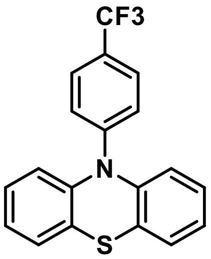


**N-(4-$CF_3$-Ph)-PTH**

**$^{13}$C{$^{19}$F} NMR** (126 MHz, $CD_3CN$) of **N-(4-$CF_3$-Ph)-PTH**

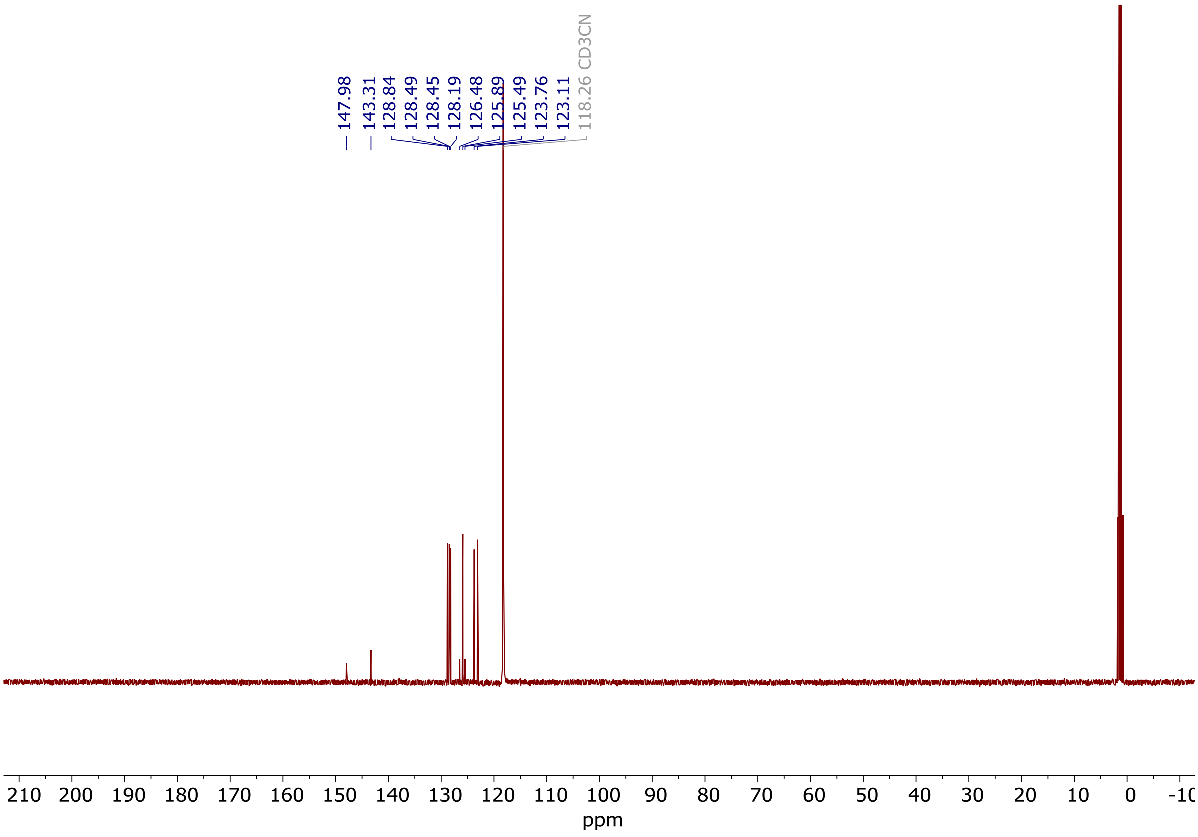

**$^{19}$F NMR** (376 MHz, $CD_3CN$) of **N-(4-$CF_3$-Ph)-PTH**

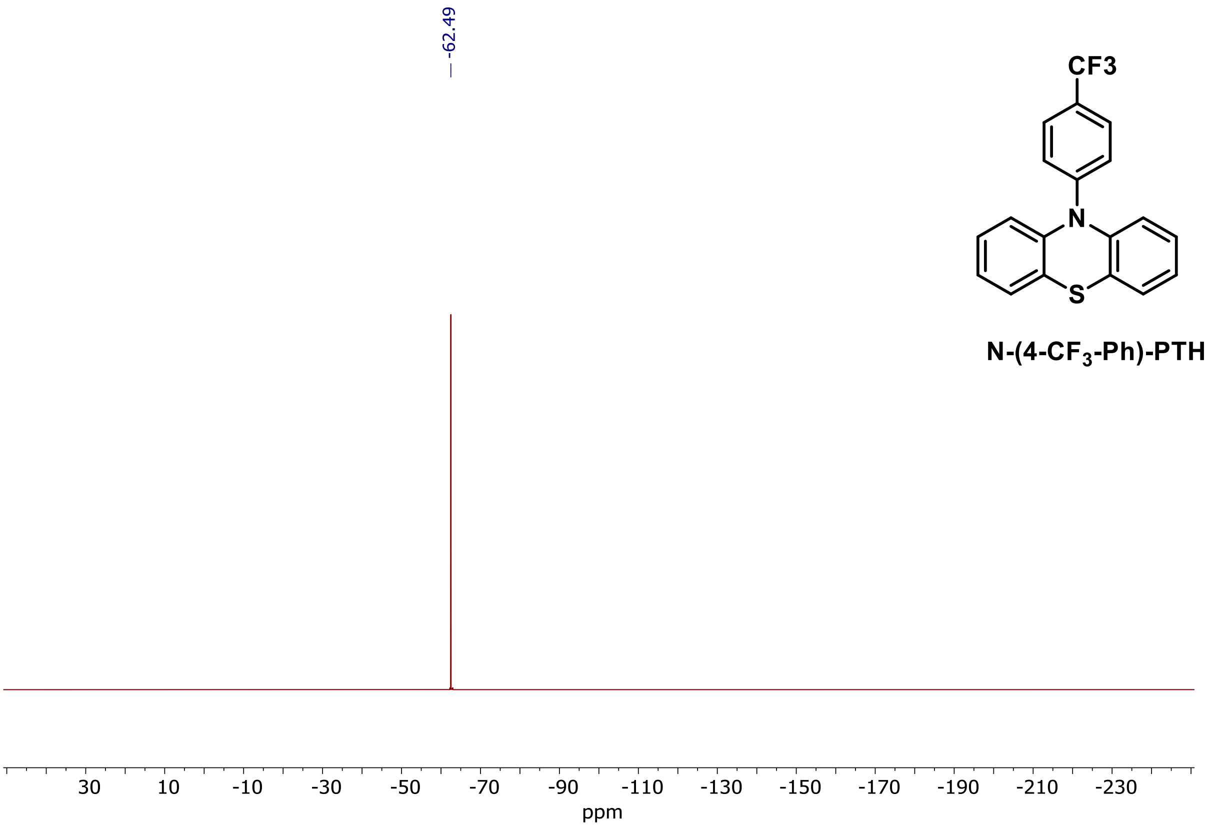


**N-(4-$CF_3$-Ph)-PTH**

$^{1}$H NMR (400 MHz, $CD_3CN$) of **2-MeO-PTH** see procedure

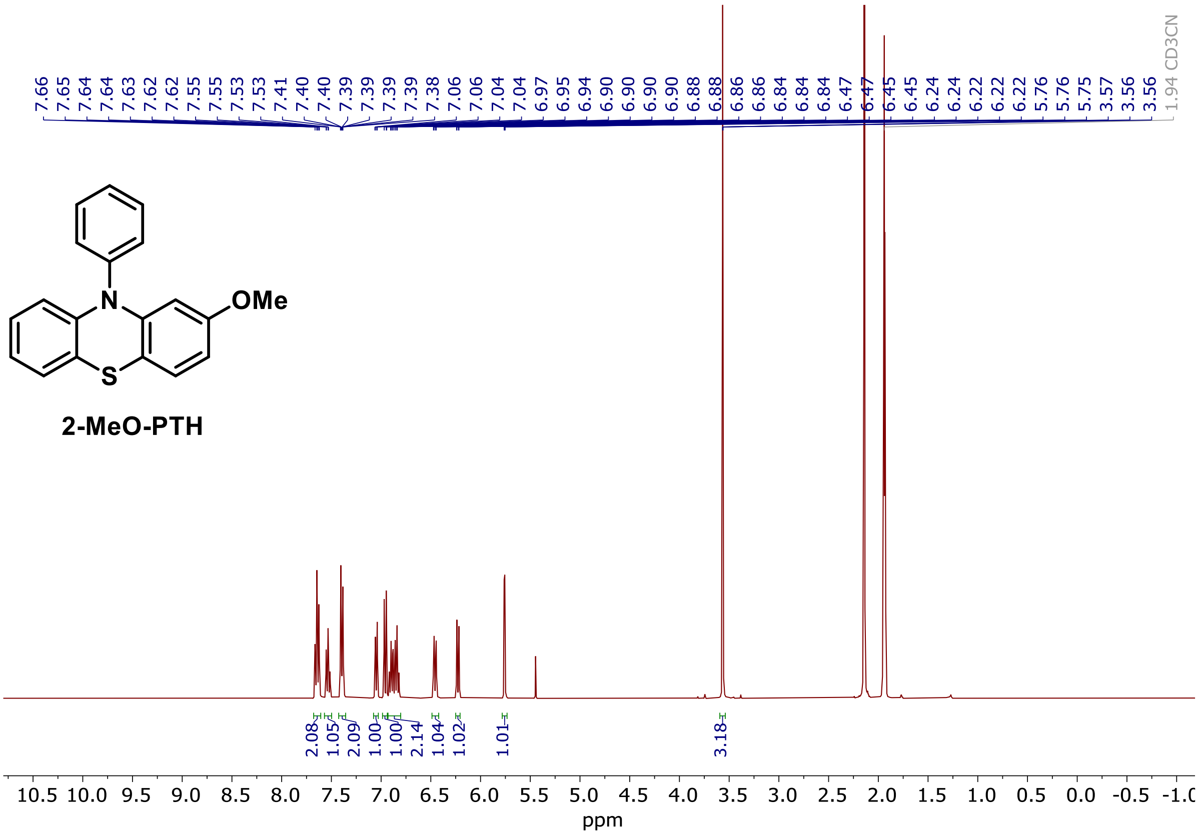


$^{13}$C NMR (101MHz, $CD_3CN$) of **2-MeO-PTH**

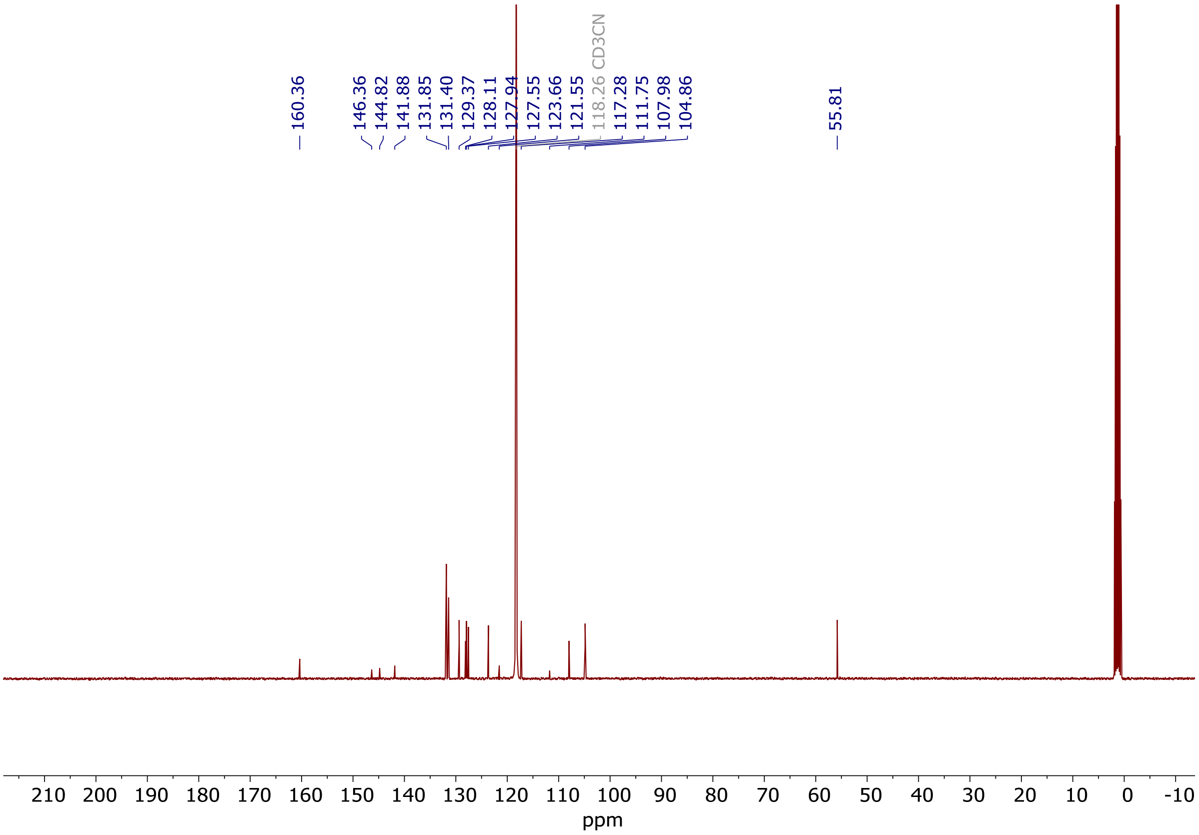

**$^{1}$H NMR** (400 MHz, $CD_3CN$) see procedure

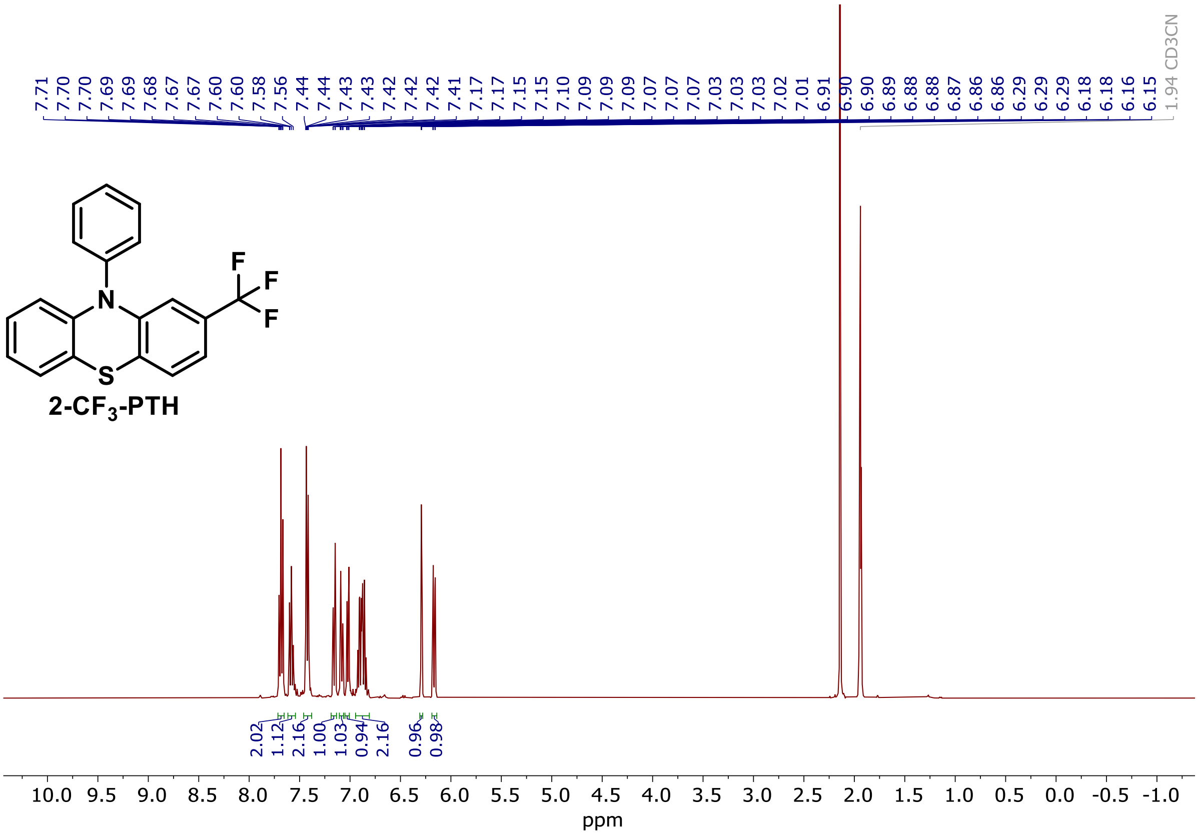


**$^{13}C\{^{19}F\}$ NMR** (126 MHz, $CD_3CN$) of **2-$CF_3$-PTH**

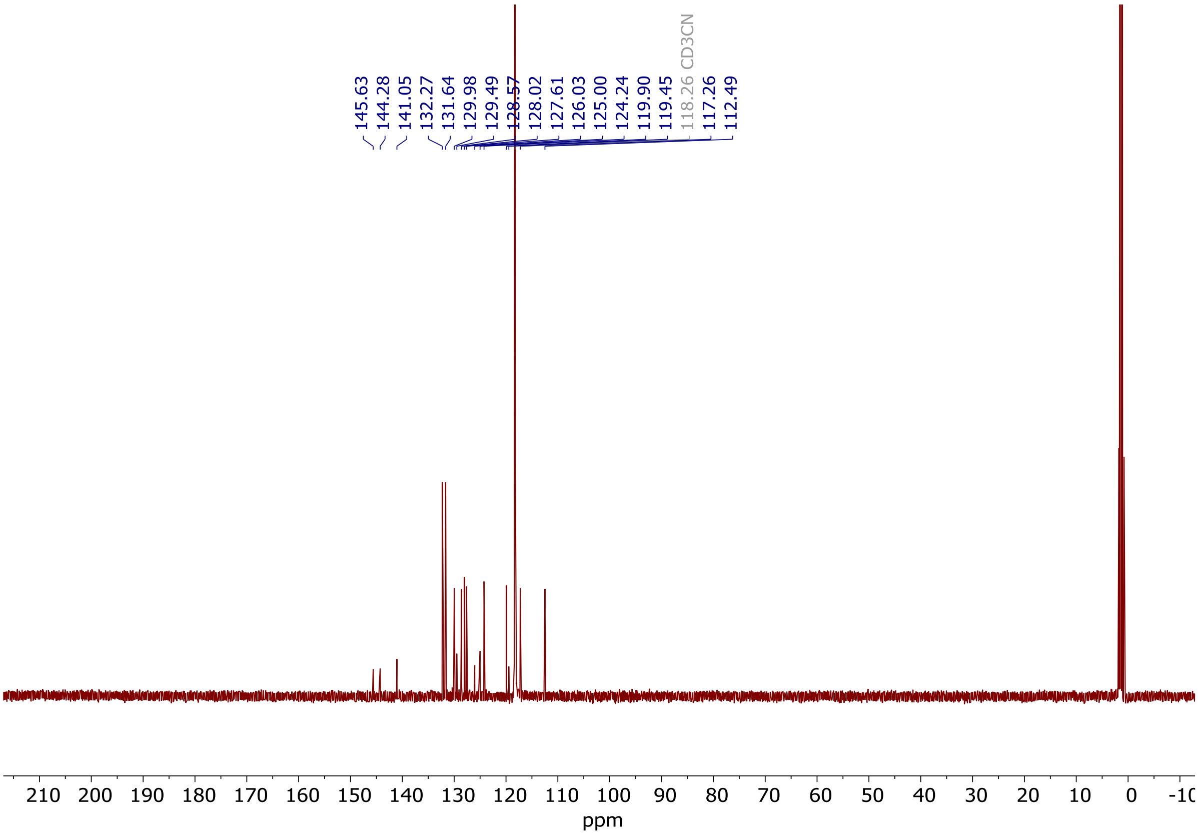

**$^{19}$F NMR** (376 MHz, $CD_3CN$) of **2-$CF_3$-PTH**

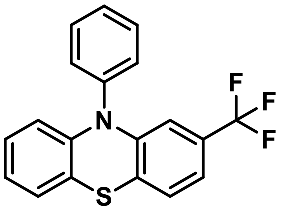

**2-$CF_3$-PTH**

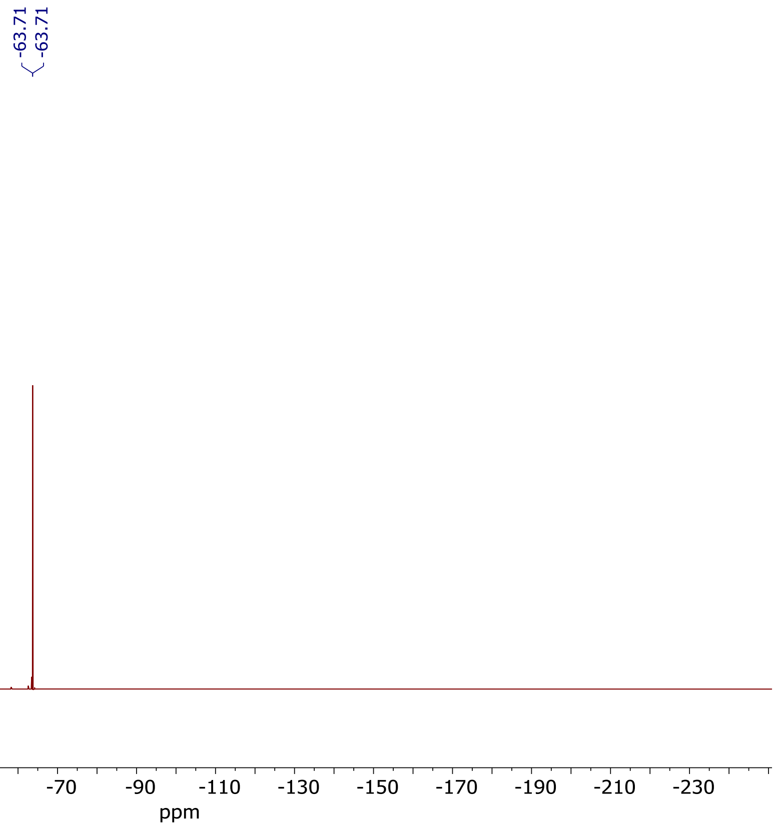

$^{1}$H NMR (599 MHz, $CDCl_3$) of **2a** see procedure

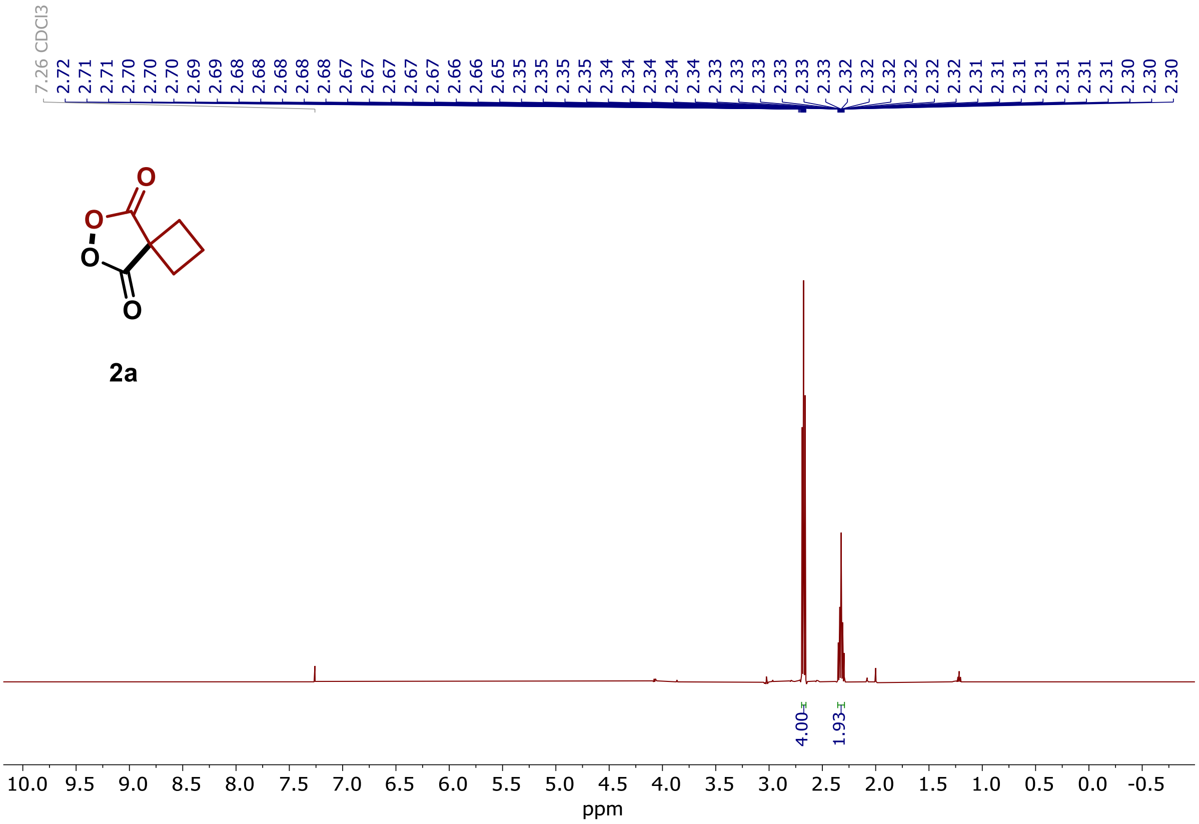


**2a**

$^{13}$C NMR (151MHz, $CDCl_3$) of **2a**

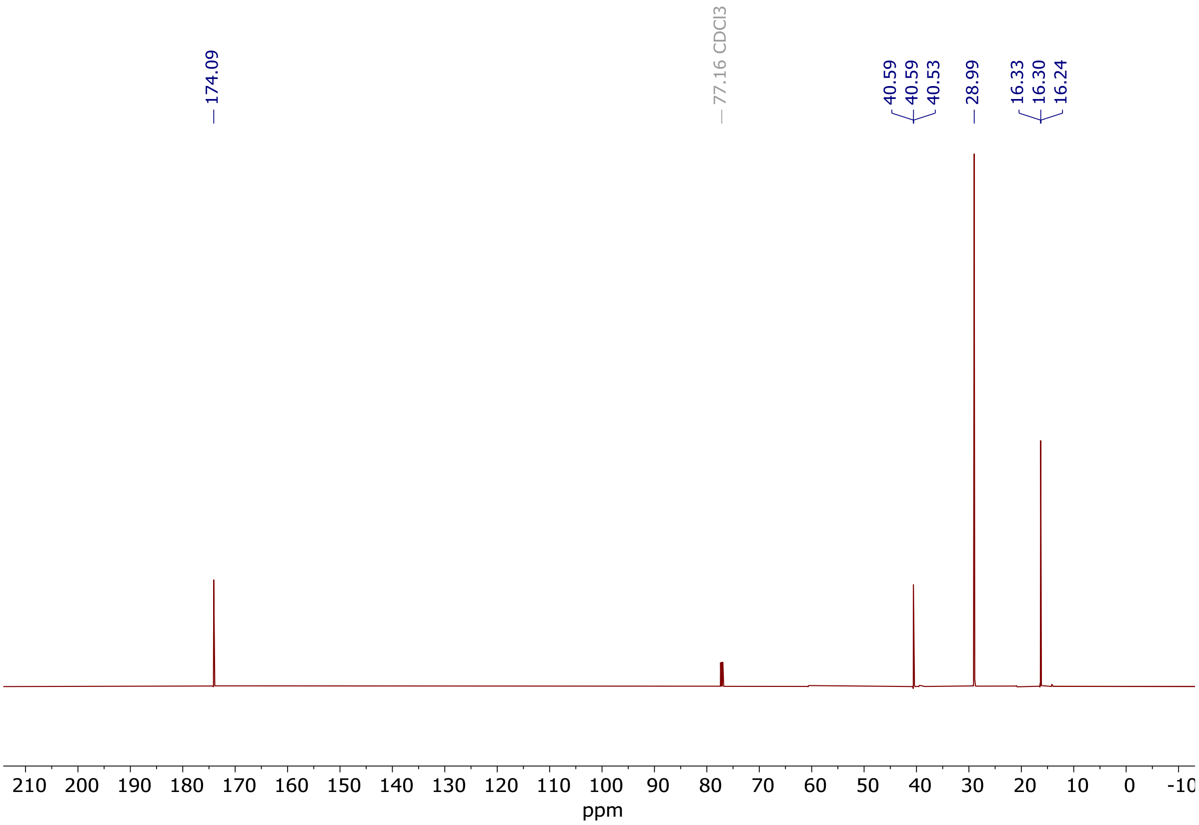

**$^{1}$H NMR** (599 MHz, $CDCl_3$) of **2b** see procedure

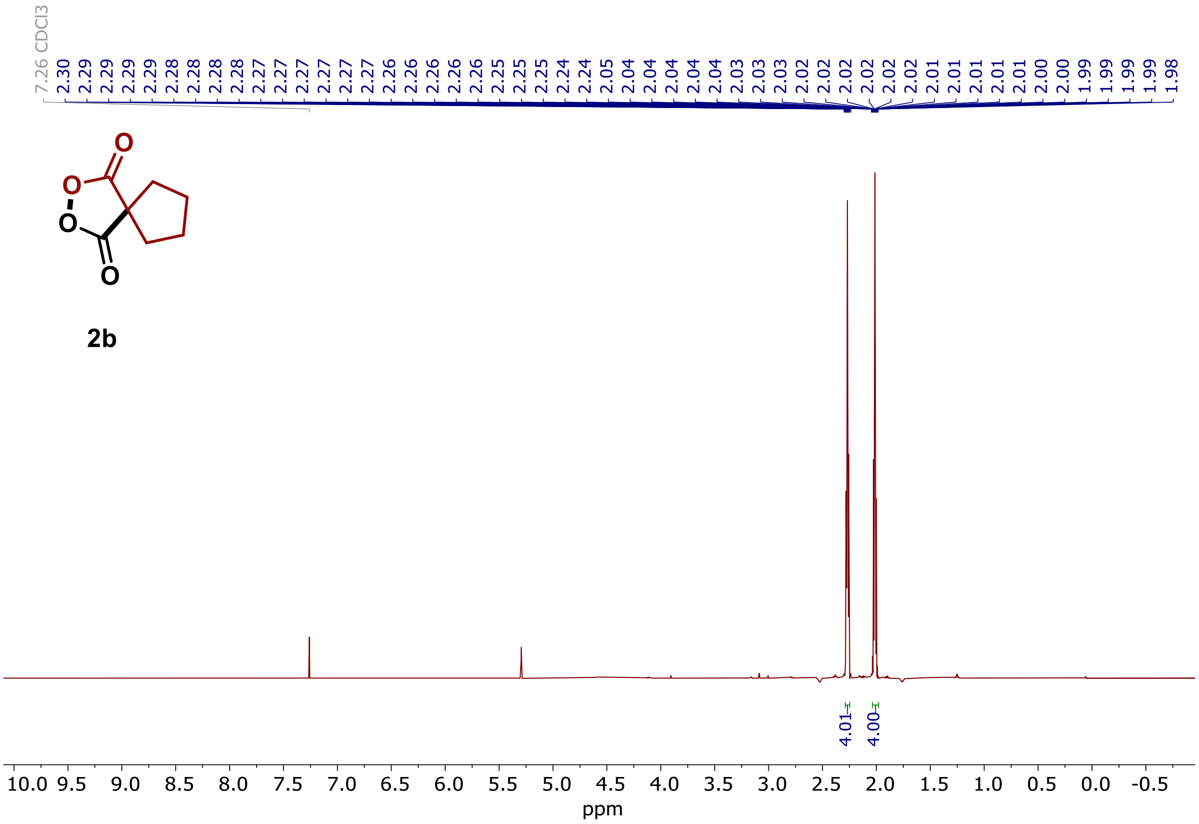


**$^{13}$C NMR** (151MHz, $CDCl_3$) of **2b**

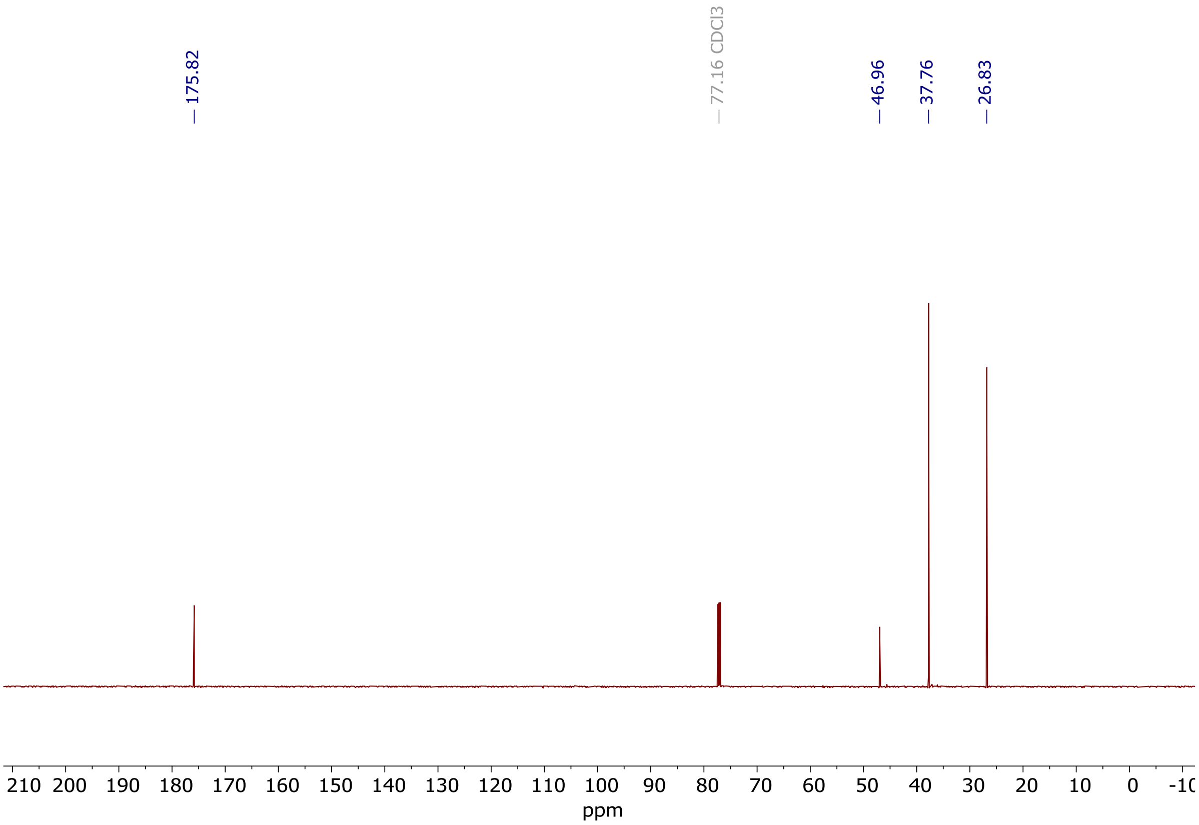

**$^1$H NMR** (599 MHz, $CDCl_3$) of **2c** see procedure

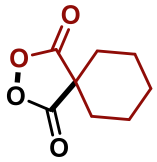

**2c**

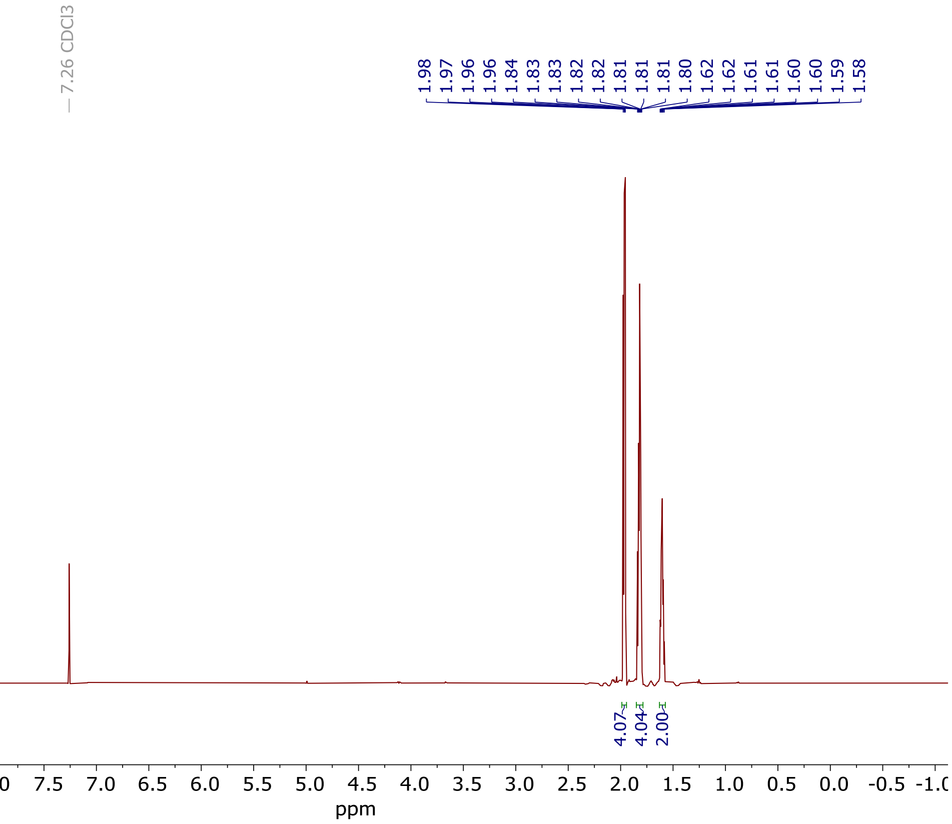


**$^{13}$C NMR** (151MHz, $CDCl_3$) of **2c**

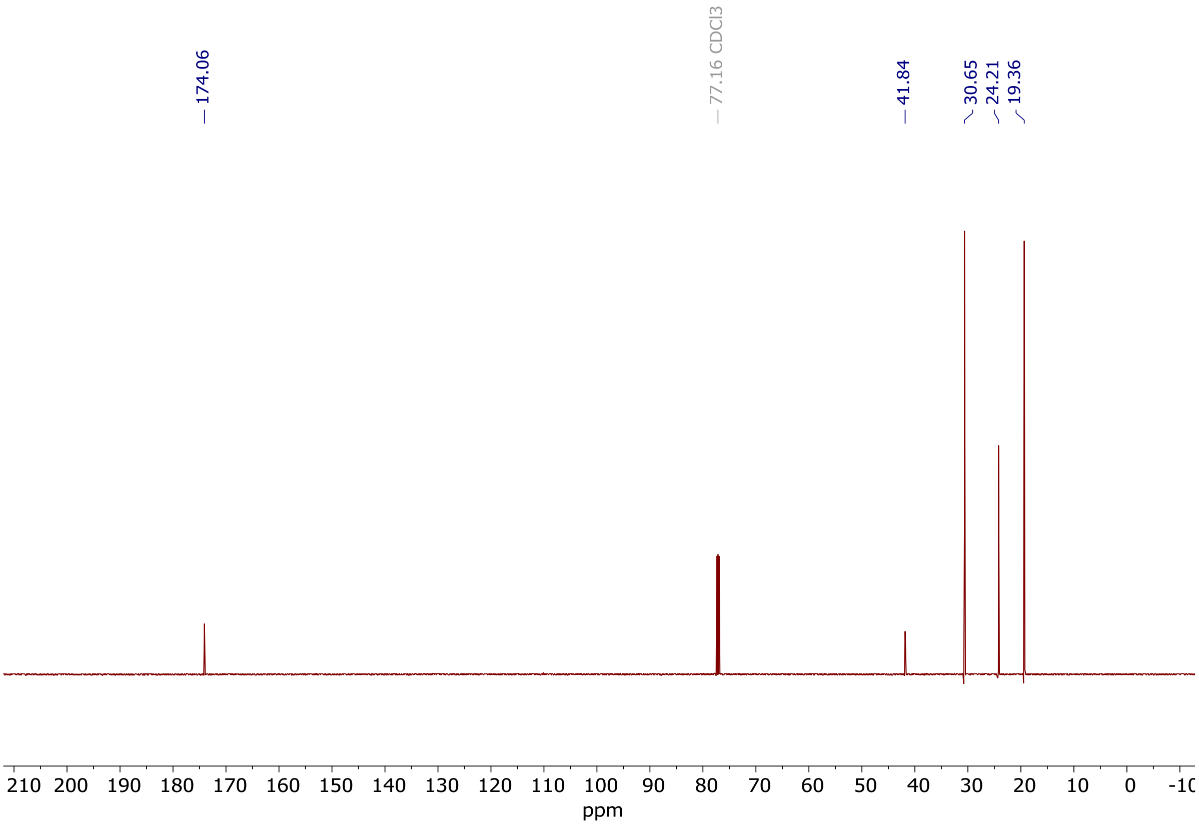

**$^{1}$H NMR** (599 MHz, CDCl3) of **2d** see procedure

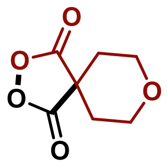

**2d**

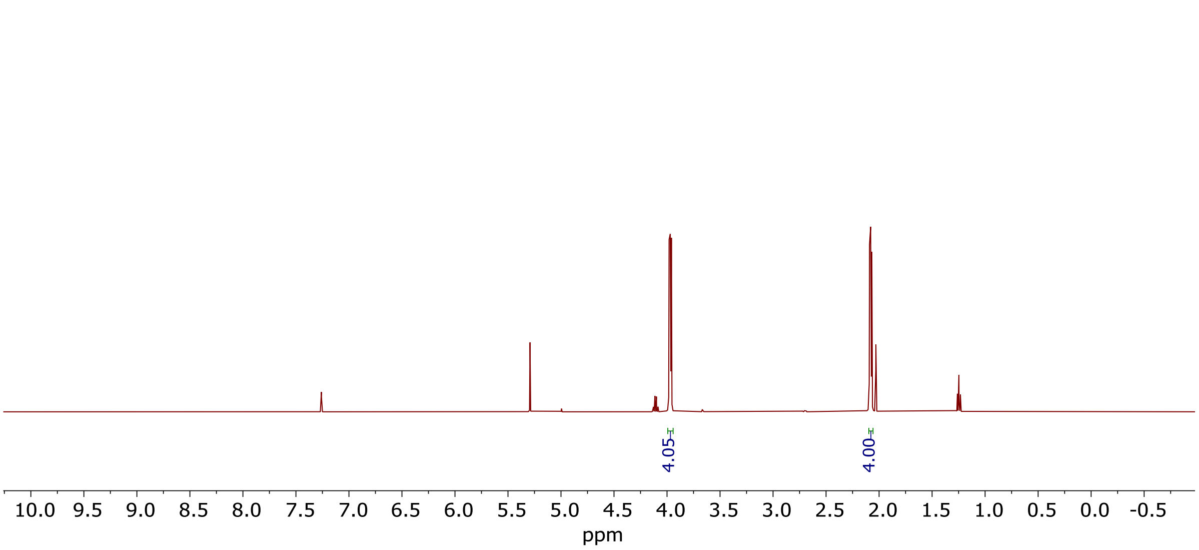


**$^{13}$C NMR** (151MHz, CDCl3) of **2d**

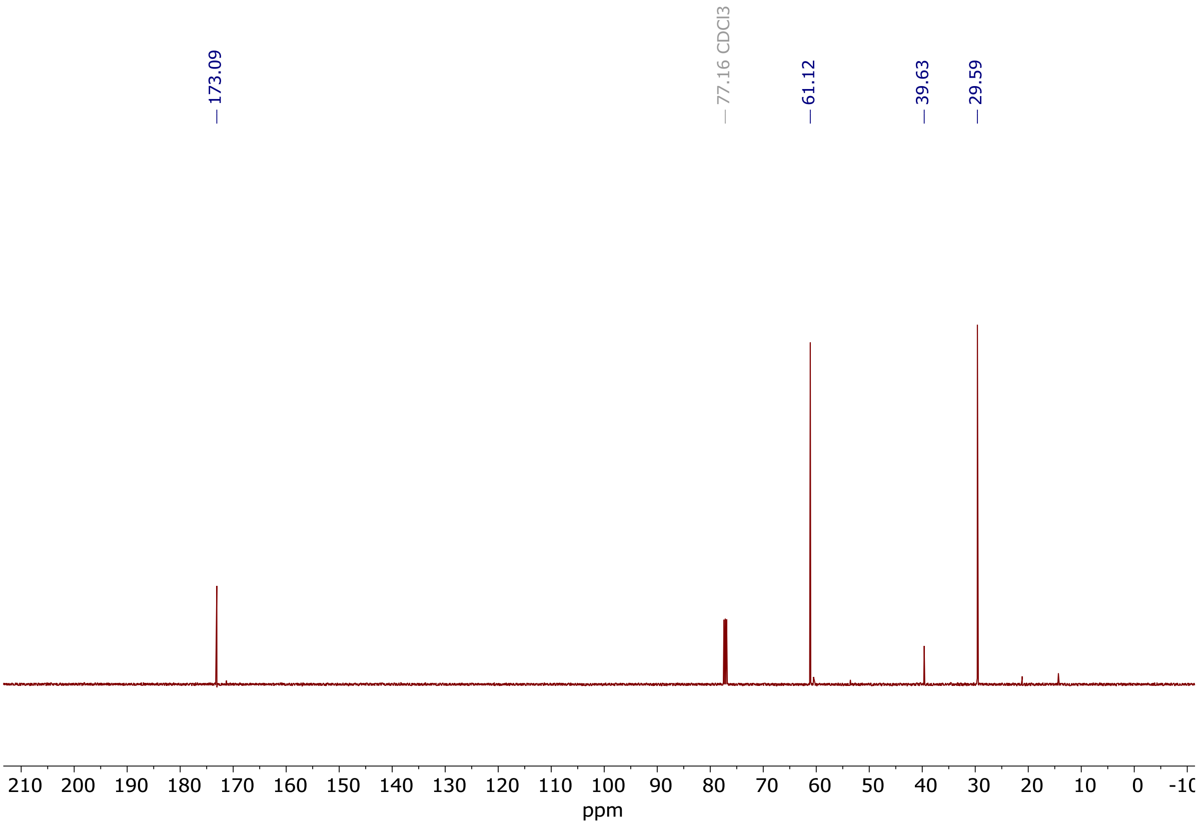

**$^{1}$H NMR** (599 MHz, $CDCl_3$) of **2e** see procedure

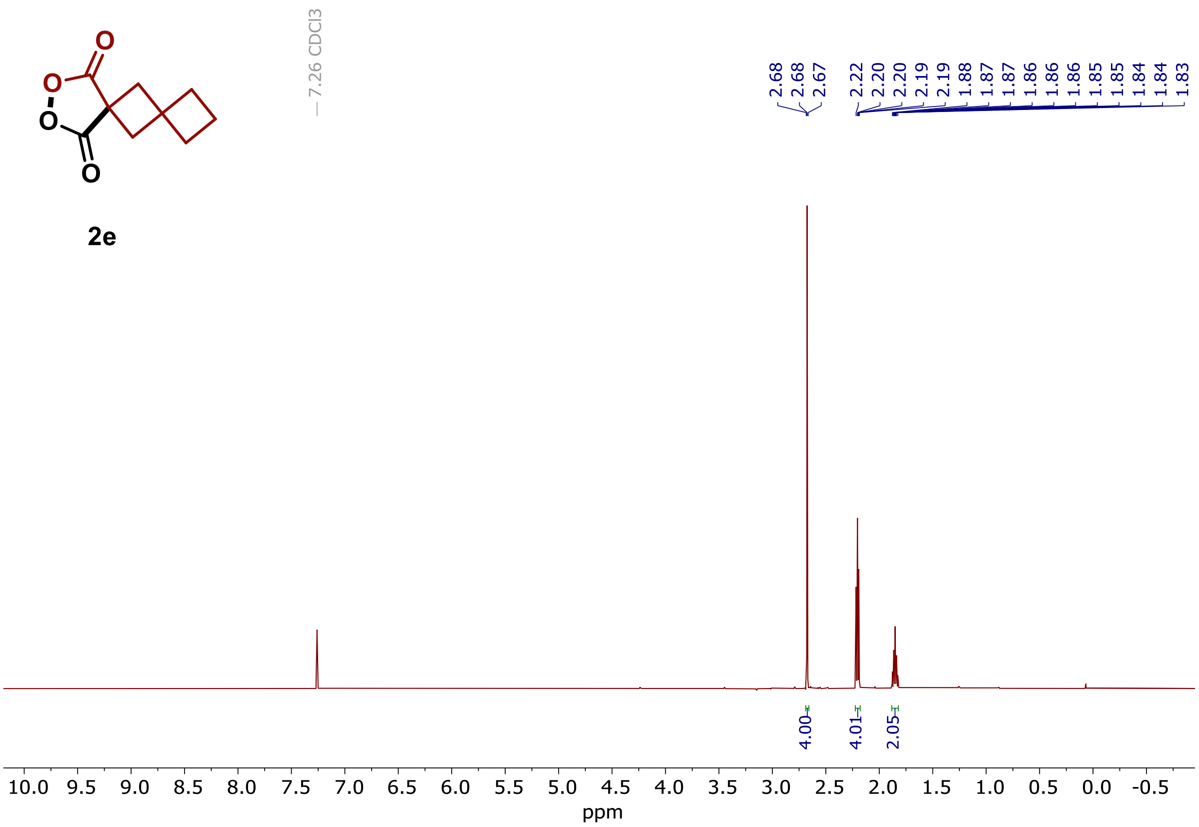


**$^{13}$C NMR** (151MHz, $CDCl_3$) of **2e**

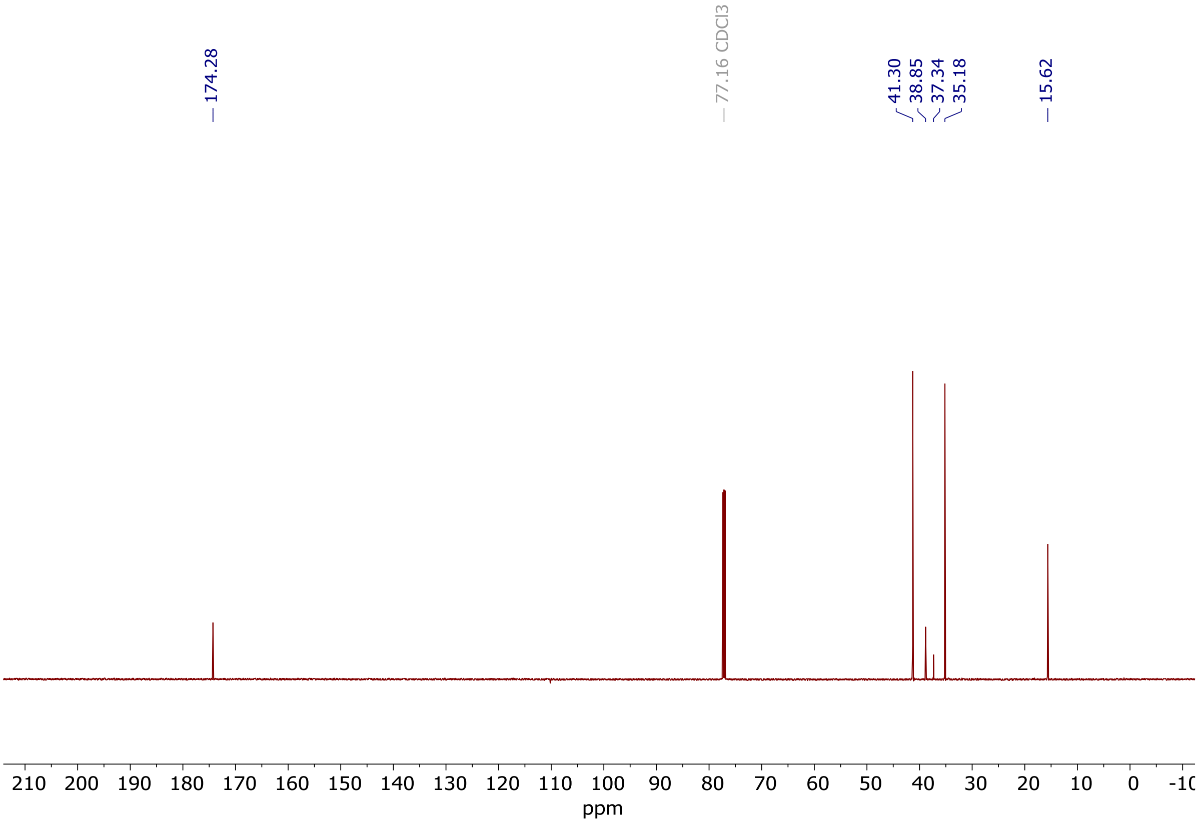

**$^{1}$H NMR** (599 MHz, CDCl$_3$) of **2f** see procedure

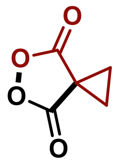

**2f**

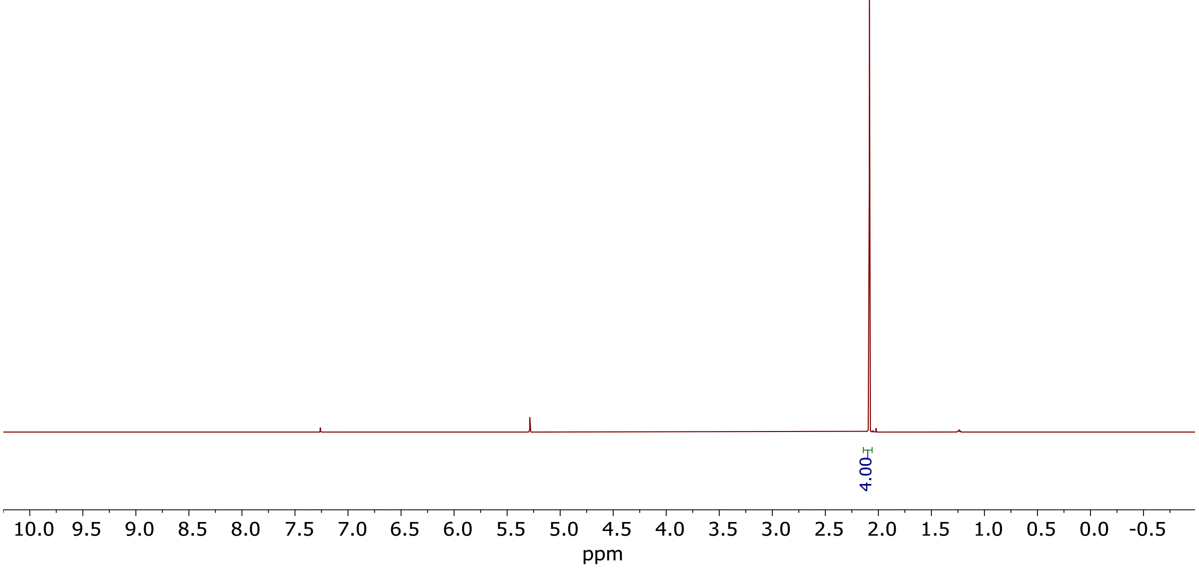


**$^{13}$C NMR** (151MHz, CDCl$_3$) of **2f**

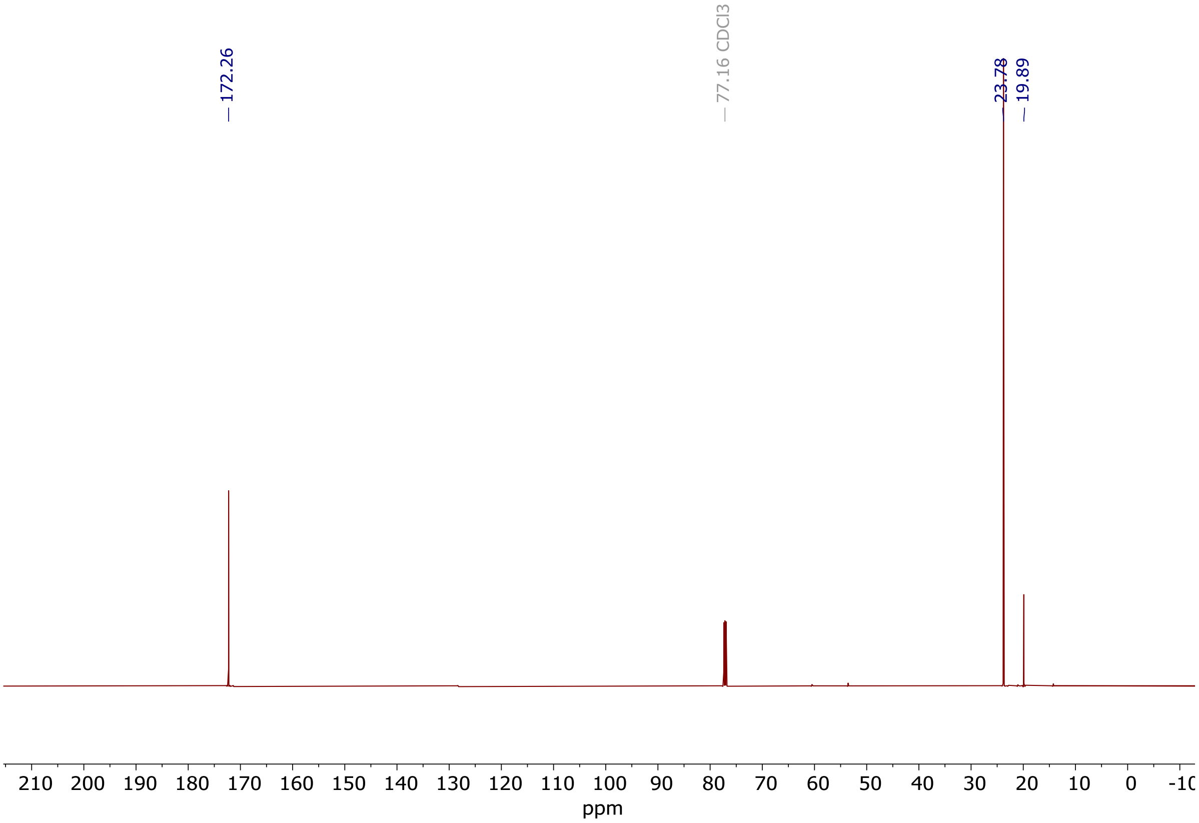

**$^1$H NMR** (599 MHz, $CDCl_3$) of **2g** see procedure

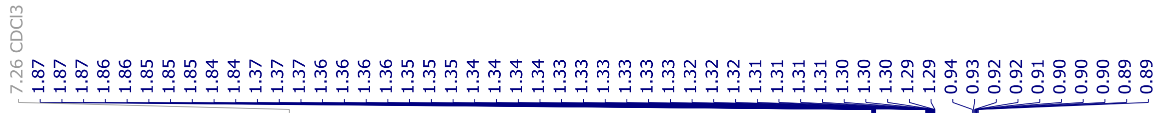

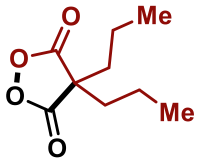

**2g**

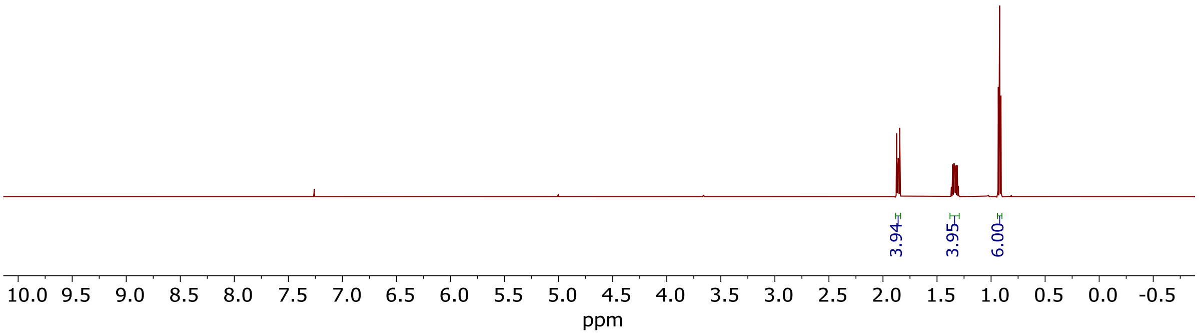

**$^{13}$C NMR** (151MHz, $CDCl_3$) of **2g**

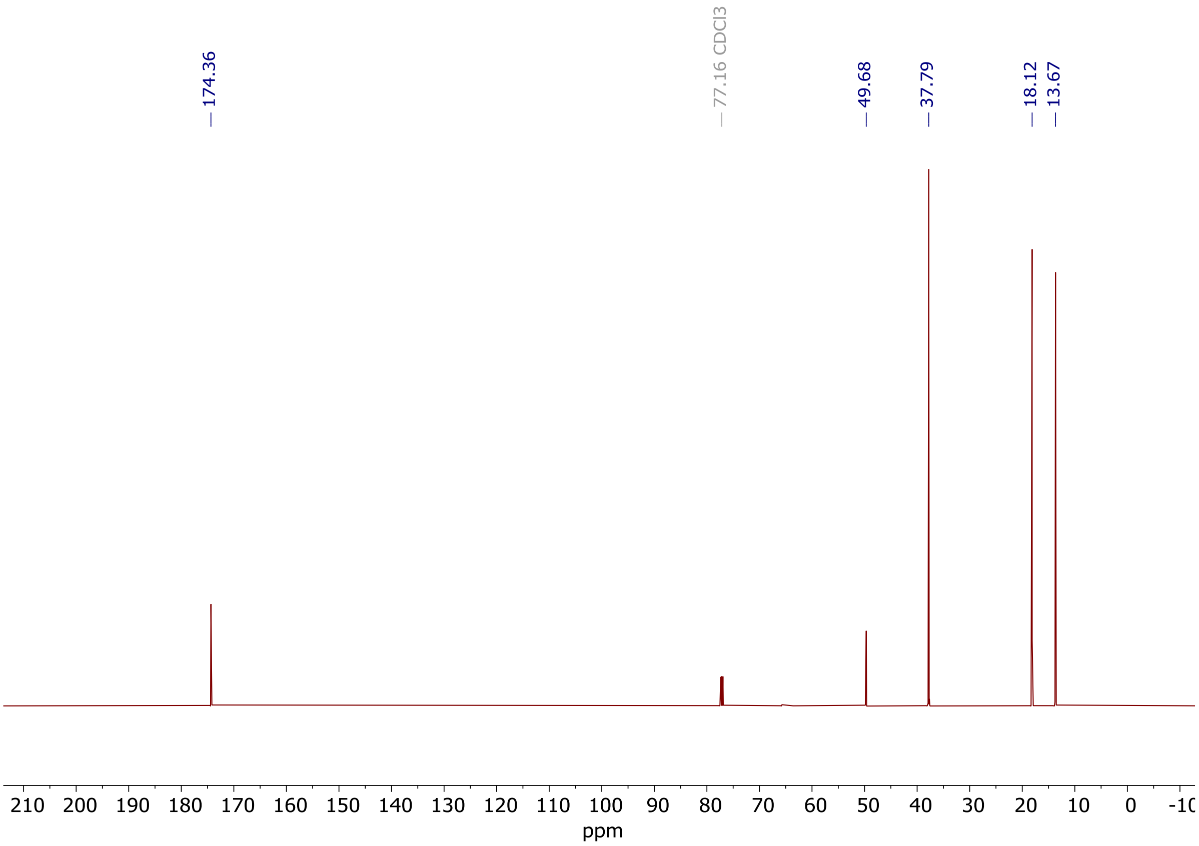

**$^{1}$H NMR** (599 MHz, $CDCl_3$) of **2h** see procedure

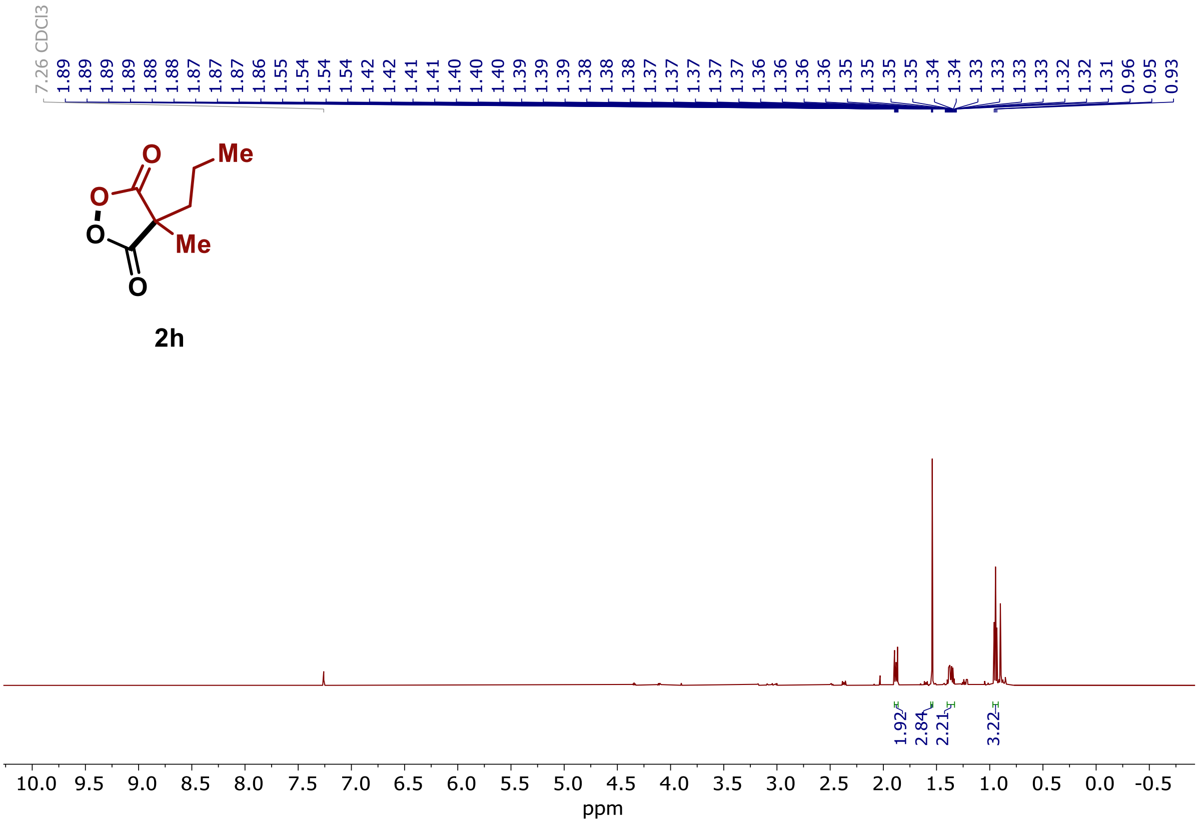


**$^{13}$C NMR** (151MHz, $CDCl_3$) of **2h**

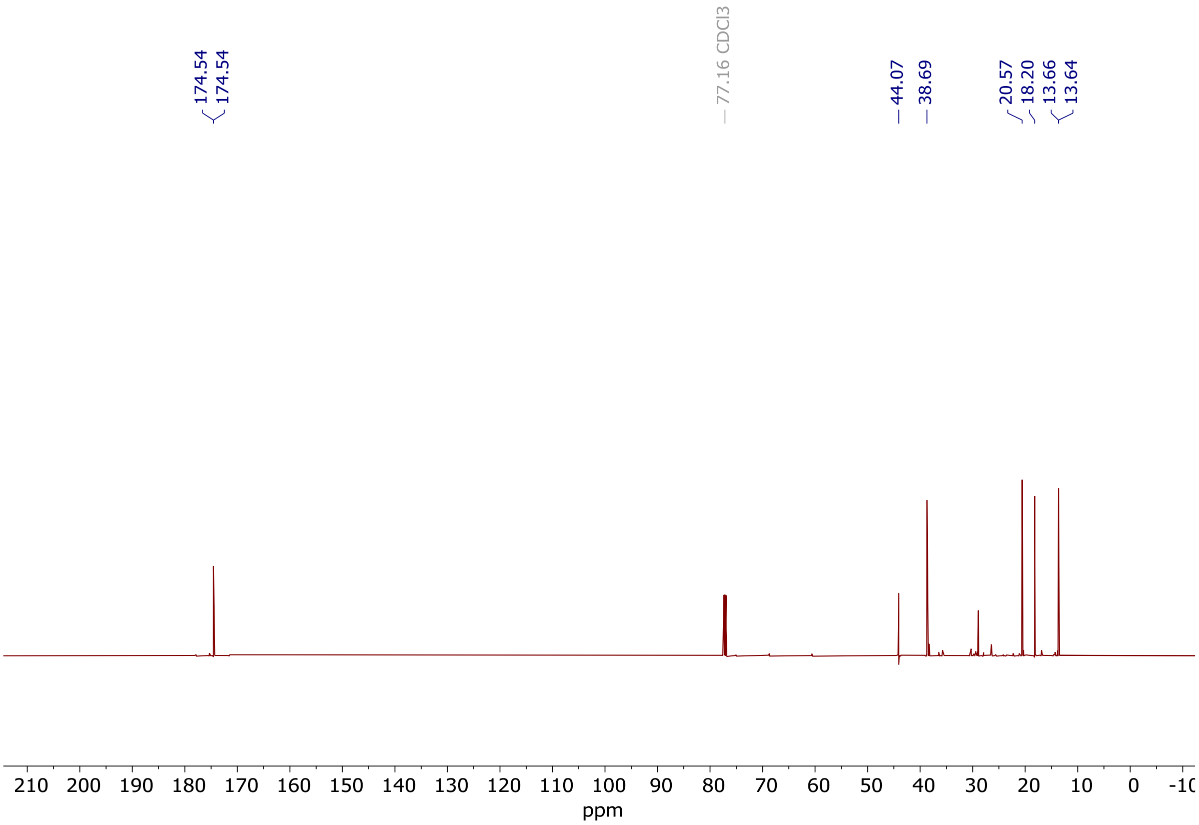

**¹H NMR** (599 MHz, $CDCl_3$) of **2j** see procedure

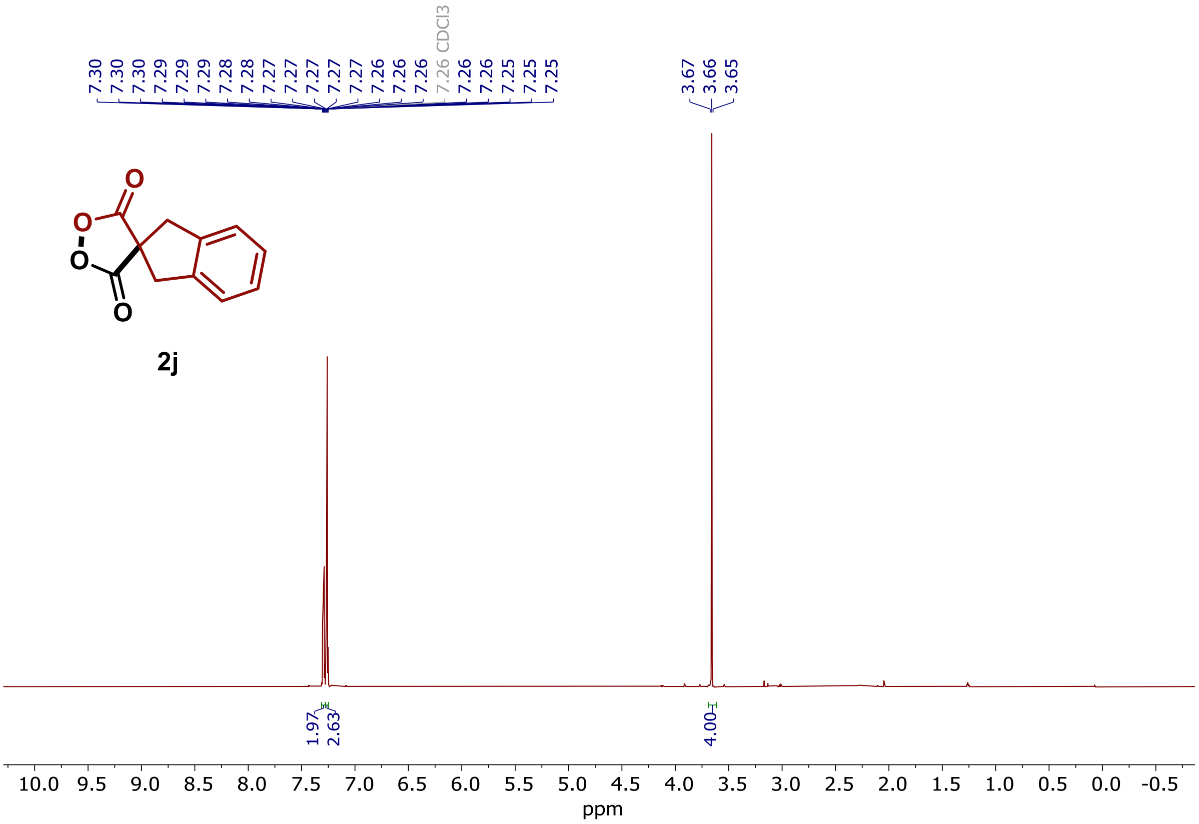


**¹³C NMR** (151MHz, $CDCl_3$) of **2j**

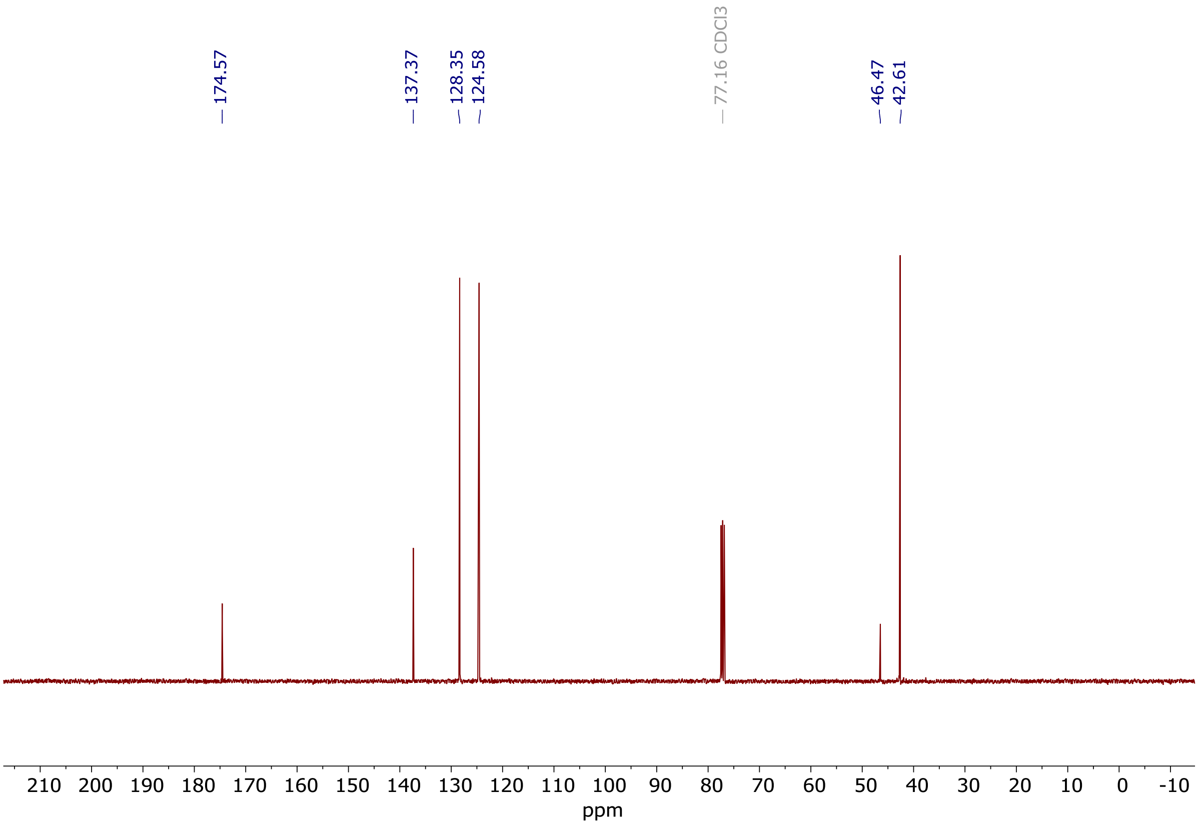

**$^1$H NMR** (599 MHz, $CDCl_3$) of **S1-2d** see procedure

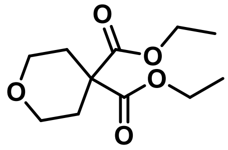

**S1-2d**

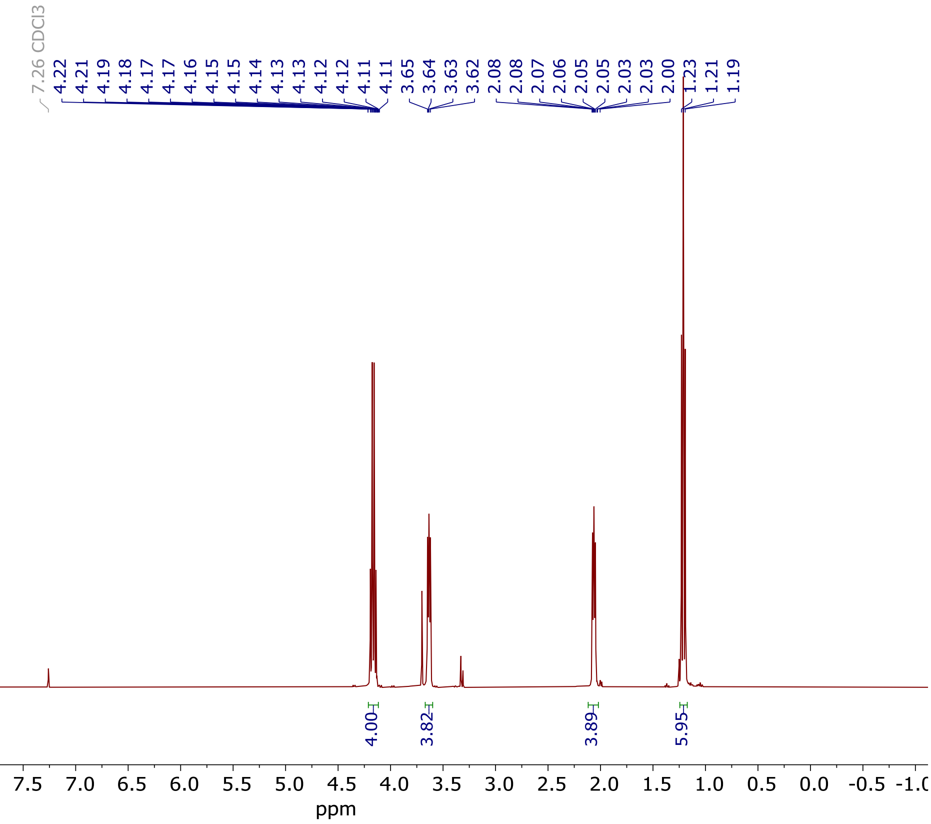

**$^{1}$H NMR** (400 MHz, DMSO-$d_6$) of **S2-2d** see procedure

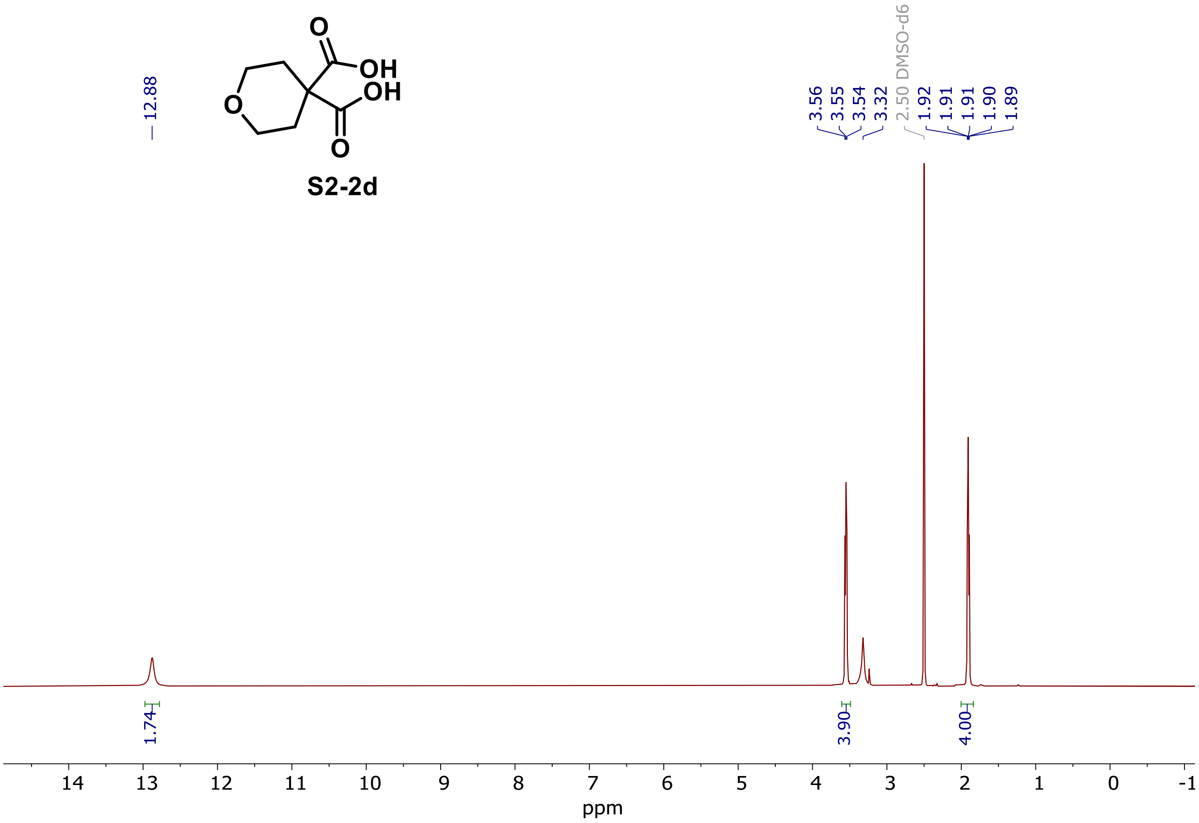


**$^{13}$C NMR** (400 MHz, DMSO-$d_6$) of **S2-2d**

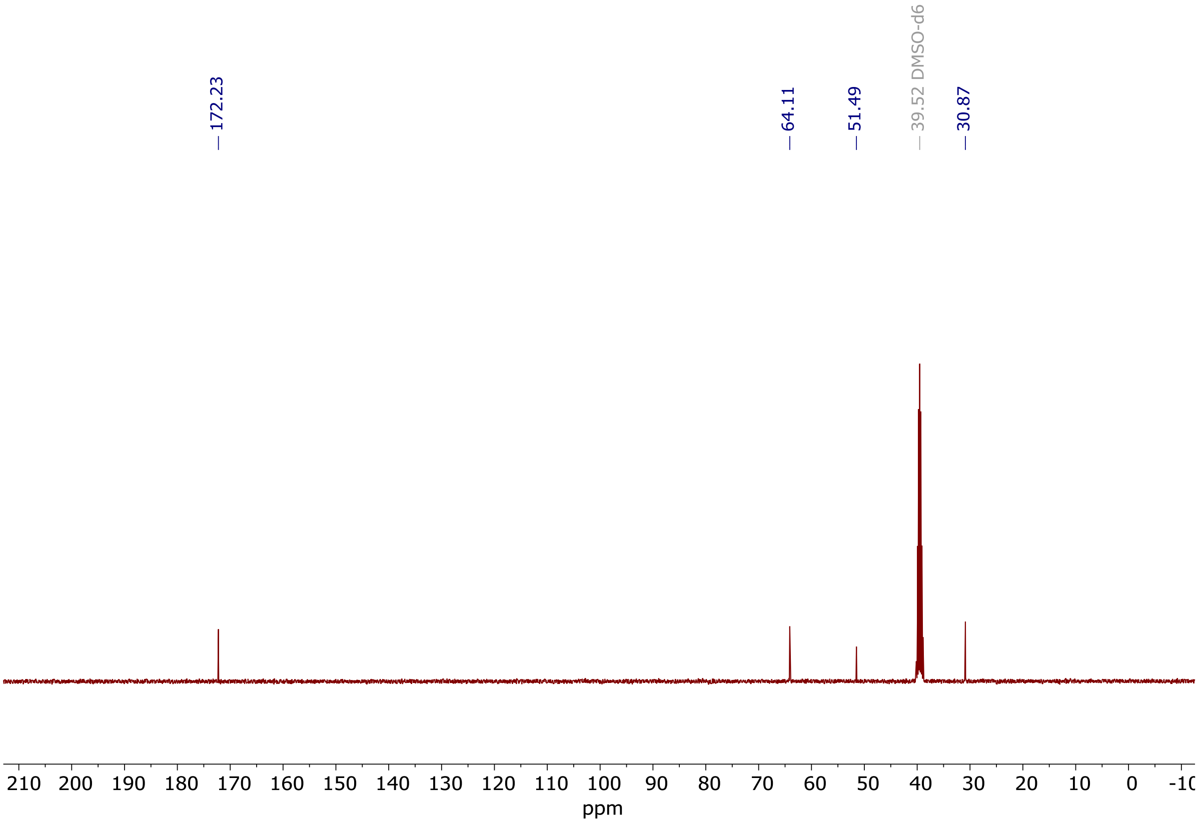

$^{1}$H NMR (400 MHz, $CDCl_3$) of **S1-2e** see procedure

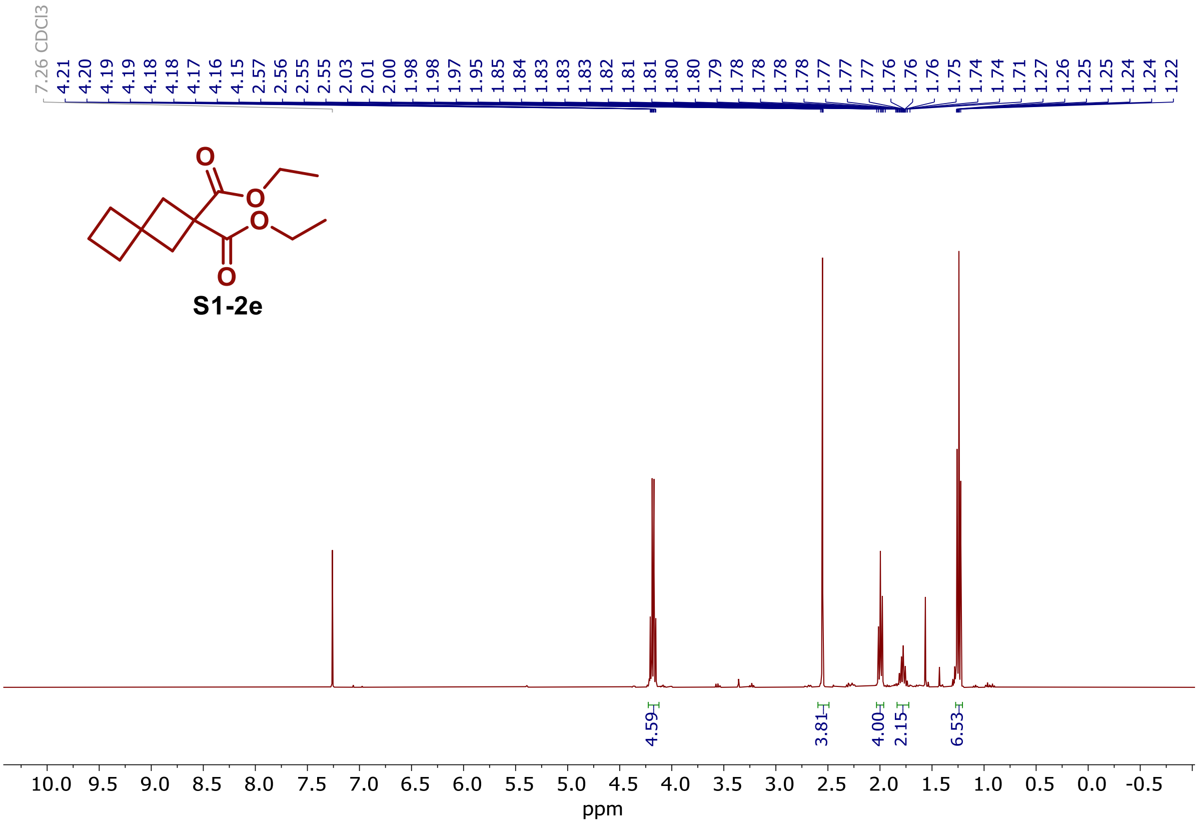


$^{13}$C NMR (101MHz, $CDCl_3$) of **S1-2e**

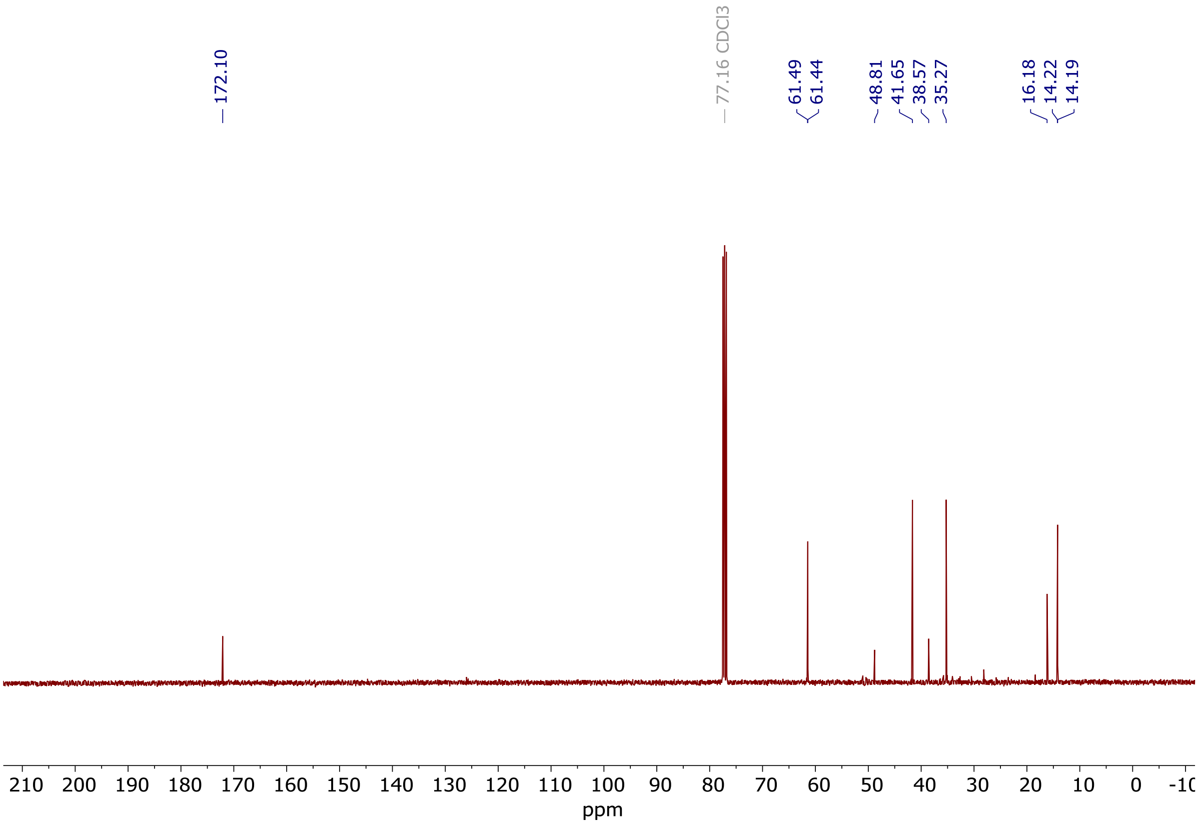

**$^1$H NMR** (400 MHz, DMSO-$d_6$) of **S2-2e** see procedure

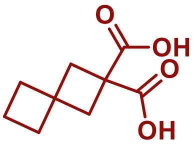

**S2-2e**

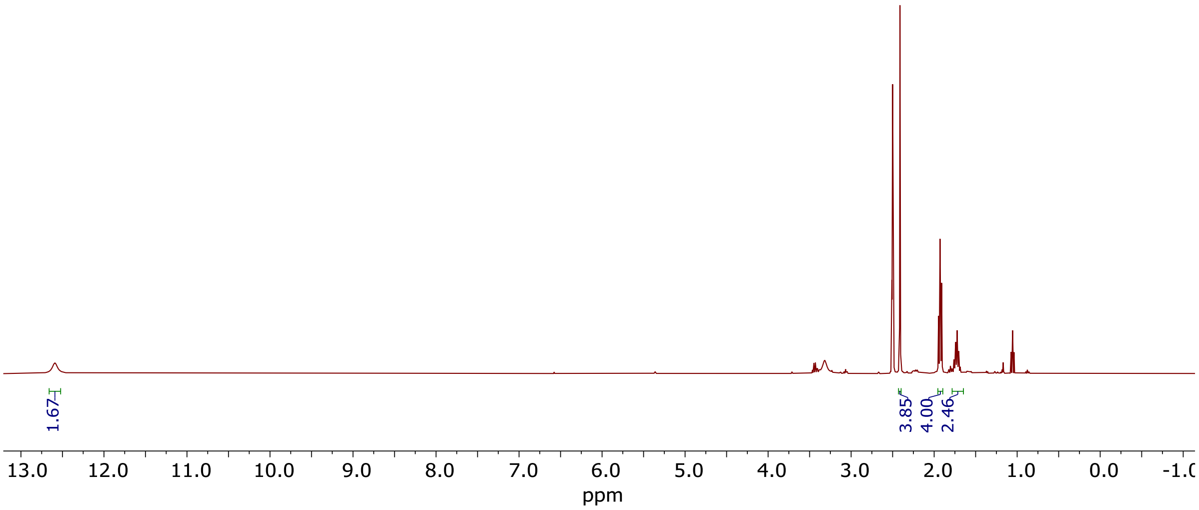


**$^{13}$C NMR** (101MHz, DMSO-$d_6$) of **S2-2e**

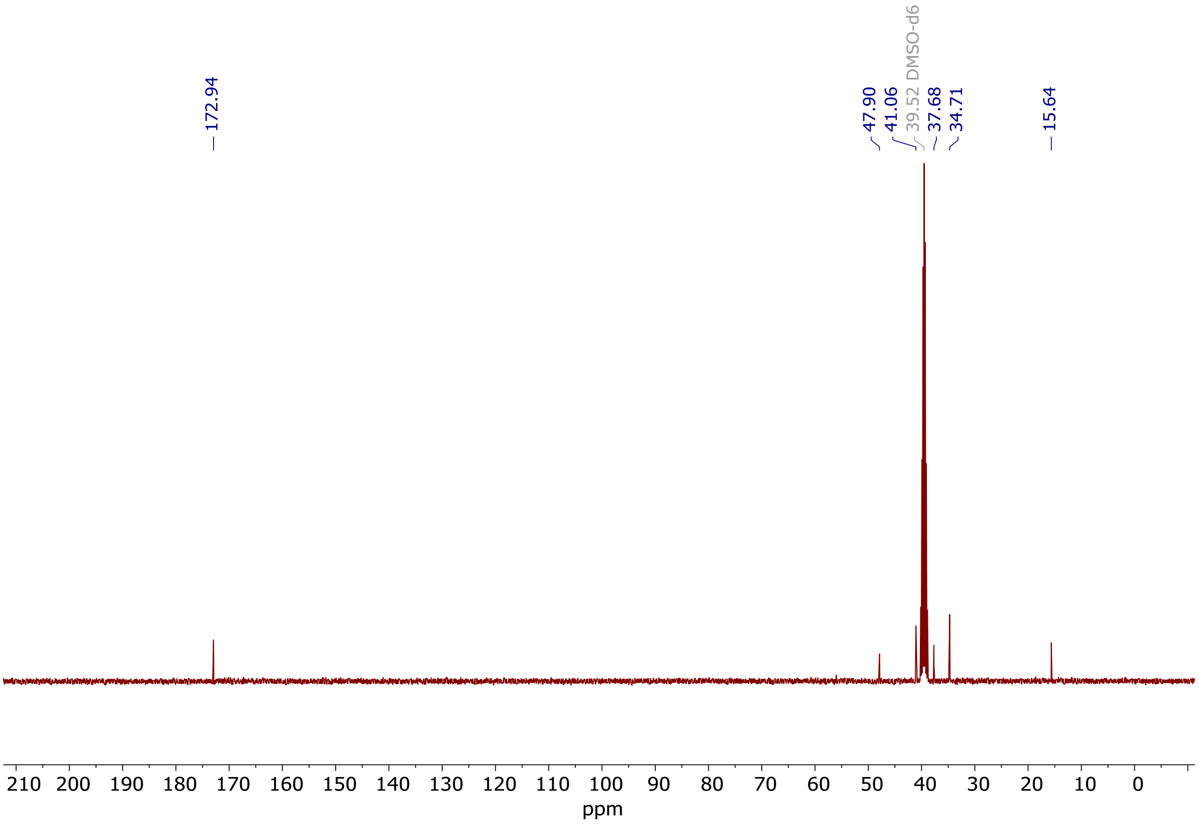

**$^{1}$H NMR** (400 MHz, $CDCl_3$) of **S1-1e** see procedure

$Ph_3P^+CD_3\ I^-$
**S1-1e**

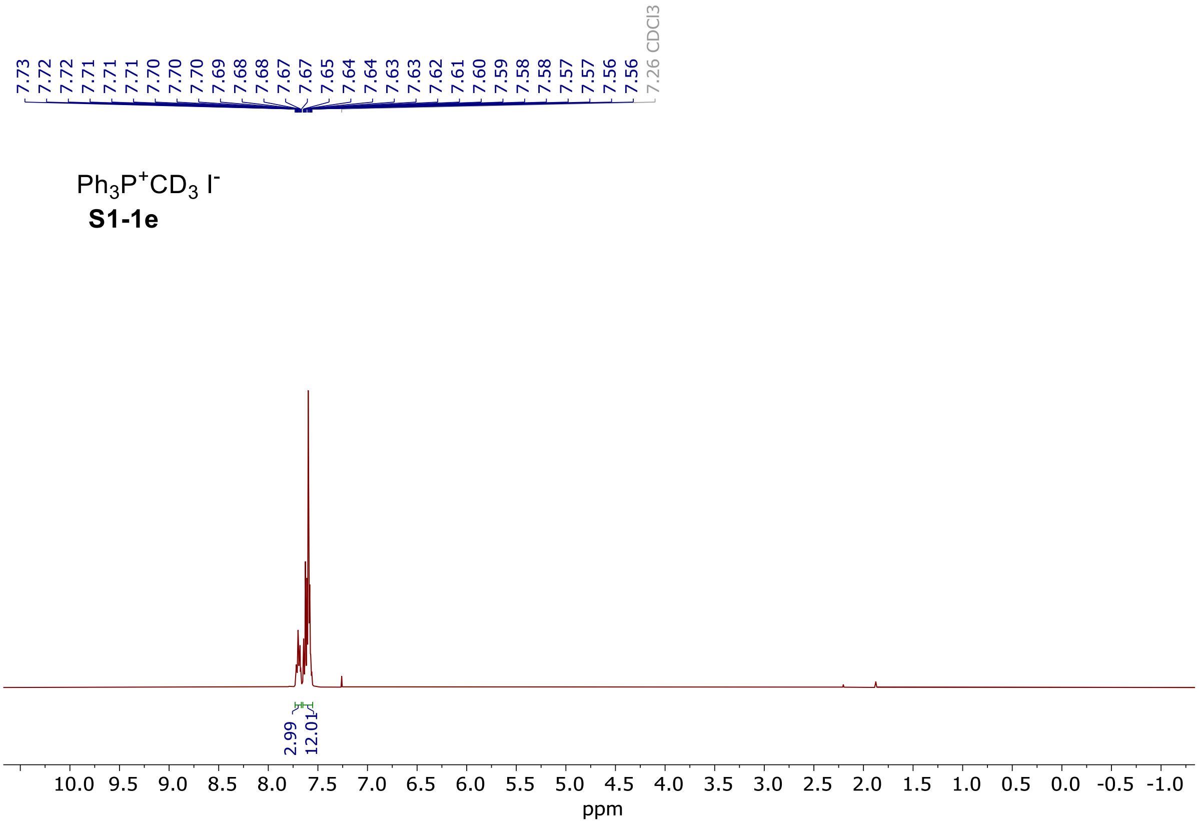


**$^{13}$C NMR** (101MHz, $CDCl_3$) of **S1-1e**

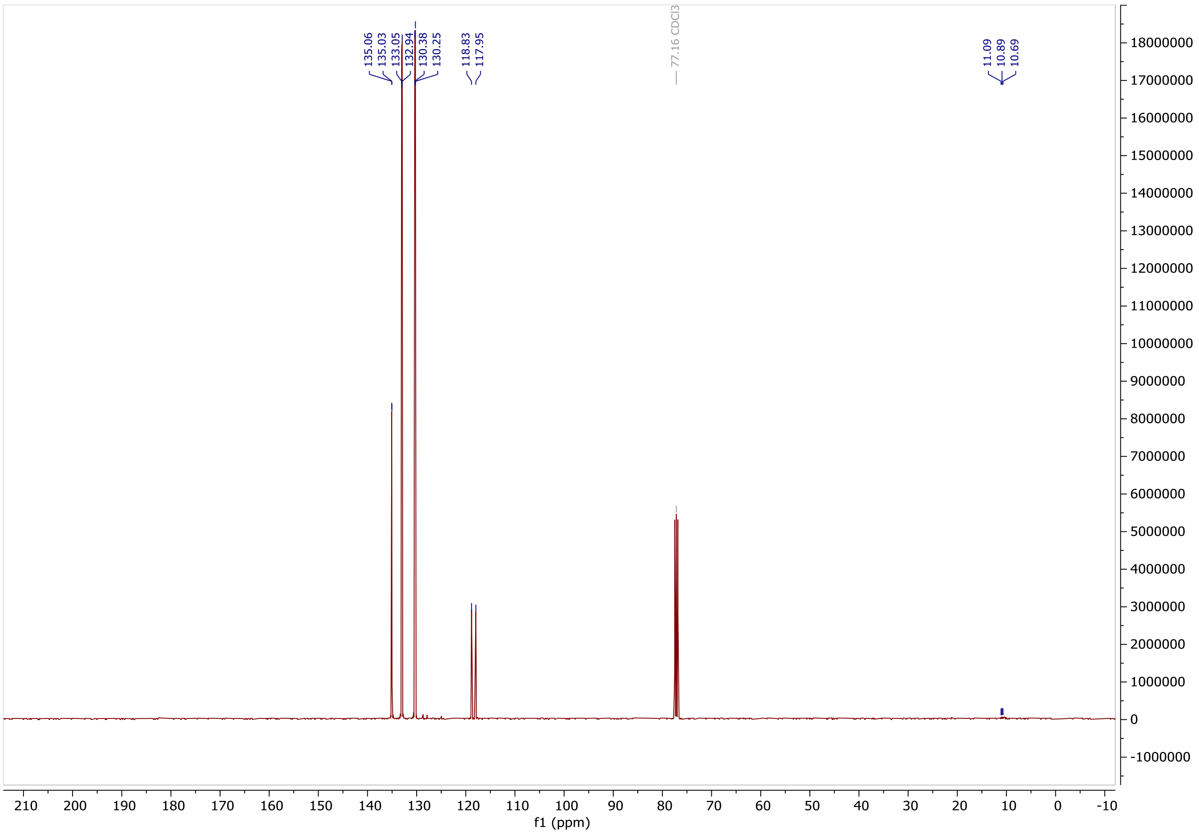

**$^{31}$P NMR** (162MHz, $CDCl_3$) of **S1-1e**

$Ph_3P^+ I^-$
**S1-1e**

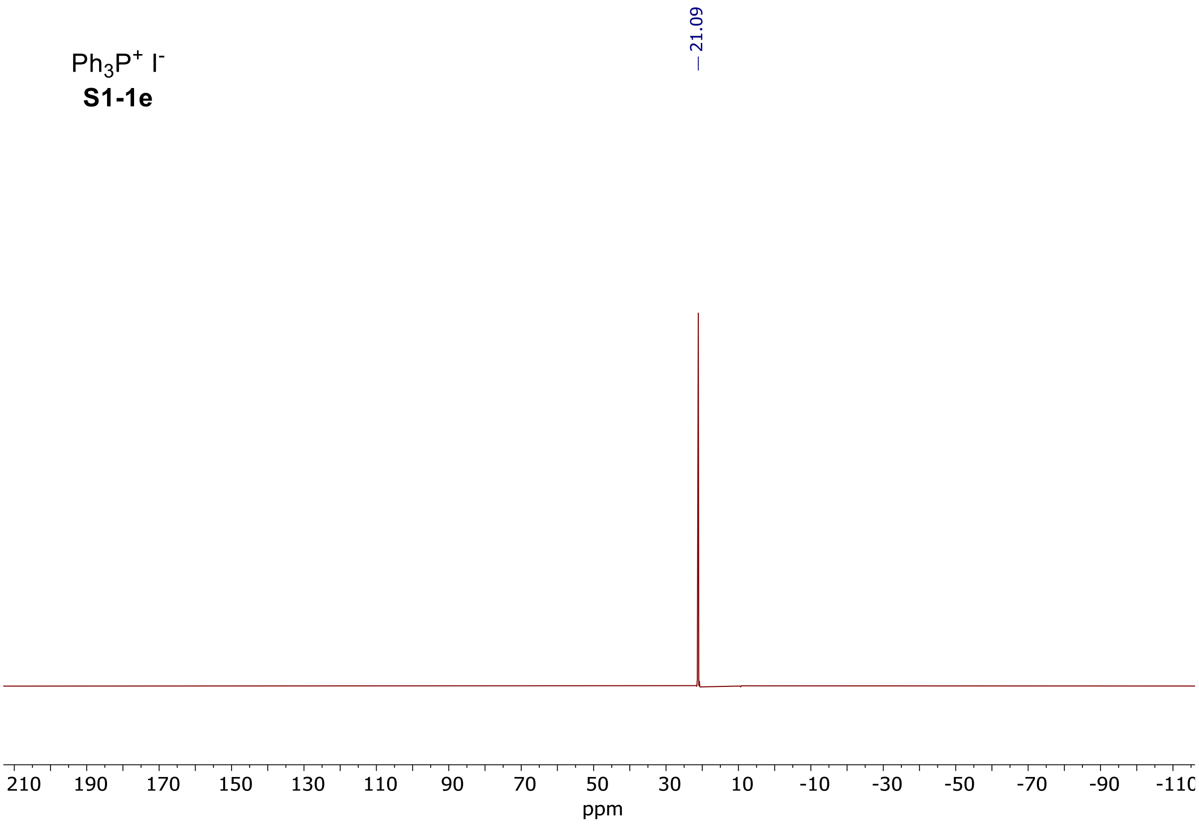

**$^1$H NMR** (500 MHz, $CDCl_3$) of **1e** see procedure

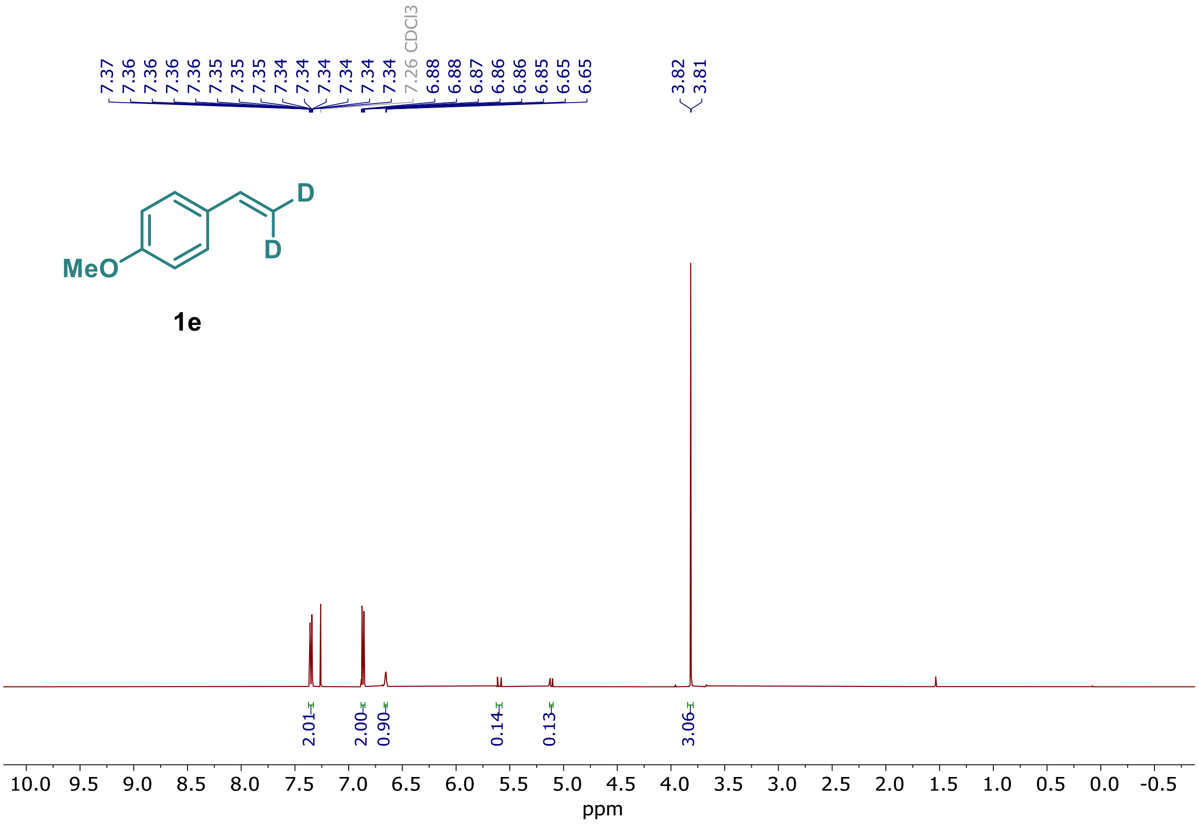


**$^{13}$C NMR** (151MHz, $CDCl_3$) of **1e**

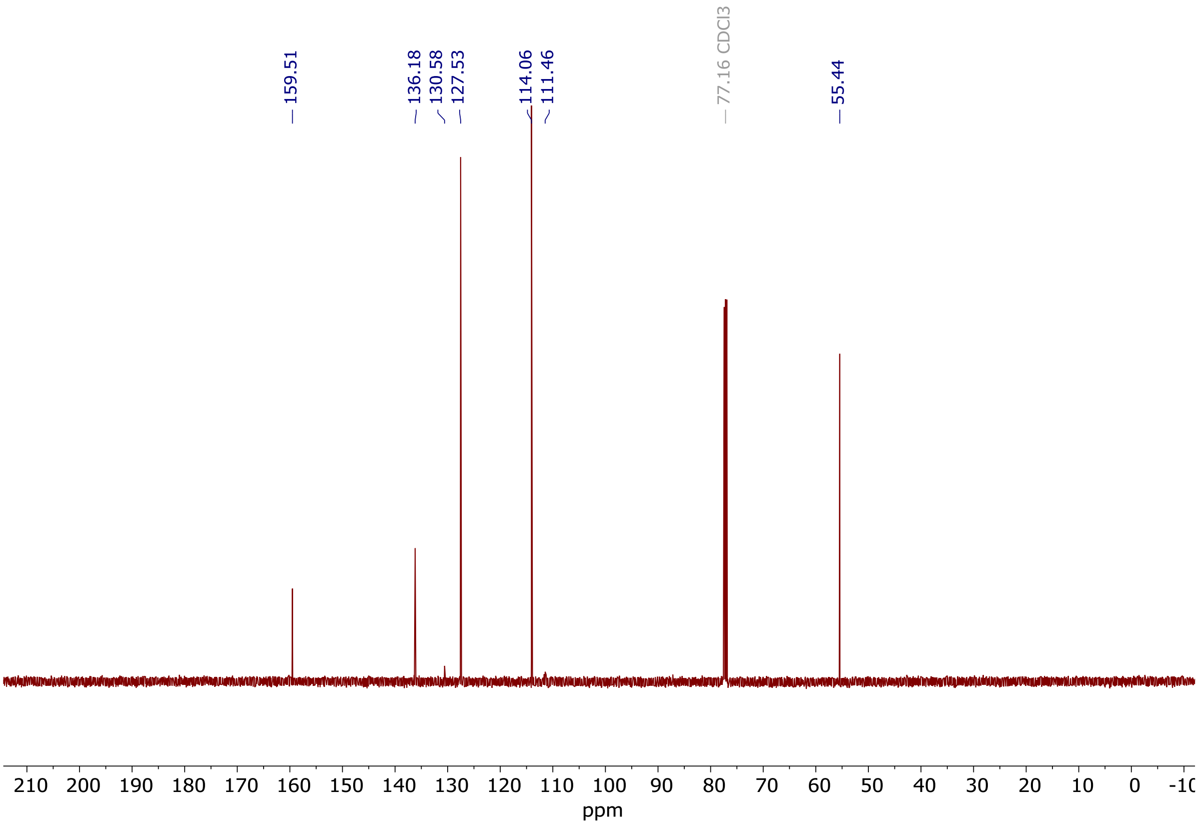

$^{1}$H NMR (400 MHz, $CDCl_3$) of **S1-1f** see procedure

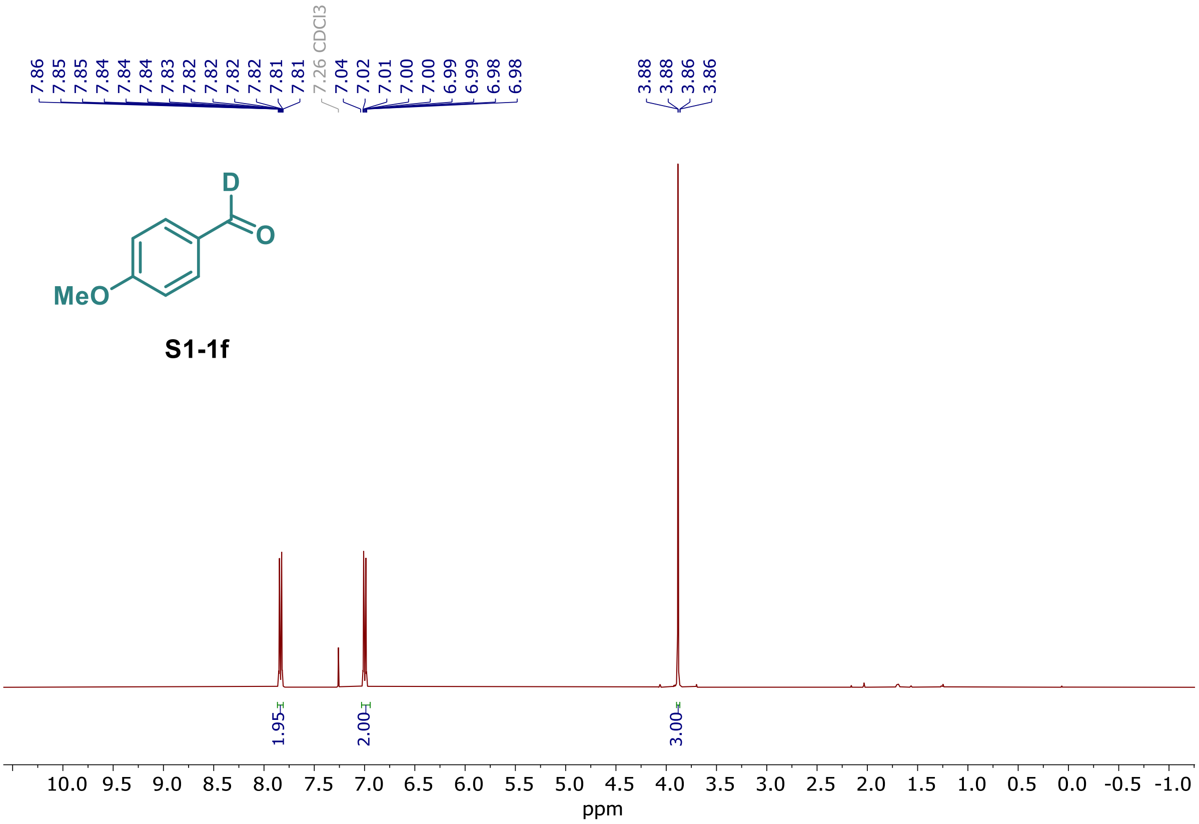


$^{13}$C NMR (101 MHz, $CDCl_3$) of **S1-1f**

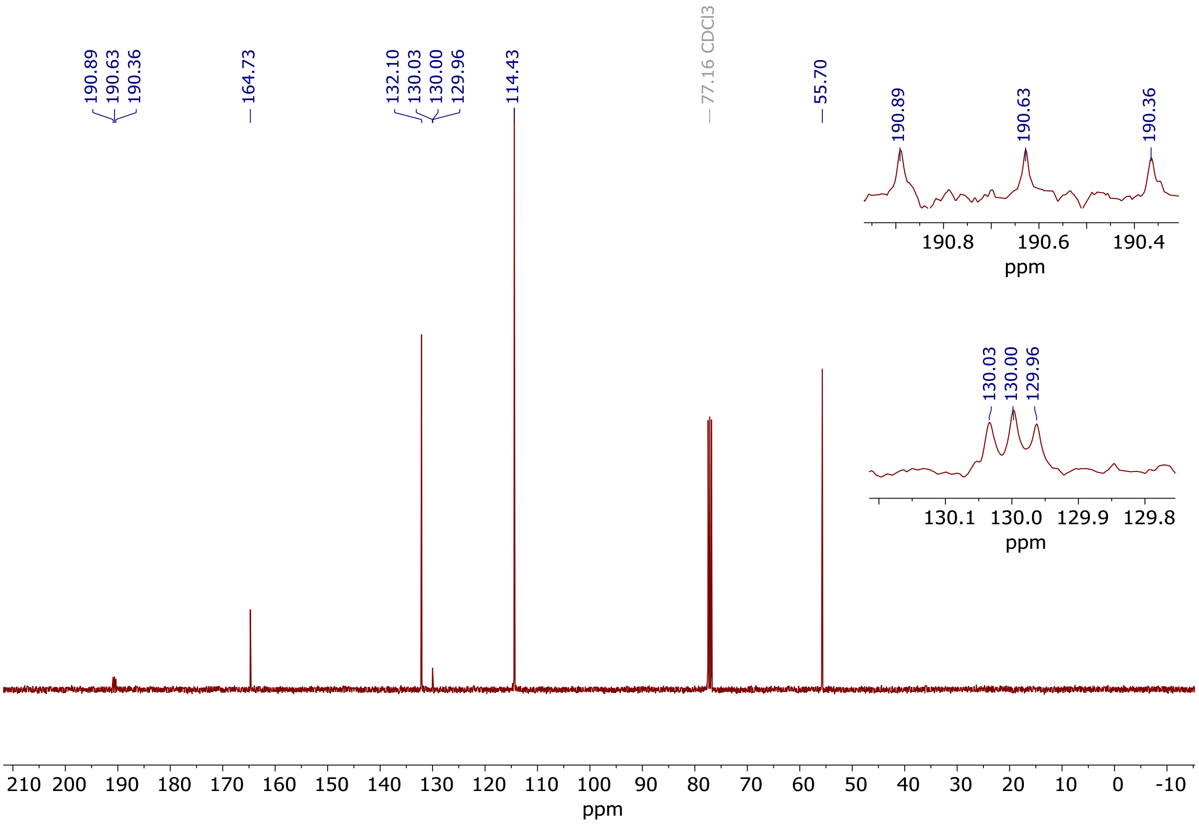

**$^{1}$H NMR** (500 MHz, $CDCl_3$) of **1f** see procedure

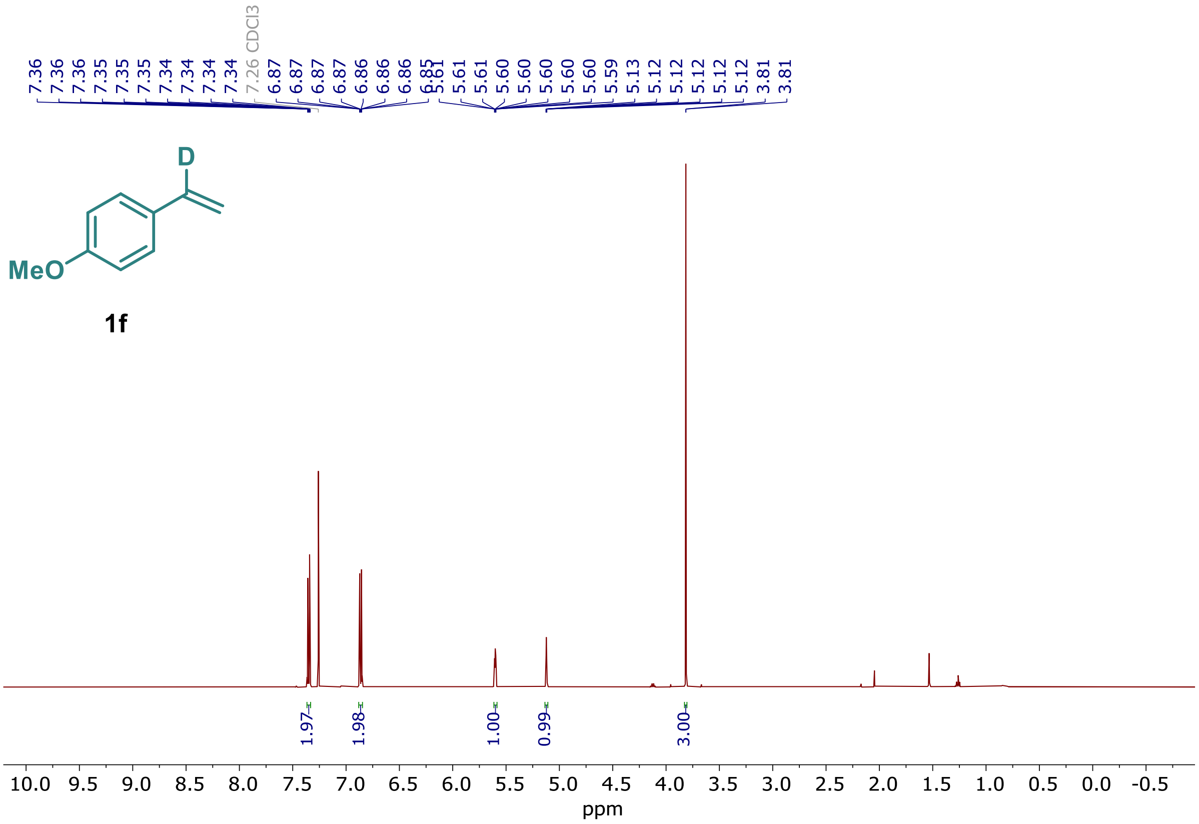


**$^{13}$C NMR** (126MHz, $CDCl_3$) of **1f**

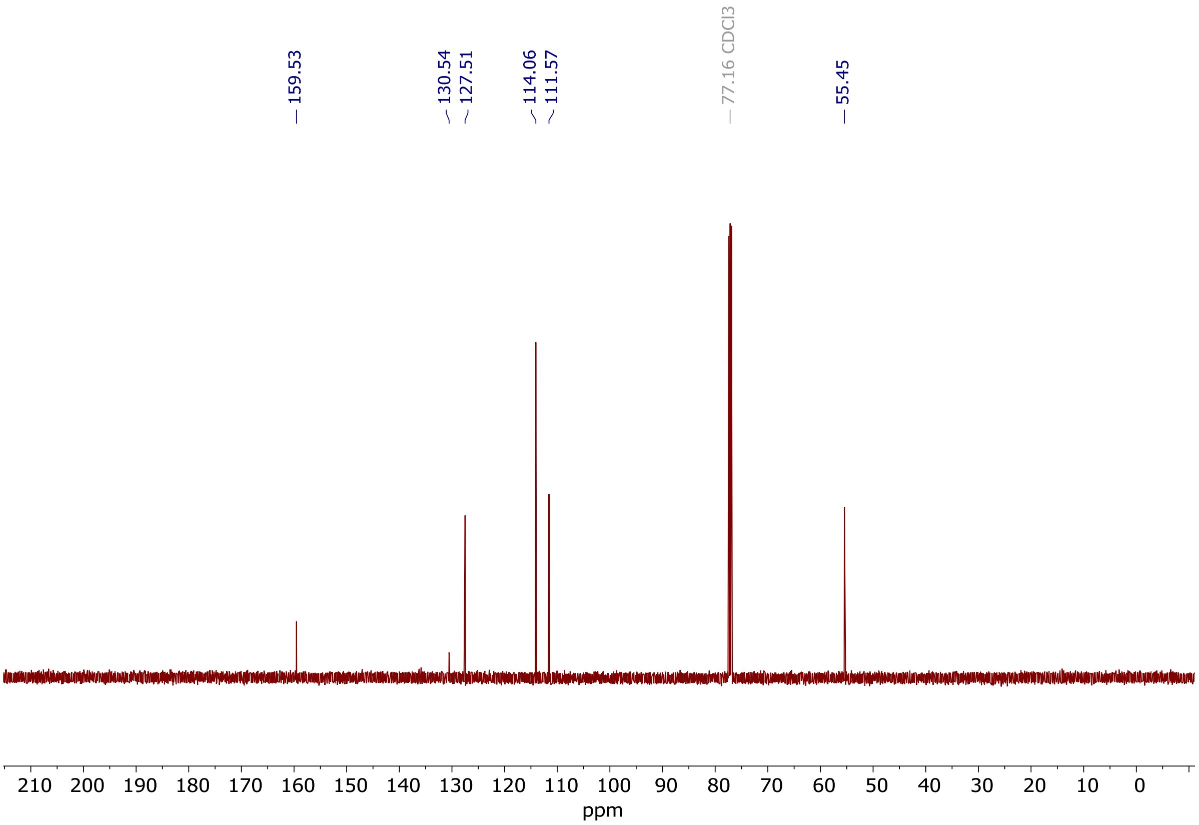

**$^{1}$H NMR** (400 MHz, $CDCl_3$) of **1s** see procedure

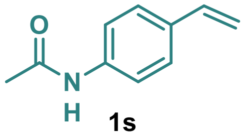


**1s**

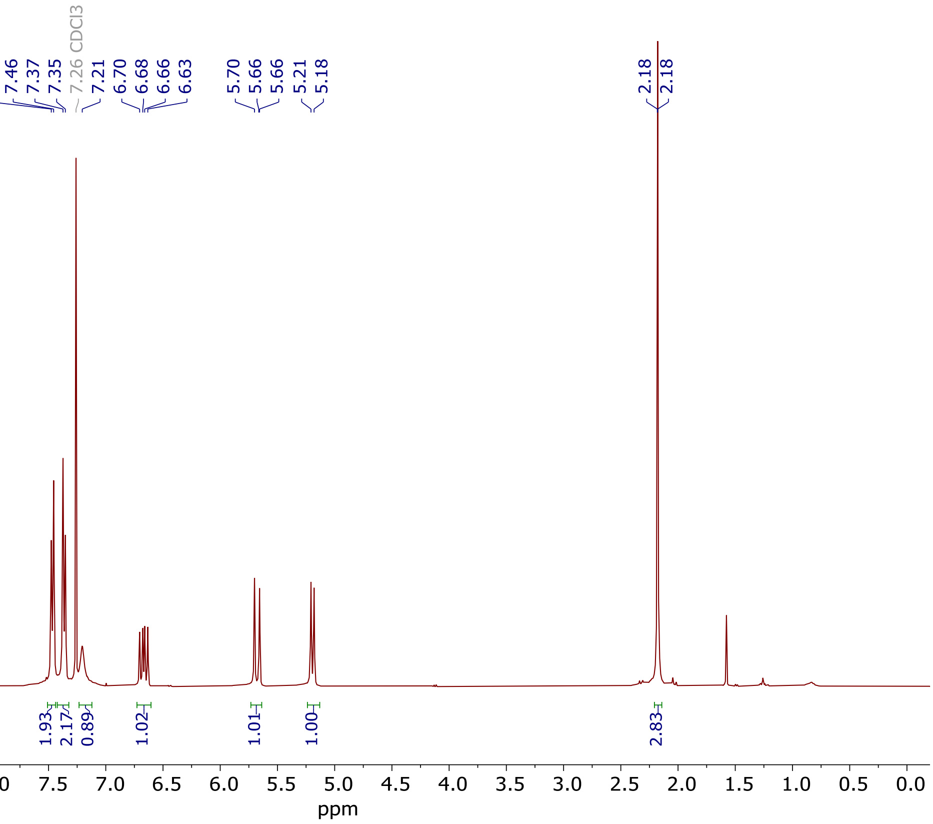


**$^{13}$C NMR** (101MHz, $CDCl_3$) of **1s**

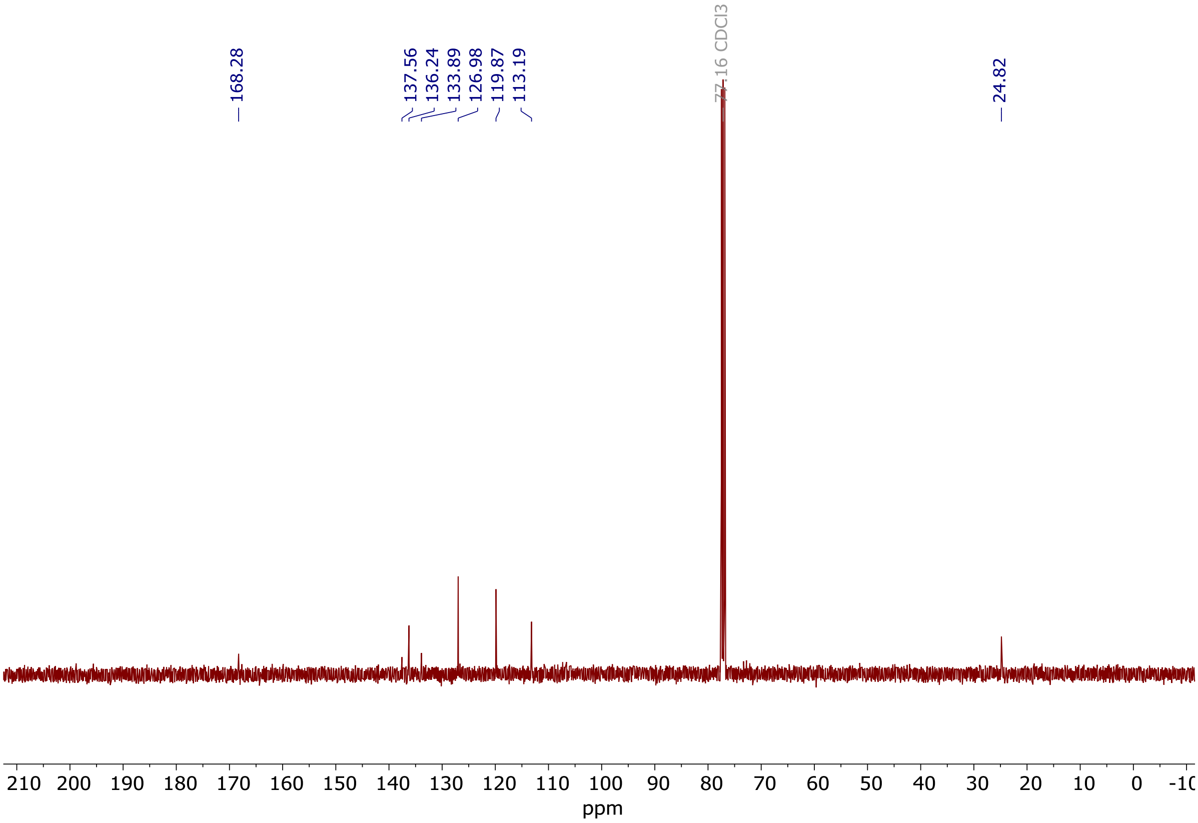

**$^{1}$H NMR** (400 MHz, $CDCl_3$) of **1t** see procedure

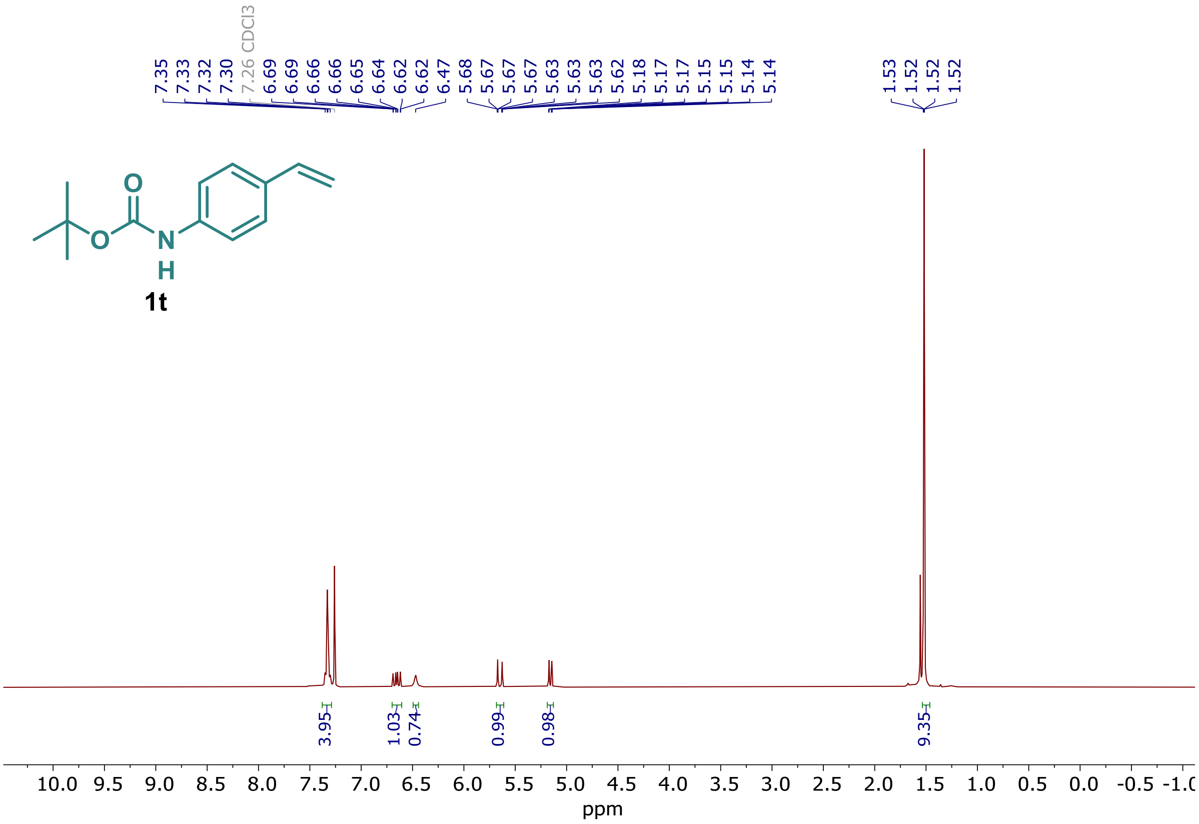


**$^{13}$C NMR** (101MHz, $CDCl_3$) of **1t**

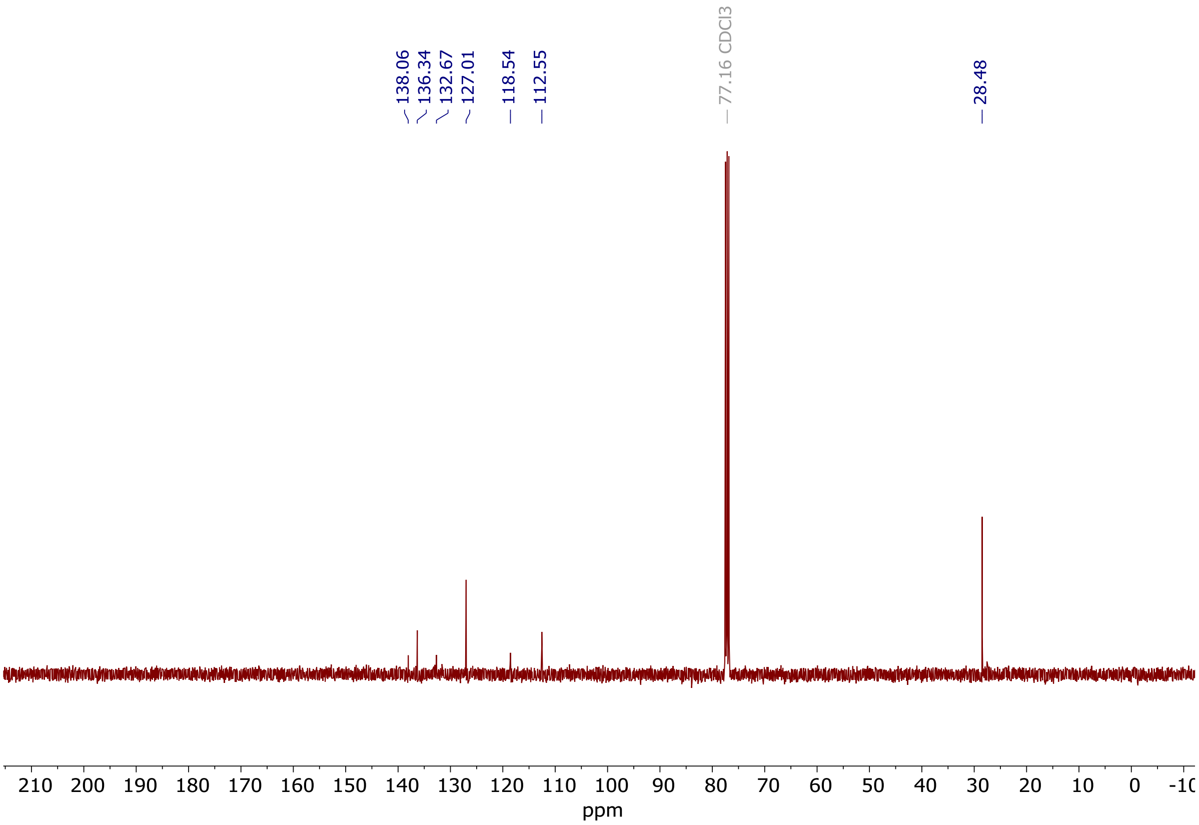

[1]**H NMR** (500 MHz, CDCl3) of **1y** see procedure

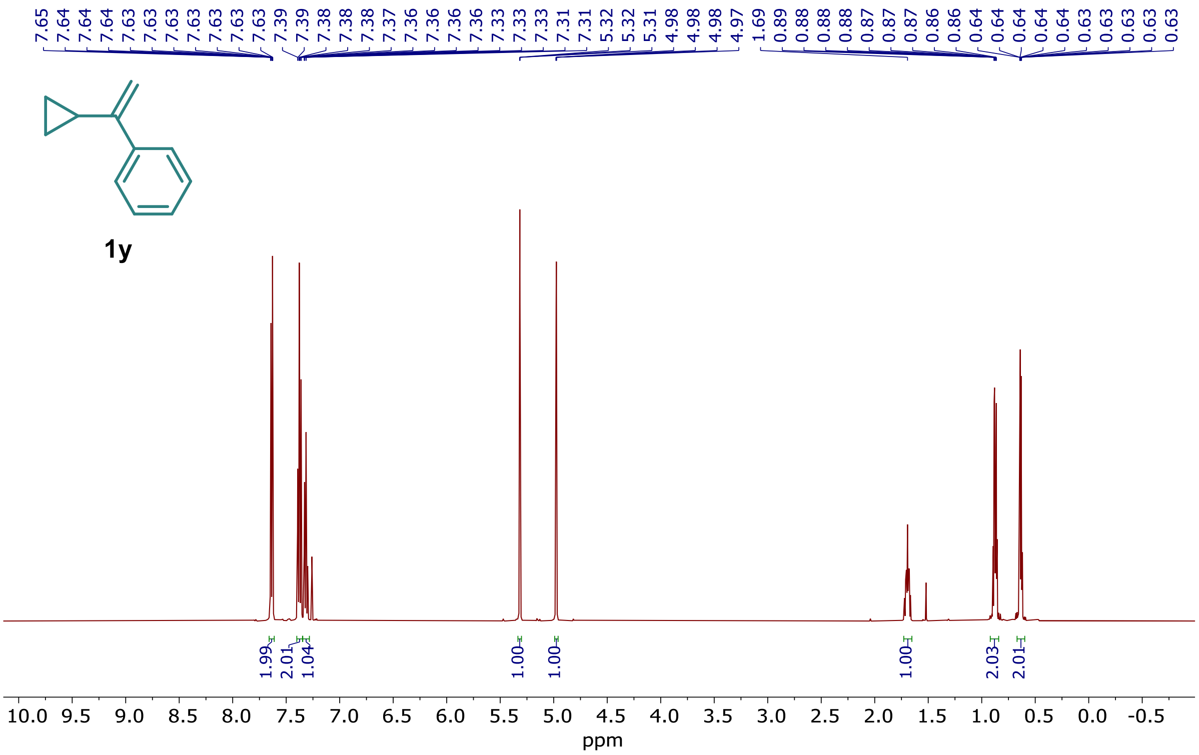


[13]**C NMR** (126MHz, CDCl3) of **1y**

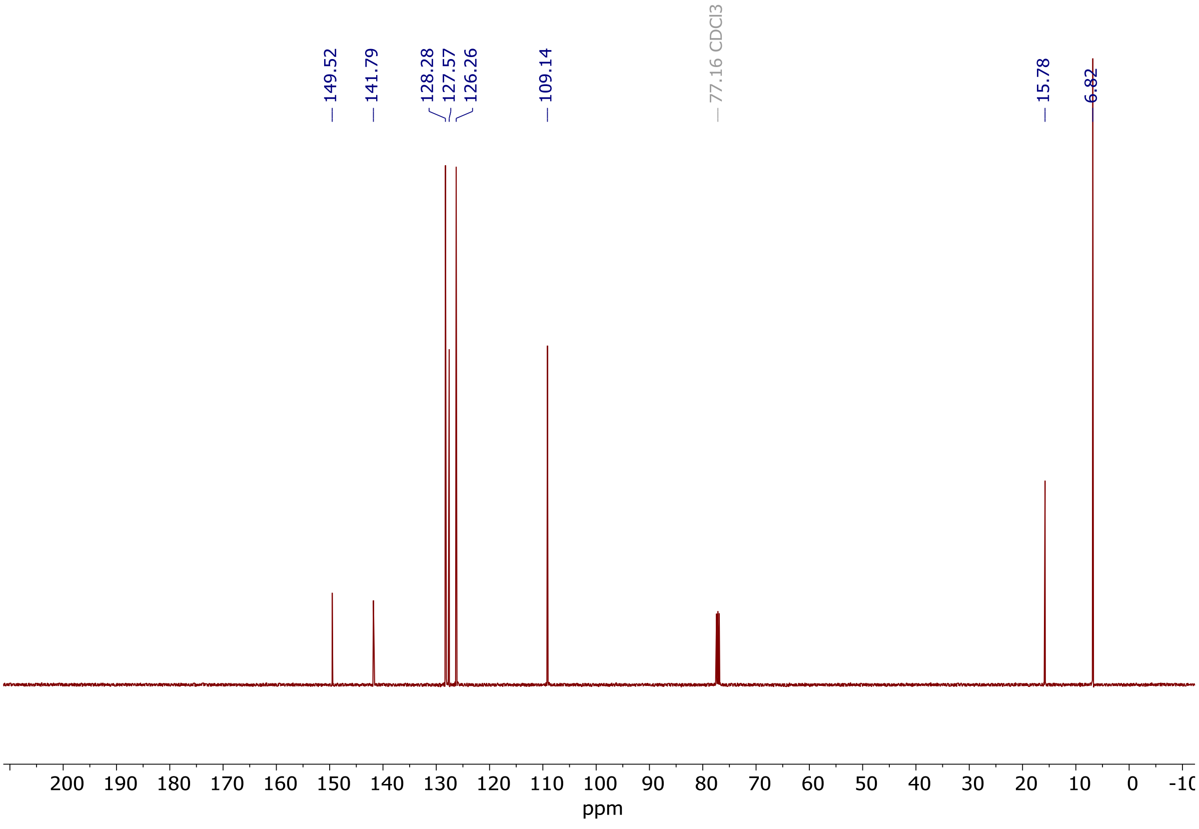

**$^{1}$H NMR** (400MHz, $CDCl_3$) of **1z** see procedure

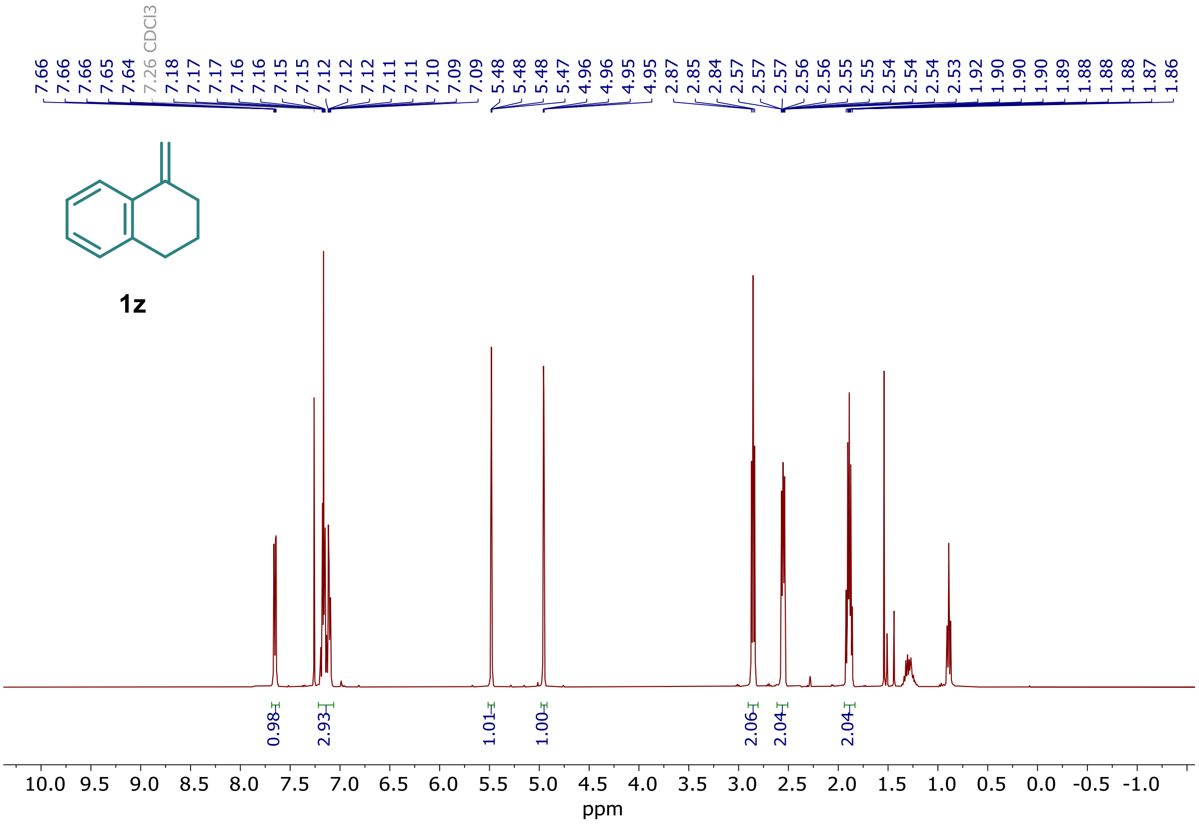


**$^{13}$C NMR** (101MHz, $CDCl_3$) of **1z**

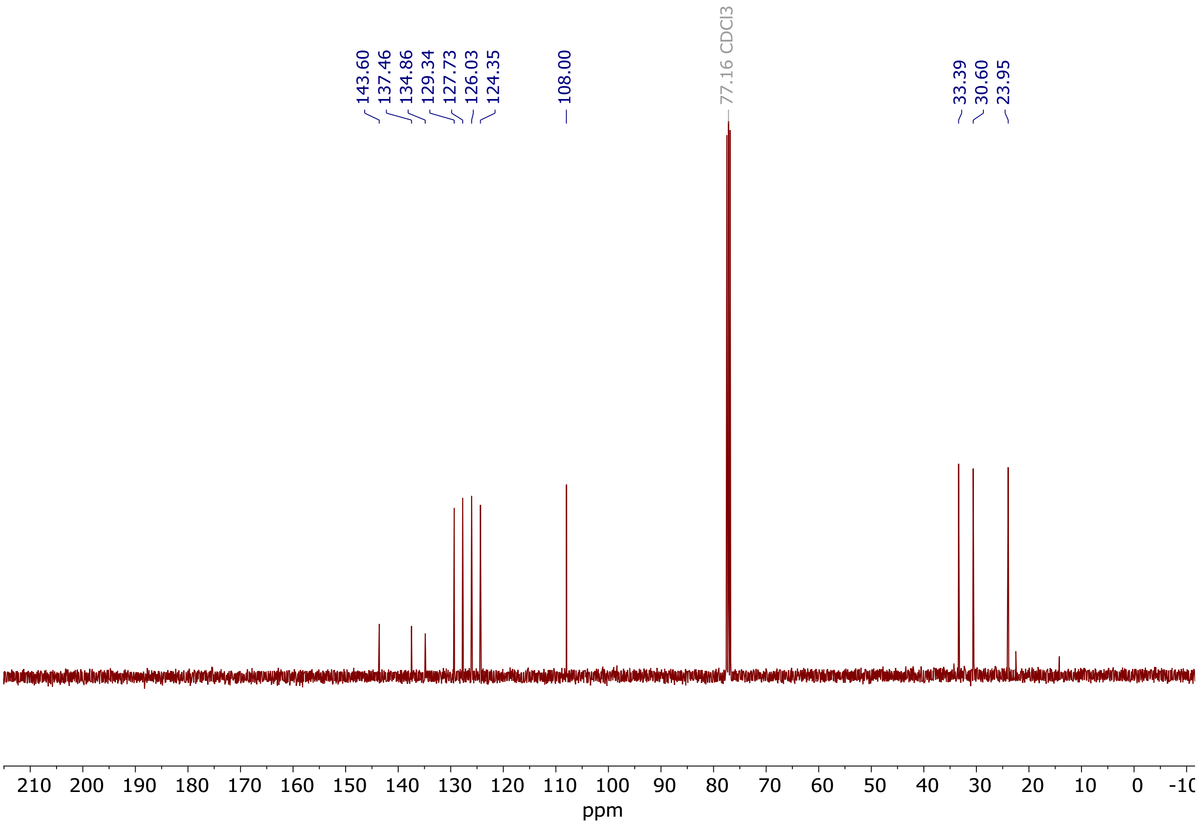

**$^{1}$H NMR** (400 MHz, $CDCl_3$) of **1aa** see procedure

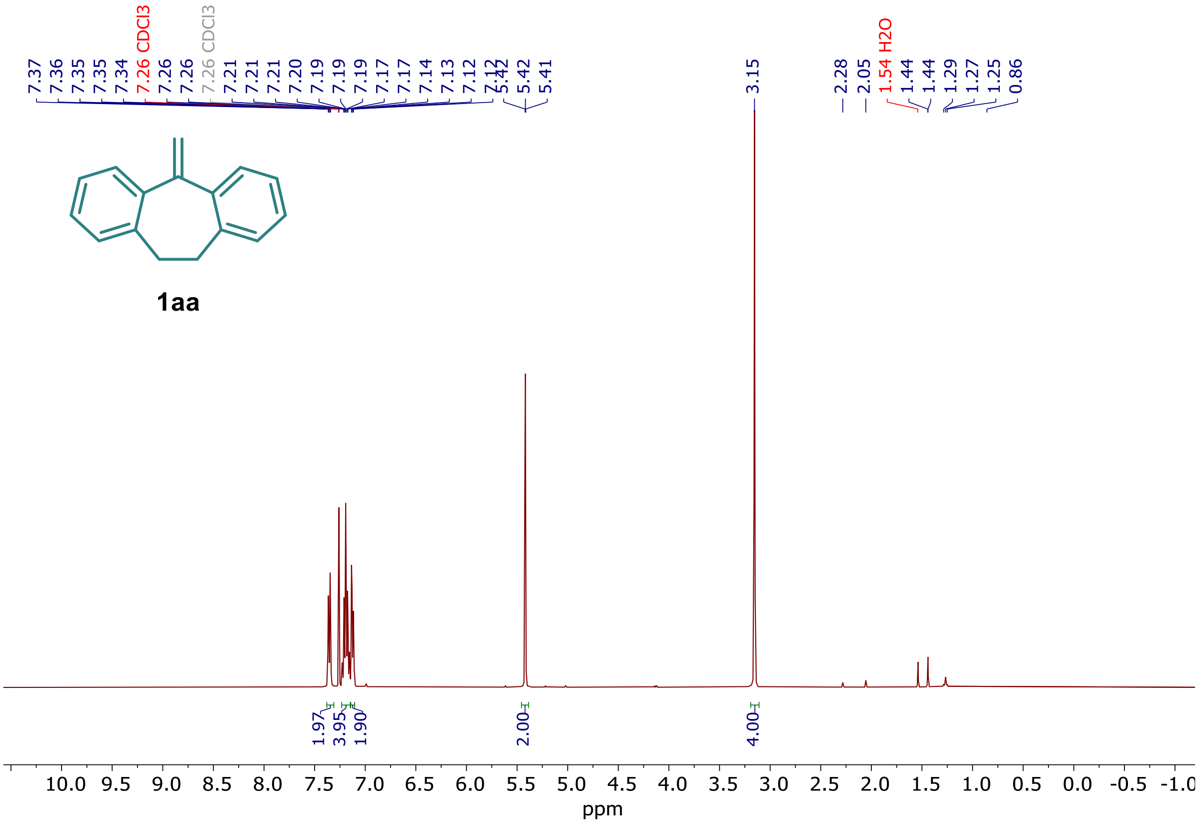


**$^{13}$C NMR** (101MHz, $CDCl_3$) of **1aa**

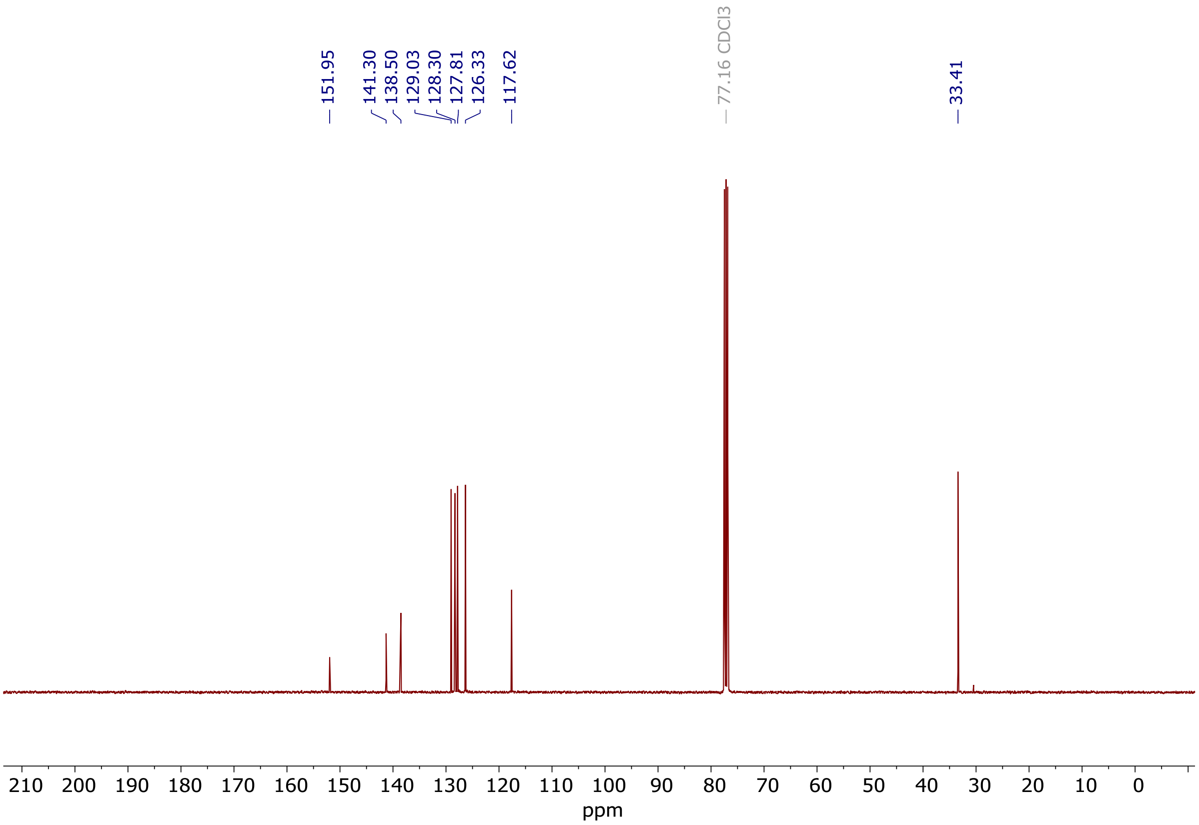

**¹H NMR** (400 MHz, $CDCl_3$) of **1ad** see procedure

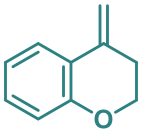

**1ad**

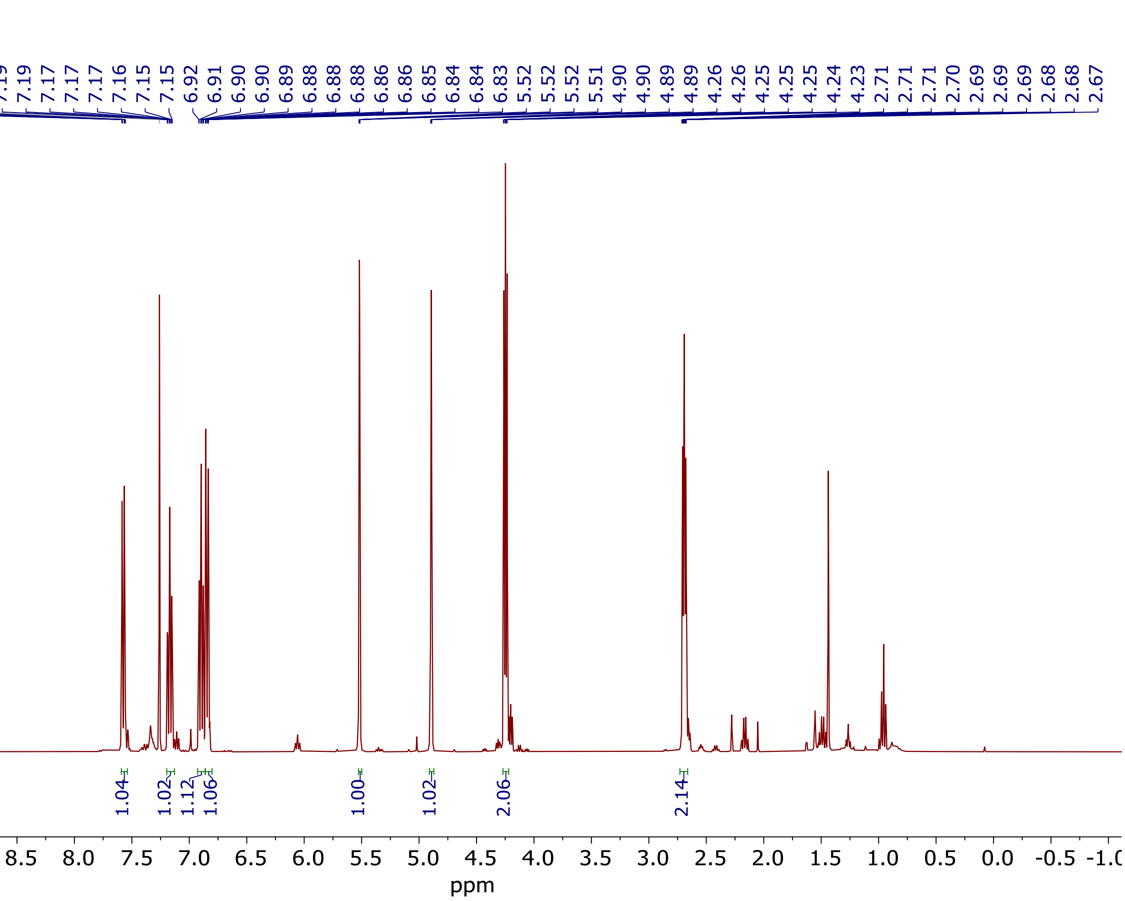

**$^{1}$H NMR** (400 MHz, $CDCl_3$) of **1ae** see procedure

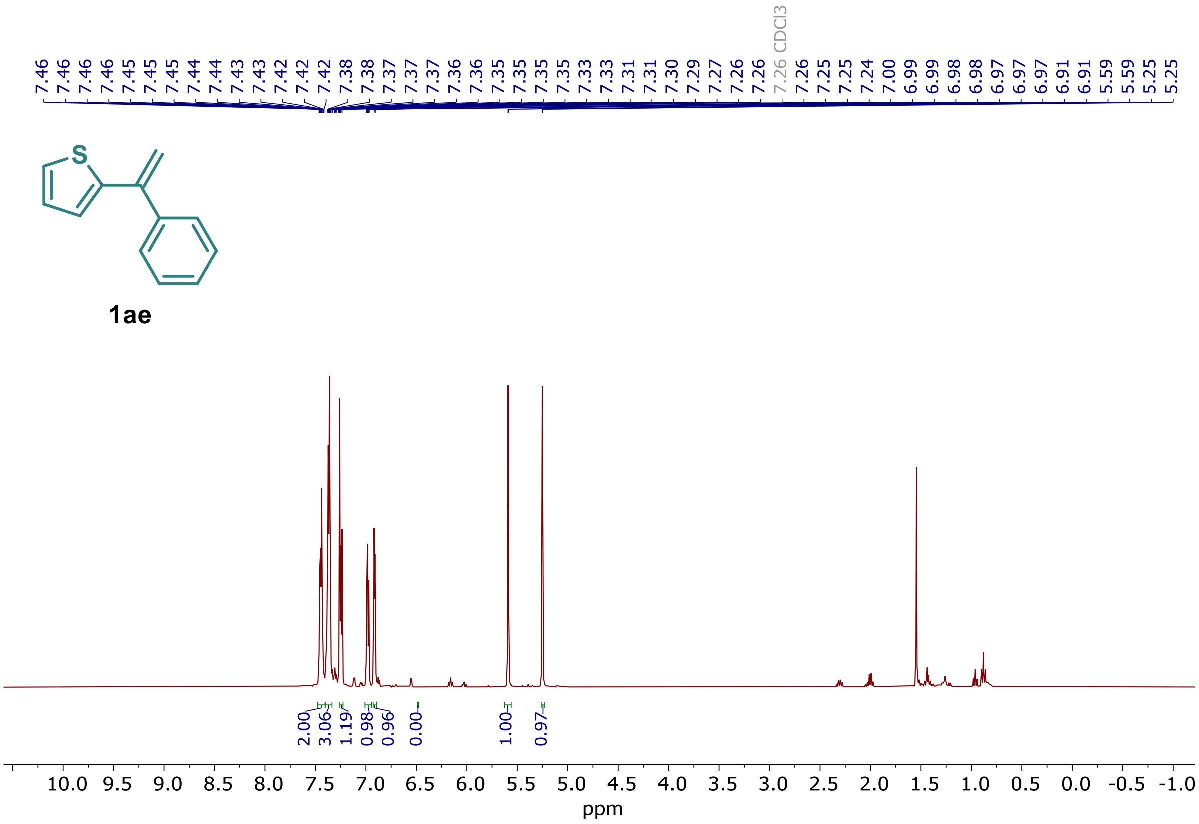


**$^{13}$C NMR** (101MHz, $CDCl_3$) of **1ae**

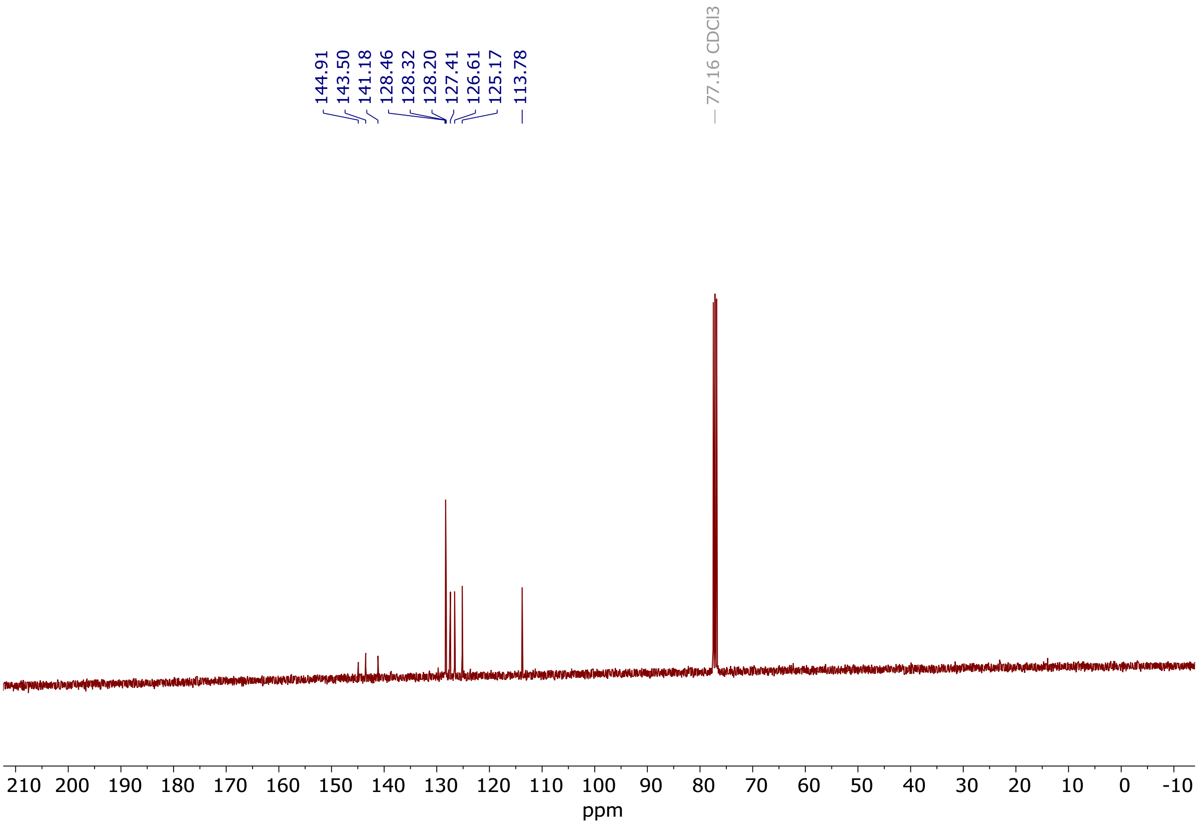

**$^{1}$H NMR** (400 MHz, $CDCl_3$) of **1ag** see procedure

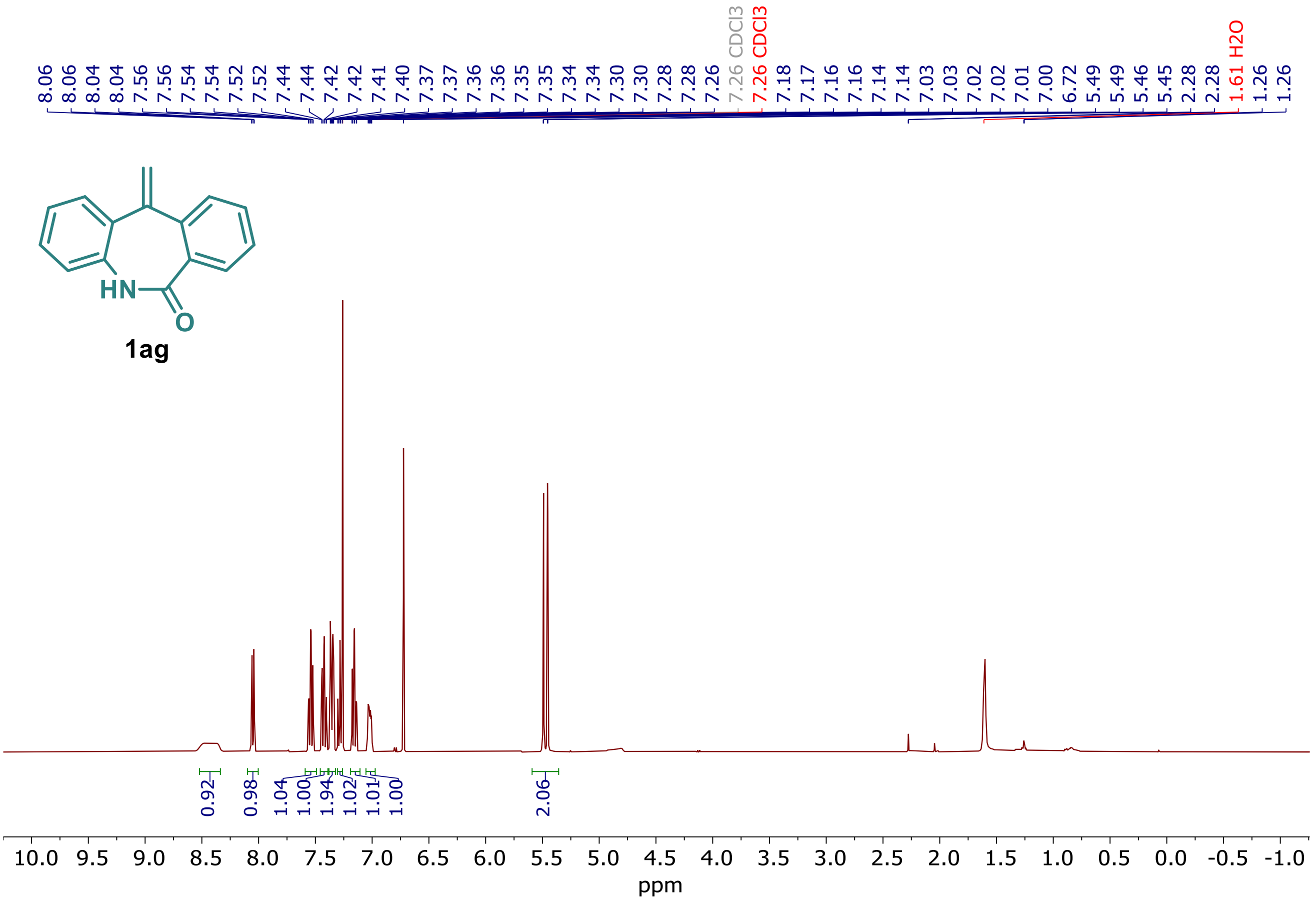


**$^{13}$C NMR** (101MHz, $CDCl_3$) of **1ag**

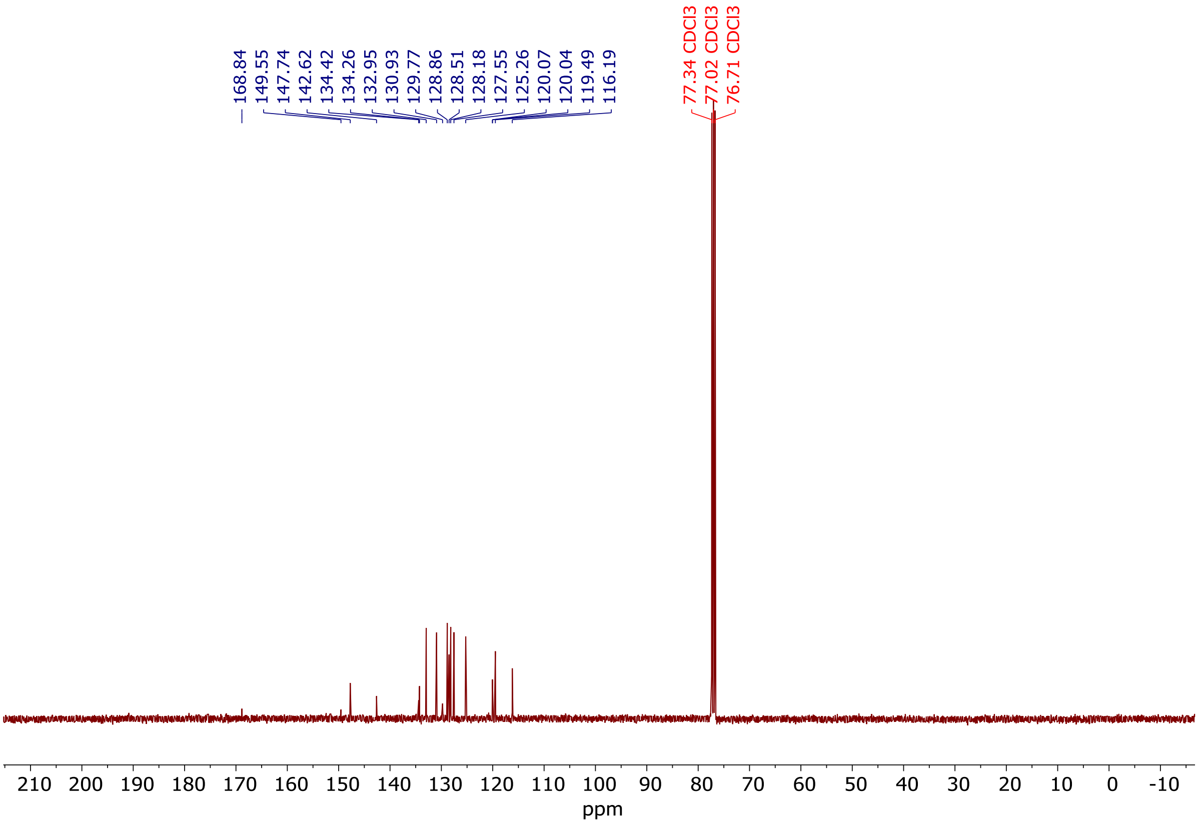

# 7. REFERENCES

(1) HepatoChem Inc. *EvoluChem™ LEDs*. https://hepatochem.com/photoreactors-leds-accessories/photoreactor-leds-evoluchem/ (accessed 2025-03-11).

(2) Griffith, J. C.; Jones, K. M.; Picon, S.; Rawling, M. J.; Kariuki, B. M.; Campbell, M.; Tomkinson, N. C. O. Alkene syn dihydroxylation with malonoyl peroxides. *J. Am. Chem. Soc.* **2010**, *132*, 14409–14411. DOI: 10.1021/ja1066674.

(3) Porter, J. E.; Schuster, G. B. Thermolysis of 4-methyl-4-phenylmalonyl peroxide: a new oxygen dependent chemiluminescent reaction. *J. Org. Chem.* **1983**, *48*, 4944–4947. DOI: 10.1021/jo00173a032.

(4) Strässler, C.; Linden, A.; Heimgartner, H. Novel Heterospirocyclic 3‐Amino-2*H*‐azirines as Synthons for Heterocyclic α‐Amino Acids. *Helv. Chim. Acta* **1997**, *80*, 1528–1554. DOI: 10.1002/hlca.19970800515.

(5) Luan, R.; Lin, P.; Li, K.; Du, Y.; Su, W. Remote-carbonyl-directed sequential Heck/isomerization/C($sp^2$)-H arylation of alkenes for modular synthesis of stereodefined tetrasubstituted olefins. *Nat. Commun.* **2024**, *15*, 1723. DOI: 10.1038/s41467-024-46051-y.

(6) Dalton, T.; Greßies, S.; Das, M.; Niehues, M.; Schrader, M. L.; Gutheil, C.; Ravoo, B. J.; Glorius, F. Silver-Catalysed Hydroarylation of Highly Substituted Styrenes. *Angew. Chem., Int. Ed.* **2021**, *60*, 8537–8541. DOI: 10.1002/anie.202016268.

(7) Yang, B.; Xu, X.-H.; Qing, F.-L. Copper-mediated radical 1,2-bis(trifluoromethylation) of alkenes with sodium trifluoromethanesulfinate. *Org. Lett.* **2015**, *17*, 1906–1909. DOI: 10.1021/acs.orglett.5b00601.

(8) Klauck, F. J. R.; Yoon, H.; James, M. J.; Lautens, M.; Glorius, F. Visible-Light-Mediated Deaminative Three-Component Dicarbofunctionalization of Styrenes with Benzylic Radicals. *ACS Catal.* **2019**, *9*, 236–241. DOI: 10.1021/acscatal.8b04191.

(9) Tyler, J. L.; Schäfer, F.; Shao, H.; Stein, C.; Wong, A.; Daniliuc, C. G.; Houk, K. N.; Glorius, F. Bicyclo1.1.0butyl Radical Cations: Synthesis and Application to 2π + 2σ Cycloaddition Reactions. *J. Am. Chem. Soc.* **2024**, *146*, 16237–16247. DOI: 10.1021/jacs.4c04403.

(10) Iwamoto, H.; Tsuruta, T.; Ogoshi, S. Development and Mechanistic Studies of (E )-Selective Isomerization/Tandem Hydroarylation Reactions of Alkenes with a Nickel(0)/Phosphine Catalyst. *ACS Catal.* **2021**, *11*, 6741–6749. DOI: 10.1021/acscatal.1c00908.

(11) Discekici, E. H.; Treat, N. J.; Poelma, S. O.; Mattson, K. M.; Hudson, Z. M.; Luo, Y.; Hawker, C. J.; Read de Alaniz, J. A highly reducing metal-free photoredox catalyst: design and application in radical dehalogenations. *Chem. Commun.* **2015**, *51*, 11705–11708. DOI: 10.1039/c5cc04677g.

(12) Yoshida, T.; Honda, Y.; Morofuji, T.; Kano, N. N-Methylphenothiazine S-Oxide Enabled Oxidative C($sp^2$)–C($sp^2$) Coupling of Boronic Acids with Organolithiums via Phenothiaziniums. *Org. Lett.* **2021**, *23*, 9664–9668. DOI: 10.1021/acs.orglett.1c03986.

(13) Tian, Y.; Yang, J.; Liu, Z.; Gao, M.; Li, X.; Che, W.; Fang, M.; Li, Z. Multistage Stimulus-Responsive Room Temperature Phosphorescence Based on Host-Guest Doping Systems. *Angew. Chem., Int. Ed.* **2021**, *60*, 20259–20263. DOI: 10.1002/anie.202107639.

(14) Huang, X.; Yao, Y.; Yin, X.; Guan, W.; Yuan, C.; Fang, Z.; Qin, H.; Liu, C.; Guo, K. Electro-oxidative quinylation of sulfides to sulfur ylides in batch and continuous flow. *iScience* **2024**, *27*, 108605. DOI: 10.1016/j.isci.2023.108605.

(15) Pitzer, L.; Schäfers, F.; Glorius, F. Rapid Assessment of the Reaction-Condition-Based Sensitivity of

Chemical Transformations. *Angew. Chem., Int. Ed.* **2019**, *58*, 8572–8576. DOI: 10.1002/anie.201901935.
(16) Collins, K. D.; Glorius, F. A robustness screen for the rapid assessment of chemical reactions. *Nat. Chem.* **2013**, *5*, 597–601. DOI: 10.1038/nchem.1669.
(17) (a) Vil', V. A.; Gorlov, E. S.; Bityukov, O. V.; Barsegyan, Y. A.; Romanova, Y. E.; Merkulova, V. M.; Terent'ev, A. O. C−O coupling of Malonyl Peroxides with Enol Ethers via [5+2] Cycloaddition: Non‐Rubottom Oxidation. *Adv. Synth. Catal.* **2019**, *361*, 3173–3181. DOI: 10.1002/adsc.201900271; (b) Vil', V. A.; Gorlov, E. S.; Bityukov, O. V.; Krylov, I. B.; Nikishin, G. I.; Pivnitsky, K. K.; Terent'ev, A. O. Oxidative C–O coupling as a new idea in the 'click-like chemistry': malonyl peroxides for the conjugation of two molecules. *Mendeleev Commun.* **2019**, *29*, 132–134. DOI: 10.1016/j.mencom.2019.03.003.
(18) Espinoza, E. M.; Clark, J. A.; Soliman, J.; Derr, J. B.; Morales, M.; Vullev, V. I. Practical Aspects of Cyclic Voltammetry: How to Estimate Reduction Potentials When Irreversibility Prevails. *J. Electrochem. Soc.* **2019**, *166*, H3175-H3187. DOI: 10.1149/2.0241905jes.
(19) Rawling, M. J.; Rowley, J. H.; Campbell, M.; Kennedy, A. R.; Parkinson, J. A.; Tomkinson, N. C. O. Mechanistic insights into the malonoyl peroxide syn-dihydroxylation of alkenes. *Chem. Sci.* **2014**, *5*, 1777–1785. DOI: 10.1039/C3SC53256A.
(20) Cismesia, M. A.; Yoon, T. P. Characterizing Chain Processes in Visible Light Photoredox Catalysis. *Chem. Sci.* **2015**, *6*, 5426–5434. DOI: 10.1039/C5SC02185E.
(21) A new sensitive chemical actinometer - II. Potassium ferrioxalate as a standard chemical actinometer. *Proc. R. Soc. Lond. A* **1956**, *235*, 518–536. DOI: 10.1098/rspa.1956.0102.
(22) Wegner, E. E.; Adamson, A. W. Photochemistry of Complex Ions. III. Absolute Quantum Yields for the Photolysis of Some Aqueous Chromium(III) Complexes. Chemical Actinometry in the Long Wavelength Visible Region. *J. Am. Chem. Soc.* **1966**, *88*, 394–404. DOI: 10.1021/ja00955a003.
(23) (a) Neese, F. Software update: The ORCA program system—Version 5.0. *Wiley Interdiscip. Rev.:Comput. Mol. Sci.* **2022**, *12*. DOI: 10.1002/wcms.1606; (b) Neese, F.; Olbrich, G. Efficient use of the resolution of the identity approximation in time-dependent density functional calculations with hybrid density functionals. *Chem. Phys. Lett.* **2002**, *362*, 170–178. DOI: 10.1016/s0009-2614(02)01053-9; (c) Neese, F. An improvement of the resolution of the identity approximation for the formation of the Coulomb matrix. *J. Comput. Chem.* **2003**, *24*, 1740–1747. DOI: 10.1002/jcc.10318; (d) Petrenko, T.; Kossmann, S.; Neese, F. Efficient time-dependent density functional theory approximations for hybrid density functionals: analytical gradients and parallelization. *J. Chem. Phys.* **2011**, *134*, 54116. DOI: 10.1063/1.3533441; (e) Neese, F.; Wennmohs, F.; Hansen, A.; Becker, U. Efficient, approximate and parallel Hartree–Fock and hybrid DFT calculations. A 'chain-of-spheres' algorithm for the Hartree–Fock exchange. *Chem. Phys.* **2009**, *356*, 98–109. DOI: 10.1016/j.chemphys.2008.10.036; (f) Helmich-Paris, B.; Souza, B. de; Neese, F.; Izsák, R. An improved chain of spheres for exchange algorithm. *J. Chem. Phys.* **2021**, *155*, 104109. DOI: 10.1063/5.0058766; (g) Neese, F. The SHARK integral generation and digestion system. *J. Comput. Chem.* **2023**, *44*, 381–396. DOI: 10.1002/jcc.26942.
(24) Hanwell, M. D.; Curtis, D. E.; Lonie, D. C.; Vandermeersch, T.; Zurek, E.; Hutchison, G. R. Avogadro: an advanced semantic chemical editor, visualization, and analysis platform. *J. Cheminf.* **2012**, *4*, 17. DOI: 10.1186/1758-2946-4-17.
(25) *Chemcraft - graphical software for visualization of quantum chemistry computations.* https://

www.chemcraftprog.com/.
(26) Bannwarth, C.; Ehlert, S.; Grimme, S. GFN2-xTB-An Accurate and Broadly Parametrized Self-Consistent Tight-Binding Quantum Chemical Method with Multipole Electrostatics and Density-Dependent Dispersion Contributions. *J. Chem. Theory Comput.* **2019**, *15*, 1652–1671. DOI: 10.1021/acs.jctc.8b01176.
(27) Chai, J.-D.; Head-Gordon, M. Long-range corrected hybrid density functionals with damped atom-atom dispersion corrections. *Phys. Chem. Chem. Phys.* **2008**, *10*, 6615–6620. DOI: 10.1039/B810189B.
(28) Weigend, F.; Ahlrichs, R. Balanced basis sets of split valence, triple zeta valence and quadruple zeta valence quality for H to Rn: Design and assessment of accuracy. *Phys. Chem. Chem. Phys.* **2005**, *7*, 3297–3305. DOI: 10.1039/B508541A.
(29) Grimme, S.; Antony, J.; Ehrlich, S.; Krieg, H. A consistent and accurate ab initio parametrization of density functional dispersion correction (DFT-D) for the 94 elements H-Pu. *J. Chem. Phys.* **2010**, *132*, 154104. DOI: 10.1063/1.3382344.
(30) Barone, V.; Cossi, M. Quantum Calculation of Molecular Energies and Energy Gradients in Solution by a Conductor Solvent Model. *J. Phys. Chem. A* **1998**, *102*, 1995–2001. DOI: 10.1021/jp9716997.
(31) Kossmann, S.; Neese, F. Efficient Structure Optimization with Second-Order Many-Body Perturbation Theory: The RIJCOSX-MP2 Method. *J. Chem. Theory Comput.* **2010**, *6*, 2325–2338. DOI: 10.1021/ct100199k.
(32) Yanai, T.; Tew, D. P.; Handy, N. C. A new hybrid exchange–correlation functional using the Coulomb-attenuating method (CAM-B3LYP). *Chem. Phys. Lett.* **2004**, *393*, 51–57. DOI: 10.1016/j.cplett.2004.06.011.
(33) Roth, H.; Romero, N.; Nicewicz, D. Experimental and Calculated Electrochemical Potentials of Common Organic Molecules for Applications to Single-Electron Redox Chemistry. *Synlett* **2016**, *27*, 714–723. DOI: 10.1055/s-0035-1561297.
(34) Popescu, M. V.; Paton, R. S. Dynamic vertical triplet energies: Understanding and predicting triplet energy transfer. *Chem* **2024**, *10*, 3428–3443. DOI: 10.1016/j.chempr.2024.07.001.
(35) Zhao, Y.; Truhlar, D. G. The M06 suite of density functionals for main group thermochemistry, thermochemical kinetics, noncovalent interactions, excited states, and transition elements: two new functionals and systematic testing of four M06-class functionals and 12 other functionals. *Theor. Chem. Acc.* **2008**, *120*, 215–241. DOI: 10.1007/s00214-007-0310-x.
(36) M. J. Frisch, G. W. Trucks, H. B. Schlegel, G. E. Scuseria, M. A. Robb, J. R. Cheeseman, G. Scalmani, V. Barone, G. A. Petersson, H. Nakatsuji, X. Li, M. Caricato, A. V. Marenich, J. Bloino, B. G. Janesko, R. Gomperts, B. Mennucci, H. P. Hratchian, J. V. Ortiz, A. F. Izmaylov, J. L. Sonnenberg, D. Williams-Young, F. Ding, F. Lipparini, F. Egidi, J. Goings, B. Peng, A. Petrone, T. Henderson, D. Ranasinghe, V. G. Zakrzewski, J. Gao, N. Rega, G. Zheng, W. Liang, M. Hada, M. Ehara, K. Toyota, R. Fukuda, J. Hasegawa, M. Ishida, T. Nakajima, Y. Honda, O. Kitao, H. Nakai, T. Vreven, K. Throssell, J. A. Montgomery, Jr., J. E. Peralta, F. Ogliaro, M. J. Bearpark, J. J. Heyd, E. N. Brothers, K. N. Kudin, V. N. Staroverov, T. A. Keith, R. Kobayashi, J. Normand, K. Raghavachari, A. P. Rendell, J. C. Burant, S. S. Iyengar, J. Tomasi, M. Cossi, J. M. Millam, M. Klene, C. Adamo, R. Cammi, J. W. Ochterski, R. L. Martin, K. Morokuma, O. Farkas, J. B. Foresman, D. J. Fox. *Gaussian 16, Revision C.01*, 2016.
(37) M. S. Teynor, N. Wohlgemuth, L. Carlson, J. Huang, S. L. Pugh, B. O. Grant, R. S. Hamilton, R. Carlsen, and D. H. Ess. *Milo, Revision 1.0.3*, 2021.

(38) Easton, R. E.; Giesen, D. J.; Welch, A.; Cramer, C. J.; Truhlar, D. G. The MIDI! basis set for quantum mechanical calculations of molecular geometries and partial charges. *Theor. Chem. Acc.* **1996**, *93*, 281–301. DOI: 10.1007/BF01127507.

(39) Hunter, J. D. Matplotlib: A 2D Graphics Environment. *Comput. Sci. Eng.* **2007**, *9*, 90–95. DOI: 10.1109/MCSE.2007.55.

(40) Fabian Pedregosa, Gaël Varoquaux, Alexandre Gramfort, Vincent Michel, Bertrand Thirion, Olivier Grisel, Mathieu Blondel, Peter Prettenhofer, Ron Weiss, Vincent Dubourg, Jake Vanderplas, Alexandre Passos, David Cournapeau, Matthieu Brucher, Matthieu Perrot, Édouard Duchesnay. Scikit-learn: Machine Learning in Python. *J. Mach. Learn. Res.* **2011**, 2825–2830.

(41) Bruker AXS. *APEX4 Version 2021.4-0, SAINT Version 8.40B and SADABS Bruker AXS area detector scaling and absorption correction Version 2016/2*, 2021.

(42) Sheldrick, G. M. SHELXT - integrated space-group and crystal-structure determination. *Acta Crystallogr., Sect. A: Found. Adv.* **2015**, *71*, 3–8. DOI: 10.1107/S2053273314026370.

(43) Sheldrick, G. M. Crystal structure refinement with SHELXL. *Acta Crystallogr., Sect. C:Struct. Chem.* **2015**, *71*, 3–8. DOI: 10.1107/S2053229614024218.

(44) Bruker AXS. *XP – Interactive molecular graphics, Version 5.1*, 1998.
